# [Ni(NHC)_2_] as a Scaffold for Structurally Characterized *trans* [H−Ni−PR_2_] and *trans* [R_2_P−Ni−PR_2_] Complexes

**DOI:** 10.1002/chem.202101484

**Published:** 2021-08-04

**Authors:** Sara Sabater, David Schmidt, Heidi (née Schneider) Schmidt, Maximilian W. Kuntze‐Fechner, Thomas Zell, Connie J. Isaac, Nasir A. Rajabi, Harry Grieve, William J. M. Blackaby, John P. Lowe, Stuart A. Macgregor, Mary F. Mahon, Udo Radius, Michael K. Whittlesey

**Affiliations:** ^1^ Department of Chemistry University of Bath Claverton Down Bath BA2 7AY UK; ^2^ Institut für Anorganische Chemie Julius-Maximilians-Universität Würzburg Am Hubland 97074 Würzburg Germany; ^3^ Institute of Chemical Sciences Heriot-Watt University Edinburgh EH14 4AS UK

**Keywords:** carbene ligands, density functional calculations, hydride ligands, nickel, phosphido ligands

## Abstract

The addition of PPh_2_H, PPhMeH, PPhH_2_, P(*para*‐Tol)H_2_, PMesH_2_ and PH_3_ to the two‐coordinate Ni^0^ N‐heterocyclic carbene species [Ni(NHC)_2_] (NHC=I*i*Pr_2_, IMe_4_, IEt_2_Me_2_) affords a series of mononuclear, terminal phosphido nickel complexes. Structural characterisation of nine of these compounds shows that they have unusual *trans* [H−Ni−PR_2_] or novel *trans* [R_2_P−Ni−PR_2_] geometries. The bis‐phosphido complexes are more accessible when smaller NHCs (IMe_4_>IEt_2_Me_2_>I*i*Pr_2_) and phosphines are employed. P−P activation of the diphosphines R_2_P−PR_2_ (R_2_=Ph_2_, PhMe) provides an alternative route to some of the [Ni(NHC)_2_(PR_2_)_2_] complexes. DFT calculations capture these trends with P−H bond activation proceeding from unconventional phosphine adducts in which the H substituent bridges the Ni−P bond. P−P bond activation from [Ni(NHC)_2_(Ph_2_P−PPh_2_)] adducts proceeds with computed barriers below 10 kcal mol^−1^. The ability of the [Ni(NHC)_2_] moiety to afford isolable terminal phosphido products reflects the stability of the Ni−NHC bond that prevents ligand dissociation and onward reaction.

## Graphical abstract

**Whole lotta terminal phosphidos**: A combined experimental and computational study of the reactivity of [Ni(NHC)_2_] (NHC=N‐heterocyclic carbene) towards secondary and primary phosphines has been undertaken. P−H bond activation, as well as P−P activation of diphosphines, yields an extensive series of structurally characterised terminal phosphido complexes that exhibit unusual *trans*‐phosphido hydrido and novel *trans*‐bis‐phosphido geometries.

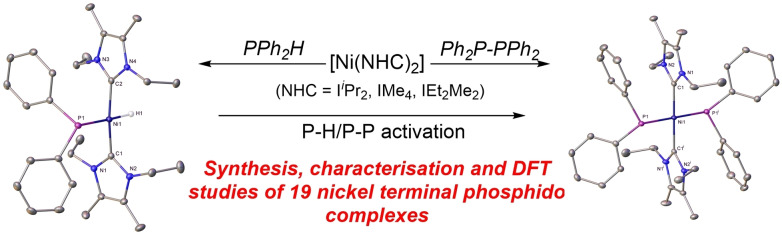

## Introduction

**Scheme 1:**
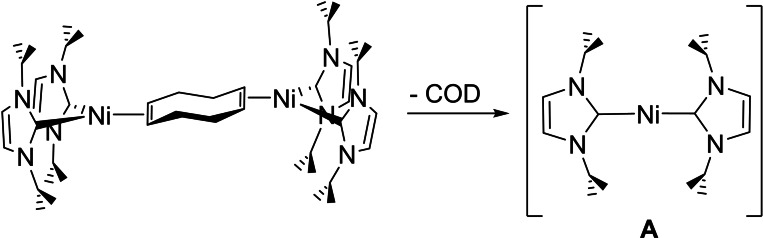
[Ni_2_(I*i*Pr_2_)_4_(COD)] as a labile source of [Ni(I*i*Pr_2_)_2_] (**A**).

Isolation of the 14‐electron nickel(0) bis‐N‐heterocyclic carbene (NHC) complex, [Ni(IMes)_2_] (IMes=1,3‐bis(2,4,6‐trimethylphenyl)imidazolin‐2‐ylidene), by Arduengo and co‐workers in 1994 provided an early illustration of the ability of NHCs to stabilize coordinatively unsaturated transition metal complexes.^[1]^ In the case of [Ni(IMes)_2_] and analogues containing even more bulky N‐aryl substituents,[^2^, ^3^] however, a price is paid for this stability in the form of somewhat altered and limited reactivity.[^4^, ^5^] [Ni(NHC)_2_] systems with higher reactivity towards small molecules^[6]^ are accessible through the use of N‐alkyl substituted carbenes, although with the exception of [Ni(I*t*Bu_2_)_2_] (I*t*Bu_2_=1,3‐di‐*tert*‐butylimidazolin‐2‐ylidene)[^2b^, ^7^] these can only be generated in situ. Particular success has been found with [Ni_2_(I*i*Pr_2_)_4_(COD)] (I*i*Pr_2_=1,3‐di‐*iso*propylimidazolin‐2‐ylidene;^[8]^ and related molecules)^[9]^ as a labile source of the N‐isopropyl substituted carbene complex [Ni(I*i*Pr_2_)_2_] (**A**, Scheme 1), which is able to activate a wide range of C−X (X=F, Cl, Br),[^10^, ^11^] S−X (X=C, S)^[12]^ and E−H (E=Si, S) bonds.[^12^, ^13^] In a number of cases, these processes have been made catalytic, allowing the Suzuki‐Miyaura cross‐coupling of aryl fluorides, chlorides and bromides,[^11^, ^14^] hydrodefluorination of aryl fluorides^[15]^ and silane dehydropolymerisation.^[16]^ An even more impressive transformation builds upon the ability of **A** to cleave strained C−C bonds,^[17]^ allowing the coupling of 2,2′‐biphenylene with PhC≡CPh to yield 9,10‐diphenylphenanthrene.^[8]^

**Scheme 2:**
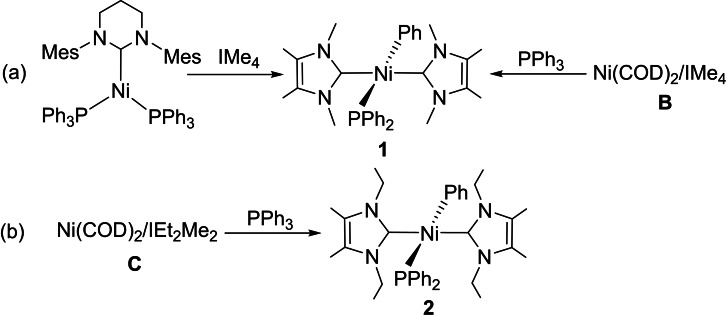
Syntheses of the [Ni(NHC)_2_(PPh_2_)Ph] complexes **1** (NHC = IMe_4_) and **2** (NHC = IEt_2_Me_2_).

Very recently, one of us reported isolation of the Ni(II) terminal phosphido phenyl complex, *trans*‐[Ni(IMe_4_)_2_(PPh_2_)Ph], (**1**, Scheme 2) from the reaction of the N‐methyl‐substituted NHC IMe_4_ (IMe_4_=1,3,4,5‐tetramethylimidazolin‐2‐ylidene) with the 16‐electron Ni(0) complex [Ni(6‐Mes)(PPh_3_)_2_] (6‐Mes=1,3‐bis(2,4,6‐trimethylphenyl)‐3,4,5,6‐tetrahydropyrimidin‐2‐ylidene). The formation of **1**, most likely through P−C activation of PPh_3_ by [Ni(IMe_4_)_2_] (**B**),^[18]^ is surprising given that P−C cleavage reactions of aryl phosphines typically yield multi‐metallic products with bridging phosphido ligands, rather than mononuclear complexes with terminal phosphido groups.^[19]^ Moreover, the isolation and structural characterization of **1** contrasts with the instability of the bis‐triethylphosphine analogue, *trans*‐[Ni(PEt_3_)_2_(PPh_2_)Ph], which has been reported as being too labile even to allow spectroscopic characterization.^[20]^

Our mutual interests in the bond activation reactions of **A** and **B** have now led us to conduct a joint investigation into their reactivity, together with that of the N−Et‐substituted derivative [Ni(IEt_2_Me_2_)_2_] (**C**; IEt_2_Me_2_=1,3‐diethyl‐4,5‐dimethylimidazolin‐2‐ylidene), towards P−H bonds of secondary and primary phosphines. At the outset of this work, we were aware of only a few examples of mononuclear nickel complexes with terminal phosphido ligands,[^21^, ^22^] and a single case of an isolated Ni phosphido hydrido complex, [Ni(d*t*bpe){P(Dmp)H}H)] (d*t*bpe=*t*Bu_2_PCH_2_CH_2_P*t*Bu_2_; Dmp=2,6‐Mes_2_C_6_H_3_).^[23]^ Herein, we describe the use of **A**–**C** to generate terminal phosphido hydrido and terminal bis‐phosphido complexes, nine of which have been structurally characterized.^[24]^ Among these are the first examples of terminal bis‐phosphido complexes with *trans* R_2_P−M−PR_2_ geometries reported for any transition metal. DFT calculations have also been used to rationalize the formation of the terminal phosphido hydrido and terminal bis‐phosphido complexes as a function of the NHCs involved.

## Results and Discussion

### In‐situ generation of [Ni(IMe_4_)_2_] (B) and [Ni(IEt_2_Me_2_)_2_] (C)

Given the atom inefficiency associated with the formation of **1** from [Ni(6‐Mes)(PPh_3_)_2_] and IMe_4_ (i. e., loss of both 6‐Mes and PPh_3_ during reaction), a more economical pathway was afforded by simply mixing [Ni(COD)_2_] with IMe_4_ and PPh_3_ in a 1 : 2 : 1 molar ratio at room temperature. This gave **1** (Scheme 2a) in about 90 % yield based on NMR spectroscopy. The *N*‐ethyl carbene derivative, *trans*‐[Ni(IEt_2_Me_2_)_2_(PPh_2_)Ph] (**2**), could be prepared similarly (Scheme 2b), again presumably through activation of a P−Ph group by [Ni(IEt_2_Me_2_)_2_] formed in situ. The combination of the N‐*i*Pr substituted NHC I*i*Pr_2_Me_2_ (I*i*Pr_2_Me_2_=1,3‐di‐*iso*propyl‐4,5‐dimethylimidazol‐2‐ylidene) with [Ni(COD)_2_] and PPh_3_ gave a mixture of uncharacterized products. The formation of **1** and **2** was quite slow, taking days to reach completion. In the case of **2**, raising the temperature to 50 °C reduced the reaction time to just 3 h. Complex **2**, which was isolated in 45 % yield, was readily characterizable by comparison of spectroscopic data to those of **1**, with a high frequency ^13^C{^1^H} NMR resonance at *δ*=166 ppm (d, ^2^ *J*(C,P)=40 Hz) diagnostic of the *ipso*‐C of the diphenylphosphido ligand.

**Figure 1:**
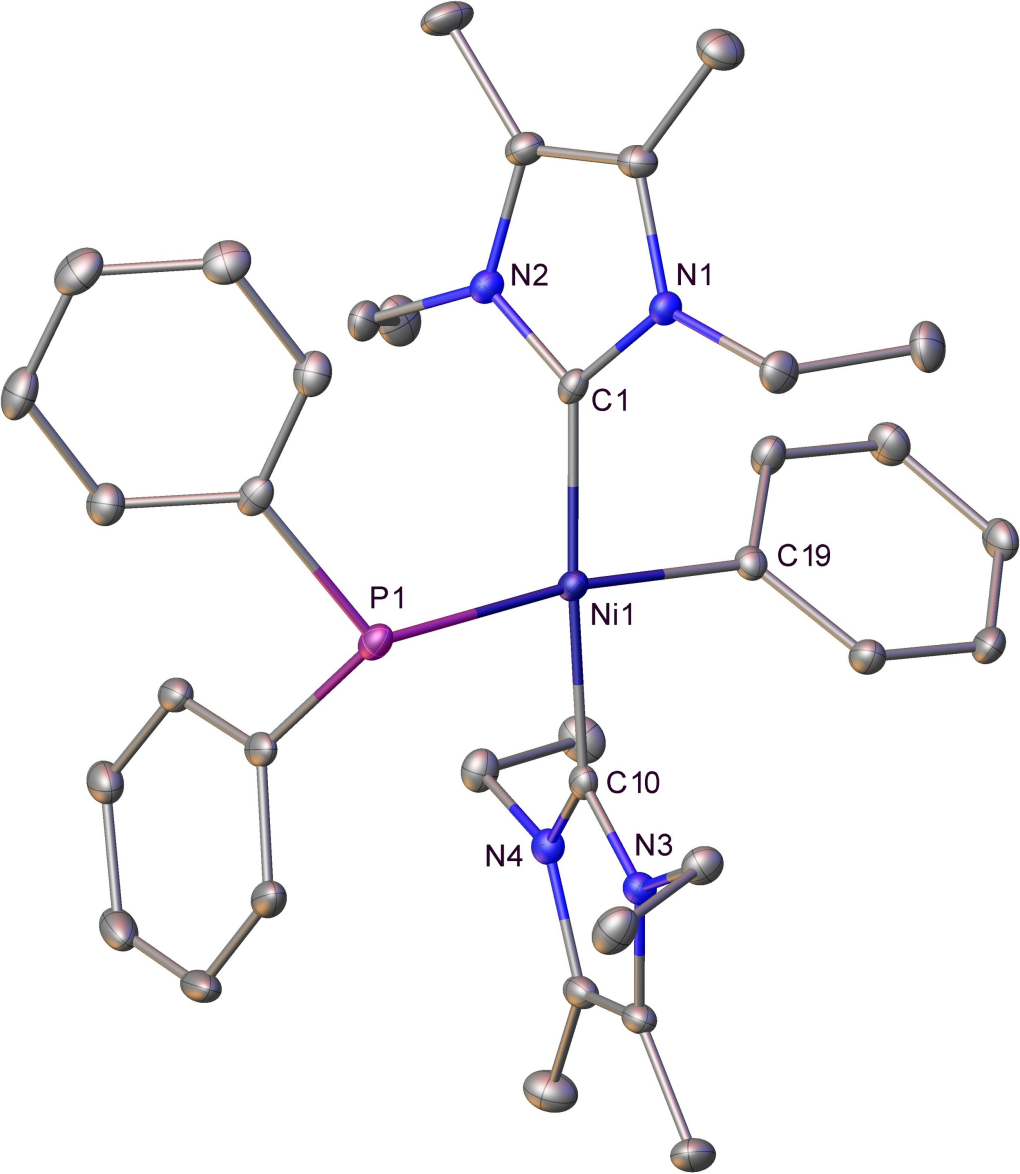
Molecular structure of *trans*‐[Ni(IEt_2_Me_2_)(PPh_2_)Ph] (**2**). Ellipsoids are shown at 30 % probability with all hydrogen atoms removed for clarity.

The molecular structure of **2** (Figure 1) confirmed the presence of a pyramidal phosphido ligand (Σ(angles at P)=325.4°; cf. 328.5° for a tetrahedral P) and a *trans* Ph−Ni−PPh_2_ geometry. In comparison to **1**, there was a slight elongation of both the Ni−C_phenyl_ and Ni−PPh_2_ distances (**2**: 1.960(3) Å, 2.2851(8) Å; **1**: 1.9371(15), 2.2520(5) Å). We attribute this to increased crowding at the nickel centre resulting from the less coplanar arrangement of the two NHC ligands in **2** (N−C−C−N dihedral angles: **2**: 142.3°, 161.6°; **1**: 159.2°, 156.7°). As will become obvious over the course of the manuscript, sterics plays a key role not only in defining the products formed between [Ni(NHC)_2_] and specific phosphines, but also their geometries.

### [Ni(NHC)_2_(PR_2_)H] formation with secondary phosphines

**Scheme 3:**
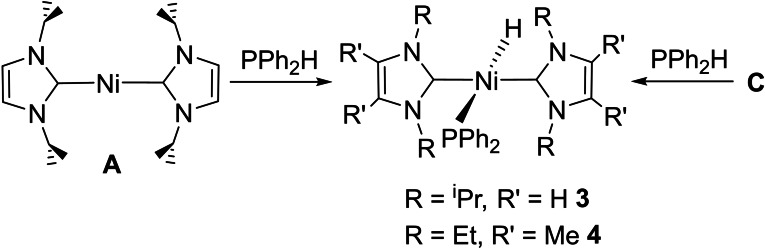
Formation of the phosphido hydrido complexes **3** and **4**.

Upon addition of the secondary aryl phosphine PPh_2_H to [Ni(I*i*Pr_2_)_2_] (**A**) or [Ni(IEt_2_Me_2_)_2_] (**C**), rapid, room temperature P−H activation ensued to afford the phosphido hydrido complexes *trans*‐[Ni(I*i*Pr_2_)_2_(PPh_2_)H] (**3**) and *trans*‐[Ni(IEt_2_Me_2_)_2_(PPh_2_)H] (**4**), respectively (Scheme 3). The *trans*‐R_2_P−M−H geometry is rare for any transition metal,^[25]^ and contrasts with the *cis*‐R_2_P−Ni−H arrangement in [Ni(d*t*bpe){P(Dmp)H}H)] resulting from the presence of the chelating phosphine ligand.^[23]^

**Figure 2:**
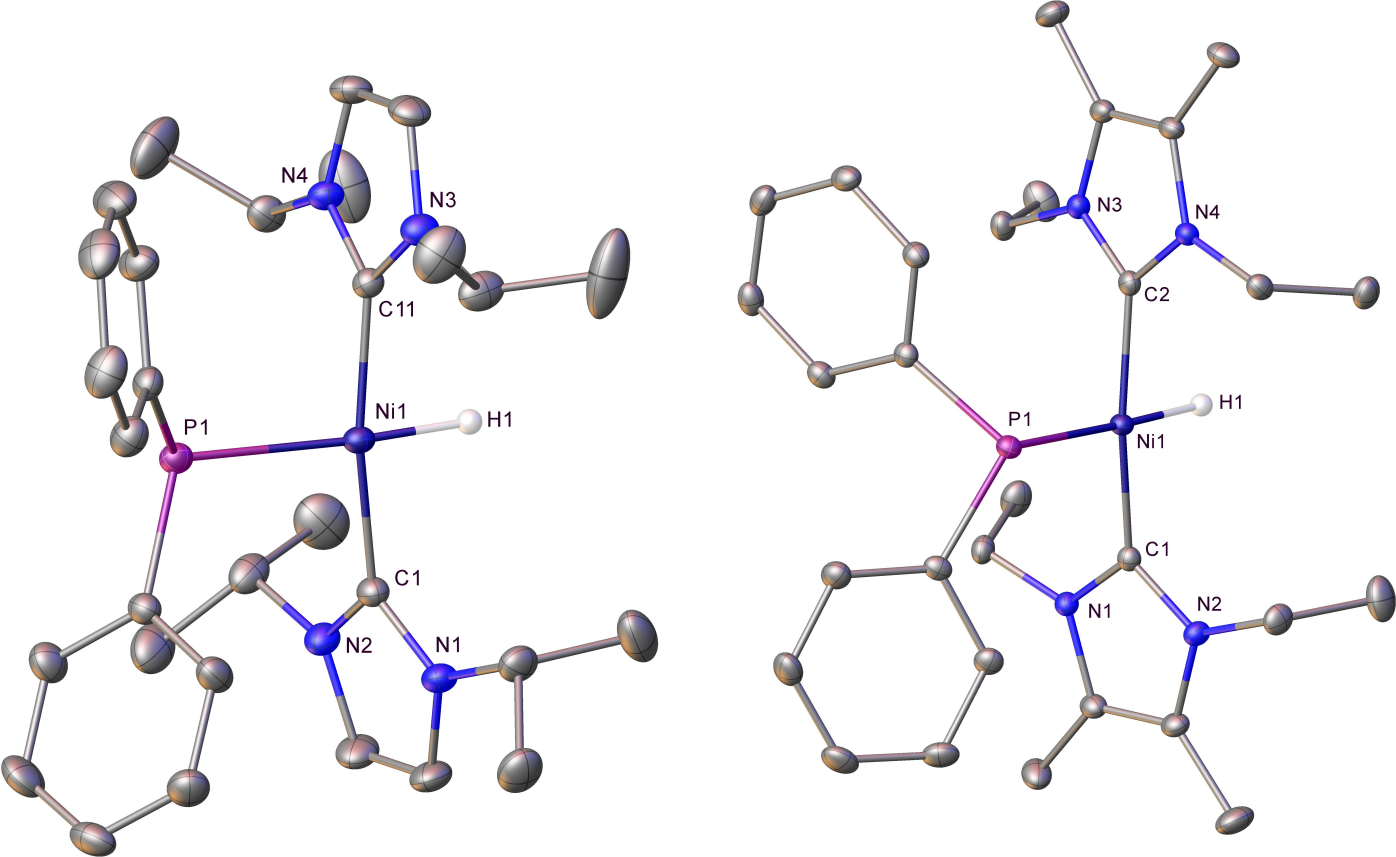
Molecular structures of left: *trans*‐[Ni(I*i*Pr_2_)_2_(PPh_2_)H] (**3**) and right: *trans*‐[Ni(IEt_2_Me_2_)_2_(PPh_2_)H] (**4**). Ellipsoids are shown at 30 % probability with all hydrogen atoms, except Ni−H, removed for clarity.

The *trans* Ph_2_P−Ni−H arrangement in both **3** and **4** was established by X‐ray crystallography (Figure 2) and multinuclear NMR spectroscopy. The change of ligand *trans* to phosphido group from Ph (**2**) to H (**4**) resulted in a slight shortening of the Ni−PPh_2_ distance, despite the higher *trans* influence of the latter (**2**: 2.2851(8) Å; **4**: 2.2783(4) Å). This may reflect the narrowing of the C_NHC_−Ni−C_NHC_ bond angle (**2**: 175.26(12)°; **4**: 169.16(5)°) that permits a closer approach of the phosphido ligand on steric grounds. Ni−H resonances for **3** and **4** were found at *δ* −11.3 ppm and *δ* −11.7 ppm, respectively, both with a doublet ^2^ *J*(H,P) splitting of 73 Hz. Identification as a *trans*‐coupling by comparing it with the *cis*‐splitting in [Ni(d*t*bpe){P(Dmp)H}H]^[23a]^ was prevented by the absence of any resolvable couplings on the Ni−H signal of the latter (even at low temperature). Although the *cis* phosphido‐Ni−H splitting was likewise unresolved in the related complex [Ni(d*t*bpe){PMes*H}H] (Mes*=2,4,6‐*t*Bu_3_C_6_H_2_),^[23b]^ this did show a coupling of comparable large magnitude (104 Hz) for *J*(*trans* phosphine‐Ni−H).^[26]^ The best comparison involves the iridium bis‐PH_2_ hydrido complex, [Ir(PEt_3_)_2_(PH_2_)_2_(CO)H],^[25c]^ which shows large *trans* (42 Hz) and much smaller *cis* (7 Hz) values of ^2^ *J*(PH_2_‐Ir−H). Final confirmation of geometry in **3** and **4** was provided by the appearance of a small (11 Hz) *cis* NHC−Ni−PPh_2_^2^ *J*(C,P) doublet splitting on each of the high frequency ^13^C carbene resonances of **3** and **4**.

### Formation of *trans*‐[Ni(NHC)_2_(PAr_2_)_2_] complexes

**Scheme 4:**
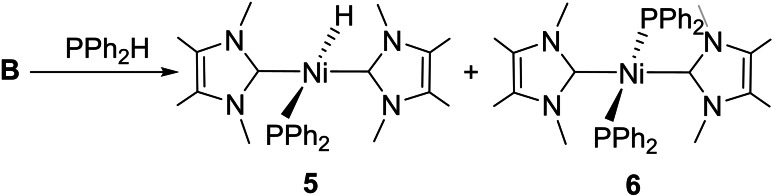
Phosphido hydrido and bis‐phosphido complexes from **B** and PPh_2_H.

In contrast to the clean formation of phosphido hydrido complexes **3** and **4**,^[27]^ [Ni(IMe_4_)_2_] (**B**) reacted with PPh_2_H to give a mixture of both the phosphido hydrido complex *trans*‐[Ni(IMe_4_)_2_(PPh_2_)H] (**5**) and the bis‐phosphido species, *trans*‐[Ni(IMe_4_)_2_(PPh_2_)_2_] (**6**, Scheme 4). Conversion through to just **6** took place over about 24 h at room temperature.^[28]^

**Scheme 5:**
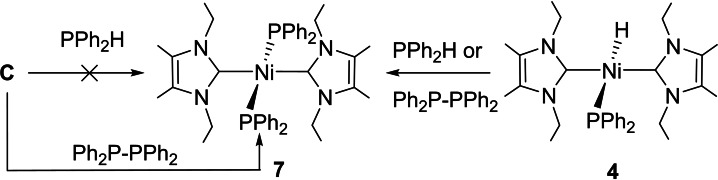
Routes to [Ni(IEt_2_Me_2_)_2_(PPh_2_)_2_] **7**.

**Scheme 6:**
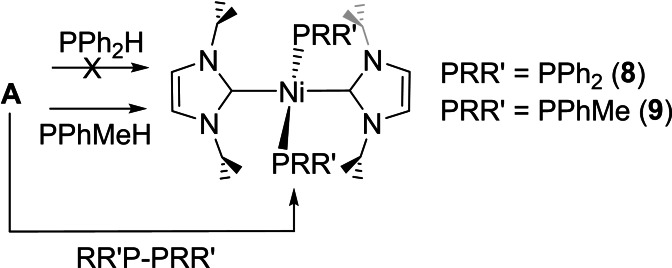
Formation of bis‐phosphido complexes from **A**.

Despite this difference in initial products from **A** and **B** (Schemes 3 and 4), we found that addition of PPh_2_H to [Ni(IEt_2_Me_2_)_2_(PPh_2_)H] (**4**) did bring about conversion through to the bis‐phosphido complex, *trans*‐[Ni(IEt_2_Me_2_)_2_(PPh_2_)_2_] (**7**, Scheme 5),^[27]^ although the process was slow, requiring over *ca*. 1 month at room temperature to reach completion.^[29]^ Steric factors likely contribute significantly to the difficulty of this transformation; indeed, exposure of the bulkier I*i*Pr_2_ precursor **A** to even a large excess of PPh_2_H failed to generate any of the bis‐PPh_2_ complex, *trans*‐[Ni(I*i*Pr_2_)_2_(PPh_2_)_2_] (**8**), although the less bulky methylphenylphosphido derivative, *trans*‐[Ni(I*i*Pr_2_)_2_(PPhMe)_2_] (**9**), could be made this way (Scheme 6).

Unexpectedly, **8** could be formed through P−P bond activation of Ph_2_P−PPh_2_ in the presence of **A** (Scheme 6), which also reacted with PhMeP−PMePh to give an alternative route to **9**. In fact, P−P activation proved to be a highly efficient route to bis‐phosphido complexes; formation of **7** was complete within just 30 min when Ph_2_P−PPh_2_ was added to **C** and within a few hours upon addition to **4**. Although P−P activation of P_4_ and other cyclic/oligomeric P_*x*_ species is well‐known,^[30]^ activation (or even simple coordination) of diphosphines at a transition metal (or p‐block) fragment is extremely rare.[^31^, ^32^, ^33^] Indeed, Ph_2_P−PPh_2_ is most commonly encountered as an unreactive elimination product in catalytic dehydrocoupling reactions of secondary phosphines.^[34]^

**Figure 3:**
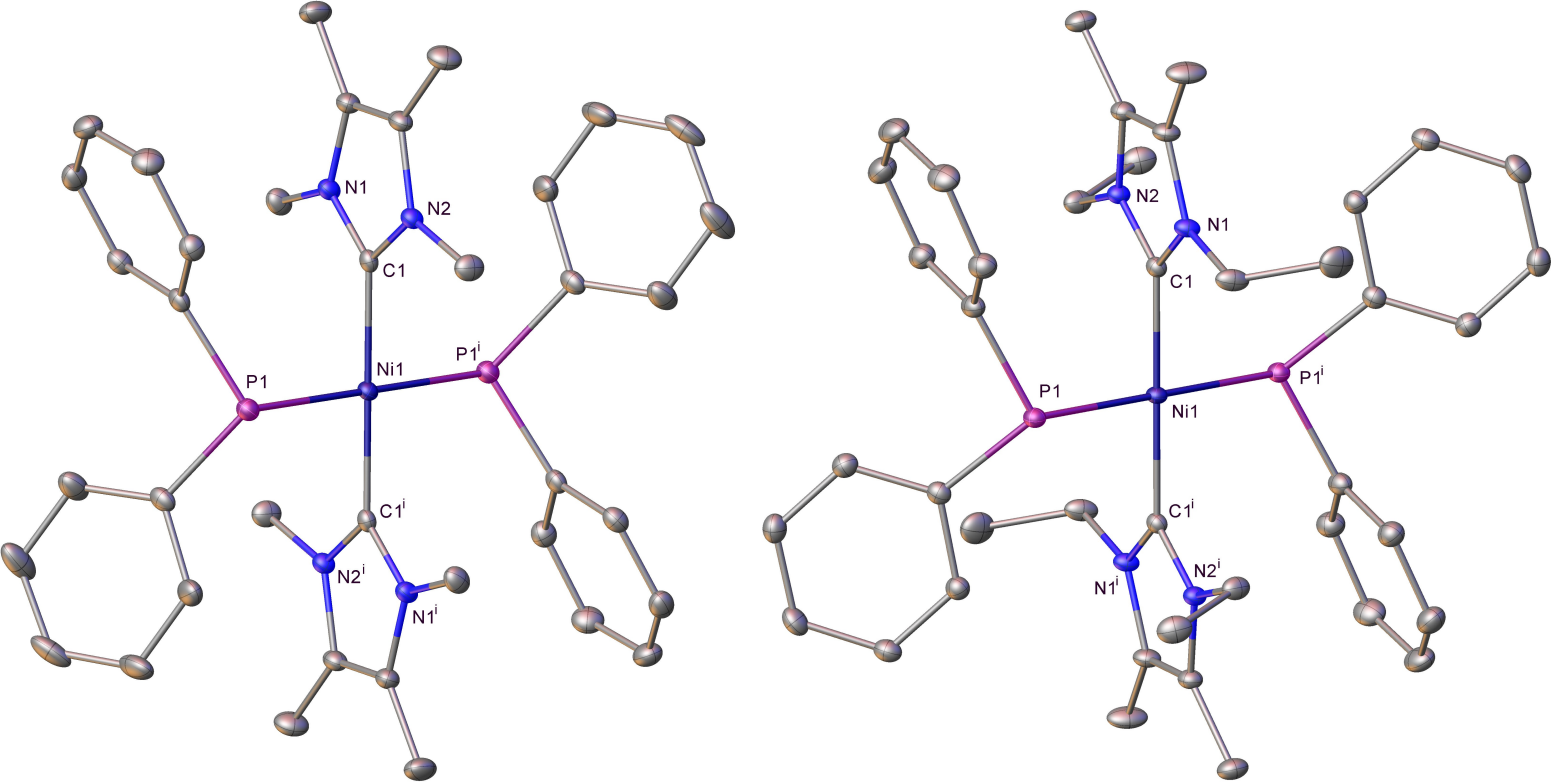
Molecular structures of *trans*‐[Ni(IMe_4_)_2_(PPh_2_)_2_] (**6**) and *trans*‐[Ni(IEt_2_Me_2_)(PPh_2_)_2_] (**7**). Ellipsoids are shown at 30 % probability and all hydrogen atoms are removed for clarity (**6**: ^i^ 1−*x*, −*y*, 1−*z* symmetry operation; **7**: ^i^ 1−*x*, 1−*y*, 1−*z* symmetry operation).

Figure 3 shows the X‐ray crystal structures of **6** and **7**, which we believe represent the first structurally characterized examples of mononuclear metal complexes with *trans* terminal phosphido ligands.[^35^, ^36^] The Ni atoms are coincident with crystallographic inversion centres in both structures, resulting in a *trans* disposition of the two sets of phenyl substituents about the P−Ni−P axis in **6** and the two sets of N−Et groups about the C−Ni−C axis in **7**. The Ni−P distance in **7** (2.2668(3) Å; 2.2628(5) Å in **6**) was shorter than in both [Ni(IEt_2_Me_2_)_2_(PPh_2_)Ph] (**2**) and [Ni(IEt_2_Me_2_)_2_(PPh_2_)H] (**4**), and there was a minor increase in the sum of the angles at the pyramidal phosphorus atoms (**2**, 325.4°; **4**, 324.3°; **7**, 329.6°).

The isolation of the NHC‐containing bis‐phosphido complexes **6** and **7** is in marked contrast to the stabilities of the bis(triethylphosphine) derivatives, *trans*‐[M(PEt_3_)_2_(P(SiMe_3_)_2_)_2_].^[37]^ The M=Pt derivative survives long enough to allow a *trans*‐R_2_P−Pt−PR_2_ arrangement to be confirmed spectroscopically, whereas the M=Ni analogue proved to be unstable above −70 °C. The difference in stability imparted by NHCs underlines the comparison made earlier between **1** and [Ni(PEt_3_)_2_(PPh_2_)Ph].^[20]^

Unequivocal spectroscopic evidence for the presence of *trans* R_2_P−Ni−PR_2_ arrangements in the four bis‐phosphido complexes **6**–**9** was provided by i) the small (^2^ *J*(C,P)=15–17 Hz) triplet splitting of the ^13^C carbenic carbon resonance and ii) the appearance in all the spectra of a virtually coupled signal at about *δ*=150 ppm for the magnetically inequivalent *ipso*‐C of the two *trans*‐phosphido ligands.

### Reactivity of [Ni(NHC)_2_] with primary aryl phosphines

**Scheme 7:**
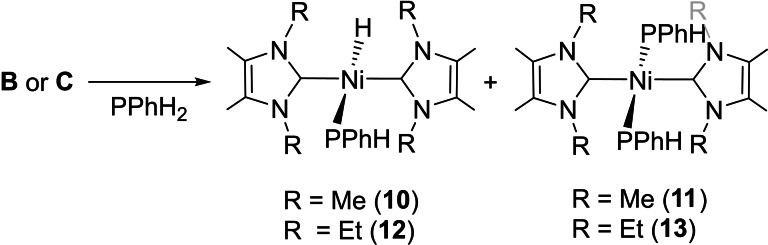
Phosphido hydrido and bis‐phosphido complexes from PPhH_2_.

**Scheme 8:**
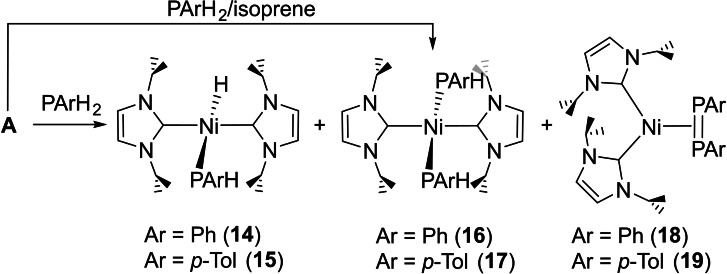
Reactivity of **A** towards primary aryl phosphines.

P−H bond activation of the primary aryl phosphine PPhH_2_ by **B** and **C** (Scheme 7) was also facile, although both reactions gave a mixture of the primary phosphido hydrido complexes *trans*‐[Ni(NHC)_2_(PPhH)H] (NHC=IMe_4_ **10**, IEt_2_Me_2_ **12**) and bis‐phosphido products, *trans*‐[Ni(NHC)_2_(PPhH)_2_] (NHC=IMe_4_ **11**, IEt_2_Me_2_ **13**). As shown in Scheme 8, **A** displayed even more complex reactivity with 1–4 equiv. PPhH_2_ (as well as P(*para*‐Tol)H_2_) giving a mixture of three products, even when the reactions were conducted at −30 °C.^[38]^ The formation of *trans*‐[Ni(I*i*Pr_2_)_2_(PArH)H] (Ar=Ph **14**, *para*‐Tol **15**) was signified by the appearance of low frequency signals (see below) in both the ^1^H and ^31^P NMR spectra, although these complexes defied all efforts at isolation. Repeating the reactions in the presence of an alkene as a hydrogen acceptor allowed isolation and characterisation of the second component of the reactions, namely the bis‐phosphido complexes, *trans*‐[Ni(I*i*Pr_2_)_2_(PArH)_2_] (Ar=Ph **16**, *para*‐Tol **17**). The diphosphene complexes, [Ni(I*i*Pr_2_)_2_(η^2^‐ArP=PAr)] (Ar=Ph **18**, *para*‐Tol **19**) were identified as being the third component upon subsequent preparation using more forcing conditions (see below).

**Scheme 9:**
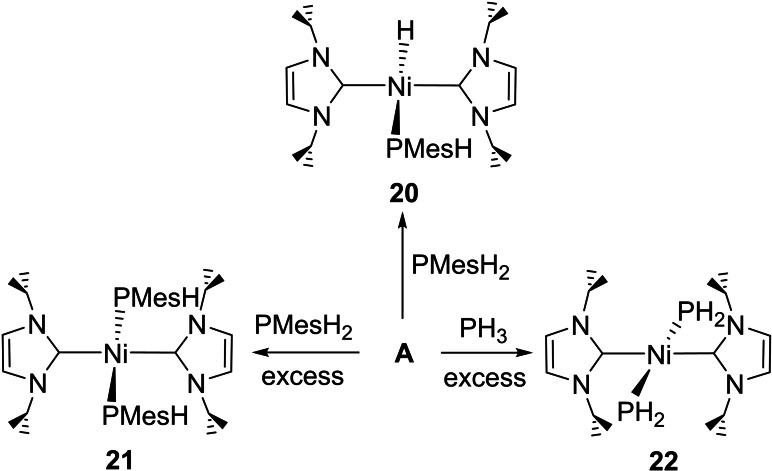
Reactivity of **A** with primary phosphines.

Increasing the phosphine bulk proved one way to direct the reactivity of **A**. Thus treatment with mesitylphosphine (PMesH_2_) gave around 95 % formation (based on NMR spectroscopy) of only the phosphido hydrido complex, *trans*‐[Ni(I*i*Pr_2_)_2_(PMesH)H] (**20**, Scheme 9). As for **10** and **12**, this was readily characterised from the ^1^H NMR spectrum through the presence of a low frequency Ni−H resonance (ca. *δ*=−11 ppm) with a ^2^ *J*(H,P) doublet splitting (61 Hz) again indicative of the hydride being *trans* to the phosphido ligand. An additional smaller coupling of 2 Hz to the PMes*H* proton was observed. The P−H resonance itself appeared at *δ*=2.9 ppm with a doublet ^1^ *J*(H,P) coupling of 200 Hz; as noted by others, the magnitude of this splitting is similar to that in the free primary phosphine.^[39]^ In the proton coupled ^31^P NMR spectrum, the phosphido ligand appeared as a very low frequency doublet of doublets (*δ*=−91 ppm), while the ^13^C{^1^H} NMR spectrum displayed the high frequency (*δ*=191 ppm) carbenic carbon resonance as the anticipated doublet with a *cis*‐[NHC−Ni−PR_2_] ^2^ *J*(C,P) coupling of 9 Hz.

Given the similarity of the NMR spectra of **20** to those of **10**, **12**, **14** and **15**, it is reasonable to assume that these four compounds also displayed *trans*‐[H−Ni−PArH] geometries; they therefore provide a direct comparison of spectroscopic data for isostructural primary and secondary phosphido hydrido complexes. Thus, the Ni−H resonances of the former resonated at slightly higher frequency and showed smaller ^2^ *J*(H,P) coupling constants (**10**: *δ*=−10.04 ppm, ^2^ *J*(H,P)=58.0 Hz c.f. **5**: *δ*=−10.97 ppm, ^2^ *J*(H,P)=68.0 Hz; **14**: *δ*=−10.41 ppm, ^2^ *J*(H,P)=62.1 Hz *c.f*. **3**: *δ*=−11.31 ppm, ^2^ *J*(H,P)=70.0 Hz), while the ^31^P NMR signals appeared at significantly lower frequencies (**10**: *δ*=−43.8 ppm; **12**: *δ*=−38.1 ppm; **14**: *δ*=−41.2 ppm cf. **5**: *δ*=19.3 ppm; **4**: *δ*=18.1 ppm; **3**: *δ*=25.1 ppm; cf. Table 1).^[40]^

**Table 1:** Comparison of bond distances [Å] and angles [°] and selected NMR spectroscopic data (*δ* (ppm), *J* [Hz]) for the bis‐phosphido complexes **6**, **7**, **11**, **16**, **17**, **21** and **22**.

	**6**	**7**	**11**	**16**	**17**	**21**	**22**
r(Ni−P)	2.2628(5)	2.2668(3)	2.2349(3)	2.2399(13)	2.2396(13)	2.2671(8)	2.2241(9)
				2.2414(13)		2.2550(8)	
r(Ni−C_NHC_)	1.8893(18)	1.8905(12)	1.8917(12)	1.896(4)	1.8852(19)	1.885(3)	1.890(3)
				1.888(4)		1.899(3)	
∠(P−Ni−P)	179.999(16)	180.00	180.00	175.42(6)	180.000(17)	172.12(3)	180.00
Σ(angles at P)	327.7	329.6	307.1	311.7/313.2	314.2	320.2/321.3	306.8
*δ* ^1^H_PH_	–	–	3.33	3.11	3.11	2.94	0.90
^31^P	31.2	47.1	−40.4	−29.5	−31.0	−71.2	−156.3
^1^ *J*(H,P)	–	–	–	198	198	200	171
*δ* ^13^C_NHC_	182.7	190.3	188.1	186.3	190.0	185.9	190.0
^2^ *J*(C_NHC_,P)	16	13	13	14	13	13	11

### Characterisation of *trans*‐[Ni(NHC)_2_(PArH)_2_] species

The bis‐phosphido complexes **11** and **13** (Scheme 7) formed as the ultimate products of the reactions of **B** and **C** with PPhH_2_ over several days at room temperature. Similarly, despite the bulk of PMesH_2_, **20** also slowly converted to *trans*‐[Ni(I*i*Pr_2_)_2_(PMesH)_2_] (**21**, Scheme 9) upon addition of further PMesH_2_, albeit in only low yield. At the opposite end of the steric spectrum to **21** was *trans*‐[Ni(I*i*Pr_2_)_2_(PH_2_)_2_] (**22**), which formed as the major product of the reaction of **A** with excess PH_3_ (Scheme 9).^[41]^

**Figure 4:**
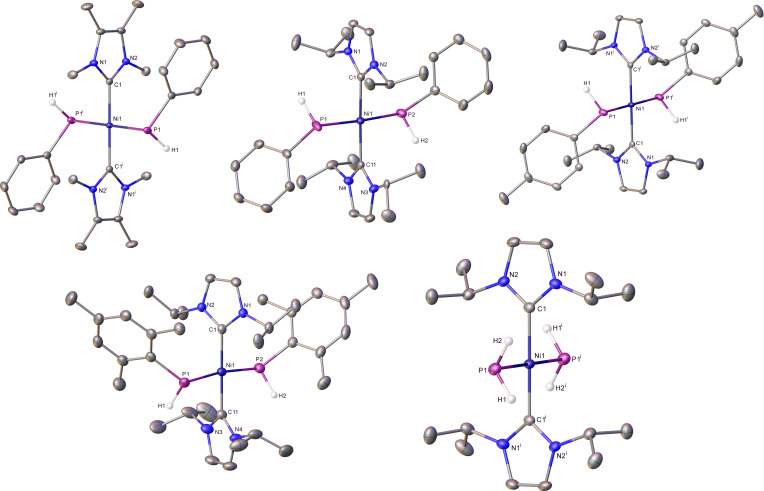
Molecular structures (from top left to bottom right) of *trans*‐[Ni(IMe_4_)_2_(PPhH)_2_] (**11**; ^i^ 1− *x*, 1−*y*, 1−*z* symmetry operation), *trans*‐[Ni(I*i*Pr_2_)_2_(PPhH)_2_] (**16**), *trans*‐[Ni(I*i*Pr_2_)_2_(P{*para‐*Tol)H}_2_] (**17**; ^i^ 1−*x*, 1−*y*, −*z* symmetry operation), *trans*‐[Ni(I*i*Pr_2_)_2_(PMesH)_2_] (**21**) and *trans*‐[Ni(I*i*Pr_2_)_2_(PH_2_)_2_] (**22**; ^i^ −*x*, 2−*y*, 1−*z* symmetry operation). Ellipsoids are represented at 30 % probability, and hydrogens (with the exception of those bonded to phosphorus) have been omitted for clarity. Also omitted is the minor disordered component in **16** and lattice solvent in **21**. Only one of the two molecules in the asymmetric unit of **22** is shown.

Complexes **11**, **16**, **17**, **21** and **22** were structurally characterized as shown in Figure 4. They represent not only rare cases of structurally characterized metal primary phosphido complexes[^21c^, ^40^, ^42^] but are the first examples of *trans*‐RHP−M‐PRH complexes. Table 1 summarises selected solid‐state and spectroscopic metrics for these compounds, together with the two bis‐PPh_2_ complexes **6** and **7** for comparison. In general, the Ni−PArH distances are slightly shorter than those in the Ni−PAr_2_ species (with the exception of the sterically crowded bis‐P(Mes)H complex **21**). Although the *trans* PR_2_−Ni−PR_2_ geometry may limit comparisons to other systems, Glueck has reported that the Pd−PR_2_ distance in [Pt(dppe)(PMes_2_)Me]>[Pt(dppe)(P(Mes)H)Me] and that the *trans* influence of PPh_2_>PHPh for the [Pt(dppe)(PR_2_)Me] complexes.^[39]^ The bis‐PH_2_ complex **22** is notable in displaying significant upfield shifts of both the ^1^H and ^31^P signals of the PH_2_ ligands, as well as a much reduced ^1^ *J*(H,P) coupling constant.^[43]^

### Isolation and characterization of [Ni(I*i*Pr_2_)_2_(η^2^‐ArP=PAr)]

The diphosphene complexes [Ni(I*i*Pr_2_)_2_(η^2^‐ArP=PAr)] (Ar=Ph **18**, *para*‐Tol **19**) were isolated in about 50 % yield by heating **A** with excess PPhH_2_ or P(*para‐*Tol)H_2_ at 140 °C. While diphosphene complexes have been synthesised previously using a range of approaches (ligand exchange of stable diphosphenes with labile M−L complexes, salt metathesis and degradation of oligophosphines),[^30e^, ^44^] their preparation by the formal dehydrocoupling of a primary phosphine in the coordination sphere of a transition metal as observed here is unusual.^[45]^

**Figure 5:**
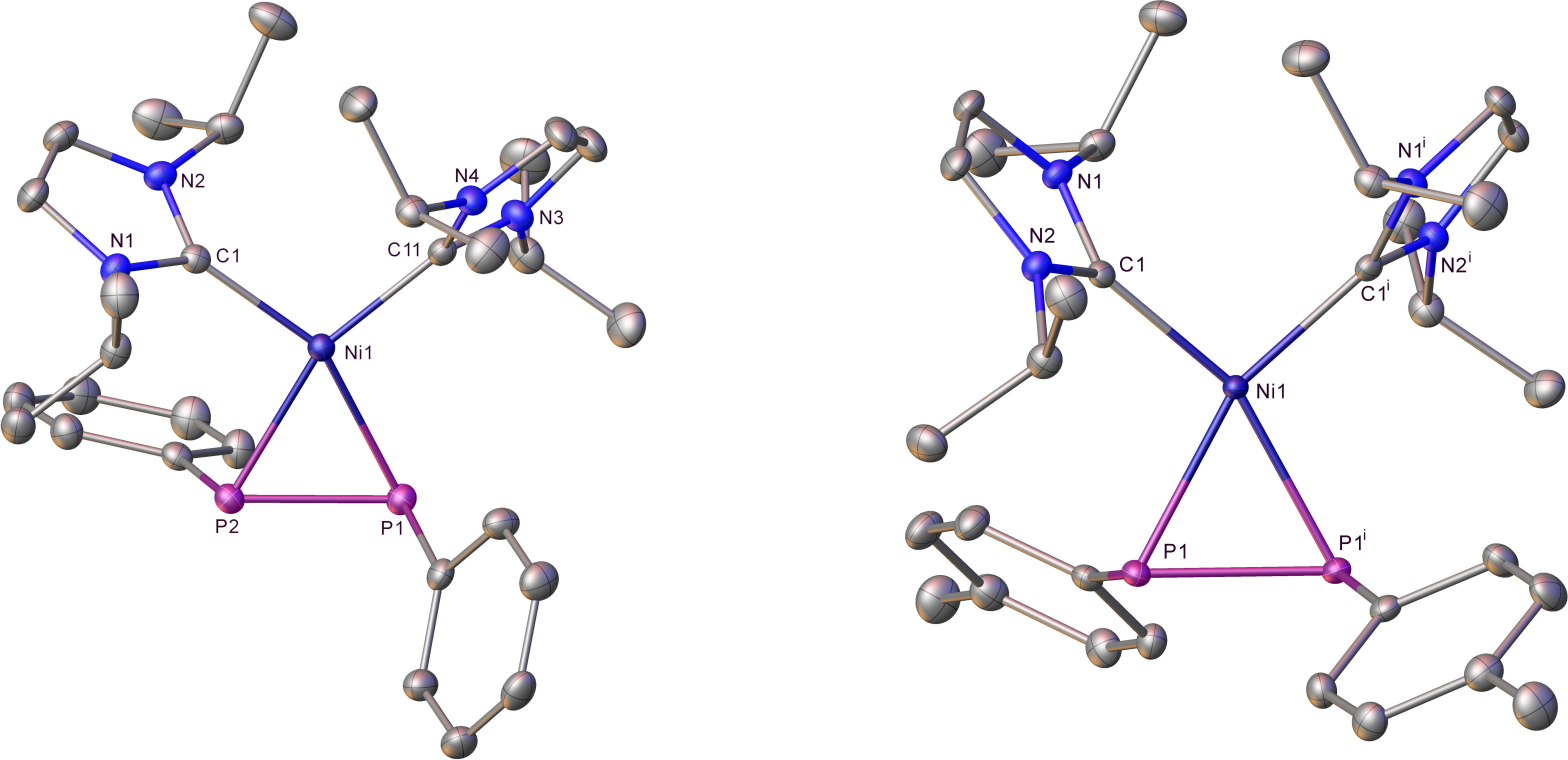
Molecular structures of [Ni(I*i*Pr_2_)_2_(η^2^‐PhP=PPh)] (**18**) and [Ni(I*i*Pr_2_)_2_{η^2^‐(*para*‐Tol)P=P(*para*‐Tol)}] (**19**). Ellipsoids are represented at 30 % probability, and hydrogens (along with lattice solvent in **19**) have been omitted for clarity. For **19**: ^i^ 1−*x*, *y*, 1/2 −*z* symmetry operation.

X‐ray crystallography (Figure 5) showed, in both cases, a *trans* disposition of the two aryl substituents in the diphosphene ligand. The P−P bond lengths (**18**: 2.1379(7) Å; **19**: 2.1342(7) Å) were in good agreement with those in [Ni(PEt_3_)_2_(*trans*‐MesP=PMes)] (2.1355(9) Å), [Ni(PMe_2_Ph)_2_(*trans*‐MesP=PMes)] (2.137(1) Å), and [Pd(dppe)(*trans*‐PhP=PPh)] (2.121(4) Å; dppe=1,2‐bis(diphenylphosphino)ethane), respectively.[^44^, ^46^] The angles C1−Ni−C11 (**18**, 104.62(6)°; **19**, 98.85(8)°) were larger than 90°, as expected for a pseudo three‐coordinated complex with small P−Ni−P angles (**18**, 56.76(2)°; **19**, 56.89(2)°).

The ^1^H NMR resonances of the I*i*Pr_2_ ligands in both **18** and **19** were broadened at room temperature, consistent with hindered rotation of the ArP=PAr ligand around the Ni‐(PP)_centroid_ vector and/or of the NHC ligands around the Ni−C bonds. Cooling to −50 °C resolved the spectra into four *i*Pr methyl and two *i*Pr methine resonances, as typically found for a *C*_2v_ type structure. At 90 °C, two *i*Pr methyl resonances and a single *i*Pr methine signal were observed, which were still broadened. Both complexes exhibited singlets in their ^31^P{^1^H} NMR spectra (**18**, *δ*=−41 ppm; **19**, *δ*=−39 ppm) to lower frequency of the Pd and Pt derivatives [M(dppe)(*trans*‐PhP=PPh)] (M=Pd, *δ*=−34 ppm; M=Pt, *δ*=−24 ppm).^[44]^

### Computational Studies

#### i) P−H Activation of PPh_2_H at [Ni(NHC)_2_]

The mechanisms of these processes were investigated using DFT calculations for the reactions of both [Ni(I*i*Pr_2_)_2_], **A**, and [Ni(IMe_4_)_2_], **B**. Geometry optimisation and frequency calculations were performed with the BP86 functional using SDD pseudopotentials and basis sets on Ni and P (with d‐orbital polarization on the latter) and 6–31G** basis sets on all other atoms (collectively termed BS1). Electronic energies were then recomputed with B97D including corrections for benzene solvent and a def2‐TZVP basis set (BS2). These were then combined with the BP86 thermochemical corrections to supply the relative free energies reported in the text. The choice of B97D was motivated by the computed barriers for the reactions of **A** and **B** with PPh_2_H, for which this functional gave values qualitatively consistent with the reactivities observed at room temperature (i. e., **B**: rapid formation of *trans*‐[Ni(IMe_4_)_2_(PPh_2_)H], **5**, followed by slower conversion to *trans*‐[Ni(IMe_4_)_2_(PPh_2_)_2_], **6**; **A**: rapid formation of *trans*‐[Ni(I*i*Pr_2_)_2_(PPh_2_)H], **3**, but no onward reaction to form *bis*‐phosphido complex **8**). The computed energetics were found to be very functional dependent, in a similar way that phosphine ligand addition energies are highly sensitive to functional choice and, in particular, the treatment of dispersion effects.[^47^, ^48^] This appears to be exacerbated here due to the sequential addition of two PPh_2_H ligands to two [Ni(NHC)_2_] systems of varying steric bulk. The data below therefore present our best proposed mechanisms in the face of these challenges.^[49]^ Full computational details, references and functional testing are provided in the Supporting Information.

**Figure 6:**
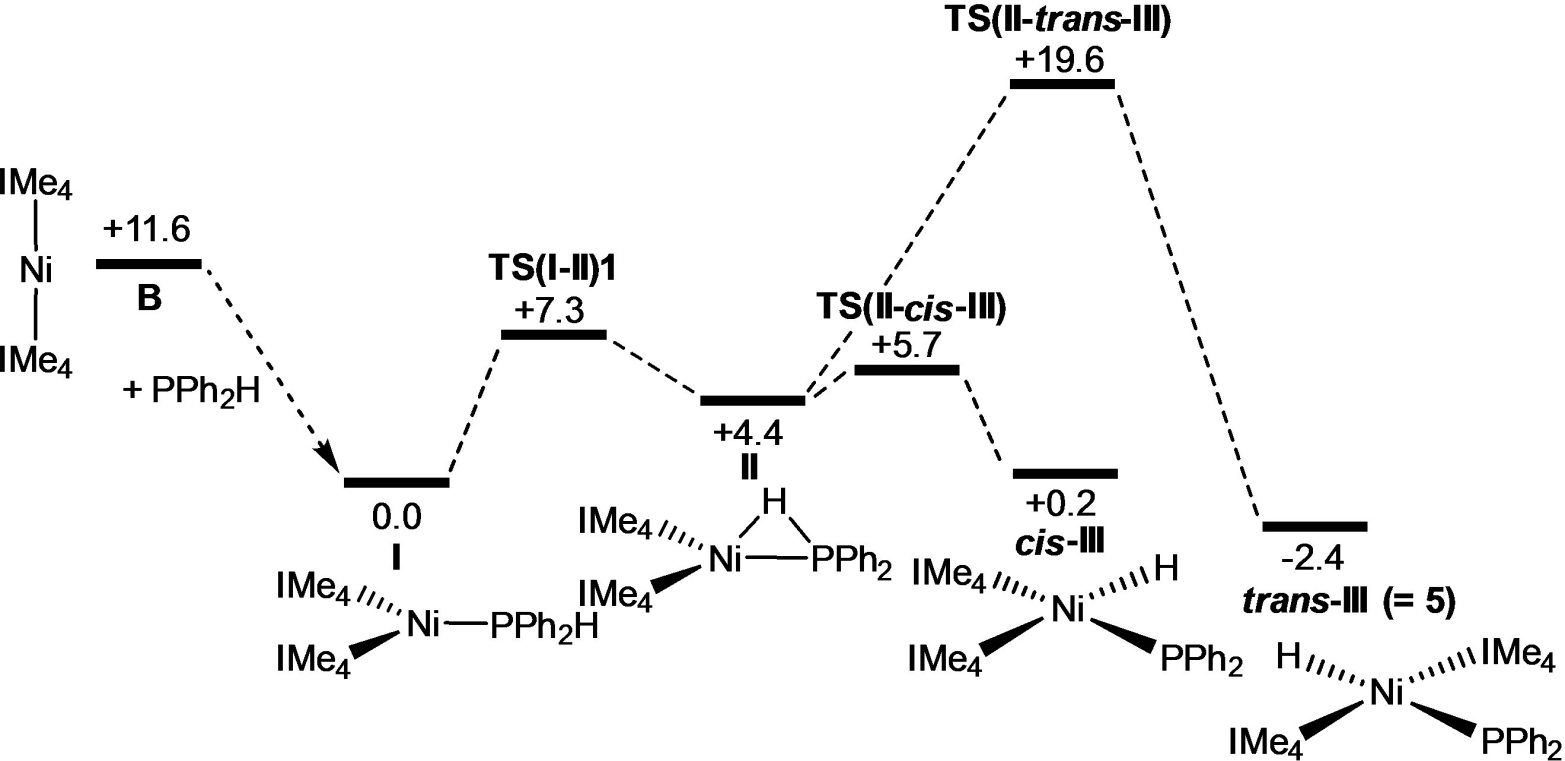
Computed free‐energy profile [kcal mol^−1^, B97D(benzene, BS2)//BP86(BS1)] for the reaction of [Ni(IMe_4_)_2_] (**B**) with PPh_2_H to form *trans*‐[Ni(IMe_4_)_2_(PPh_2_)H] **5**. The triplet form of [Ni(IMe_4_)_2_(PPh_2_)H], ^**3**^ **III**, is computed at +23.7 kcal mol^−1^.

**Figure 7:**
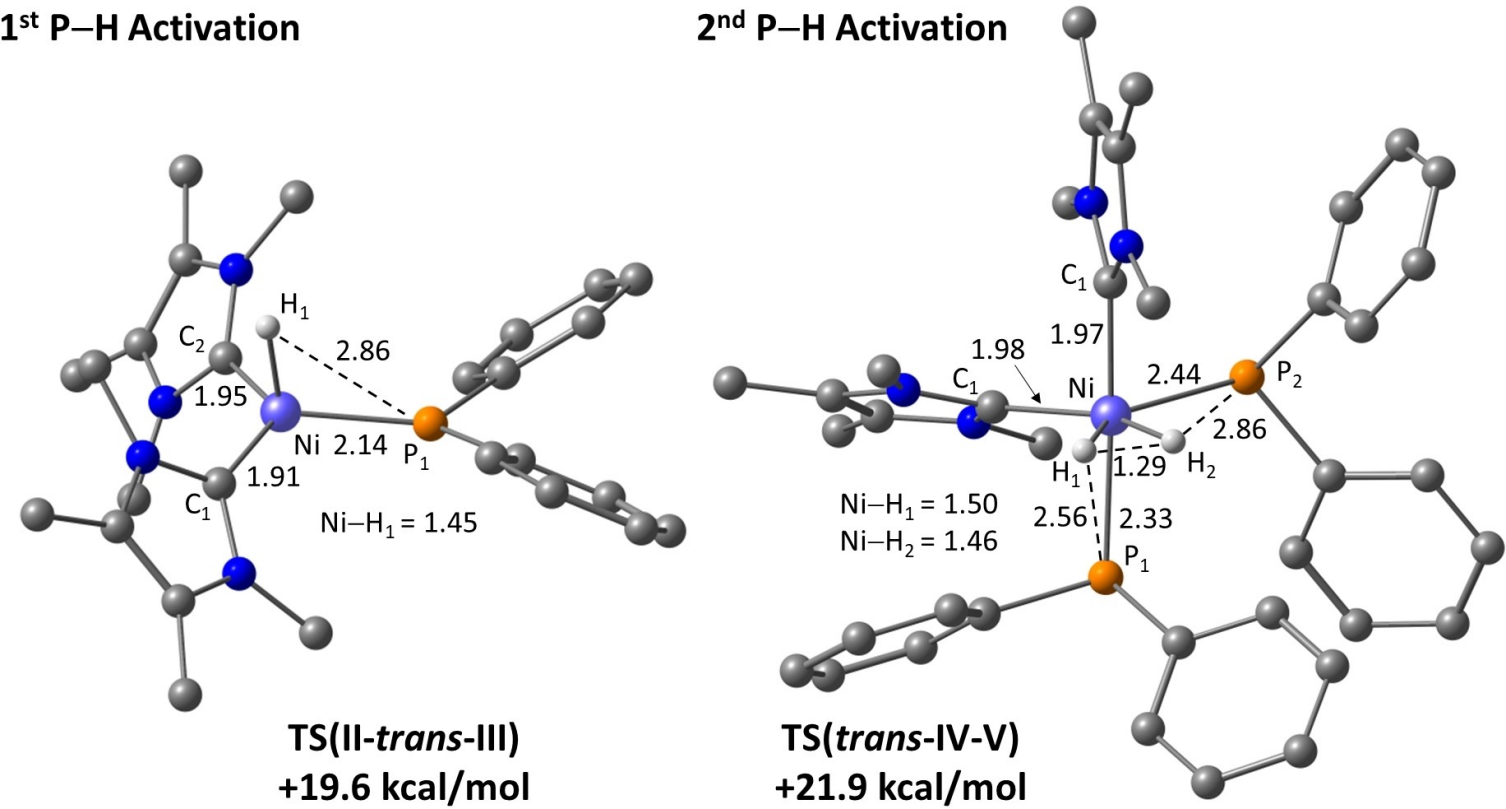
Computed geometries for the rate‐limiting transition states for the first and second P−H activation of PPh_2_H at **B**. Selected distances are shown in Å, with IMe_4_ and phenyl hydrogens omitted for clarity.

The free energy profile computed with B97D for the reaction of **B** with PPh_2_H to form **5** is shown in Figure 6. Addition of PPh_2_H to **B** is exergonic by 11.6 kcal mol^−1^ and forms [Ni(IMe_4_)_2_(PPh_2_H)], **I**, the energy of which is set to 0.0 kcal mol^−1^.^[50]^ P−H activation then proceeds via an initial rearrangement to intermediate **II**, in which the H substituent bridges the Ni−P bond (Ni−H=1.62 Å, P−H=1.59 Å, Ni−P=2.18 Å). The P−H bond is therefore significantly weakened and readily undergoes cleavage to form the *cis* isomer of **5**, denoted *cis*‐**III** in the computational study. Formation of *cis*‐**III** is slightly endergonic, and hence reversible, allowing the *trans* isomer to be accessed via **TS(II**‐*trans*‐**III)** at +19.6 kcal mol^−1^ (see Figure 7 for the computed geometries of rate‐limiting transition states). The much higher barrier associated with **TS(II**‐*trans*‐**III)** arises from the coupling of P−H bond cleavage with a *cis‐trans* isomerisation process. The triplet form of [Ni(IMe_4_)_2_(PPh_2_)H], ^**3**^ **III**, has a computed energy of +23.7 kcal mol^−1^ and so is not implicated in this isomerisation.^[51]^ IMe_4_ dissociation from *cis*‐**III** was also ruled out as this incurs a free energy penalty of 25.7 kcal mol^−1^. Overall, *trans*‐**III** is formed with an overall barrier of 19.6 kcal mol^−1^ in a process that is exergonic by 2.4 kcal mol^−1^ relative to **I**.

**Figure 8:**
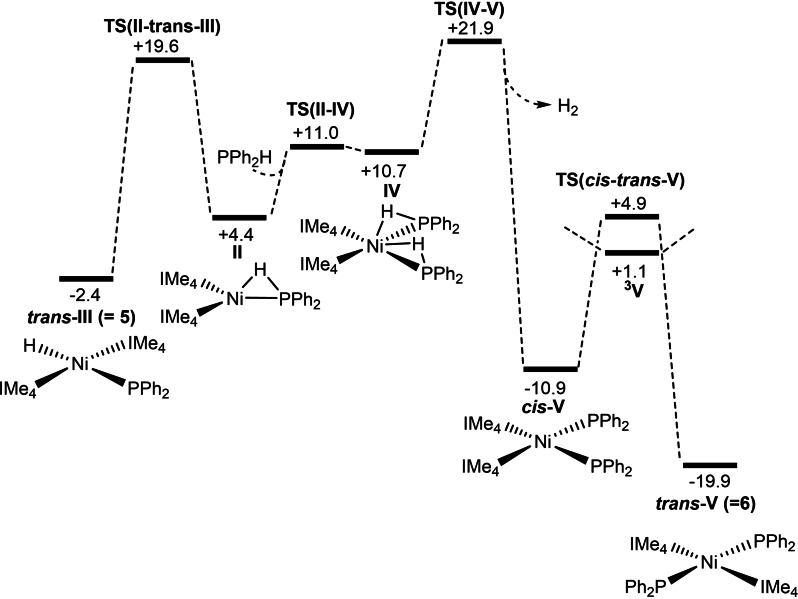
Computed free‐energy profile [kcal mol^−1^, B97D(benzene, BS2)//BP86(BS1)] for the reaction of **5** with PPh_2_H to form *trans*‐[Ni(IMe_4_)_2_(PPh_2_)_2_] **6**.

The lowest energy pathway for the onward reaction of **5** with a second molecule of PPh_2_H to form **6** is shown in Figure 8.^[52]^ This involves an initial P−H reductive coupling to reform **II** which can then add a second PPh_2_H molecule in the unconventional H‐bridged binding mode to give **IV** (+10.7 kcal mol^−1^). Both P−H bonds in **IV** are significantly elongated (ca. 1.63 Å) and a P−H activation transition state, **TS(IV**–**V)**, was located at +21.9 kcal mol^−1^ in which both P−H bonds are cleaved (Figure 8). This occurs with concomitant H_2_ elimination to give *cis*‐**V** directly.^[53]^ *cis*‐**V** can then isomerise to *trans*‐**V** (i. e., **6**) at −19.9 kcal mol^−1^ via **TS**(*cis*‐*trans*‐**V**) at +4.9 kcal mol^−1^ on the singlet surface, although in this case the triplet species ^**3**^ **V** lies 3.8 kcal mol^−1^ below **TS**(*cis*‐*trans*‐**V**) and so isomerisation may involve a spin crossover process.^[54]^ These isomerisation pathways do not affect the overall energy span of 24.3 kcal mol^−1^ for the second P−H bond activation, as this is associated with **TS(IV–V)** as the highest point on the profile. The formation of **6** from **5** is strongly exergonic (−17.5 kcal mol^−1^).

Overall, the first P−H activation at **B** to form **5** is more kinetically accessible (Δ*G* ^≠^=19.6 kcal mol^−1^) than the second to form **6** (Δ*G* ^≠^=+24.3 kcal mol^−1^) and this is qualitatively consistent with the rapid formation of **5** at room temperature and its subsequent slower conversion to **6**. Calculations for the reactions of **A** with PPh_2_H followed similar pathways, with a barrier of 20.2 kcal mol^−1^ for the first P−H activation to form **3** at −0.8 kcal mol^−1^. In this case the potential second P−H bond activation entails a higher overall barrier of 26.6 kcal mol^−1^, consistent with the formation of *trans*‐[Ni(I*i*Pr_2_)_2_(PPh_2_)_2_], **8**, being much less accessible at room temperature, at least by this route (see Figures S74 and S75 in the Supporting Information).^[55]^

**Figure 9:**
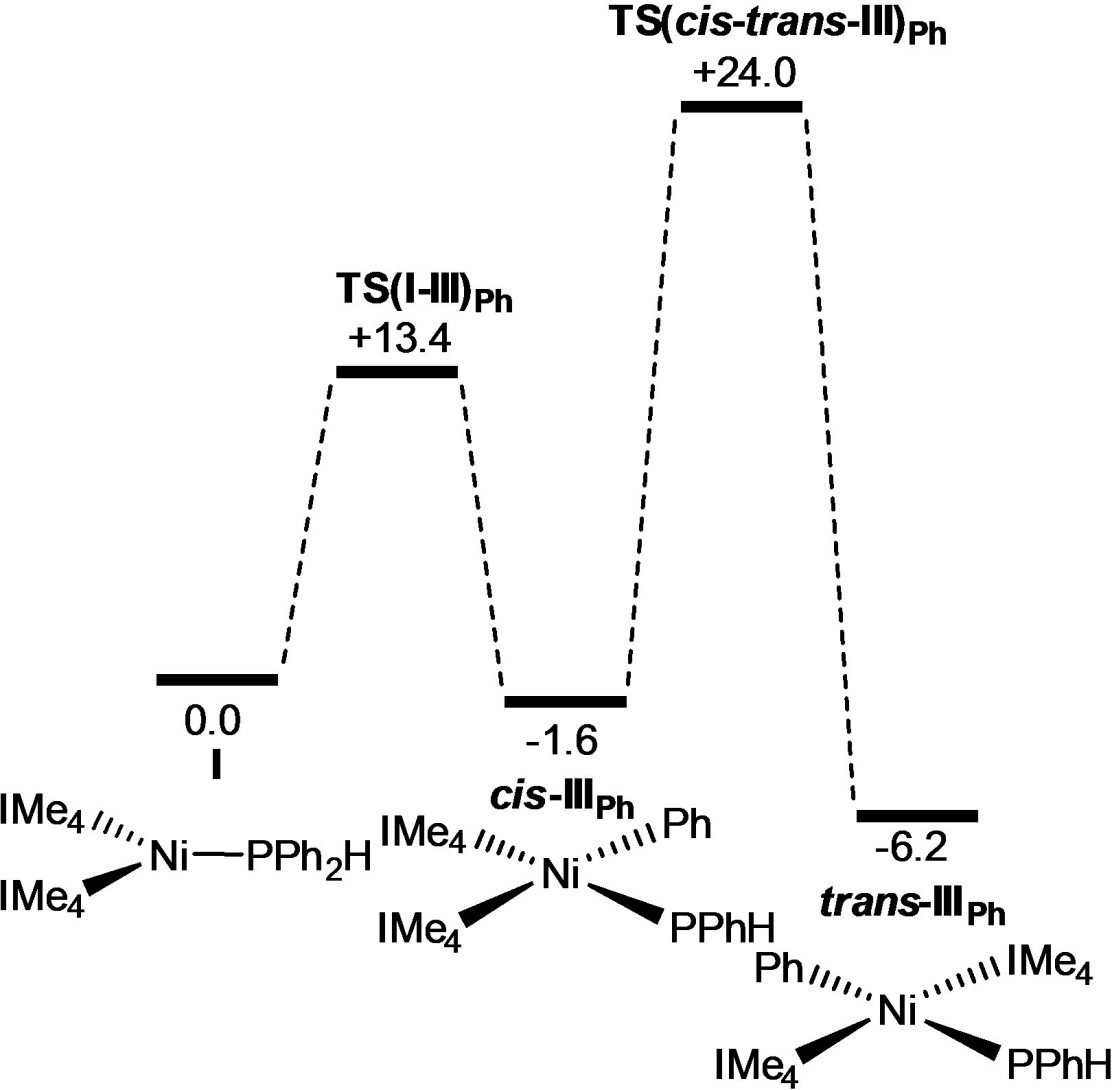
Computed free‐energy profile [kcal mol^−1^, B97D(benzene, BS2)//BP86(BS1)] for competing P−C bond activation in [Ni(IMe_4_)_2_(PPh_2_H)], **I**. The triplet form of [Ni(IMe_4_)_2_(PPh_2_)Ph], ^**3**^ **IV_Ph_**, is computed at +25.6 kcal mol^−1^.

As well as the observed P−H bond activations, the alternative P−Ph bond activation of PPh_2_H at [Ni(NHC)]_2_ is a potentially competing process (see Figure 9 for the IMe_4_ system). This proceeds with an initial computed barrier of 13.4 kcal mol^−1^ to give *cis*‐[Ni(IMe_4_)_2_(PPhH)Ph], *cis*‐**III_Ph_**, at −1.6 kcal mol^−1^ from which *cis–trans* isomerisation entails a barrier of 25.6 kcal mol^−1^ to give *trans*‐**III_Ph_** at −6.2 kcal mol^−1^. Therefore, in the absence of a second equivalent of PPh_2_H, P−H bond activation to form **6** is kinetically favoured over *trans*‐**III_Ph_** (Δ*G* ^≠^=+19.6 kcal mol^−1^, Δ*G*=−2.4 kcal mol^−1^) but as this could be reversible, this could in principle allow access to *trans*‐**III_Ph_** at −6.2 kcal mol^−1^ as the thermodynamically more stable product of P−C bond activation. This latter process, however, would have a significant barrier of 26.4 kcal mol^−1^ relative to **6**. Moreover, in the presence of excess PPh_2_H, a second P−H activation to form **6** is favoured both kinetically (Δ*G* ^≠^=+24.3 kcal mol^−1^) and thermodynamically (Δ*G*=−19.9 kcal mol^−1^). The pattern of a kinetically favoured P−H bond activation versus a thermodynamically favoured P−C bond activation has been reported for the reaction of [Pt(dppe)(*trans*‐stilbene)] with PMes_2_H.[^56^, ^57^] Attempts to realise this pattern experimentally through the reaction of [Ni(COD)_2_] with 2 equiv of IMe_4_ and only one equiv. of PPh_2_H were unsuccessful with a complex mixture of products being formed.

#### ii) P−P Activation of Ph_2_P−PPh_2_ at [Ni(NHC)_2_]

**Figure 10:**
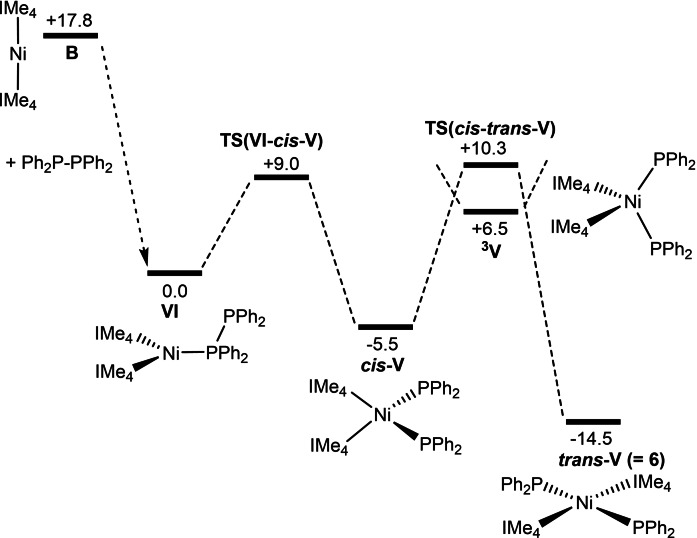
Computed free energy profiles [kcal mol^−1^, B97D(benzene, BS2)//BP86(BS1)] for the reaction of **B** with Ph_2_P‐PPh_2_ to form **6**.

**Figure 11:**
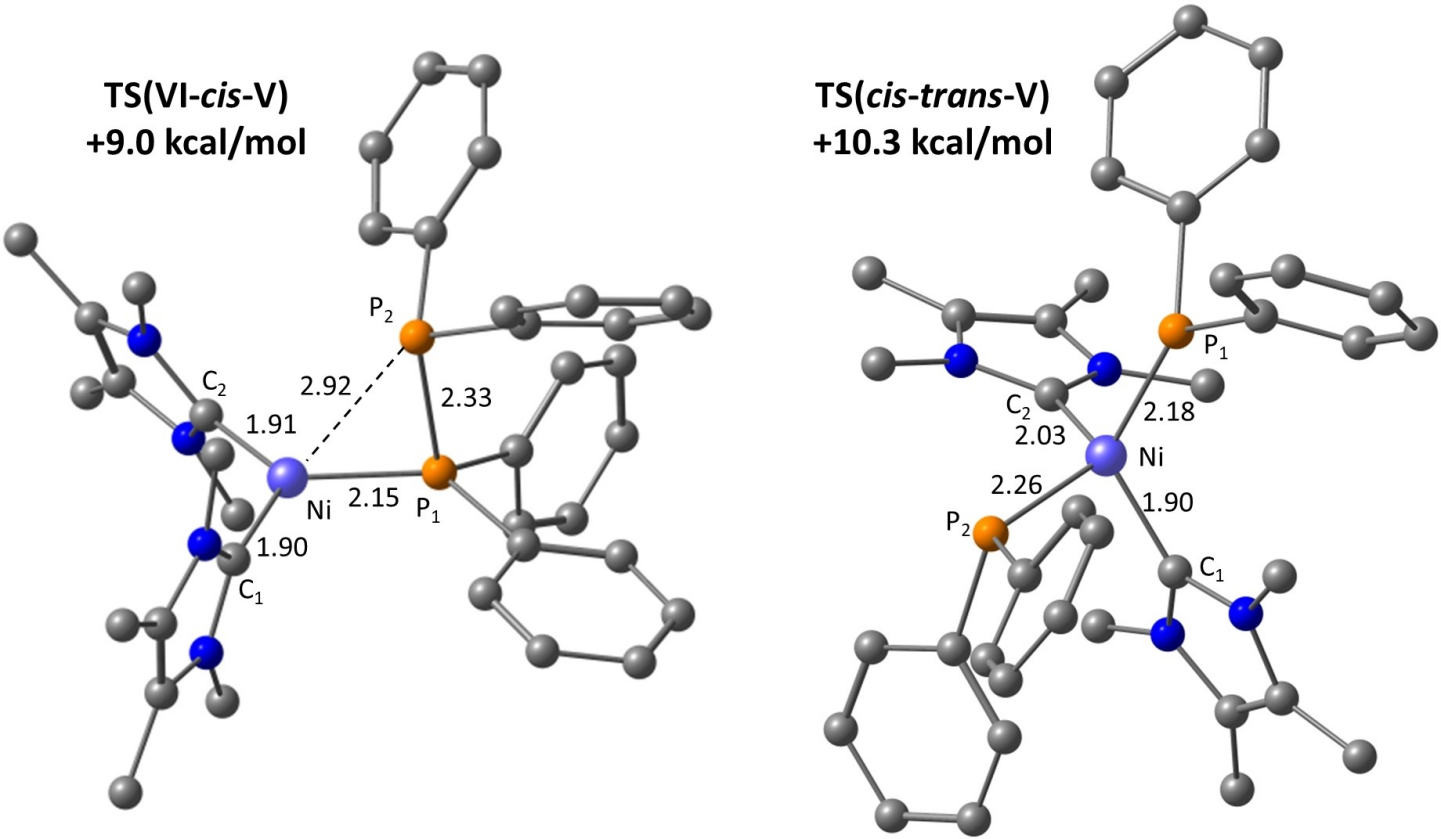
Computed geometries for key transition states in the P−P bond activation of Ph_2_PPPh_2_ at **B**. Selected distances are shown in Å, with IMe_4_ and phenyl hydrogens omitted for clarity.

The computed profile for the alternative route to *trans‐*[Ni(IMe_4_)_2_(PPh_2_)_2_], **6**, through reaction of Ph_2_P−PPh_2_ with **B** is shown in Figure 10, with the geometries of key transition states in Figure 11. Ph_2_P−PPh_2_ addition to **B** is exergonic by 17.8 kcal mol^−1^ (cf. 11.6 kcal mol^−1^ for PPh_2_H) and gives trigonal planar [Ni(IMe_4_)_2_(Ph_2_PPPh_2_)] (**VI**, Ni−P=2.35 Å), the energy of which is set to 0.0 kcal mol^−1^. P−P bond activation then proceeds via a facile transfer of one PPh_2_ group onto Ni via **TS(VI**‐*cis*‐**V)** at +9.0 kcal mol^−1^. This forms *cis*‐**V** at −5.5 kcal mol^−1^ that, as discussed above, can undergo *cis–trans* isomerisation on the singlet surface via **TS**(*cis*‐*trans*‐**VI**) at +10.3 kcal mol^−1^ or potentially via a lower energy spin crossover mechanism via ^**3**^ **V** at +6.5 kcal mol^−1^.^[58]^ *Cis–trans* isomerisation on the singlet surface represents the largest barrier along the profile for the formation of **6**, but at only 15.8 kcal mol^−1^, the implication is that the overall reaction to form **6** should be readily accessible, consistent with the experiment observation of this process at room temperature.

**Figure 12:**
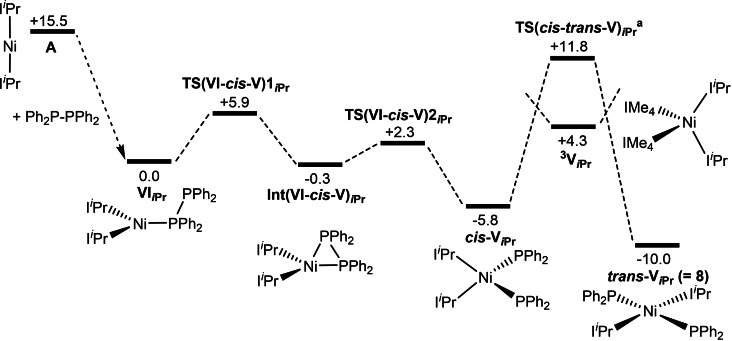
Computed free‐energy reaction profile [kcal mol^−1^, B97D(benzene, BS2)//BP86(BS1)] for the reaction of **A** with Ph_2_P−PPh_2_ to form *trans*‐[Ni(I*i*Pr)_2_(PPh_2_)_2_], **8**. [a] Only the rate‐limiting transition state for *cis–trans* isomerisation is indicated (see Figure S78 for more details).

P−P bond activation in the analogous reaction of Ph_2_P−PPh_2_ with **A** is shown in Figure 12. This now involves a two‐step process from the initial Ph_2_P−PPh_2_ adduct, **IV**_***i*** **Pr**_, via an intermediate **Int(VI**‐*cis*‐**V)**_***i*** **Pr**_ at −0.3 kcal mol^−1^ that features a bridging PPh_2_ ligand, followed by facile P−P bond cleavage to give *cis*‐**V**_***i*** **Pr**_ at −5.8 kcal mol^−1^. *Cis–trans* isomerisation on the singlet surface then proceeds via **TS**(*cis‐trans*‐**V**)_***i*** **Pr**_ at +11.8 kcal mol^−1^. The details of this process are complicated by the existence of numerous conformations along the pathway and only the highest‐lying transition state is shown in Figure 12 (see Figure S78 for more details). The triplet form, ^**3**^ **V**_***i*** **Pr**_, is again accessible at +4.3 kcal mol^−1^. As with the analogous reaction with **B**, all barriers are readily accessible, the largest energy span being only 17.6 kcal mol^−1^ (corresponding to isomerisation on the singlet surface) and so the formation of **8** should be readily accessible, as is the case experimentally.

## Conclusions

Facile P−H activation of secondary and primary phosphines by the 14‐electron fragments [Ni(I*i*Pr_2_)_2_] (**A**), [Ni(IMe_4_)_2_] (**B**) and [Ni(IEt_2_Me_2_)_2_] (**C**) provides access to a unprecedented series of stable terminal phosphido complexes with *trans* H−Ni−PR_2_ and *trans*‐R_2_P−Ni−PR_2_ structures. Within the family of [Ni(NHC)_2_] fragments that we have considered, clear evidence for the stereoelectronic influence of the N substituents is apparent. This is best evidenced in the initial reactions of **A**, **B** and **C** with PPh_2_H. With bulkier **A** and **C**, the initial room temperature reaction yields isolable phosphido hydrido complexes, *trans*‐[Ni(I*i*Pr_2_)_2_(PPh_2_)H] (**3**) and *trans*‐[Ni(IEt_2_Me_2_)_2_(PPh_2_)H] (**4**), respectively. With **B**, a mixture of the equivalent phosphido hydrido *trans*‐[Ni(IMe_4_)_2_(PPh_2_)H] (**5**) and bis‐phosphido *trans*‐[Ni(IMe_4_)_2_(PPh_2_)_2_] (**6**) complexes is first seen that then converts fully to **6** over 24 h. Thus **B**, that features the smallest of the carbenes, IMe_4_ can most easily accommodate two P−H bond activations to form a *bis*‐phosphido directly. Further reaction of **4** with PPh_2_H to form *trans*‐[Ni(IEt_2_Me_2_)_2_(PPh_2_)_2_] (**7**) does occur but is very slow, requiring weeks at room temperature. No further reaction between **3** and PPh_2_H is seen and so [Ni(I*i*Pr_2_)_2_(PPh_2_)_2_] (**8**) is inaccessible by this route. We were able to prepare **8**, as well as **7** and the mixed aryl/alkyl bis‐phosphido complex [Ni(I*i*Pr_2_)_2_(PPhMe_2_)_2_] (**9**) by the unexpected P−P cleavage of the diphosphines R_2_P−PR_2_, which like the P−H activation reactions above, takes place under very mild conditions.

These trends were captured by DFT calculations on the reactions of **A** and **B**, although the computed energetics of these processes were found to be very functional dependent. With PPh_2_H low barriers for the first P−H activation (**A**: 20.2 kcal mol^−1^; **B**: 19.6 kcal mol^−1^, B97D functional) contrasted with increased barriers for the second P−H activation (**A**: 26.6 kcal mol^−1^; **B**: 24.3 kcal mol^−1^). Each P−H bond activation proceeds from an unconventional phosphine adduct in which the H substituent bridges the Ni−P bond. The rate limiting transition states then correspond to P−H bond cleavage; for the first P−H activation, this is coupled to *cis*–*trans* isomerisation to form *trans*‐[Ni(NHC)_2_(PPh_2_)H], whereas for the second P−H activation, a novel transition state involving dual P−H bond cleavage of two PPh_2_H ligands is located. P−P bond activation from adducts of the type [Ni(NHC)_2_(Ph_2_P−PPh_2_)] (NHC=I*i*Pr_2_, IMe_4_,) is computed to be an extremely facile process, with barriers below 10 kcal mol^−1^ to form *cis*‐[Ni(NHC)_2_(PPh_2_)_2_]. In this case *cis–trans* isomerisation has a higher barrier but remains readily accessible.

The ability of the [Ni(NHC)_2_] moiety to afford isolable terminal phosphido products is in marked contrast to previous efforts to prepare analogues based on a [Ni(PR_3_)_2_] core, all of which have been reported to be unstable even at low temperatures. The latter often react on to form bridging phosphido dimers and higher nuclearity species,^[20]^ suggesting phosphine loss as a key process in this onward reactivity. The higher binding energies of NHC ligands^[59]^ may therefore contribute to the greater stabilities exhibited here. To quantify this hypothesis, calculations on ligand dissociation and subsequent dimer formation were performed on *trans*‐[NiL_2_(PH_2_)_2_] (L=PH_3_, IMe_2_; IMe_2_=1,3‐dimethylimidazolin‐2‐ylidene) where these simple model systems were employed to probe electronic effects directly.

**Scheme 10:**
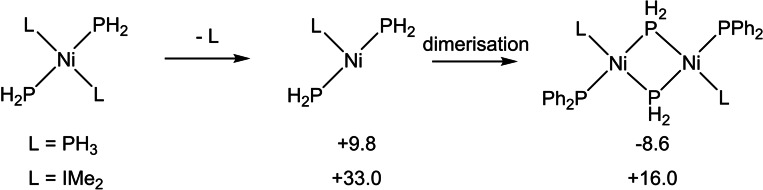
Computed free energies [kcal mol^−1^, B97D(benzene, BS2)//BP86(BS1)] for ligand loss and dimerization in *trans*‐[NiL_2_(PH_2_)_2_] (L=PH_3_, IMe_2_).

As shown in Scheme 10, phosphine dissociation from *trans*‐[Ni(PH_3_)_2_(PH_2_)_2_] is significantly more accessible than NHC loss from *trans*‐[Ni(IMe_2_)_2_(PH_2_)_2_]. Moreover, subsequent dimer formation is exergonic for the phosphine system, but strongly endergonic with the NHC analogue. This provides some insight into the remarkable ability of the [Ni(NHC)_2_] fragment to not only effect a wide range of novel bond activation processes, but also to stabilize the resultant products.

## Conflict of interest

The authors declare no conflict of interest.

## Supporting information

As a service to our authors and readers, this journal provides supporting information supplied by the authors. Such materials are peer reviewed and may be re‐organized for online delivery, but are not copy‐edited or typeset. Technical support issues arising from supporting information (other than missing files) should be addressed to the authors.

Supporting Information
