# Supplementary material for: [Ni(NHC)2] as a Scaffold for Structurally Characterized trans [H−Ni−PR2] and trans [R2P−Ni−PR2] Complexes
Source: Chemistry. 2021 Aug 4;27(52):13221–34. doi: 10.1002/chem.202101484 (PMC8518396; doi:10.1002/chem.202101484)
Supplement: Supplementary file 1 — Supporting Information [file CHEM-27-13221-s001.pdf]

Chemistry–A European Journal

Supporting Information

$[Ni(NHC)_2]$ as a Scaffold for Structurally Characterized *trans* $[H{-}Ni{-}PR_2]$ and *trans* $[R_2P{-}Ni{-}PR_2]$ Complexes

Sara Sabater[+], David Schmidt[+], Heidi (née Schneider) Schmidt, Maximilian W. Kuntze-Fechner, Thomas Zell, Connie J. Isaac, Nasir A. Rajabi, Harry Grieve, William J. M. Blackaby, John P. Lowe, Stuart A. Macgregor,* Mary F. Mahon, Udo Radius,* and Michael K. Whittlesey*

S-1 Experimental..S2
Experimental details S2-S14
Multinuclear NMR spectra of complexes **2-22** S15-S67
Multinuclear NMR spectra and molecular structure of $[Ni(IEt_2Me_2)]_2(\mu\text{-}PPh_2)_2$..... S68-S72
Crystal data for compounds **2-4**, **6-7**, **11**, **16-19** and **21-22**…………………..............S73
S-2 Computational S75
Computational Details………………………………………………………………….………..S75
Functional Testing……………………………………………………………...……S76-S88
S-3. Computed Cartesian coordinates (Å) and energies (au)……………………….. S90-S160
S-4. References………………………………………………………………………….S169

S-1 Experimental

All manipulations were carried out using standard Schlenk, high vacuum and glovebox techniques. Solvents were purified using an MBraun SPS solvent system (hexane) or under a nitrogen atmosphere from sodium benzophenone ketyl (benzene, THF). $[D_6]$benzene, $[D_8]$THF and $[D_8]$toluene were vacuum *trans*ferred from potassium. NMR spectra were recorded on Bruker Avance 200, 300, 400 or 500 and Agilent 500 MHz NMR spectrometers and referenced to solvent signals (benzene: δ = 7.16 ppm (^{1}H), δ = 128.0 ppm (^{13}C); toluene; δ = 2.09 ppm, δ = 20.4 ppm; THF: δ = 3.58 ppm , δ = 23.6 ppm). ^{31}P{^{1}H} spectra were run either broad band decoupled or, for quantitative determinations, with inverse-gated decoupling (d1 = 10-15 s) and were referenced externally to 85% H_3PO_4 (δ 0.0). In terms of signal multiplicities, 'vm' = virtual multiplet, 'vt' = virtual triplet. Infrared spectra were recorded on a Nicolet 380 FT-IR spectrometer as solids using an ATR unit, as well as on a Bruker Alpha Platinum-ATR-spectrometer in a glovebox. High resolution mass analyses were performed on a Thermo Scientific Exactive Plus mass spectrometer, equipped with an Orbitrap Mass Analyzer. Measurements were accomplished using an ASAP/APCI source with a corona needle, and a carrier-gas (N_2) temperature of 250 °C. Elemental analyses were performed by Elemental

Microanalysis Ltd, Okehampton, Devon, UK or in the Institut für Anorganische Chemie at Julius-Maximilians-Universität, Würzburg, Germany. [Ni(COD)$_2$] was purchased (Strem) or prepared using literature methods.[1] [Ni_2(I^{i}Pr$_2$)$_4$(COD)], I^{i}Pr$_2$, IMe$_4$ and IEt$_2$Me$_2$ were prepared according to literature methods.[2-4] PPh$_2$H, PPhMeH, PPhH$_2$, P(*para*-Tol)H$_2$ and PMesH$_2$ were either purchased (Strem) or prepared using standard procedures from PCl_3, Grignard reagents and $LiAlH_4$.[5] Ph$_2$P-PPh$_2$ and PPhMe-PPhMe used in Würzburg were prepared according to the literature[6] while PPh$_2$-PPh$_2$ used in Bath was a kind gift from Dr Danila Gasperini in the Webster group in Bath.

***trans*-[Ni(IEt$_2$Me$_2$)$_2$(PPh$_2$)Ph] (2).** [Ni(COD)$_2$] (31 mg, 0.11 mmol) was added to a solution of IEt$_2$Me$_2$ (42 mg, 0.28 mmol) and PPh$_3$ (30 mg, 0.11 mmol) in benzene (2 mL) in a J. Young resealable ampoule and the solution heated at 50 °C for 2.5 h. After removal of the solvent, the residue was redissolved in toluene and layered with pentane to afford red/orange crystals. Yield: 32 mg (45%). ^{1}H NMR (500 MHz, [D$_6$]benzene, 298 K): δ = 7.41-7.27 (m, 6H, Ph), 7.08-7.01 (m, 2H, Ph), 6.95-6.83 (m, 7H, Ph), 5.05 (m, 4H, NC*H$_2$*), 3.94 (m, 4H, NC*H$_2$*), 1.44 (s, 12H, NCC*H$_3$*), 1.25 (t, ^3J(H,H) = 6.9 Hz, 12H, NCH$_2$C*H*$_3$) ppm; ^{31}P{^{1}H} NMR (202 MHz, [D$_6$]benzene, 298 K): δ = 22.4 (s) ppm; ^{13}C{^{1}H} NMR (126 MHz, [D$_6$]benzene, 298 K): δ = 187.5 (d, ^2J(C,P) = 17 Hz, N*C*N), 165.9 (d, ^1J(C,P) = 40 Hz, *ipso*-*C*-PPh$_2$), 150.6 (d, ^2J(C,P) = 31 Hz, *ipso*-*C*-Ph), 140.4 (s), 134.2 (d, J(C,P) = 15 Hz, Ph), 126.5 (d, J(C,P) = 6 Hz, Ph), 126.1 (d, J(C,P) = 2 Hz, Ph), 124.2 (s), 123.2 (s), 121.5 (s), 43.3 (d, ^4J(C,P) = 5 Hz, N*C*H$_2$), 15.7 (s, NCH$_2$*C*H$_3$), 8.6 (s, NC*C*H$_3$) ppm; elemental analysis (%) calcd for $C_{36}H_{47}N_4PNi$ (625.4): C 69.13, H 7.58, N, 8.96; found: C 69.04, H 7.50, N, 9.00.

***trans*-[Ni(I^{i}Pr$_2$)$_2$(PPh$_2$)H] (3).** PPh$_2$H (74.5 mg; 69.6 µl, 400 µmol) was added at room temperature to a suspension of [Ni_2(I^{i}Pr$_2$)$_4$(COD)] (168 mg, 200 µmol) in 20 mL toluene. After stirring the resulting brownish solution for 30 minutes at room temperature all volatiles were removed *in vacuo* and the residue was suspended in 10 mL hexane. The product was collected by filtration, washed twice with 5 mL hexane and dried *in vacuo* to afford 210 mg (96 %) of a

yellow solid. Single crystals suitable for X-ray diffraction were obtained by cooling down a saturated solution of **3** in toluene to -30 °C. ^{1}H NMR (200 MHz, [D$_6$]benzene, 296 K): δ = 7.38 (m, 4H, Ph), 7.00 (m, 4H, Ph), 6.89 (m, 2H, Ph), 6.37 (s, 4H, NCH=C*H*N), 5.77 (d sept, 4H, ^{3}J(H,H) = 7 Hz, ^{5}J(H,P) = 2 Hz, C*H*Me$_2$), 1.29 (br s, 12H, CH*Me*$_2$), 1.17 (br s, 12H, CH*Me*$_2$), -11.31 (d, 1H, ^{2}J(H,P) = 70.0 Hz, Ni*H*) ppm; ^{31}P NMR (82 MHz, [D$_6$]benzene, 296 K): δ = 25.1 (dm, ^{2}J(P,H) =73 Hz) ppm; ^{13}C{^{1}H} NMR (50 MHz, [D$_6$]benzene, 296 K): δ = 189.3 (d, ^{2}J(C,P) = 11 Hz, N*C*N), 151.5 (d, ^{1}J(C,P) = 29 Hz, *ipso*-*C*-Ph), 133.7 (d, ^{2}J(C,P) = 16 Hz, *ortho*-*C*-Ph), 127.1 (d, ^{3}J(C,P) = 6 Hz, *meta*-*C*-Ph), 115.5 (NCH=*C*HN), 123.0 (*para*-*C*-Ph), 51.7 (d, ^{4}J(C,P) = 6 Hz, *C*HMe$_2$), 22.1 (br s, CH*Me*$_2$), 23.2 (br s, CH*Me*$_2$) ppm; IR (ATR): $\tilde{\nu}$/cm^{-1} = 1716 (s, $\nu_{Ni\text{-}H}$); elemental analysis (%) calcd for $C_{30}H_{43}N_4NiP$ (549.35): C 65.52, H 7.89, N 10.20; found C 65.52, H 7.88, N 10.12.

***trans*-[Ni(IEt$_2$Me$_2$)$_2$(PPh$_2$)H] (4).** [Ni(COD)$_2$] (25 mg, 0.091 mmol) was added to a solution of IEt$_2$Me$_2$ (29 mg, 0.19 mmol) and PPh$_2$H (13.6 µL, 0.078 mmol) in [D$_6$]benzene (0.6 mL) in a J. Young resealable NMR tube and the solution shaken for 2.5 h at room temperature. After removal of the solvent, the residue was crystallized from a concentrated Et$_2$O/pentane solution to afford **4** as yellow/orange crystals. Yield: 20 mg (40%). ^{1}H NMR (400 MHz, [D$_8$]THF, 235 K): δ = 6.85-6.79 (m, 5H, PPh$_2$), 6.73-6.62 (m, 5H, PPh$_2$), 4.66 (m, 4H, NC*H*$_2$CH$_3$), 3.75 (m, 4H, NC*H*$_2$CH$_3$), 1.98 (s, 12H, NCC*H*$_3$), 1.37 (t, ^{3}J(H,H) = 7.2 Hz, 12H, NCH$_2$C*H*$_3$), -11.64 (d, ^{2}J(H,P) = 73.2 Hz, 1H, Ni*H*) ppm; ^{31}P{^{1}H} NMR (162 MHz, [D$_8$]THF, 235 K): δ = 18.2 (s) ppm; ^{13}C{^{1}H} NMR (101 MHz, [D$_8$]THF, 235 K): δ = 188.0 (d, ^{2}J(C,P) = 11 Hz, N*C*N), 152.3 (d, ^{2}J(C,P) = 30 Hz, *ipso*-*C*-PPh$_2$), 133.0 (d, *J*(C,P) = 15 Hz, *ortho*-*C*-PPh$_2$), 126.7 (d, *J*(C,P) = 5 Hz, *meta*-*C*-PPh$_2$), 124.0 (s, *para*-*C*-PPh$_2$), 122.4 (s, N*C*CH$_3$=CCH$_3$N), 43.1 (s, N*C*H$_2$CH$_3$), 15.8 (s, NCH$_2$*C*H$_3$), 8.7 (s, NC*C*H$_3$=CCH$_3$N) ppm; elemental analysis (%) for $C_{30}H_{44}N_4PNi$ (549.33): C, 65.59; H, 7.89; N, 10.20; found: C, 65.53; H, 7.89; N, 10.29.

***trans*-[Ni(IMe$_4$)$_2$(PPh$_2$)H] (5).** [Ni(COD)$_2$] (15 mg, 0.055 mmol) was added to a solution of IMe$_4$ (14 mg, 0.11 mmol) and PPh$_2$H (9.5 µL, 0.055 mmol) in [D$_8$]toluene (0.6 mL)

in a J. Youngs resealable NMR tube and the solution rapidly transferred to a pre-cooled (-62 °C) NMR spectrometer for characterization of **5**. Selected ^{1}H NMR (400 MHz, [D_8]toluene, 211 K): δ = 3.56 (s, 12H, N*CH₃*), 1.42 (s, 12H, NCC*H₃*), -10.97 (d, ^{2}J(H,P) = 68.0 Hz, 1H, Ni*H*) ppm; $^{31}P\{^{1}H\}$ NMR (162 MHz, [D_8]toluene, -62 °C): δ = 19.3 (s) ppm; $^{13}C\{^{1}H\}$ NMR (101 MHz, [D_8]toluene, 211 K): δ = 188.3 (d, ^{2}J(C,P) = 10 Hz, N*C*N), 151.6 (d, ^{2}J(C,P) = 30 Hz, *ipso*-*C*-PPh_2) ppm.

***trans*-[Ni(IMe$_4$)$_2$(PPh$_2$)$_2$] (6).** [Ni(COD)$_2$] (22 mg, 0.08 mmol) was added to a solution of IMe_4 (21 mg, 0.17 mmol) and PPh_2H (14 μL, 0.08 mmol) in [D_6]benzene (0.6 mL) in a J. Young resealable NMR tube and the suspension shaken at room temperature for 24 h to afford a deep red homogeneous solution. After removal of the solvent, Et_2O (1 mL) was added and the suspension left to stand for 72 h at room temperature to give a red microcrystalline solid. Yield: 8 mg (15%). Recrystallization from benzene/hexane afforded material appropriate for X-ray crystallography. ^{1}H NMR (500 MHz, [D_6]benzene, 298 K): δ = 7.37-7.33 (m, 8H, PPh_2), 6.85-6.82 (m, 12H, PPh_2), 3.67 (s, 12H, N$C\!H_3$), 1.25 (s, 12H, NCCH_3) ppm; $^{31}P\{^{1}H\}$ NMR ([D_6]benzene, 202 MHz, 298 K): δ = 31.2 (s) ppm; $^{13}C\{^{1}H\}$ NMR (126 MHz, [D_6]benzene, 298 K): δ = 182.7 (t, ^{2}J(C,P) = 182.7, N*C*N), 149.3 (vm, *ipso*-*C*-PPh_2), 134.2 (vt, *J*(C,P) = 10 Hz, PPh_2), 126.5 (vt, *J*(C,P) = 3 Hz, PPh_2), 124.9 (s, N*C*CH$_3$=*C*CH$_3$N), 123.8 (s, PPh_2), 37.7 (t, ^{4}J(C,P) = 6 Hz, N*C*H$_3$), 8.4 (s, NC*C*H$_3$=CCH$_3$N); elemental analysis (%) calcd for $C_{38}H_{44}N_4P_2Ni$ (677.42): C 67.37, H 6.54, N 8.27; found: C 66.88, H 6.71, N, 8.48.

***trans*-[Ni(IEt$_2$Me$_2$)$_2$(PPh$_2$)$_2$] (7).** [Ni(COD)$_2$] (10 mg, 0.036 mmol), IEt_2Me_2 (11 mg, 0.072 mmol) and Ph_2P-PPh_2 (14 mg, 0.037 mmol) were dissolved in [D_6]benzene (0.6 mL) in a J. Youngs resealable NMR tube and the solution shaken for 14 h at room temperature to afford a red microcrystalline solid. The crystals were isolated by filtration, the filtrate reduced to dryness and recrystallized from benzene/pentane to afford further red crystals of **7**. Yield: 13 mg (49%). The product was also accessible by reaction of **4** with PPh_2H or Ph_2P-PPh_2. ^{1}H NMR (500 MHz, [D_6]benzene, 298 K): δ = 7.22-7.17 (m, 6H, PPh_2), 7.00-6.94 (m, 2H, PPh_2), 6.88-6.83 (m, 12H,

PPh_2), 4.26 (q, ^3J(H,H) = 7.3 Hz, 8H, NC$H_2$$CH_3$), 1.49 (t, ^3J(H,H) = 7.3 Hz, 12H, NCH_2CH_3), 1.42 (s, 12H, NCCH_3=CCH_3N) ppm; $^{31}P\{^1H\}$ NMR (202 MHz, [D$_6$]benzene, 298 K): δ = 35.6 (s) ppm; $^{13}C\{^1H\}$ NMR (126 MHz, [D$_6$]benzene, 298 K): δ = 181.2 (t, ^2J(C,P) = 17 Hz, N*C*N), 149.2 (vm, *ipso*-*C*-Ph), 134.5 (vt, *J*(C,P) = 8 Hz, Ph), 126.6 (vt, *J(C,P)* = 2 Hz, Ph), 125.4 (s, N*C*CH_3=CCH_3N), 123.8 (s, *para*-*C*-Ph), 43.5 (s, N*C*$H_2$$CH_3$), 15.9 (s, NCH_2*C*H_3), 8.6 (s, NC*C*H_3=CCH_3N) ppm; elemental analysis (%) calcd for $C_{42}H_{52}N_4P_2Ni$ (733.48): C 68.77, H 7.15, N 7.64; found values were agreeable for %H and %N, but consistently low for %C: e.g. C 67.96, H 7.28, N 7.36.

***trans*-[Ni(I^{i}Pr$_2$)$_2$(PPh$_2$)$_2$] (8).** To a suspension of 200 mg [Ni$_2$(I^{i}Pr$_2$Im)$_4$(COD)] (240 μmol) in 10 mL toluene 178 mg Ph_2P-PPh_2 (480 μmol) were added at room temperature. After stirring the resulting red solution for 16 h at room temperature all volatiles were removed *in vacuo* and the residue was suspended in 10 mL hexane. The product was collected by filtration, washed twice with 5 mL hexane and dried *in vacuo* to afford 253 mg (72 % yield) of red solid. ^1H NMR (200 MHz, [D$_6$]benzene, 298 K): δ = 6.98-6.84 (m, 20H, Ph), 6.40 (s, 4H, NC*H*=CHN), 5.78 (sept, ^3J(H,H) = 7 Hz, 4H, C*H*Me_2), 1.12 (d, ^3J(H,P) = 7 Hz, 24H, CH*Me*$_2$) ppm; ^{31}P NMR (81 MHz, [D$_6$]benzene, 296 K): δ = 36.9 (s) ppm; $^{13}C\{^1H\}$ NMR (50 MHz, [D$_6$]benzene, 298 K): δ = 182.8 (t, ^2J(C,P) = 17 Hz, N*C*N), 149.3 (vm, *ipso*-*C*-Ph), 135.4 (vt, $|^2J(C,P) + {}^4J(C,P)|$ = 9 Hz, *ortho*-*C*-Ph), 127.3 (vt, $|^3J(C,P) + {}^5J(C,P)|$ = 3 Hz, *meta*-*C*-Ph), 124.2 (s, *para*-*C*-Ph), 117.7 (s, N*C*H=CHN), 52.1 (s, *C*HMe_2), 23.4 (s, CH*Me*$_2$) ppm; elemental analysis (%) calcd for $C_{30}H_{43}N_4NiP$ (733.53): C 68.77, H 7.15, N 7.64; found C 68.22, H 7.43, N 7.63.

***trans*-[Ni(I^{i}Pr$_2$)$_2$(PPhMe)$_2$] (9).** PhMeP-PMePh (59 mg, 240 μmol) was added at room temperature to a suspension of [Ni$_2$(I^{i}Pr$_2$)$_4$(COD)] (100 mg, 120 μmol) in 10 mL of toluene. After stirring the resulting orange solution for 1 day at room temperature all volatiles were removed *in vacuo* and the residue was suspended in 10 mL of hexane. The product was collected by filtration, washed twice with 5 mL of hexane and dried *in vacuo* to afford 25 mg (41 μmol,

yield: 34 %) of an orange solid. ^{1}H NMR (200 MHz, [D$_6$]benzene, 298 K): δ = 7.01-6.95 (m, 4H, Ph), 6.91–6.84 (m, 6H, Ph), 6.41 (s, 4H, NC*H*=CHN), 5.66 (sept, ^{3}J(H,H) = 7 Hz, 4H, C*H*Me$_2$), 1.29 (d, ^{3}J(H,H) = 7 Hz, 24H, CH*Me*$_2$), 0.89 (vt, |^{2}J(H,P) + ^{4}J(H,P)| = 7 Hz, 6H, PC*H*$_3$) ppm; ^{31}P NMR (202 MHz, [D$_6$]benzene, 298 K): δ = -8.2 (br s) ppm; ^{13}C{^{1}H} NMR (126 MHz, [D$_6$]benzene, 298 K): δ = 188.4 (t, ^{2}J(C,P) = 15 Hz, N*C*N), 154.5 (vt, |^{1}J(C,P) + ^{3}J(C,P)| = 37 Hz, *ipso*-*C*-Ph), 130.7 (vt, |^{2}J(C,P) + ^{4}J(C,P)| = 16 Hz, *ortho*-*C*-Ph), 127.4 (vt, |^{3}J(C,P) + ^{5}J(C,P)| = 4 Hz, *meta*-*C*-Ph), 122.5 (*para*-*C*-Ph), 117.0 (s, N*C*H=CHN), 52.0 (t, ^{4}J(C,P) = 4 Hz, *C*HMe$_2$), 23.5 (t, ^{5}J(C,P) = 2 Hz, CH*Me*$_2$), 8.9 (vt, |^{1}J(C,P) + ^{3}J(C,P)| = 28 Hz, P*C*H$_3$) ppm; elemental analysis (%) calcd for $C_{32}H_{48}N_4NiP_2$ (609.39): C 63.07, H 7.94, N 9.19; found C 63.33, H 7.87, N 8.51.

***trans*-[Ni(IMe$_4$)$_2$(PPhH)H] (10).** [Ni(COD)$_2$] (15 mg, 0.055 mmol) was added to a solution of IMe$_4$ (14 mg, 0.11 mmol) and PPhH$_2$ (6 µL, 0.055 mmol) in [D$_8$]toluene (0.6 mL) in a J. Youngs resealable NMR tube and the solution rapidly transferred to a pre-cooled (211 K) NMR spectrometer for characterization of **10**. Even under these conditions, **10** was present along with the bis-phosphido complex **11**. Selected ^{1}H NMR (400 MHz, [D$_8$]toluene, 211 K): δ = -10.04 (d, ^{2}J(H,P) = 58.0 Hz, 1H, Ni*H*) ppm; ^{31}P NMR (162 MHz, [D$_8$]toluene, 211 K): δ = -43.8 (dd, ^{1}J(P,H) = 190 Hz, $^{2}J_{PH}$ = 59 Hz) ppm; Selected ^{13}C{^{1}H} NMR (101 MHz, [D$_8$]toluene, 211 K): δ = 189.9 (d, ^{2}J(C,P) = 10 Hz, N*C*N), 154.6 (d, ^{2}J(C,P) = 31 Hz, *ipso*-*C*-PPh$_2$) ppm.

***trans*-[Ni(IMe$_4$)$_2$(PPhH)$_2$] (11).** [Ni(COD)$_2$] (33 mg, 0.12 mmol) was added to a solution of IMe$_4$ (31 mg, 0.25 mmol) and PPhH$_2$ (13 µL, 0.12 mmol) in [D$_6$]benzene (0.6 mL) in a J. Young resealable NMR tube and the solution shaken at room temperature for 5 days. This afforded orange-red crystals which proved suitable for X-ray diffraction. Yield 15 mg (24%). ^{1}H NMR (500 MHz, [D$_6$]benzene, 298 K): δ = 7.08-7.02 (m, 4H, Ph), 6.88-6.77 (m, 6H, Ph), 3.61 (s, 12H, NC*H$_3$*), 3.33 (AA′XX′ signal, 2H, P*H*Ph), 1.42 (s, 12H, NCC*H$_3$*=CCH$_3$N) ppm; ^{31}P{^{1}H} NMR ([D$_6$]benzene, 162 MHz, 298 K): δ = -40.3 (s) ppm; ^{13}C{^{1}H} NMR (126 MHz,

$[D_6]$benzene, 298 K): δ = 186.1 (t, ^2J(C,P) = 13 Hz, N*C*N), 151.4 (vm, *ipso*-*C*-PPh_2), 132.4 (vt, *J(C,P)* = 7 Hz, Ph), 126.7 (vt, *J(C,P)* = 2 Hz, Ph), 124.7 (s, N*C*CH_3=CCH_3N), 122.5 (s, Ph), 34.2 (t, ^4J(C,P) = 3 Hz, N*C*H_3), 8.4 (s, NC*C*H_3=CCH_3N) ppm; elemental analysis calcd for $C_{26}H_{36}N_4P_2Ni$ (525.19): C 59.46, H 6.91, N 10.67; found: C 59.55, H 6.87, N, 10.61.

***trans*-[Ni(IEt$_2$Me$_2$)$_2$(PPhH)H] (12).** [Ni(COD)$_2$] (15 mg, 0.055 mmol) was added to a solution of IEt$_2$Me$_2$ (18 mg, 0.12 mmol) and $PPhH_2$ (6 μL, 0.055 mmol) in $[D_8]$toluene (0.6 mL) in a J. Young resealable NMR tube and the solution rapidly transferred to a pre-cooled (223 K) NMR spectrometer for characterization of **12**. Selected ^{1}H NMR (400 MHz, $[D_8]$toluene, 236 K): δ = 4.46 (br m, 4H, NC*H*$_2$), 3.94 (br m, 4H, NC*H*$_2$), 3.25 (d, ^1J(H,P) = 185.6 Hz, 1H, PPh*H*), 1.43 (t, ^3J(H,H) = 6.0 Hz, 12H, NCH_2C*H*$_3$), -10.17 (d, ^2J(H,P) = 62.9 Hz, 1H, Ni*H*) ppm; ^{31}P{^{1}H} NMR (162 MHz, $[D_8]$toluene, 223 K): δ = -37.6 (s) ppm; Selected ^{13}C{^{1}H} NMR (126 MHz, $[D_8]$toluene, 223 K): δ = 189.2 (d, ^2J(C,P) = 10 Hz, N*C*N), 154.5 (d, ^2J(C,P) = 31 Hz, *ipso*-*C*-Ph) ppm.

***trans*-[Ni(IEt$_2$Me$_2$)$_2$(PPhH)$_2$] (13).** [Ni(COD)$_2$] (25 mg, 0.091 mmol) was added to a solution of IEt$_2$Me$_2$ (29 mg, 0.19 mmol) and $PPhH_2$ (10 μL, 0.091 mmol) in $[D_6]$benzene (0.6 mL) in a J. Young resealable NMR tube and the sample shaken at room temperature for 10 days. The volatiles were then removed and the residue recrystallised from Et_2O/pentane to afford 15 mg (27% yield) of a red crystalline solid. ^{1}H NMR (500 MHz, $[D_8]$THF, 298 K): δ = 6.73-6.54 (m, 10H, Ph), 4.18 (quart, ^3J(H,H) = 7.0 Hz, 8H, NC*H*$_2$), 2.64 (AA′XX′ signal (Figure S41 for simulation), 2H, PPh*H*), 2.02 (s, 12H, NCC*H*$_3$=$NCCH_3$), 1.49 (t, ^3J(H,H) = 7.0 Hz, 12H, NCH_2C*H*$_3$) ppm; ^{31}P{^{1}H) NMR (202 MHz, $[D_8]$THF, 298 K): δ = -28.2 (s) ppm; ^{13}C{^{1}H} NMR (126 MHz, $[D_8]$THF, 298 K): δ = 185.5 (t, ^2J(C,P) = 14 Hz, N*C*N), 152.0 (vt, $|^1J(C,P) + {}^3J(C,P)|$ = 14 Hz, *ipso*-*C*-Ph), 151.7 (vt, $|^1J(C,P) + {}^3J(C,P)|$ = 14 Hz, *ipso*-*C*-Ph), 132.4 (vt, $|^2J(C,P) + {}^4J(C,P)|$ = 6 Hz, *ortho*-*C*-Ph), 127.0 (s, *meta*-/*para*-*C*-Ph), 125.6 (s, N*C*CH_3=$NCCH_3$), 122.8 (s, *meta*-/*para*-*C*-Ph), 43.3 (s, N*C*H_2), 16.4 (t, ^4J(C,P) = 4 Hz, NC*C*H_3=$NCCH_3$), 8.8 (s, NCH_2*C*H_3)

ppm; elemental analysis (%) calcd for $C_{30}H_{44}N_4P_2Ni$ (581.34): C 61.98, H 7.63, N 9.64; found: C 61.99, H 7.67, N, 9.72.

***trans*-[Ni(I^{i}Pr$_2$)$_2$(PPhH)H] (14).** The reaction of $PPhH_2$ and [Ni$_2$(I^{i}Pr$_2$)$_4$(COD)] was monitored in [D$_6$]benzene and selected NMR data were identified in the very beginning of this reaction for complex **14.** ^{1}H NMR (500 MHz, [D$_6$]benzene, 296 K): δ = -10.41 (d, ^2J(H,P) = 62.1 Hz). ^{31}P NMR (202 MHz, [D$_6$]benzene, 296 K): δ = -41.2 ppm (dd, ^1J(P,H) = 195.5 Hz, ^2J(P,H) = 62.1 Hz) ppm.

***trans*-[Ni(I^{i}Pr$_2$)$_2${P(*para*-Tol)H}H] (15).** The reaction of P(*para*-Tol)H$_2$ and [Ni$_2$(I^{i}Pr$_2$)$_4$(COD)] was monitored in [D$_6$]benzene and a low frequency hydride signal identified at the very beginning of this reaction for complex **15.** ^{1}H NMR (500 MHz, [D$_6$]benzene, 296 K): δ = -10.71 (d, ^2J(H,P) = 60.3 Hz).

***trans*-[Ni(I^{i}Pr$_2$)$_2$(PPhH)$_2$] (16).** Isoprene (96 µl, 960 µmol) and $PPhH_2$ (106 µl, 960 µmol) were added at room temperature to a suspension of [Ni$_2$(I^{i}Pr$_2$)$_4$(COD)] (200 mg, 240 µmol) in 10 mL of toluene. After stirring the resulting brownish solution for 1 day at room temperature all volatiles were removed in vacuo and the residue suspended in 10 mL of hexane. The product was collected by filtration, washed twice with hexane (5 mL) and dried in vacuo to afford 140 mg (50 % yield) of an orange solid. Single crystals suitable for X-ray diffraction were obtained from a saturated solution of **16** in benzene at 5 °C. ^{1}H NMR (300 MHz, [D$_6$]benzene, 296 K): δ = 6.92-6.84 (m, 10H, PPh), 6.40 (s, 4H, NC*H*=CHN), 5.80 (sept, ^3J(H,H) = 7 Hz, 4H, C*H*Me$_2$), 3.11 (AA′XX′ signal, 2H, PPh*H*), 1.26 (d, ^3J(H,H) = 7 Hz, 24H, CH*Me*$_2$) ppm; ^{31}P NMR (202 MHz, [D$_6$]benzene, 296 K): δ = -29.5 (dm, ^1J(P,H) = 198 Hz). ^{13}C{^{1}H} NMR (75 MHz, [D$_6$]benzene, 296 K): δ = 186.3 (t, ^2J(C,P) = 14 Hz, N*C*N), 150.6 (vm, *ipso*-*C*-Ph), 134.0 (vt, $|^2J(C,P) + {}^4J(C,P)|$ = 13 Hz, *ortho*-*C*-Ph), 131.8 (vt, $|^3J(C,P) + {}^5J(C,P)|$ = 7 Hz, *meta*-*C*-Ph), 123.0 (s, *para*-*C*-PPh$_2$), 117.0 (s, N*C*H=CHN), 52.1 (t, ^4J(C,P) = 3 Hz, *C*HMe$_2$), 23.4 (s, CH*Me*$_2$) ppm; IR (ATR): $\tilde{\nu}$/cm^{-1} = 2284 (w, $\nu_{P\text{-}H}$); elemental analysis (%) calcd for $C_{30}H_{44}N_4P_2Ni$ (581.34): C 61.98, H 7.63, N 9.64; found: C 61.96, H 7.70, N 9.52.

***trans*-[Ni(I^{i}Pr$_2$)$_2${P(*para*-Tol)H}$_2$] (17).** As for **16** with P(*para*-Tol)H_2 (199 µl, 960 µmol) to give 152 mg (52 % yield) of **17**. ^{1}H NMR (500 MHz, [D$_6$]benzene, 363 K): δ = 6.88-6.78 (m, 4H, Tol), 6.78-6.68 (m, 4H, Tol), 6.43 (s, 4H, NC*H*=CHN), 5.82 (sept, ^3J(H,H) = 7 Hz, 4H, C*H*Me$_2$), 3.11 (AA′XX′ signal, 2H, P(*para*-Tol)*H*), 2.09 (s, 6H, *para*-Me), 1.28 (d, ^3J(H,H) = 6.8 Hz, 24H, CH*Me*$_2$) ppm; ^{31}P NMR (202 MHz, [D$_6$]benzene, 296 K): δ = -31.0 (AA′XX′ signal) ppm; ^{13}C{^{1}H} NMR (126 MHz, [D$_6$]benzene, 296 K): δ = 190.0 (t, ^2J(C,P) = 13 Hz, N*C*N), 146.4 (vm, *ipso*-*C*-Tol), 134.0 (vt, |^2J(C,P) + ^4J(C,P)| = 13 Hz, *ortho*-*C*-Tol) 131.9 (vt, |^3J(C,P) + ^5J(C,P)| = 13 Hz, *meta*-*C*-Tol), 131.8 (s, *para*-*C*-Tol), 128.5 (s, Tol), 116.9 (s, N*C*H=CHN), 52.0 (t, ^4J(C,P) = 3 Hz, *C*HMe$_2$), 23.4 (s, CH*Me*$_2$), 21.1 (s, *para-Me*) ppm; IR (ATR): ṽ/cm^{-1} = 2243 (m, $\nu_{P\text{-}H}$); elemental analysis calcd for $C_{30}H_{48}N_4P_2Ni$ (609.39): C 63.07, H 7.94, N 9.19; found: C 61.93, H 7.90, N 9.77.

[Ni(I^{i}Pr$_2$)$_2$(η^2-PhP=PPh)] (18). *Method A:* PPhH_2 (106 mg, 106 µl, 960 µmol) was added at room temperature to a suspension of [Ni$_2$(I^{i}Pr$_2$)$_4$(COD)] (200 mg, 240 µmol) in 10 mL of xylene. After heating the resulting brownish suspension for 2 days to 140 °C, the volatiles were removed in vacuo and the residue suspended in 10 mL of hexane. The product was collected by filtration, washed twice with 5 mL of hexane and dried in vacuo to afford 140 mg (50 % yield) of an orange solid. *Method B:* A solution of *trans*-[Ni(I^{i}Pr$_2$)$_2$(PPhH)$_2$] (**16**) (11 mg, 18.9 µmol) in toluene (1 mL) was heated to 140 °C. After 24 h, quantitative conversion to **18** was observed by NMR spectroscopy. Single crystals suitable for X-ray diffraction were obtained by cooling a saturated boiling toluene solution of **18** to room temperature. ^{1}H NMR (500 MHz, [D$_6$]benzene, 296 K): δ = 7.76-7.74 (m, 2H, *ortho*-Ph), 7.02-6.99 (m, 4H, *meta*-Ph), 6.97–6.93 (m, 2H, *para*-Ph), 6.44 (br s, 2H, NC*H*=CHN), 6.36 (br s, 2H, NCH=C*H*N), 5.73 (br s, 2H, C*H*Me$_2$), 4.76 (br s, 2H, C*H*Me$_2$), 1.61 (br s, 6H, CH*Me*$_2$), 1.01 (br s, 6H, CH*Me*$_2$), 0.89 (br s, 6H, CH*Me*$_2$), 0.51 (br s, 6H, CH*Me*$_2$) ppm; ^{1}H NMR (200 MHz, [D$_8$]toluene, 363 K): δ = 7.63-7.48 (m, 4H, Ph), 6.90-6.81 (m, 4H, Ph), 6.47 (s, 4H, NC*H*=CHN), 5.16 (br s, 4H, C*H*Me$_2$), 1.05

(br s, 12H, CH*Me*$_2$), 0.97 (d, ^3J(H,H) = 7 Hz, 12H, CH*Me*$_2$) ppm; ^{1}H NMR (200 MHz, [D$_8$]toluene, 243 K): δ = 7.73-7.63 (m, 4H, Ph), 6.97–6.92 (m, 4H, Ph), 6.33 (d, ^3J(H,H) = 7 Hz, 2H, NC*H*=CHN), 6.26 (d, ^3J(H,H) = 7 Hz, 2H, NCH=C*H*N), 5.66 (sept, ^3J(H,H) = 7 Hz, 2H, C*H*Me$_2$), 4.78 (sept, ^3J(H,H) = 7 Hz, 2H, C*H*Me$_2$), 1.57 (d, ^3J(H,H) = 7 Hz, 6H, CH*Me*$_2$), 0.95 (d, ^3J(H,H) = 6.7 Hz, 6H, CH*Me*$_2$), 0.89 (d, ^3J(H,H) = 7 Hz, 6H, CH*Me*$_2$), 0.46 (d, ^3J(H,H) = 7 Hz, 6H, CH*Me*$_2$) ppm; ^{31}P NMR (202 MHz, [D$_6$]benzene, 296 K): δ = -40.7 (s) ppm; ^{13}C{^{1}H} NMR (126, [D$_6$]benzene, 296 K): δ = 193.9 (t, ^2J(C,P) = 8 Hz, N*C*N), 127.5 (dd, ^1J(C,P) = 19 Hz, ^2J(C,P) = 18 Hz, *ipso*-*C*-Ph), 134.1 (vt, $|^2J(C,P) + {}^4J(C,P)|$ = 13 Hz, *ortho*-*C*-Ph), 127.5 (vt, $|^3J(C,P) + {}^5J(C,P)|$ = 3 Hz, *meta*-*C*-Ph), 124.0 (br s, *para*-*C*-Ph), 116.4 (br s, N*C*H=CHN), 52.2 (br s, *C*HMe$_2$), 51.3 (br s, *C*HMe$_2$), 24.8 (br s, CH*Me*$_2$), 22.7 (br s, CH*Me*$_2$) ppm; elemental analysis calcd for $C_{30}H_{44}N_4NiP_2$ (579.32): C 62.20, H 7.31, N 9.67; found: C 61.72, H 7.70, N 9.65.

[Ni(I^{i}Pr$_2$)$_2$(η^2-{(*para*-Tol)P=P(*para*-Tol)}] (19). *Method A*: As for **18** using P(*para*-Tol)H$_2$ (199 µl, 960 µmol) to afford 149 mg (51% yield) of **19** as a dark orange solid. *Method B*: As for **18** using *trans*-[Ni(I^{i}Pr$_2$)$_2${P(*para*-Tol)H}$_2$] **17** (11 mg, 18.9 µmol). ^{1}H NMR (500 MHz, [D$_6$]benzene, 363 K): δ = 7.70-7.68 (d, ^3J(H,H) = 8 Hz, 2H, Tol), 6.85-6.83 (d, ^3J(H,H) = 8 Hz, 2H, Tol), 6.45 (br s, 2H, NC*H*=CHN), 6.40 (br s, 2H, NCH=C*H*N), 5.78 (br s, 2H, C*H*Me$_2$), 4.81 (br s, 2H, C*H*Me$_2$), 2.50 (s, 6H, *para*-Me), 1.63 (br s, 6H, CH*Me*$_2$), 1.03 (br s, 6H, CH*Me*$_2$), 0.95 (br s, 6H, CH*Me*$_2$), 0.53 (br s, 6H, CH*Me*$_2$) ppm; ^{31}P NMR (202 MHz, [D$_6$]benzene, 296 K): δ -39.4 (s) ppm; ^{13}C{^{1}H} NMR (126 MHz, [D$_6$]benzene, 296 K): δ = 194.3 (t, ^2J(C,P) = 8 Hz, N*C*N), 147.5 (vt, $|^1J(C,P) + {}^2J(C,P)|$ = 36 Hz, *ipso*-*C*-Tol), 134.1 (vt, $|^2J(C,P) + {}^3J(C,P)|$ = 25 Hz, *ortho*-*C*-Tol), 132.9 (s, *meta*-*C*-Tol), 128.4 (s, *para*-*C*-Tol), 116.4 (br s, N*C*H=CHN), 116.2 (br s, NCH=*C*HN), 52.2 (br s, *C*HMe$_2$), 51.2 (br s, *C*HMe$_2$), 24.9 (br s, CH*Me*$_2$), 22.8 (br s, CH*Me*$_2$) ppm; elemental analysis (%) calcd for $C_{30}H_{46}N_4NiP_2$ (607.38): C 63.28, H 7.63, N 9.22; found: C 63.04, H 7.79, N, 9.97.

***trans*-[Ni(I^{i}Pr$_2$)$_2$(PMesH)H] (20).** PMesH$_2$ (73 mg, 73.0 µl, 480 µmol) was added at room temperature to a suspension of [Ni$_2$(I^{i}Pr$_2$)$_4$(COD)] (200 mg, 240 µmol) in 10 mL of toluene. After stirring the resulting yellow greenish solution for 1 day at room temperature, the volatiles were removed to give 236 mg of **20** as a sticky brownish oil in quantitative (> 95 %) yield. This proved to very soluble in all common organic solvents, even at -80 °C. ^{1}H NMR (200 MHz, [D$_6$]benzene, 298 K): δ = 6.78 (s, 2H, Mes), 6.43 (s, 4H, NC*H*=CHN), 5.65 (dsept, ^3J(H,H) = 7 Hz, ^5J(H,H) = 1.3 Hz, 4H, C*H*Me$_2$), 2.94 (dd, ^1J(H,P) = 200.5 Hz, ^3J(H,H) = 2 Hz, 1H, PMes*H*), 2.40 (s, 6H, *ortho-Me*), 2.21 (s, 3H, *para-Me*), 1.26 (br s, 24H, CH*Me*$_2$), -11.05 (dd, ^2J(H,H) = 61 Hz, ^3J(H,H) = 2 Hz, 1H, Ni*H*) ppm; ^{31}P NMR (81 MHz, C$_6$H$_6$, 298 K): δ = -90.6 (dd, ^1J(P,H) =200 Hz, ^2J(P,H) = 61 Hz) ppm; ^{13}C{^{1}H} NMR (50 MHz, [D$_6$]benzene, 298 K): δ = 191.0 (d, ^2J(C,P) = 9 Hz, N*C*N), 146.7 (d, ^1J(C,P) = 38 Hz, *ipso-C*-Mes), 140.0 (d, ^2J(C,P) = 9 Hz, *ortho-C*-Mes), 130.3 (s, *para-C*-Mes), 127.8 (d, ^3J(C,P) = 3 Hz, *meta-C*-Mes), 115.5 (s, N*C*H=CHN), 51.7 (d, ^4J(C,P) = 3 Hz, *C*HMe$_2$), 24.3 (d, ^3J(C,P) = 11 Hz, *ortho*-Me), 23.2 (s, CH*Me*$_2$), 22.5 (s, CH*Me*$_2$), 22.1 (s, *para*-Me) ppm; IR (ATR): ṽ/cm^{-1} = 1738 (w, $\nu_{\text{Ni-H}}$), 2292 (w, $\nu_{\text{P-H}}$).

***trans*-[Ni(I^{i}Pr$_2$)$_2$(PMesH)$_2$] (21).** PMesH$_2$ (146 mg, 146 µl, 960 µmol) was added at room temperature to a suspension of [Ni$_2$(I^{i}Pr$_2$)$_4$(COD)] (200 mg, 240 µmol) in 10 mL of toluene. After stirring the resulting yellow greenish solution for 1 day at room temperature, the volatiles were removed and the residue suspended in hexane (10 mL). The product was collected by filtration, washed twice with 5 mL of hexane and dried in vacuo to afford 101 mg of a brownish solid (yield 31 %). Single crystals suitable for X-ray diffraction were obtained from a saturated solution of **21** in toluene at -30 °C. ^{1}H NMR (500 MHz, [D$_6$]benzene, 298 K): δ = 6.75 (m, 4H, Mes), 6.32 (s, 4H, NC*H*=CHN), 5.51 (sept, ^3J(H,H) = 7 Hz, 4H, C*H*Me$_2$), 2.94 (AA′XX′ signal, 2H, PMes*H*), 2.21 (s, 12H, *ortho-Me*), 2.16 (s, 6H, *para-Me*), 1.34 (d, ^3J(H,H) = 7 Hz, 24H, CH*Me*$_2$) ppm; ^{31}P NMR (202 MHz, [D$_6$]benzene, 298 K): δ = -71.2 (AA′XX′ signal) ppm;

$^{13}C\{^1H\}$ NMR (126 MHz, [D$_6$]benzene, 298 K): δ = 185.9 (t, ^2J(C,P) = 13 Hz, N*C*N), 143.9 (vm, *ipso*-*C*-Mes), 141.6 (t, |^3J(C,P) + ^5J(C,P)| = 5 Hz, *ortho*-*C*-Mes), 132.7 (s, *para*-*C*-Mes), 116.9 (s, N*C*H=CHN), 52.0 (s, *C*HMe$_2$), 25.1 (s, *ortho*-Me), 24.0 (s, CH*Me*$_2$), 21.0 (s, *para*-Me) ppm; IR (ATR): ṽ/cm^{-1} = 2293 (s, $\nu_{P\text{-}H}$); elemental analysis (%) calcd. for $C_{36}H_{56}N_4NiP_2$ (665.50): C 64.97, H 8.48, N 8.42; found: C 64.61, H 8.70, N 8.37.

***trans*-[Ni(I^{i}Pr$_2$)$_2$(PH$_2$)$_2$] (22).** PH_3 was generated *in situ* by the slow addition of P(OPh)$_3$ (629 µl, 2.40 mmol) to a suspension of $LiAlH_4$ (546 mg, 14.4 mmol) in 10 mL Et_2O. The emerging gaseous PH_3 was passed by a slight argon flow into a suspension of [Ni$_2$(I^{i}Pr$_2$)$_4$(COD)] (200 mg, 240 µmol) in 10 mL of toluene. After the gas emergence stopped, the resulting reaction mixture was stirred for 1 h at room temperature. All the volatiles were removed *in vacuo* and the remaining solid shown to be a mixture of *trans*-[Ni(I^{i}Pr$_2$)$_2$(PH$_2$)$_2$] **22** and [{Ni(I^{i}Pr$_2$)$_2$}$_2$($\mu_2,\eta^{2:2}$-P$_2$)] (ratio 1:0.35) by ^{1}H NMR spectroscopy. Crystals of **22** suitable for X-ray diffraction were obtained upon storing a saturated THF solution of this mixture at -30 °C for several days. ^{1}H NMR (500 MHz, [D$_8$]toluene, 296 K): δ = 6.35 (s, 4H, NC*H*=CHN), 5.92 (sept, ^3J(H,H) = 7 Hz, 4H, C*H*Me$_2$), 1.38 (d, ^3J(H,H) = 7 Hz, 24H, CH*Me*$_2$), 0.90 (AA′A″A‴XX′ signal (Figure S63 for simulation), 4H, P*H*$_2$) ppm; ^{31}P NMR (202 MHz, [D$_8$]toluene, 296 K): δ = -156.3 (tt$_{vt}$, ^1J(P,H) = 171 Hz, ^3J(P,H) =1 Hz, $^2J_{PP}$ = 30 Hz) ppm; $^{13}C\{^1H\}$ NMR (126 MHz, [D$_8$]toluene, 296 K): δ = 190.0 (t, ^2J(C,P) = 11 Hz, N*C*N), 116.3 (s, N*C*H=CHN), 51.9 (s, *C*HMe$_2$), 23.2 (s, CH*Me*$_2$) ppm.

X-ray crystallography. Data for **2, 4, 6** and **7** were collected on an Agilent Xcalibur instrument while those for **3**, **16**, **17**, **18**, **19, 21** and **22** were obtained using Bruker D8 Apex-1 or Bruker X8 Apex-2 diffractometers, all employing Mo- Kα radiation. An Agilent SuperNova machine, equipped with a Cu-Kα source, was used to analyse **11** and [Ni(IEt$_2$Me$_2$)]$_2$(µ-PPh$_2$)$_2$ (Supporting Information only). All structures were solved using either SHELXS[7] or the olex2.solve[8] structure solution programme followed by refinement using SHELXL[7] via the

Olex2[9] or SHELXTL[7] interfaces. Refinements were largely straightforward, and only points of particular merit will be noted hereafter. The hydride ligands in **2** and **3** and the phosphorus bound hydrogen atoms in **11**, **16**, **17**, **21** and **22** were readily located and refined without restraints. In **6**, **7**, **11**, **17**, **19**, **22** and $[Ni(IEt_2Me_2)]_2(\mu\text{-}PPh_2)_2$ (Supporting Information only), the asymmetric unit each contained half of one molecule of the nickel containing complex. Molecular entities were completed in **6**, **7**, **11**, **17** and **22** courtesy of crystallographic inversion centres, coincident with the transition metal in each case. In **19**, a 2-fold rotation axis (implicit to the space group symmetry) served to generate one full molecule of this compound. Two crystal structures, **19** and **21**, were seen to contain solvent in the motif with the asymmetric unit in the former hosting half of a benzene molecule and the latter housing a full benzene moiety that was treated for disorder between two overlaid positions in a 60:40 split. 60:40 disorder was also modelled for one of the isopropyl groups in **16**.

Crystallographic data for all compounds have been deposited with the Cambridge Crystallographic Data Centre as supplementary publications CCDC 2009924-2009936 for **2**-**4**, **6**-**7**, **11**, 1**6**-**19** and **21**-**22** and $[Ni(IEt_2Me_2)]_2(\mu\text{-}PPh_2)_2$ (Supporting Information only). Copies of these data can be obtained free of charge on application to CCDC, 12 Union Road, Cambridge CB2 1EZ, UK [fax(+44) 1223 336033, e-mail: deposit@ccdc.cam.ac.uk.

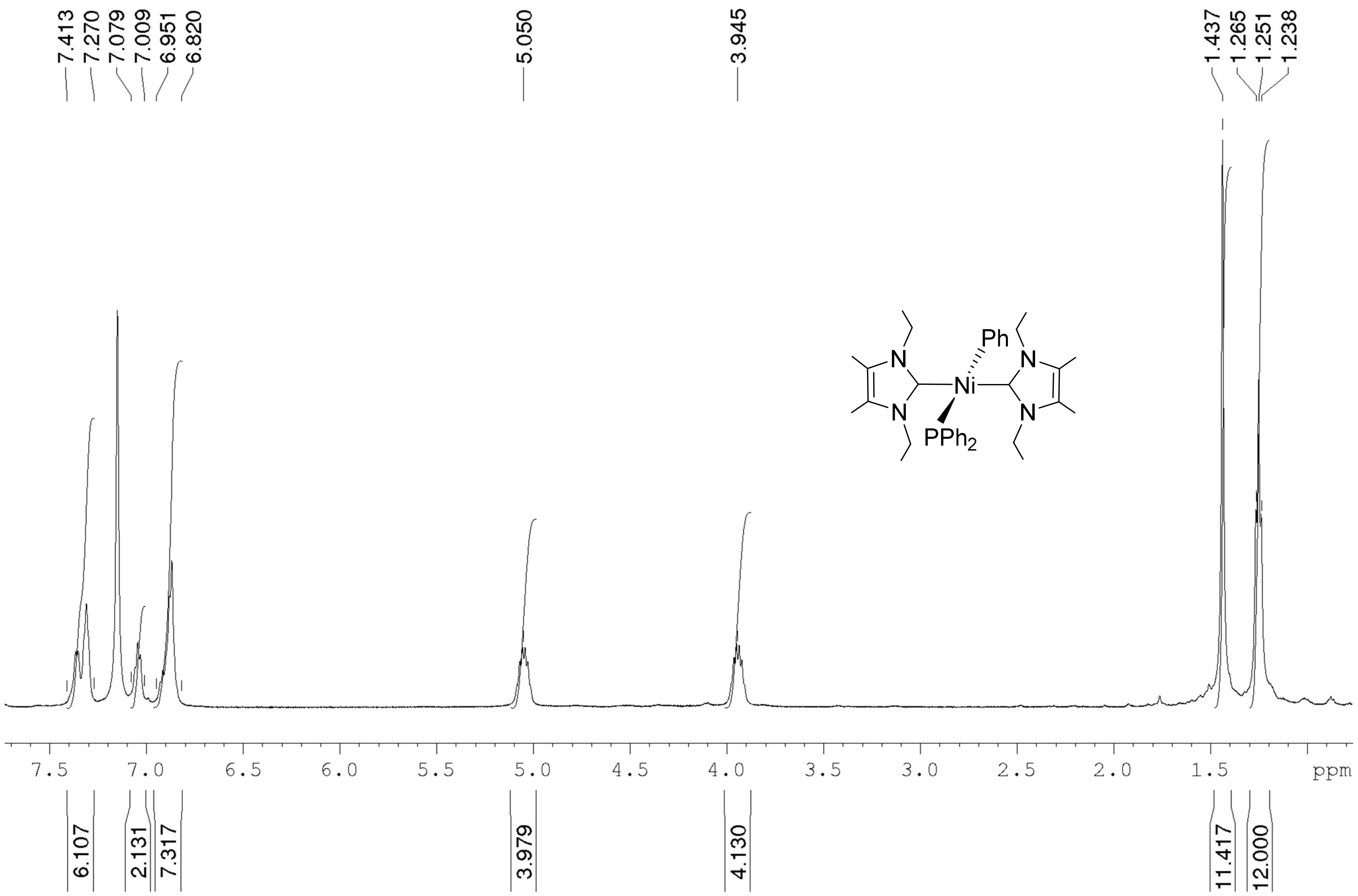

Figure S1. ^{1}H NMR spectrum (500 MHz, [D_6]benzene, 298 K) of *trans*-[Ni(IEt_2Me_2)$_2$(PPh_2)Ph] (**2**).

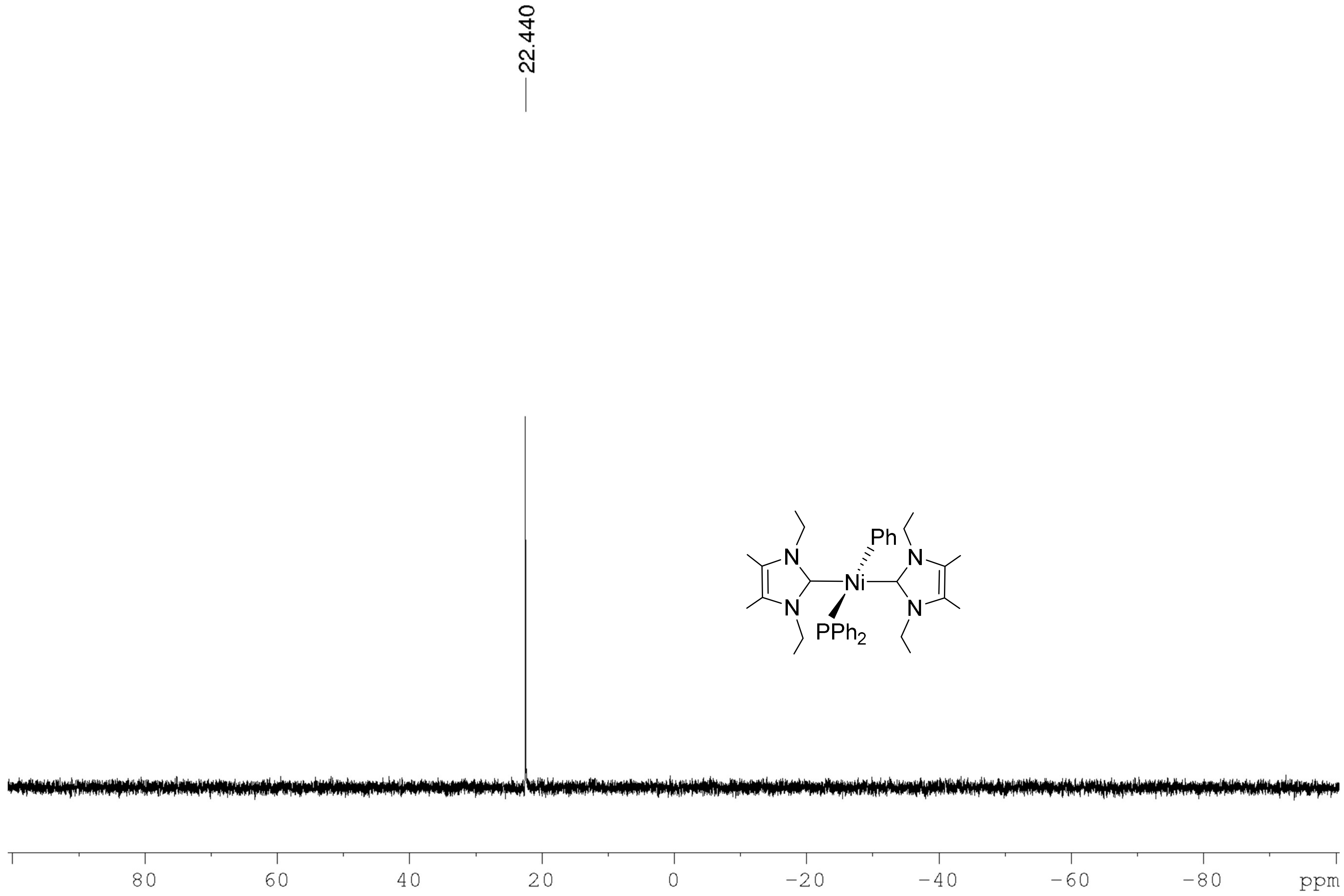

Figure S2. $^{31}P\{^{1}H\}$ NMR spectrum (202 MHz, [D$_6$]benzene, 298 K) of *trans*-[Ni(IEt$_2$Me$_2$)$_2$(PPh$_2$)Ph] (**2**).

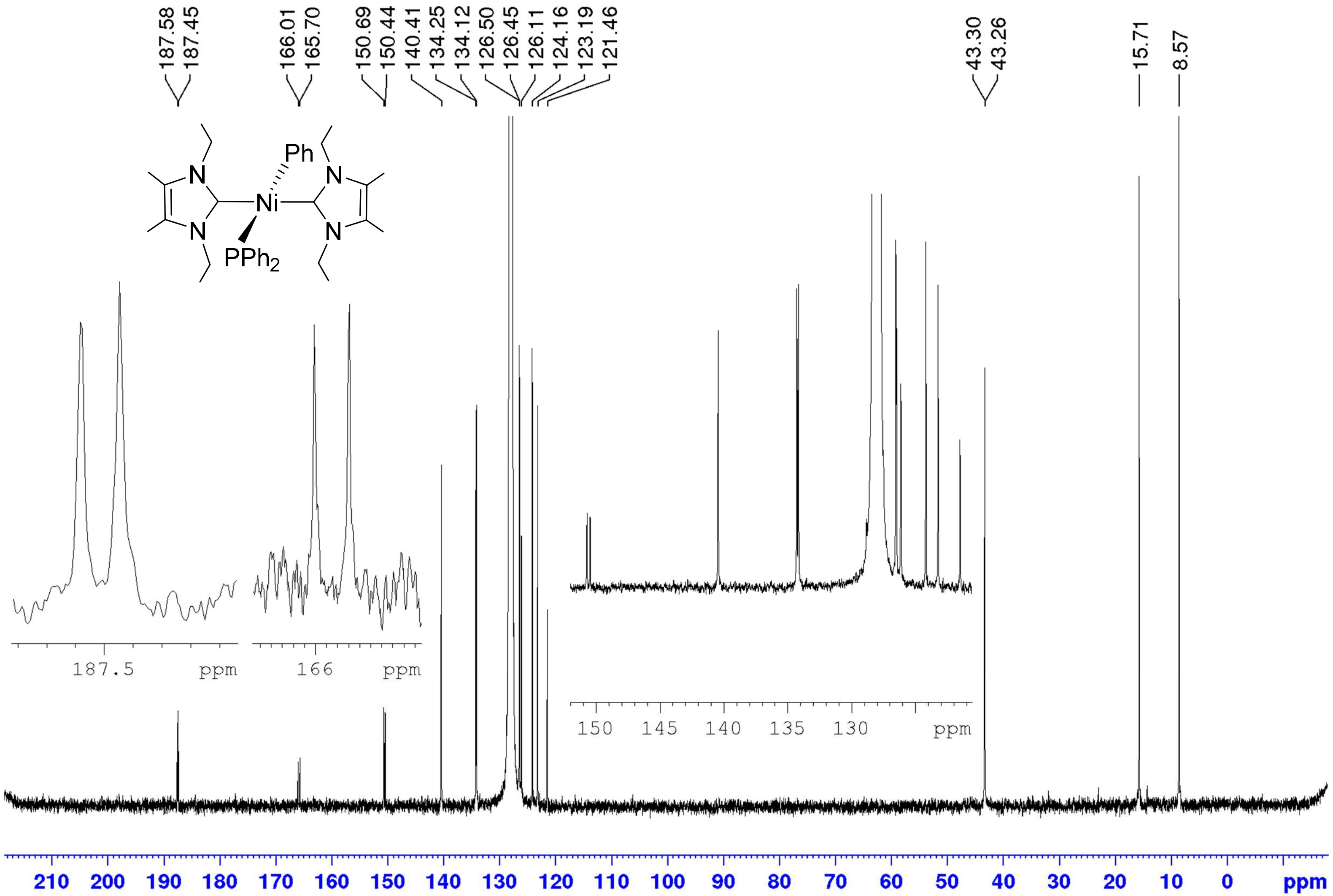

Figure S3. $^{13}C\{^1H\}$ NMR spectrum (126 MHz, [D$_6$]benzene, 298 K) of *trans*-[Ni(IEt$_2$Me$_2$)$_2$(PPh$_2$)Ph] (**2**). Insets highlight higher frequency signals.

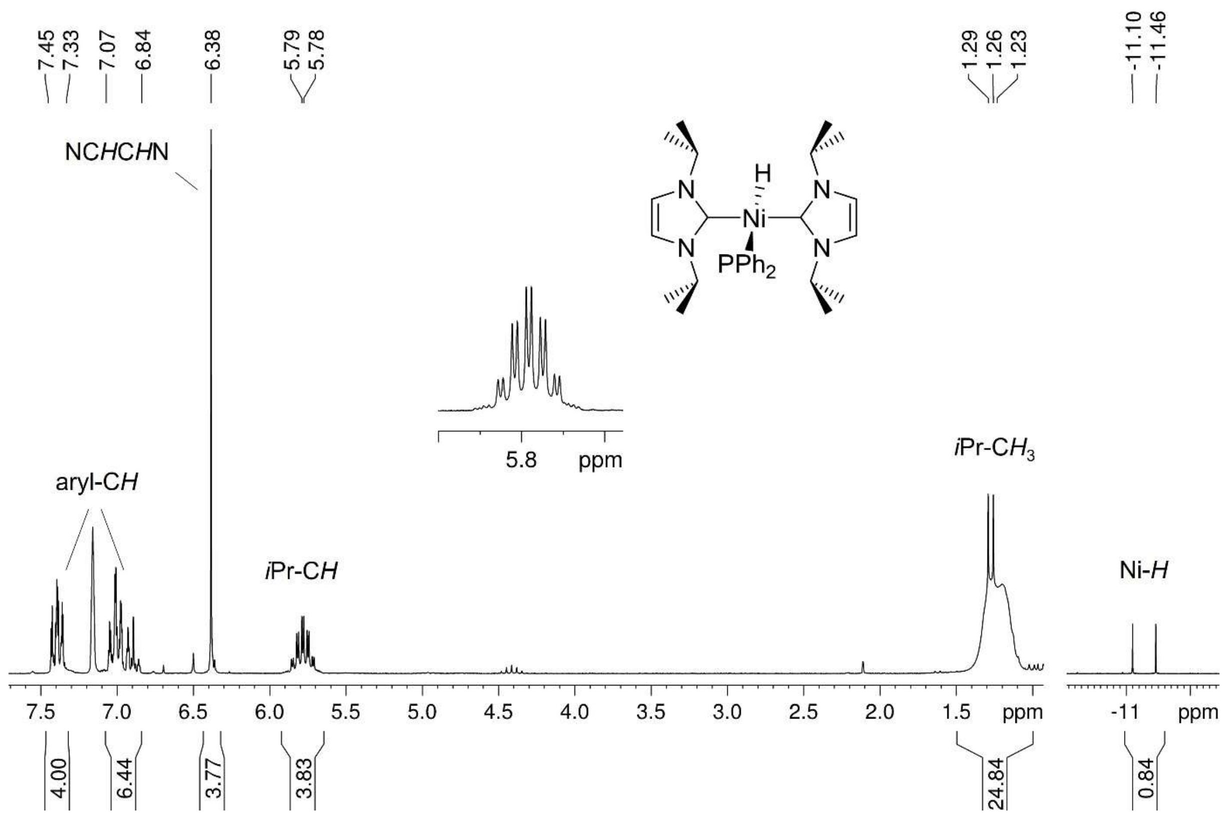

Figure S4. ^{1}H NMR spectrum (200 MHz, [D$_6$]benzene, 296 K) of *trans*-[Ni(I^{i}Pr$_2$)$_2$(PPh$_2$)H] (**3**).

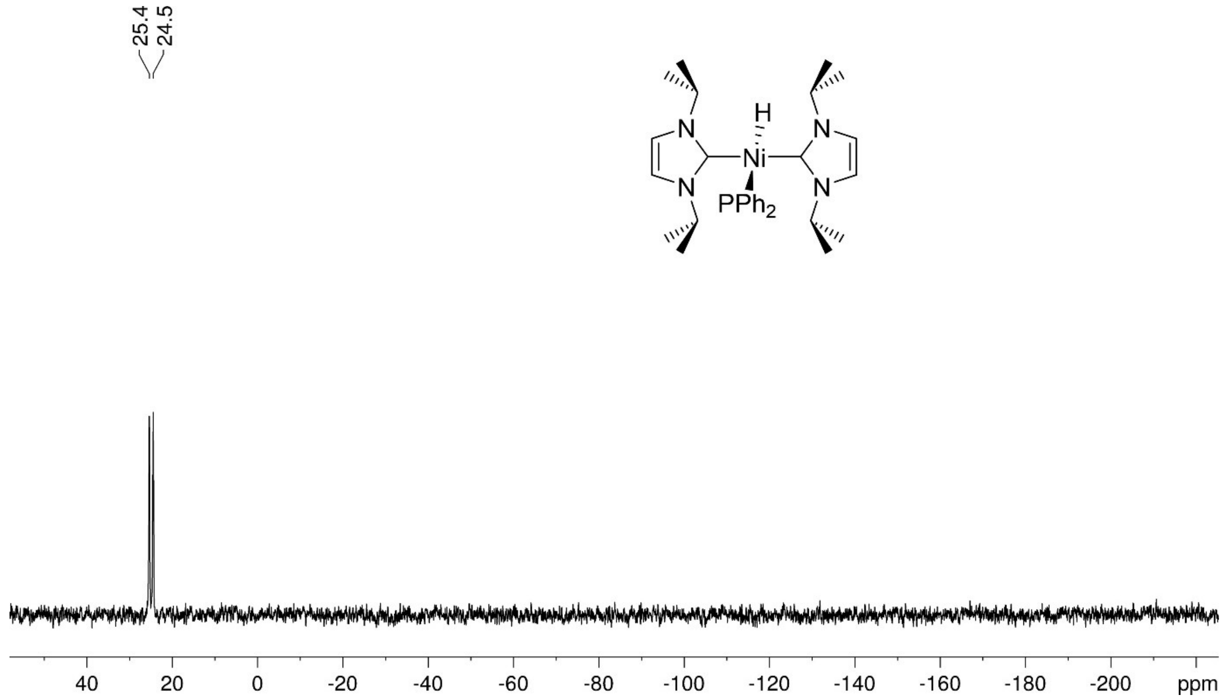

Figure S5. ^{31}P NMR spectrum (82 MHz, [D$_6$]benzene, 298 K) of *trans*-[Ni(I^{i}Pr$_2$)$_2$(PPh$_2$)H] (**3**).

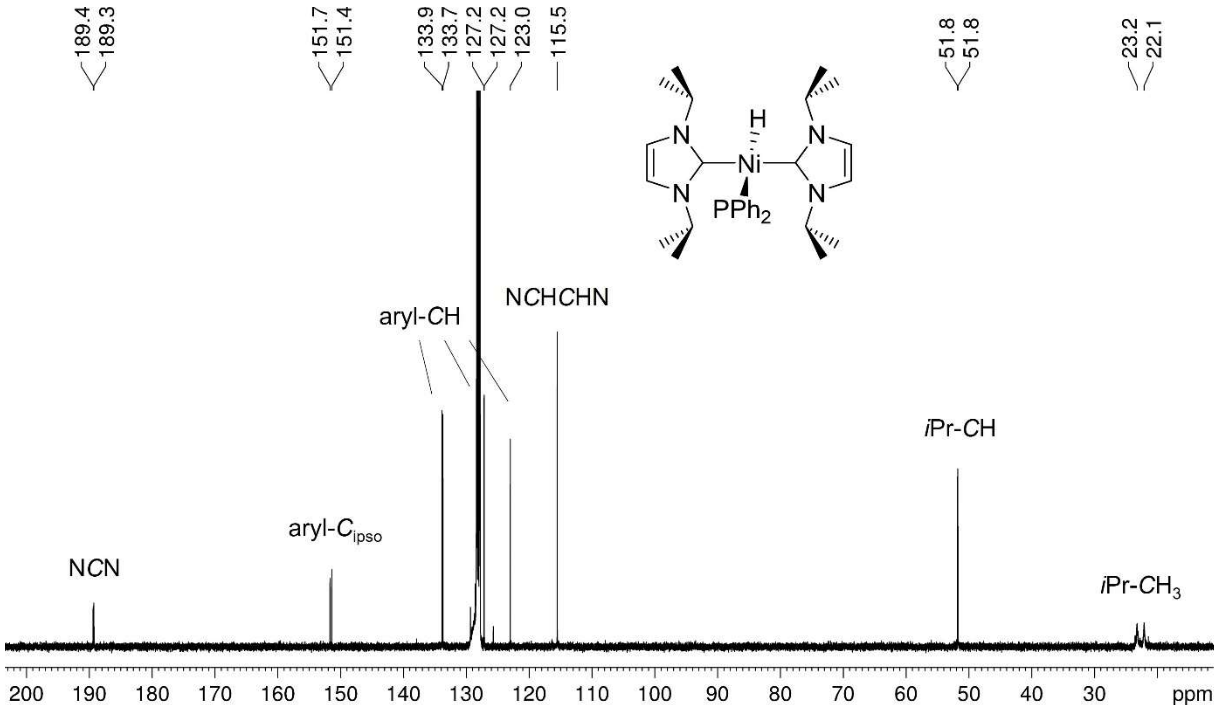

Figure S6. $^{13}C\{^1H\}$ NMR spectrum (50 MHz, [D6]benzene, 296 K) of *trans*-[Ni(I^{i}Pr$_2$)$_2$(PPh$_2$)H] (**3**)

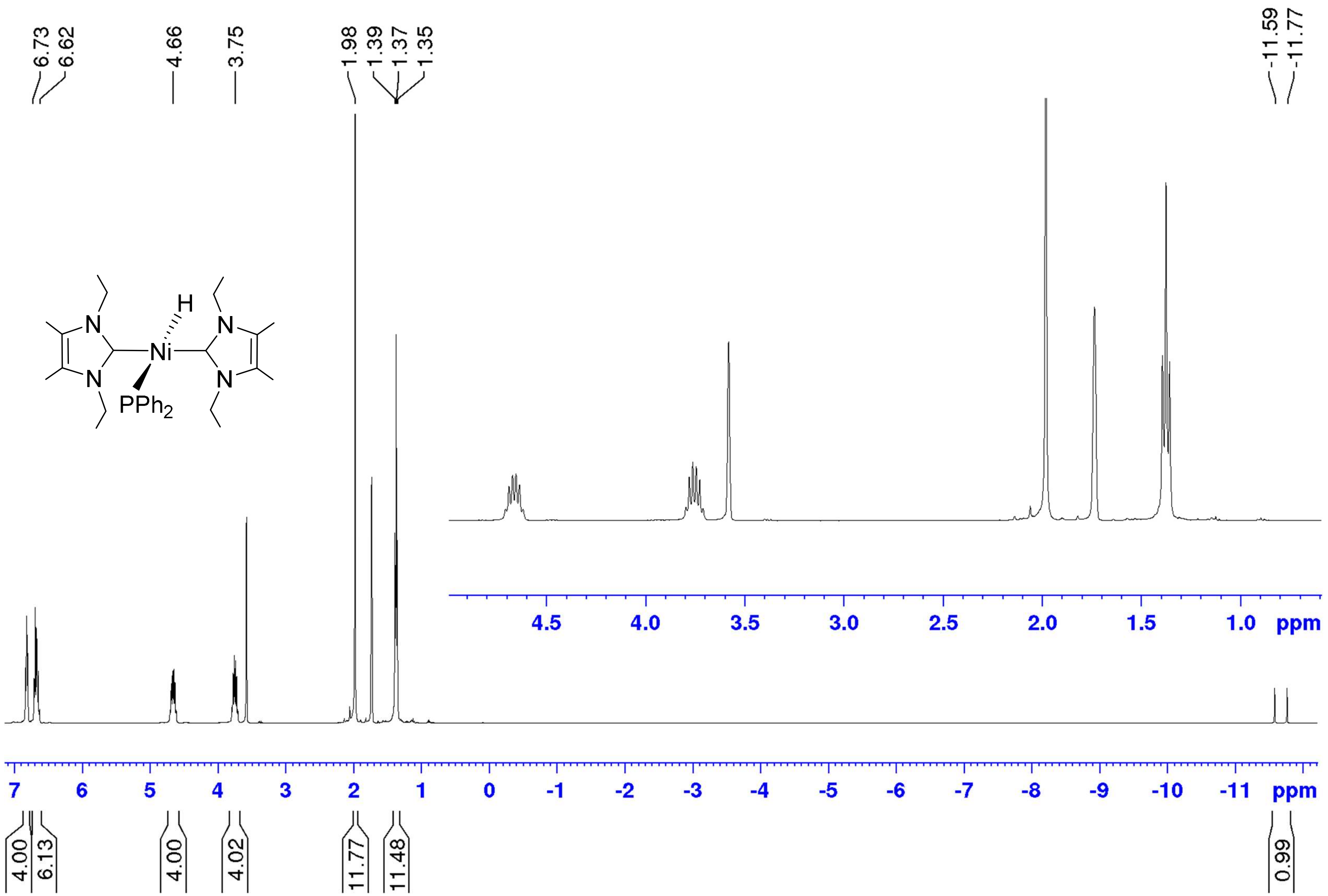

Figure S7. ^{1}H NMR spectrum (400 MHz, [D$_8$]THF, 235 K) of *trans*-[Ni(IEt$_2$Me$_2$)$_2$(PPh$_2$)H] (**4**). Inset emphasises the multiplicity of medium frequency signals.

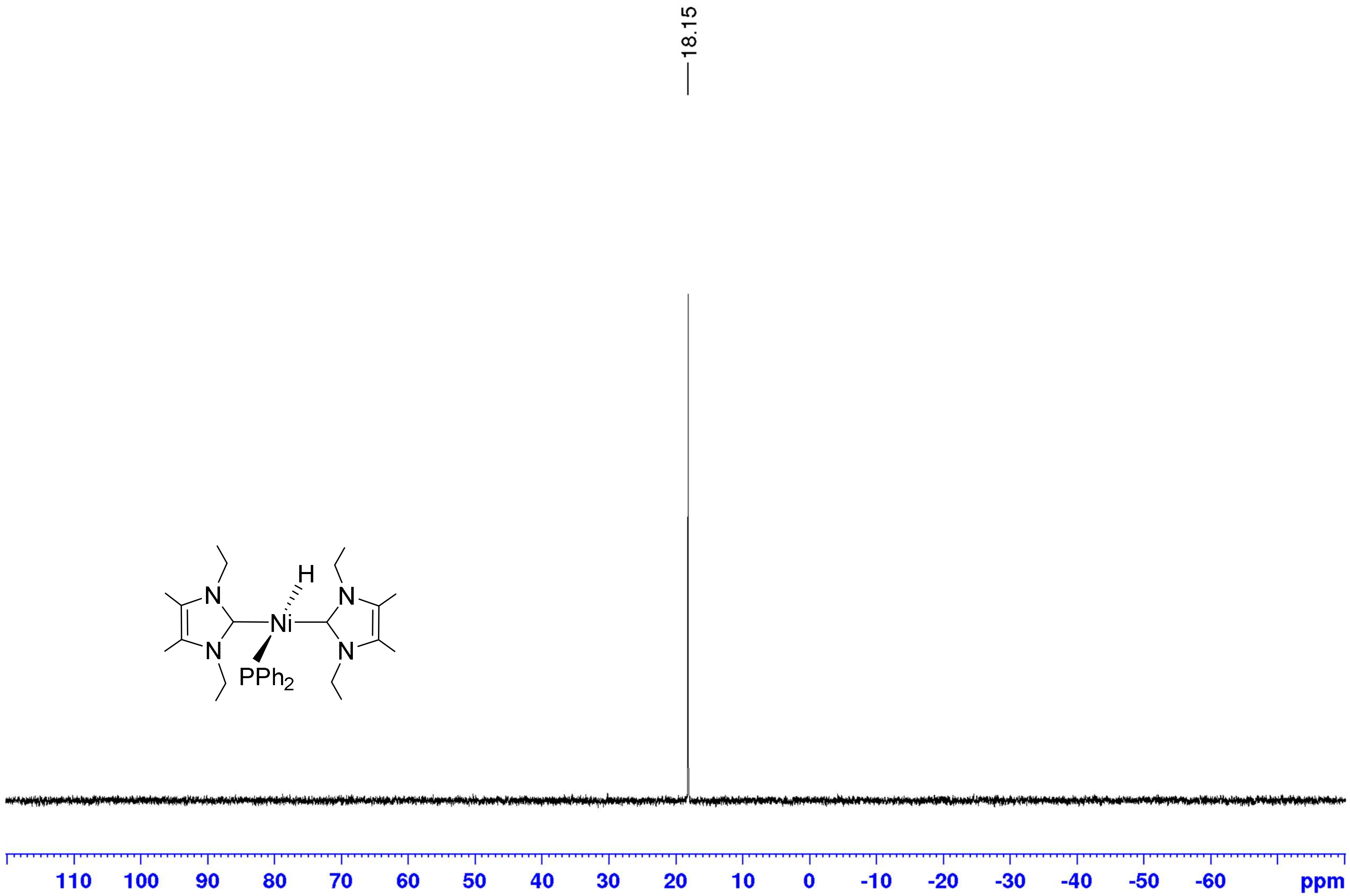

Figure S8. ^{31}P{^{1}H} NMR spectrum (162 MHz, [D$_8$]THF, 235 K) of *trans*-[Ni(IEt$_2$Me$_2$)$_2$(PPh$_2$)H] (**4**).

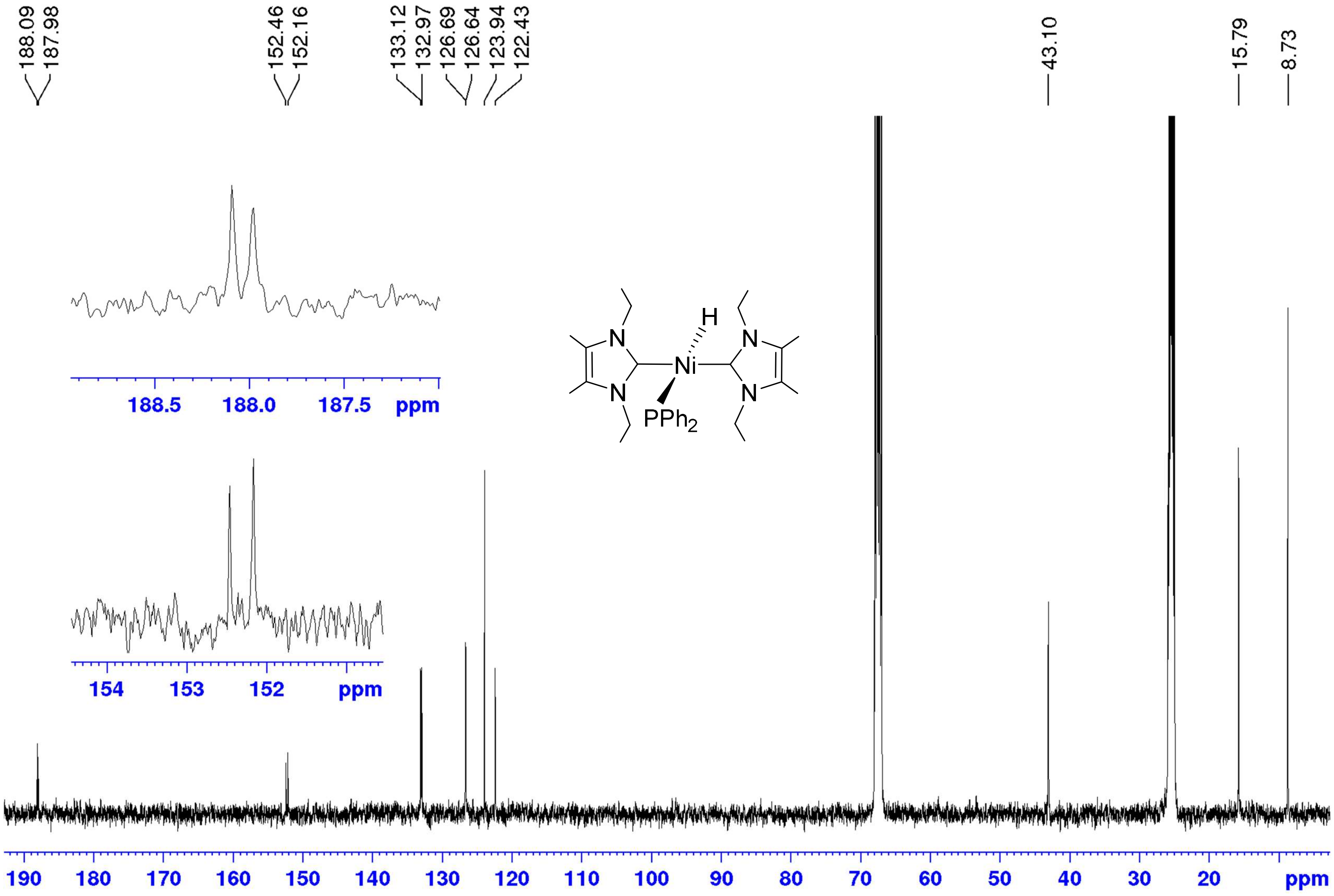

Figure S9. $^{13}C\{^1H\}$ NMR spectrum (101 MHz, [D$_8$]THF, 235 K) of *trans*-[Ni(IEt$_2$Me$_2$)$_2$(PPh$_2$)H] (**4**). Insets highlight NHC and *ipso*-*C*-PPh$_2$ signals.

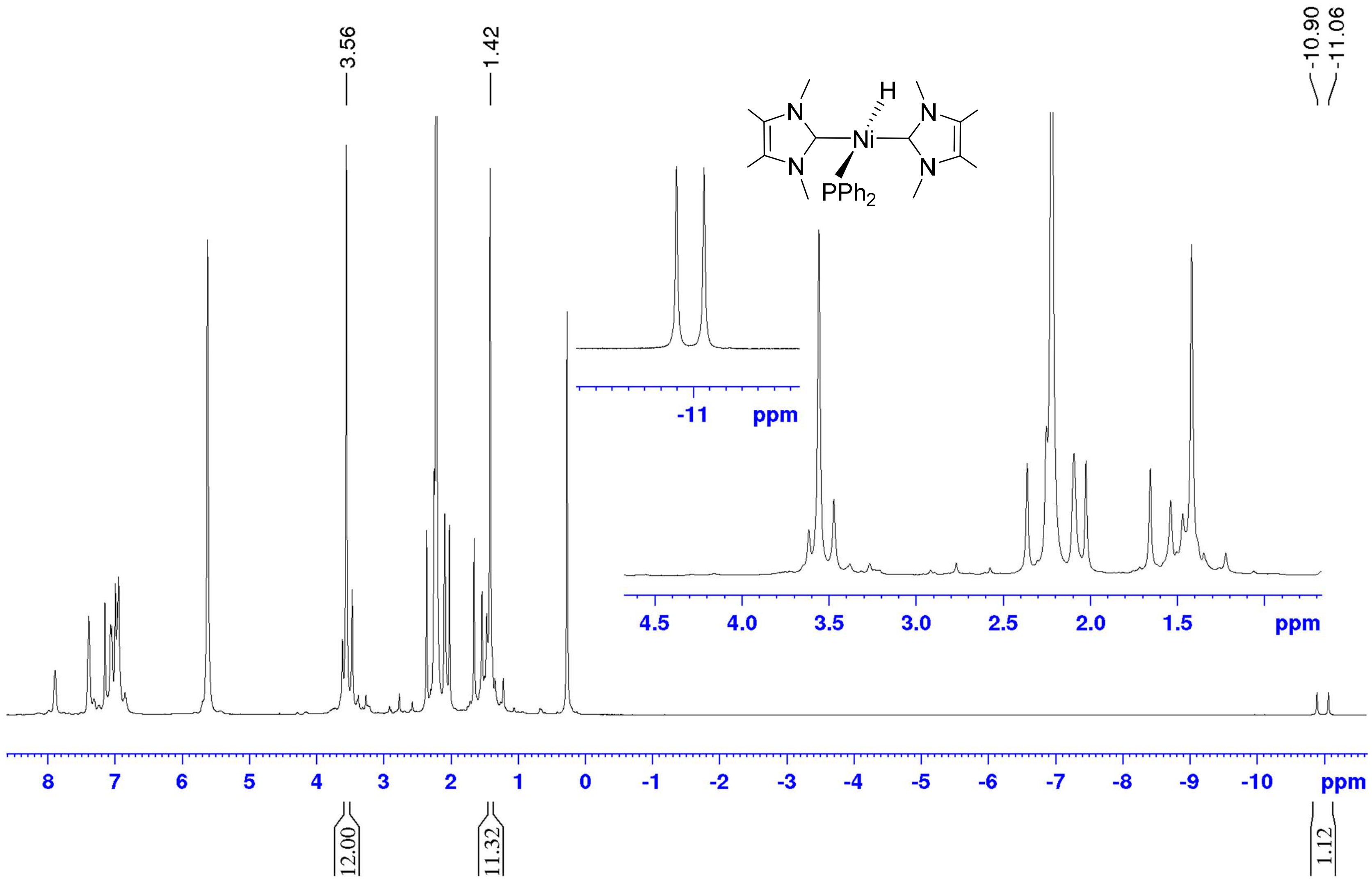

Figure S10. ^{1}H NMR spectrum (400 MHz, [D_8]toluene, 211 K) of the reaction of [Ni(COD)$_2$], IMe$_4$ and PPh$_2$ (1:2:1 ratio) inserted into a pre-cooled NMR spectrometer at 211 K. Insets highlight signals assigned to *trans*-[Ni(IMe$_4$)$_2$(PPh$_2$)H] (**5**).

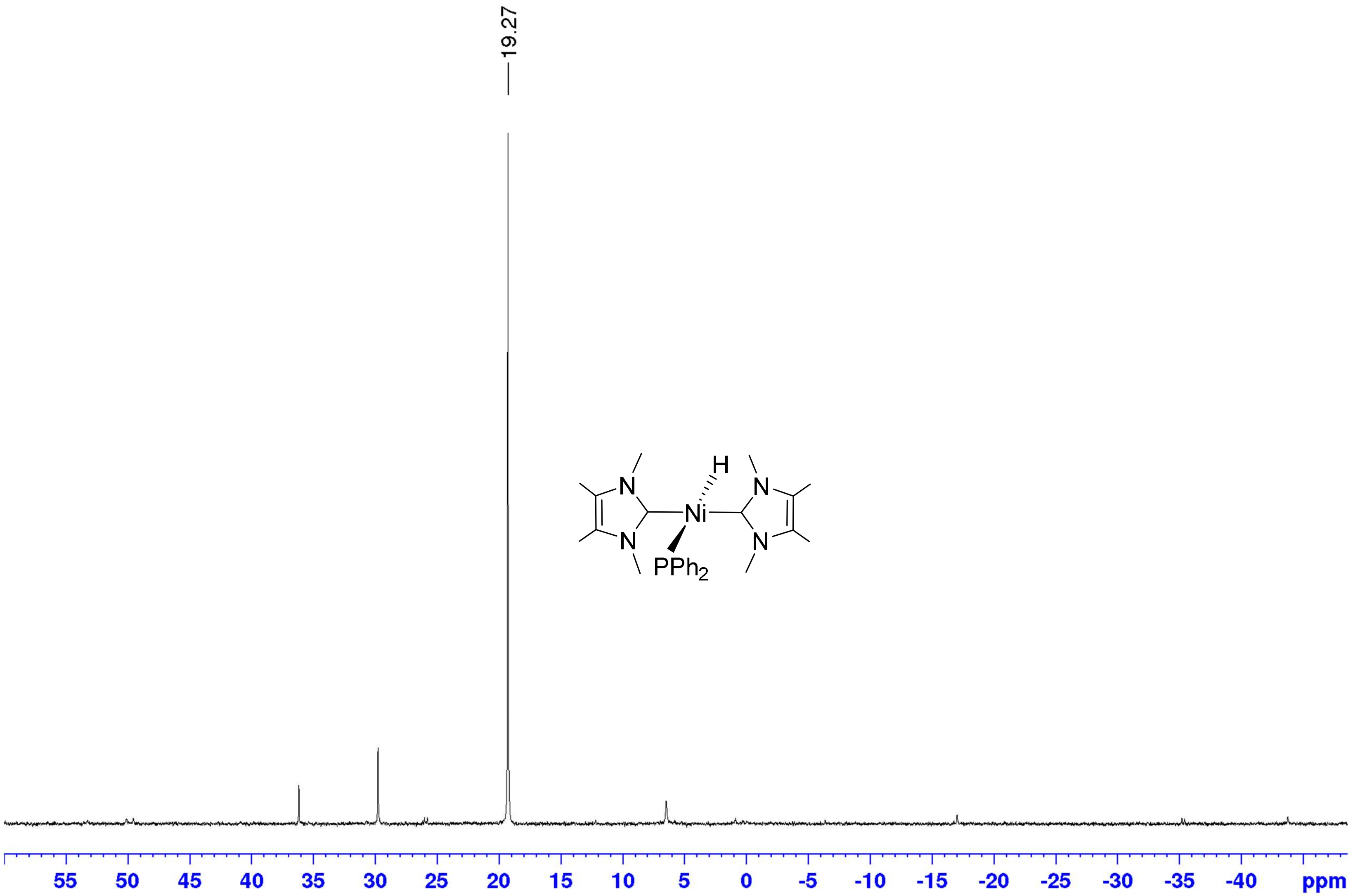

Figure S11. $^{31}P\{^1H\}$ NMR spectrum (400 MHz, [D_8]toluene, 211 K) of the reaction of [Ni(COD)$_2$], IMe$_4$ and PPh$_2$ (1:2:1 ratio) inserted into a pre-cooled NMR spectrometer at 211 K. Major signal assigned as *trans*-[Ni(IMe$_4$)$_2$(PPh$_2$)H] (**5**).

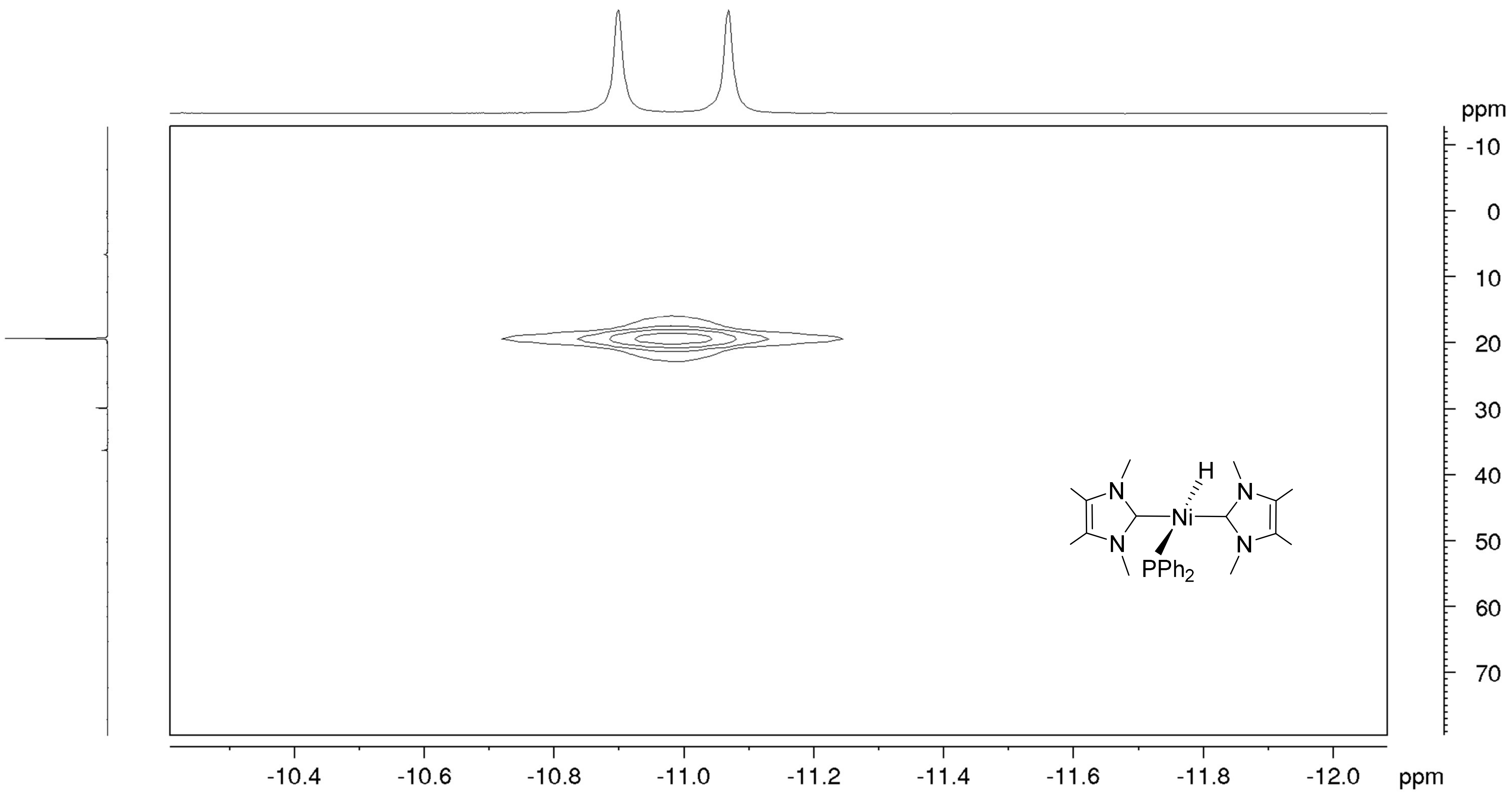

Figure S12. $^{31}P\{^{1}H\}$-^{1}H HMQC spectrum ([D_8]toluene, 211 K) of *trans*-[$Ni(IMe_4)_2(PPh_2)H$] (**5**).

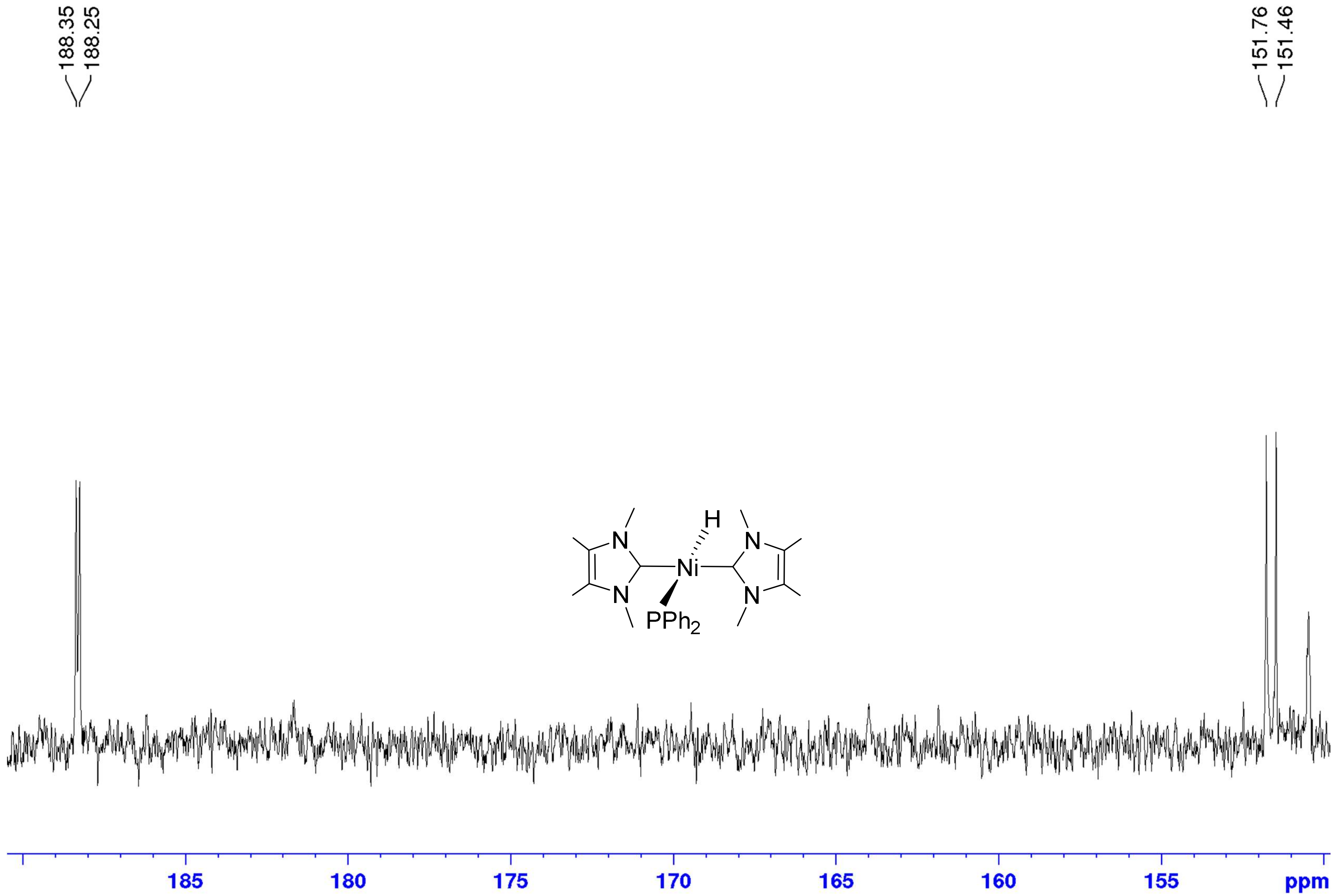

Figure S13. High frequency region of the $^{13}C\{^{1}H\}$ PENDANT NMR spectrum (101 MHz, [D_8]toluene, 211 K) illustrating the NHC and *ipso*-*C*-PPh_2 resonances of *trans*-[Ni(IMe_4)$_2$(PPh_2)H] (**5**).

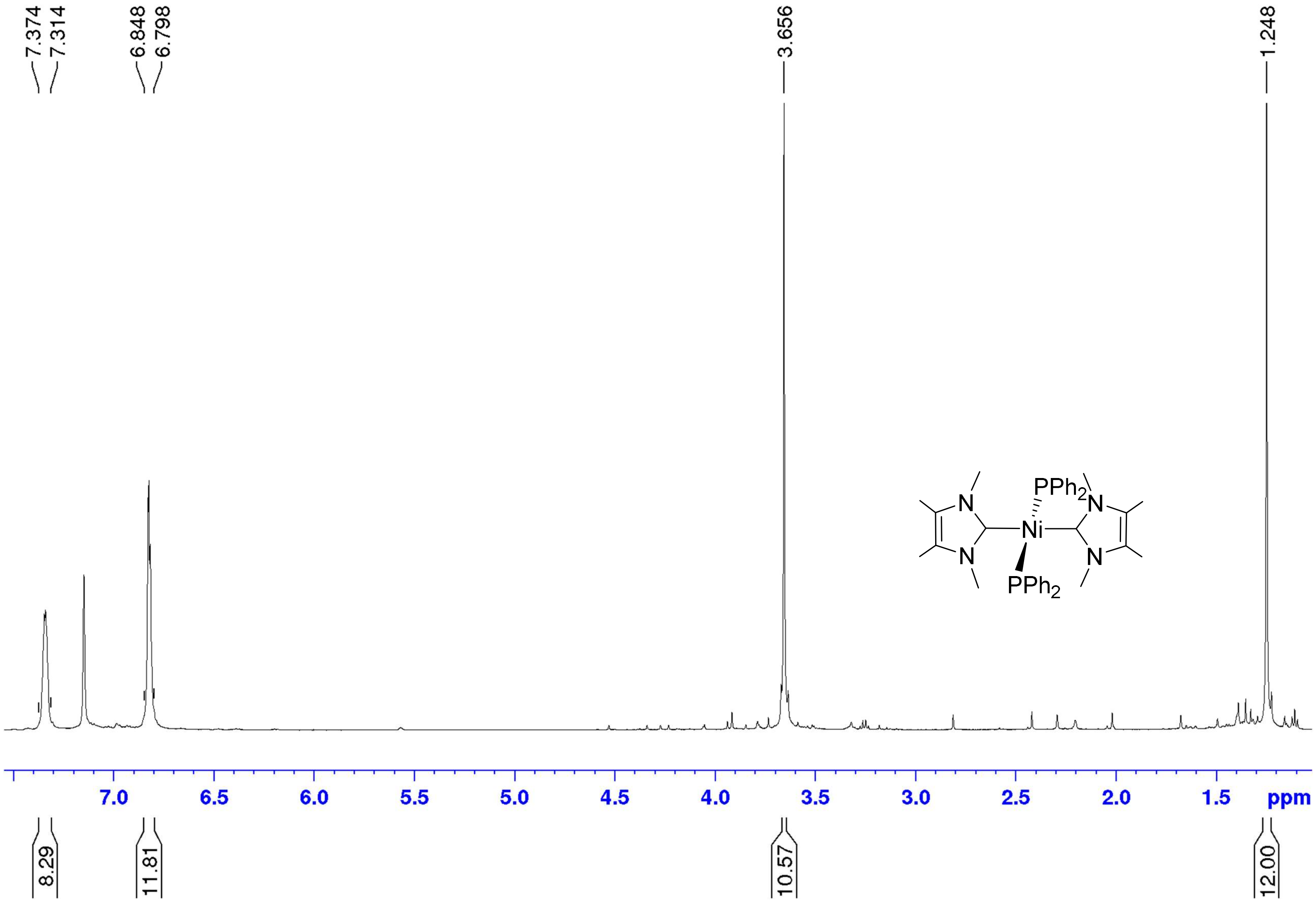

Figure S14. ^{1}H NMR spectrum (500 MHz, [D_6]benzene, 298 K) of *trans*-[Ni(IMe_4)$_2$(PPh_2)$_2$] (**6**).

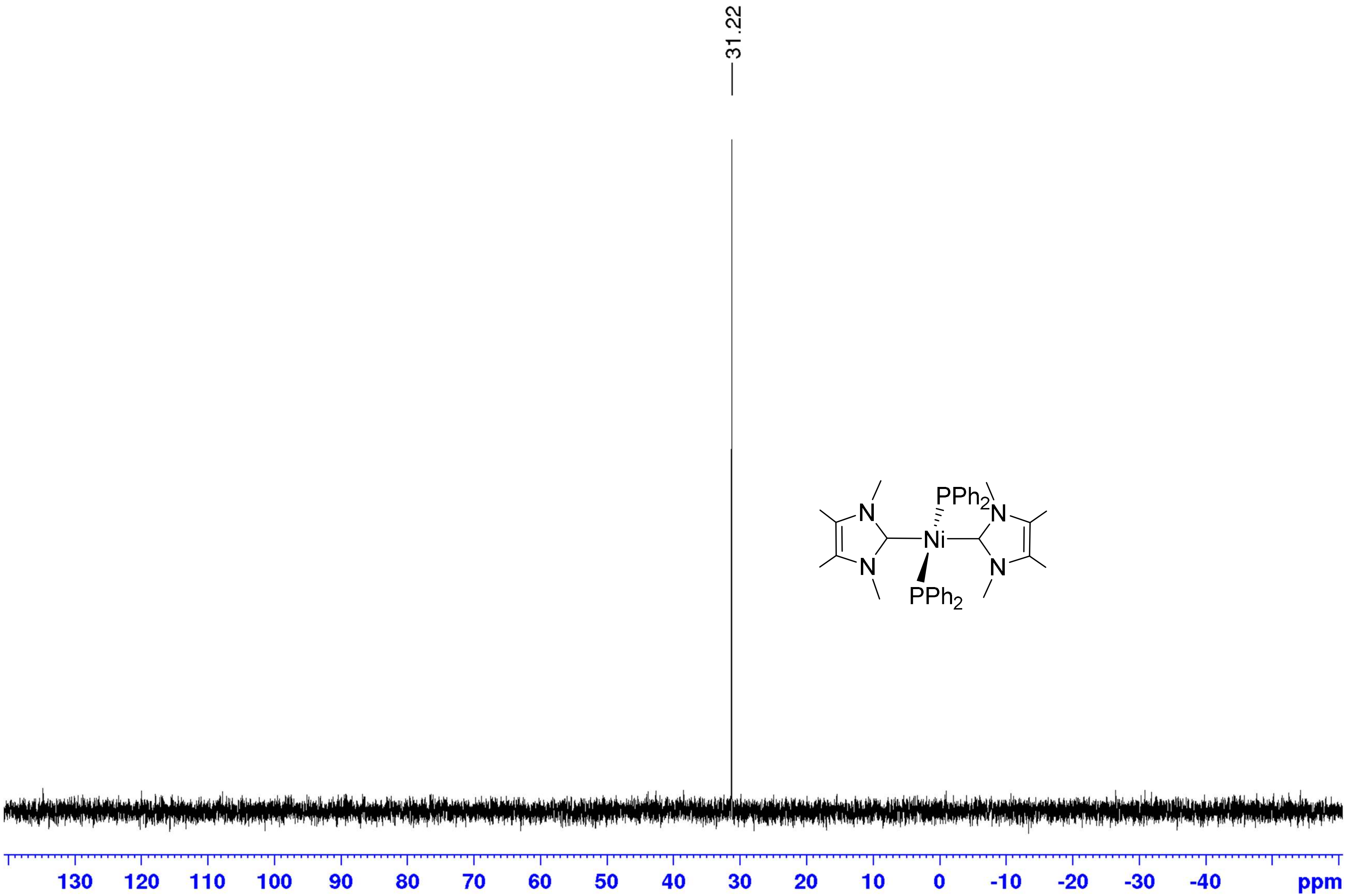

Figure S15. $^{31}P\{^1H\}$ NMR spectrum (202 MHz, [D$_6$]benzene, 298 K) of *trans*-[Ni(IMe$_4$)$_2$(PPh$_2$)$_2$] (**6**).

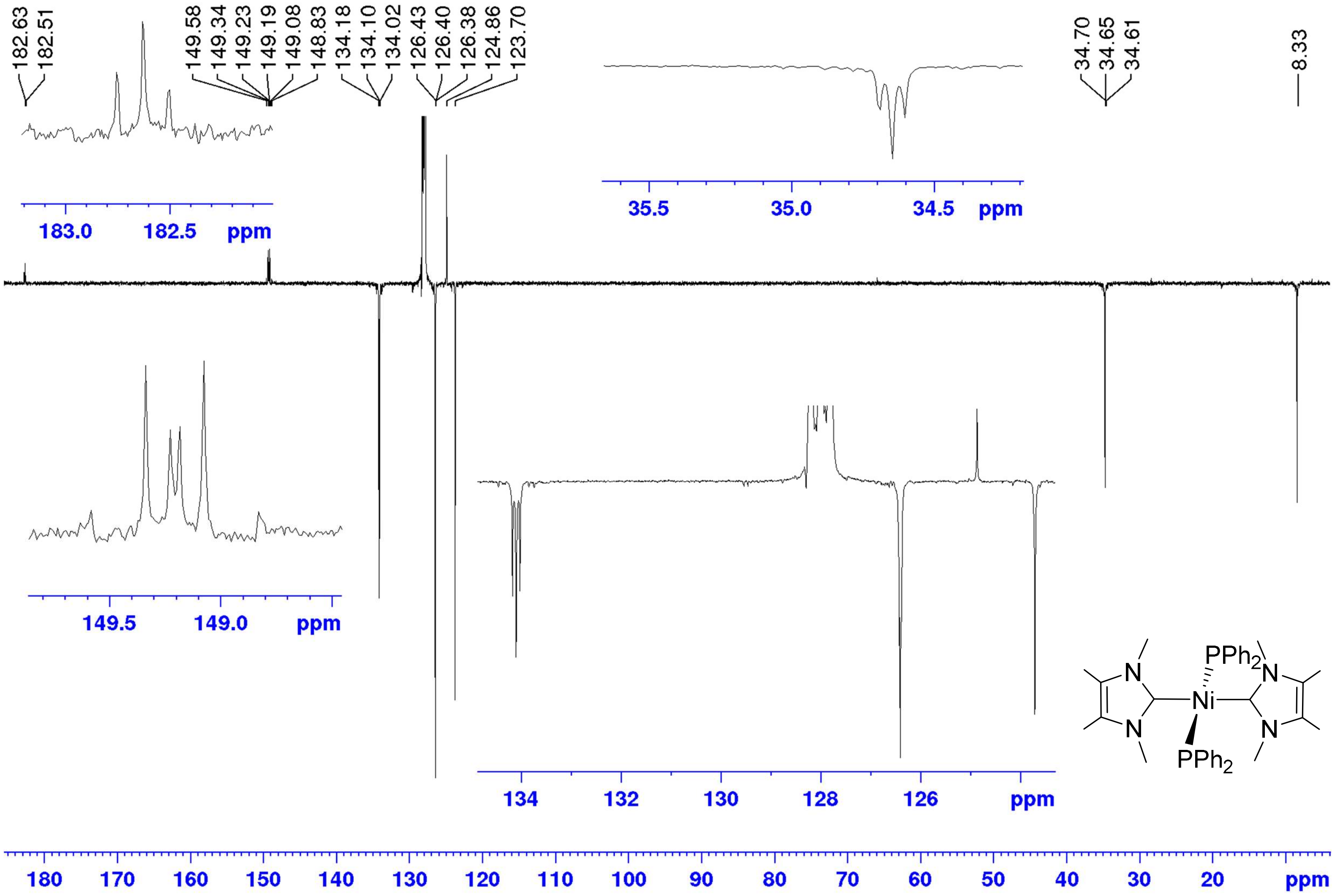

Figure S16. $^{13}C\{^{1}H\}$ PENDANT NMR spectrum (126 MHz, [D$_6$]benzene, 298 K) of *trans*-[Ni(IMe$_4$)$_2$(PPh$_2$)$_2$] (**6**).

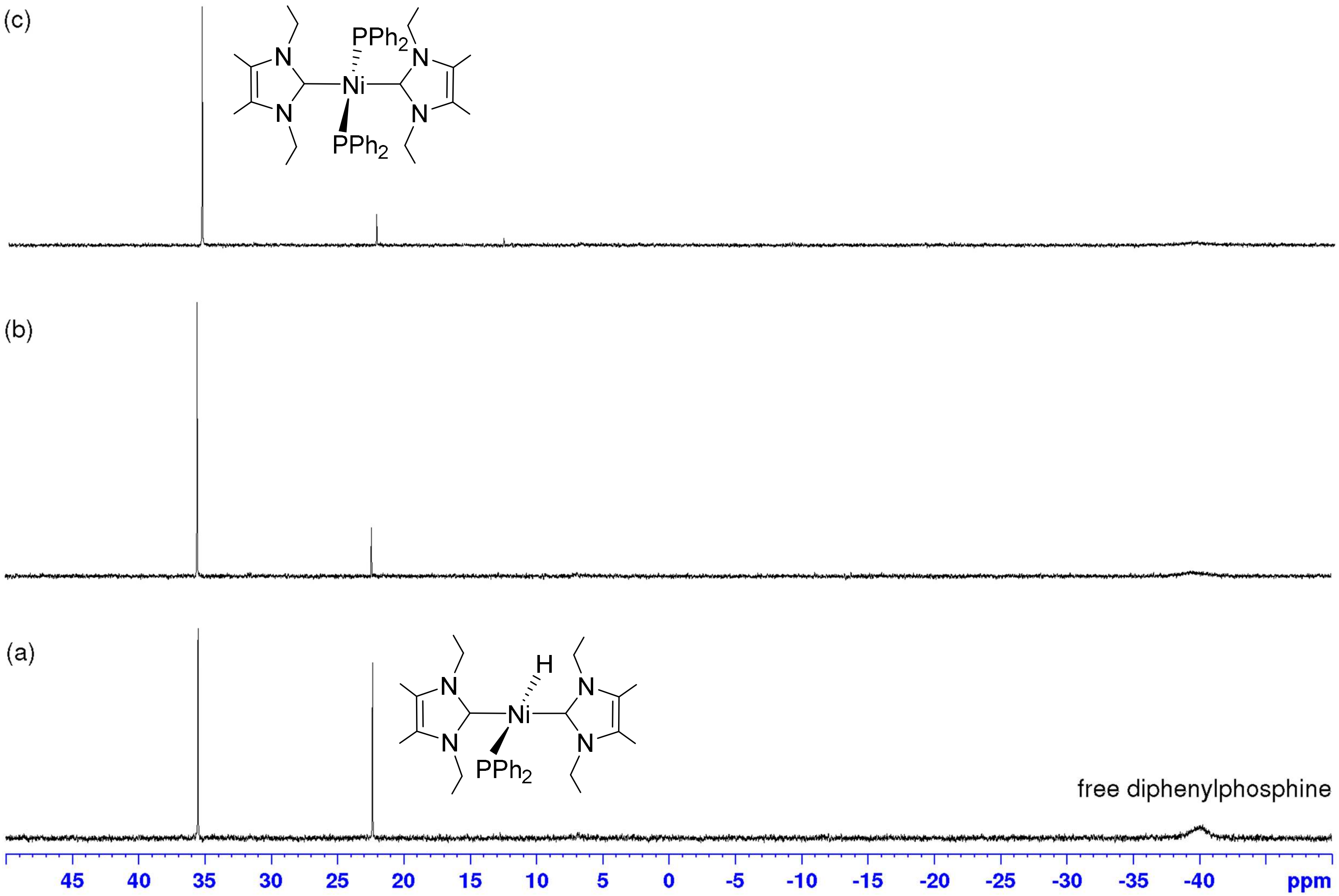

Figure S17. $^{31}P\{^{1}H\}$ NMR spectra (202 MHz, [D$_6$]benzene, 298 K) showing the reaction of PPh_2H with *trans*-[Ni(IEt$_2$Me$_2$)$_2$(PPh$_2$)H] (**4**; δ 22) over (a) 9 days, (b) 22 days and (c) 1 month to form *trans*-[Ni(IEt$_2$Me$_2$)$_2$(PPh$_2$)$_2$] (**7**; δ 35).

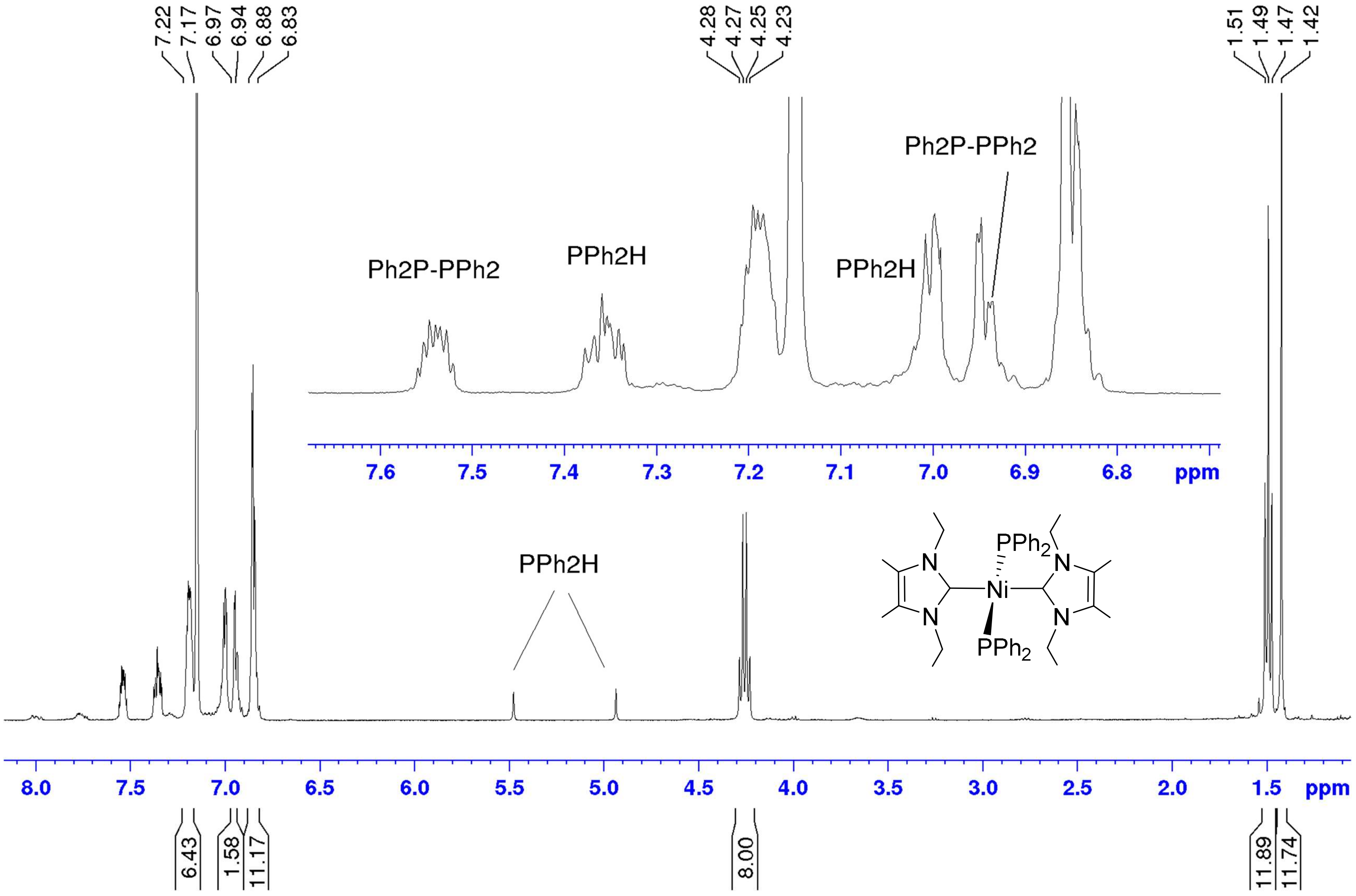

Figure S18. ^{1}H NMR spectrum (400 MHz, [D$_6$]benzene, 298 K) of *trans*-[Ni(IEt$_2$Me$_2$)$_2$(PPh$_2$)$_2$] (**7**) formed by reaction of **4** with Ph_2P-PPh_2.

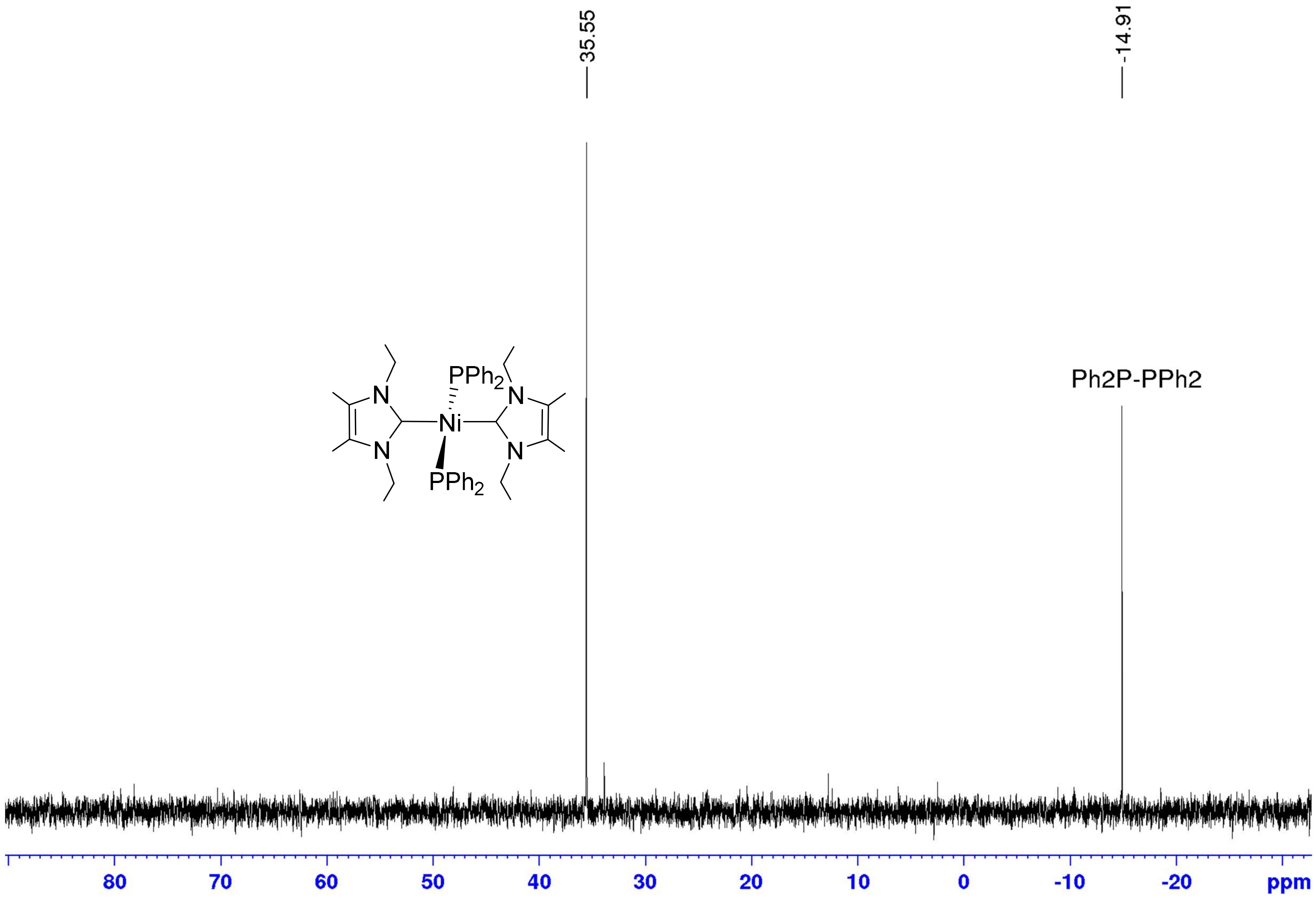

Figure S19. $^{31}P\{^{1}H\}$ NMR spectrum (202 MHz, [D$_6$]benzene, 298 K) of *trans*-[Ni(IEt$_2$Me$_2$)$_2$(PPh$_2$)$_2$] (**7**) formed by reaction of **4** with Ph_2P-PPh_2.

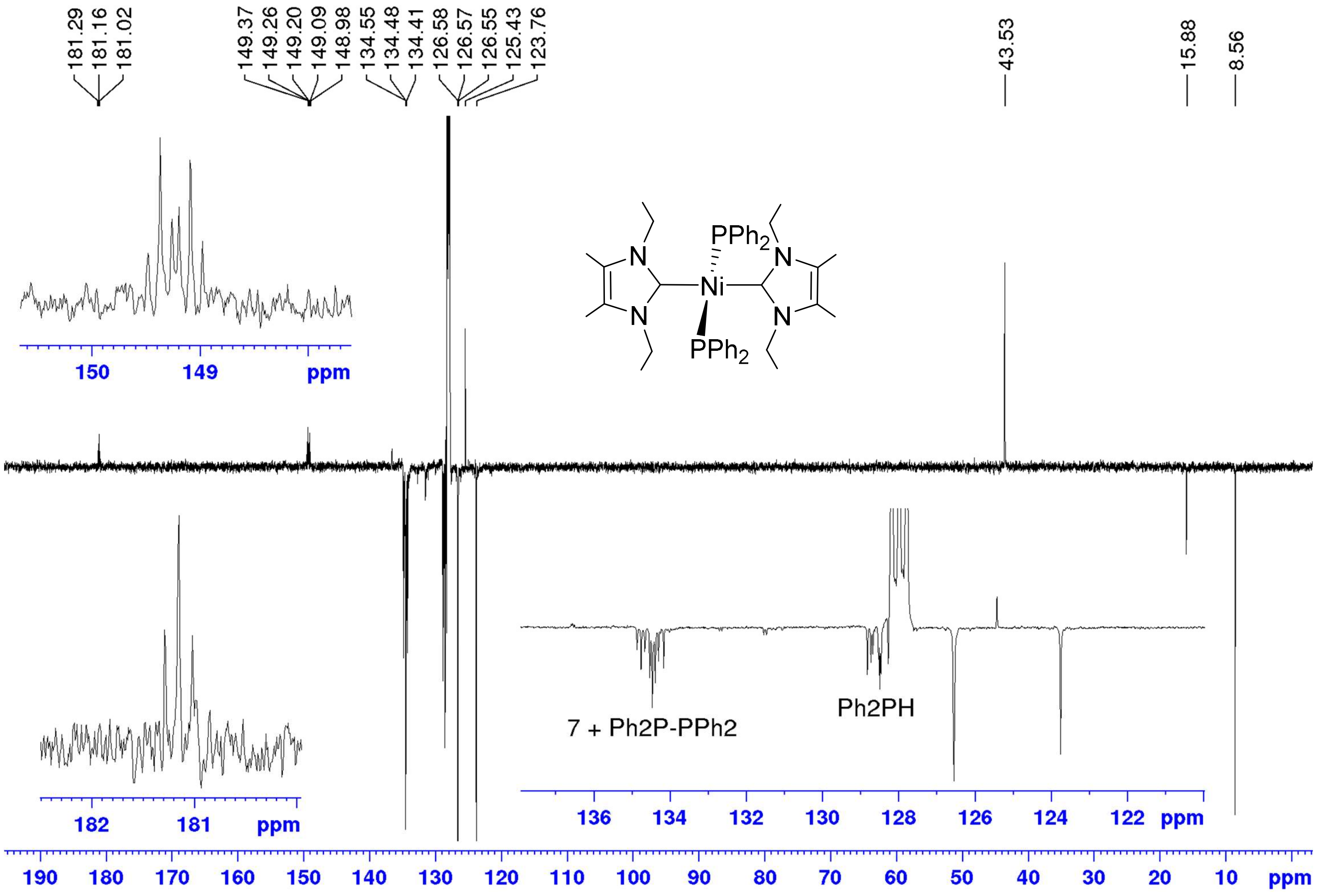

Figure S20. $^{13}C\{^{1}H\}$ NMR spectrum (126 MHz, [D$_6$]benzene, 298 K) of *trans*-[Ni(IEt$_2$Me$_2$)$_2$(PPh$_2$)$_2$] (**7**) formed by reaction of **4** with Ph$_2$P-PPh$_2$.

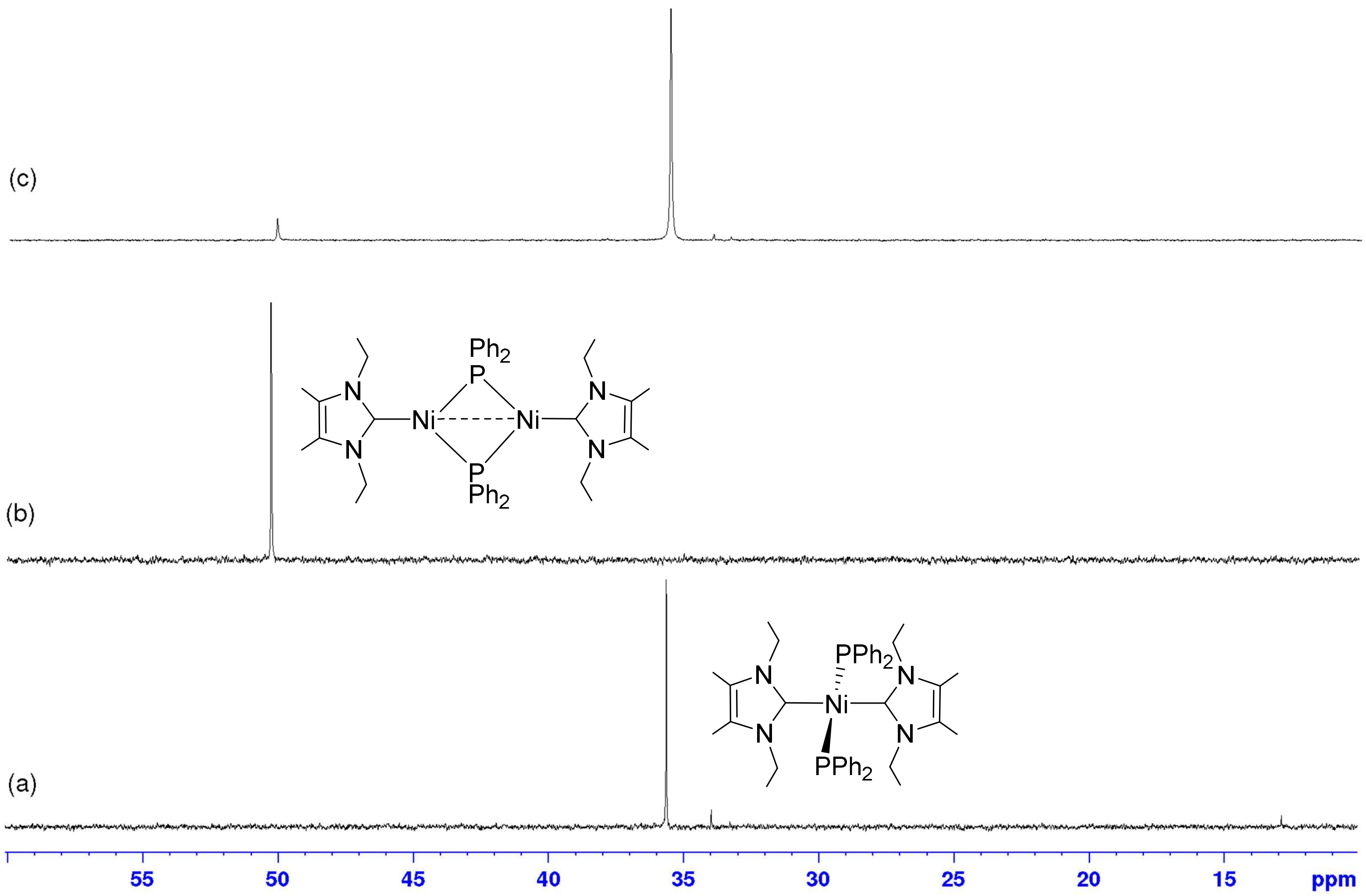

Figure S21. $^{31}P\{^{1}H\}$ NMR spectra (202 MHz, [D$_6$]benzene) of (a) *trans*-[Ni(IEt$_2$Me$_2$)$_2$(PPh$_2$)$_2$] (**7**; from reaction of **4** and Ph$_2$P-PPh$_2$), (b) [Ni(IEt$_2$Me$_2$)]$_2$(μ-PPh$_2$)$_2$ and (c) showing the formation of both species in the reaction of [Ni(COD)$_2$], IEt$_2$Me$_2$ and Ph$_2$P-PPh$_2$.

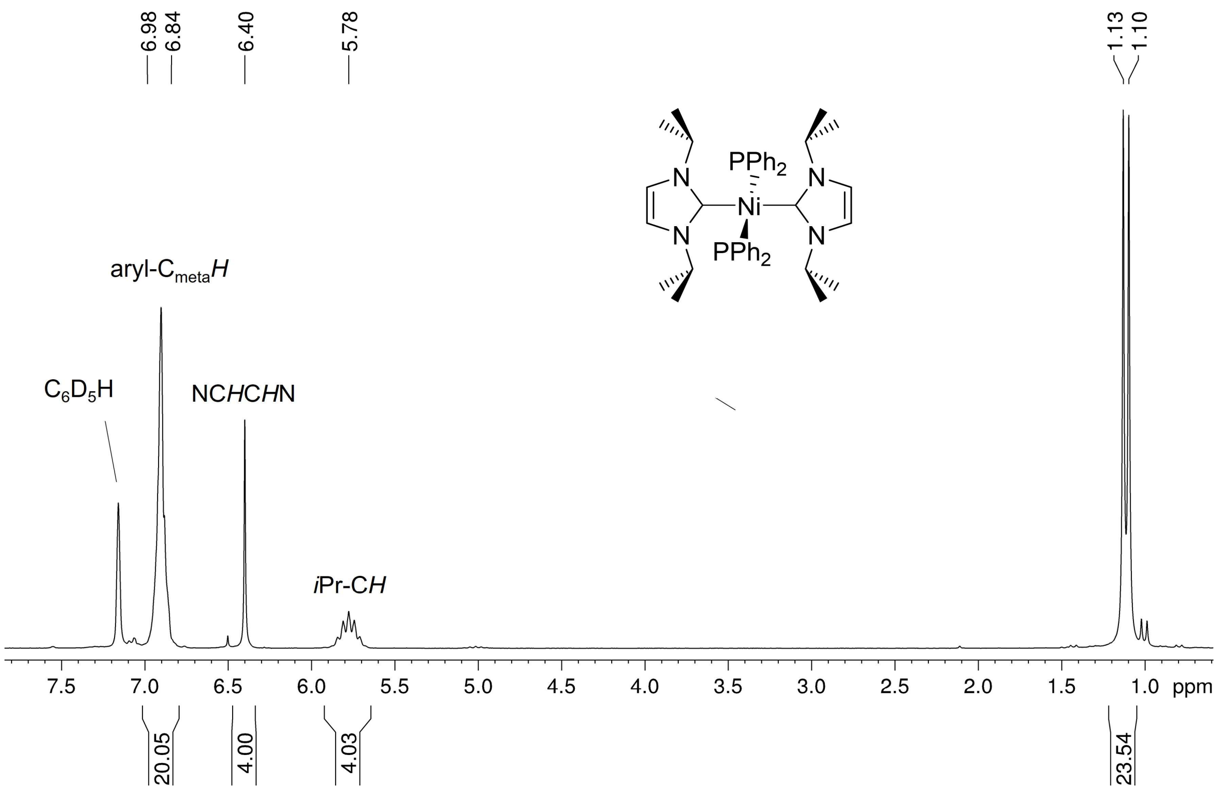

Figure S22. ^{1}H NMR spectrum (200 MHz, [D6]benzene, 298 K) of *trans*-[Ni(I^{i}Pr2)2(PPh2)2] (**8**).

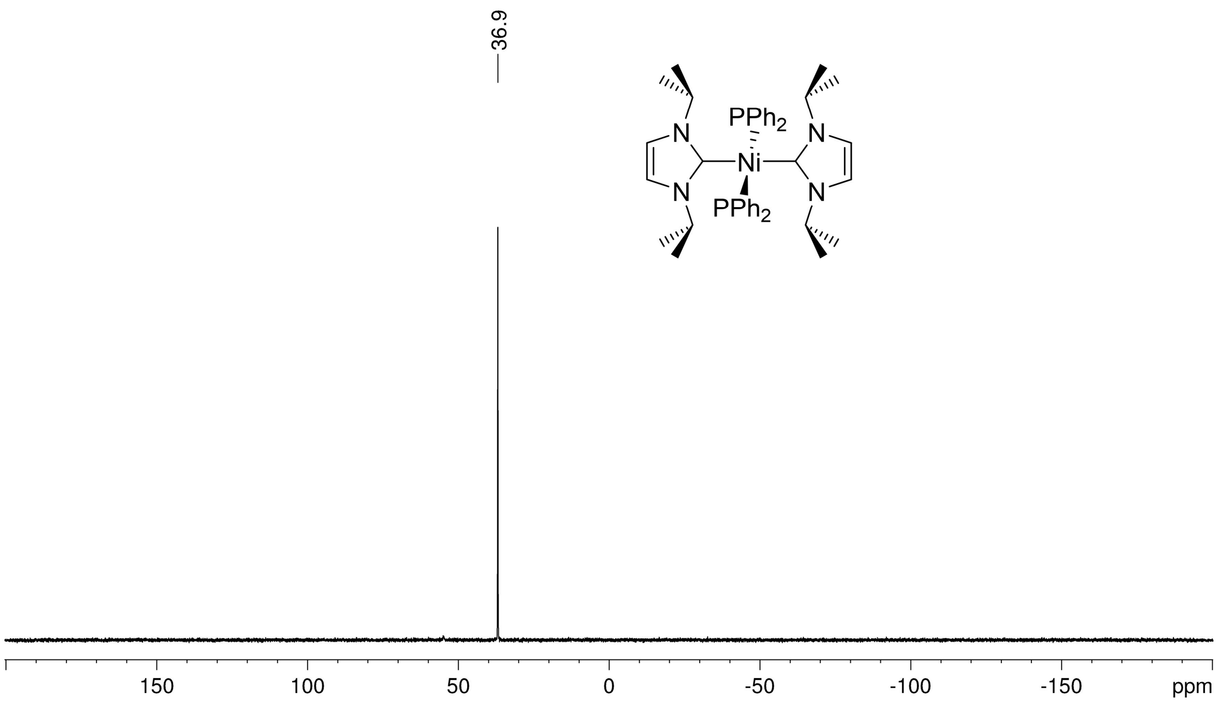

Figure S23. ^{31}P NMR spectrum (81 MHz, [D6]benzene, 298 K) of *trans*-[Ni(I^{i}Pr2)2(PPh2)2] (**8**).

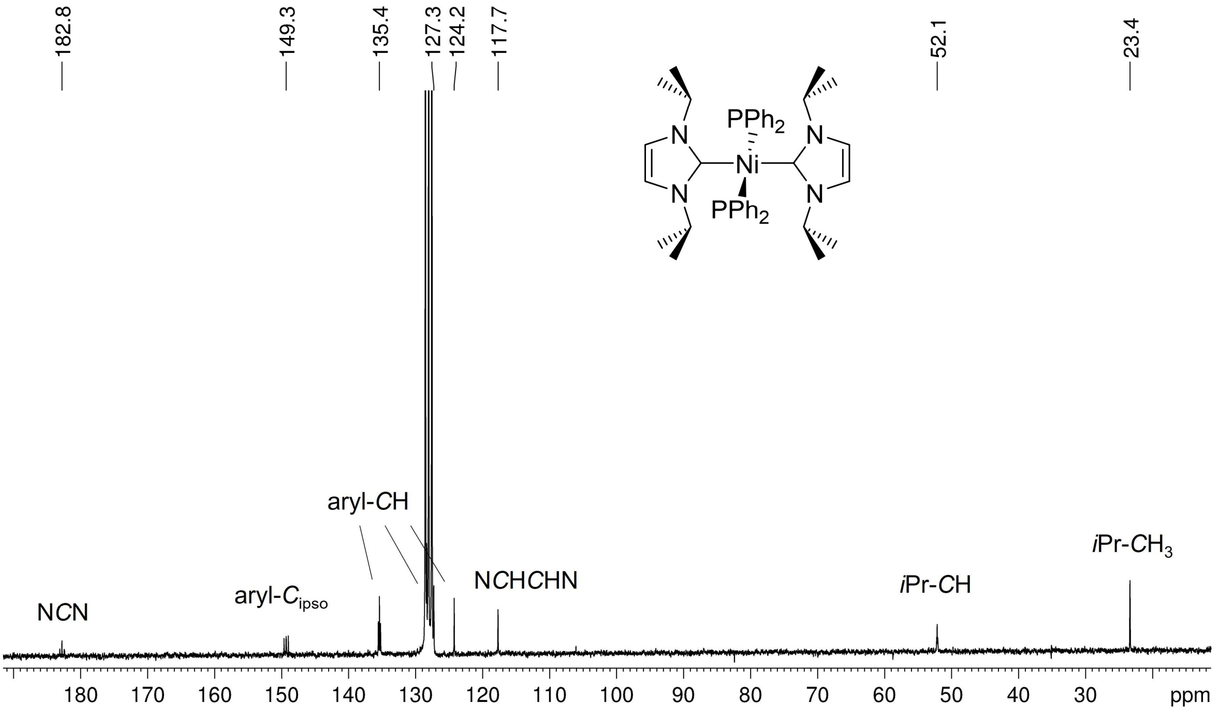

Figure S24. $^{13}C\{^1H\}$ NMR spectrum (50 MHz, [D$_6$]benzene, 298 K) of *trans*-[Ni(I^{i}Pr$_2$)$_2$(PPh$_2$)$_2$] (**8**).

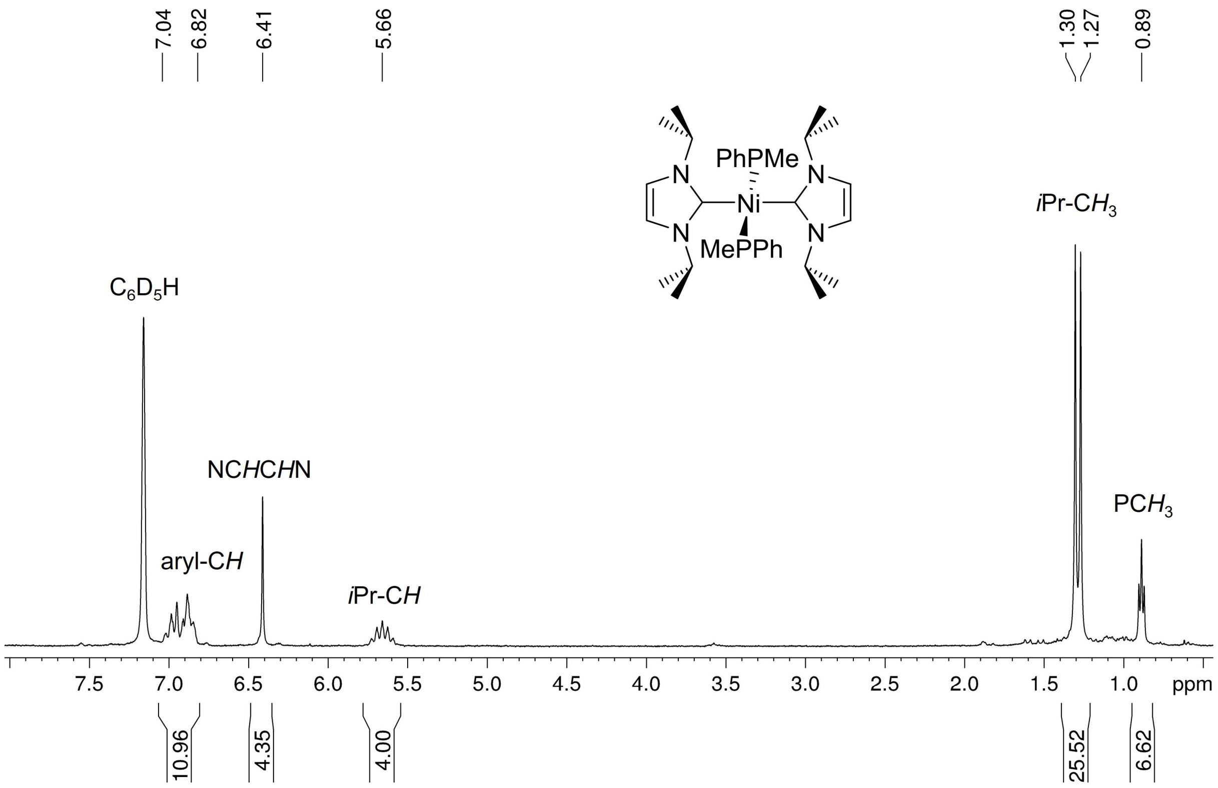

Figure S25. ^1H NMR spectrum (200 MHz, [D$_6$]benzene, 298 K) of *trans*-[Ni(I^{i}Pr$_2$)$_2$(PPhMe)$_2$] (**9**).

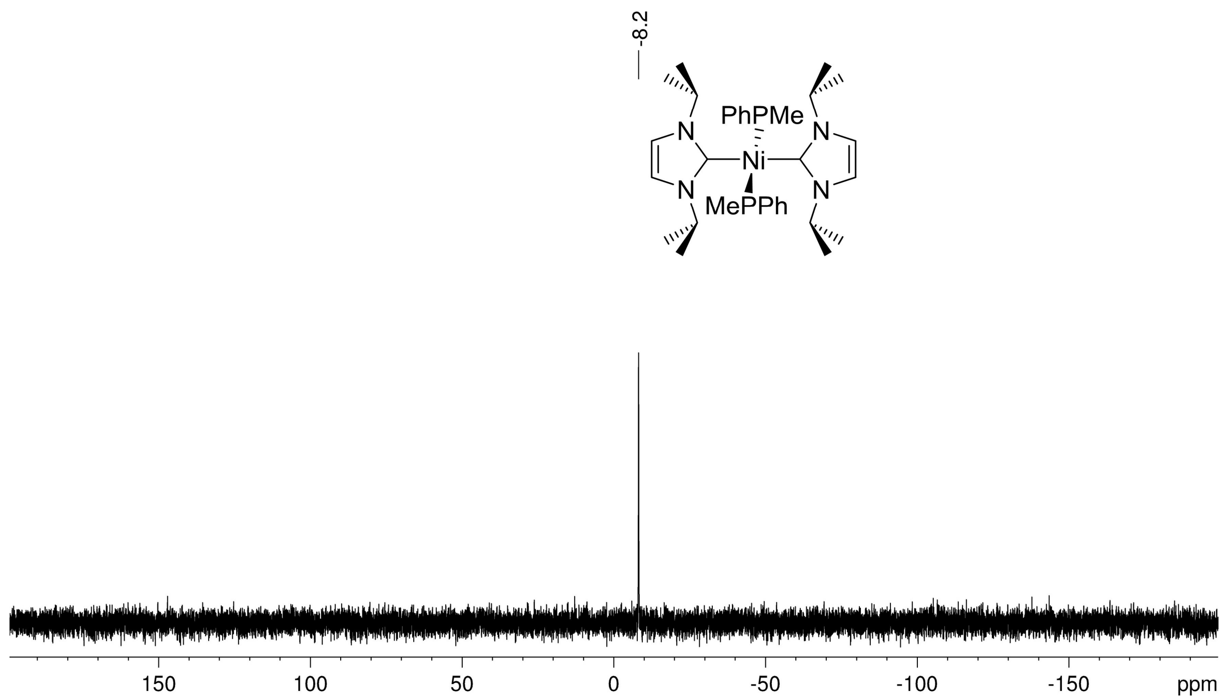

Figure S26. ^{31}P NMR spectrum (50 MHz, [D$_6$]benzene, 298 K) of *trans*-[Ni(I^{i}Pr$_2$)$_2$(PPhMe)$_2$] (**9**).

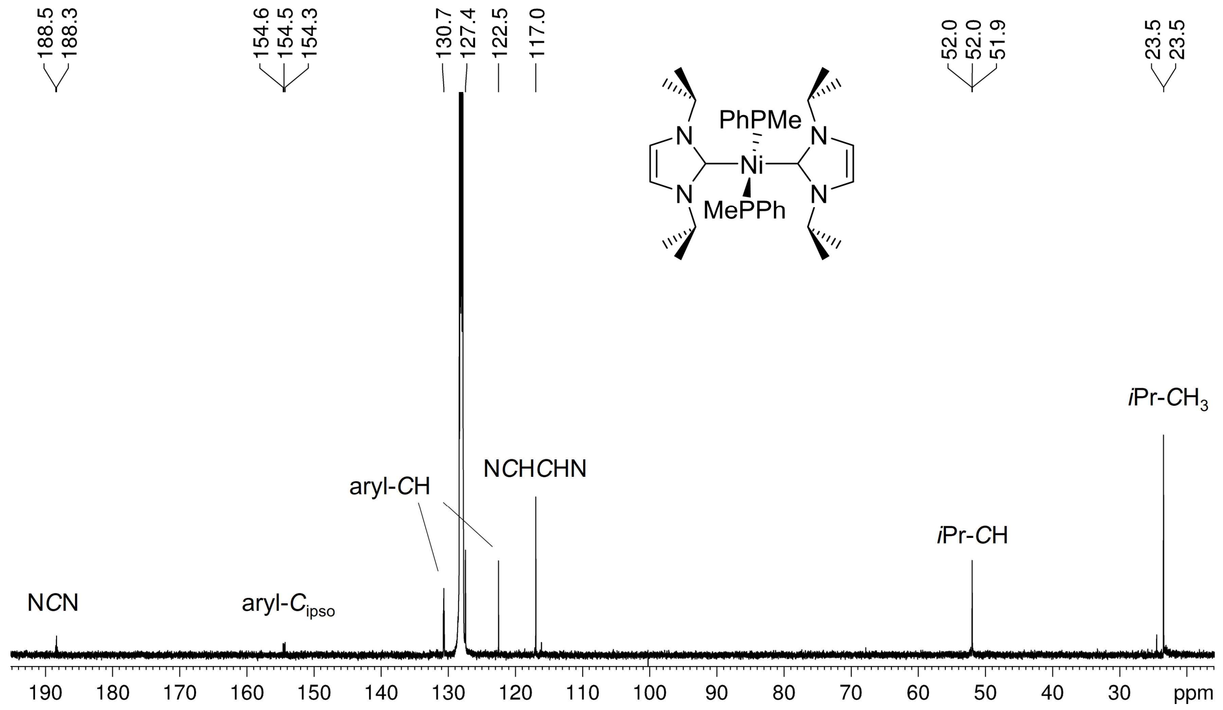

Figure S27. $^{13}C\{^{1}H\}$ NMR spectrum (126 MHz, [D$_6$]benzene, 298 K) of *trans*-[Ni(I^{i}Pr$_2$)$_2$(PPhMe)$_2$] (**9**).

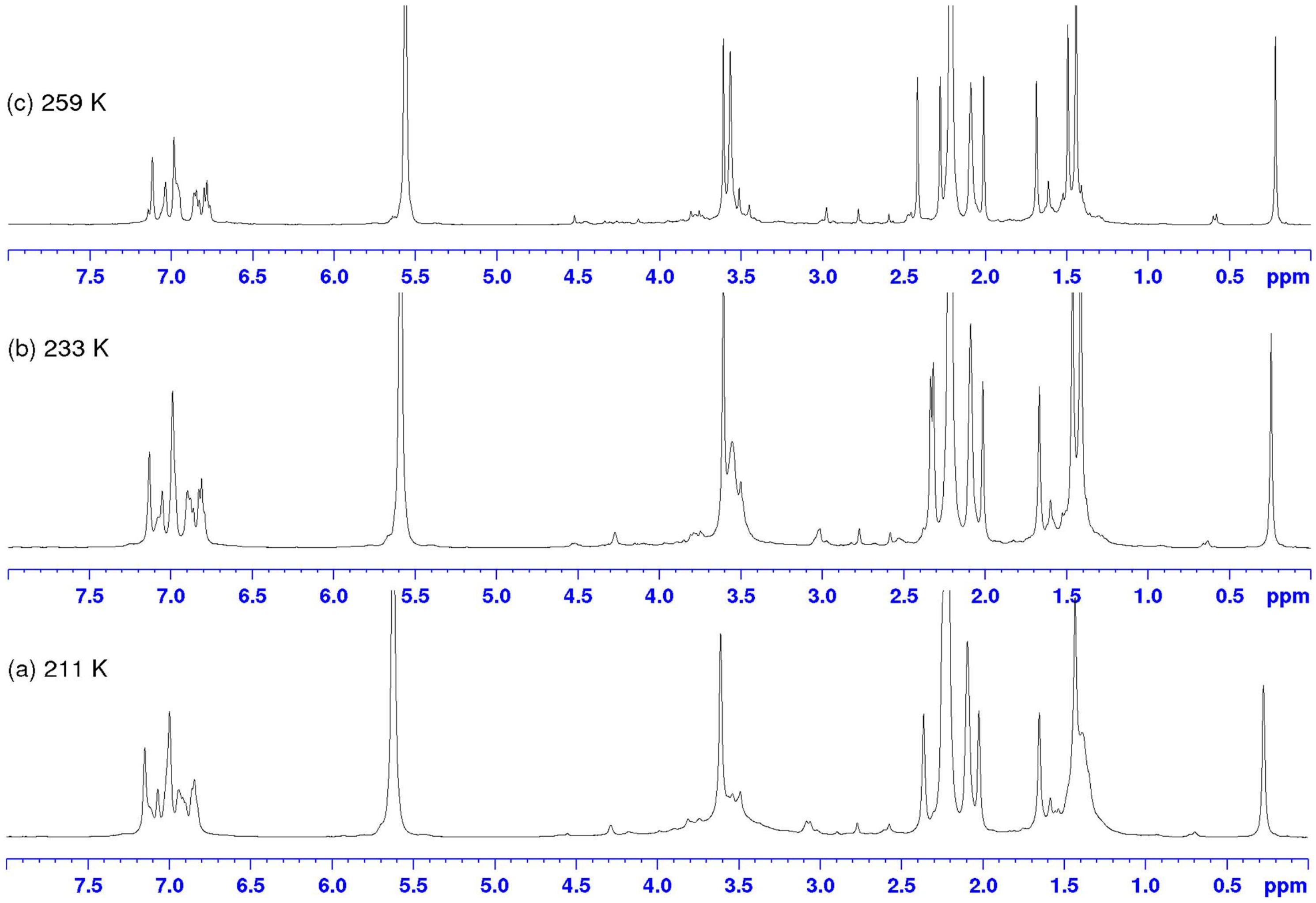

Figure S28. High frequency region of the ^{1}H NMR spectra (400 MHz, [D_8]toluene) of the reaction of [Ni(COD)$_2$], IMe$_4$ and PPhH$_2$ (1:2:1 ratio) inserted into a pre-cooled NMR spectrometer at (a) 211 K and warmed therein to (b) 233 K and (c) 259 K highlighting the formation of more than one species due to the presence of both *trans*-[Ni(IMe$_4$)$_2$(PPhH)H] (**10**) and *trans*-[Ni(IMe$_4$)$_2$(PPhH)$_2$] (**11**).

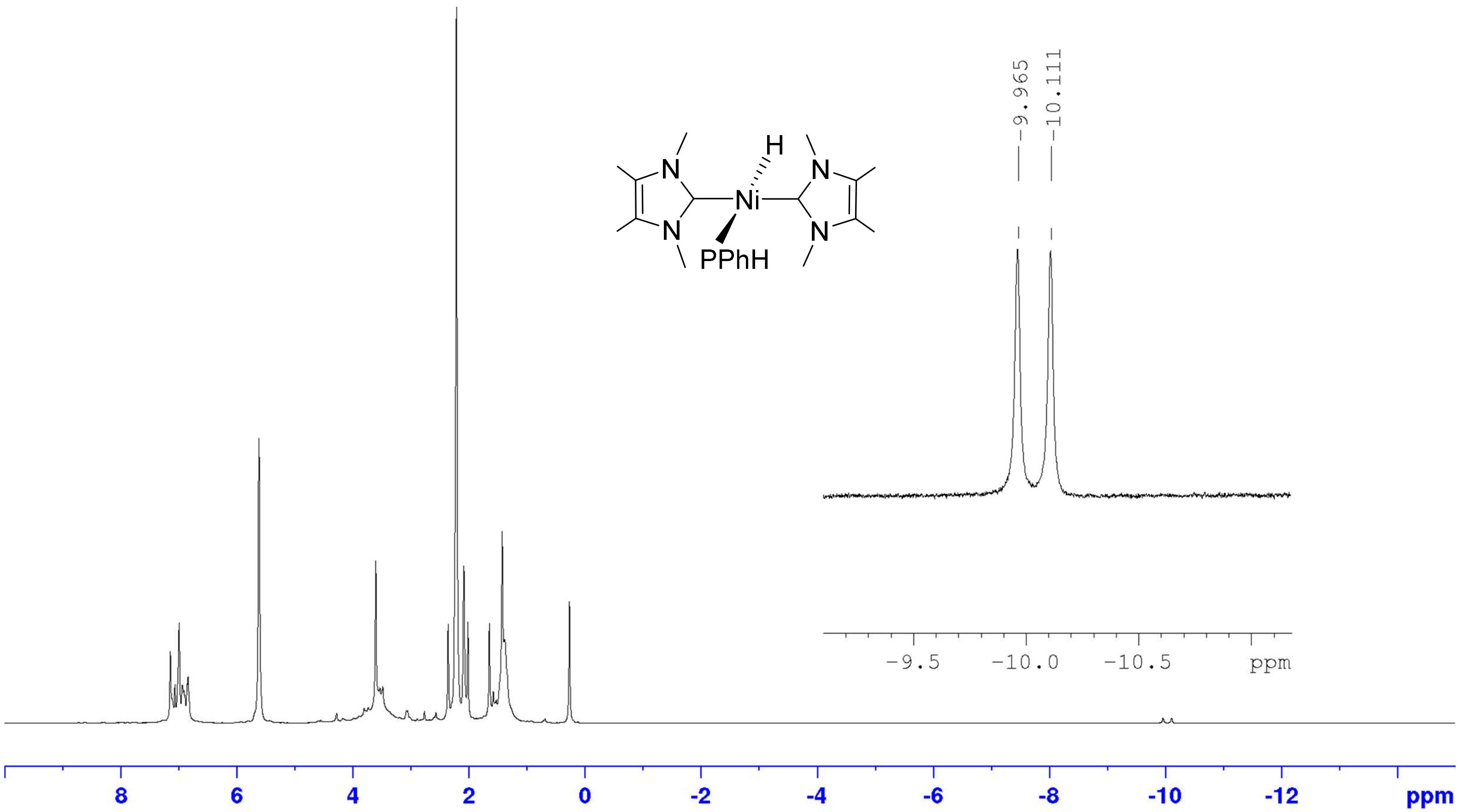

Figure S29. ^{1}H NMR spectrum (400 MHz, [D8]toluene, 211 K) of the reaction of [Ni(COD)2], IMe4 and PPhH2 (1:2:1 ratio) inserted into a pre-cooled NMR spectrometer at 211 K. Inset highlights hydride signal of *trans*-[Ni(IMe4)2(PPhH)H] (**10**).

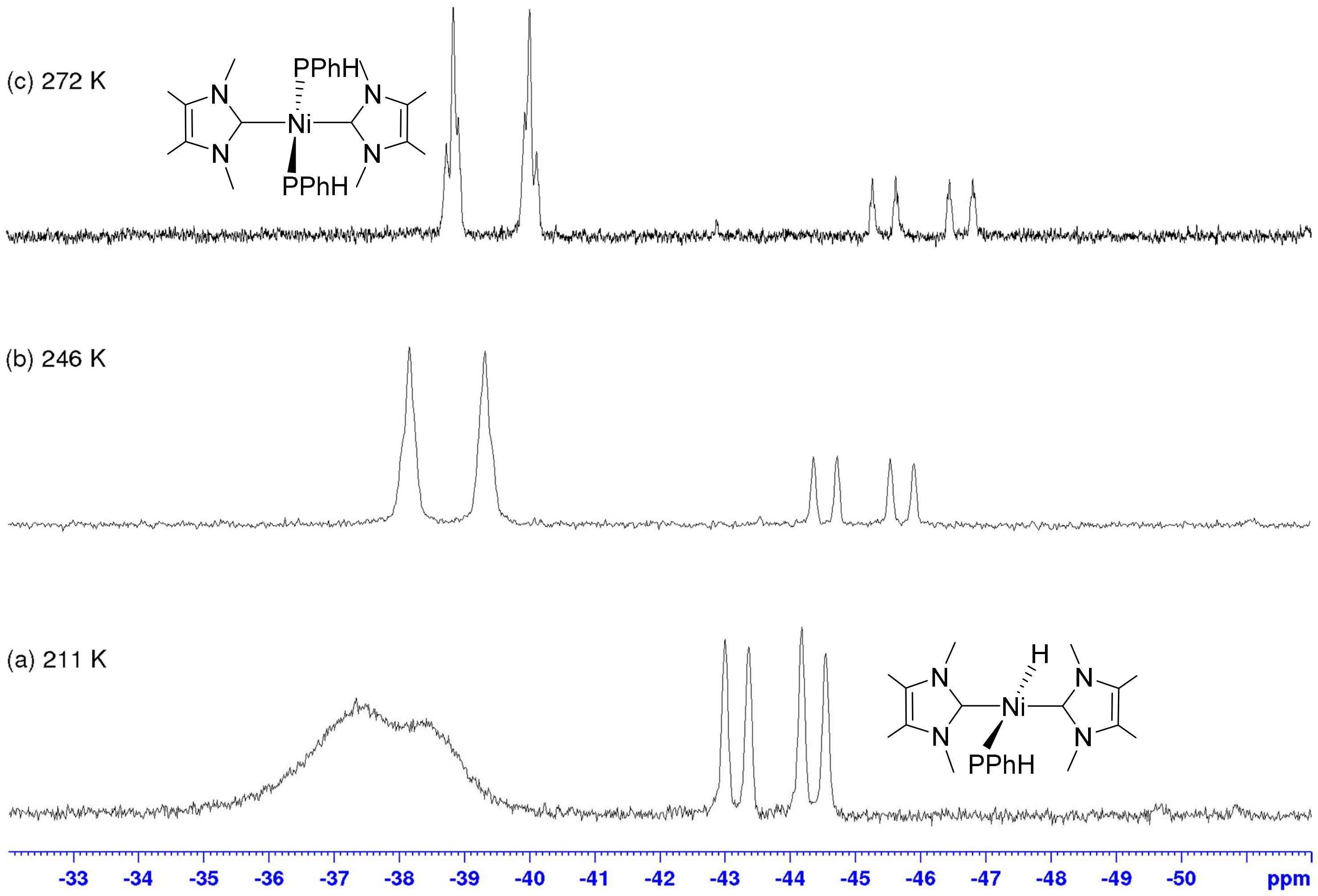

Figure S30 ^{31}P NMR spectra (162 MHz, [D$_8$]toluene) of the reaction of [Ni(COD)$_2$], IMe$_4$ and PPhH$_2$ (1:2:1 ratio) inserted into a pre-cooled NMR spectrometer at (a) 211 K and warmed therein to (b) 246 K and (c) 272 K illustrating the formation of both *trans*-[Ni(IMe$_4$)$_2$(PPhH)H] (**10**) and *trans*-[Ni(IMe$_4$)$_2$(PPhH)$_2$] (**11**).

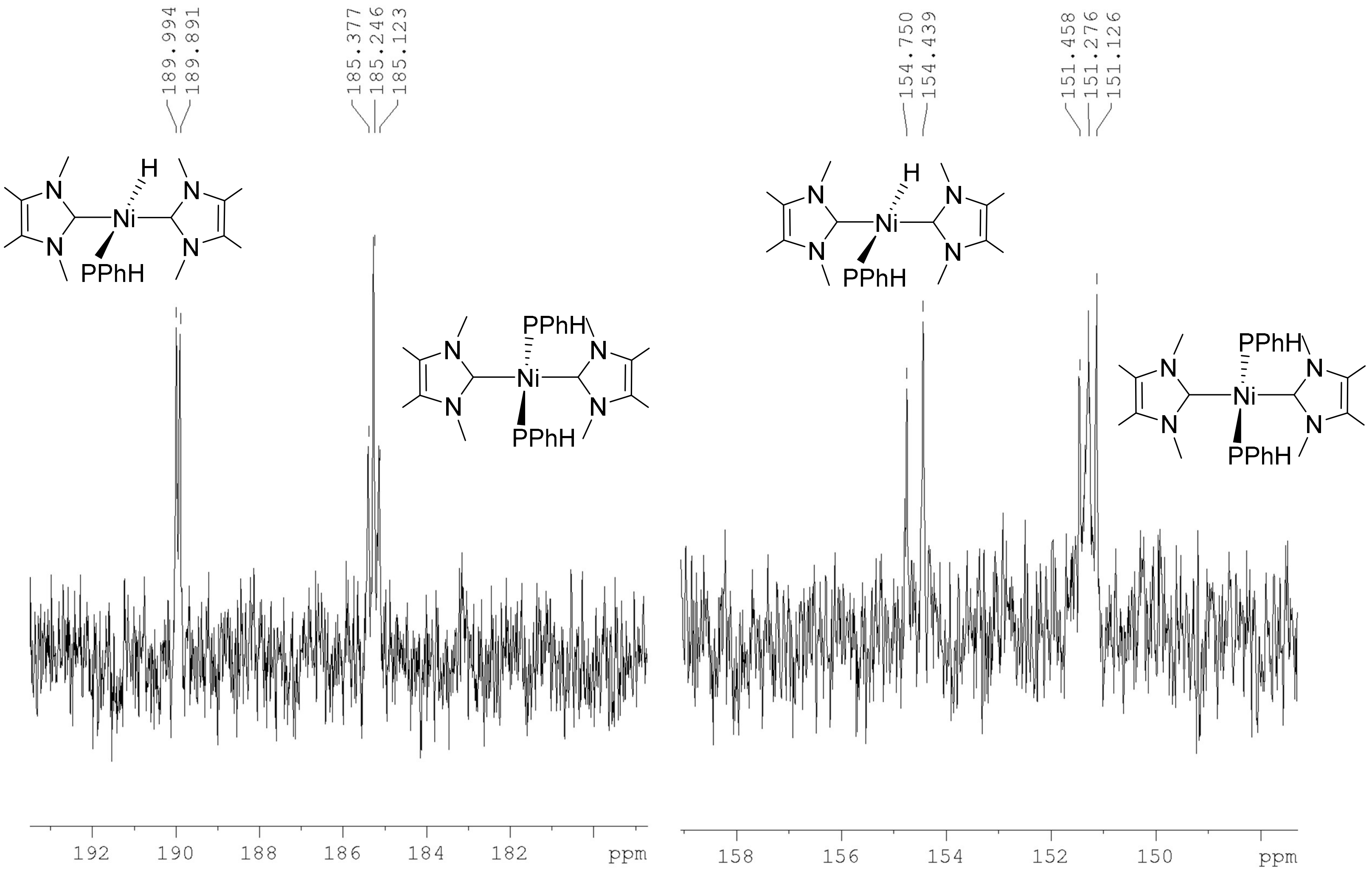

Figure S31. High frequency region of the low temperature $^{13}C\{^{1}H\}$ NMR spectrum (101 MHz, [D$_8$]toluene, 211 K) of the reaction of [Ni(COD)$_2$], IMe$_4$ and PPhH$_2$ (1:2:1 ratio) inserted into a pre-cooled NMR spectrometer at 211 K showing NHC and *ipso*-*C*-PPh$_2$ resonances of *trans*-[Ni(IMe$_4$)$_2$(PPhH)H] (**10**) and *trans*-[Ni(IMe$_4$)$_2$(PPhH)$_2$] (**11**).

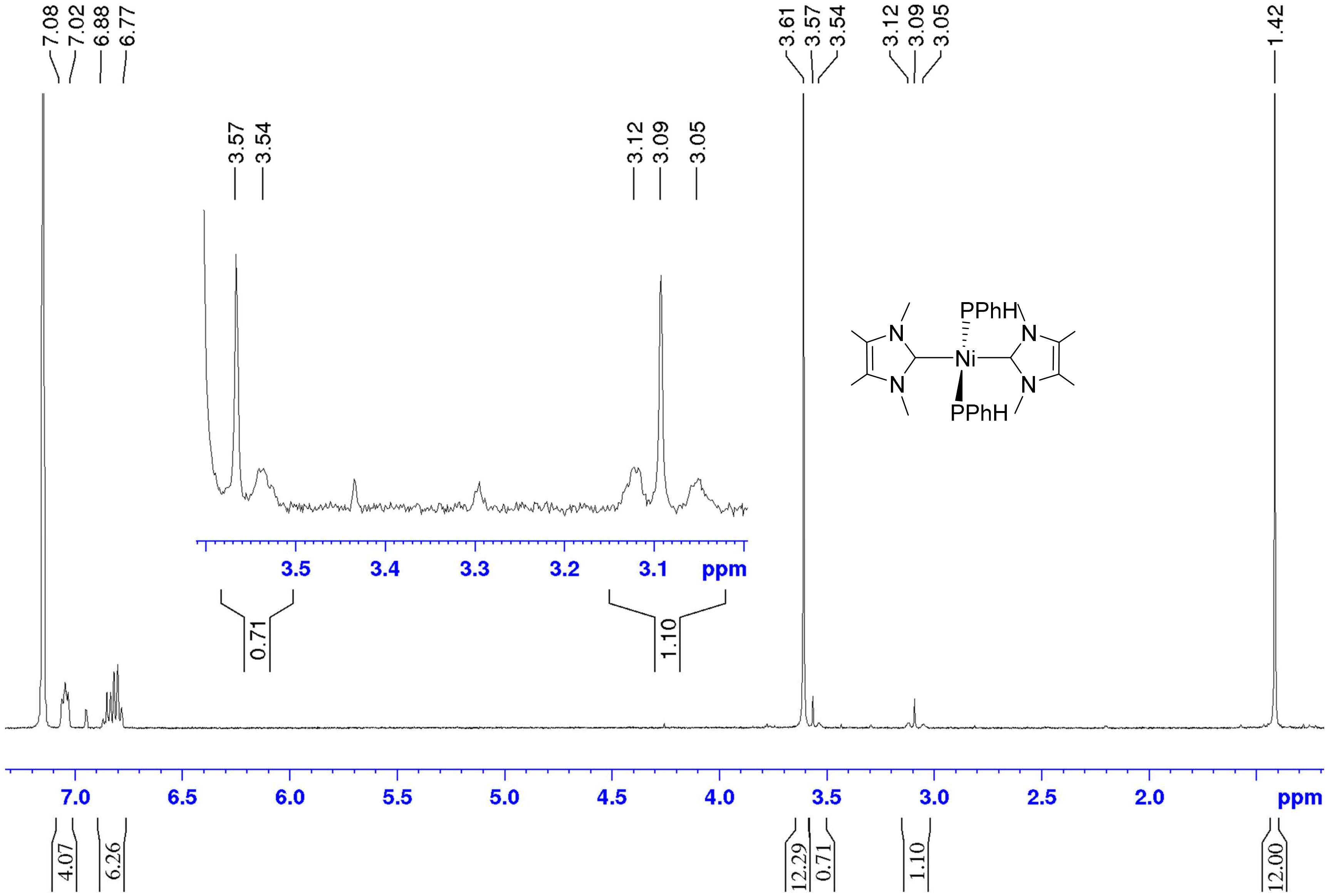

Figure S32. ^{1}H NMR spectrum (500 MHz, [D$_6$]benzene, 298 K) of *trans*-[Ni(IMe$_4$)$_2$(PPhH)$_2$] (**11**). Inset shows the P-*H* resonance.

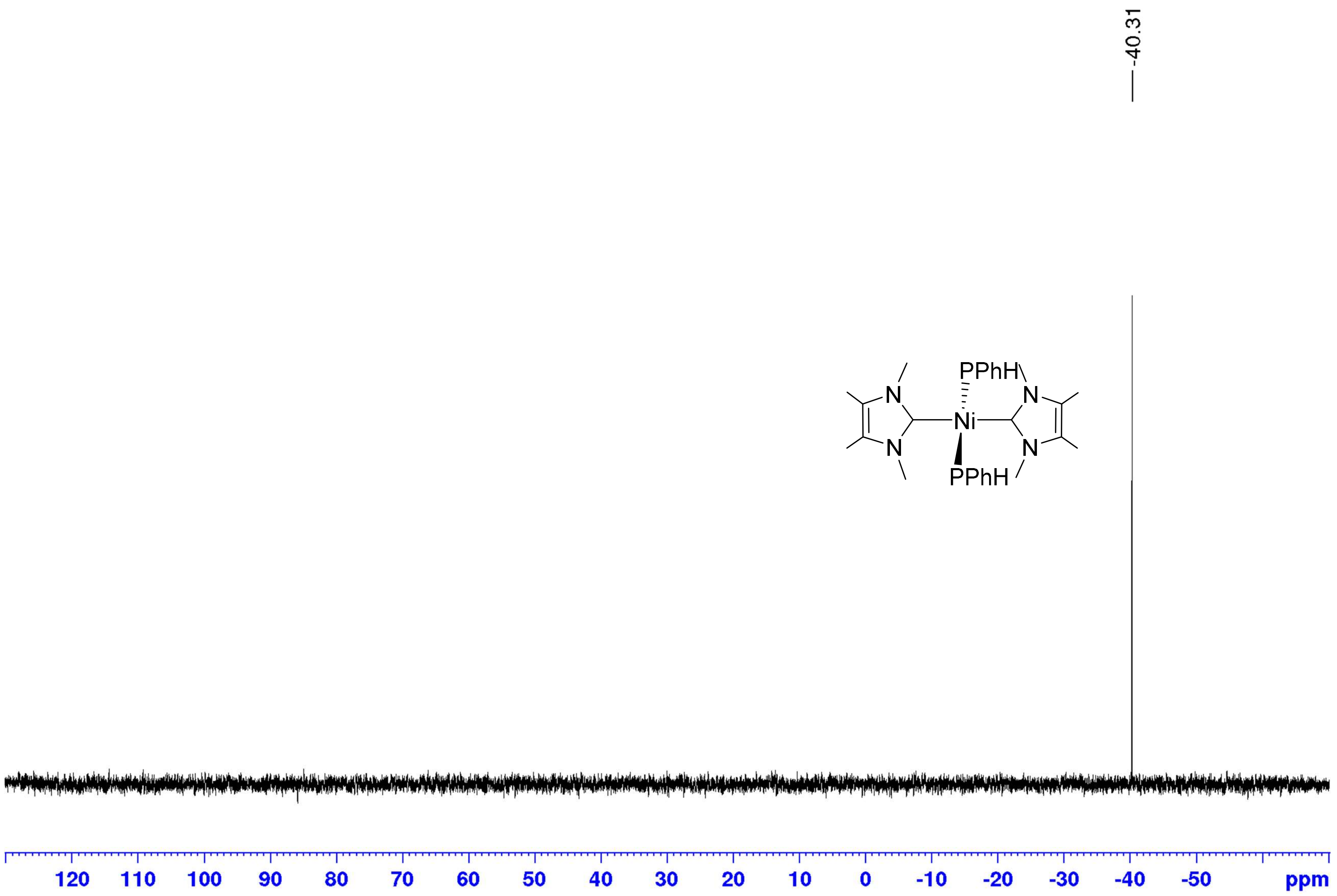

Figure S33. $^{31}P\{^{1}H\}$ NMR spectrum (162 MHz, [D6]benzene, 298 K) of *trans*-[Ni(IMe4)2(PPhH)2] (**11**).

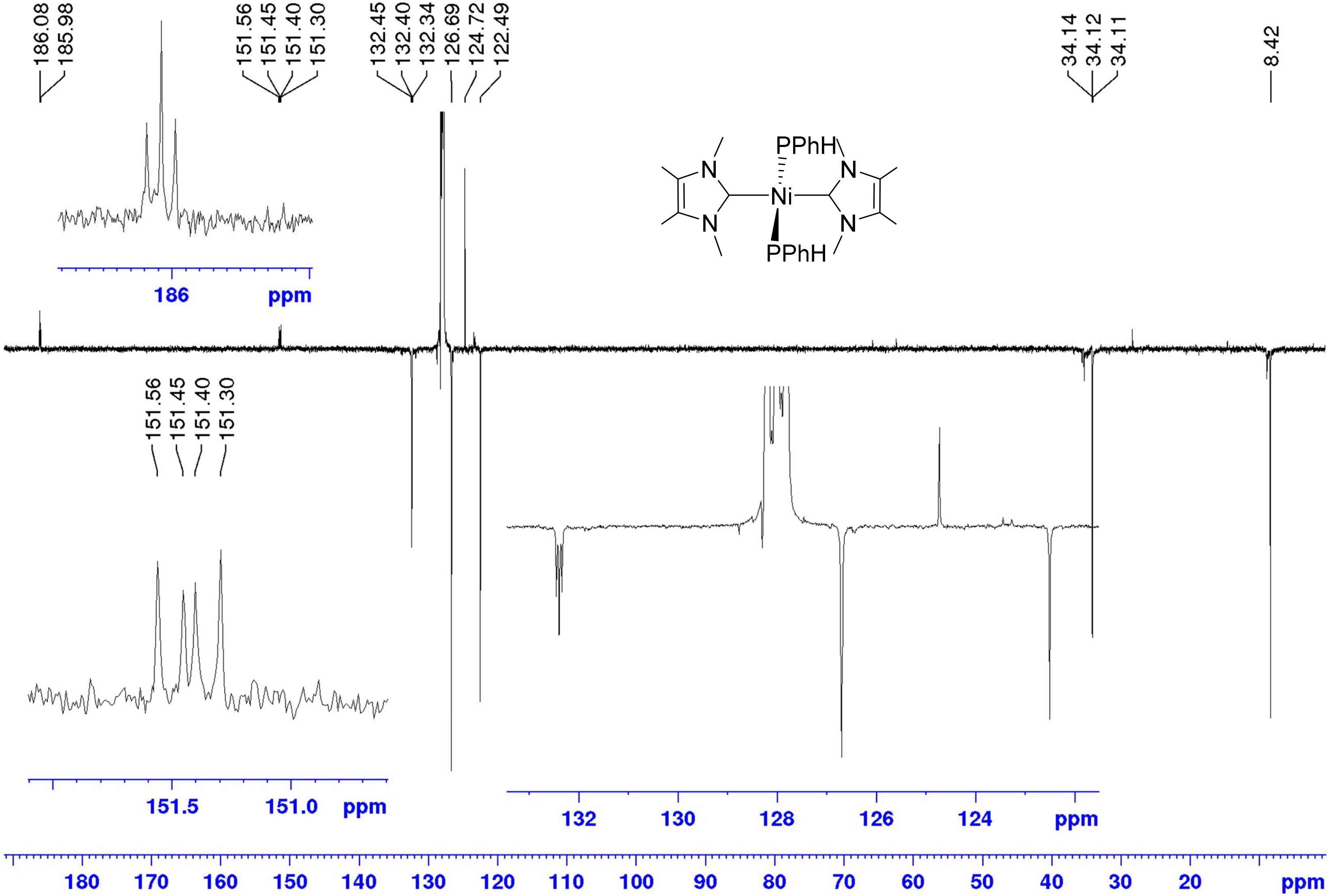

Figure S34. $^{13}C\{^1H\}$ PENDANT NMR spectrum (101 MHz, [D$_6$]benzene, 298 K) of *trans*-[Ni(IMe$_4$)$_2$(PPhH)$_2$] (**11**).

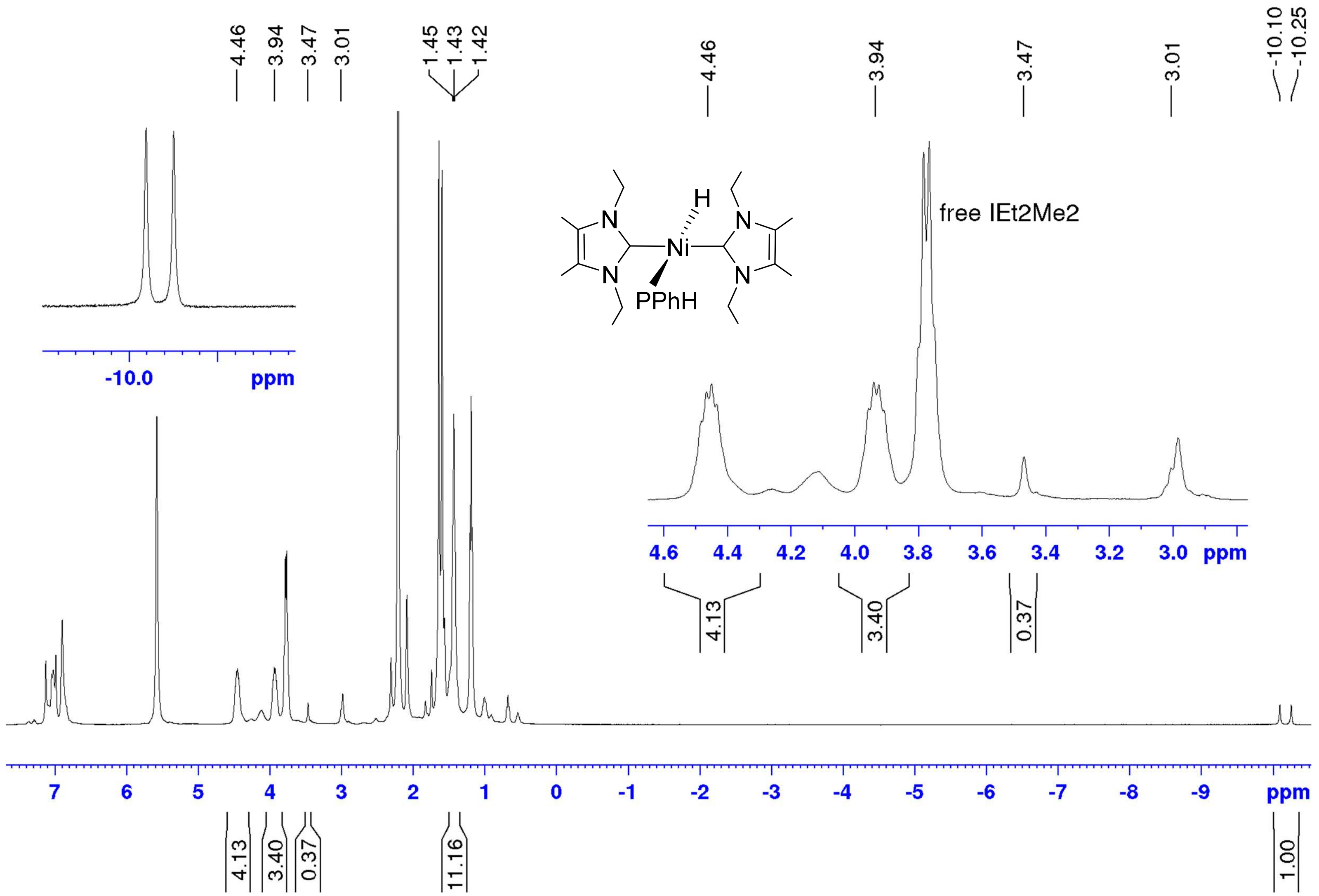

Figure S35. ^{1}H NMR spectrum (400 MHz, [D_8]toluene, 236 K) of the reaction of [Ni(COD)$_2$], IEt$_2$Me$_2$ and PPhH$_2$ (1:2:1 ratio) inserted into a pre-cooled (223 K) NMR spectrometer. Resonances of *trans*-[Ni(IEt$_2$Me$_2$)$_2$(PPhH)H] (**12**) are peak picked and integrated.

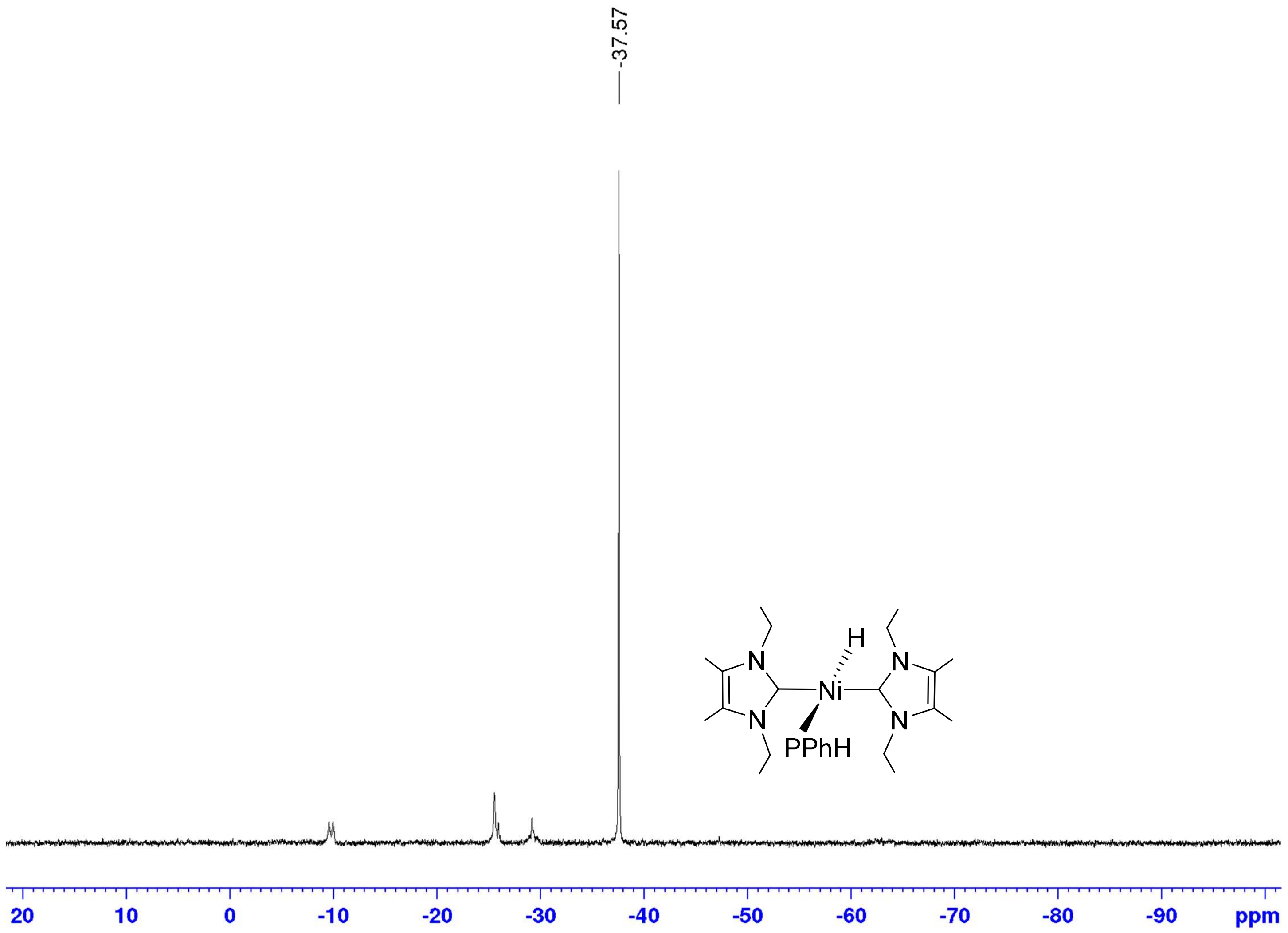

Figure S36. $^{31}P\{^{1}H\}$ NMR spectrum (162 MHz, [D$_8$]toluene, 223 K) of the reaction of [Ni(COD)$_2$], IEt$_2$Me$_2$ and PPhH$_2$ (1:2:1 ratio) inserted into a pre-cooled (223 K) NMR spectrometer. Main signal arises from *trans*-[Ni(IEt$_2$Me$_2$)$_2$(PPhH)H] (**12**).

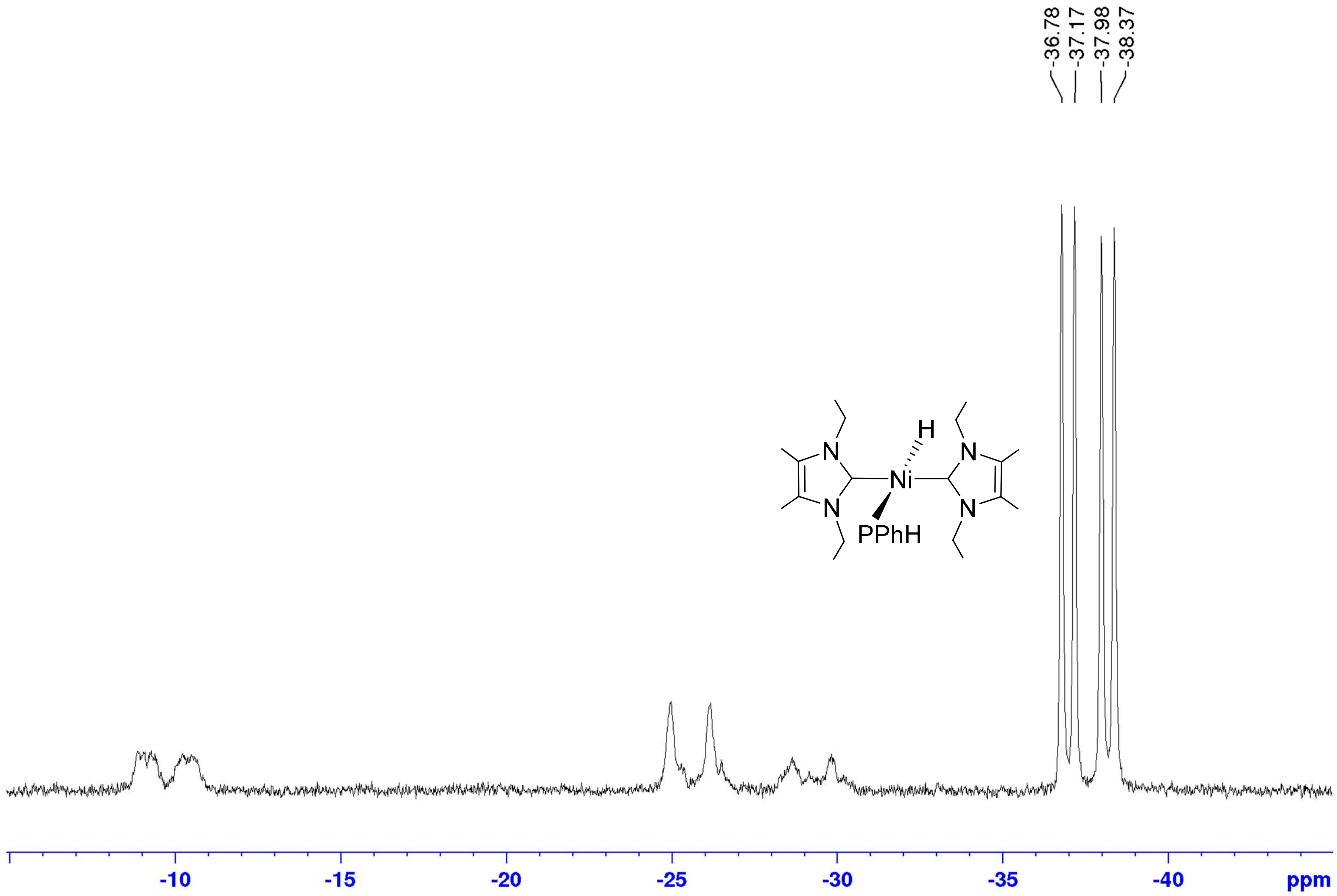

Figure S37. ^{31}P NMR spectrum (162 MHz, [D$_8$]toluene, 223 K) of the reaction of [Ni(COD)$_2$], IEt$_2$Me$_2$ and PPhH$_2$ (1:2:1 ratio) inserted into a pre-cooled (223 K) NMR spectrometer. Main doublet of doublets signal arises from *trans*-[Ni(IEt$_2$Me$_2$)$_2$(PPhH)H] (**12**).

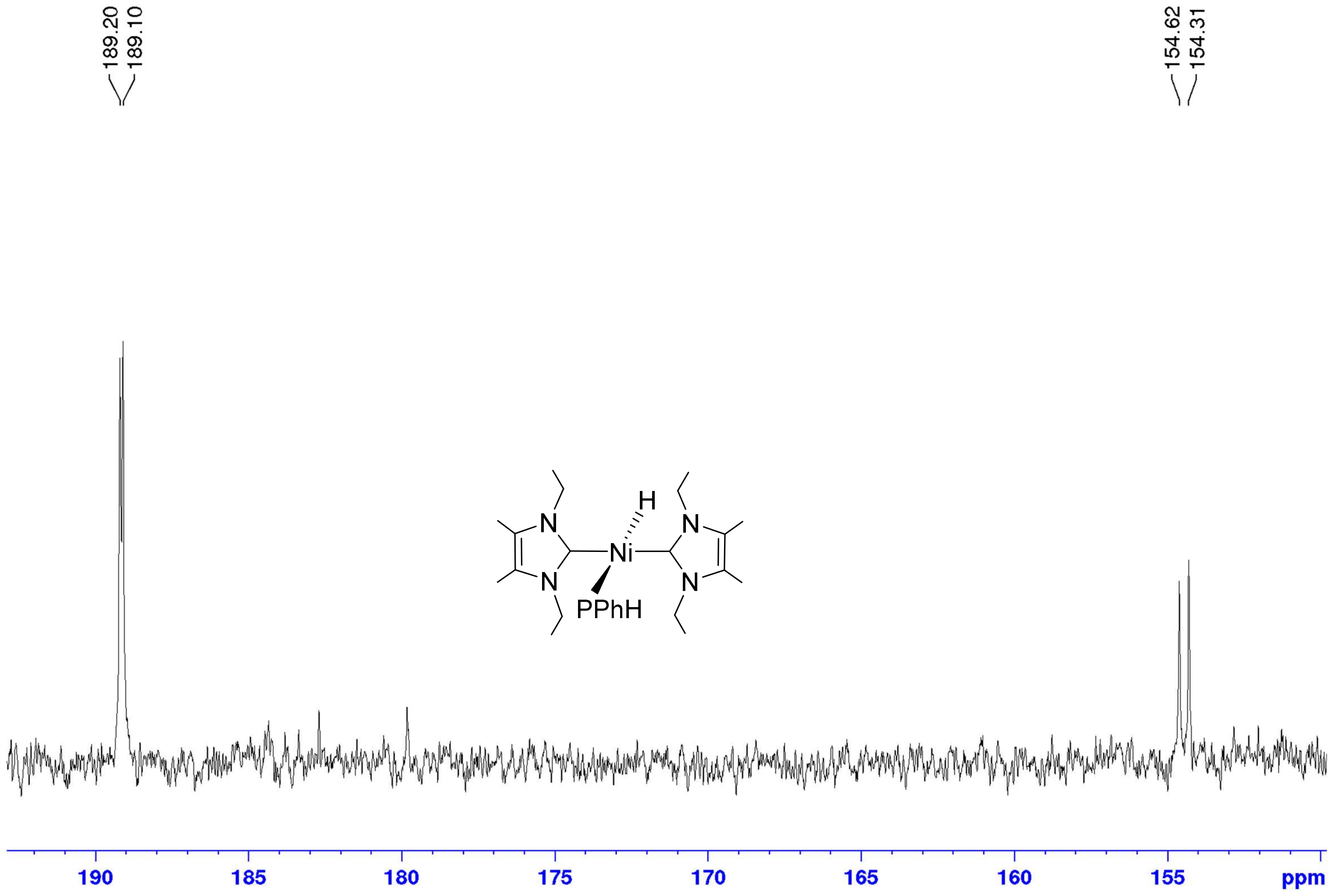

Figure S38. High frequency region of the $^{13}C\{^{1}H\}$ PENDANT NMR spectrum (101 MHz, [D_8]toluene, 223 K) of the reaction of [Ni(COD)$_2$], IEt$_2$Me$_2$ and PPhH$_2$ (1:2:1 ratio) inserted into a pre-cooled (223 K) NMR spectrometer. Shown are the NHC and *ipso*-*C*-PPhH signals of *trans*-[Ni(IEt$_2$Me$_2$)$_2$(PPhH)H] (**12**).

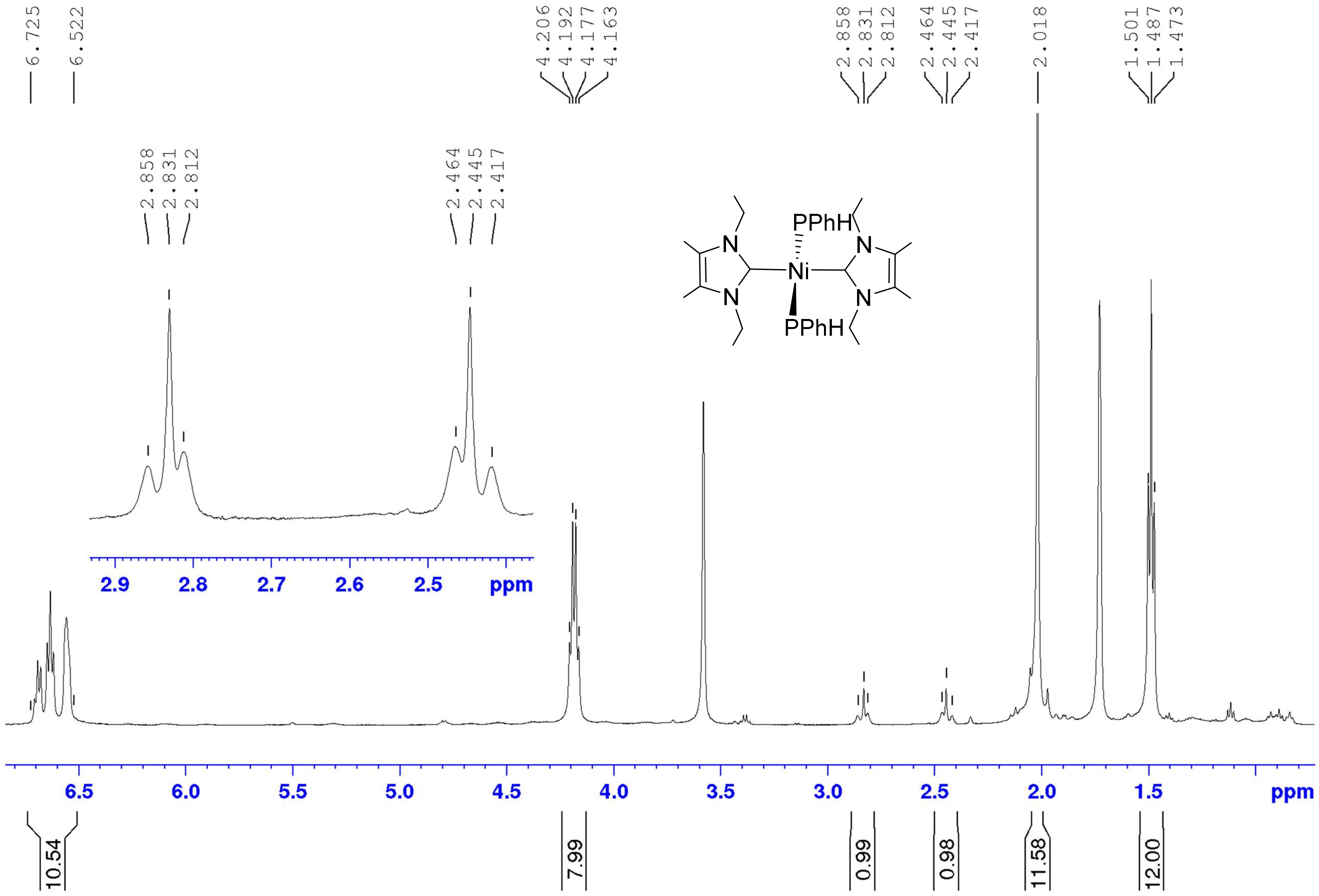

Figure S39. ^{1}H NMR spectrum (500 MHz, $[D_8]$THF, 298 K) of *trans*-$[Ni(IEt_2Me_2)_2(PPhH)_2]$ (**13**). Inset highlights P-*H* resonance.

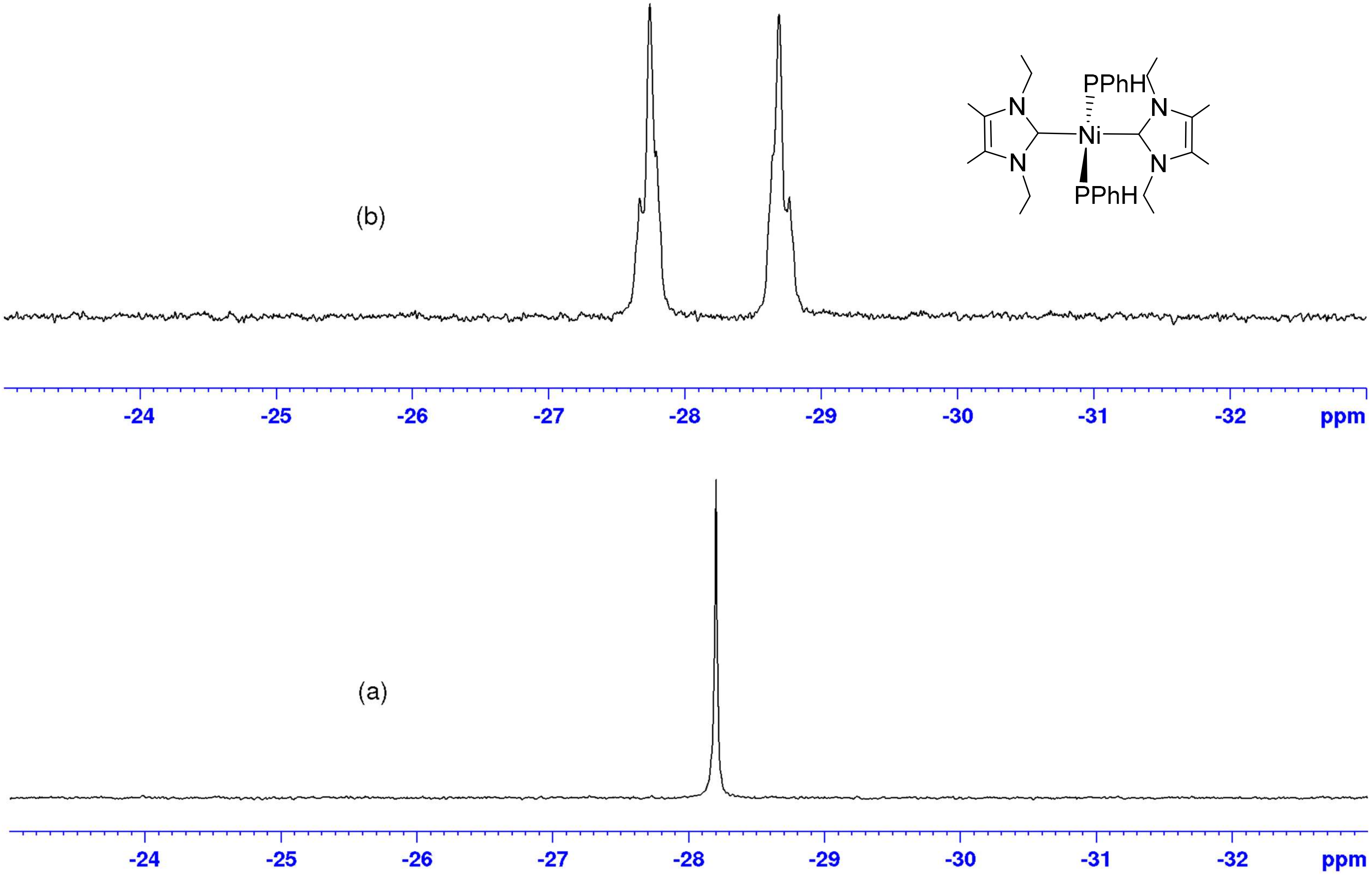

Figure S40. (a) $^{31}P\{^{1}H\}$ and (b) ^{31}P NMR spectra (202 MHz, [D$_8$]THF, 298 K) of *trans*-[Ni(IEt$_2$Me$_2$)$_2$(PPhH)$_2$] (**13**).

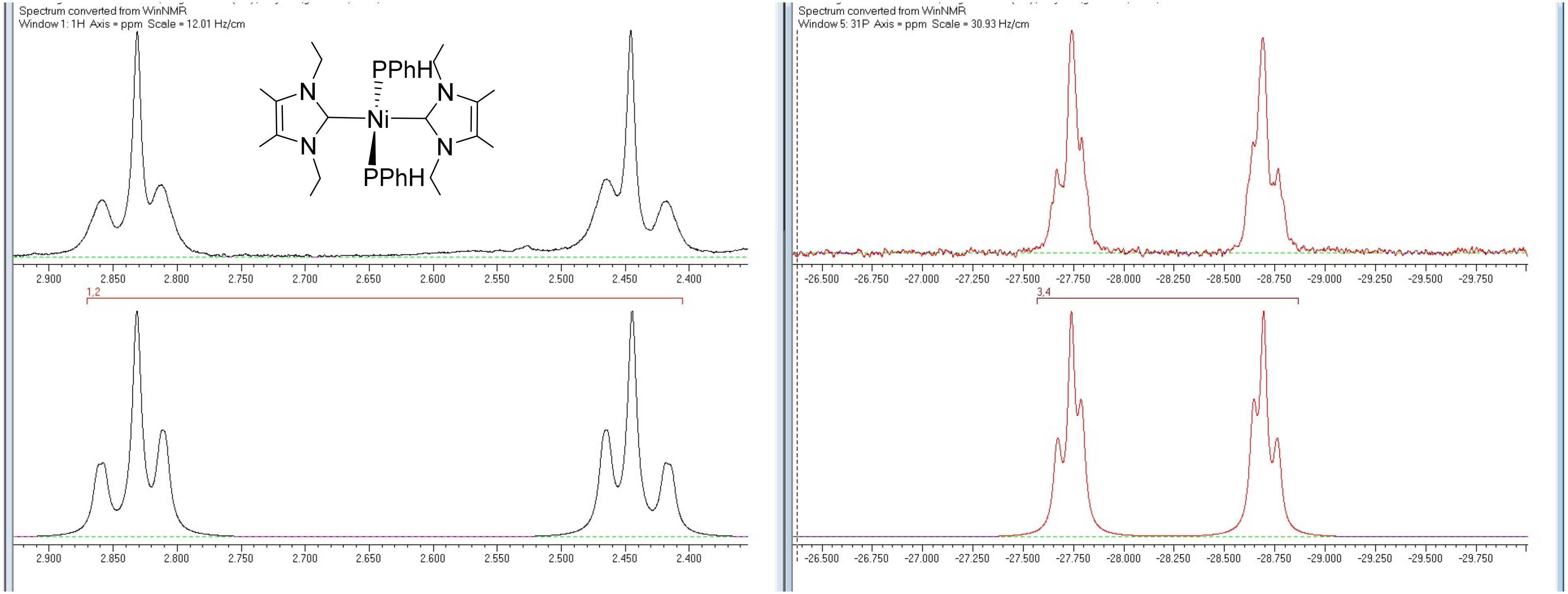

Figure S41. Experimental (500/162 MHz, [D_8]THF, 298 K; top) and simulated (bottom) P-*H* resonance (left) and ^{31}P signal (right) of *trans*-[Ni(IEt_2Me_2)$_2$(PPhH)$_2$] (**13**). Simulation parameters: $^{1}J_{PH}$ = 194.6 Hz, $^{4}J_{HH}$ = 2.43 Hz $^{3}J_{PH}$ = -1.12 Hz, $^{2}J_{PP}$ = 24.35 Hz. For a comparison to these values, see the homoleptic secondary phosphine complexes, (tetrahedral) [Ni(PMesH_2)$_4$] and (square planar) [Pd(PMesH_2)$_4$]$^{2+}$. I. V. Kourkine, S. V. Maslennikov, R. Ditchfield, D. S. Glueck, G. P. A. Yap, L. M. Liable-Sands, A. L. Rheingold, *Inorg. Chem*. **1996**, *35*, 6708-6716.

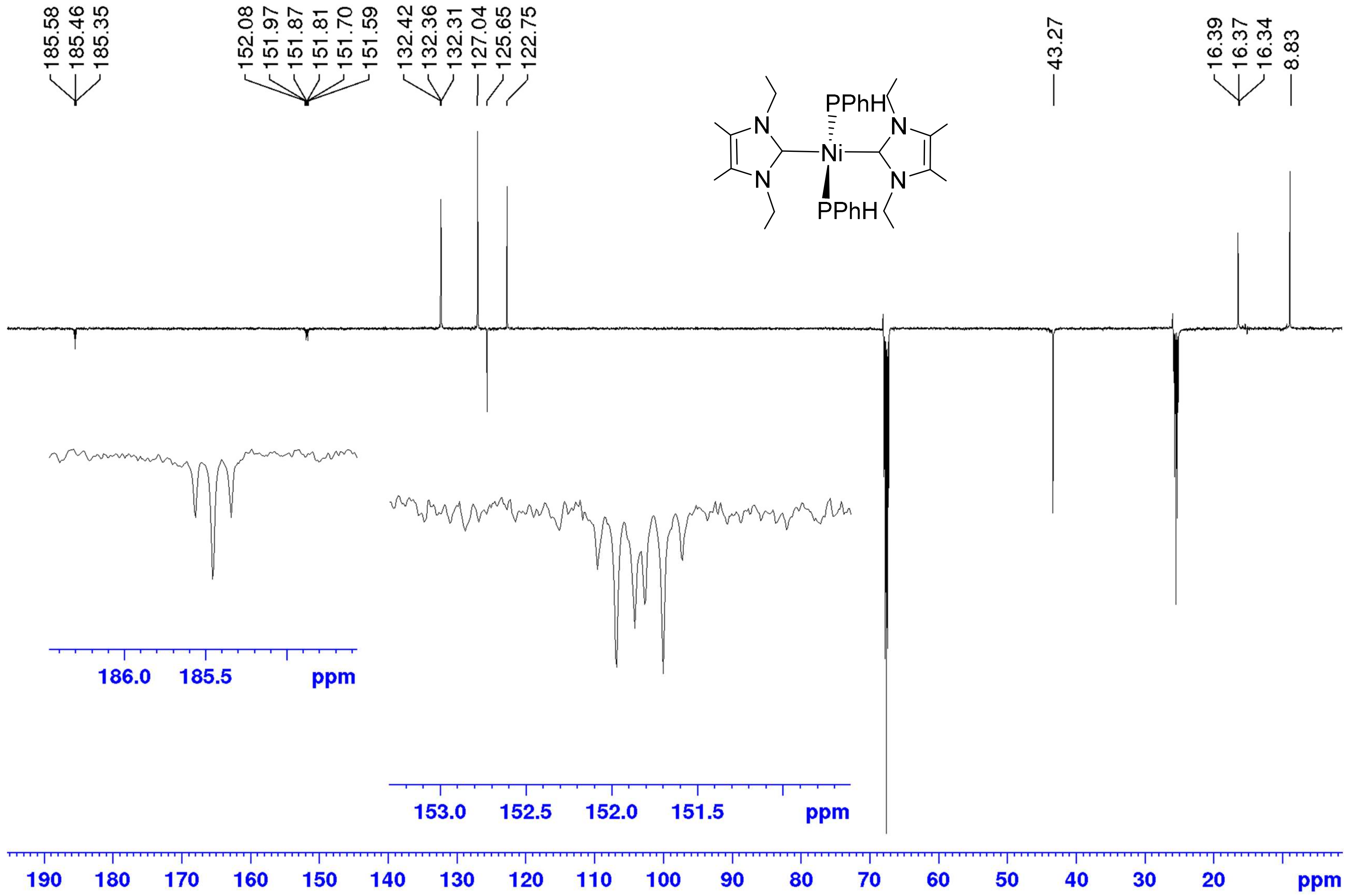

Figure S42. $^{13}C\{^{1}H\}$ PENDANT NMR spectrum (101 MHz, [D$_6$]benzene, 298 K) of *trans*-[Ni(IEt$_2$Me$_2$)$_2$(PPhH)$_2$] (**13**). Insets highlight NHC and *ipso*-*C*-PPh$_2$ signals.

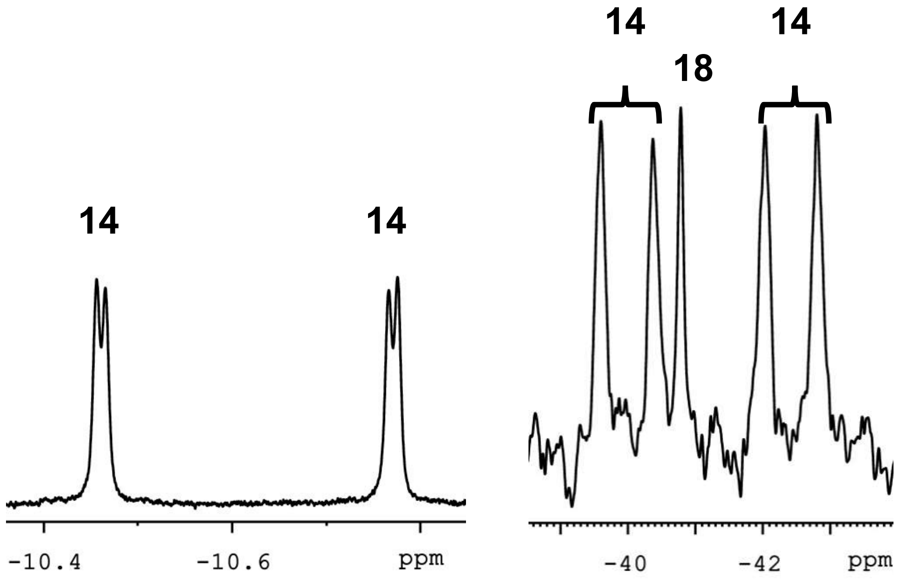

Figure S42. Selected regions of the (feft) ^{1}H NMR (500 MHz, [D$_6$]benzene, 296 K) and (right) ^{31}P NMR (202 MHz, [D$_6$]benzene, 296 K) spectra recorded after the room temperature addition of $PPhH_2$ to [Ni$_2$(I^{i}Pr$_2$)$_4$(COD)], illustrating signals assigned to *trans*-[Ni(I^{i}Pr$_2$)$_2$(PPhH)H] (**14**), alongside those resulting from [Ni(I^{i}Pr$_2$)(η^2-PhP=PPh)] (**18**).

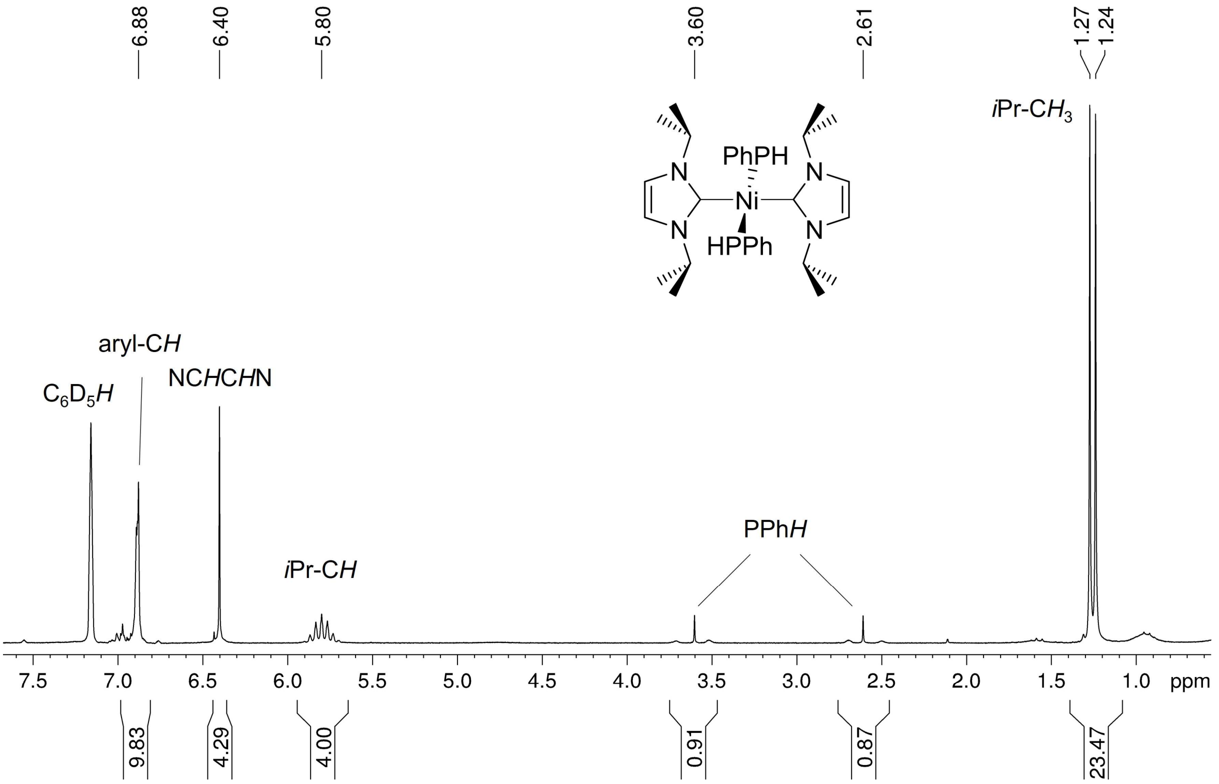

Figure S43. ^{1}H NMR spectrum (300 MHz, [D6]benzene, 296 K) of *trans*-[Ni(I^{i}Pr$_2$)$_2$(PPhH)$_2$] (**16**).

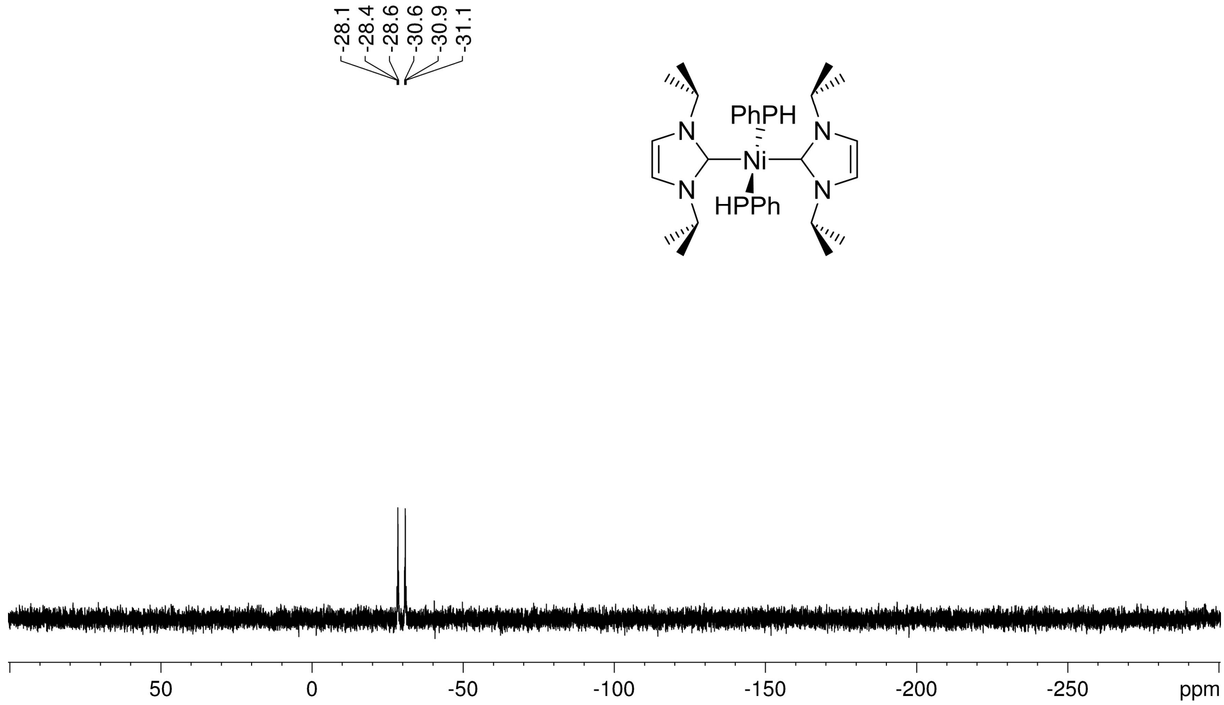

Figure S44. $^{31}P\{^{1}H\}$ NMR spectrum (202 MHz, [D6]benzene, 296 K) of *trans*-[Ni(I^{i}Pr$_2$)$_2$(PPhH)$_2$] (**16**).

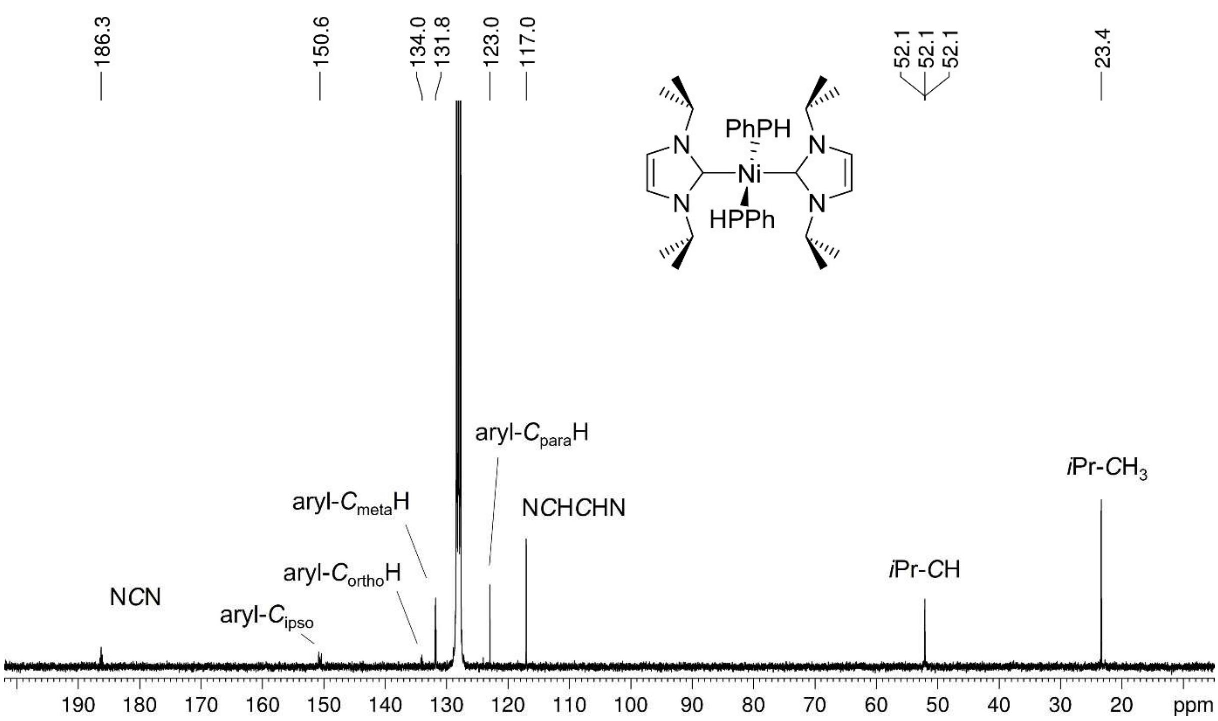

Figure S45. $^{13}C\{^1H\}$ NMR spectrum (75 MHz, [D$_6$]benzene, 296 K) of *trans*-[Ni(I^{i}Pr$_2$)$_2$(PPhH)$_2$] (**16**).

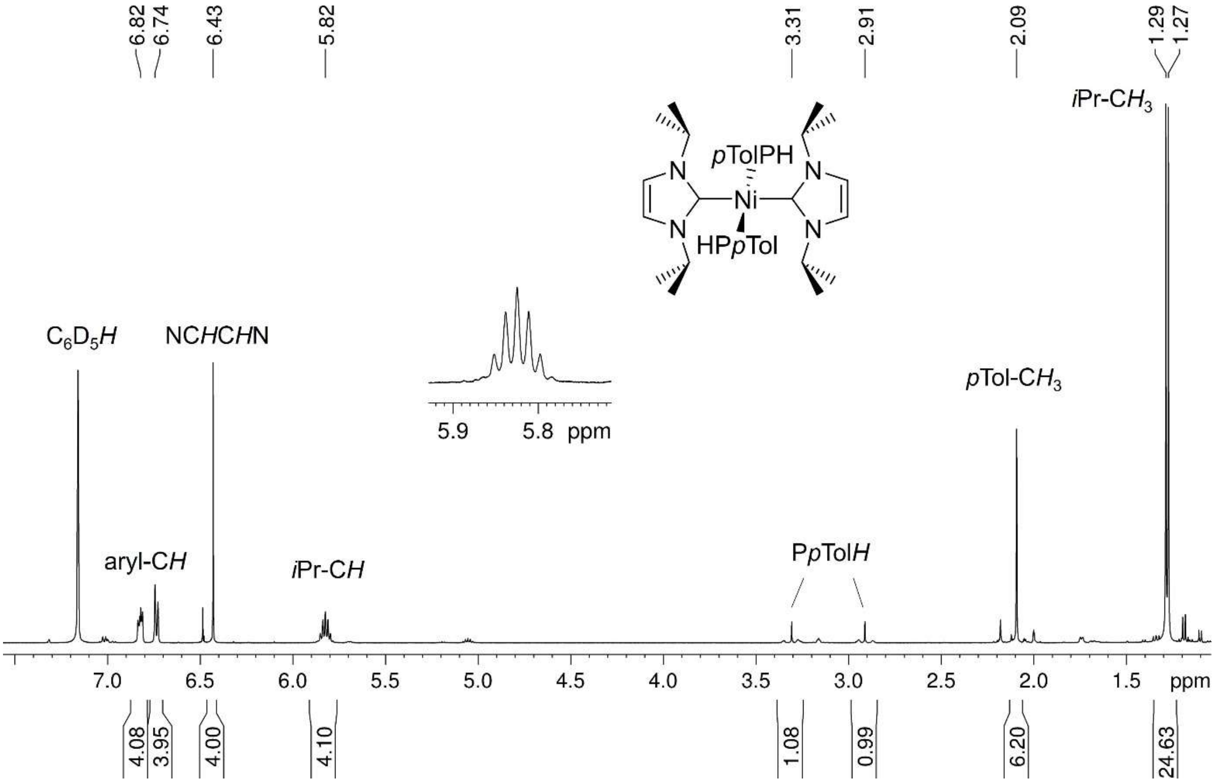

Figure S46. ^1H NMR spectrum (500 MHz, [D$_6$]benzene, 363 K) of *trans*-[Ni(I^{i}Pr$_2$)$_2$\{P(*para*-Tol)H\}$_2$] (**17**).

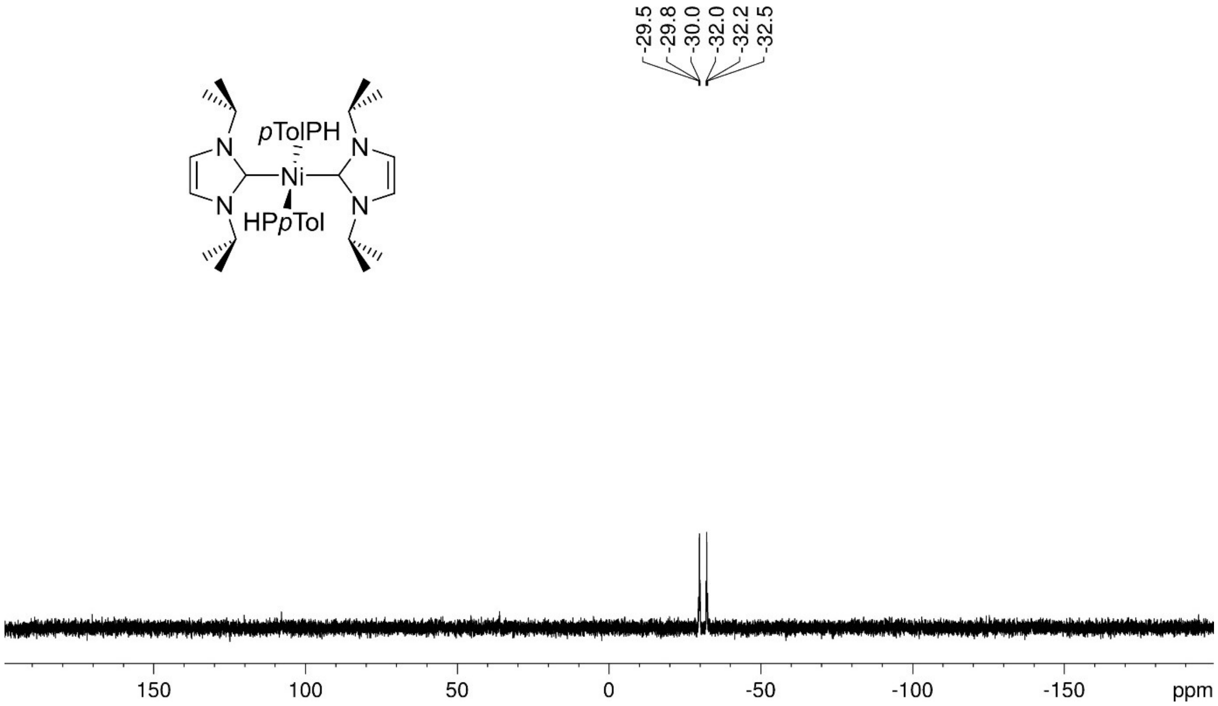

Figure S47. ^{31}P NMR spectrum (202 MHz, [D$_6$]benzene, 296 K) of *trans*-[Ni(I^{i}Pr$_2$)$_2${P(*para*-Tol)H}$_2$] (**17**).

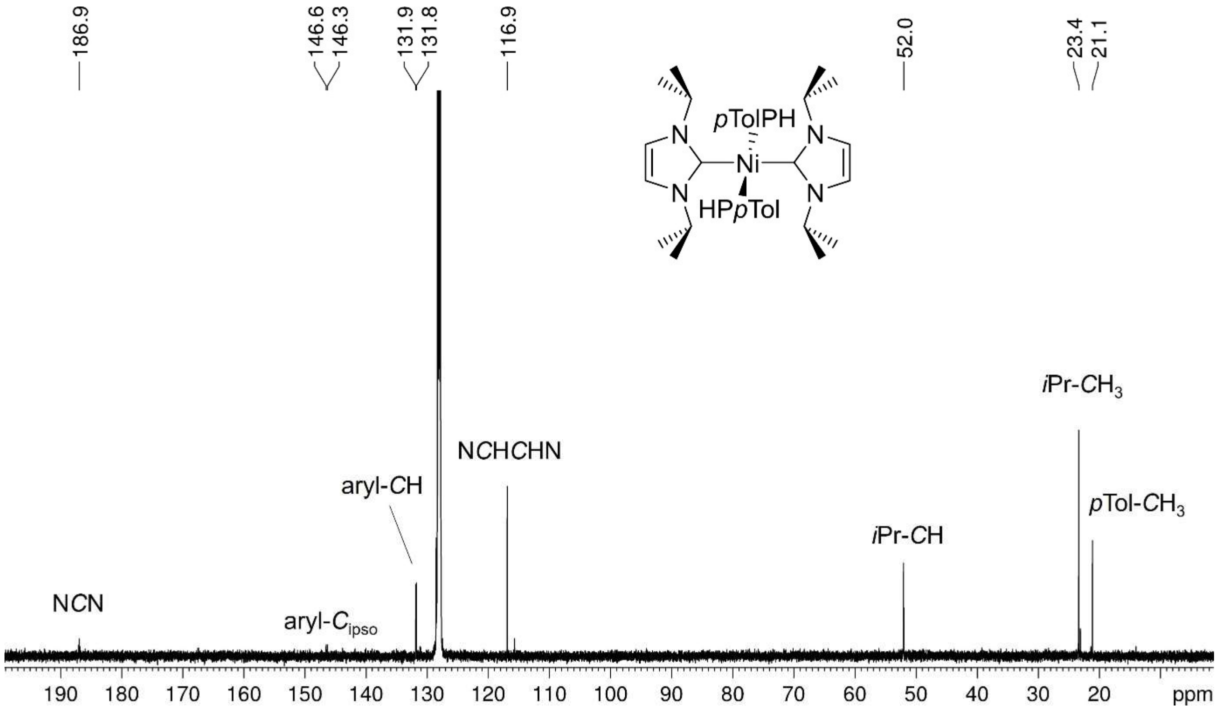

Figure S48. ^{13}C{^{1}H} NMR spectrum (126 MHz, [D$_6$]benzene, 296 K) of *trans*-[Ni(I^{i}Pr$_2$)$_2${P(*para*-Tol)H}$_2$] (**17**).

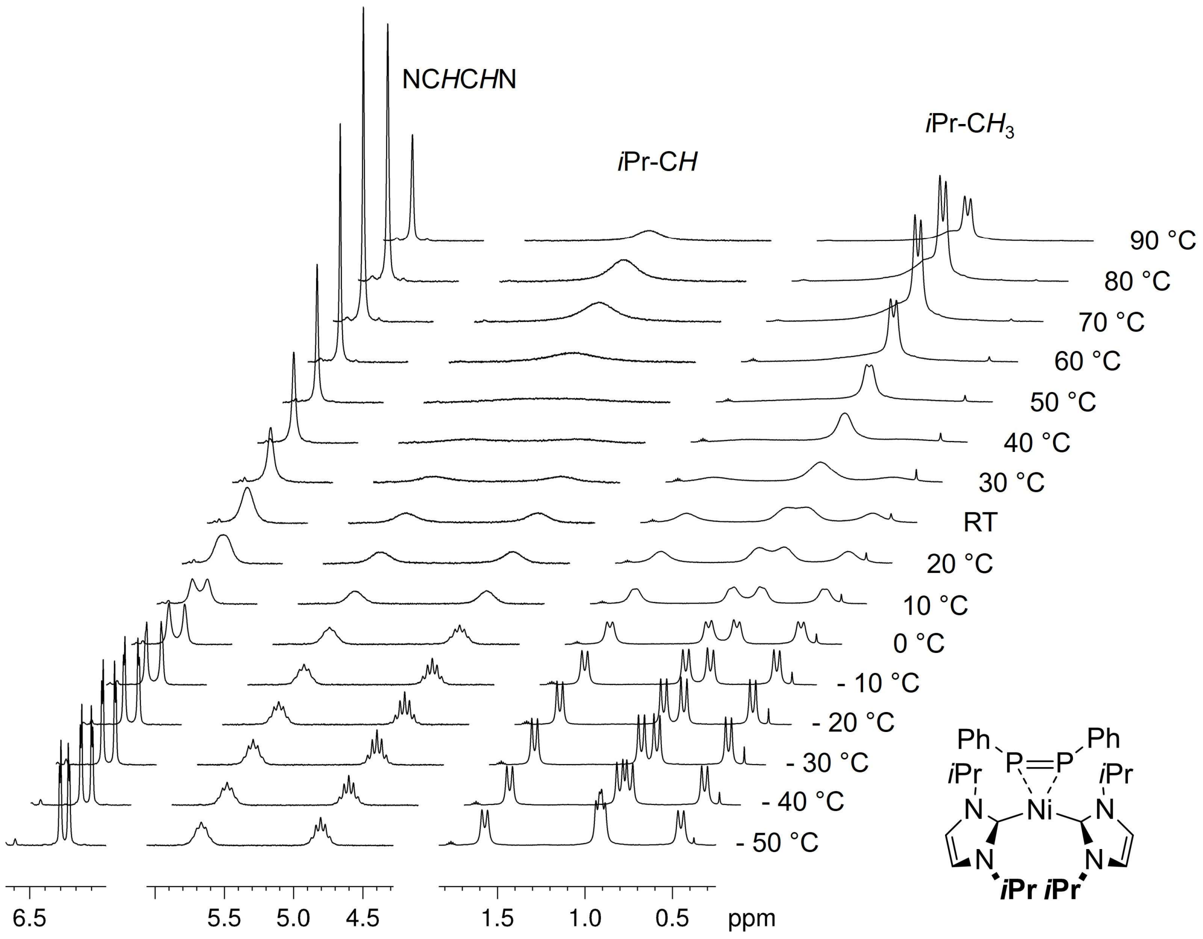

Figure S49. Variable temperature ^{1}H NMR spectra (200 MHz, [D$_6$]benzene) of *trans*-[Ni(I^{i}Pr$_2$)$_2$(η^2-PhP=PPh)] (**18**).

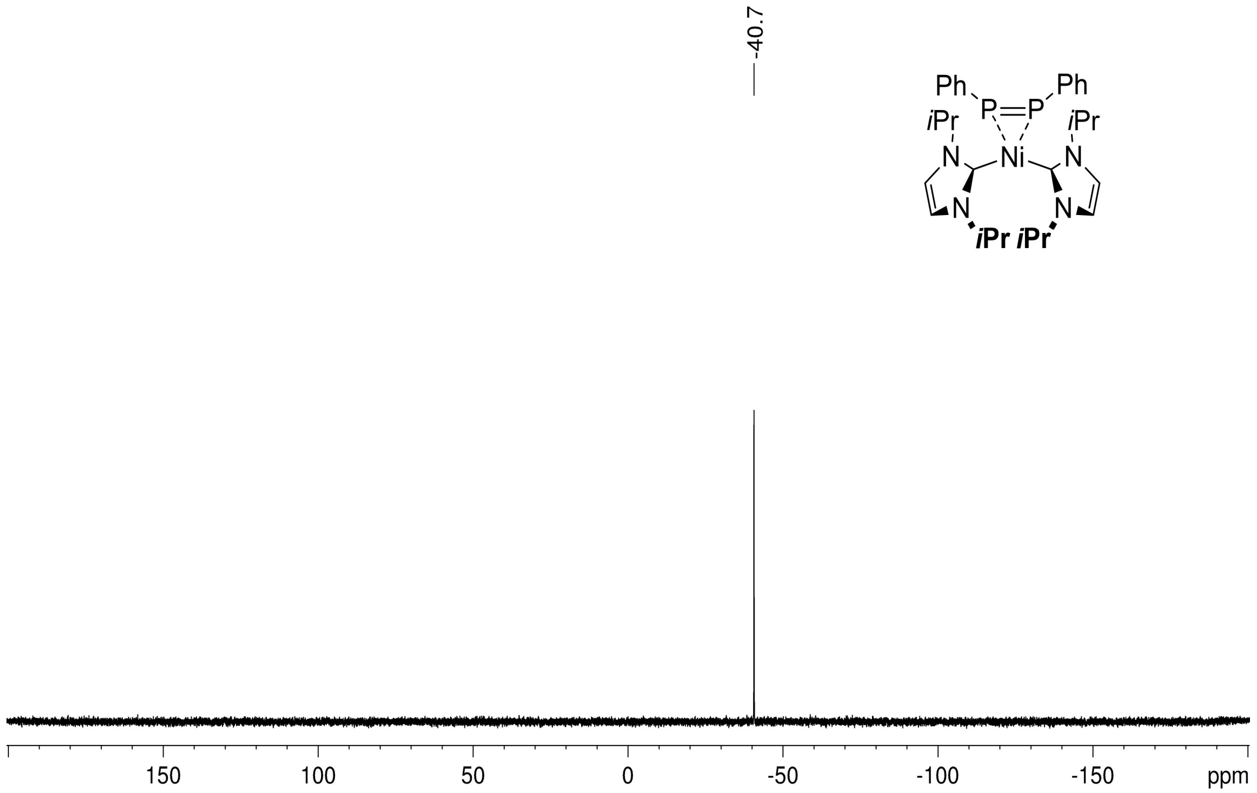

Figure S50. ^{31}P NMR spectrum (202 MHz, [D_6]benzene, 296 K) of *trans*-[Ni(I^iPr_2)$_2$(η^2-PhP=PPh)] (**18**).

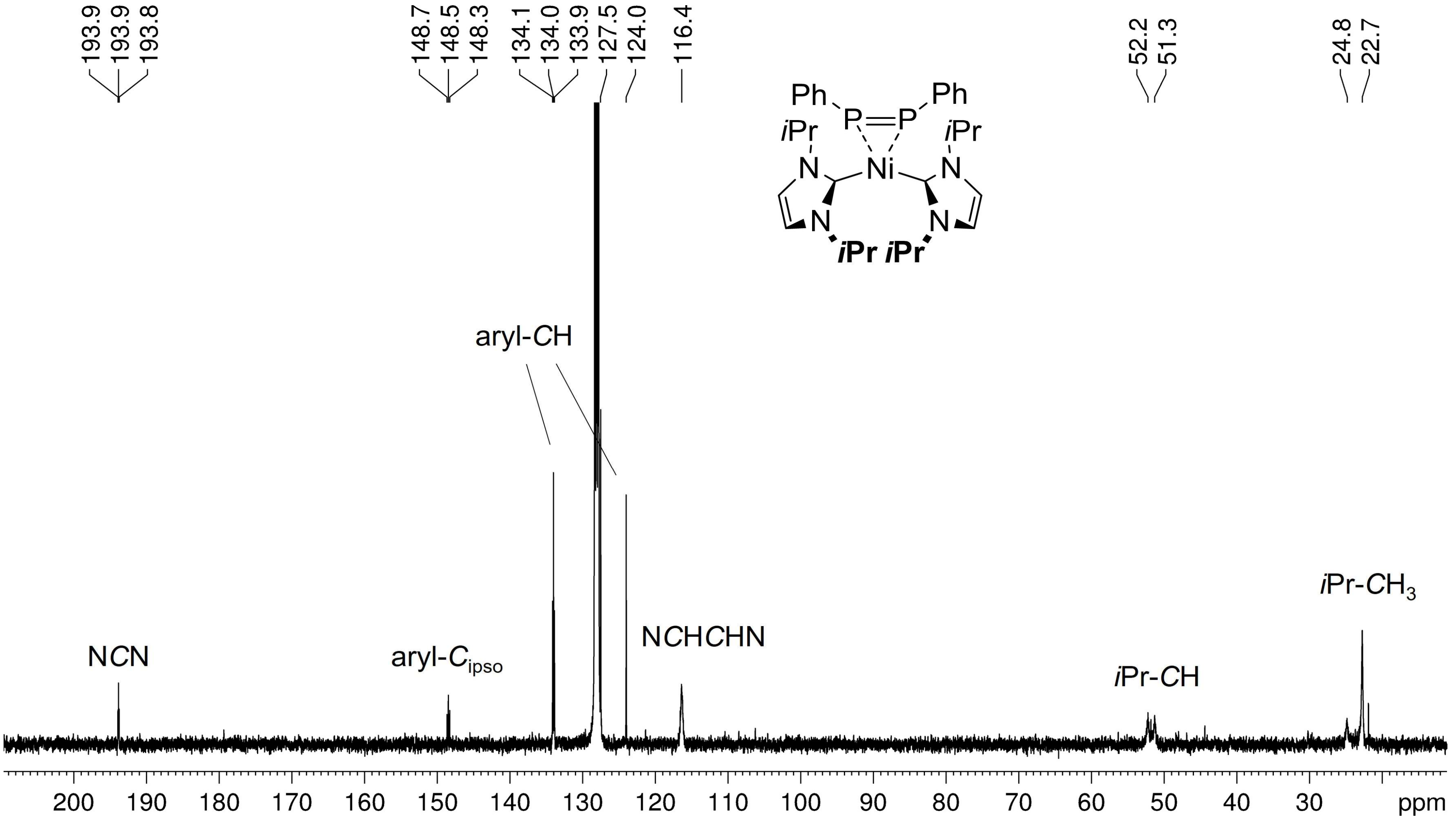

Figure S51. ^{13}C NMR spectrum (126 MHz, [D$_6$]benzene, 296 K) of *trans*-[Ni(I^{i}Pr$_2$)$_2$(η^2-PhP=PPh)] (**18**).

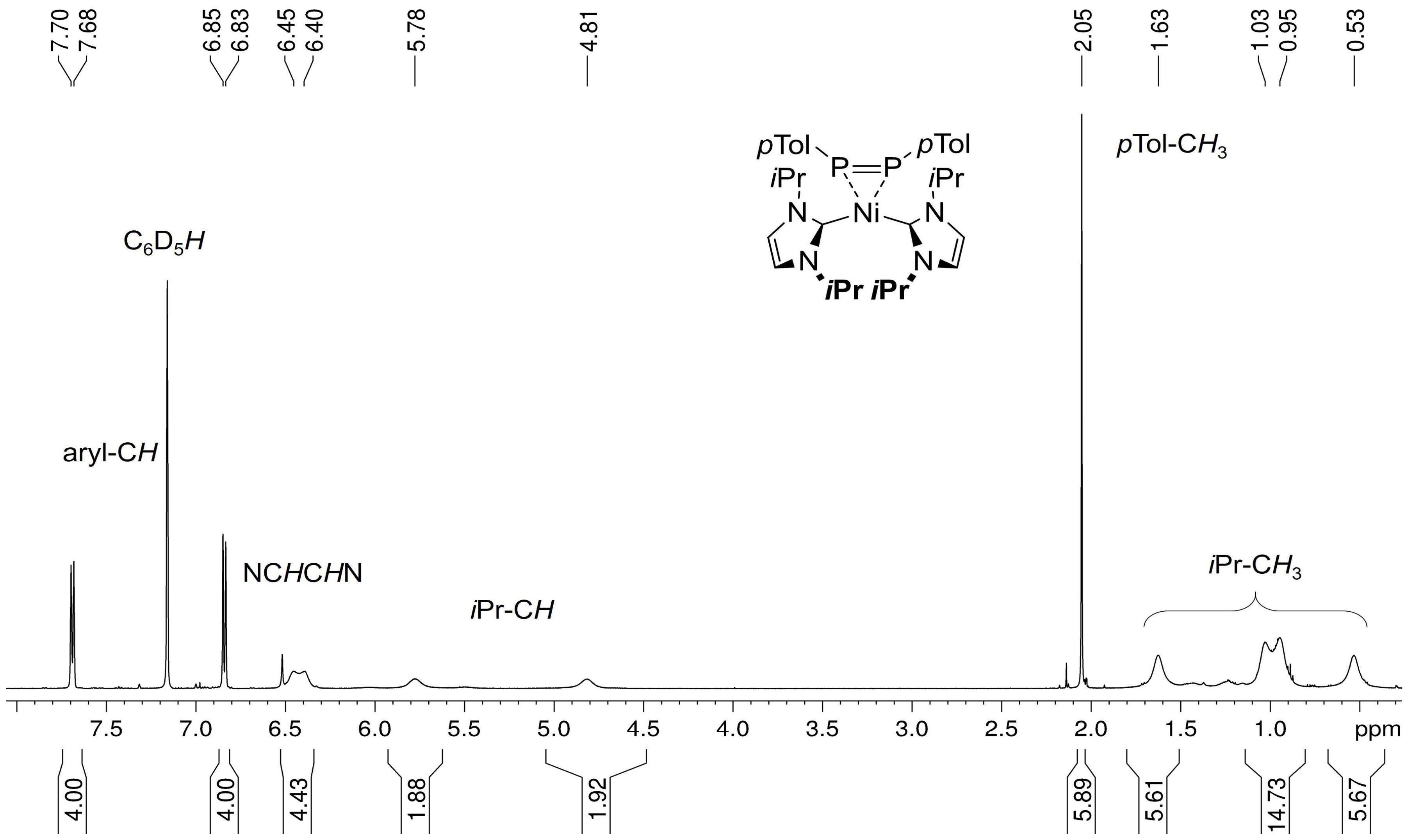

Figure S52. ^{1}H NMR spectrum (500 MHz, [D$_6$]benzene, 363 K) of *trans*-[Ni(I^{i}Pr$_2$)$_2$(η^2-{(*para*-Tol)P=P(*para*-Tol)}] (**19**).

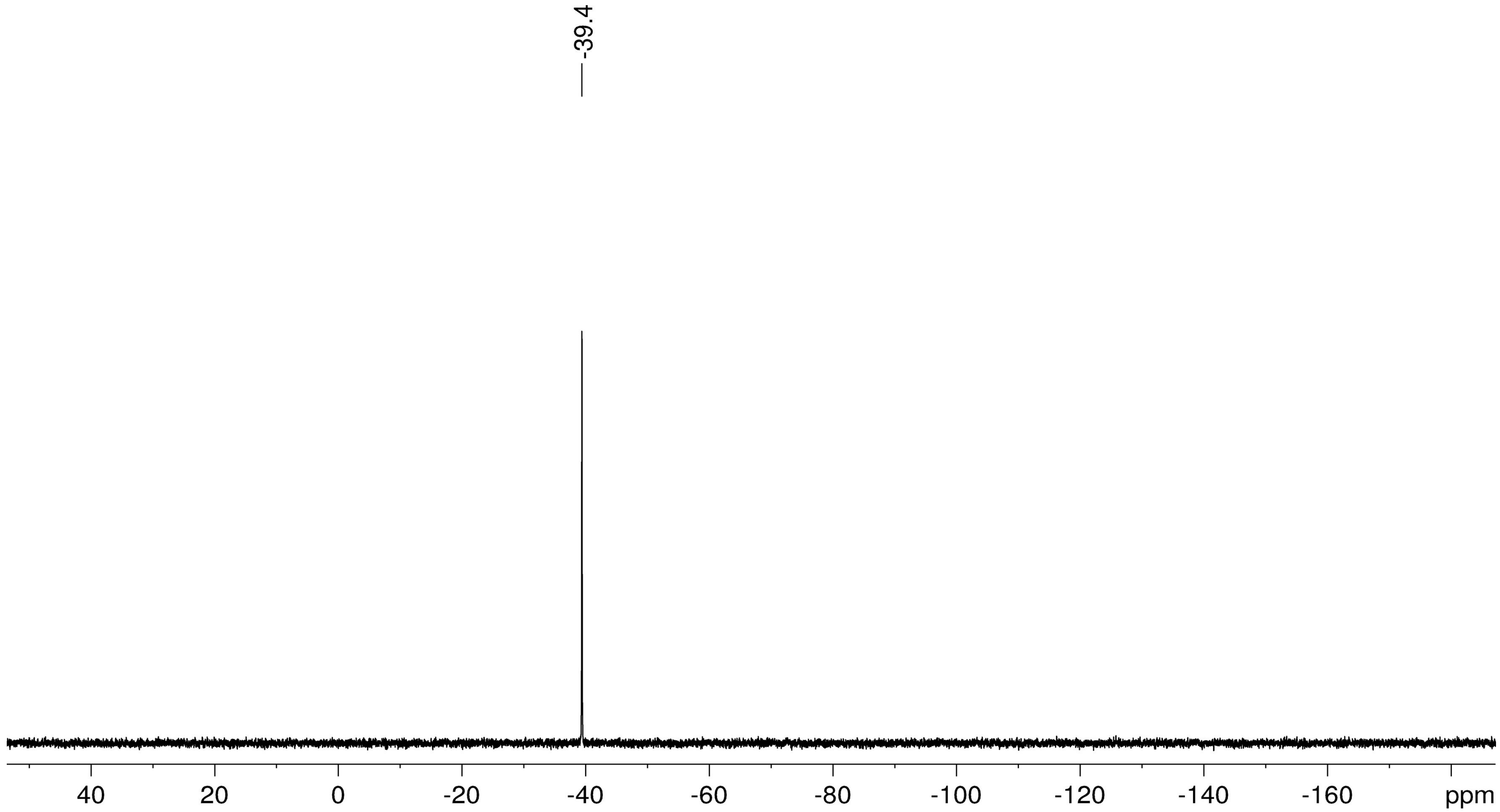

Figure S53. ^{31}P NMR spectrum (202 MHz, [D$_6$]benzene, 296 K) of *trans*-[Ni(I^{i}Pr$_2$)$_2$(η^2-{(*para*-Tol)P=P(*para*-Tol)}] (**19**).

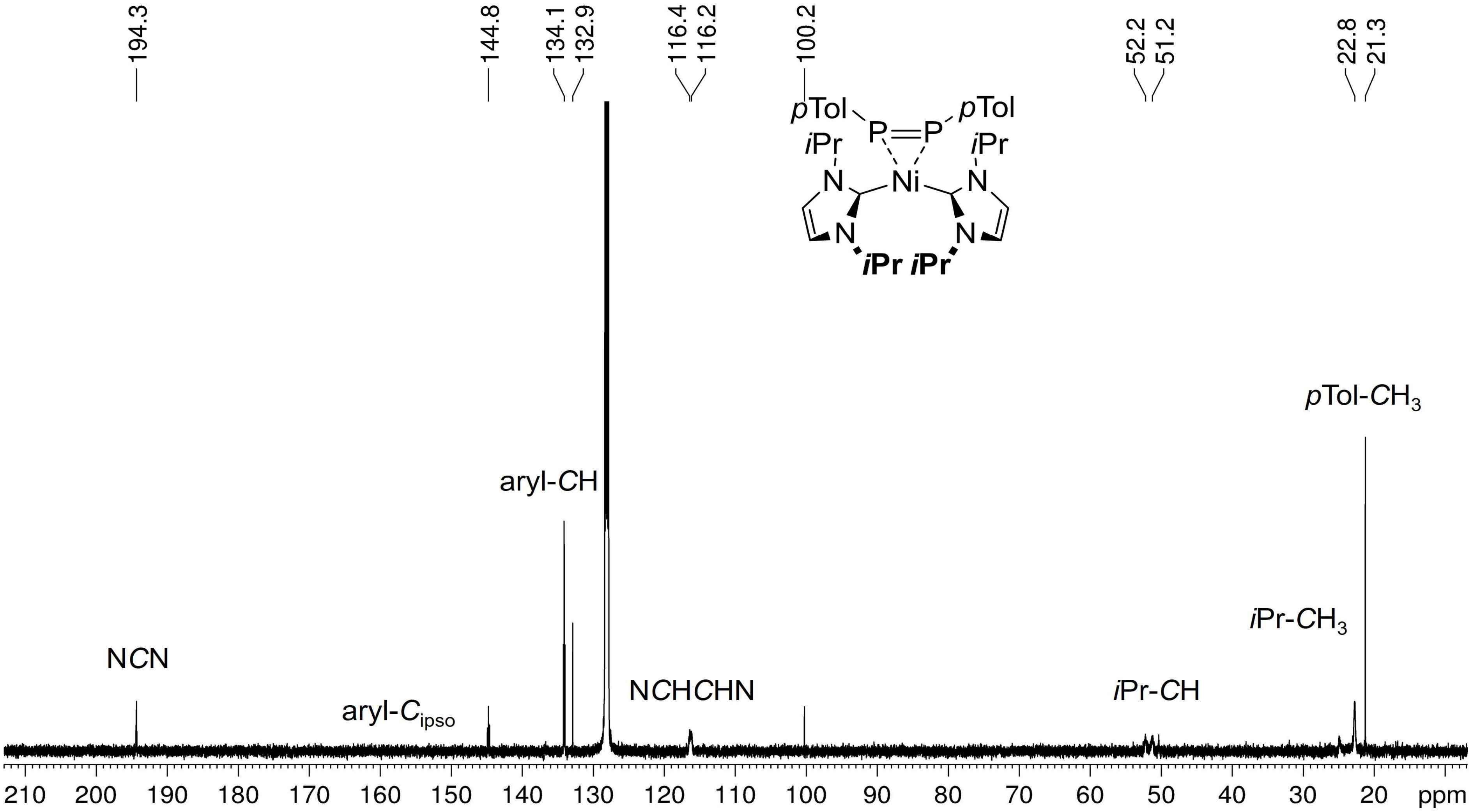

Figure S54. ^{13}C NMR spectrum (126 MHz, [D$_6$]benzene, 296 K) of *trans*-[Ni(I^{i}Pr$_2$)$_2$(η^2-{(*para*-Tol)P=P(*para*-Tol)}] (**19**).

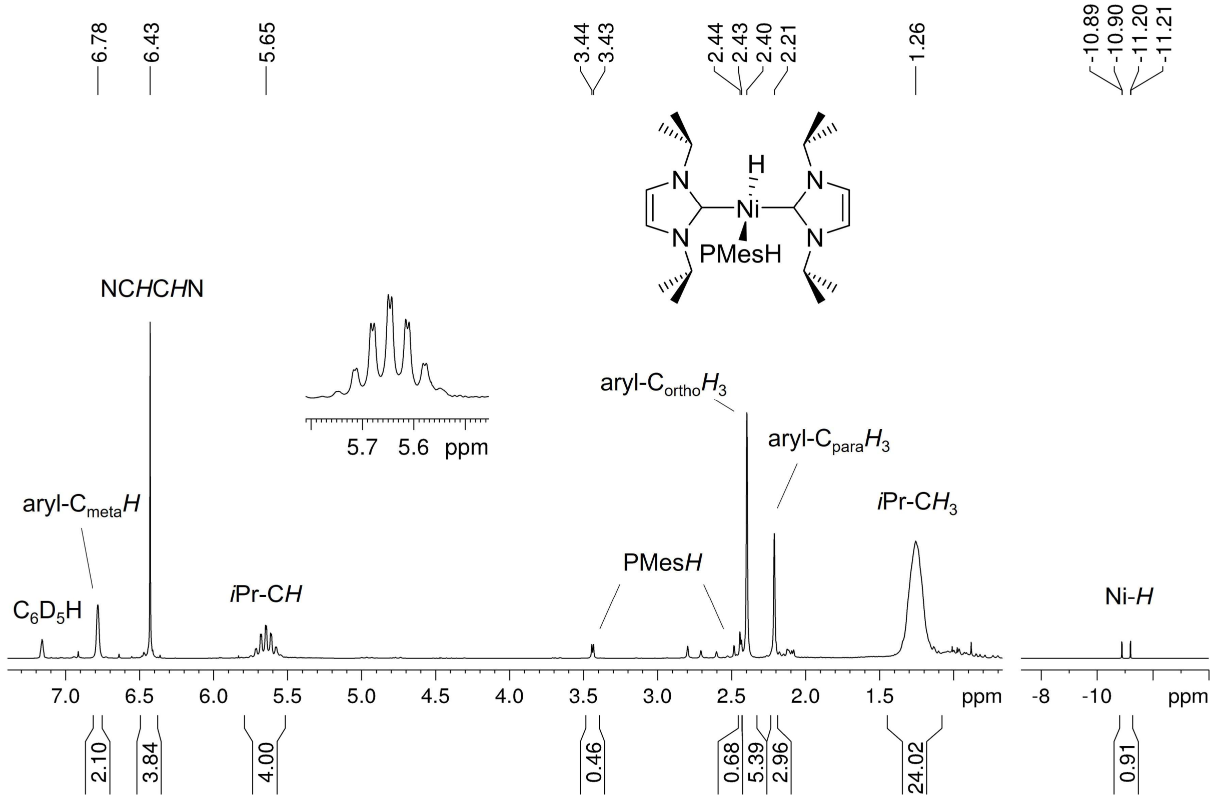

Figure S55. ^{1}H NMR spectrum (200 MHz, [D$_6$]benzene, 298 K) of *trans*-[Ni(I^{i}Pr$_2$)$_2$(PMesH)H] (**20**).

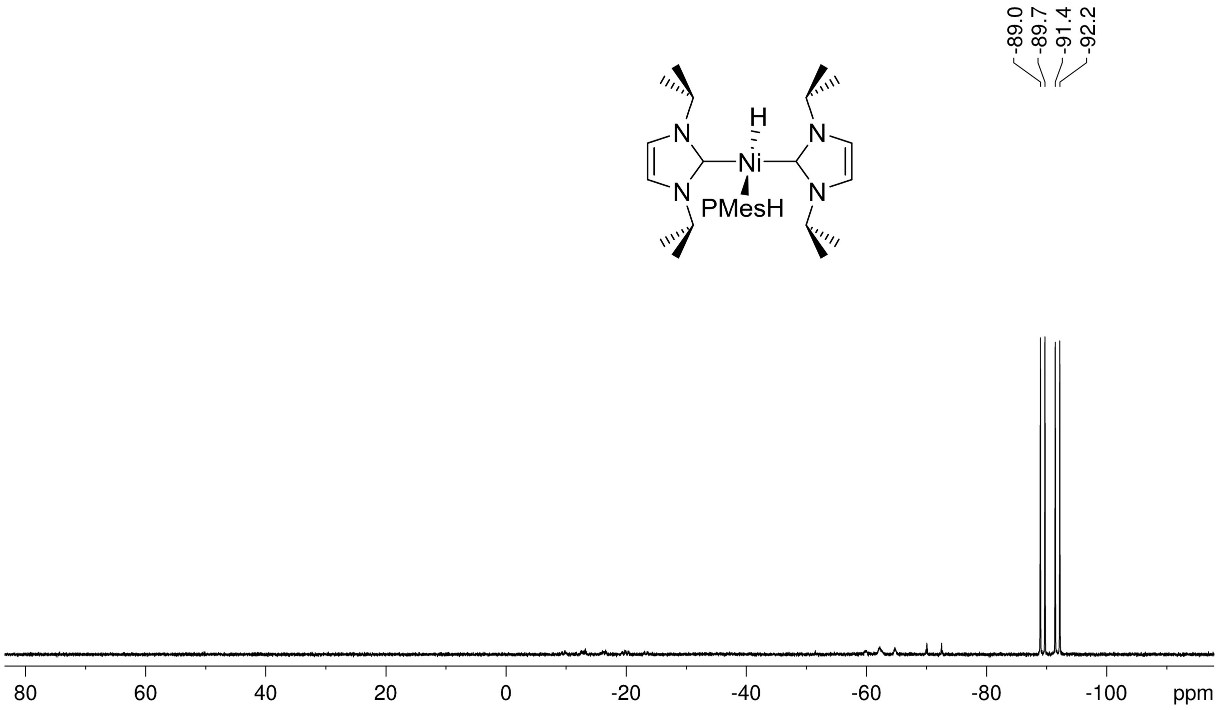

Figure S56. ^{31}P NMR spectrum (81 MHz, [D$_6$]benzene, 298 K) of *trans*-[Ni(I^{i}Pr$_2$)$_2$(PMesH)H] (**20**).

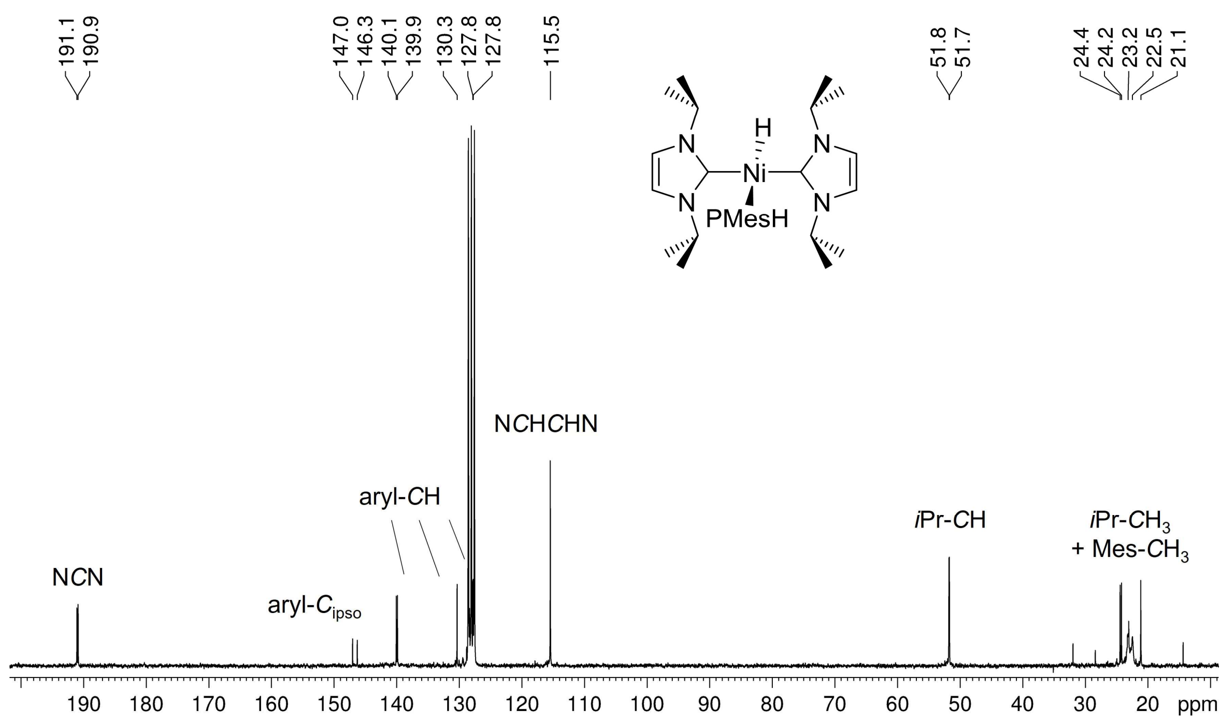

Figure S57. $^{13}C\{^1H\}$ NMR spectrum (50 MHz, [D$_6$]benzene, 298 K) of *trans*-[Ni(I^{i}Pr$_2$)$_2$(PMesH)H] (**20**).

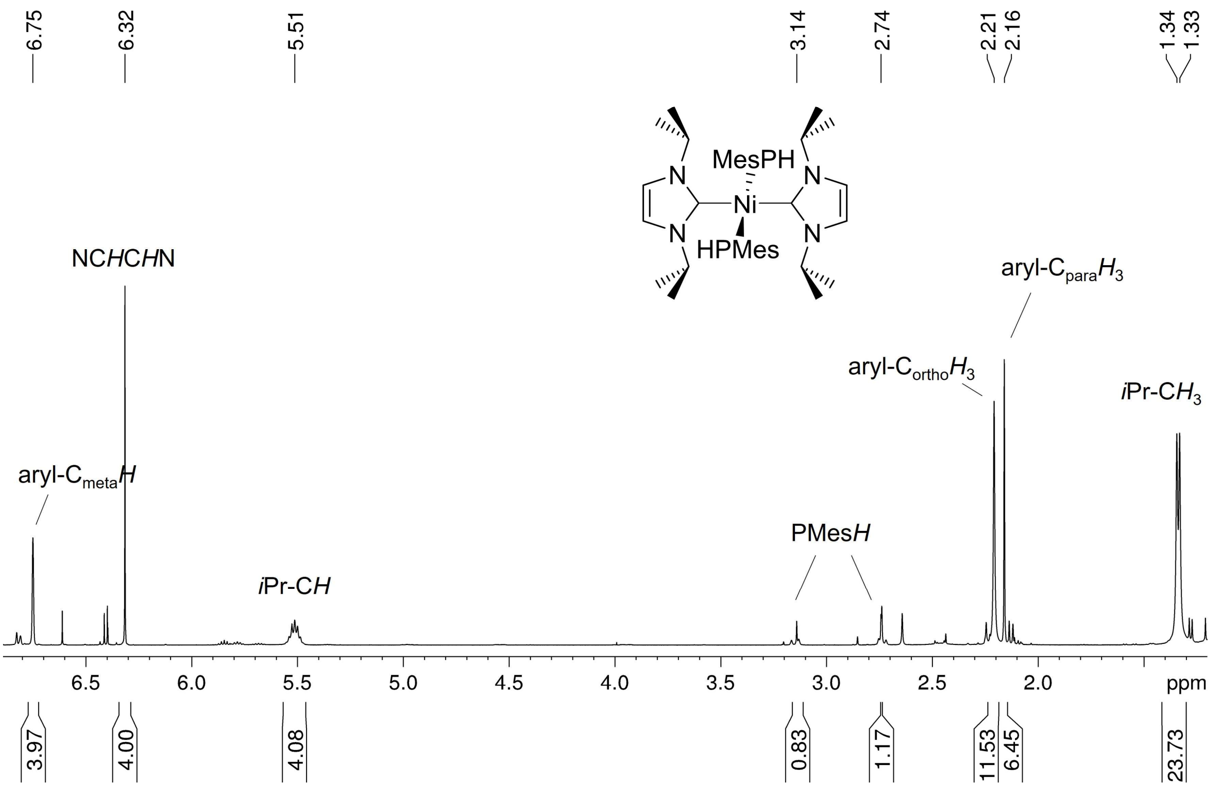

Figure S58. ^1H NMR spectrum (500 MHz, [D$_6$]benzene, 298 K) of *trans*-[Ni(I^{i}Pr$_2$)$_2$(PMesH)$_2$] (**21**).

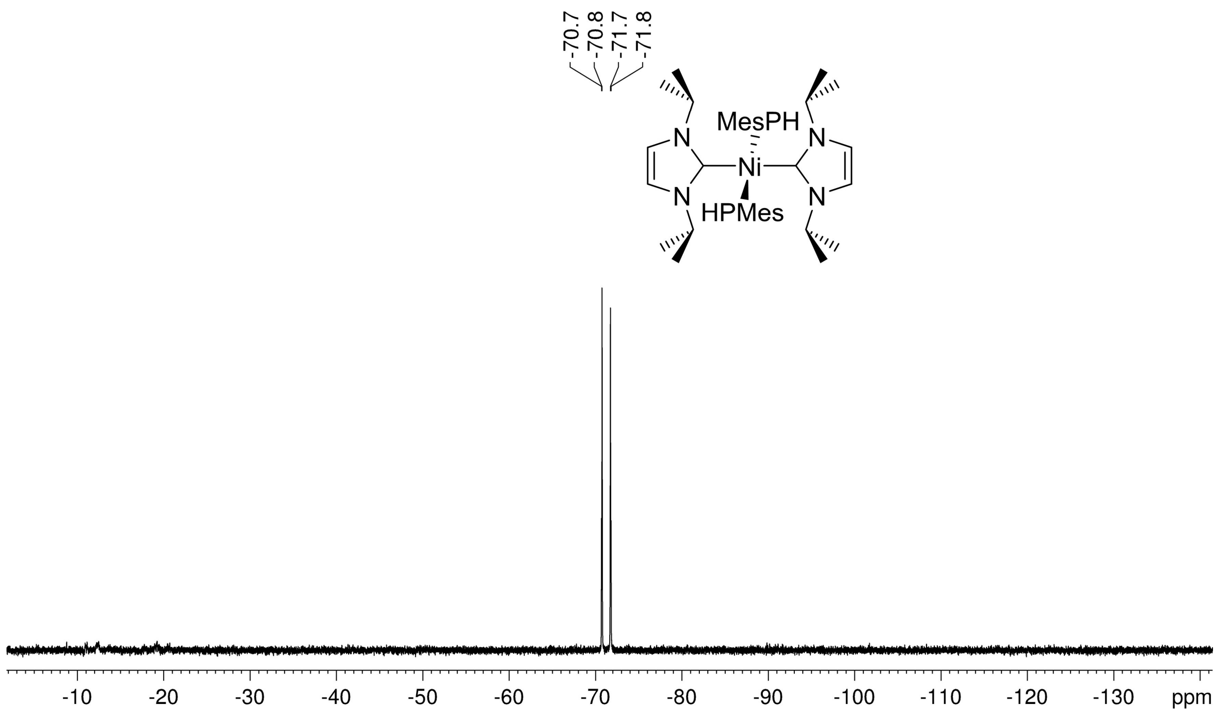

Figure S59. ^{31}P NMR spectrum (202 MHz, [D$_6$]benzene, 298 K) of *trans*-[Ni(I^{i}Pr$_2$)$_2$(PMesH)$_2$] (**21**).

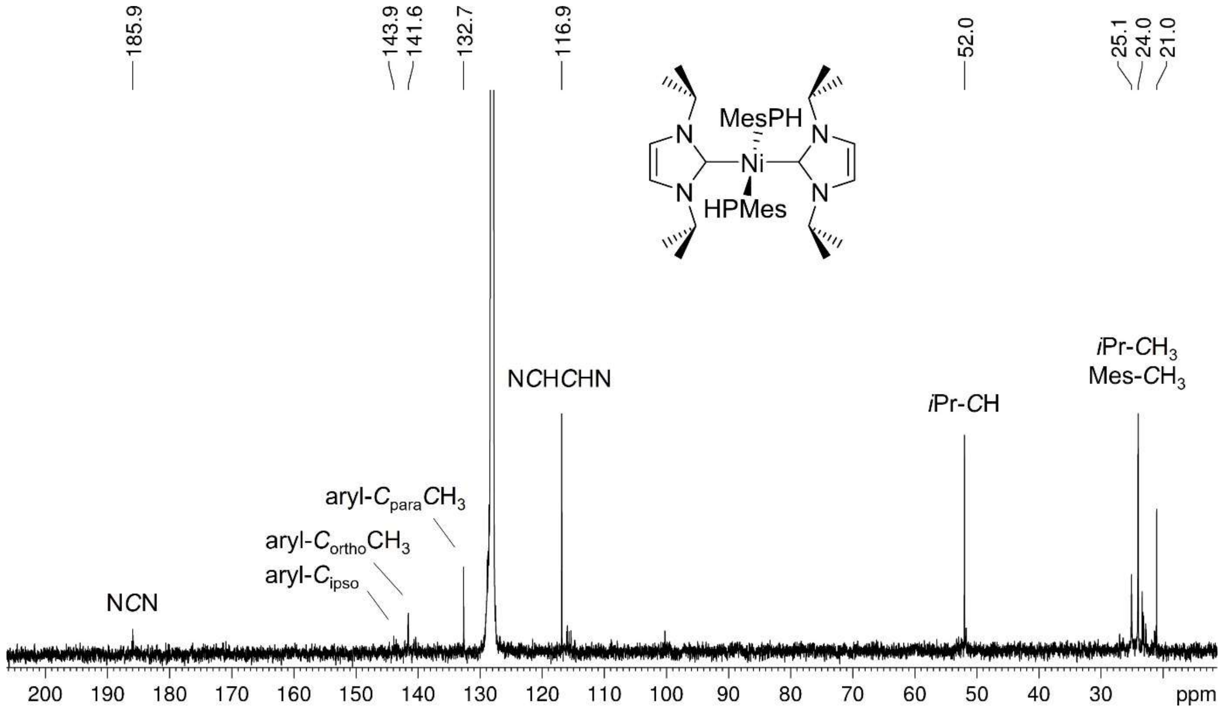

Figure S60. ^{13}C{^{1}H} NMR spectrum (126 MHz, [D$_6$]benzene, 298 K) of *trans*-[Ni(I^{i}Pr$_2$)$_2$(PMesH)$_2$] (**21**).

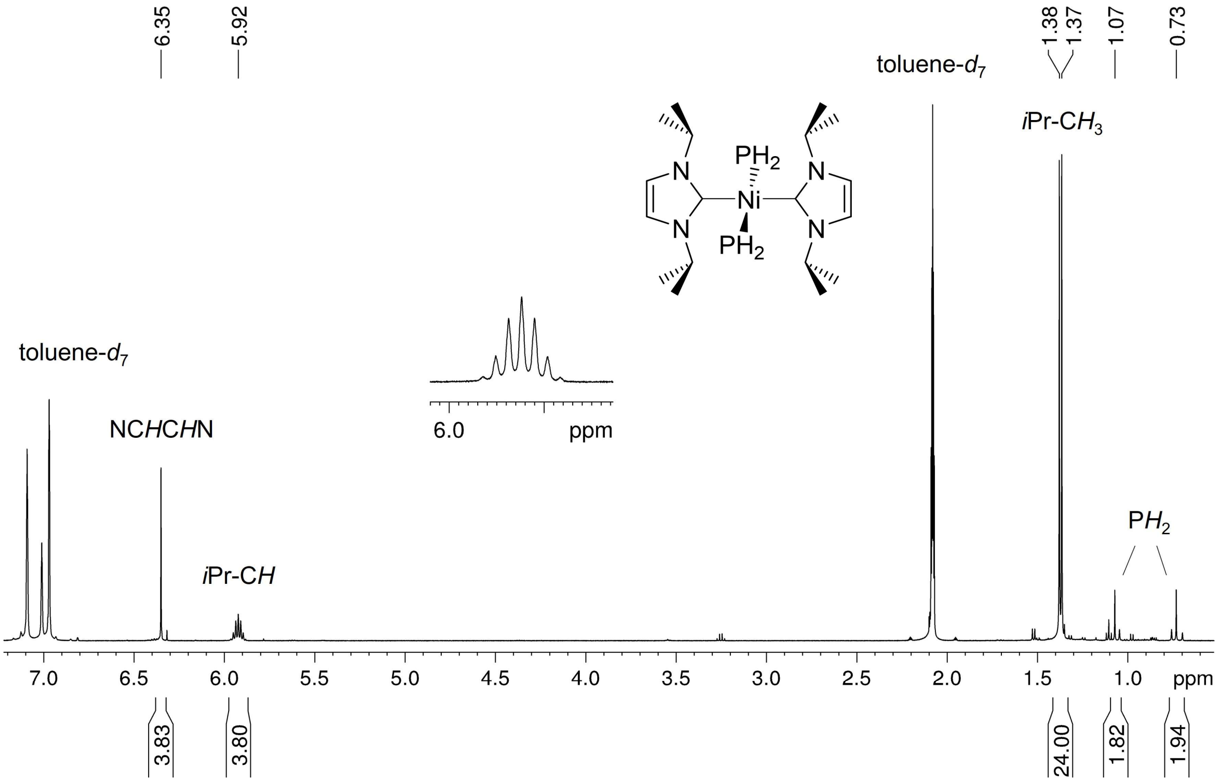

Figure S61. ^1H NMR spectrum (500 MHz, [D$_6$]benzene, 296 K) of *trans*-[Ni(I^{i}Pr$_2$)$_2$(PH$_2$)$_2$] (**22**).

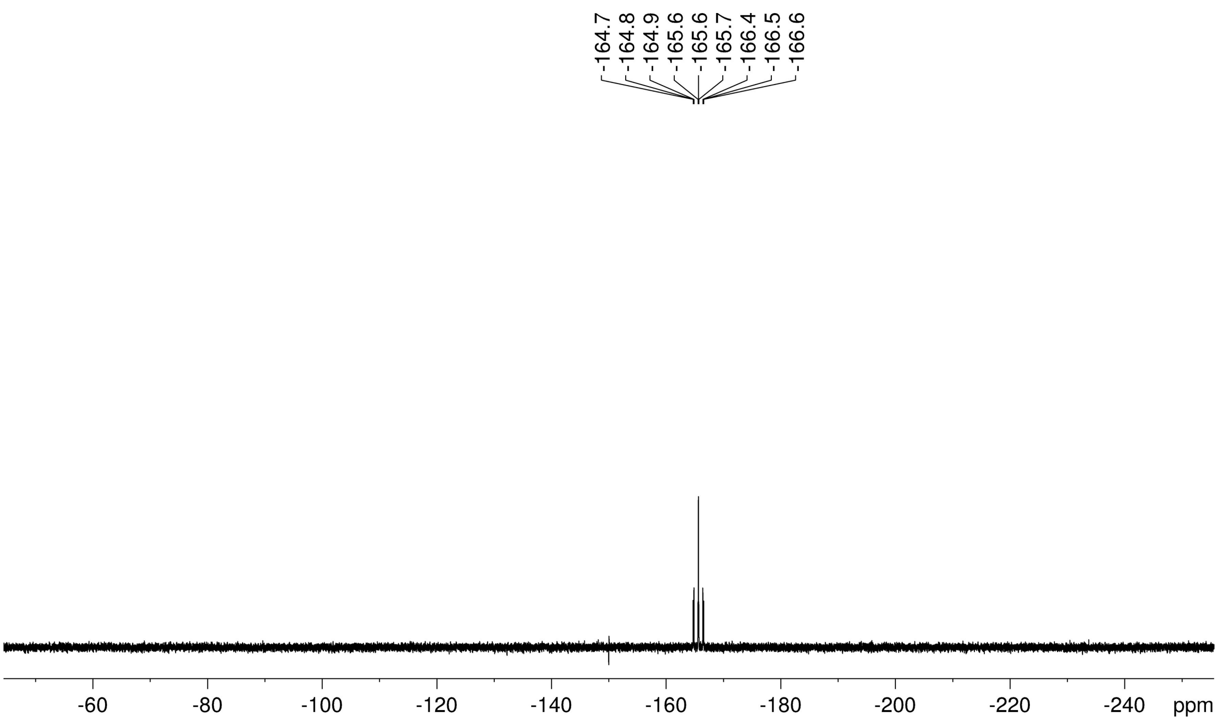

Figure S62. ^{31}P NMR spectrum (202 MHz, [D$_6$]benzene, 296 K) of *trans*-[Ni(I^{i}Pr$_2$)$_2$(PH$_2$)$_2$] (**22**).

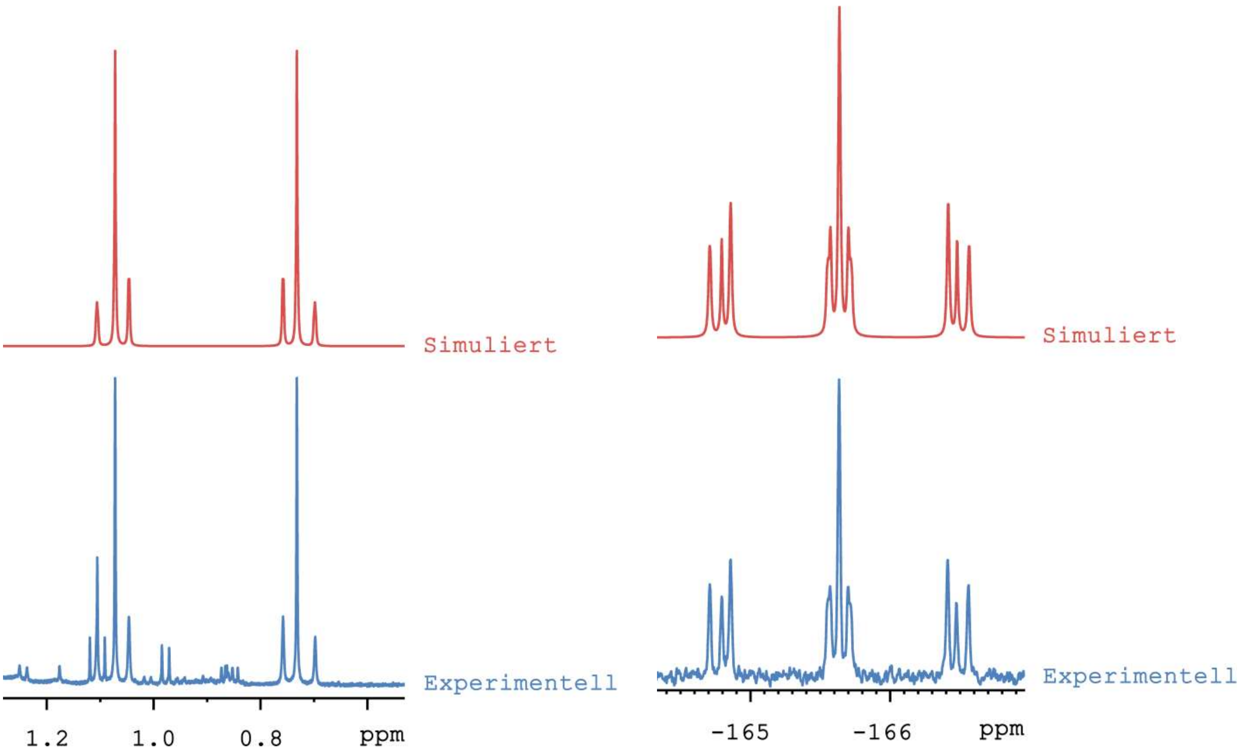

Figure S63. Experimental (C_6D_6, blue) and simulated (red) ^1H (left) and ^{31}P (right) NMR spectra of *trans*-[Ni(I^{i}Pr$_2$)$_2$(PH$_2$)$_2$] **22**.

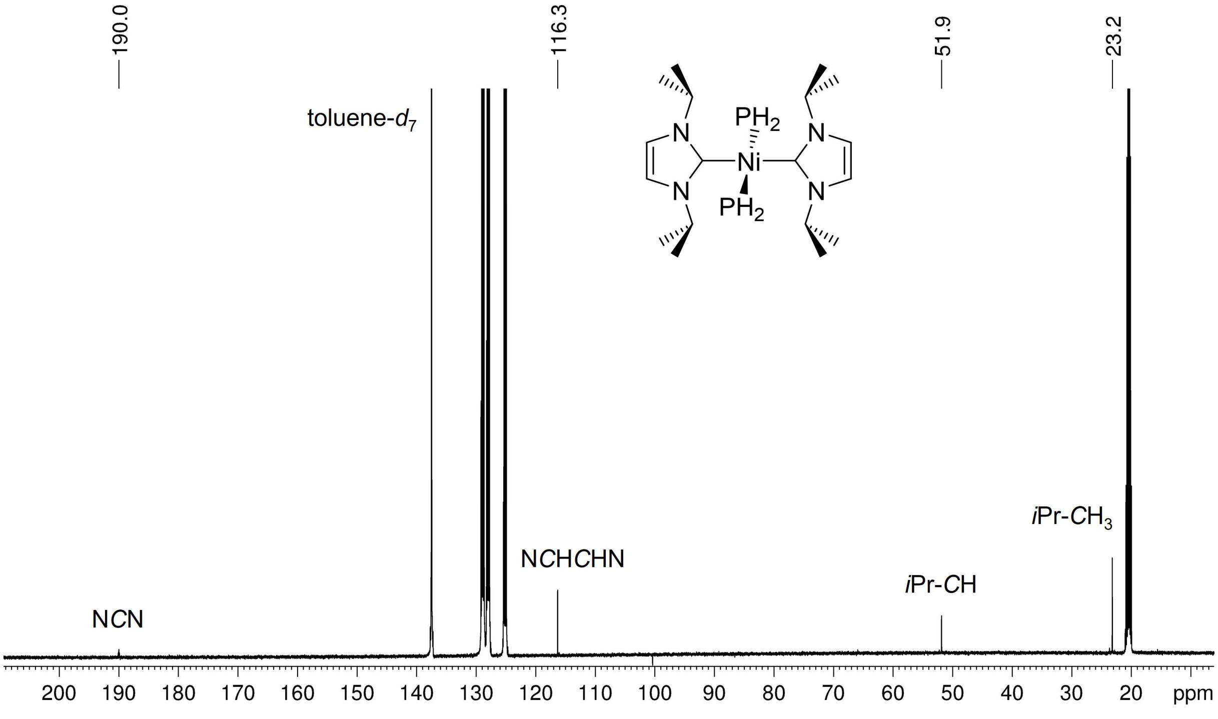

Figure S64. $^{13}C\{^1H\}$ NMR spectrum (126 MHz, [D$_6$]benzene, 296 K) of *trans*-[Ni(I^{i}Pr$_2$)$_2$(PH$_2$)$_2$] (**22**).

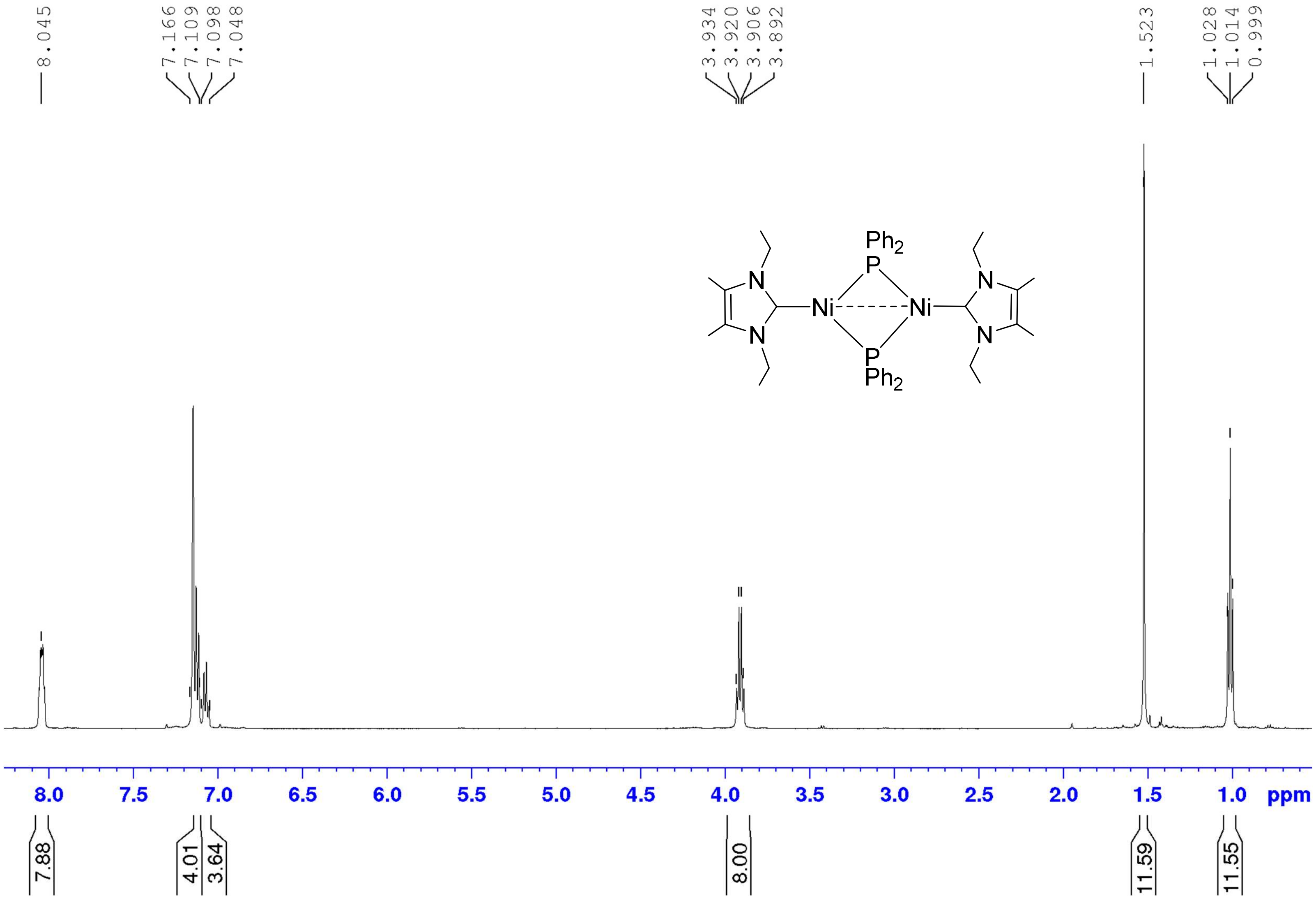

Figure S65. ^{1}H NMR spectrum (500 MHz, [D$_6$]benzene, 298 K) of $[Ni(IEt_2Me_2)]_2(\mu\text{-}PPh_2)_2$. Incorrect integration of aryl protons results from partial overlap with C_6D_5H resonance.

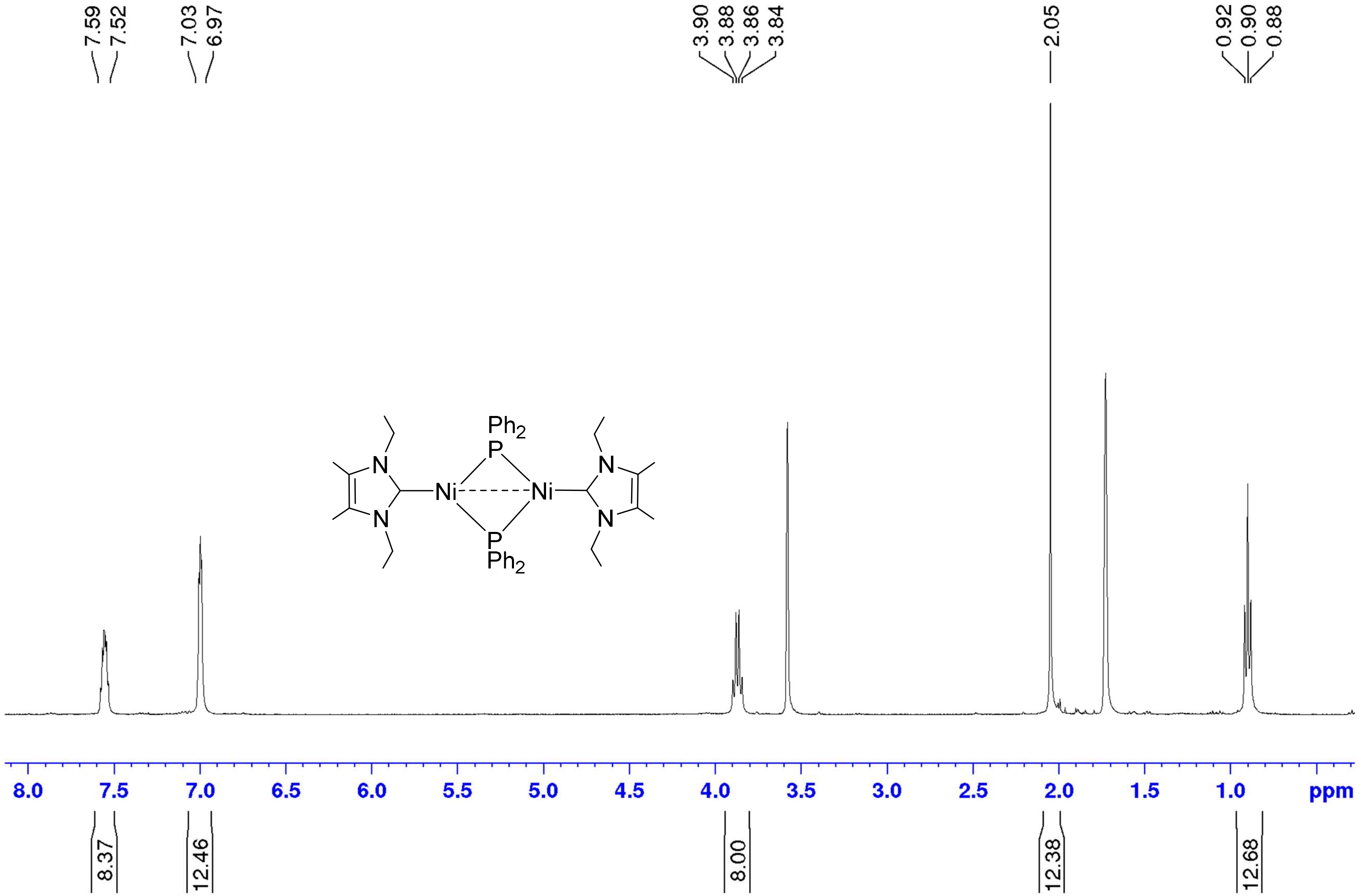

Figure S66. ^{1}H NMR spectrum (500 MHz, [D_8]THF, 298 K) of [Ni(IEt_2Me_2)]$_2$(μ-PPh_2)$_2$.

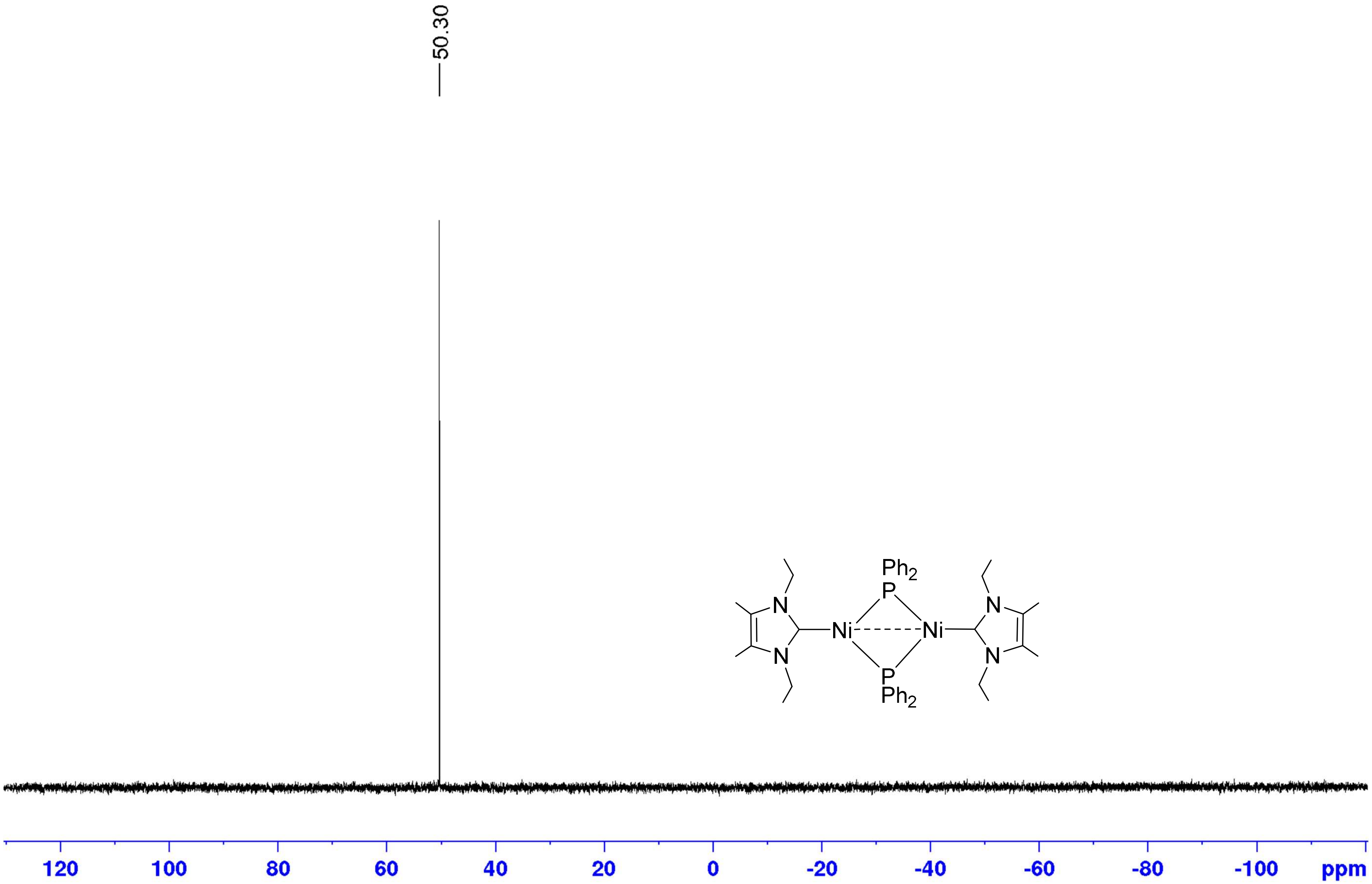

Figure S67. $^{31}P\{^{1}H\}$ NMR spectrum (162 MHz, [D$_6$]benzene, 298 K) of [Ni(IEt$_2$Me$_2$)]$_2$(μ-PPh$_2$)$_2$.

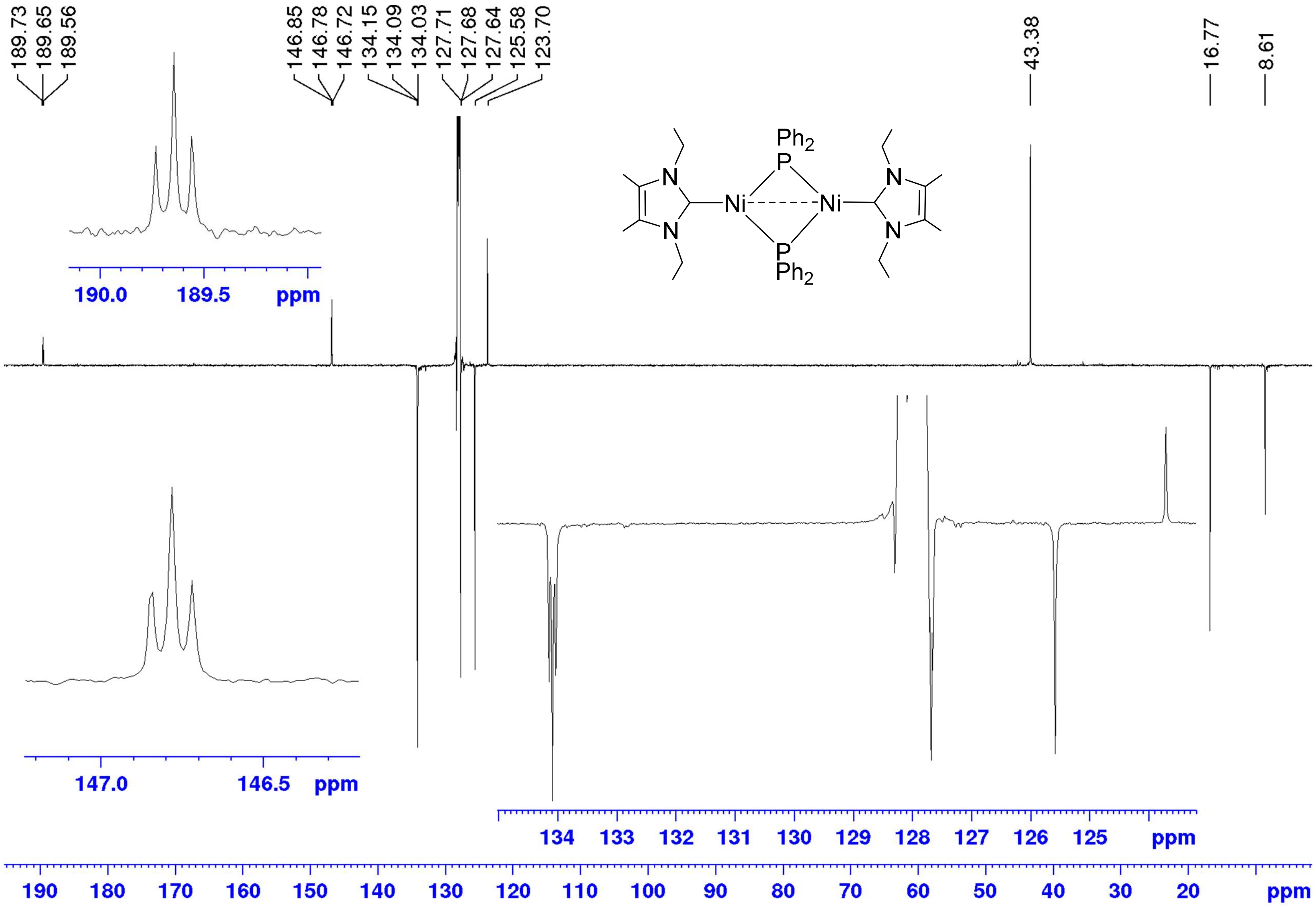

Figure S68. $^{13}C\{^{1}H\}$ PENDANT NMR spectrum (126 MHz, [D$_6$]benzene, 298 K) of $[Ni(IEt_2Me_2)]_2(\mu\text{-}PPh_2)_2$.

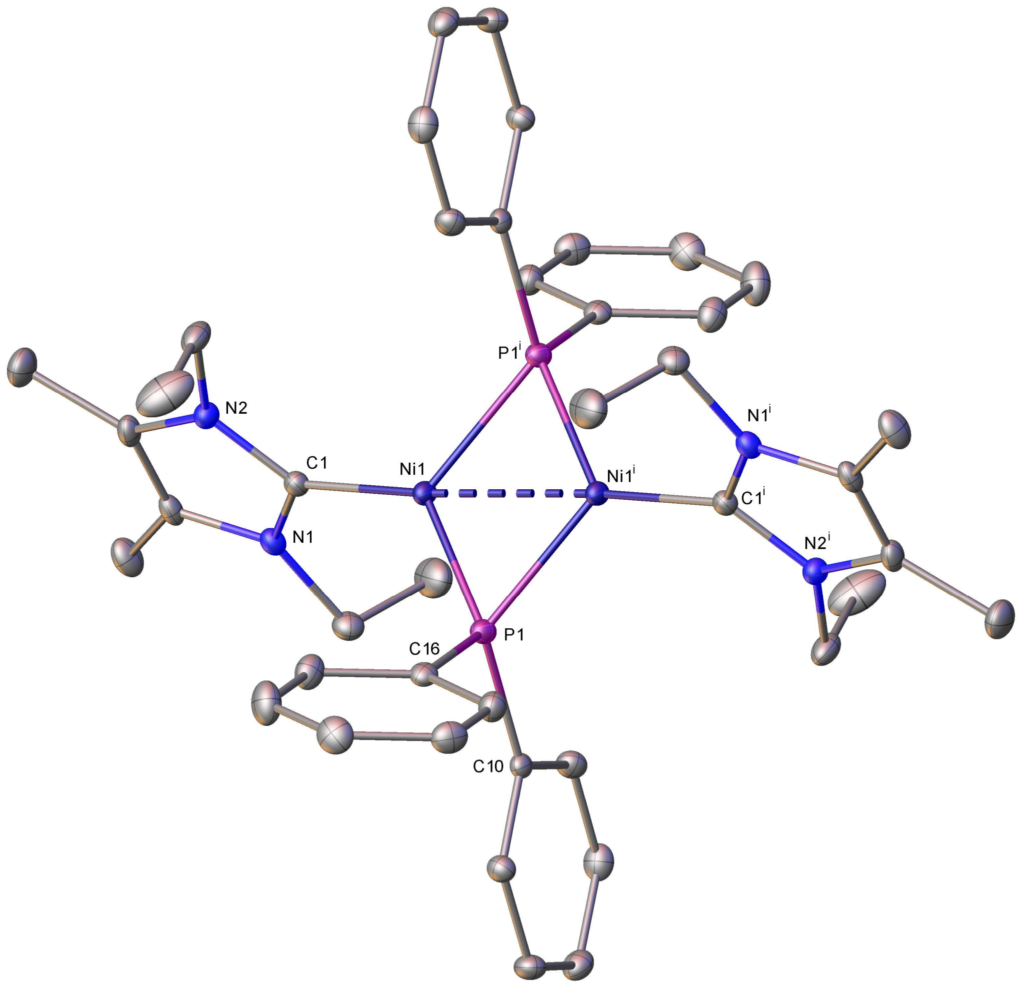

Figure S69. Molecular structure of $[Ni(IEt_2Me_2)]_2(\mu\text{-}PPh_2)_2$. Ellipsoids are shown at 30% probability and all hydrogens are removed for clarity.

Table S1. Crystal data and structural refinement details for compounds **2**, **3**, **4**, **6**, **7**, **11**, **16-19**, **21** and **22**.

Identification code	**2**	**3**	**4**	**6**	7	**11**
Empirical formula	$C_{36}H_{47}N_4PNi$	$C_{30}H_{43}N_4NiP$	$C_{30}H_{43}N_4NiP$	$C_{38}H_{44}N_4NiP_2$	$C_{42}H_{52}N_4NiP_2$	$C_{26}H_{36}N_4NiP_2$
Formula weight	625.45	549.36	549.36	677.42	733.52	525.24
Temperature/ K	150.00(10)	173(2)	150.00(10)	150.00(10)	150.00(10)	150.00(10)
Crystal system	monoclinic	orthorhombic	triclinic	monoclinic	monoclinic	monoclinic
Space group	$P2_1$	$P2_12_12_1$	$P{-}1$	$P2_1/n$	$P2_1/c$	$P2_1/c$
a/ Å	9.4511(2)	11.107(2)	12.2005(4)	9.1133(4)	9.7318(2)	9.61209(11)
b/ Å	17.6908(5)	15.709(3)	12.2420(4)	18.0555(7)	11.9332(3)	9.74476(12)
c/ Å	10.3105(3)	17.284(4)	13.0317(4)	10.8006(4)	16.3381(4)	14.79785(19)
α/ °	90	90.00	117.254(3)	90	90	90
β/ °	103.001(3)	90.00	116.915(3)	104.855(4)	90.457(2)	102.9540(12)
γ/ °	90	90.00	90.114(2)	90	90	90
U/ Å^3	1679.70(8)	3015.8(10)	1488.14(10)	1717.78(12)	1897.31(8)	1350.80(3)
Z	2	4	2	2	2	2
ρ_{calc}/ g cm^{-3}	1.237	1.210	1.226	1.310	1.284	1.291
μ/ mm^{-1}	0.655	0.720	0.730	0.691	0.631	2.306
F(000)	668.0	1176.0	588.0	716.0	780.0	556.0
Crystal size/ mm^3	0.364 × 0.178 × 0.072	0.33 × 0.25 × 0.12	0.584 × 0.382 × 0.279	0.354 × 0.344 × 0.295	0.573 × 0.408 × 0.31	0.205 × 0.192 × 0.15
2θ range for data collection/°	6.64 to 54.964	3.5 to 56.12	6.928 to 59.078	6.774 to 54.962	7.27 to 60.586	9.442 to 146.892
Index ranges	$-12 \le h \le 12$, $-18 \le k \le 22$, $-13 \le l \le 13$	$-14 \le h \le 14$, $-20 \le k \le 20$, $-22 \le l \le 22$	$-15 \le h \le 16$, $-13 \le k \le 16$, $-17 \le l \le 16$	$-11 \le h \le 11$, $-23 \le k \le 23$, $-13 \le l \le 12$	$-13 \le h \le 12$, $-15 \le k \le 16$, $-21 \le l \le 22$	$-7 \le h \le 11$, $-11 \le k \le 12$, $-18 \le l \le 18$
Reflections collected	14602	62384	13223	15115	16797	13502
Independent reflections, R_{int}	6553, 0.0268	7320, 0.0482	6883, 0.0145	3880, 0.0331	5052, 0.0248	2696, 0.0250
Data/restraints/parameters	6553/1/387	7320/0/337	6883/0/337	3880/0/209	5052/0/327	2696/0/159
Goodness–of–fit on F^2	1.031	1.065	1.034	1.039	1.045	1.077
Final $R1$, $wR2$ [I>=2σ(I)]	0.0299, 0.0623	0.0268, 0.0689	0.0271, 0.0688	0.0344, 0.0764	0.0310, 0.0700	0.0265, 0.0705
Final $R1$, $wR2$ [all data]	0.0332, 0.0636	0.0290, 0.0703	0.0324, 0.0716	0.0514, 0.0828	0.0403, 0.0741	0.0277, 0.0712
Largest diff. peak/hole/ e Å^{-3}	0.29/−0.24	0.27/−0.23	0.34/−0.18	0.37/−0.24	0.32/−0.22	0.27/−0.22
Flack Parameter	−0.002(6)	0.027(4)				

Identification code	**16**	**17**	**18**	**19**	**21**	**22**
Empirical formula	$C_{30}H_{44}N_4NiP_2$	$C_{32}H_{48}N_4NiP_2$	$C_{30}H_{42}N_4NiP_2$	$C_{38}H_{52}N_4NiP_2$	$C_{45}H_{65}N_4NiP_2$	$C_{18}H_{36}N_4NiP_2$
Formula weight	581.34	609.39	579.32	685.48	782.66	429.16
Temperature/ K	173(2)	100(2)	173(2)	100(2)	173(2)	173(2)
Crystal system	orthorhombic	monoclinic	triclinic	monoclinic	orthorhombic	monoclinic
Space group	*Pbca*	$P2_1/c$	$P-1$	$C2/c$	*Pbca*	$C2/c$
a/ Å	15.791(3)	10.2285(9)	9.7264(19)	13.7093(9)	14.4297(14)	31.944(6)
b/ Å	17.623(4)	9.4065(8)	9.861(2)	14.9111(10)	21.881(2)	9.0818(18)
c/ Å	22.122(4)	17.5961(15)	17.821(4)	19.6501(13)	29.015(3)	17.252(4)
α/ °	90	90	77.25(3)	90	90	90
β/ °	90	99.068(3)	87.58(3)	107.382(2)	90	111.38(3)
γ/ °	90	90	67.65(3)	90	90	90
U/ Å^3	6157(2)	1671.8(2)	1540.3(6)	3833.5(4)	9161.0(16)	4660.4(18)
Z	8	2	2	4	8	8
ρ_{calc}/ g cm^{-3}	1.254	1.211	1.249	1.188	1.135	1.223
μ/ mm^{-1}	0.759	0.702	0.758	0.620	0.526	0.978
F(000)	2480.0	652.0	616.0	1464.0	3368.0	1840.0
Crystal size/ mm^3	0.23 × 0.09 × 0.08	0.03 × 0.02 × 0.02	0.29 × 0.23 × 0.13	0.38 × 0.30 × 0.25	0.23 × 0.15 × 0.12	0.29 × 0.28 × 0.02
2θ range for data collection/°	3.682 to 52.042	4.032 to 52.044	4.532 to 52.29	4.142 to 52.12	3.66 to 52.348	2.738 to 52.182
Index ranges	$-19 \le h \le 19$, $0 \le k \le 21$, $0 \le l \le 27$	$-12 \le h \le 12$, $-11 \le k \le 11$, $-21 \le l \le 21$	$-12 \le h \le 12$, $-12 \le k \le 12$, $-22 \le l \le 22$	$-16 \le h \le 16$, $-18 \le k \le 18$, $-24 \le l \le 24$	$-17 \le h \le 17$, $-27 \le k \le 27$, $-35 \le l \le 36$	$-39 \le h \le 39$, $-10 \le k \le 11$, $-21 \le l \le 21$
Reflections collected	13293	20515	33918	24387	111285	21130
Independent reflections, R_{int}	13293, #	3290, 0.0517	6107, 0.0271	3798, 0.0315	9148, 0.0951	4612, 0.0426
Data/restraints/parameters	13293/18/381	3290/0/187	6107/0/342	3798/0/209	9148/210/522	4612/3/253
Goodness–of–fit on F^2	1.155	1.019	1.050	1.031	1.054	1.144
Final R1, wR2 [I>=2σ(I)]	0.0711, 0.1189	0.0323, 0.0680	0.0267, 0.0707	0.0278, 0.0734	0.0470, 0.1023	0.0458, 0.1023
Final R1, wR2 [all data]	0.0996, 0.1294	0.0545, 0.0755	0.0293, 0.0722	0.0305, 0.0751	0.0754, 0.1184	0.0579, 0.1072
Largest diff. peak/hole/ e Å^{-3}	0.56/−0.32	0.36/−0.27	0.42/−0.17	0.51/−0.20	0.48/−0.27	0.85/−0.25

#data from twinned sample.

S-2. Computational Details

DFT calculations were run with Gaussian 09 (Revision D.01).[10] Geometry optimizations and thermodynamic corrections were performed with the BP86 functional[11,12] with Ni and P centres described by Stuttgart RECPs and associated basis sets[13] and 6-31G** basis sets for all other atoms.[14,15] A set of d-orbital polarization functions was added to P ($\zeta^d = 0.387$).[16] All stationary points were fully characterized via analytical frequency calculations as either minima (all positive frequencies) or transition states (one negative frequency) and the latter were characterized via IRC calculations and subsequent geometry optimizations to confirm the adjacent minima. Key stationary points were subjected to conformational searching using our published protocol[17] and the lowest energy conformers reported. Electronic energies were recomputed with the B97D functional[18] using def2-TZVP basis sets[19,20] and a correction for benzene solvent (PCM approach).[21] Additional testing was performed with the BLYP,[11,22] B3LYP,[23] PBE,[24] PBE0,[25] B97D3,[26] ωB97x-D,[27] M06[28] and TPSS[29] functionals. Details of functional testing and all computed structures are provided below, the latter also as a separate XYZ file. RMSD calculations were performed with Chemcraft.[30] Following a suggestion by a reviewer, key stationary points were also optimised including benzene solvent in the optimisation procedure. Geometries were not significantly affected and computed energy differences were all within 0.8 kcal/mol of those determined using the ‘gas-phase’ protocol described above (see Table S3).

Details of Functional Testing

Table S2. Comparison of computed vs experiment geometries (Å) for **6**, **7**, **22**, **21** and **16** using a range of density functionals. RMSD is the root mean square deviation between the structures, neglecting the H atom positions.

	Experiment	BP86	BLYP	B3LYP	PBE	PBE0	B97D	B97D3	wB97x-D	M06	TPSS
**$Ni(IMe_4)_2(PPh_2)_2$, 6**											
Ni-P	2.2628(5)	2.327	2.378	2.343	2.325	2.298	2.339	2.378	2.299	2.318	2.33
Ni-C	1.8893(18)	1.892	1.924	1.919	1.890	1.897	1.883	1.896	1.885	1.884	1.897
RMSD		0.645	0.968	0.959	0.608	0.889	0.944	0.585	0.611	0.242	0.643
**$Ni(IEt_2Me_2)_2(PPh_2)_2$, 7**											
Ni-P	2.2668(3)	2.331	2.398	2.363	2.329	2.31	2.344	2.384	2.315	2.334	2.334
Ni-C	1.8905(12)	1.896	1.923	1.917	1.893	1.891	1.885	1.894	1.886	1.885	1.900
RMSD		0.226	0.277	0.227	0.169	0.146	0.249	0.264	0.213	0.211	0.233
**$Ni(I^iPr)_2(PH_2)_2$, 22**											
Ni-P	2.2241(9)	2.281	2.324	2.301	2.277	2.263	2.29	2.313	2.271	2.275	2.284
Ni-C	1.890(3)	1.888	1.913	1.909	1.886	1.887	1.882	1.9	1.885	1.878	1.893
RMSD		0.179	0.166	0.161	0.185	0.181	0.344	0.247	0.269	0.288	0.171
**$Ni(IiPr)_2(PMesH)_2$, 21**											
Ni-P(ave)	2.2610(11)	2.326	2.386	2.352	2.321	2.301	2.319	2.352	2.300	2.317	2.327
Ni-C(ave)	1.892(3)	1.904	1.931	1.928	1.901	1.902	1.89	1.906	1.893	1.891	1.909
RMSD		0.166	0.226	0.170	0.134	0.111	0.14	0.115	0.110	0.104	0.171
**$Ni(IiPr)_2(PPhH)_2$, 16**											
Ni-P(ave)	2.2407(18)	2.292	2.344	2.316	2.287	2.272	2.293	2.330	2.278	2.286	2.295
Ni-C(ave)	1.892(6)	1.896	1.921	1.917	1.893	1.891	1.884	1.906	1.886	1.882	1.899
RMSD[a]											

[a] disorder in the experimental structure prevented an RMSD measurement.

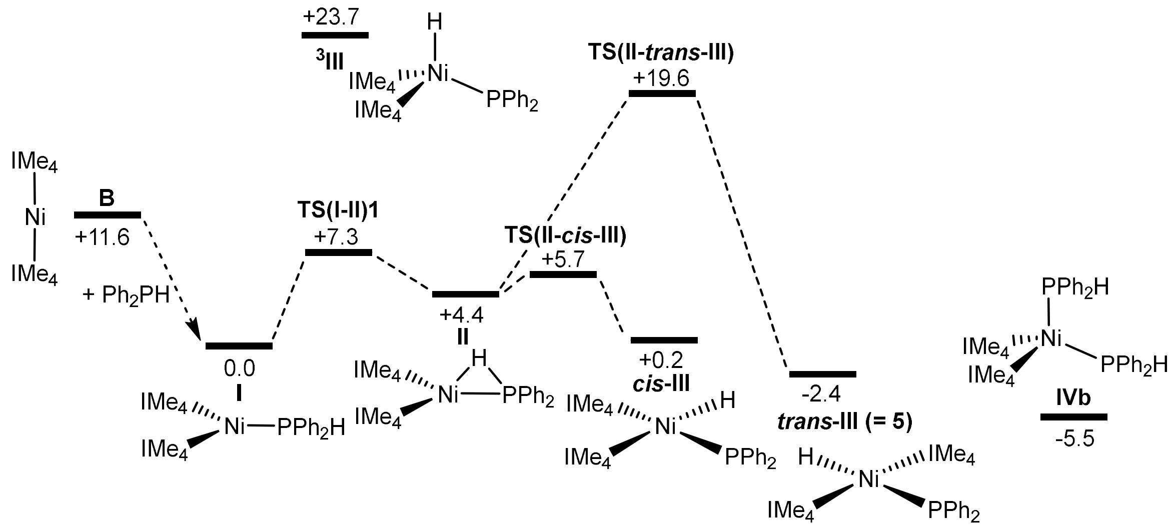

		BP86	ωB97x-D	BLYP	B3LYP	PBE	PBE0	B97D3	B97D	M06	TPSS
1	**II**	2.76	5.13	4.42	5.07	2.57	3.69	4.09	4.36	5.77	2.87
2	***cis*-III**	-1.88	-8.46	-0.56	-5.31	-0.85	-6.98	0.88	0.23	-3.44	-2.53
3	***trans*-III**	-5.36	-13.20	-4.38	-10.36	-3.58	-11.55	-2.60	-2.36	-8.11	-5.44
4	**IVb**	-17.58	-4.20	-10.13	-7.15	-10.93	-8.86	-9.55	-5.50	0.28	-13.86
5	**^{3}III**	22.38	13.44	22.15	14.68	23.57	12.13	24.09	23.70	20.24	20.12
6	**^{3}III →TS(II-trans-III)**	-4.79	6.26	-3.28	4.26	-5.87	5.67	-3.47	-4.06	3.20	-3.21
7	***cis*-III → *trans*-III**	-3.47	-4.74	-3.82	-5.05	-2.73	-4.57	-3.48	-2.59	-4.67	-2.91
8	**IVb→*trans*-III**	12.22	-9.00	5.75	-3.21	7.34	-2.70	6.95	3.14	-8.38	8.42
9	**$\Delta G^{\ddagger}$**	19.47	28.15	19.43	24.25	18.54	24.77	20.62	19.64	26.88	19.44

Figure S70. Computed free energy profile (kcal/mol, B97D) and functional testing for the reaction of [Ni(IMe$_4$)$_2$] (**B**) with PPh$_2$H to form *trans*-[Ni(IMe$_4$)$_2$(PPh$_2$)H] **5**.

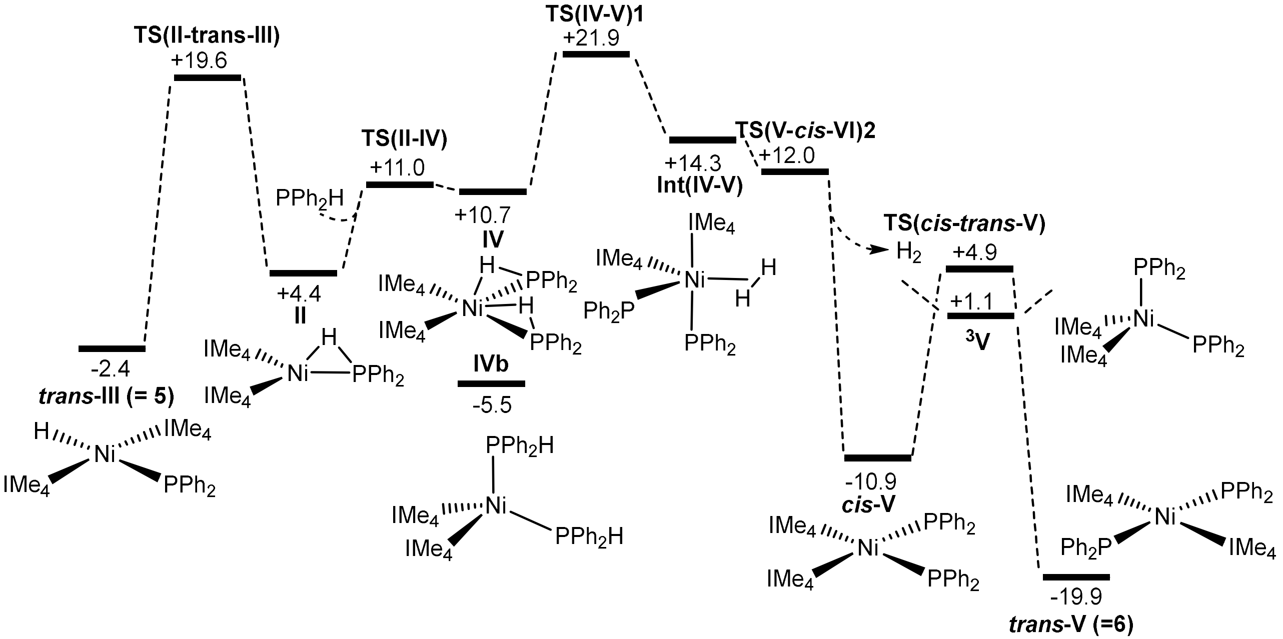

		BP86	ωB97x-D	BLYP	B3LYP	PBE	PBE0	B97D3	B97D	M06	TPSS
1	**IV**	-0.65	13.01	7.65	10.94	4.83	7.93	8.17	10.74	17.56	3.74
2	**IVb**	-17.58	-4.20	-10.13	-7.15	-10.93	-8.86	-9.55	-5.50	0.28	-13.86
3	***trans*-III**	-5.36	-13.20	-4.38	-10.36	-3.58	-11.55	-2.60	-2.36	-8.11	-5.44
4	***cis*-V**	-21.33	-14.26	-16.69	-16.76	-12.18	-15.77	-13.75	-10.90	-8.33	-16.97
5	***trans*-V**	-29.46	-26.88	-25.49	-27.90	-20.31	-26.82	-22.48	-19.89	-20.70	-25.31
6	**^{3}V**	-6.40	-3.92	-3.20	-6.57	0.58	-8.09	-1.62	1.11	4.68	-4.84
7	**^{3}V → TS(*cis-trans*-V)**	1.79	15.31	2.73	11.26	2.78	14.91	3.84	3.75	10.35	4.32
8	***cis*-V → *trans*-V**	-8.13	-12.62	-8.80	-11.14	-8.13	-11.05	-8.73	-8.99	-12.37	-8.34
9	**$\Delta G^{\ddagger}$**	12.28	34.95	20.66	29.11	18.71	27.66	22.79	24.26	36.46	16.82
10	**$\Delta\Delta G^{\ddagger}$ (1st PH vs 2nd PH) TS(IV-V) → TS(II-trans-II)**	-7.19	6.80	1.22	4.87	0.17	2.89	2.17	4.62	9.58	-2.62

Figure S71. Computed free energy profile (kcal/mol, B97D) and functional testing for the reaction of **5** to form *trans*-[Ni(IMe$_4$)$_2$(PPh$_2$)$_2$] **6**.

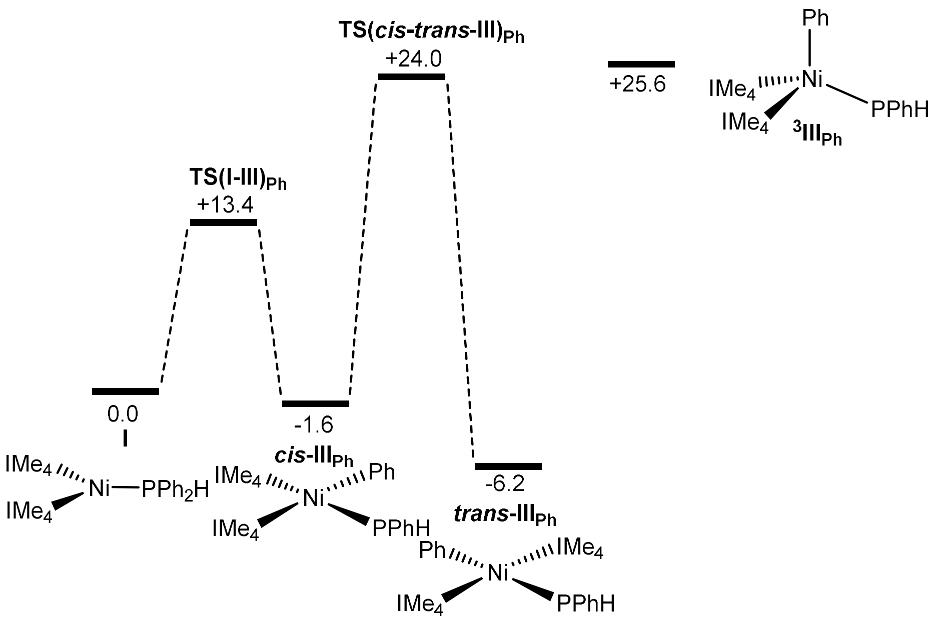

		BP86	ωB97x-D	BLYP	B3LYP	PBE	PBE0	B97D3	B97D	M06	TPSS
1	***cis*-III**$_{Ph}$	-1.80	-10.08	-1.38	-6.40	-0.04	-6.99	0.84	-1.59	-6.13	-3.73
2	***trans*-III**$_{Ph}$	-6.12	-16.18	-6.06	-12.10	-4.49	-12.68	-3.85	-6.15	-12.19	-8.00
3	^{3}**III**$_{Ph}$	25.32	9.81	23.25	12.71	27.23	10.89	25.30	22.98	16.97	21.08
4	$\Delta G^{\ddagger}$	25.54	35.46	25.35	31.22	25.03	32.35	26.50	25.57	33.88	25.50
5	$\Delta\Delta G^{\ddagger}$ **(P-H vs. P-C)**	-6.06	-7.31	-5.92	-6.98	-6.49	-7.59	-5.88	-5.93	-7.00	-6.06
6	^{3}**III**$_{Ph}$ → **TS(*cis-trans*-III)**$_{Ph}$	-1.59	15.57	0.73	12.11	-2.24	14.48	1.20	1.00	10.77	0.69
7	***trans*-III** → ***trans*-III**$_{Ph}$	-0.78	-3.00	-1.71	-1.77	-0.93	-1.15	-1.27	-3.81	-4.10	-2.59

Figure S72. Computed free energy profile (kcal/mol, B97D) and functional testing for P-C bond activation in [Ni(IMe$_4$)$_2$(PPh$_2$H)], **I**.

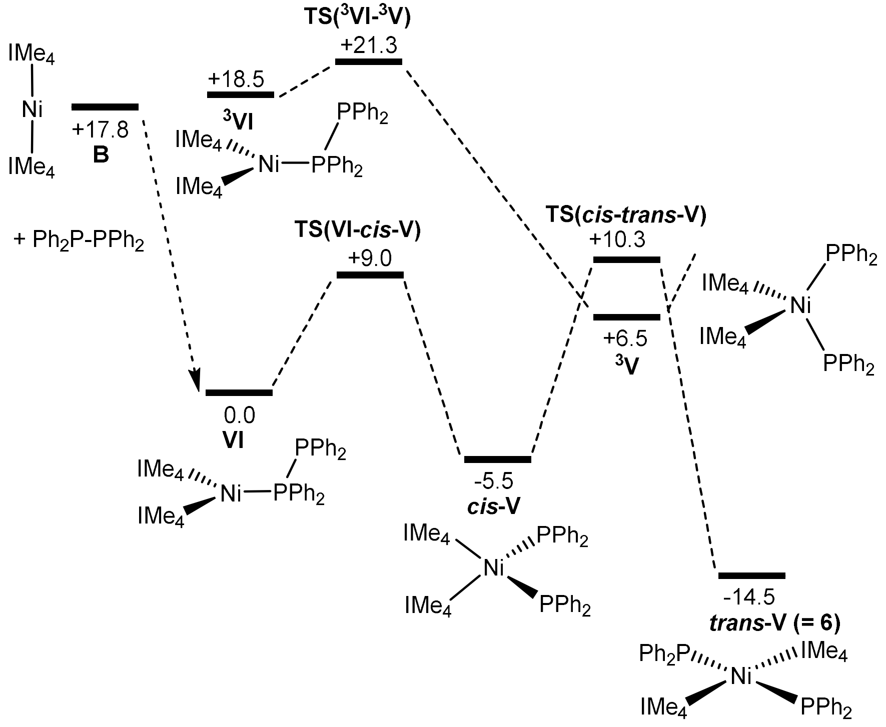

		BP86	ωB97x-D	BLYP	B3LYP	PBE	PBE0	B97D3	B97D	M06	TPSS
1	**VI → trans-V**	-15.52	-27.88	-15.44	-22.13	-13.79	-22.83	-13.27	-14.50	-22.54	-17.26
2	**ΔG‡**	16.73	25.65	16.22	21.45	15.55	22.59	15.97	15.76	23.37	16.45
3	**³VI → VI**	-24.54	-9.62	-20.28	-12.17	-22.84	-11.26	-19.97	-18.47	-12.66	-21.27
4	**³V → TS(cis-trans-VI)**	1.79	15.31	2.73	11.26	2.78	14.91	3.84	3.75	10.35	4.32
5	**³V → trans-V**	-23.07	-22.96	-22.29	-21.34	-20.90	-18.73	-20.86	-21.00	-25.39	-20.46

Figure S73. Computed free energy profiles (kcal/mol, B97D) and functional testing for the reaction of **B** with $Ph_2P{-}PPh_2$ to form **6**.

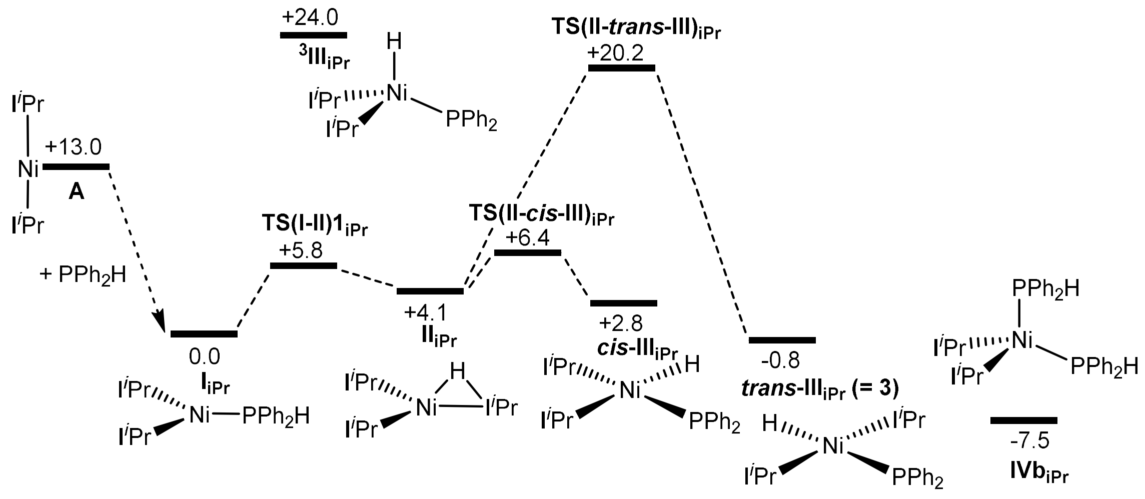

		BP86	ωB97x-D	BLYP	B3LYP	PBE	PBE0	B97D3	B97D	M06	TPSS
1	**II_{iPr}**	2.41	4.99	4.03	4.76	2.26	3.45	3.71	4.08	5.40	2.53
2	***cis*-III_{iPr}**	0.97	-4.70	2.57	-1.59	2.02	-3.38	4.07	2.82	-0.02	0.42
3	***trans*-III_{iPr}**	-3.81	-11.59	-2.59	-8.42	-1.96	-9.71	-0.81	-0.80	-7.32	-3.62
4	**IVb**	-19.88	-6.72	-12.19	-9.04	-11.82	-9.91	-11.23	-7.51	-4.54	-14.70
5	**^{3}III**	22.75	14.51	22.54	15.38	23.83	12.76	24.36	23.98	20.58	20.33
6	**^{3}III → TS(II-trans-III)**	-4.64	6.13	-3.10	4.32	-5.57	5.73	-3.16	-3.83	3.03	-2.89
7	***cis*-III_{iPr} → *trans*-III_{iPr}**	-4.78	-6.89	-5.16	-6.82	-3.98	-6.33	-4.88	-3.62	-7.30	-4.05
8	**IVb → *trans*-III_{iPr}**	16.07	-4.87	9.60	0.62	9.86	0.19	10.43	6.71	-2.78	11.08
9	**$\Delta G^{\ddagger}$**	18.11	20.64	19.44	19.70	18.26	18.49	21.20	20.15	23.61	17.44

Figure S74. Computed free energy profile (kcal/mol, B97D) and functional testing for the reaction of $[Ni(I^{i}Pr)_2]$ (**A**) with PPh_2H to form *trans*-$[Ni(I^{i}Pr)_2(PPh_2)H]$ **3**.

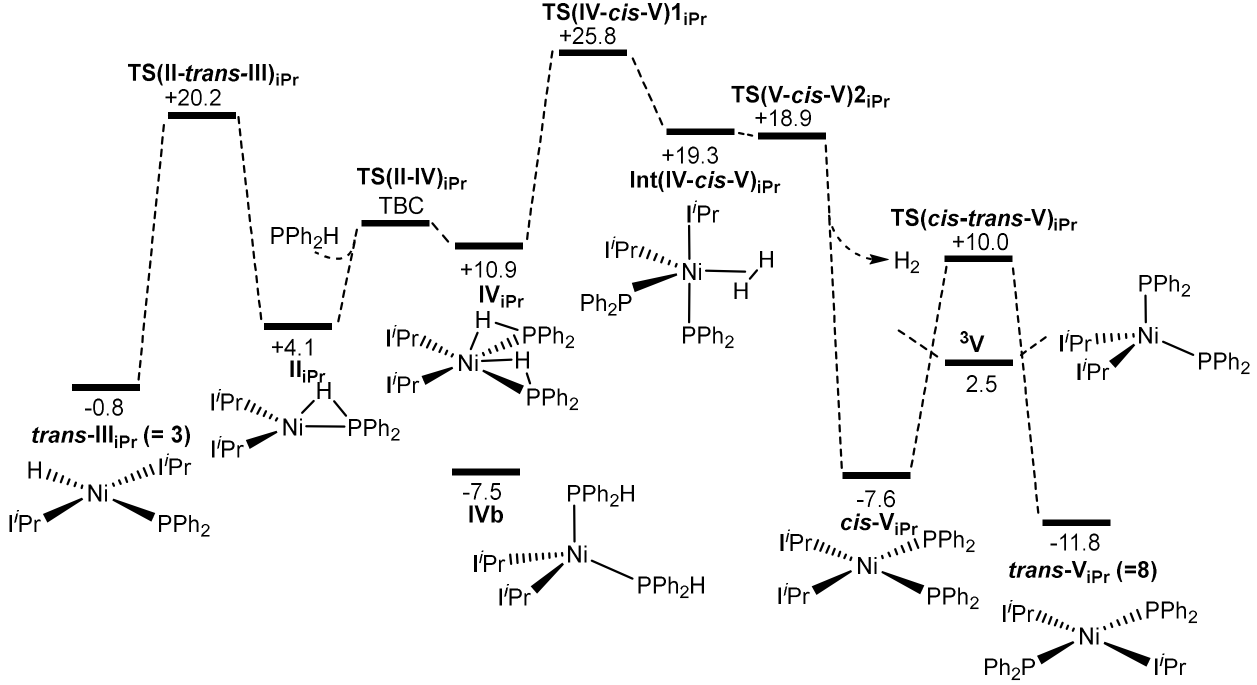

		BP86	ωB97x-D	BLYP	B3LYP	PBE	PBE0	B97D3	B97D	M06	TPSS
1	**IV_{iPr}**	0.45	13.82	8.71	12.25	6.77	9.97	9.24	10.90	16.67	5.63
2	**IVb_{iPr}**	-19.88	-6.72	-12.19	-9.04	-11.82	-9.91	-11.23	-7.51	-4.54	-14.70
3	***trans*-III_{iPr}**	-3.81	-11.59	-2.59	-8.42	-1.96	-9.71	-0.81	-0.80	-7.32	-3.62
4	***cis*-V_{iPr}**	-17.79	-4.19	-12.76	-9.05	-7.96	-6.88	-10.13	-7.57	-1.13	-12.42
5	***trans*-V_{iPr}**	-22.61	-18.09	-16.29	-17.76	-10.85	-16.69	-13.05	-11.76	-11.89	-15.95
6	**^3V**	-5.24	-1.32	-1.40	-3.71	2.71	-4.94	0.19	2.49	6.22	-2.77
7	***cis*-V_{iPr} → *trans*-V_{iPr}**	-4.83	-13.90	-3.53	-8.70	-2.90	-9.81	-2.92	-4.20	-10.76	-3.53
8	**3 → 8**	-18.80	-6.50	-13.70	-9.34	-8.90	-6.98	-12.24	-10.96	-4.57	-12.33
9	**IVb → 8**	-2.74	-11.37	-4.11	-8.72	0.97	-6.78	-1.82	-4.26	-7.35	-1.25
10	**^3V → TS(*cis-trans*-V)**	5.48	19.82	6.30	14.95	7.32	19.33	8.02	7.53	14.02	8.83
11	**$\Delta G^{\ddagger}$**	15.02	37.87	23.74	32.48	22.28	31.43	25.78	26.61	38.27	20.61
12	**$\Delta\Delta G^{\ddagger}$ (2nd P-H vs. 1st P-H)**	3.09	-17.23	-4.30	-12.79	-4.02	-12.94	-4.58	-6.46	-14.66	-3.17

Figure S75. Computed free energy profile (kcal/mol, B97D) and functional testing for the reaction of **3** to form *trans*-[Ni(I^{i}Pr)$_2$(PPh$_2$)$_2$] **8**.

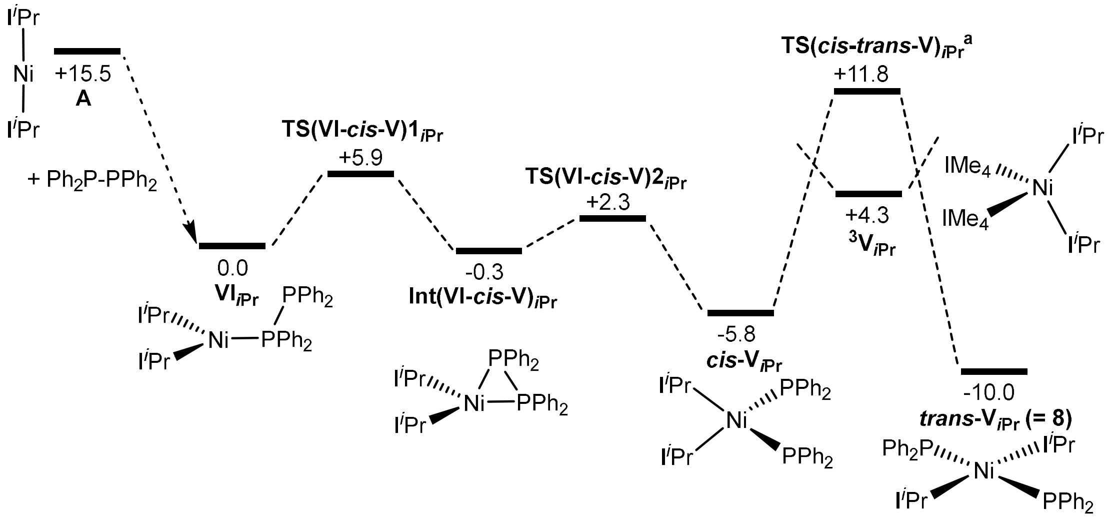

		BP86	ωB97x-D	BLYP	B3LYP	PBE	PBE0	B97D3	B97D	M06	TPSS
1	**ΔG**	-12.98	-23.41	-10.54	-16.79	-9.19	-17.97	-8.41	-9.98	-19.71	-12.87
2	**ΔG‡**	18.02	22.69	17.67	20.29	17.98	21.27	18.34	17.58	21.37	18.48
3	**^{3}VI→ V**	-17.37	-16.77	-14.90	-14.04	-13.57	-11.75	-13.24	-14.25	-18.11	-13.18
4	**^{3}V → TS(cis-trans-V)**	5.48	19.82	6.30	14.94	7.32	19.33	8.02	7.53	14.02	8.83
5	**^{3}V → trans-V**	-17.37	-16.77	-14.90	-14.04	-13.57	-11.75	-13.24	-14.25	-18.11	-13.18

Figure S76. Computed free energy reaction profile (kcal/mol) and functional testing for the reaction of **A** with Ph_2P-PPh_2 to form *trans*-[Ni(I^{i}Pr)$_2$(PPh$_2$)$_2$], **8**; [a] only the rate-limiting transition state for *cis-trans* isomerisation is indicated (see Figure SX for more details).

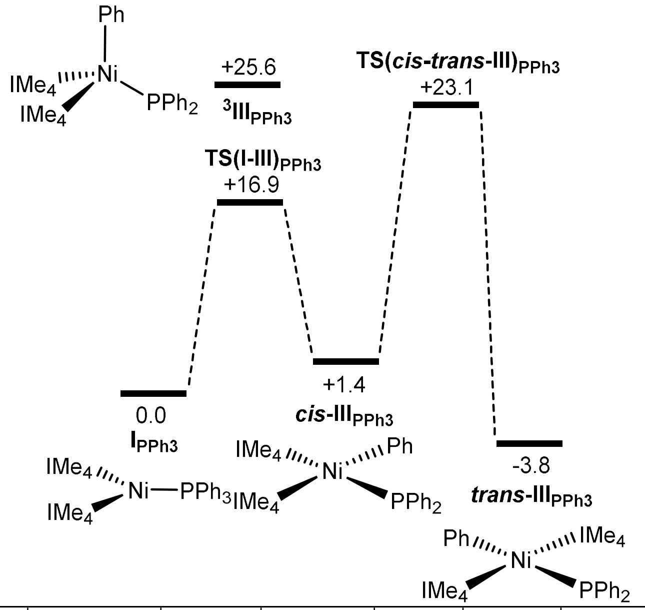

		BP86	ωB97x-D	BLYP	B3LYP	PBE	PBE0	B97D3	B97D	M06	TPSS
1	ΔG	-4.22	-14.90	-4.18	-10.26	-2.75	-10.97	-1.87	-3.84	-11.32	-6.31
2	$\Delta G^{\ddagger}$	22.54	31.36	22.62	27.32	23.33	28.39	25.16	23.13	29.83	21.82
3	^{3}III$_{PPh3}$ → TS(*cis-trans*-III)$_{PPh3}$	-4.98	13.49	-2.70	9.31	-5.02	12.18	-2.17	-2.48	7.60	-2.41

Figure S77. Computed free energy profile (kcal/mol, B97D) and functional testing for P-C bond activation in [Ni(IMe$_4$)$_2$(PPh$_3$)].

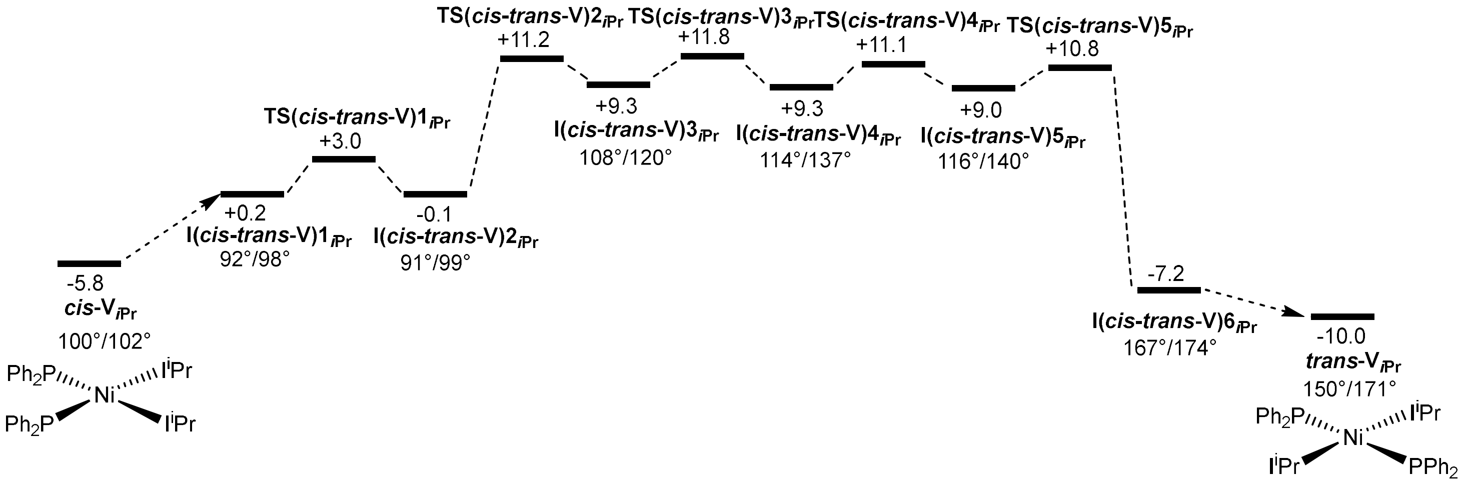

		BP86	ωB97x-D	BLYP	B3LYP	PBE	PBE0	B97D3	B97D	M06	TPSS
1	***cis*-V** $_{\mathbf{iPr}}$	-8.15	-9.51	-7.01	-8.09	-6.29	-8.16	-5.49	-5.79	-8.95	-9.35
2	***trans*-V** $_{\mathbf{iPr}}$	-12.98	-23.41	-10.54	-16.79	-9.19	-17.97	-8.41	-9.98	-19.71	-12.87
3	**TS(*cis-trans*-V)3** $_{\mathbf{iPr}}$	9.87	13.18	10.66	12.20	11.69	13.11	12.85	11.80	12.43	9.14
4	***cis*-V** $_{\mathbf{iPr}}$ **→ TS(*cis-trans*-V)3** $_{\mathbf{iPr}}$	18.02	22.69	17.67	20.29	17.98	21.27	18.34	17.58	21.37	18.48

Figure S78. Computed free energy profile (kcal/mol, B97D) and functional testing for *cis-trans* isomerisation in [Ni(I^{i}Pr)$_2$(PPh$_2$)$_2$]; also indicated are the P-Ni-P and C-Ni-C angles for each intermediate.

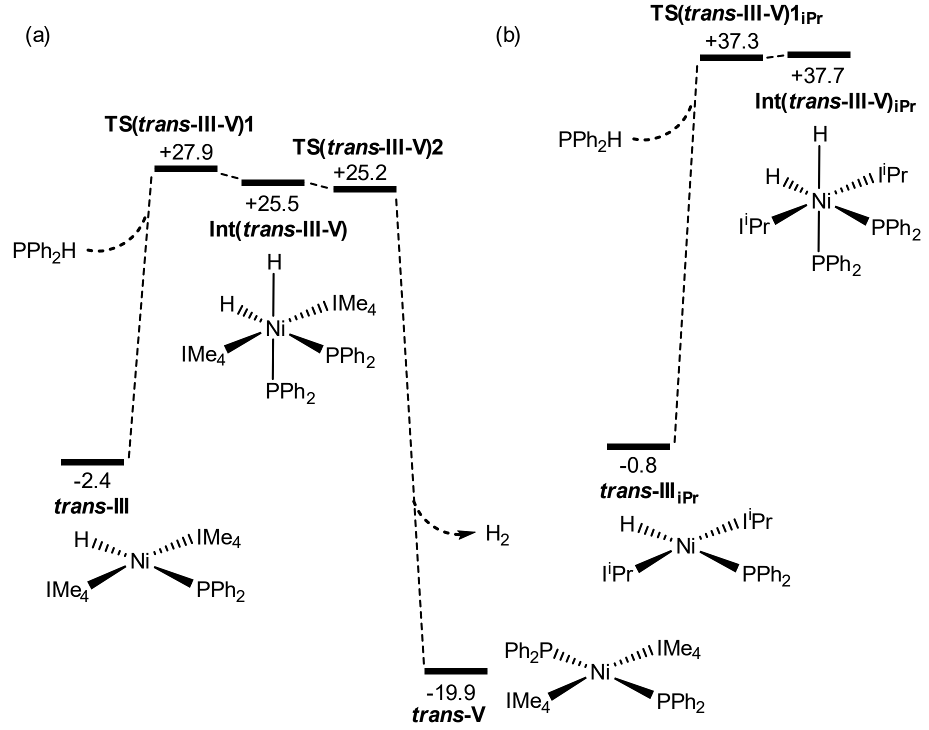

	IMe4	BP86	ωB97x-D	BLYP	B3LYP	PBE	PBE0	B97D3	B97D	M06	TPSS
1	***trans*-III**	-5.36	-13.20	-4.38	-10.36	-3.58	-11.55	-2.60	-2.36	-8.11	-5.44
2	**TS(*trans*-III-V)1**	12.16	28.09	21.64	24.28	21.07	21.97	26.21	27.93	34.24	16.85
3	**$\Delta G^‡$**	17.51	41.29	26.02	34.64	24.65	33.52	28.80	30.29	42.35	22.29
4	**TS(trans-III-V)1 → TS(*cis*-IV- V)**	-5.23	-6.35	-5.37	-5.53	-5.94	-5.86	-6.02	-6.03	-5.88	-5.46
	**I^{i}Pr**	**BP86**	**ωB97x-D**	**BLYP**	**B3LYP**	**PBE**	**PBE0**	**B97D3**	**B97D**	**M06**	**TPSS**
5	***trans*-III$_{iPr}$**	-3.81	-11.59	-2.59	-8.42	-1.96	-9.71	-0.81	-0.80	-7.32	-3.62
6	**TS(*trans*-III-V)1$_{iPr}$**	21.84	39.44	32.20	35.87	30.56	33.08	36.66	37.32	43.85	27.36
7	**$\Delta G^‡$**	25.65	51.02	34.79	44.29	32.52	42.79	37.47	38.12	51.17	30.98
8	**TS(trans-III-V)1 → TS(*cis*-IV- V)**	-10.63	-13.15	-11.05	-11.81	-10.24	-11.36	-11.69	-11.51	-12.90	-10.37

Figure S79. Reaction profile (kcal/mol, B97D) and functional testing for P-H activation at (a) ***trans*-III** (**5**) to form ***trans*-V** (**6**) and (b) ***trans*-III$_{iPr}$**(**3**). In the latter case only **TS(*trans*-III-V)1** was characterised.

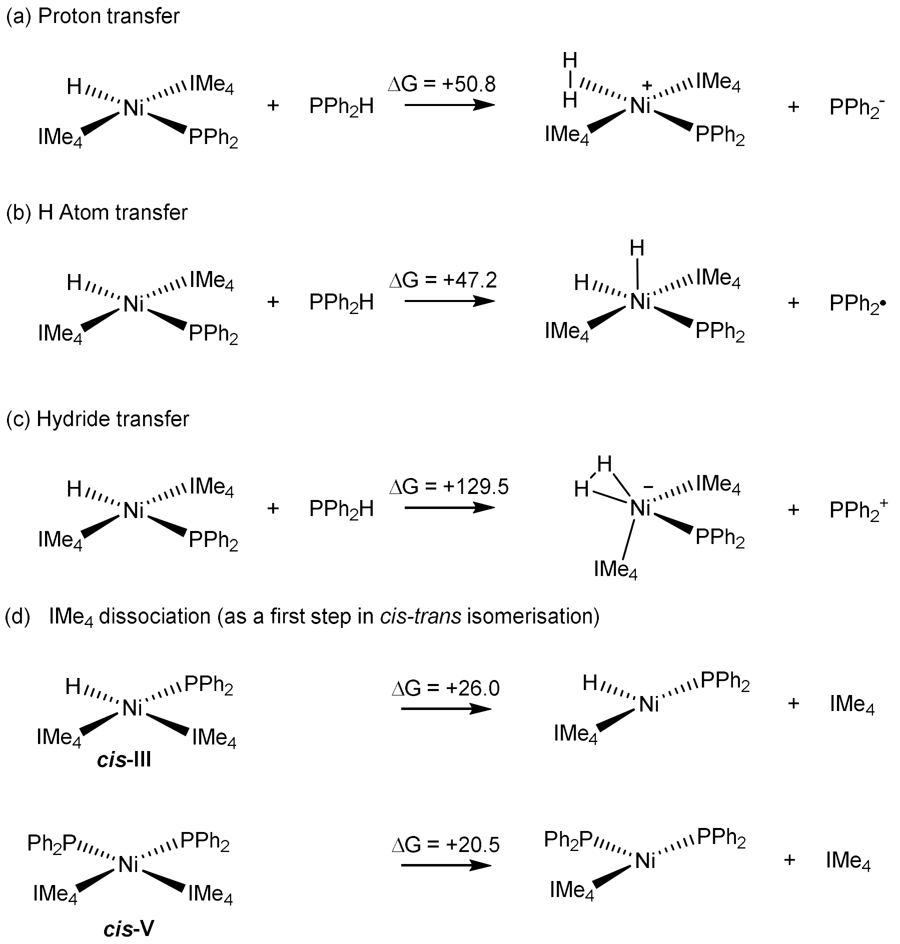

		BP86	ωB97x-D	BLYP	B3LYP	PBE	PBE0	B97D3	B97D	M06	TPSS
1	**ΔG H^+ Transfer**	51.34	57.49	51.37	54.63	51.64	55.26	51.46	50.81	55.10	52.25
2	**ΔG H atom transfer**	47.86	56.25	48.90	52.45	47.31	51.19	49.89	47.19	56.16	46.27
3	**ΔG H^- Transfer**	131.83	157.07	132.13	146.74	130.47	149.27	132.48	129.54	142.81	134.10
4	***cis*-III - IMe_4**	33.80	31.67	27.69	29.80	29.41	32.70	25.68	26.04	24.49	32.95
5	***cis*-V - IMe_4**	29.69	25.68	22.42	23.36	23.63	26.04	21.08	20.45	20.29	26.08

Figure S80. Computed energies (kcal/mol, B97D) and functional testing for (a) proton transfer, (b) H atom transfer and (c) hydride transfer from PPh_2H to **trans-III** (**5**) and (d) IMe_4 dissociation from ***cis*-III** and ***cis*-V**

Table S3. Comparison of the relative free energies of key stationary points (kcal/mol) based on geometries optimised with and without benzene solvent. Energies are computed with B97D with a def2-tzvp basis set. Energies are quoted relative to **I** or **VI** as appropriate.

**Stationary Point**	**'Gas-phase' optimization**	**Optimisation in solvent**
**I**	0.0	0.0
**TS(II-*trans*-III)**	+19.64	+19.66
**TS(IV-*cis*-V)**	+21.90	+22.71
***trans*-III**	-2.36	-2.59
***cis*-V**	-10.90	-10.44
***trans*-V**	-19.89	-19.43
**VI**	0.0	0.0
**TS(*cis-trans*-V)**	+10.25	+10.60

S-4. Computed Cartesian Coordinates (Å) and Energies (hartrees) for all species.

1. Ni(IMe4)2

(i) 1st P-H Activation

Ni(IMe4)2, **B**
BP86
SCF = -938.029118479
H(0 K)= -937.672328
H(298 K)= -937.647064
G(298 K)= -937.727462
SCF(C6H6) = -938.034845779
SCF(D3BJ) = -938.107881829
SCF(BS2) = -2275.83679126
Low Freq. = 16.2338cm-1, 22.8507cm-1
B97D
SCF(C6H6,BS2)= -2275.72910768

43
B

```
Ni  0.00000 -0.00008  0.00033
C   1.85410 -0.00002  0.00025
N   2.72651  0.76584  0.76307
C   4.07116  0.48784  0.48590
C   4.07091 -0.48804 -0.48633
N   2.72615 -0.76594 -0.76295
C   2.25900  1.74267  1.73619
C   2.25816 -1.74274 -1.73588
C   5.20305  1.18024  1.17538
C   5.20250 -1.18054 -1.17622
C  -1.85409 -0.00004  0.00024
N  -2.72611  0.76310 -0.76578
C  -4.07089  0.48632 -0.48809
C  -4.07117 -0.48597  0.48773
N  -2.72654 -0.76302  0.76589
C  -2.25810  1.73635 -1.74224
C  -2.25908 -1.73601  1.74289
C  -5.20246  1.17613 -1.18071
C  -5.20310 -1.17591  1.17962
H  -1.15538  1.70518 -1.71098
H  -2.60497  2.75448 -1.49021
H  -2.60501  1.48100 -2.75952
H  -1.15635 -1.70592  1.71122
H  -2.60706 -2.75396  1.49172
H  -2.60531 -1.47958  2.76014
H  -5.18518  2.27020 -1.01951
H  -6.16796  0.80261 -0.80429
H  -5.18631  1.00901 -2.27392
H  -5.18714 -2.26969  1.01629
H  -6.16841 -0.80059  0.80448
H  -5.18615 -1.01092  2.27312
H   5.18590 -2.27385 -1.00982
H   6.16799 -0.80472 -0.80207
H   5.18571 -1.01863 -2.27020
H   5.18621  2.27360  1.00935
H   6.16838  0.80467  0.80056
H   5.18691  1.01797  2.26931
H   2.60599  2.75982  1.48040
H   2.60618  1.49089  2.75427
H   1.15627  1.71161  1.70541
H   1.15543 -1.71183 -1.70437
H   2.60548 -2.75986 -1.48042
H   2.60464 -1.49081 -2.75416
```

PPh_2H
BP86
SCF = -470.453464231
H(0 K)= -470.267964
H(298 K)= -470.255588
G(298 K)= -470.307875
SCF(C6H6) = -470.455504051
SCF(D3BJ) = -470.503651437
SCF(BS2) = -805.445409822
Low Freq. = 19.0392cm-1, 26.1331cm-1
B97D
SCF(C6H6,BS2)= -805.068399390

24
PPh_2H

```
P   0.05793  1.69275 -0.27242
C   1.46886  0.45948 -0.13520
C   1.36731 -0.90011 -0.49620
C   2.69464  0.94485  0.36565
H   0.41585 -1.29179 -0.87126
H   2.77746  1.99614  0.66520
C   2.47463 -1.75231 -0.36851
C   3.80572  0.09301  0.48530
H   2.38428 -2.80676 -0.65169
H   4.75273  0.48381  0.87280
C   3.69660 -1.25723  0.11912
H   4.55902 -1.92499  0.21808
C  -1.43832  0.59565 -0.07850
C  -2.19589  0.12540 -1.17327
C  -1.86752  0.27587  1.22944
H  -1.88198  0.36634 -2.19490
H  -1.30097  0.64627  2.09166
C  -3.34981 -0.64865 -0.96526
C  -3.01087 -0.50959  1.43604
H  -3.92760 -1.00347 -1.82559
H  -3.32601 -0.75361  2.45622
C  -3.75712 -0.97156  0.33859
H  -4.65471 -1.57764  0.50012
H   0.00367  1.74560 -1.71753
```

I
BP86
SCF = -1408.50464953
H(0 K)= -1407.962262
H(298 K)= -1407.923454
G(298 K)= -1408.038577
SCF(C6H6) = -1408.50979099
SCF(D3BJ) = -1408.66171559
SCF(BS2) = -3081.30871617
Low Freq. = 8.0358cm-1, 14.4474cm-1
B97D
SCF(C6H6,BS2)= -3080.83488392

```
67
I
Ni  0.02981 -0.62434 -0.19204
P  -0.00163  1.45703 -0.73311
N  -2.25351 -2.28224 -1.10608
N  -2.77893 -1.17446  0.67232
N   2.77805 -1.67924 -0.69155
N   2.12975 -2.04636  1.33721
C  -1.71244 -1.38493 -0.19322
C  -3.58899 -2.60768 -0.82229
C  -3.92025 -1.90804  0.31489
C  -1.48660 -2.79979 -2.22736
H  -0.42553 -2.59328 -2.00559
H  -1.75934 -2.30398 -3.17683
H  -1.63847 -3.88761 -2.33629
C  -2.68859 -0.29921  1.83184
H  -1.71340  0.20914  1.77659
H  -2.75037 -0.87240  2.77532
H  -3.48614  0.46233  1.81798
C  -4.39730 -3.54371 -1.66298
H  -4.47315 -3.20157 -2.71202
H  -5.42231 -3.62493 -1.26749
H  -3.97161 -4.56461 -1.68452
C  -5.19487 -1.85664  1.09505
H  -5.05882 -2.19470  2.13939
H  -5.95387 -2.50855  0.63411
H  -5.61667 -0.83516  1.13760
C   1.68935 -1.46874  0.15017
C   3.83170 -2.35632 -0.05838
C   3.42293 -2.58216  1.23549
C   4.12349 -3.24575  2.37800
H   4.23369 -2.57093  3.24733
H   5.13445 -3.56209  2.07554
H   3.58942 -4.14729  2.73271
C   5.10651 -2.71004 -0.75561
H   4.93515 -3.36246 -1.63218
H   5.78114 -3.24848 -0.07119
H   5.64862 -1.81633 -1.11762
C   2.78578 -1.24960 -2.08016
H   2.68574 -2.10460 -2.77419
H   3.71455 -0.70398 -2.31933
H   1.92262 -0.57631 -2.21102
C   1.30623 -2.07041  2.53485
H   0.27098 -1.87111  2.20874
H   1.61101 -1.29518  3.26139
H   1.35805 -3.05812  3.02441
C  -1.37027  2.56233 -0.07285
C  -2.66504  2.47148 -0.63208
H  -2.82824  1.84064 -1.51405
C  -3.74090  3.17636 -0.07171
H  -4.73432  3.10538 -0.52962
C  -3.54647  3.97705  1.06857
H  -4.38605  4.52712  1.50696
C  -2.26376  4.07250  1.63393
H  -2.09902  4.70091  2.51674
C  -1.18474  3.37429  1.06769
H  -0.18543  3.47011  1.50710
C   1.46642  2.63338 -0.58327
C   1.53331  3.87228 -1.25677
C   2.55328  2.23548  0.21935
C   2.66651  4.69095 -1.13358
C   3.68333  3.06047  0.35614
C   3.74286  4.28772 -0.32310
H   0.69069  4.19900 -1.87726
H   2.50237  1.26035  0.71991
H   2.71006  5.64756 -1.66664
H   4.51994  2.74173  0.98842
H   4.62563  4.92919 -0.22498
H  -0.27817  1.72460 -2.13479
```

TS(I-II)

```
BP86
SCF  =       -1408.49744658
H(0 K)=      -1407.956332
H(298 K)=    -1407.918092
G(298 K)=    -1408.030648
SCF(C6H6) =        -1408.50285262
SCF(D3BJ) =        -1408.65300693
SCF(BS2) =         -3081.30055977
Low Freq. = -210.3382cm-1,
15.607cm-1
B97D
SCF(C6H6,BS2)=  -3080.82403754
```

```
67
TS(I-II)
Ni -0.00291 -0.69087  0.01065
P  -0.02775  1.47464  0.12370
N  -2.24860 -2.31724 -1.02942
N  -2.77388 -1.41814  0.86269
N   2.77467 -1.39264 -0.89083
N   2.27463 -2.22773  1.03865
C  -1.72658 -1.48361 -0.04807
C  -3.55192 -2.74631 -0.74103
C  -3.88389 -2.17819  0.46757
C  -1.49361 -2.69151 -2.21530
H  -0.44844 -2.39326 -2.02332
H  -1.86343 -2.16972 -3.11650
H  -1.54756 -3.78086 -2.38556
C  -2.69972 -0.65303  2.09871
H  -1.79070 -0.03325  2.04154
H  -2.63519 -1.31639  2.98042
H  -3.58103  0.00123  2.20744
C  -4.33454 -3.64363 -1.64595
H  -4.46831 -3.20511 -2.65231
H  -5.33791 -3.82674 -1.22975
H  -3.84989 -4.62845 -1.78276
C  -5.13418 -2.27581  1.28217
H  -4.94320 -2.68869  2.29017
H  -5.86335 -2.93684  0.78753
H  -5.62038 -1.29222  1.42088
C   1.72776 -1.44824  0.02421
C   3.91203 -2.09069 -0.45673
C   3.59417 -2.62101  0.77201
C   4.39832 -3.46454  1.70915
H   4.49586 -2.99941  2.70758
H   5.41545 -3.61402  1.31335
H   3.95339 -4.46618  1.85983
C   5.16663 -2.18289 -1.26534
H   5.00433 -2.69345 -2.23338
H   5.93374 -2.75354 -0.71824
H   5.59176 -1.18745 -1.49101
C   2.70624 -0.63393 -2.13021
H   3.00814 -1.25553 -2.99106
```

```
H   3.34936  0.26318 -2.08875
H   1.66104 -0.31437 -2.26048
C   1.53414 -2.56072  2.24544
H   0.47641 -2.32290  2.03847
H   1.87554 -1.96560  3.11187
H   1.63719 -3.63339  2.48449
C  -1.46875  2.66653 -0.00755
C  -2.53529  2.40356 -0.89619
H  -2.45296  1.56371 -1.59598
C  -3.69098  3.19997 -0.88964
H  -4.50230  2.98471 -1.59462
C  -3.80573  4.27575  0.00729
H  -4.70631  4.89908  0.01082
C  -2.75326  4.54597  0.89908
H  -2.83093  5.38346  1.60184
C  -1.59954  3.74703  0.89548
H  -0.78546  3.97042  1.59509
C   1.44014  2.65128  0.02045
C   1.40694  3.92565 -0.58751
C   2.65763  2.19713  0.57230
C   2.56334  4.71936 -0.64616
C   3.81554  2.98954  0.51070
C   3.77158  4.25479 -0.09799
H   0.47004  4.29824 -1.01477
H   2.68466  1.20800  1.04472
H   2.52178  5.70453 -1.12483
H   4.75128  2.62237  0.94772
H   4.67191  4.87730 -0.14299
H  -0.06345  1.00133 -1.28025
```

II

```
BP86
SCF  =       -1408.50107212
H(0 K)=      -1407.959703
H(298 K)=    -1407.920947
G(298 K)=    -1408.035199
SCF(C6H6) =        -1408.50669453
SCF(D3BJ) =        -1408.65861338
SCF(BS2) =         -3081.30313771
Low Freq. = 9.7039cm-1, 15.0778cm-1
B97D
SCF(C6H6,BS2)=  -3080.82773855
```

67

II

```
Ni  0.17897 -0.59722 -0.02583
P  -0.17698  1.48753 -0.57074
N   2.55340 -1.93993  1.13642
N   3.01457 -1.15384 -0.82156
N  -2.53246 -1.57210  0.83822
N  -1.87030 -2.50580 -0.99177
C   1.96900 -1.24245  0.08835
C   3.89552 -2.26613  0.89053
C   4.18707 -1.77093 -0.35934
C   1.82482 -2.27371  2.35078
H   0.75933 -2.08220  2.13929
H   2.14449 -1.64750  3.20316
H   1.96825 -3.33608  2.61383
C   2.89024 -0.49073 -2.11177
H   1.90951  0.01097 -2.12886
H   2.94641 -1.21549 -2.94421
H   3.68335  0.26483 -2.24290
C   4.74666 -3.00795  1.87112
H   4.82647 -2.47683  2.83779
H   5.76778 -3.13006  1.47643
H   4.35396 -4.01913  2.08744
C   5.45013 -1.81672 -1.15858
H   5.31339 -2.33668 -2.12502
H   6.23548 -2.35368 -0.60346
H   5.83770 -0.80620 -1.38574
C  -1.45467 -1.59003 -0.03523
C  -3.56772 -2.42936  0.43794
C  -3.14872 -3.02099 -0.73154
C  -3.82341 -4.01887 -1.61784
H  -3.94007 -3.64469 -2.65176
H  -4.82990 -4.25134 -1.23527
H  -3.26606 -4.97251 -1.67790
C  -4.83437 -2.59034  1.21628
H  -4.65099 -2.97621  2.23649
H  -5.50722 -3.30140  0.71137
H  -5.38054 -1.63512  1.32360
C  -2.58589 -0.72318  2.02035
H  -2.69627 -1.32664  2.93882
H  -3.42115 -0.00556  1.95336
H  -1.63843 -0.16498  2.06044
C  -1.05280 -2.85043 -2.14496
H  -0.02829 -2.50922 -1.91983
H  -1.40688 -2.34608 -3.06195
H  -1.05471 -3.94120 -2.31178
C   1.02389  2.88899 -0.22318
C   2.20913  2.64993  0.50736
H   2.35717  1.66614  0.96881
C   3.18715  3.64820  0.64102
H   4.09532  3.44416  1.22032
C   3.00164  4.90739  0.04605
H   3.76322  5.68746  0.15240
C   1.82886  5.15710 -0.68741
H   1.67320  6.13502 -1.15734
C   0.85334  4.15761 -0.82491
H  -0.05523  4.36555 -1.40185
C  -1.81936  2.30304 -0.16443
C  -1.98567  3.38178  0.73430
C  -2.97086  1.76162 -0.78095
C  -3.26237  3.89584  1.01020
C  -4.24807  2.27367 -0.50358
C  -4.39946  3.34450  0.39432
H  -1.10726  3.82216  1.21766
H  -2.85394  0.92496 -1.47963
H  -3.37030  4.73072  1.71232
H  -5.12648  1.84294 -0.99796
H  -5.39438  3.74973  0.60866
H   0.09568  0.77777  0.82918
```

TS(II-III)

```
BP86
SCF  =       -1408.50064396
H(0 K)=      -1407.959575
H(298 K)=    -1407.921505
G(298 K)=    -1408.032511
SCF(C6H6) =        -1408.50672060
SCF(D3BJ) =        -1408.65934706
SCF(BS2) =         -3081.30210050
Low Freq. = -47.4124cm-1,
14.0929cm-1
```

B97D
SCF(C6H6,BS2)= -3080.82794777

67
TS(II-*cis*-III)
```
Ni  0.38702 -0.42068 -0.02791
P  -0.51331  1.48322 -0.64452
N   2.84490 -1.57144  1.19995
N   3.29162 -0.57879 -0.66445
N  -2.03071 -2.01486  0.69546
N  -1.22336 -2.64384 -1.20470
C   2.22664 -0.87688  0.17300
C   4.23051 -1.69538  1.01341
C   4.51348 -1.06827 -0.17782
C   2.11165 -2.08416  2.34742
H   1.04131 -2.01003  2.09623
H   2.31340 -1.49012  3.25675
H   2.37587 -3.13792  2.54201
C   3.13928  0.16614 -1.90683
H   2.09132  0.50376 -1.95904
H   3.36503 -0.46580 -2.78454
H   3.80302  1.04733 -1.92165
C   5.12407 -2.39486  1.98725
H   5.08232 -1.93696  2.99290
H   6.17075 -2.34917  1.64728
H   4.86215 -3.46303  2.10490
C   5.80845 -0.88314 -0.90233
H   5.79235 -1.34181 -1.90847
H   6.63238 -1.35014 -0.34000
H   6.06281  0.18462 -1.03629
C  -0.99282 -1.74138 -0.17991
C  -2.87161 -3.03722  0.23168
C  -2.36066 -3.43617 -0.98195
C  -2.82684 -4.48183 -1.94398
H  -3.04542 -4.06067 -2.94271
H  -3.75091 -4.95511 -1.57603
H  -2.07958 -5.28499 -2.08597
C  -4.06419 -3.51414  0.99712
H  -3.78846 -3.92251  1.98721
H  -4.58166 -4.31320  0.44310
H  -4.79476 -2.70306  1.17165
C  -2.22982 -1.28548  1.94132
H  -2.15200 -1.95876  2.81370
H  -3.21234 -0.78475  1.95056
H  -1.44026 -0.51949  1.99412
C  -0.37861 -2.71461 -2.38768
H   0.53143 -2.13508 -2.16476
H  -0.87741 -2.27538 -3.26997
H  -0.10699 -3.76039 -2.61215
C   0.24991  3.17915 -0.34034
C   1.51142  3.32889  0.27702
H   1.99962  2.44680  0.71006
C   2.13922  4.58312  0.34845
H   3.11520  4.67435  0.83973
C   1.51609  5.71965 -0.19232
H   2.00268  6.69924 -0.13331
C   0.25973  5.58703 -0.80944
H  -0.23795  6.46604 -1.23504
C  -0.36106  4.33171 -0.88895
H  -1.33734  4.24247 -1.37975
C  -2.25335  1.77315 -0.01247
C  -2.59180  2.67911  1.01987
C  -3.28969  1.00070 -0.58828
C  -3.91732  2.79856  1.46620
C  -4.61502  1.12060 -0.14350
C  -4.93566  2.01939  0.88990
H  -1.80877  3.29724  1.47152
H  -3.04308  0.29621 -1.39117
H  -4.15653  3.50466  2.26995
H  -5.40278  0.51954 -0.61255
H  -5.97015  2.11764  1.23657
H   0.37866  0.96947  0.66927
```

***cis*-III**
BP86
SCF =   -1408.50942200
H(0 K)=   -1407.966356
H(298 K)=   -1407.928127
G(298 K)=   -1408.038646
SCF(C6H6) =   -1408.51780873
SCF(D3BJ) =   -1408.67267566
SCF(BS2) =   -3081.30704223
Low Freq. = 12.5832cm-1, 21.1968cm-1
B97D
SCF(C6H6,BS2)= -3080.83921310

67
***cis*-III**
```
Ni  0.56546  0.42413 -0.11270
P  -1.42753  1.00547 -0.88711
N   3.29573  0.24526  1.08964
N   3.27581  1.33855 -0.77242
N  -0.34560 -2.23034  0.88241
N   0.72616 -2.42460 -0.97966
C   2.44237  0.63764  0.07819
C   4.61067  0.69494  0.88448
C   4.59729  1.38588 -0.30476
C   2.85656 -0.49185  2.26636
H   1.81548 -0.79522  2.08847
H   2.90184  0.13947  3.17108
H   3.48098 -1.38796  2.42272
C   2.80915  1.98134 -1.99592
H   1.71127  1.89144 -2.01239
H   3.23760  1.49615 -2.89058
H   3.08483  3.04928 -2.00097
C   5.72335  0.41388  1.84286
H   5.51861  0.82313  2.84932
H   6.65911  0.87157  1.48580
H   5.91017 -0.66952  1.96259
C   5.69447  2.08031 -1.04610
H   5.83856  1.66431 -2.06024
H   6.64834  1.97418 -0.50623
H   5.49825  3.16196 -1.16241
C   0.31649 -1.47770 -0.06402
C  -0.36649 -3.59452  0.56051
C   0.31713 -3.72084 -0.62667
C   0.62065 -4.92353 -1.46147
H   0.19404 -4.84263 -2.47827
H   0.19542 -5.82688 -0.99698
H   1.70776 -5.09107 -1.57619
C  -1.06257 -4.61103  1.40685
H  -0.66655 -4.63852  2.43858
H  -0.93961 -5.61765  0.97747
```

```
H  -2.14616 -4.40440  1.47339
C  -1.01646 -1.64866  2.04068
H  -0.57633 -2.02503  2.98117
H  -2.09379 -1.87852  2.01748
H  -0.88651 -0.55683  1.97381
C   1.44943 -2.09061 -2.19933
H   1.66323 -1.01239 -2.16092
H   0.83966 -2.30497 -3.09404
H   2.39531 -2.65581 -2.26444
C  -2.13584  2.61505 -0.23790
C  -1.53849  3.40207  0.77282
H  -0.64189  3.02225  1.27372
C  -2.07230  4.65177  1.13424
H  -1.58514  5.24201  1.91949
C  -3.22961  5.13755  0.50675
H  -3.64855  6.10908  0.79144
C  -3.84725  4.36286 -0.49131
H  -4.75002  4.73091 -0.99249
C  -3.30274  3.12474 -0.86112
H  -3.78229  2.53903 -1.65416
C  -2.80287 -0.16855 -0.39296
C  -3.79582  0.11116  0.57856
C  -2.86384 -1.42860 -1.03954
C  -4.78948 -0.82859  0.89709
C  -3.84665 -2.37471 -0.71382
C  -4.81847 -2.08210  0.26106
H  -3.78653  1.07886  1.09063
H  -2.12429 -1.66266 -1.81494
H  -5.54436 -0.58054  1.65291
H  -3.86695 -3.33820 -1.23715
H  -5.59625 -2.81314  0.50779
H   0.55790  1.90108 -0.02601
```

TS(II-trans-III)

```
BP86
SCF  =       -1408.48298255
H(0 K)=      -1407.941780
H(298 K)=    -1407.903714
G(298 K)=    -1408.014208
SCF(C6H6) =         -1408.48936666
SCF(D3BJ) =         -1408.64042575
SCF(BS2) =          -3081.28179470
Low Freq. = -125.8845cm-1,
15.1840cm-1
B97D
SCF(C6H6,BS2)=  -3080.80628365
```

67

TS(II-trans-III)

```
Ni -0.19586 -0.59069 -0.23345
P   0.21211  1.48842  0.08260
N  -2.64161 -2.18332 -0.83600
N  -3.01962 -0.79665  0.78021
N   2.57470 -1.62257 -0.89021
N   1.75563 -2.57394  0.86444
C  -2.00782 -1.20715 -0.07916
C  -3.98408 -2.35960 -0.47343
C  -4.22270 -1.48102  0.55754
C  -1.98004 -2.94251 -1.88857
H  -0.89714 -2.78946 -1.76927
H  -2.28290 -2.58978 -2.89067
H  -2.22310 -4.01466 -1.79745
C  -2.86806  0.24874  1.78523
H  -1.81250  0.56242  1.78567
H  -3.14516 -0.12976  2.78435
H  -3.49433  1.12337  1.54074
C  -4.88733 -3.34573 -1.14289
H  -4.96713 -3.16392 -2.23037
H  -5.90266 -3.28044 -0.72128
H  -4.54293 -4.38869 -1.01153
C  -5.46229 -1.22222  1.35295
H  -5.32510 -1.44994  2.42644
H  -6.29025 -1.84923  0.98643
H  -5.78716 -0.16797  1.28385
C   1.44111 -1.63225 -0.10083
C   3.55653 -2.51283 -0.43178
C   3.03712 -3.11735  0.69002
C   3.61791 -4.14804  1.60477
H   3.67389 -3.79225  2.65004
H   4.64098 -4.40620  1.28887
H   3.02872 -5.08410  1.61030
C   4.87662 -2.68470 -1.11275
H   4.76427 -3.03574 -2.15518
H   5.49043 -3.42673 -0.57833
H   5.45076 -1.74079 -1.14743
C   2.74633 -0.74290 -2.04129
H   2.87983 -1.32893 -2.96738
H   3.61836 -0.08371 -1.89708
H   1.83689 -0.12889 -2.11775
C   0.85081 -2.92148  1.95153
H  -0.13250 -2.49189  1.69830
H   1.19488 -2.50080  2.91308
H   0.76336 -4.01692  2.05037
H  -0.29008 -0.71898 -1.67169
C  -0.94492  2.93102 -0.08292
C  -2.14543  2.73565 -0.81050
H  -2.29512  1.77848 -1.32409
C  -3.12030  3.74147 -0.88416
H  -4.03587  3.56939 -1.46222
C  -2.91977  4.97251 -0.23526
H  -3.67735  5.76127 -0.29728
C  -1.73468  5.18269  0.49129
H  -1.56945  6.13645  1.00587
C  -0.76092  4.17582  0.56962
H   0.15254  4.35208  1.14758
C   1.87850  2.29547  0.17728
C   2.22900  3.47999 -0.51985
C   2.90067  1.62940  0.90046
C   3.54076  3.97518 -0.48997
C   4.21327  2.12262  0.92512
C   4.54166  3.30093  0.23183
H   1.46414  4.01178 -1.09545
H   2.64731  0.71429  1.44775
H   3.78499  4.89219 -1.03880
H   4.98169  1.59255  1.50011
H   5.56464  3.69173  0.25641
```

trans-III

```
BP86
SCF  =       -1408.51299200
H(0 K)=      -1407.969969
H(298 K)=    -1407.931794
G(298 K)=    -1408.042100
SCF(C6H6) =         -1408.52017499
```

SCF(D3BJ) = -1408.68040133
SCF(BS2) = -3081.30972497
Low Freq. = 9.5167cm-1, 15.4151cm-1
B97D
SCF(C6H6,BS2)= -3080.84345933

67
trans-III

```
Ni   0.02363 -1.14099  0.02235
P    0.01420  0.88863 -1.03269
N   -2.65675 -2.09990 -0.63080
N   -2.70554 -0.78765  1.08394
N    2.80676 -1.41275 -0.96392
N    2.67516 -1.47538  1.19146
C   -1.84028 -1.32453  0.16153
C   -3.99877 -2.03812 -0.22261
C   -4.02802 -1.20291  0.87008
C   -2.15657 -2.88459 -1.75201
H   -1.06028 -2.92617 -1.65051
H   -2.41769 -2.41916 -2.71845
H   -2.56780 -3.90740 -1.71971
C   -2.28911  0.13524  2.13245
H   -1.21779  0.33410  1.97772
H   -2.44629 -0.30819  3.13134
H   -2.84268  1.08472  2.05320
C   -5.09415 -2.77722 -0.92184
H   -5.17373 -2.49235 -1.98707
H   -6.06355 -2.55775 -0.44788
H   -4.95007 -3.87311 -0.88602
C   -5.16519 -0.72905  1.71616
H   -5.02842 -0.98803  2.78210
H   -6.10873 -1.18840  1.38238
H   -5.28573  0.36784  1.65411
C    1.89749 -1.31357  0.06570
C    4.11220 -1.63165 -0.49421
C    4.02914 -1.66368  0.87768
C    5.08625 -1.83719  1.92026
H    5.14234 -0.96836  2.60149
H    6.07383 -1.94911  1.44637
H    4.91616 -2.73379  2.54468
C    5.28788 -1.76884 -1.40718
H    5.17114 -2.61119 -2.11345
H    6.20371 -1.95166 -0.82375
H    5.45530 -0.85651 -2.00881
C    2.44642 -1.36993 -2.37722
H    2.51477 -2.37481 -2.83164
H    3.11284 -0.68138 -2.92130
H    1.41439 -0.99527 -2.45198
C    2.12655 -1.46353  2.53971
H    1.03206 -1.54691  2.44036
H    2.38226 -0.52932  3.07017
H    2.50696 -2.32178  3.11859
H    0.04393 -2.56669  0.48721
C   -1.45208  1.95625 -0.59383
C   -2.72226  1.57960 -1.10244
H   -2.80009  0.68329 -1.72946
C   -3.87501  2.32828 -0.82751
H   -4.83948  2.00721 -1.23889
C   -3.79562  3.49690 -0.04704
H   -4.69282  4.09053  0.15927
C   -2.54261  3.90091  0.44412
H   -2.45847  4.81525  1.04379
C   -1.39050  3.14323  0.17810
H   -0.42716  3.47613  0.57774
C    1.41901  1.99196 -0.44161
C    2.04859  2.84473 -1.37772
C    1.90654  2.00455  0.88623
C    3.12220  3.67228 -1.00760
C    2.97962  2.82987  1.26327
C    3.59330  3.66570  0.31541
H    1.68420  2.85362 -2.41162
H    1.42741  1.35704  1.62879
H    3.59114  4.32402 -1.75384
H    3.33542  2.82457  2.30051
H    4.43093  4.30901  0.60693
```

^{3}III
BP86
SCF = -1408.47340686
H(0 K)= -1407.931915
H(298 K)= -1407.893602
G(298 K)= -1408.005690
SCF(C6H6) = -1408.48057459
SCF(D3BJ) = -1408.63608199
SCF(BS2) = -3081.26718741
Low Freq. = 11.4930cm-1, 15.2332cm-1
B97D
SCF(C6H6,BS2)= -3080.79875809

67
^{3}III

```
Ni   0.23283 -0.78597  0.22281
P   -0.12224  1.23026 -0.84082
N    2.91442 -1.83537  0.97196
N    3.06467 -0.93353 -0.98408
N   -2.66464 -1.20104  1.00576
N   -2.10074 -2.41045 -0.69042
C    2.14900 -1.18162  0.02195
C    4.24736 -1.98713  0.57433
C    4.34511 -1.40752 -0.67057
C    2.37526 -2.33977  2.23101
H    1.41384 -1.82019  2.39557
H    3.06869 -2.11503  3.05793
H    2.21065 -3.43070  2.18453
C    2.75231 -0.26297 -2.24206
H    1.68691  0.01409 -2.23239
H    2.95579 -0.93127 -3.09705
H    3.35276  0.65531 -2.35181
C    5.27932 -2.66399  1.41889
H    5.45330 -2.13166  2.37215
H    6.24169 -2.70586  0.88530
H    4.99284 -3.70119  1.67144
C    5.51670 -1.24887 -1.58594
H    5.34734 -1.72931 -2.56708
H    6.41354 -1.70889 -1.14255
H    5.75006 -0.18585 -1.77921
C   -1.58895 -1.48165  0.19583
C   -3.80990 -1.91433  0.63326
C   -3.45134 -2.69188 -0.44579
C   -4.24114 -3.66730 -1.25867
H   -4.28184 -3.38422 -2.32666
H   -5.27812 -3.71808 -0.89160
H   -3.82236 -4.68954 -1.20738
```

C -5.12003 -1.76256 1.33687
H -5.05945 -2.05865 2.40016
H -5.88748 -2.39244 0.86040
H -5.47532 -0.71690 1.30611
C -2.63062 -0.22040 2.08873
H -3.04732 -0.66147 3.00989
H -3.20424 0.67939 1.81180
H -1.57206 0.04634 2.24191
C -1.31435 -3.01260 -1.75910
H -0.29494 -2.60036 -1.68530
H -1.73567 -2.76552 -2.74887
H -1.27208 -4.11012 -1.64968
H 0.29407 -0.35556 1.72672
C 1.01538 2.56692 -0.19481
C 1.92695 2.32003 0.85699
H 1.88150 1.35069 1.36651
C 2.85121 3.30170 1.25461
H 3.54700 3.09107 2.07567
C 2.87702 4.55260 0.61760
H 3.59572 5.31840 0.93013
C 1.96917 4.81706 -0.42368
H 1.97857 5.79114 -0.92642
C 1.05475 3.83322 -0.82880
H 0.35980 4.04348 -1.65029
C -1.78806 1.96495 -0.49380
C -2.02667 3.04821 0.39061
C -2.91075 1.39650 -1.15143
C -3.32479 3.53537 0.60583
C -4.20855 1.87587 -0.92736
C -4.42595 2.95200 -0.04612
H -1.18257 3.50846 0.91375
H -2.75242 0.56848 -1.85282
H -3.47833 4.37543 1.29354
H -5.05430 1.41958 -1.45531
H -5.43797 3.33603 0.12181

(ii) 2nd P-H Activation

TS(cis-trans-III)
BP86
SCF = -1408.48298255
H(0 K)= -1407.941780
H(298 K)= -1407.903714
G(298 K)= -1408.014208
SCF(C6H6) = -1408.48936666
SCF(D3BJ) = -1408.64042575
SCF(BS2) = -3081.28179470
Low Freq. = -125.8845cm-1, 15.1840cm-1
B97D
SCF(C6H6,BS2)= -3080.80628365

67
TS(cis-trans-III)
Ni -0.19586 -0.59069 -0.23345
P 0.21211 1.48842 0.08260
N -2.64161 -2.18332 -0.83600
N -3.01962 -0.79665 0.78021
N 2.57470 -1.62257 -0.89021
N 1.75563 -2.57394 0.86444
C -2.00782 -1.20715 -0.07916
C -3.98408 -2.35960 -0.47343
C -4.22270 -1.48102 0.55754
C -1.98004 -2.94251 -1.88857
H -0.89714 -2.78946 -1.76927
H -2.28290 -2.58978 -2.89067
H -2.22310 -4.01466 -1.79745
C -2.86806 0.24874 1.78523
H -1.81250 0.56242 1.78567
H -3.14516 -0.12976 2.78435
H -3.49433 1.12337 1.54074
C -4.88733 -3.34573 -1.14289
H -4.96713 -3.16392 -2.23037
H -5.90266 -3.28044 -0.72128
H -4.54293 -4.38869 -1.01153
C -5.46229 -1.22222 1.35295
H -5.32510 -1.44994 2.42644
H -6.29025 -1.84923 0.98643
H -5.78716 -0.16797 1.28385
C 1.44111 -1.63225 -0.10083
C 3.55653 -2.51283 -0.43178
C 3.03712 -3.11735 0.69002
C 3.61791 -4.14804 1.60477
H 3.67389 -3.79225 2.65004
H 4.64098 -4.40620 1.28887
H 3.02872 -5.08410 1.61030
C 4.87662 -2.68470 -1.11275
H 4.76427 -3.03574 -2.15518
H 5.49043 -3.42673 -0.57833
H 5.45076 -1.74079 -1.14743
C 2.74633 -0.74290 -2.04129
H 2.87983 -1.32893 -2.96738
H 3.61836 -0.08371 -1.89708
H 1.83689 -0.12889 -2.11775
C 0.85081 -2.92148 1.95153
H -0.13250 -2.49189 1.69830
H 1.19488 -2.50080 2.91308
H 0.76336 -4.01692 2.05037
H -0.29008 -0.71898 -1.67169
C -0.94492 2.93102 -0.08292
C -2.14543 2.73565 -0.81050
H -2.29512 1.77848 -1.32409
C -3.12030 3.74147 -0.88416
H -4.03587 3.56939 -1.46222
C -2.91977 4.97251 -0.23526
H -3.67735 5.76127 -0.29728
C -1.73468 5.18269 0.49129
H -1.56945 6.13645 1.00587
C -0.76092 4.17582 0.56962
H 0.15254 4.35208 1.14758
C 1.87850 2.29547 0.17728
C 2.22900 3.47999 -0.51985
C 2.90067 1.62940 0.90046
C 3.54076 3.97518 -0.48997
C 4.21327 2.12262 0.92512
C 4.54166 3.30093 0.23183
H 1.46414 4.01178 -1.09545
H 2.64731 0.71429 1.44775
H 3.78499 4.89219 -1.03880
H 4.98169 1.59255 1.50011
H 5.56464 3.69173 0.25641

TS(II-IV)
BP86

SCF = -1878.94984696
H(0 K)= -1878.222415
H(298 K)= -1878.171533
G(298 K)= -1878.313387
SCF(C6H6) = -1878.95687638
SCF(D3BJ) = -1879.19821333
SCF(BS2) = -3886.73751653
Low Freq. = -54.6172cm-1, 7.3129cm-1
B97D
SCF(C6H6,BS2)= -3885.91050410

91
TS(II-IV)

```
C  -3.50035 -2.37177  1.75222
N  -2.60509 -1.97776  0.74995
C  -1.55109 -1.22251  1.23724
N  -1.82501 -1.18143  2.59389
C  -3.00296 -1.86685  2.93022
C  -1.02833 -0.44825  3.57197
C  -2.82388 -2.25417 -0.66563
Ni -0.19169 -0.33919  0.09392
P  -1.38400  1.69171  0.02453
P   1.90320  0.18715  1.54872
C   0.56901 -1.69422 -1.09723
N   0.75253 -1.71202 -2.47269
C   1.31518 -2.91469 -2.92660
C   1.50593 -3.69494 -1.81110
N   1.04244 -2.94188 -0.72432
C   0.41428 -0.61477 -3.37070
C   1.11446 -3.40291  0.65778
H   0.13420  0.25187 -2.75518
H  -0.43196 -0.88320 -4.02736
H   1.28112 -0.34996 -3.99931
H   2.15802 -3.40339  1.01615
H   0.69704 -4.42029  0.74483
H   0.52196 -2.70312  1.26521
C  -4.73329 -3.16900  1.47015
C  -3.51538 -1.97005  4.33150
H  -0.06646 -0.18937  3.10604
H  -0.84179 -1.07554  4.45953
H  -1.54418  0.47529  3.88771
H  -2.94693 -3.33803 -0.83417
H  -1.93844 -1.88860 -1.20493
H  -3.71814 -1.72064 -1.02987
C   2.41830  1.96670  1.80232
H   1.26203  0.43283  0.12771
H  -0.65429  0.59943 -1.03867
C   3.45646 -0.55717  0.81205
C  -3.11454  1.31210 -0.59334
C  -0.84970  3.01373 -1.20060
C   1.60786 -3.18557 -4.36803
C   2.08908 -5.06404 -1.66439
H  -2.81254 -2.50576  4.99648
H  -3.69712 -0.97763  4.78273
H  -4.46875 -2.52115  4.34873
H  -5.40379 -2.64367  0.76589
H  -4.50315 -4.15689  1.03000
H  -5.29696 -3.34520  2.39977
H   2.40136 -5.45371 -2.64599
H   2.97850 -5.06396 -1.00848
H   1.36669 -5.78408 -1.23728
H   2.02940 -4.19590 -4.48796
H   0.70000 -3.12859 -4.99644
H   2.33953 -2.47080 -4.78786
C  -3.48892  1.30114 -1.95762
C  -4.80171  0.98973 -2.34451
C  -5.77057  0.67190 -1.37582
C  -5.41508  0.68071 -0.01602
C  -4.10294  1.00411  0.36831
H  -2.74471  1.55389 -2.72044
H  -5.07010  0.99311 -3.40734
H  -6.79576  0.43179 -1.67804
H  -6.16534  0.45220  0.74964
H  -3.83594  1.02020  1.43126
C  -1.71435  4.07409 -1.56123
C  -1.26151  5.13261 -2.36242
C   0.07199  5.16912 -2.80703
C   0.94603  4.13312 -2.44136
C   0.48872  3.06653 -1.65054
H  -2.75422  4.06648 -1.21571
H  -1.95365  5.93679 -2.63772
H   0.42582  5.99963 -3.42728
H   1.99057  4.15271 -2.77313
H   1.17969  2.26368 -1.36615
C   1.41429  2.89268  2.17488
C   1.73998  4.22685  2.45964
C   3.07422  4.66248  2.39480
C   4.07846  3.74971  2.03314
C   3.75655  2.41548  1.73744
H   0.36722  2.57011  2.21848
H   0.94407  4.92971  2.73002
H   3.32810  5.70350  2.62141
H   5.12327  4.07666  1.97732
H   4.55209  1.71855  1.45485
C   3.85764 -0.35787 -0.52821
C   5.02379 -0.95946 -1.02729
C   5.81250 -1.77204 -0.19466
C   5.42806 -1.97979  1.13978
C   4.25790 -1.37927  1.63556
H   3.24812  0.27554 -1.18134
H   5.32138 -0.78992 -2.06834
H   6.72369 -2.23884 -0.58437
H   6.03977 -2.60793  1.79700
H   3.95932 -1.54240  2.67801
```

IV
BP86
SCF = -1878.94992490
H(0 K)= -1878.222103
H(298 K)= -1878.170506
G(298 K)= -1878.313831
SCF(C6H6) = -1878.95694301
SCF(D3BJ) = -1879.19895483
SCF(BS2) = -3886.73775298
Low Freq. = 9.4388cm-1, 10.8253cm-1
B97D
SCF(C6H6,BS2)= -3885.91060028

91
IV

```
C  -3.41848 -2.44053  1.73749
N  -2.52381 -2.01947  0.74574
C  -1.49097 -1.24369  1.24645
```

```
N  -1.77765 -1.21854  2.60086
C  -2.94275 -1.93197  2.92272
C  -1.01004 -0.47091  3.59173
C  -2.72277 -2.30048 -0.67180
Ni -0.11035 -0.33214  0.13164
P  -1.43189  1.65685 -0.00749
P   1.85106  0.20005  1.54761
C   0.63574 -1.66350 -1.10661
N   0.84053 -1.63971 -2.47785
C   1.42642 -2.82154 -2.95721
C   1.60863 -3.63107 -1.86141
N   1.11794 -2.91494 -0.76132
C   0.50740 -0.51792 -3.34694
C   1.17665 -3.41265  0.60886
H  -0.30673 -0.78275 -4.04439
H   1.38918 -0.20833 -3.93301
H   0.18449  0.31971 -2.71318
H   0.57826 -2.72951  1.22914
H   2.21691 -3.42129  0.97661
H   0.76010 -4.43260  0.66513
C  -4.63049 -3.26388  1.44007
C  -3.46612 -2.05495  4.31841
H  -1.54062  0.45108  3.88706
H  -0.03864 -0.20885  3.14784
H  -0.84213 -1.09002  4.48842
H  -1.84034 -1.91717 -1.20326
H  -3.62544 -1.78785 -1.04498
H  -2.82040 -3.38684 -0.84066
C   2.31236  1.98618  1.85164
H   1.25706  0.49431  0.07075
H  -0.76227  0.52717 -1.00425
C   3.43928 -0.47637  0.81282
C  -3.16413  1.23342 -0.59750
C  -0.93232  2.96689 -1.25693
C   1.74682 -3.04583 -4.40068
C   2.20635 -4.99664 -1.74363
H  -3.67226 -1.06955  4.77446
H  -4.40821 -2.62537  4.32282
H  -2.75892 -2.58090  4.98653
H  -5.30762 -2.74884  0.73457
H  -4.37439 -4.24303  0.99474
H  -5.19684 -3.45989  2.36406
H   1.48674 -5.73656 -1.34687
H   2.53745 -5.35548 -2.73079
H   3.08622 -5.00409 -1.07499
H   2.47363 -2.30762 -4.78698
H   2.18657 -4.04550 -4.54264
H   0.84865 -2.98483 -5.04252
C  -3.55026  1.17197 -1.95710
C  -4.86135  0.82759 -2.32091
C  -5.81511  0.52475 -1.33242
C  -5.44729  0.58165  0.02254
C  -4.13710  0.93970  0.38336
H  -2.81703  1.41072 -2.73503
H  -5.14011  0.79254 -3.38043
H  -6.83881  0.25786 -1.61668
H  -6.18588  0.36319  0.80221
H  -3.85973  0.99372  1.44233
C  -1.83067  3.97618 -1.67660
C  -1.39884  5.02860 -2.49746
C  -0.05578  5.10890 -2.90535
C   0.85054  4.12411 -2.48109
C   0.41630  3.06468 -1.66821
H  -2.87915  3.93450 -1.36154
H  -2.11592  5.79338 -2.81745
H   0.28064  5.93417 -3.54200
H   1.90290  4.17922 -2.78258
H   1.13299  2.30472 -1.33399
C   3.64273  2.46416  1.85083
C   3.92517  3.79696  2.18933
C   2.88942  4.68114  2.53345
C   1.56317  4.21701  2.53586
C   1.27632  2.88445  2.20527
H   4.46338  1.79029  1.58445
H   4.96444  4.14571  2.18124
H   3.11280  5.72124  2.79393
H   0.74277  4.89707  2.79150
H   0.23526  2.54064  2.19719
C   3.86381 -0.22148 -0.51074
C   5.05780 -0.77133 -1.00342
C   5.85216 -1.58902 -0.18113
C   5.44516 -1.85263  1.13649
C   4.24777 -1.30198  1.62569
H   3.25024  0.41558 -1.15681
H   5.37277 -0.55751 -2.03122
H   6.78483 -2.01601 -0.56572
H   6.06073 -2.48483  1.78626
H   3.93247 -1.50652  2.65591
```

IVb

```
BP86
SCF  =       -1878.97142242
H(0 K)=      -1878.241327
H(298 K)=    -1878.190119
G(298 K)=    -1878.331240
SCF(C6H6) =        -1878.97693232
SCF(D3BJ) =        -1879.22418687
SCF(BS2) =         -3886.76655939
Low Freq. = 9.5569cm-1, 14.6272cm-1
B97D
SCF(C6H6,BS2)=  -3885.94056716
```

91

IVb

```
C   4.52362  0.41472 -1.54948
C   3.29373  0.72646 -0.93175
C   3.27841  1.67955  0.10403
C   4.46406  2.31574  0.51284
C   5.68192  1.99976 -0.10859
C   5.70864  1.04630 -1.14165
P   1.65406 -0.07584 -1.45962
C   2.29047 -1.80137 -1.84919
C   2.42741 -2.27155 -3.17511
C   2.81847 -3.59591 -3.43601
C   3.09290 -4.47227 -2.37401
C   2.97012 -4.01487 -1.05005
C   2.56657 -2.69701 -0.79004
Ni -0.22730  0.31725 -0.38634
C  -0.16451  2.19045  0.16886
N  -0.13805  2.82931  1.40925
C  -0.11749  4.22963  1.30636
C  -0.13125  4.51505 -0.03776
N  -0.16865  3.27966 -0.69970
C  -0.13216  2.12390  2.68084
```

C	-0.09157	5.13362	2.49757
C	-0.11087	5.82626	-0.75685
C	-0.16942	3.13927	-2.14960
C	-1.86435	0.03622	-1.43963
N	-3.12933	0.53455	-1.14058
C	-4.10750	0.14253	-2.06418
C	-3.46466	-0.63073	-3.00028
N	-2.12018	-0.68771	-2.60133
C	-5.54515	0.53582	-1.94248
C	-3.43682	1.31214	0.05321
C	-1.11038	-1.44297	-3.32669
C	-3.98574	-1.31434	-4.22445
P	-0.45389	-1.32703	1.05288
C	0.65239	-1.77563	2.52720
C	0.35157	-2.80511	3.44583
C	1.23898	-3.11463	4.48801
C	2.44507	-2.40386	4.62489
C	2.75748	-1.38205	3.71552
C	1.86223	-1.06830	2.67699
C	-2.15123	-1.58701	1.80822
C	-2.50247	-1.04352	3.06455
C	-3.82131	-1.11791	3.53966
C	-4.82064	-1.73238	2.76502
C	-4.48384	-2.28182	1.51544
C	-3.16354	-2.21009	1.04357
H	0.74121	2.41277	3.29100
H	-0.07427	1.05015	2.45081
H	-1.05198	2.32759	3.25744
H	-0.43095	2.09091	-2.36541
H	0.82422	3.36634	-2.57579
H	-0.91522	3.81461	-2.60267
H	-1.49794	-2.43771	-3.60236
H	-0.24529	-1.56559	-2.66118
H	-0.79138	-0.92002	-4.24648
H	-3.97717	2.23668	-0.21512
H	-2.47618	1.56392	0.52458
H	-4.04800	0.72255	0.75814
H	-3.47180	-0.97560	-5.14305
H	-3.87008	-2.41328	-4.17485
H	-5.05954	-1.10344	-4.35069
H	-5.68489	1.63235	-1.97760
H	-6.13270	0.10184	-2.76703
H	-5.98922	0.18169	-0.99410
H	-0.05275	6.65736	-0.03628
H	0.75776	5.91375	-1.43510
H	-1.01860	5.98328	-1.36913
H	-0.07844	6.18768	2.17787
H	-0.97666	4.99583	3.14631
H	0.80122	4.96883	3.12906
H	-1.73186	-0.57423	3.68630
H	-4.06866	-0.69919	4.52192
H	-5.84910	-1.79287	3.13684
H	-5.25035	-2.77854	0.90956
H	-2.91251	-2.64120	0.06774
H	-0.58538	-3.36418	3.34607
H	0.99196	-3.91569	5.19406
H	3.13770	-2.64932	5.43745
H	3.69731	-0.82693	3.81111
H	2.10061	-0.27101	1.96111
H	2.23321	-1.59027	-4.01232
H	2.91948	-3.93875	-4.47216
H	3.40290	-5.50328	-2.57529
H	3.18728	-4.68896	-0.21407
H	2.47289	-2.35569	0.24718
H	2.31985	1.91553	0.58029
H	4.43548	3.05705	1.31981
H	6.60807	2.49069	0.21031
H	6.65673	0.79237	-1.62930
H	4.55497	-0.33438	-2.34854
H	-0.39296	-2.64703	0.44919
H	1.75156	0.36833	-2.84555

TS(IV-cis-V)

BP86
SCF = -1878.93278252
H(0 K)= -1878.204934
H(298 K)= -1878.154284
G(298 K)= -1878.292494
SCF(C6H6) = -1878.94164735
SCF(D3BJ) = -1879.19445041
SCF(BS2) = -3886.71539074
Low Freq. = -680.4455cm-1, 9.8821cm-1
B97D
SCF(C6H6,BS2)= -3885.89700771

91

TS(IV-cis-V)

C	-3.37549	-2.66685	0.96097
N	-2.38709	-2.18329	0.09387
C	-1.53715	-1.29112	0.71300
N	-2.02002	-1.24883	2.00299
C	-3.14231	-2.07218	2.17848
C	-1.45733	-0.43328	3.07718
C	-2.31407	-2.56102	-1.31333
Ni	-0.08966	-0.27126	-0.17191
P	-1.45186	1.60277	0.11241
P	1.64862	0.34492	1.42672
C	0.99313	-1.85058	-0.61590
N	1.60087	-2.15011	-1.81739
C	2.27955	-3.37803	-1.77275
C	2.08415	-3.88107	-0.50930
N	1.30332	-2.93689	0.17367
C	1.60770	-1.27648	-2.98844
C	0.88187	-3.10112	1.56396
H	1.87979	-1.86437	-3.87652
H	2.33429	-0.45904	-2.85329
H	0.60727	-0.84044	-3.12547
H	0.83576	-2.10741	2.03336
H	1.62717	-3.70972	2.09723
H	-0.10155	-3.59811	1.62705
C	-4.44397	-3.61596	0.52260
C	-3.86762	-2.19557	3.48033
H	-2.06651	0.47101	3.24348
H	-0.43726	-0.13629	2.79017
H	-1.41373	-1.02111	4.00839
H	-1.49312	-1.98372	-1.76105
H	-3.25616	-2.30604	-1.82491
H	-2.11704	-3.64217	-1.41701
C	1.81785	2.15892	1.82056
H	0.56335	0.51795	-1.21263
H	-0.52427	-0.06576	-1.59176
C	3.27428	0.01837	0.53745
C	-3.10294	1.08313	-0.64458

C -0.95541 2.93109 -1.11518
C 3.06422 -3.92998 -2.92027
C 2.57198 -5.14909 0.11601
H -4.24916 -1.22123 3.83497
H -4.73137 -2.86952 3.37079
H -3.22673 -2.60705 4.28181
H -5.08760 -3.17052 -0.25763
H -4.02884 -4.55500 0.11339
H -5.09004 -3.88270 1.37334
H 1.74448 -5.77510 0.49604
H 3.12407 -5.74735 -0.62523
H 3.25921 -4.95946 0.96098
H 3.88731 -3.25574 -3.21769
H 3.51491 -4.89398 -2.63766
H 2.43940 -4.11043 -3.81440
C -3.30365 0.77791 -2.01130
C -4.56502 0.39308 -2.49136
C -5.65842 0.29388 -1.61053
C -5.47942 0.59613 -0.25179
C -4.21535 0.99372 0.22035
H -2.45679 0.85170 -2.70266
H -4.69855 0.17262 -3.55709
H -6.64388 -0.00366 -1.98576
H -6.32718 0.53837 0.44048
H -4.08663 1.25290 1.27809
C -1.91760 3.70960 -1.80286
C -1.53288 4.79723 -2.60358
C -0.17886 5.14456 -2.72996
C 0.78626 4.39623 -2.03486
C 0.40336 3.30720 -1.23850
H -2.98063 3.46538 -1.71365
H -2.30067 5.37546 -3.13072
H 0.12057 5.99233 -3.35573
H 1.84653 4.66412 -2.10624
H 1.16359 2.75071 -0.68197
C 3.00448 2.91211 1.62797
C 3.10398 4.24090 2.06830
C 2.02394 4.86671 2.71142
C 0.84101 4.13665 2.91543
C 0.74163 2.80642 2.48541
H 3.86651 2.45146 1.13661
H 4.03885 4.79042 1.90572
H 2.10269 5.90530 3.04990
H -0.01546 4.60568 3.41341
H -0.19274 2.26351 2.65565
C 3.64155 0.62075 -0.69017
C 4.86894 0.33037 -1.30793
C 5.75800 -0.58694 -0.71928
C 5.41191 -1.19729 0.49596
C 4.18883 -0.88857 1.11833
H 2.95310 1.33146 -1.16126
H 5.13406 0.82320 -2.25096
H 6.71567 -0.81450 -1.20037
H 6.10227 -1.90376 0.97140
H 3.93757 -1.34494 2.08298

Int(IV-cis-V)
BP86
SCF = -1878.94059941
H(0 K)= -1878.211599
H(298 K)= -1878.160478
G(298 K)= -1878.298951
SCF(C6H6) = -1878.94979666
SCF(D3BJ) = -1879.20221230
SCF(BS2) = -3886.72466528
Low Freq. = 11.1808cm-1, 14.6698cm-1
B97D
SCF(C6H6,BS2)= -3885.91055984

91
Int(IV-cis-V)
C -3.80610 -2.26531 0.64848
N -2.74429 -1.85485 -0.16963
C -1.78821 -1.14568 0.52568
N -2.28005 -1.14925 1.81621
C -3.50802 -1.82128 1.91482
C -1.61554 -0.52960 2.95986
C -2.71367 -2.07938 -1.60903
Ni -0.13672 -0.32883 -0.09444
P -1.19912 1.74056 0.31913
P 1.74204 0.23388 1.19241
C 0.74513 -2.04935 -0.43899
N 1.24615 -2.56074 -1.62252
C 1.72205 -3.87482 -1.47316
C 1.51938 -4.20935 -0.15671
N 0.93276 -3.08839 0.44827
C 1.32309 -1.82194 -2.87960
C 0.56000 -3.03811 1.86024
H 1.85364 -2.43263 -3.62236
H 1.87642 -0.88365 -2.72957
H 0.31722 -1.58577 -3.26400
H 0.72581 -2.01150 2.22160
H 1.20356 -3.72470 2.42998
H -0.49514 -3.32819 2.00201
C -4.99426 -3.00539 0.12418
C -4.25684 -1.95841 3.20173
H -2.19928 0.32755 3.33391
H -0.62928 -0.17247 2.62826
H -1.48907 -1.26576 3.77243
H -1.72270 -1.76929 -1.96771
H -3.48351 -1.47166 -2.11320
H -2.87213 -3.14688 -1.83650
C 2.12772 2.00839 1.62349
H 0.27260 0.35728 -1.63962
H -0.45566 0.02731 -1.79691
C 3.32818 -0.26053 0.30021
C -2.87784 1.53194 -0.52638
C -0.51319 3.04573 -0.84701
C 2.33898 -4.66616 -2.58270
C 1.83823 -5.46907 0.58400
H -4.51904 -0.97596 3.63464
H -5.19594 -2.50974 3.03887
H -3.67867 -2.50780 3.96740
H -5.54197 -2.40987 -0.62859
H -4.71721 -3.96546 -0.34865
H -5.69503 -3.23113 0.94303
H 0.94601 -5.90929 1.06478
H 2.24524 -6.22226 -0.10830
H 2.59483 -5.30867 1.37405
H 3.26664 -4.20023 -2.96240
H 2.60530 -5.67145 -2.22098
H 1.65454 -4.79772 -3.44068
C -3.06874 1.53773 -1.92958

```
C  -4.34429  1.37290 -2.49239
C  -5.46772  1.19192 -1.66461
C  -5.30061  1.19316 -0.27000
C  -4.02243  1.36986  0.28732
H  -2.20514  1.69375 -2.58548
H  -4.46435  1.39264 -3.58221
H  -6.46455  1.07181 -2.10316
H  -6.17051  1.07838  0.38715
H  -3.90611  1.39946  1.37727
C  -1.31069  4.18108 -1.13977
C  -0.81553  5.23659 -1.91827
C   0.49864  5.19646 -2.41611
C   1.30790  4.08931 -2.12100
C   0.80508  3.02808 -1.34970
H  -2.33452  4.23542 -0.75369
H  -1.45888  6.09765 -2.13321
H   0.88705  6.02241 -3.02191
H   2.33923  4.04804 -2.48982
H   1.44694  2.17608 -1.10677
C   3.30660  2.69737  1.24642
C   3.57645  3.98913  1.72332
C   2.67359  4.63871  2.58060
C   1.49865  3.97176  2.96558
C   1.23569  2.67431  2.50451
H   4.02775  2.21569  0.57903
H   4.50123  4.49255  1.41752
H   2.88408  5.64921  2.94700
H   0.78291  4.46088  3.63621
H   0.32315  2.16584  2.83201
C   3.67590  0.14658 -1.00931
C   4.86916 -0.27914 -1.61688
C   5.74511 -1.13560 -0.92807
C   5.42103 -1.55044  0.37317
C   4.23020 -1.11167  0.97814
H   3.00407  0.81761 -1.55718
H   5.11745  0.06195 -2.62893
H   6.67559 -1.46981 -1.40008
H   6.10112 -2.21037  0.92410
H   3.99039 -1.42465  2.00110
```

TS(IV-cis-V)2

```
BP86
SCF  =       -1878.94036565
H(0 K)=      -1878.212927
H(298 K)=   -1878.161760
G(298 K)=   -1878.300226
SCF(C6H6) =        -1878.94958821
SCF(D3BJ) =        -1879.20301967
SCF(BS2) =         -3886.72421770
Low Freq. = -121.7350cm-1,
10.243cm-1
B97D
SCF(C6H6,BS2)=  -3885.91267308
```

91

TS(IV-cis-V)2

```
C  -3.86010 -2.26914  0.52240
N  -2.76289 -1.85162 -0.24551
C  -1.83865 -1.15026  0.49735
N  -2.38170 -1.17074  1.76708
C  -3.61263 -1.84323  1.80605
C  -1.75640 -0.57294  2.94412
C  -2.66651 -2.03797 -1.68896
Ni -0.15327 -0.33748 -0.00478
P  -1.21016  1.73907  0.36467
P   1.75694  0.23937  1.16510
C   0.73728 -2.04403 -0.36700
N   1.30470 -2.52698 -1.53157
C   1.77744 -3.84210 -1.38560
C   1.50833 -4.20573 -0.08857
N   0.88492 -3.09993  0.50782
C   1.45530 -1.75347 -2.76087
C   0.46132 -3.06284  1.90551
H   2.17536 -2.25932 -3.41823
H   1.84171 -0.75431 -2.51739
H   0.49452 -1.64966 -3.29324
H   0.63148 -2.04425  2.28642
H   1.06959 -3.77029  2.48849
H  -0.60376 -3.33239  2.00532
C  -5.03035 -2.99958 -0.05389
C  -4.41109 -1.99955  3.06059
H  -2.36426  0.26269  3.32803
H  -0.77064 -0.18760  2.64392
H  -1.63468 -1.32903  3.73924
H  -1.60267 -2.02443 -1.96289
H  -3.19030 -1.22722 -2.22360
H  -3.10118 -3.00980 -1.97063
C   2.16247  2.00888  1.60453
H   0.08171  0.38637 -1.75225
H  -0.57974 -0.00788 -1.89434
C   3.31881 -0.28792  0.24871
C  -2.85714  1.56305 -0.54707
C  -0.47764  3.05644 -0.76108
C   2.45903 -4.60193 -2.47891
C   1.79410 -5.48017  0.64037
H  -4.68911 -1.02369  3.49861
H  -5.34368 -2.54696  2.85322
H  -3.86347 -2.56183  3.83928
H  -5.53898 -2.40174 -0.83154
H  -4.74383 -3.96570 -0.50897
H  -5.76849 -3.21498  0.73438
H   0.88072 -5.93140  1.06833
H   2.23555 -6.21799 -0.04741
H   2.51173 -5.33646  1.46915
H   3.41609 -4.13444 -2.77491
H   2.68804 -5.62384 -2.13872
H   1.83375 -4.68949 -3.38589
C  -3.00149  1.65613 -1.95344
C  -4.25714  1.52411 -2.56859
C  -5.40736  1.29015 -1.79348
C  -5.28708  1.20547 -0.39624
C  -4.02995  1.34927  0.21433
H  -2.11816  1.85729 -2.56908
H  -4.33946  1.61207 -3.65855
H  -6.38831  1.19636 -2.27231
H  -6.17845  1.05029  0.22284
H  -3.95271  1.31369  1.30747
C  -1.21114  4.25689 -0.93734
C  -0.69911  5.31742 -1.69717
C   0.57076  5.21531 -2.29278
C   1.31663  4.04080 -2.11722
C   0.79439  2.97533 -1.36376
H  -2.19842  4.35696 -0.47221
H  -1.29181  6.23125 -1.81991
```

```
H   0.97417  6.04580 -2.88237
H   2.31237  3.95015 -2.56670
H   1.38309  2.06415 -1.21763
C   3.36045  2.68344  1.26591
C   3.63901  3.96442  1.76745
C   2.72735  4.61577  2.61332
C   1.53487  3.96089  2.96387
C   1.26300  2.67483  2.47762
H   4.09351  2.19941  0.61390
H   4.57872  4.45682  1.49071
H   2.94490  5.61771  2.99866
H   0.81217  4.45059  3.62649
H   0.33499  2.17609  2.77623
C   3.76458  0.25196 -0.98190
C   4.92709 -0.22196 -1.61265
C   5.67208 -1.26295 -1.03218
C   5.24961 -1.81256  0.18852
C   4.09342 -1.32328  0.82086
H   3.19851  1.06606 -1.44831
H   5.25243  0.22414 -2.55985
H   6.57893 -1.63360 -1.52258
H   5.82919 -2.61554  0.65859
H   3.78231 -1.73999  1.78589
```

H2
BP86
SCF =   -1.17646508743
H(0 K)=   -1.166533
H(298 K)=   -1.163228
G(298 K)=   -1.178039
SCF(C6H6) =   -1.17652510343
SCF(D3BJ) =   -1.17657507617
SCF(BS2) =   -1.17758474363
Low Freq. = 4359.8244cm-1, cm-1
B97D
SCF(C6H6,BS2)= -1.18311990532

```
2
H2
H   0.00000  0.00000  0.37513
H   0.00000  0.00000 -0.37513
```

cis-V
BP86
SCF =   -1877.78811574
H(0 K)=   -1877.074223
H(298 K)=   -1877.023740
G(298 K)=   -1877.161844
SCF(C6H6) =   -1877.79671373
SCF(D3BJ) =   -1878.04543066
SCF(BS2) =   -3885.57179759
Low Freq. = 10.7928cm-1, 13.7108cm-1
B97D
SCF(C6H6,BS2)= -3884.75057513

89
cis-V

```
C   1.37884  3.11126 -1.48521
C   1.61595  2.93716 -0.10320
C   1.93958  4.07958  0.66433
C   2.03736  5.34862  0.07258
C   1.80390  5.50316 -1.30396
C   1.47261  4.38152 -2.08013
P   1.54375  1.28020  0.77942
C   3.34818  0.79792  0.62150
C   3.88280 -0.09121  1.58711
C   5.22678 -0.49124  1.55242
C   6.08705  0.00139  0.55545
C   5.58271  0.90367 -0.39660
C   4.23475  1.29749 -0.36362
Ni  0.08162 -0.30308  0.08808
C   1.47362 -1.59928 -0.13120
N   2.25524 -1.73711 -1.25777
C   3.17290 -2.78985 -1.14068
C   2.95842 -3.35223  0.09543
N   1.91665 -2.61683  0.68423
C   2.17329 -0.84310 -2.40784
C   1.41511 -2.85866  2.03002
C  -1.31484 -1.52740  0.51474
N  -2.05074 -1.49785  1.68027
C  -2.99777 -2.53137  1.73227
C  -2.85093 -3.24657  0.56701
N  -1.81372 -2.62540 -0.14695
C  -1.87668 -0.48256  2.71519
C  -1.35471 -3.06578 -1.45857
P  -1.39807  1.17295 -0.83427
C  -2.95636  0.26659 -1.37015
C  -4.13087  0.10206 -0.59599
C  -5.24594 -0.58535 -1.09982
C  -5.22154 -1.13146 -2.39568
C  -4.07044 -0.97006 -3.18577
C  -2.95934 -0.27553 -2.67856
C  -2.15603  2.47350  0.27954
C  -1.55261  2.88208  1.49150
C  -2.07837  3.94414  2.24556
C  -3.22562  4.62643  1.81208
C  -3.83535  4.23950  0.60557
C  -3.30494  3.18596 -0.15176
C   4.16755 -3.11586 -2.20745
C   3.63933 -4.48894  0.78692
C  -3.60401 -4.42358  0.03671
C  -3.93597 -2.71154  2.88211
H  -0.48206 -2.45360 -1.72368
H  -2.14296 -2.91718 -2.21439
H  -1.06230 -4.12937 -1.42863
H  -1.76910 -0.95692  3.70534
H  -2.73013  0.21497  2.73537
H  -0.96457  0.08625  2.47511
H   2.00373 -1.41548 -3.33569
H   3.09965 -0.25239 -2.50040
H   1.32652 -0.16112 -2.23134
H   0.62978 -2.11754  2.22827
H   2.22217 -2.74019  2.77313
H   0.99289 -3.87450  2.11802
H  -4.17562  0.53760  0.40783
H  -6.14307 -0.69261 -0.47855
H  -6.09713 -1.65727 -2.79198
H  -4.04592 -1.36534 -4.20824
H  -2.07593 -0.12662 -3.31203
H  -0.65300  2.36506  1.84645
H  -1.58473  4.23672  3.17972
H  -3.63690  5.45332  2.40122
H  -4.72590  4.76786  0.24570
```

```
H  -3.78701  2.91024 -1.09590
H   3.23132 -0.44672  2.39484
H   5.61231 -1.17148  2.32138
H   7.14067 -0.29750  0.53403
H   6.24591  1.31316 -1.16800
H   3.86471  2.01518 -1.10376
H   2.12020  3.96453  1.73947
H   2.29179  6.21881  0.68822
H   1.87440  6.49332 -1.76757
H   1.28195  4.49317 -3.15368
H   1.09669  2.24794 -2.09450
H  -4.59394 -3.57643  2.70507
H  -3.40234 -2.89051  3.83389
H  -4.58183 -1.82726  3.03076
H  -4.35913 -4.75368  0.76699
H  -4.13380 -4.17420 -0.90068
H  -2.94565 -5.28675 -0.17218
H   4.14708 -4.16367  1.71345
H   2.93556 -5.29583  1.06246
H   4.40564 -4.92900  0.13017
H   4.76220 -3.99668 -1.91956
H   3.68438 -3.34206 -3.17551
H   4.87038 -2.27964 -2.37394
```

TS(cis-trans-V)

```
BP86
SCF  =       -1877.76858425
H(0 K)=      -1877.054999
H(298 K)=    -1877.005402
G(298 K)=    -1877.140562
SCF(C6H6) =        -1877.77584063
SCF(D3BJ) =        -1878.02022457
SCF(BS2) =         -3885.55393997
Low Freq. = -22.2113cm-1,
10.9976cm-1
B97D
SCF(C6H6,BS2)=  -3884.72721421
```

89

TS(cis-trans-V)

```
C   2.19990  3.78650 -0.90559
C   2.03913  2.90401  0.18900
C   1.99772  3.46085  1.48871
C   2.11120  4.84743  1.68869
C   2.26277  5.70831  0.58952
C   2.30795  5.17285 -0.70893
P   1.83270  1.05967 -0.10632
C   3.62526  0.57894 -0.18058
C   3.99457 -0.62140 -0.84170
C   5.32958 -1.04493 -0.89165
C   6.34332 -0.27871 -0.29007
C   5.99891  0.91971  0.35829
C   4.66373  1.34410  0.41190
Ni  0.10842 -0.26576 -0.06619
C   1.05178 -2.05745 -0.20275
N   1.28195 -2.92116 -1.26063
C   2.09840 -4.00757 -0.90629
C   2.39503 -3.84425  0.42647
N   1.74640 -2.66870  0.82725
C   0.83024 -2.68315 -2.62754
C   1.86357 -2.10200  2.16599
C  -1.19023 -0.10572  1.30543
N  -1.66778  1.01530  1.96057
C  -2.59162  0.69409  2.96414
C  -2.71978 -0.67439  2.94771
N  -1.86532 -1.13827  1.93932
C  -1.28533  2.38493  1.64494
C  -1.78006 -2.54563  1.56700
P  -1.48852 -0.07106 -1.64710
C  -3.00125 -1.11091 -1.27799
C  -4.04123 -0.74086 -0.38980
C  -5.11720 -1.60473 -0.13144
C  -5.17990 -2.86723 -0.74946
C  -4.15970 -3.25134 -1.63572
C  -3.08926 -2.38079 -1.89680
C  -2.23535  1.62457 -1.84735
C  -1.36092  2.74032 -1.81200
C  -1.81013  4.03037 -2.12507
C  -3.15111  4.24614 -2.48788
C  -4.02901  3.15065 -2.54654
C  -3.57919  1.85804 -2.23653
C   2.49281 -5.07887 -1.87233
C   3.22166 -4.67524  1.35487
C  -3.56922 -1.58917  3.77000
C  -3.23997  1.72846  3.82766
H  -1.03110 -2.62730  0.76725
H  -2.75489 -2.90339  1.19600
H  -1.47021 -3.15530  2.43353
H  -0.83899  2.87702  2.52495
H  -2.15844  2.96445  1.30306
H  -0.54316  2.34443  0.83949
H   0.24024 -3.53928 -2.99749
H   1.69281 -2.53076 -3.30013
H   0.20524 -1.77640 -2.63090
H   1.22660 -1.20609  2.20035
H   2.90566 -1.80276  2.36991
H   1.53908 -2.83087  2.92888
H  -4.00399  0.23589  0.10362
H  -5.91295 -1.29042  0.55413
H  -6.02144 -3.53937 -0.54961
H  -4.20341 -4.22642 -2.13407
H  -2.30720 -2.68237 -2.60310
H  -0.31299  2.58206 -1.53239
H  -1.10764  4.87012 -2.07820
H  -3.50647  5.25439 -2.72666
H  -5.07449  3.30078 -2.84026
H  -4.27769  1.01874 -2.30975
H   3.21823 -1.21406 -1.33373
H   5.58241 -1.97298 -1.41760
H   7.38777 -0.60547 -0.33505
H   6.77772  1.53296  0.82646
H   4.42042  2.28201  0.92065
H   1.88313  2.79422  2.35147
H   2.08403  5.25559  2.70566
H   2.34736  6.78946  0.74347
H   2.43294  5.83642 -1.57210
H   2.24211  3.37687 -1.92128
H  -3.93367  1.25227  4.53802
H  -2.50066  2.30036  4.41803
H  -3.81924  2.46011  3.23554
H  -4.16563 -1.01012  4.49221
H  -4.27132 -2.16719  3.14194
H  -2.96708 -2.31685  4.34468
H   4.07335 -4.10560  1.76882
```

```
H   2.63406 -5.05570  2.21082
H   3.63365 -5.54763  0.82384
H   3.14324 -5.81777 -1.37856
H   1.61699 -5.62269 -2.27217
H   3.04799 -4.67513 -2.73856
```

trans-V

```
BP86
SCF  =      -1877.80117589
H(0 K)=     -1877.086949
H(298 K)=   -1877.036499
G(298 K)=   -1877.175608
SCF(C6H6) =       -1877.80854592
SCF(D3BJ) =       -1878.06041595
SCF(BS2) =        -3885.58336824
Low Freq. = 11.0985cm-1, 12.0431cm-
1
B97D
SCF(C6H6,BS2)=  -3884.76419163
```

89

trans-V

```
Ni -0.00005 -0.00003  0.46401
P   0.64522  2.18137  0.84581
N   2.65826 -0.60982 -0.66531
N   2.56576 -1.11313  1.43251
N  -2.56576  1.11303  1.43282
N  -2.65833  0.61003 -0.66508
C   1.80375 -0.59271  0.41056
C   3.91702 -1.12603 -0.33218
C   3.86061 -1.44519  1.00335
C   2.30375 -0.10727 -1.98469
H   1.28116  0.29389 -1.90881
H   2.33060 -0.91724 -2.73323
H   2.98559  0.70567 -2.28226
C   2.11832 -1.22460  2.81726
H   1.03539 -1.04098  2.83846
H   2.63656 -0.48696  3.45581
H   2.31202 -2.23818  3.20360
C   5.02898 -1.23868 -1.32370
H   4.74947 -1.86587 -2.18957
H   5.91304 -1.69750 -0.85443
H   5.33274 -0.24853 -1.70926
C   4.88961 -2.03194  1.91464
H   5.11649 -1.36882  2.76944
H   5.83028 -2.19774  1.36703
H   4.57148 -3.00629  2.32890
C  -1.80382  0.59268  0.41078
C  -3.86059  1.44525  1.00372
C  -3.91705  1.12628 -0.33185
C  -5.02899  1.23924 -1.32335
H  -4.74934  1.86643 -2.18918
H  -5.91294  1.69823 -0.85405
H  -5.33297  0.24918 -1.70898
C  -4.88954  2.03195  1.91511
H  -5.11633  1.36880  2.76990
H  -5.83026  2.19772  1.36757
H  -4.57142  3.00630  2.32936
C  -2.11826  1.22416  2.81758
H  -2.63533  0.48536  3.45575
H  -2.31339  2.23720  3.20459
H  -1.03505  1.04211  2.83849
C  -2.30388  0.10757 -1.98451
H  -1.28149 -0.29408 -1.90859
H  -2.33027  0.91770 -2.73290
H  -2.98608 -0.70499 -2.28231
P  -0.64521 -2.18144  0.84588
C  -0.27849  3.36664 -0.28971
C  -0.74344  4.58202  0.26242
C  -0.54516  3.12510 -1.65745
C  -1.44669  5.51746 -0.51572
C  -1.24317  4.05820 -2.44283
C  -1.69938  5.25836 -1.87218
H  -0.54233  4.79307  1.31914
H  -0.19341  2.19174 -2.11003
H  -1.79409  6.45255 -0.06171
H  -1.42753  3.85063 -3.50364
H  -2.24490  5.98689 -2.48188
C  -2.40673 -2.55732  0.33377
C  -2.79500 -3.24832 -0.83959
C  -3.43422 -2.17661  1.23368
H  -2.02988 -3.58449 -1.54685
H  -3.15973 -1.68097  2.17264
C  -4.14624 -3.52357 -1.10900
C  -4.78388 -2.43840  0.96093
H  -4.41360 -4.06750 -2.02286
H  -5.55266 -2.13193  1.67978
C  -5.15108 -3.11361 -0.21699
H  -6.20358 -3.33340 -0.42611
C   2.40676  2.55736  0.33397
C   3.43417  2.17622  1.23381
C   2.79517  3.24888 -0.83904
H   3.15959  1.68013  2.17251
H   2.03014  3.58535 -1.54625
C   4.78385  2.43817  0.96134
C   4.14644  3.52427 -1.10817
H   5.55256  2.13138  1.68014
H   4.41390  4.06860 -2.02176
C   5.15118  3.11393 -0.21623
H   6.20370  3.33383 -0.42512
C   0.27850 -3.36681 -0.28954
C   0.74277 -4.58250  0.26252
C   0.54583 -3.12508 -1.65711
H   0.54116 -4.79369  1.31912
H   0.19461 -2.19148 -2.10962
C   1.44596 -5.51804 -0.51554
C   1.24381 -4.05827 -2.44240
H   1.79282 -6.45336 -0.06159
H   1.42871 -3.85054 -3.50309
C   1.69932 -5.25873 -1.87183
H   2.24482 -5.98734 -2.48146
```

^{3}V

```
BP86
SCF  =      -1877.77239518
H(0 K)=     -1877.059321
H(298 K)=   -1877.008379
G(298 K)=   -1877.149674
SCF(C6H6) =       -1877.78040846
SCF(D3BJ) =       -1878.01969130
SCF(BS2) =        -3885.55521167
Low Freq. = 12.3042cm-1, 15.3514cm-
1
B97D
```

SCF(C6H6,BS2)= -3884.72788476

89
^{3}V

```
C   5.07498  1.57096  2.68461
C   3.96805  1.30121  3.51024
C   2.66895  1.31058  2.98155
C   2.43553  1.59729  1.61186
C   3.56185  1.87885  0.79900
C   4.86365  1.86116  1.32519
P   0.76407  1.50839  0.80598
C  -0.35750  2.32433  2.03060
C   0.08052  3.30192  2.96157
C  -0.83065  3.95573  3.80263
C  -2.20453  3.66553  3.73164
C  -2.65641  2.71254  2.80281
C  -1.74806  2.04901  1.96619
Ni  0.25172 -0.30525 -0.42054
C   2.03031 -0.53184 -1.32350
N   2.40370 -0.08282 -2.57351
C   3.76883 -0.28100 -2.82680
C   4.28078 -0.89040 -1.70312
N   3.20648 -1.03876 -0.81589
C   1.48750  0.56786 -3.50695
C   3.33413 -1.61201  0.52094
C   4.42868  0.12632 -4.10563
C   5.67006 -1.33316 -1.37282
C  -0.15472 -2.06087  0.40354
N  -0.07525 -3.30136 -0.20203
C  -0.50799 -4.33689  0.64042
C  -0.86606 -3.73838  1.82594
N  -0.63632 -2.36558  1.65781
C   0.32690 -3.50749 -1.58906
C  -0.94826 -1.36491  2.67350
C  -0.52438 -5.77277  0.22287
C  -1.40207 -4.32178  3.09396
H   0.69503 -2.54575 -1.97265
H  -0.53041 -3.83613 -2.20225
H   1.12526 -4.26624 -1.65309
H  -0.51459 -0.40990  2.34399
H  -0.51401 -1.65645  3.64404
H  -2.03927 -1.24828  2.78200
H  -1.15423 -5.93953 -0.66997
H  -0.92739 -6.40039  1.03290
H   0.48694 -6.15111 -0.01610
H  -0.73082 -4.14053  3.95351
H  -1.52193 -5.41177  2.99279
H  -2.38950 -3.89999  3.35399
H   6.05192 -0.82779 -0.46733
H   6.35540 -1.09753 -2.20189
H   5.73038 -2.42215 -1.19163
H   4.01251 -0.40828 -4.97946
H   5.50653 -0.09596 -4.06653
H   4.31888  1.20814 -4.30234
H   1.59819  0.13522 -4.51489
H   1.67715  1.65335 -3.55643
H   0.46094  0.40016 -3.14746
H   2.32991 -1.62481  0.96656
H   4.00012 -0.99258  1.14402
H   3.73178 -2.63992  0.46349
H   1.14355  3.55662  3.01990
H  -0.46460  4.70512  4.51406
H  -2.91379  4.17963  4.38909
H  -3.72429  2.47717  2.73091
H  -2.10824  1.30506  1.24585
H   1.81795  1.09585  3.63696
H   3.40584  2.11991 -0.25860
H   4.11903  1.07809  4.57282
H   5.71499  2.09518  0.67531
H   6.08864  1.56641  3.09949
P  -1.74713  0.04616 -1.40921
C  -2.25496  1.68409 -2.11760
C  -3.32505 -0.64333 -0.73155
C  -4.39614  0.16085 -0.26091
C  -5.54508 -0.41743  0.29828
C  -5.65955 -1.81374  0.41448
C  -4.60689 -2.62595 -0.04232
C  -3.46180 -2.05031 -0.61069
H  -4.32383  1.25051 -0.33769
H  -6.35636  0.22999  0.65096
H  -6.55887 -2.26263  0.84942
H  -4.68482 -3.71707  0.03019
H  -2.65837 -2.69686 -0.97974
C  -3.44563  1.83911 -2.87357
C  -3.74773  3.05426 -3.50350
C  -2.86502  4.14512 -3.40712
C  -1.67849  4.00473 -2.66899
C  -1.37562  2.79094 -2.03224
H  -4.13737  0.99522 -2.96900
H  -4.67528  3.14818 -4.08009
H  -3.10206  5.09353 -3.90129
H  -0.98690  4.85009 -2.57538
H  -0.45521  2.69613 -1.44215
```

(iii) PPh2H addition to trans-III

TS(trans-III-trans-V)1
BP86
SCF =   -1878.92556762
H(0 K)=   -1878.197047
H(298 K)=   -1878.146983
G(298 K)=   -1878.283150
SCF(C6H6) =   -1878.93267147
SCF(D3BJ) =   -1879.19124752
SCF(BS2) =   -3886.70705402
Low Freq. = -334.2500cm-1,
13.5216cm-1
B97D
SCF(C6H6,BS2)= -3885.88952400

91
TS(trans-III-trans-V)1

```
Ni  0.00811 -0.02965  0.18756
P  -0.84260  1.92102 -0.98282
N  -2.66851 -0.72628  1.27549
N  -2.58039 -1.13820 -0.84590
N   2.58499  1.05681 -0.80943
N   2.72697  0.57703  1.29576
C  -1.80676 -0.68318  0.20025
C  -3.93256 -1.20912  0.91336
C  -3.87369 -1.48009 -0.43153
C  -2.37063 -0.24459  2.62046
H  -2.77039  0.77494  2.75876
H  -1.27568 -0.22083  2.72542
```

```
H  -2.81554 -0.92232  3.36635
C  -2.15707 -1.24358 -2.23943
H  -1.20314 -0.71332 -2.35466
H  -2.91379 -0.77850 -2.89212
H  -2.02103 -2.29987 -2.52514
C  -5.06417 -1.33464  1.88188
H  -4.83310 -2.02684  2.71207
H  -5.95780 -1.72286  1.36924
H  -5.33266 -0.35904  2.32554
C  -4.90352 -2.03099 -1.36421
H  -5.16374 -1.31818 -2.16814
H  -5.82945 -2.25917 -0.81416
H  -4.56363 -2.96480 -1.84671
C   1.84870  0.53080  0.23264
C   3.87276  1.43147 -0.40681
C   3.96600  1.11816  0.92601
C   5.11173  1.24028  1.87782
H   4.86266  1.85701  2.76034
H   5.97116  1.71166  1.37654
H   5.44189  0.25164  2.24508
C   4.87038  2.03777 -1.33998
H   5.15635  1.34476 -2.15253
H   5.78922  2.29991 -0.79310
H   4.48710  2.96080 -1.81021
C   2.13002  1.20707 -2.18981
H   2.90965  0.83973 -2.87613
H   1.90956  2.26426 -2.41492
H   1.22200  0.60573 -2.32452
C   2.47854  0.09241  2.64909
H   1.41849 -0.20045  2.69857
H   2.69429  0.88651  3.38393
H   3.10786 -0.78890  2.85700
P   0.84125 -1.90493 -1.13927
C   0.12662  3.47228 -0.51150
C   0.84847  3.65745  0.68844
C   0.13482  4.52271 -1.45906
C   1.54696  4.85156  0.93597
C   0.82635  5.71977 -1.21202
C   1.53725  5.88732 -0.01216
H   0.86305  2.85291  1.43157
H  -0.41449  4.39393 -2.39946
H   2.09796  4.97408  1.87569
H   0.81192  6.52121 -1.95924
H   2.08125  6.81822  0.18172
C   2.50520 -2.37361 -0.39605
C   2.68518 -3.05497  0.83128
C   3.66623 -2.05617 -1.14182
H   1.80961 -3.34016  1.42277
H   3.55172 -1.57047 -2.11834
C   3.96719 -3.39391  1.29439
C   4.94996 -2.38123 -0.67598
H   4.07418 -3.93573  2.24188
H   5.82841 -2.12906 -1.28151
C   5.10848 -3.05323  0.54787
H   6.10734 -3.32623  0.90563
H  -0.44549  1.26201  0.81074
H   0.14856 -0.61297  1.58083
C  -2.52188  2.40318 -0.28256
C  -2.70702  3.10179  0.93344
C  -3.66845  2.08985 -1.04676
H  -1.83331  3.37759  1.53415
H  -3.54220  1.58239 -2.01035
C  -3.99076  3.46913  1.36805
C  -4.95517  2.44235 -0.60748
H  -4.10801  4.02258  2.30714
H  -5.82791  2.19142 -1.22112
C  -5.12189  3.13581  0.60261
H  -6.12264  3.42682  0.93990
C  -0.14141 -3.41588 -0.56719
C  -0.67523 -3.59895  0.72875
C  -0.34755 -4.44268 -1.51731
H  -0.53675 -2.80597  1.47201
H   0.06609 -4.31996 -2.52575
C  -1.38123 -4.76671  1.06347
C  -1.05778 -5.61032 -1.18809
H  -1.78045 -4.88988  2.07736
H  -1.20088 -6.39247 -1.94257
C  -1.57698 -5.77614  0.10586
H  -2.12907 -6.68578  0.36724
```

Int(trans-III-trans-V)

```
BP86
SCF  =       -1878.92946626
H(0 K)=      -1878.199259
H(298 K)=    -1878.148919
G(298 K)=    -1878.285326
SCF(C6H6) =        -1878.93667202
SCF(D3BJ) =        -1879.19705667
SCF(BS2) =         -3886.71007117
Low Freq. = 12.7684cm-1, 18.7019cm-
1
B97D
SCF(C6H6,BS2)=  -3885.89511596
```

91

Int(trans-III-trans-V)

```
Ni -0.00003  0.00001  0.23739
P  -0.52732  1.92957 -1.11371
N  -2.70968 -0.30084  1.36384
N  -2.75337 -0.64341 -0.77199
N   2.75335  0.64339 -0.77188
N   2.70964  0.30082  1.36393
C  -1.89160 -0.33563  0.25711
C  -4.04458 -0.57647  1.03507
C  -4.06915 -0.80260 -0.31910
C  -2.27121  0.03195  2.71594
H  -2.19899  1.12573  2.83806
H  -1.27849 -0.40870  2.88290
H  -2.99428 -0.37375  3.43902
C  -2.39018 -0.81153 -2.17699
H  -1.36436 -0.44866 -2.31994
H  -3.07038 -0.21908 -2.80978
H  -2.44743 -1.87482 -2.46387
C  -5.15700 -0.57084  2.03402
H  -5.01671 -1.32573  2.82946
H  -6.11032 -0.79888  1.53275
H  -5.26707  0.41451  2.52145
C  -5.19958 -1.15307 -1.23182
H  -5.36900 -0.37683 -2.00028
H  -6.13290 -1.25735 -0.65747
H  -5.02350 -2.10840 -1.75775
C   1.89157  0.33558  0.25721
C   4.06912  0.80257 -0.31900
C   4.04454  0.57645  1.03517
```

```
C   5.15693  0.57080  2.03416
H   5.01669  1.32576  2.82953
H   6.11029  0.79871  1.53289
H   5.26689 -0.41452  2.52168
C   5.19954  1.15313 -1.23170
H   5.36857  0.37723 -2.00058
H   6.13298  1.25679 -0.65743
H   5.02371  2.10879 -1.75713
C   2.39019  0.81147 -2.17690
H   3.06999  0.21852 -2.80964
H   2.44802  1.87468 -2.46395
H   1.36415  0.44918 -2.31973
C   2.27123 -0.03199  2.71605
H   1.27806  0.40777  2.88262
H   2.99375  0.37465  3.43917
H   2.20005 -1.12581  2.83850
P   0.52728 -1.92954 -1.11377
C   0.69767  3.30688 -0.68704
C   1.36120  3.47797  0.54849
C   0.95430  4.25181 -1.70826
C   2.23876  4.55483  0.75916
C   1.83216  5.32988 -1.50291
C   2.47833  5.48485 -0.26600
H   1.18594  2.74988  1.34742
H   0.44807  4.13625 -2.67427
H   2.73743  4.66860  1.72897
H   2.00927  6.04971 -2.31020
H   3.16377  6.32364 -0.10178
C   2.05612 -2.69657 -0.31722
C   2.06871 -3.34523  0.93967
C   3.26671 -2.65781 -1.04644
H   1.14161 -3.41287  1.51818
H   3.27125 -2.19912 -2.04241
C   3.24327 -3.92285  1.44798
C   4.44779 -3.22250 -0.53608
H   3.22135 -4.43185  2.41901
H   5.37011 -3.18251 -1.12718
C   4.44156 -3.85881  0.71554
H   5.35640 -4.31464  1.10987
H  -0.14961  0.96499  1.36213
H   0.14942 -0.96482  1.36230
C  -2.05613  2.69663 -0.31714
C  -2.06865  3.34544  0.93967
C  -3.26678  2.65771 -1.04626
H  -1.14150  3.41320  1.51809
H  -3.27138  2.19889 -2.04217
C  -3.24320  3.92307  1.44801
C  -4.44785  3.22241 -0.53587
H  -3.22123  4.43219  2.41897
H  -5.37022  3.18229 -1.12689
C  -4.44154  3.85888  0.71567
H  -5.35637  4.31472  1.11002
C  -0.69765 -3.30688 -0.68698
C  -1.36114 -3.47795  0.54857
C  -0.95426 -4.25188 -1.70816
H  -1.18588 -2.74982  1.34747
H  -0.44806 -4.13634 -2.67418
C  -2.23863 -4.55485  0.75931
C  -1.83206 -5.32998 -1.50274
H  -2.73727 -4.66860  1.72915
H  -2.00916 -6.04986 -2.30999
C  -2.47819 -5.48492 -0.26580
H  -3.16358 -6.32374 -0.10152
```

TS(trans-III-trans-V)2

```
BP86
SCF  =       -1878.92875725
H(0 K)=     -1878.200001
H(298 K)=   -1878.149876
G(298 K)=   -1878.285394
SCF(C6H6) =       -1878.93578129
SCF(D3BJ) =       -1879.19697743
SCF(BS2) =        -3886.70952262
Low Freq. = -308.5364cm-1,
14.5290cm-1
B97D
SCF(C6H6,BS2)=  -3885.89478053
```

91

TS(trans-III-trans-V)2

```
Ni -0.00000  0.00001  0.19362
P   0.36663 -2.04049 -1.01856
N   2.79753  0.07820  1.16569
N   2.73467  0.41646 -0.97045
N  -2.73466 -0.41652 -0.97047
N  -2.79754 -0.07813  1.16565
C   1.91595  0.17674  0.11163
C   4.12593  0.25984  0.75406
C   4.08364  0.48282 -0.60030
C   2.41945 -0.22832  2.54298
H   2.10441 -1.28106  2.62570
H   1.58029  0.41439  2.84702
H   3.28438 -0.05028  3.19747
C   2.29366  0.60758 -2.34935
H   1.23503  0.32902 -2.41511
H   2.87921 -0.03939 -3.02251
H   2.41444  1.66207 -2.64940
C   5.29817  0.17497  1.67845
H   5.25470  0.92568  2.48855
H   6.22830  0.35508  1.11753
H   5.38231 -0.82276  2.14565
C   5.17682  0.75712 -1.58213
H   5.24178 -0.02229 -2.36346
H   6.14982  0.79009 -1.06831
H   5.03733  1.72681 -2.09280
C  -1.91595 -0.17674  0.11162
C  -4.08363 -0.48286 -0.60034
C  -4.12594 -0.25980  0.75401
C  -5.29819 -0.17486  1.67837
H  -5.25474 -0.92553  2.48852
H  -6.22832 -0.35501  1.11746
H  -5.38235  0.82289  2.14551
C  -5.17679 -0.75722 -1.58216
H  -5.24175  0.02214 -2.36355
H  -6.14980 -0.79017 -1.06835
H  -5.03730 -1.72695 -2.09278
C  -2.29362 -0.60773 -2.34934
H  -2.87917  0.03918 -3.02256
H  -2.41438 -1.66224 -2.64933
H  -1.23499 -0.32915 -2.41511
C  -2.41949  0.22848  2.54293
H  -2.10447  1.28123  2.62559
H  -1.58032 -0.41419  2.84701
H  -3.28443  0.05046  3.19742
```

```
P  -0.36661  2.04042 -1.01869
C  -0.99877 -3.25951 -0.54431
C  -1.35645 -4.21326 -1.52559
C  -1.67836 -3.30255  0.69346
C  -2.34981 -5.17685 -1.28014
C  -2.67007 -4.26536  0.94503
C  -3.01063 -5.20564 -0.04173
H  -0.83741 -4.19780 -2.49170
H  -1.42247 -2.56468  1.46140
H  -2.60501 -5.90678 -2.05690
H  -3.17879 -4.28249  1.91615
H  -3.78563 -5.95496  0.15376
C  -1.80720  2.90531 -0.16347
C  -1.72658  3.57310  1.08113
C  -3.04916  2.92992 -0.83981
H  -0.77300  3.59599  1.61870
H  -3.12543  2.45603 -1.82550
C  -2.84171  4.22800  1.62870
C  -4.17134  3.56978 -0.28838
H  -2.74674  4.74866  2.58901
H  -5.11947  3.57519 -0.83853
C  -4.07308  4.22365  0.95086
H  -4.94121  4.73915  1.37603
H   0.05178 -0.78632  1.46260
H  -0.05181  0.78642  1.46255
C   1.80720 -2.90532 -0.16325
C   3.04918 -2.92997 -0.83956
C   1.72656 -3.57303  1.08140
H   3.12547 -2.45615 -1.82528
H   0.77297 -3.59588  1.61895
C   4.17135 -3.56980 -0.28807
C   2.84168 -4.22789  1.62903
H   5.11949 -3.57524 -0.83819
H   2.74668 -4.74849  2.58937
C   4.07306 -4.22358  0.95122
H   4.94118 -4.73905  1.37644
C   0.99878  3.25948 -0.54450
C   1.35648  4.21317 -1.52583
C   1.67834  3.30260  0.69329
H   0.83746  4.19764 -2.49194
H   1.42243  2.56477  1.46127
C   2.34983  5.17677 -1.28042
C   2.67005  4.26542  0.94482
H   2.60505  5.90665 -2.05721
H   3.17875  4.28260  1.91595
C   3.01063  5.20564 -0.04199
H   3.78563  5.95497  0.15347
```

(iv) P-C Bond Activation

TS(I-cis-III)Ph

```
BP86
SCF  =      -1408.48686759
H(0 K)=     -1407.944718
H(298 K)=   -1407.906894
G(298 K)=   -1408.015760
SCF(C6H6) =       -1408.49293805
SCF(D3BJ) =       -1408.64915772
SCF(BS2) =        -3081.28612800
Low Freq. = -131.6266cm-1,
20.6539cm-1
B97D
SCF(C6H6,BS2)=  -3080.81857835
```

```
67
```

TS(I-cis-III)Ph

```
C  -0.28334 -3.97100  2.28350
C   0.64829 -2.94271  2.48860
C   0.87238 -1.97363  1.48882
C   0.10862 -1.96307  0.28952
C  -0.78515 -3.05031  0.07864
C  -0.98422 -4.02899  1.05976
Ni -0.23480 -0.10211 -0.23443
C   0.48960  1.63930  0.24606
N   0.55947  2.19693  1.51415
C   1.01374  3.52490  1.49978
C   1.25621  3.83238  0.18241
N   0.94263  2.67824 -0.55360
C   0.14645  1.49402  2.72033
C   1.06279  2.58781 -2.00300
C   1.18036  4.34320  2.74031
C   1.76595  5.08793 -0.44989
P   1.16667 -1.12705 -1.53113
C   2.95503 -1.34924 -1.07818
C   3.70758 -2.46363 -1.51821
C   5.07922 -2.56081 -1.24286
C   5.73001 -1.55130 -0.51216
C   4.99326 -0.44305 -0.06074
C   3.62228 -0.34247 -0.34118
C  -2.16418 -0.10108 -0.37363
N  -3.13984 -0.41490  0.55546
C  -4.44052 -0.25111  0.05133
C  -4.29696  0.17201 -1.24874
N  -2.91525  0.25028 -1.48327
C  -2.84725 -0.85346  1.91406
C  -2.32150  0.65435 -2.74996
C  -5.66765 -0.52772  0.85979
C  -5.31943  0.49243 -2.29181
H   0.98881 -2.49222 -1.97818
H  -1.77243 -1.07109  1.97516
H  -3.40495 -1.77368  2.15240
H  -3.11307 -0.07276  2.65014
H  -1.24951  0.40461 -2.70335
H  -2.44099  1.73934 -2.92350
H  -2.78454  0.10684 -3.58817
H  -5.73176 -1.58652  1.17294
H  -6.57160 -0.30347  0.27176
H  -5.70930  0.08605  1.77846
H  -5.22065  1.52685 -2.66938
H  -6.33433  0.38765 -1.87687
H  -5.25027 -0.18134 -3.16636
H   2.73868  4.93784 -0.95444
H   1.90907  5.86914  0.31321
H   1.06541  5.48734 -1.20608
H   0.23634  4.43865  3.30765
H   1.51588  5.36045  2.48401
H   1.93211  3.91270  3.42821
H  -0.84505  1.83851  3.06669
H   0.87995  1.65087  3.52869
H   0.09470  0.42085  2.48329
H   0.97010  1.52670 -2.28192
H   2.04685  2.96100 -2.33219
H   0.27320  3.17395 -2.50768
H   3.20915 -3.26288 -2.07923
```

```
H   3.04577  0.51918  0.01619
H   5.64179 -3.43439 -1.59180
H   5.48933  0.34647  0.51597
H   6.79964 -1.63217 -0.29064
H   1.66885 -1.23332  1.62585
H  -1.35441 -3.10058 -0.85722
H   1.23327 -2.90829  3.41597
H  -1.69847 -4.84154  0.87765
H  -0.44189 -4.73815  3.04918
```

cis-IIIPh

```
BP86
SCF  =      -1408.50655536
H(0 K)=     -1407.963010
H(298 K)=   -1407.924751
G(298 K)=   -1408.034488
SCF(C6H6) =       -1408.51558169
SCF(D3BJ) =       -1408.67435942
SCF(BS2) =        -3081.30293055
Low Freq. = 12.6225cm-1, 21.9520cm-
1
B97D
SCF(C6H6,BS2)=  -3080.84341702
```

67

cis-IIIPh

```
C  -3.30765 -4.19880  0.50407
C  -2.85591 -3.39621  1.56565
C  -2.00160 -2.30747  1.31764
C  -1.54647 -1.98218  0.01244
C  -2.01687 -2.81440 -1.03531
C  -2.88186 -3.89997 -0.79931
Ni -0.36120 -0.45259 -0.18581
C   0.95533  0.93999 -0.07182
N   1.86567  1.13730  0.94376
C   2.67194  2.26352  0.72580
C   2.25544  2.81101 -0.46489
N   1.21287  1.99001 -0.92663
C   2.02595  0.22220  2.06917
C   0.50306  2.19472 -2.18191
C   3.76951  2.66119  1.65913
C   2.73682  4.01112 -1.21539
P   1.08797 -1.96769 -1.01537
C   2.86701 -1.73996 -0.49995
C   3.51483 -2.61326  0.41040
C   4.87096 -2.45719  0.73812
C   5.62401 -1.41369  0.17192
C   5.00388 -0.54150 -0.74123
C   3.65216 -0.71009 -1.07642
C  -1.91837  0.64424  0.01338
N  -2.38316  1.32862  1.11419
C  -3.62738  1.93752  0.87768
C  -3.95770  1.63736 -0.42262
N  -2.90397  0.85811 -0.92506
C  -1.68200  1.37525  2.38983
C  -2.88358  0.27179 -2.26019
C  -4.35264  2.72963  1.91757
C  -5.15626  1.99998 -1.23931
H   0.89189 -3.16821 -0.24029
H  -0.74562  0.81366  2.26710
H  -2.28377  0.90624  3.18681
H  -1.45225  2.41595  2.67714
H  -1.92247 -0.25099 -2.37608
H  -2.98300  1.05441 -3.03215
H  -3.69557 -0.46464 -2.37686
H  -4.59122  2.12242  2.81006
H  -5.30446  3.10795  1.51343
H  -3.76883  3.60332  2.26192
H  -4.88759  2.58505 -2.13828
H  -5.85363  2.61022 -0.64459
H  -5.70768  1.10605 -1.58321
H   3.13573  3.74757 -2.21245
H   3.54688  4.50783 -0.65901
H   1.93560  4.75708 -1.37069
H   3.40206  2.83535  2.68689
H   4.24714  3.59191  1.31518
H   4.55285  1.88350  1.71395
H   1.79364  0.72595  3.02427
H   3.05399 -0.17229  2.09988
H   1.33247 -0.61808  1.90578
H  -0.16200  1.33197 -2.32400
H   1.21070  2.24636 -3.02657
H  -0.09494  3.12265 -2.15726
H   2.94024 -3.42666  0.86881
H   3.19105 -0.03301 -1.80553
H   5.34128 -3.15248  1.44355
H   5.58233  0.26538 -1.20693
H   6.68216 -1.29036  0.42698
H  -1.67319 -1.70132  2.17303
H  -1.67256 -2.63699 -2.06288
H  -3.17171 -3.61750  2.59324
H  -3.21393 -4.52153 -1.64059
H  -3.97689 -5.04593  0.69200
```

TS(cis-trans-III)Ph

```
BP86
SCF  =      -1408.47049301
H(0 K)=     -1407.928352
H(298 K)=   -1407.890639
G(298 K)=   -1407.998798
SCF(C6H6) =       -1408.47724312
SCF(D3BJ) =       -1408.63384720
SCF(BS2) =        -3081.26853541
Low Freq. = -88.5162cm-1,
20.1473cm-1
B97D
SCF(C6H6,BS2)=  -3080.80229004
```

67

TS(cis-trans-III)Ph

```
Ni -0.34804  0.29769  0.02296
P   0.92752  2.01559 -0.24668
N  -3.06561 -0.72062 -0.57490
N  -2.83184  1.30037 -1.31133
N   1.02073 -2.15348 -1.21759
N   1.94969 -1.66830  0.66462
C  -2.12925  0.29753 -0.66048
C  -4.29833 -0.36362 -1.13444
C  -4.14906  0.91967 -1.60695
C  -2.79310 -2.01795  0.03082
H  -1.70038 -2.12434  0.09913
H  -3.21563 -2.82225 -0.59450
H  -3.21423 -2.08117  1.04783
C  -2.29281  2.61904 -1.62216
```

H -1.19301 2.54679 -1.62409
H -2.60278 3.36312 -0.86747
H -2.64537 2.94465 -2.61385
C -5.47726 -1.28316 -1.16229
H -5.28851 -2.19128 -1.76489
H -6.34841 -0.77346 -1.60265
H -5.76379 -1.61680 -0.14853
C -5.12000 1.81621 -2.30710
H -5.26004 2.77356 -1.77317
H -6.10499 1.32865 -2.37671
H -4.79988 2.05949 -3.33736
C   0.91472 -1.26730 -0.15886
C   2.08244 -3.05850 -1.06493
C   2.67333 -2.74897 0.13860
C   3.84113 -3.36404 0.84164
H   4.65339 -2.63408 1.01110
H   4.25699 -4.19071 0.24440
H   3.56121 -3.77477 1.82910
C   2.40752 -4.11333 -2.07407
H   1.57494 -4.82709 -2.21712
H   3.28609 -4.69354 -1.75090
H   2.64161 -3.68275 -3.06504
C   0.16711 -2.09100 -2.39720
H -0.31079 -3.06705 -2.59067
H   0.74608 -1.79442 -3.28931
H -0.60273 -1.32868 -2.20100
C   2.33278 -0.96065 1.88440
H   1.62560 -0.13186 2.02462
H   3.35191 -0.55166 1.77954
H   2.29311 -1.63210 2.75832
C -1.08826 0.19936 1.79783
C -1.74589 1.31297 2.37592
H -1.87960 2.22649 1.78673
C -2.21560 1.28140 3.70089
H -2.70360 2.16939 4.12161
C -2.06425 0.12458 4.48448
H -2.43457 0.09804 5.51523
C -1.43424 -0.99703 3.92414
H -1.31000 -1.91208 4.51717
C -0.95706 -0.95929 2.59915
H -0.46485 -1.84957 2.18931
C   2.75190 2.22769 -0.41960
C   3.55048 1.14613 -0.87519
H   3.06065 0.20683 -1.15478
C   4.94177 1.26979 -0.98825
H   5.53403 0.42095 -1.35007
C   5.57904 2.47909 -0.65653
H   6.66580 2.57689 -0.75060
C   4.80346 3.56413 -0.21165
H   5.28641 4.51387 0.04587
C   3.41195 3.44136 -0.09635
H   2.82044 4.29602 0.25200
H   0.65133 3.43547 -0.06856

trans-IIIPh
BP86
SCF =   -1408.51430182
H(0 K)=   -1407.971032
H(298 K)=   -1407.932433
G(298 K)=   -1408.043837
SCF(C6H6) =   -1408.52170549
SCF(D3BJ) =   -1408.68138861
SCF(BS2) =   -3081.31044912
Low Freq. = 16.4175cm-1, 21.9100cm-1
B97D
SCF(C6H6,BS2)= -3080.84907562

67
trans-IIIPh
Ni -0.38664 0.05043 -0.20347
P   0.32517 -1.92609 -1.13443
N   1.89275 1.64739 -1.14137
N   2.30583 0.75228 0.77887
N -3.04616 -0.80589 -1.16794
N -2.83675 -1.06638 0.96501
C   1.33969 0.81748 -0.19478
C   3.16856 2.09868 -0.76537
C   3.43296 1.52327 0.45517
C   1.19332 2.05208 -2.35439
H   0.33687 1.37414 -2.48062
H   1.85840 1.96614 -3.22939
H   0.82096 3.08741 -2.27230
C   2.19269 -0.07415 1.97440
H   1.24404 -0.62559 1.89636
H   2.18900 0.54937 2.88506
H   3.02080 -0.80014 2.02106
C   3.98477 3.02022 -1.61352
H   4.22732 2.57840 -2.59783
H   4.93807 3.25193 -1.11353
H   3.46759 3.97873 -1.80211
C   4.63779 1.59478 1.33645
H   4.39468 1.98074 2.34326
H   5.39305 2.26565 0.89821
H   5.10700 0.60241 1.46653
C -2.13908 -0.65215 -0.14458
C -4.28311 -1.28798 -0.70694
C -4.14893 -1.45332 0.65113
C -5.11667 -1.94128 1.68065
H -4.76747 -2.86585 2.17671
H -6.08835 -2.16569 1.21395
H -5.29374 -1.19023 2.47194
C -5.44050 -1.54519 -1.61721
H -5.75328 -0.63430 -2.15970
H -6.30806 -1.89921 -1.03895
H -5.21157 -2.31688 -2.37537
C -2.76152 -0.48456 -2.56201
H -3.33494 0.40043 -2.88902
H -3.00900 -1.33801 -3.21431
H -1.68360 -0.28199 -2.64170
C -2.27520 -1.05154 2.30931
H -1.18953 -0.90798 2.20601
H -2.47181 -2.00852 2.82048
H -2.69038 -0.22091 2.90486
C -1.10344 1.76929 0.38992
C -0.90740 2.25134 1.70942
H -0.32397 1.65410 2.42319
C -1.42791 3.48428 2.14474
H -1.25304 3.81618 3.17625
C -2.16084 4.29495 1.26269
H -2.56389 5.25803 1.59549
C -2.36782 3.85050 -0.05335
H -2.93906 4.46968 -0.75699
C -1.85277 2.60976 -0.47308

```
H  -2.04807  2.29107 -1.50592
C   1.94717 -2.49200 -0.41892
C   3.15704 -1.90528 -0.86990
H   3.11793 -1.11138 -1.62519
C   4.40180 -2.32020 -0.37366
H   5.31812 -1.84662 -0.74589
C   4.48090 -3.35037  0.58130
H   5.45273 -3.68108  0.96314
C   3.29475 -3.96098  1.02461
H   3.33803 -4.77232  1.76090
C   2.05008 -3.53552  0.53535
H   1.13551 -4.01862  0.89882
H  -0.46965 -2.92426 -0.44639
```

^{3}IIIPh

```
BP86
SCF  =       -1408.46711374
H(0 K)=      -1407.924922
H(298 K)=    -1407.886028
G(298 K)=    -1407.999276
SCF(C6H6) =        -1408.47561495
SCF(D3BJ) =        -1408.63389078
SCF(BS2) =         -3081.25704135
Low Freq. = 16.7079cm-1, 19.7317cm-1
B97D
SCF(C6H6,BS2)=  -3080.80002133
```

67

^{3}IIIPh

```
C  -1.65790 -4.41882  2.21693
C  -0.42097 -4.28141  1.56502
C  -0.09546 -3.07985  0.90926
C  -0.97471 -1.96079  0.88289
C  -2.21932 -2.14490  1.54513
C  -2.55928 -3.34134  2.20215
Ni -0.36058 -0.23468  0.15108
C   0.97551  1.22575  0.53769
N   2.02669  1.11011  1.41976
C   2.88726  2.21328  1.37583
C   2.35700  3.07581  0.44075
N   1.19985  2.45316 -0.04898
C   2.26213 -0.08414  2.22910
C   0.35629  3.00343 -1.10514
C   4.11819  2.31793  2.21802
C   2.82238  4.41629 -0.03131
P   0.57748 -0.80648 -1.97547
C   2.34426 -1.24344 -1.63210
C   2.82039 -2.58128 -1.57824
C   4.17602 -2.86809 -1.36183
C   5.10625 -1.82897 -1.18460
C   4.65863 -0.49607 -1.23789
C   3.30477 -0.20974 -1.46134
C  -2.12967  0.51565 -0.29761
N  -2.81859  1.51247  0.36695
C  -4.13561  1.65081 -0.09747
C  -4.28863  0.71289 -1.09305
N  -3.06421  0.03505 -1.18651
C  -2.26738  2.27269  1.48251
C  -2.80323 -1.09039 -2.08342
C  -5.09388  2.65232  0.46356
C  -5.47146  0.39864 -1.95307
H   0.15213 -2.18557 -2.11878
H  -1.20606  1.99901  1.57837
H  -2.78940  2.02493  2.42290
H  -2.35804  3.35734  1.30048
H  -2.27192 -0.75918 -2.99121
H  -3.75622 -1.56066 -2.36687
H  -2.17896 -1.81719 -1.54259
H  -5.28031  2.49132  1.54139
H  -6.06444  2.58197 -0.05162
H  -4.73198  3.69058  0.34587
H  -5.23946  0.49378 -3.02908
H  -6.29730  1.09206 -1.72975
H  -5.84911 -0.62679 -1.78588
H   3.02846  4.42566 -1.11714
H   3.75305  4.70087  0.48374
H   2.07808  5.20949  0.16695
H   3.88694  2.29578  3.29865
H   4.64555  3.26230  2.01159
H   4.82162  1.49056  2.01475
H   2.41318  0.18926  3.28664
H   3.14529 -0.62990  1.85774
H   1.37638 -0.73326  2.13925
H  -0.43636  2.26880 -1.30689
H   0.94120  3.16149 -2.02656
H  -0.08807  3.96473 -0.79331
H   2.11081 -3.40490 -1.71482
H   2.98111  0.83608 -1.51879
H   4.50828 -3.91228 -1.33018
H   5.37384  0.32684 -1.11935
H   6.16490 -2.05346 -1.01626
H   0.87591 -3.01327  0.40122
H  -2.95269 -1.32601  1.55208
H   0.29221 -5.11590  1.56245
H  -3.53166 -3.43442  2.70344
H  -1.91836 -5.35500  2.72417
```

(v) P-C Bond Activation in Ni(IMe$_4$)$_2$(PPh$_3$)

I.PPh3

```
BP86
SCF  =       -1639.55273330
H(0 K)=      -1638.930296
H(298 K)=    -1638.886423
G(298 K)=    -1639.011624
SCF(C6H6) =        -1639.55805045
SCF(D3BJ) =        -1639.74829245
SCF(BS2) =         -3312.43417272
Low Freq. = 8.3538cm-1, 17.0283cm-1
B97D
SCF(C6H6,BS2)=  -3311.79911972
```

77

I.PPh3

```
Ni -0.17644  0.81762 -0.16573
P   0.11057 -1.32343 -0.00193
N  -2.92163  1.55454  0.75794
N  -2.56311  2.16224 -1.28571
N   1.83702  2.72133  0.87299
N   2.48826  1.90782 -1.01946
C  -1.93497  1.51588 -0.22567
C  -4.09323  2.19764  0.32661
```

```
C  -3.86767  2.57714 -0.97605
C  -2.72772  0.98889  2.08380
H  -1.78364  0.42077  2.06046
H  -3.55467  0.30724  2.34833
H  -2.65694  1.77577  2.85717
C  -1.90540  2.35503 -2.56786
H  -0.82873  2.18243 -2.39879
H  -2.06242  3.38293 -2.93795
H  -2.26959  1.64084 -3.32868
C  -5.29105  2.37752  1.20361
H  -5.71394  1.41218  1.53972
H  -6.08617  2.91358  0.66145
H  -5.05836  2.96285  2.11288
C  -4.75070  3.28566 -1.95310
H  -4.32901  4.25706 -2.27311
H  -5.73540  3.48839 -1.50272
H  -4.92142  2.68884 -2.86843
C   1.42972  1.83588 -0.12156
C   3.08404  3.30598  0.60364
C   3.49445  2.79250 -0.60483
C   4.73178  3.04647 -1.40530
H   5.32116  2.12464 -1.56573
H   5.38256  3.76906 -0.88779
H   4.50365  3.46320 -2.40415
C   3.74416  4.28305  1.52343
H   3.15157  5.20851  1.65107
H   4.72853  4.57740  1.12595
H   3.90701  3.86038  2.53240
C   1.01508  3.01035  2.03760
H   1.00449  4.09395  2.24651
H   1.36901  2.47551  2.93728
H  -0.00490  2.66709  1.79446
C   2.52114  1.14912 -2.26103
H   1.62955  0.50461 -2.26966
H   3.41891  0.51031 -2.31575
H   2.50001  1.81868 -3.14026
C  -1.24164 -2.57658 -0.42858
C  -2.54994 -2.08530 -0.60672
H  -2.71568 -1.00555 -0.50835
C  -3.60769 -2.95501 -0.92206
H  -4.61942 -2.55677 -1.06051
C  -3.36710 -4.33028 -1.07062
H  -4.18883 -5.00993 -1.32222
C  -2.06447 -4.83176 -0.90333
H  -1.86947 -5.90358 -1.02289
C  -1.00923 -3.96151 -0.58572
H   0.00487 -4.35812 -0.46649
C   1.53767 -2.12198 -0.94093
C   2.81169 -2.33784 -0.37205
C   1.37481 -2.36541 -2.32662
C   3.88828 -2.77424 -1.16211
C   2.44880 -2.80475 -3.11431
C   3.71521 -3.00649 -2.53680
H   2.96293 -2.17052  0.69942
H   0.39196 -2.21625 -2.78904
H   4.86605 -2.94300 -0.69621
H   2.29591 -2.99359 -4.18315
H   4.55497 -3.34960 -3.15061
C   0.50091 -1.77929  1.78231
C   1.43224 -0.96343  2.47038
C  -0.12907 -2.82324  2.49365
C   1.74302 -1.20472  3.81785
C   0.16967 -3.05098  3.84768
C   1.10990 -2.24882  4.51375
H   1.89828 -0.12911  1.93386
H  -0.85951 -3.46210  1.98809
H   2.47654 -0.57013  4.32838
H  -0.33279 -3.86544  4.38186
H   1.34494 -2.43274  5.56775
```

TS(I-III).PPh3

```
BP86
SCF  =       -1639.52952368
H(0 K)=      -1638.907358
H(298 K)=    -1638.864516
G(298 K)=    -1638.984378
SCF(C6H6) =        -1639.53615636
SCF(D3BJ) =        -1639.72800406
SCF(BS2) =         -3312.40643867
Low Freq. = -121.8965cm-1,
15.7812cm-1
B97D
SCF(C6H6,BS2)=  -3311.77618458
```

```
77
```

TS(I-III).PPh3

```
Ni -0.47022  0.12176  0.03032
P   1.52534  0.02169 -0.86576
N  -2.59403 -1.80741  1.02851
N  -2.50680 -1.70610 -1.12414
N  -1.82527  2.72914  0.65339
N  -1.24876  2.71361 -1.42309
C  -1.89947 -1.18259  0.00739
C  -3.59196 -2.67454  0.55300
C  -3.53504 -2.61073 -0.81881
C  -2.35328 -1.56557  2.44601
H  -1.46344 -0.92995  2.53948
H  -2.16050 -2.51253  2.97597
H  -3.22177 -1.06508  2.91141
C  -2.12564 -1.32950 -2.47795
H  -1.21347 -0.71741 -2.40063
H  -2.92277 -0.74239 -2.96889
H  -1.91125 -2.22210 -3.08989
C  -4.47665 -3.46978  1.45900
H  -3.90239 -4.17094  2.09256
H  -5.18994 -4.06694  0.86937
H  -5.06569 -2.82611  2.13822
C  -4.33303 -3.31908 -1.86646
H  -4.86648 -2.61559 -2.53208
H  -5.08961 -3.96761 -1.39739
H  -3.70184 -3.96039 -2.50953
C  -1.16927  1.93915 -0.27706
C  -2.30302  3.93008  0.10824
C  -1.93934  3.91918 -1.21701
C  -2.15817  4.92929 -2.29769
H  -1.20779  5.34377 -2.68282
H  -2.75322  5.77374 -1.91580
H  -2.70172  4.50455 -3.16148
C  -3.03258  4.95754  0.91338
H  -3.94709  4.54936  1.38144
H  -3.33732  5.79954  0.27216
H  -2.40729  5.37225  1.72608
C  -2.04851  2.32563  2.03475
H  -3.10263  2.03866  2.20118
```

```
H  -1.78857  3.14575  2.72476
H  -1.39877  1.46320  2.24250
C  -0.71181  2.30885 -2.71685
H  -0.02172  1.46655 -2.54802
H  -0.15488  3.14158 -3.17794
H  -1.51900  2.00147 -3.40671
C   2.33970 -1.61313 -1.22800
C   1.49516 -2.71151 -1.52849
H   0.41150 -2.59334 -1.40352
C   2.01934 -3.93264 -1.97564
H   1.34228 -4.76811 -2.18961
C   3.40493 -4.08602 -2.15469
H   3.81691 -5.03692 -2.50904
C   4.25363 -3.00047 -1.88119
H   5.33596 -3.10261 -2.02183
C   3.73121 -1.78031 -1.42468
H   4.41185 -0.94696 -1.22517
C   2.91792  1.08011 -0.22029
C   3.85304  0.66550  0.75849
C   3.00611  2.40401 -0.70847
C   4.85226  1.54123  1.21235
C   3.99869  3.28315 -0.24833
C   4.92951  2.85190  0.71196
H   3.79190 -0.34840  1.16526
H   2.28581  2.74219 -1.46239
H   5.56897  1.19968  1.96786
H   4.05095  4.30311 -0.64582
H   5.70914  3.53347  1.06936
C   0.79100 -0.68430  1.27013
C   1.01981  0.29178  2.28360
C   0.94295 -2.04558  1.64329
C   1.23020 -0.08006  3.62664
C   1.20077 -2.41321  2.97226
C   1.31977 -1.43545  3.98019
H   1.04780  1.35299  2.01172
H   0.83697 -2.82943  0.88679
H   1.36742  0.69805  4.38770
H   1.29780 -3.47572  3.22742
H   1.51432 -1.72818  5.01776
```

cis-III.PPh3

```
BP86
SCF  =      -1639.54802456
H(0 K)=     -1638.924754
H(298 K)=   -1638.881328
G(298 K)=   -1639.003488
SCF(C6H6) =       -1639.55701601
SCF(D3BJ) =       -1639.75301606
SCF(BS2) =        -3312.42338585
Low Freq. = 10.0984cm-1, 10.5349cm-1
B97D
SCF(C6H6,BS2)=  -3311.80031634
```

77

cis-III.PPh3

```
Ni  0.54439  0.14214 -0.14386
P  -1.38575  0.94267 -1.01582
N  -0.08581 -2.77643 -0.46464
N  -0.99298 -1.92626  1.29733
N   3.11623 -0.81974  1.04068
N   3.31057 -0.32950 -1.05155
C  -0.21057 -1.58478  0.21496
C  -0.78350 -3.82255  0.16022
C  -1.35987 -3.27933  1.28365
C   0.62500 -2.91477 -1.72754
H   1.02665 -1.92605 -1.98669
H  -0.06008 -3.24696 -2.52620
H   1.45366 -3.63887 -1.64049
C  -1.43332 -0.96871  2.30503
H  -1.15511  0.03471  1.94511
H  -0.95339 -1.16432  3.28071
H  -2.52749 -1.01441  2.42011
C  -0.83898 -5.20781 -0.39827
H  -1.33130 -5.23466 -1.38779
H  -1.41453 -5.86516  0.27179
H   0.16580 -5.65332 -0.51781
C  -2.25051 -3.88533  2.31953
H  -1.85890 -3.74332  3.34319
H  -2.35268 -4.96853  2.14987
H  -3.26467 -3.44705  2.28701
C   2.37987 -0.38502 -0.03805
C   4.47032 -1.01242  0.71747
C   4.59213 -0.69996 -0.61602
C   5.78255 -0.70771 -1.52039
H   5.65541 -1.40136 -2.37198
H   6.68051 -1.02462 -0.96757
H   5.98739  0.29344 -1.94104
C   5.48480 -1.46867  1.71624
H   5.57738 -0.76561  2.56436
H   6.47639 -1.54594  1.24385
H   5.24113 -2.46128  2.13820
C   2.55793 -1.01751  2.37158
H   3.03525 -0.34279  3.10252
H   2.69110 -2.06111  2.70535
H   1.48519 -0.78635  2.31143
C   3.00606  0.14190 -2.39880
H   1.91804  0.29387 -2.45981
H   3.32757 -0.59899 -3.15092
H   3.50084  1.10679 -2.59689
C   1.21925  1.97573 -0.16279
C   1.18799  2.84740 -1.28056
H   0.70409  2.51259 -2.20794
C   1.71836  4.15044 -1.23104
H   1.66273  4.79256 -2.11916
C   2.29952  4.63702 -0.04992
H   2.70655  5.65353 -0.00614
C   2.34904  3.79912  1.07702
H   2.79740  4.16148  2.01104
C   1.83211  2.49343  1.01042
H   1.90049  1.86476  1.90922
C  -2.16795  2.39365 -0.11888
C  -1.71537  2.91049  1.11516
H  -0.85267  2.44716  1.60284
C  -2.32926  4.02996  1.70307
H  -1.95063  4.41571  2.65680
C  -3.42008  4.65194  1.07656
H  -3.89981  5.52326  1.53589
C  -3.88638  4.15008 -0.15102
H  -4.73274  4.63030 -0.65560
C  -3.26232  3.04206 -0.74255
H  -3.62525  2.66817 -1.70701
C  -2.81534 -0.27063 -0.90096
C  -2.83219 -1.34388 -1.82709
```

```
C  -3.90128 -0.17991  0.00375
C  -3.86245 -2.29562 -1.83292
C  -4.93998 -1.12614 -0.00479
C  -4.92529 -2.19434 -0.91778
H  -2.02840 -1.40995 -2.57134
H  -3.93857  0.65352  0.71327
H  -3.85038 -3.10707 -2.57064
H  -5.77133 -1.02258  0.70312
H  -5.74012 -2.92648 -0.92973
```

TS(cis-trans-III).PPh3

```
BP86
SCF  =      -1639.52128482
H(0 K)=     -1638.898485
H(298 K)=   -1638.855825
G(298 K)=   -1638.975335
SCF(C6H6) =       -1639.52760308
SCF(D3BJ) =       -1639.72085326
SCF(BS2) =        -3312.39798311
Low Freq. = -35.4624cm-1,
14.4815cm-1
B97D
SCF(C6H6,BS2)=  -3311.76709489
```

77

TS(cis-trans-III).PPh3

```
Ni  0.42140 -0.28530  0.07309
P  -0.90019  1.38911 -0.26865
N  -0.93047 -2.83999 -1.01845
N  -1.98903 -2.13202  0.71740
N   3.10888 -1.40349 -0.48389
N   2.81336  0.46334 -1.54079
C  -0.88313 -1.84180 -0.05976
C  -2.02359 -3.70394 -0.85224
C  -2.69859 -3.25028  0.25713
C   0.01504 -2.93852 -2.12270
H   0.76600 -2.14707 -1.97986
H  -0.49227 -2.78544 -3.09152
H   0.50855 -3.92602 -2.13377
C  -2.44010 -1.30698  1.83584
H  -1.71410 -0.49225  1.96436
H  -2.49875 -1.90320  2.76209
H  -3.42943 -0.87383  1.61300
C  -2.29659 -4.86020 -1.76064
H  -2.44615 -4.54122 -2.80868
H  -3.20997 -5.38756 -1.44346
H  -1.47232 -5.59752 -1.75921
C  -3.94120 -3.75269  0.91984
H  -3.76298 -4.04567  1.97076
H  -4.32839 -4.63741  0.39025
H  -4.74015 -2.98923  0.92369
C   2.15944 -0.41453 -0.68834
C   4.30654 -1.14323 -1.16209
C   4.11753  0.04041 -1.83703
C   5.03787  0.80718 -2.73247
H   4.65968  0.86892 -3.76995
H   6.02443  0.31933 -2.77142
H   5.19229  1.84280 -2.37928
C   5.49280 -2.05156 -1.09870
H   5.83637 -2.20978 -0.06039
H   6.33343 -1.62047 -1.66449
H   5.28368 -3.04861 -1.52973
C   2.88485 -2.57804  0.35068
H   3.34242 -2.45485  1.34623
H   3.30461 -3.47208 -0.13992
H   1.79892 -2.69073  0.48033
C   2.24117  1.70749 -2.04387
H   1.14682  1.64414 -1.94079
H   2.51046  1.84337 -3.10359
H   2.59887  2.57578 -1.46393
C   1.22745 -0.29047  1.82688
C   2.17780  0.67214  2.24846
H   2.53918  1.42232  1.53626
C   2.67683  0.69304  3.56374
H   3.39989  1.46511  3.85521
C   2.25923 -0.26834  4.49881
H   2.64753 -0.25528  5.52317
C   1.34633 -1.25500  4.09390
H   1.02025 -2.02656  4.80300
C   0.84743 -1.26911  2.77713
H   0.15314 -2.06608  2.48618
C  -0.63542  3.22762 -0.14142
C   0.41279  3.69864  0.68674
H   0.96485  2.97919  1.30129
C   0.73002  5.06428  0.74576
H   1.53595  5.40637  1.40533
C   0.01134  5.99291 -0.02630
H   0.25858  7.05909  0.01885
C  -1.03188  5.54181 -0.85406
H  -1.59804  6.25752 -1.46139
C  -1.35293  4.17756 -0.91084
H  -2.16868  3.84061 -1.55942
C  -2.74562  1.30523 -0.47665
C  -3.29812  0.28986 -1.29662
C  -3.64476  2.14315  0.23178
C  -4.68668  0.11363 -1.39950
C  -5.03245  1.96919  0.12613
C  -5.56232  0.95238 -0.68850
H  -2.62017 -0.36142 -1.85894
H  -3.24730  2.93690  0.87311
H  -5.08740 -0.67329 -2.04902
H  -5.70510  2.62939  0.68576
H  -6.64646  0.82048 -0.77308
```

trans-III.PPh3

```
BP86
SCF  =      -1639.55694945
H(0 K)=     -1638.933496
H(298 K)=   -1638.889914
G(298 K)=   -1639.013181
SCF(C6H6) =       -1639.56405041
SCF(D3BJ) =       -1639.76205650
SCF(BS2) =        -3312.43208322
Low Freq. = 7.3487cm-1, 16.6965cm-1
B97D
SCF(C6H6,BS2)=  -3311.80790340
```

77

trans-III.PPh3

```
Ni  0.02561 -0.61816 -0.15278
P  -0.03552  1.52585 -0.98576
N  -2.76814 -0.78520 -1.12260
N  -2.64783 -0.89915  1.02954
N   2.76063 -1.17534 -1.07232
```

N   2.76269 -0.26574 0.88551
C -1.86362 -0.73027 -0.08702
C -4.08068 -0.98682 -0.66441
C -4.00319 -1.05723 0.70578
C -2.41275 -0.66155 -2.53220
H -1.35576 -0.36172 -2.58852
H -3.02407 0.11839 -3.01485
H -2.56303 -1.61864 -3.06227
C -2.11726 -0.94164 2.38476
H -1.04492 -0.70558 2.32211
H -2.23224 -1.94704 2.82342
H -2.62496 -0.19946 3.02399
C -5.25458 -1.07173 -1.58557
H -5.40424 -0.13623 -2.15545
H -6.17540 -1.25850 -1.01147
H -5.14946 -1.89160 -2.31947
C -5.06455 -1.24497 1.74116
H -4.88248 -2.13865 2.36555
H -6.04718 -1.37145 1.26085
H -5.13862 -0.37632 2.42113
C   1.91525 -0.65270 -0.12292
C   4.10337 -1.12488 -0.66493
C   4.10431 -0.53943 0.57950
C   5.22758 -0.17953 1.49706
H   5.28790 0.91257 1.65640
H   6.18886 -0.50773 1.07189
H   5.12347 -0.65525 2.48929
C   5.22599 -1.63710 -1.50887
H   5.11360 -2.71174 -1.74159
H   6.18482 -1.51105 -0.98243
H   5.30486 -1.09873 -2.47153
C   2.29519 -1.75922 -2.32483
H   2.34293 -2.86075 -2.29180
H   2.89916 -1.38689 -3.16865
H   1.24873 -1.45234 -2.46277
C   2.31819 0.42911 2.08741
H   1.23117 0.57255 1.99096
H   2.80165 1.41680 2.16222
H   2.53924 -0.16467 2.99121
C   0.06995 -2.54171 0.21499
C   0.57794 -3.07533 1.42692
H   0.97590 -2.39607 2.19300
C   0.60460 -4.45811 1.68742
H   1.00330 -4.82526 2.64182
C   0.13520 -5.36890 0.72700
H   0.15978 -6.44682 0.92242
C -0.36575 -4.87464 -0.48849
H -0.73856 -5.57061 -1.25091
C -0.40257 -3.48864 -0.72998
H -0.81975 -3.13947 -1.68404
C -1.52241 2.48985 -0.34810
C -2.24337 3.28521 -1.26786
H -1.89995 3.33040 -2.30804
C -3.37752 4.01573 -0.87308
H -3.91676 4.62516 -1.60744
C -3.81482 3.97088 0.46020
H -4.69680 4.54107 0.77217
C -3.10947 3.19182 1.39302
H -3.43861 3.15800 2.43846
C -1.98050 2.45897 0.99006
H -1.43517 1.85398 1.72260
C   1.35457 2.61468 -0.36763
C   2.64896 2.40194 -0.90860
C   1.20932 3.67257 0.56334
C   3.74747 3.17763 -0.51268
C   2.30642 4.45896 0.95169
C   3.58589 4.21306 0.42644
H   2.79039 1.61500 -1.65861
H   0.22367 3.88601 0.98893
H   4.73265 2.98483 -0.95369
H   2.15713 5.27139 1.67290
H   4.43989 4.82906 0.72827

^{3}III.PPh3
BP86
SCF =   -1639.51250475
H(0 K)=   -1638.890515
H(298 K)=   -1638.846329
G(298 K)=   -1638.972238
SCF(C6H6) =   -1639.52104505
SCF(D3BJ) =   -1639.71205217
SCF(BS2) =   -3312.38224866
Low Freq. = 11.6657cm-1, 17.9875cm-1
B97D
SCF(C6H6,BS2)= -3311.75745624

77
^{3}III.PPh3
Ni -0.49369 -0.06325 0.06398
P   1.23383 0.85749 -1.16146
N   1.01456 -2.63155 0.81816
N   0.07917 -2.76073 -1.12225
N -3.45116 -0.98213 0.12846
N -3.20207 0.73017 -1.15460
C   0.21060 -1.91423 -0.03739
C   1.39227 -3.87044 0.28159
C   0.79705 -3.95442 -0.95593
C   1.50846 -2.12979 2.09829
H   0.95133 -1.21541 2.34203
H   2.58250 -1.89070 2.02261
H   1.35377 -2.88428 2.88725
C -0.64015 -2.41392 -2.34399
H   0.06224 -2.17667 -3.16123
H -1.24809 -1.52302 -2.13002
H -1.29261 -3.24747 -2.65380
C   2.28324 -4.82754 1.00629
H   3.26328 -4.37412 1.23946
H   2.46658 -5.72135 0.38984
H   1.84262 -5.16758 1.96145
C   0.82852 -5.03673 -1.98752
H -0.17284 -5.46727 -2.17476
H   1.48267 -5.85841 -1.65678
H   1.21528 -4.67469 -2.95737
C -2.47503 -0.16098 -0.39443
C -4.74051 -0.61161 -0.27959
C -4.58115 0.48507 -1.09771
C -5.58825 1.31813 -1.82441
H -5.54379 2.37909 -1.51885
H -6.60688 0.95556 -1.61584
H -5.44332 1.28656 -2.91992
C -5.97259 -1.33876 0.15533
H -5.96328 -2.39877 -0.15845
H -6.86777 -0.87208 -0.28431

H -6.09647 -1.32390 1.25359
C -3.16536 -2.09956 1.02324
H -3.49709 -3.05320 0.57735
H -3.67127 -1.96176 1.99350
H -2.07740 -2.12087 1.18021
C -2.59678 1.82526 -1.90917
H -1.50754 1.67074 -1.91989
H -2.81284 2.79852 -1.43804
H -2.97449 1.83243 -2.94497
C 2.95033 0.33793 -0.69910
C 3.36755 -0.98213 -1.01572
H 2.68561 -1.64203 -1.56452
C 4.63758 -1.45301 -0.65433
H 4.93160 -2.47505 -0.92204
C 5.54188 -0.61545 0.02402
H 6.53822 -0.97879 0.29751
C 5.15137 0.69871 0.33419
H 5.84412 1.36598 0.86036
C 3.87726 1.16885 -0.01749
H 3.59198 2.19467 0.23676
C 1.33154 2.71690 -1.08673
C 0.40181 3.47066 -0.33112
C 2.25533 3.42451 -1.89692
C 0.38769 4.87470 -0.39337
C 2.25112 4.82566 -1.94621
C 1.31086 5.55894 -1.19952
H -0.29257 2.94522 0.33383
H 2.98057 2.86324 -2.49705
H -0.33756 5.43547 0.20786
H 2.97846 5.34864 -2.57794
H 1.30385 6.65358 -1.24307
C -0.46405 0.68813 1.92022
C -1.65133 0.80806 2.69048
C 0.70629 1.24751 2.50405
C -1.67741 1.43203 3.95225
C 0.69631 1.87149 3.76457
C -0.49882 1.96581 4.49774
H -2.59632 0.41522 2.29182
H 1.65457 1.19491 1.95383
H -2.62067 1.50310 4.50954
H 1.62420 2.29191 4.17268
H -0.51186 2.45244 5.47984

(vi) Alternative pathways

$[Ni(IMe_4)_2(PPh_2)(H)_2)]^+$
BP86
SCF = -1408.93551129
H(0 K)= -1408.382473
H(298 K)= -1408.343571
G(298 K)= -1408.454144
SCF(C6H6) = -1408.96523444
SCF(D3BJ) = -1409.10676052
SCF(BS2) = -3081.72701161
Low Freq. = 12.4200cm-1, 22.9075cm-1
B97D
SCF(C6H6,BS2)= -3081.29658555

68
$[Ni(IMe_4)_2(PPh_2)(H)_2)]^+$
Ni 0.09271 -1.26414 0.19223
N 2.89494 -1.12147 1.13730
N 2.84231 -1.46188 -0.99617
N -2.70754 -1.93955 -0.40875
N -2.59661 -0.67460 1.33994
C 2.02565 -1.25491 0.08560
C 4.23098 -1.22218 0.72607
C 4.19735 -1.44354 -0.63308
C 2.48228 -0.89681 2.51960
H 2.88349 -1.68590 3.17618
H 1.38232 -0.92233 2.55160
H 2.83091 0.08664 2.87384
C 2.37733 -1.74295 -2.35540
H 1.30095 -1.53013 -2.40339
H 2.56904 -2.79677 -2.61898
H 2.89606 -1.09131 -3.07528
C 5.38032 -1.08988 1.67241
H 5.39743 -0.10037 2.16284
H 6.33196 -1.20699 1.13308
H 5.35457 -1.85661 2.46726
C 5.30234 -1.63948 -1.62052
H 5.22765 -2.61200 -2.13860
H 6.27646 -1.61171 -1.11004
H 5.31128 -0.84999 -2.39299
C -1.81609 -1.26203 0.38197
C -4.02710 -1.76881 0.03455
C -3.95543 -0.96008 1.14810
C -5.02590 -0.41994 2.03988
H -4.87732 -0.71625 3.09344
H -6.00992 -0.79909 1.72644
H -5.06717 0.68314 2.00344
C -5.19742 -2.39940 -0.64863
H -5.30147 -2.05649 -1.69350
H -6.12858 -2.14047 -0.12313
H -5.12301 -3.50138 -0.66606
C -2.32646 -2.74143 -1.56968
H -2.84319 -2.38349 -2.47484
H -2.57409 -3.80397 -1.41042
H -1.24082 -2.63556 -1.71347
C -2.08249 0.17132 2.41325
H -0.98793 0.20701 2.31198
H -2.34678 -0.24763 3.39786
H -2.48418 1.19273 2.32219
P 0.04616 0.61029 -1.04814
C -1.52658 1.60041 -0.82598
C -1.66996 2.72891 0.01312
C -2.61489 1.24102 -1.65769
H -0.83141 3.05601 0.63547
H -2.50473 0.40075 -2.35344
C -2.87130 3.45682 0.03621
C -3.81637 1.96373 -1.62970
H -2.95867 4.33592 0.68379
H -4.64134 1.67608 -2.29000
C -3.95024 3.07362 -0.77782
H -4.88122 3.64939 -0.76563
H 0.52621 -2.88053 0.66572
H -0.22096 -2.80260 0.94376
C 1.31762 1.81235 -0.37713
C 1.53261 2.09544 0.99112
C 2.09673 2.50036 -1.33419
H 0.93461 1.57881 1.74958
H 1.94050 2.30131 -2.40056
C 2.49362 3.03868 1.39029

```
C   3.05832  3.44486 -0.93657
H   2.63756  3.25482  2.45472
H   3.64693  3.97228 -1.69435
C   3.25986  3.71514  0.42571
H   4.00610  4.45346  0.73589
```

PPh_2^-

```
BP86
SCF  =       -469.884386255
H(0 K)=      -469.710036
H(298 K)=    -469.697911
G(298 K)=    -469.748156
SCF(C6H6) =        -469.925647985
SCF(D3BJ) =        -469.932966137
SCF(BS2) =         -804.883664092
Low Freq. = 44.3018cm-1, 48.7096cm-1
B97D
SCF(C6H6,BS2)=  -804.535425718
```

23

PPh_2^-

```
P  -0.00002  1.59217  0.00015
C   1.46792  0.47619  0.01279
C   2.70732  1.03924 -0.42519
C   1.54507 -0.85659  0.52130
C   3.91500  0.33585 -0.36538
C   2.75195 -1.56719  0.56524
C   3.95652 -0.98649  0.12257
H   2.69918  2.06113 -0.82798
H   0.63626 -1.32843  0.90994
H   4.83733  0.81725 -0.71916
H   2.75582 -2.59002  0.96745
H   4.89864 -1.54641  0.15590
C  -1.46791  0.47616 -0.01270
C  -1.54499 -0.85668 -0.52107
C  -2.70741  1.03926  0.42499
H  -0.63611 -1.32857 -0.90951
H  -2.69936  2.06120  0.82766
C  -2.75185 -1.56728 -0.56513
C  -3.91507  0.33586  0.36504
H  -2.75564 -2.59017 -0.96723
H  -4.83746  0.81732  0.71860
C  -3.95651 -0.98655 -0.12274
H  -4.89861 -1.54648 -0.15616
```

$Ni(IMe_4)_2(PPh_2)$

```
BP86
SCF  =       -1407.91040864
H(0 K)=      -1407.375652
H(298 K)=    -1407.337465
G(298 K)=    -1407.449475
SCF(C6H6) =        -1407.91741509
SCF(D3BJ) =        -1408.07140506
SCF(BS2) =         -3080.70636966
Low Freq. = 10.8737cm-1, 18.6873cm-1
B97D
SCF(C6H6,BS2)=  -3080.23739717
```

66

$Ni(IMe_4)_2(PPh_2)$

```
Ni  0.13318 -0.94778 -0.12447
N   2.69688 -1.78113  1.07258
N   2.99166 -1.23322 -0.99620
N  -2.43928 -2.27201 -0.73454
N  -2.61353 -1.03873  1.02923
C   2.00284 -1.31180 -0.03193
C   4.05664 -1.98286  0.80620
C   4.24336 -1.63508 -0.51342
C   2.05987 -2.02885  2.35938
H   2.22949 -3.06862  2.68877
H   0.97947 -1.85943  2.21395
H   2.43987 -1.33970  3.13377
C   2.74977 -0.78561 -2.36365
H   1.71862 -0.40176 -2.41327
H   2.87294 -1.61817 -3.07892
H   3.44550  0.02632 -2.63218
C   5.02225 -2.48103  1.83355
H   5.08102 -1.80856  2.70915
H   6.03395 -2.55316  1.40467
H   4.75026 -3.48444  2.21022
C   5.47619 -1.64043 -1.35938
H   5.36854 -2.29231 -2.24566
H   6.33698 -2.00889 -0.77978
H   5.73135 -0.62935 -1.72615
C  -1.69493 -1.43684  0.07969
C  -3.77182 -2.37945 -0.31301
C  -3.88178 -1.58833  0.80919
C  -5.05622 -1.28144  1.68147
H  -4.88486 -1.57898  2.73239
H  -5.94980 -1.81849  1.32661
H  -5.29030 -0.20137  1.67839
C  -4.78442 -3.21993 -1.02281
H  -4.92446 -2.90499 -2.07341
H  -5.76249 -3.14317 -0.52251
H  -4.50373 -4.28954 -1.03757
C  -1.88190 -2.93546 -1.90528
H  -2.37000 -2.58632 -2.83186
H  -1.99313 -4.03143 -1.83074
H  -0.81111 -2.67589 -1.94147
C  -2.29542 -0.10133  2.09995
H  -1.22775  0.14827  2.00190
H  -2.48573 -0.55693  3.08749
H  -2.88566  0.82384  1.99582
P  -0.01867  1.15638 -0.91888
C  -1.60068  2.03055 -0.47984
C  -1.71367  3.11580  0.42749
C  -2.79753  1.56221 -1.08400
H  -0.81218  3.50832  0.90908
H  -2.73895  0.73615 -1.80292
C  -2.95843  3.69464  0.71913
C  -4.04242  2.13169 -0.78388
H  -3.01079  4.53381  1.42327
H  -4.94593  1.74767 -1.27243
C  -4.13414  3.20488  0.12257
H  -5.10435  3.66007  0.34942
C   1.24628  2.38594 -0.28602
C   2.21009  2.05400  0.69432
C   1.33015  3.67242 -0.87693
H   2.15726  1.06912  1.16940
H   0.60088  3.95458 -1.64515
C   3.21959  2.96047  1.06199
C   2.32648  4.58488 -0.50058
```

H 3.95340 2.67589 1.82570
H 2.36369 5.57368 -0.97250
C 3.28220 4.23112 0.46887
H 4.06499 4.94068 0.75880

PPh_2
BP86
SCF = -469.833721228
H(0 K)= -469.657234
H(298 K)= -469.645141
G(298 K)= -469.696372
SCF(C6H6) = -469.835657562
SCF(D3BJ) = -469.882669290
SCF(BS2) = -804.822000433
Low Freq. = 31.7503cm-1, 48.3119cm-1
B97D
SCF(C6H6,BS2)= -804.444396553

23
PPh_2
P -0.00010 1.61622 0.00046
C 1.45366 0.48752 0.03148
C 2.66609 1.00951 -0.49300
C 1.48418 -0.79790 0.63321
C 3.85237 0.26659 -0.44458
C 2.67439 -1.53554 0.68511
C 3.86078 -1.01090 0.14242
H 2.66705 2.00607 -0.95030
H 0.57151 -1.20881 1.07557
H 4.77348 0.68379 -0.86540
H 2.67910 -2.52311 1.15924
H 4.78834 -1.59131 0.18401
C -1.45359 0.48728 -0.03133
C -1.48383 -0.79846 -0.63235
C -2.66632 1.00958 0.49223
H -0.57095 -1.20978 -1.07387
H -2.66747 2.00636 0.94905
C -2.67409 -1.53601 -0.68461
C -3.85264 0.26677 0.44343
H -2.67856 -2.52384 -1.15820
H -4.77396 0.68425 0.86348
C -3.86079 -1.01101 -0.14296
H -4.78837 -1.59136 -0.18485

$Ni(IMe_4)_2(PPh_2)(H)_2$
BP86
SCF = -1409.05906386
H(0 K)= -1408.510028
H(298 K)= -1408.471429
G(298 K)= -1408.582563
SCF(C6H6) = -1409.06655630
SCF(D3BJ) = -1409.22643834
SCF(BS2) = -3081.85535845
Low Freq. = 11.8874cm-1, 18.0677cm-1
B97D
SCF(C6H6,BS2)= -3081.38962258

68
$Ni(IMe_4)_2(PPh_2)(H)_2$
Ni 0.30757 -1.15958 -0.23155
N 3.04722 -1.42281 0.85120
N 3.12429 -0.96839 -1.25983
N -2.45123 -2.26532 -0.13174
N -2.15460 -0.74829 1.37769
C 2.23348 -1.14440 -0.22772
C 4.40610 -1.40417 0.50676
C 4.45417 -1.11715 -0.83722
C 2.54407 -1.71000 2.18795
H 2.99349 -2.63875 2.57717
H 1.45405 -1.84475 2.10265
H 2.76474 -0.88349 2.88653
C 2.74607 -0.69656 -2.64572
H 1.65836 -0.83728 -2.72424
H 3.26565 -1.40100 -3.31699
H 3.00140 0.33783 -2.92691
C 5.49785 -1.65930 1.49577
H 5.47165 -0.94321 2.33745
H 6.48135 -1.56224 1.01040
H 5.44211 -2.67569 1.92772
C 5.61820 -0.96343 -1.76244
H 5.61128 -1.71750 -2.57083
H 6.56287 -1.08150 -1.20928
H 5.63434 0.03160 -2.24261
C -1.50368 -1.39083 0.34888
C -3.66074 -2.16507 0.57096
C -3.47225 -1.20046 1.53171
C -4.40894 -0.63273 2.54861
H -4.00719 -0.70800 3.57540
H -5.36798 -1.17343 2.52714
H -4.62499 0.43372 2.35334
C -4.86337 -2.98539 0.23217
H -5.24189 -2.76652 -0.78328
H -5.67955 -2.77363 0.94022
H -4.65461 -4.06971 0.27826
C -2.22599 -3.22036 -1.21119
H -3.02876 -3.14145 -1.96282
H -2.19575 -4.25201 -0.81897
H -1.25562 -2.96960 -1.66935
C -1.54183 0.26412 2.22759
H -0.61486 0.59853 1.73904
H -1.30907 -0.14610 3.22662
H -2.21589 1.12766 2.33411
P 0.08800 1.00124 -1.07945
C -1.71315 1.48743 -1.11321
C -2.32308 2.49246 -0.32399
C -2.53538 0.80338 -2.04618
H -1.71951 3.05153 0.39888
H -2.08354 0.03111 -2.68049
C -3.68806 2.79782 -0.46099
C -3.89855 1.10034 -2.17552
H -4.12807 3.58824 0.15929
H -4.50409 0.55693 -2.91067
C -4.48718 2.10333 -1.38355
H -5.54979 2.34588 -1.49265
H 0.45280 -2.57869 0.18379
H 0.15435 -1.73140 -1.67298
C 0.83505 2.33694 -0.00163
C 1.65018 2.05470 1.12037
C 0.72226 3.69705 -0.39421
H 1.75935 1.01362 1.44282
H 0.11809 3.95005 -1.27287
C 2.32099 3.07444 1.81749

C 1.37259 4.71827 0.31178
H 2.94656 2.82063 2.68181
H 1.25864 5.75877 -0.01389
C 2.18148 4.41328 1.42212
H 2.69665 5.21074 1.96839

[Ni(IMe$_4$)$_2$(PPh$_2$)(H)$_2$]$^-$
BP86
SCF = -1409.09925310
H(0 K)= -1408.554539
H(298 K)= -1408.515224
G(298 K)= -1408.627895
SCF(C6H6) = -1409.13891791
SCF(D3BJ) = -1409.25942043
SCF(BS2) = -3081.90832054
Low Freq. = 10.0947cm-1, 15.7179cm-1
B97D
SCF(C6H6,BS2)= -3081.46334318

68
[Ni(IMe$_4$)$_2$(PPh$_2$)(H)$_2$]$^-$
Ni 0.29568 -0.71716 -0.88334
N 2.83296 -1.57456 0.46318
N 3.23221 -0.24347 -1.19502
N -1.59230 -2.96196 -0.29387
N -1.79919 -1.40225 1.18698
C 2.15736 -0.84058 -0.52443
C 4.22593 -1.44745 0.38677
C 4.47997 -0.60569 -0.67065
C 2.14083 -2.38989 1.44375
H 2.48443 -3.44123 1.40388
H 1.06992 -2.33393 1.17923
H 2.29191 -2.00599 2.47017
C 3.04899 0.64158 -2.33461
H 2.01791 1.03370 -2.28838
H 3.18348 0.10287 -3.29340
H 3.76784 1.47730 -2.28840
C 5.16085 -2.12493 1.33747
H 4.99210 -1.81168 2.38607
H 6.20729 -1.88360 1.08615
H 5.06075 -3.22733 1.31408
C 5.77766 -0.09854 -1.21457
H 5.90869 -0.34599 -2.28520
H 6.62451 -0.54356 -0.66577
H 5.86892 1.00137 -1.12621
C -1.03601 -1.72378 0.05977
C -2.64884 -3.35327 0.54138
C -2.77429 -2.36166 1.48589
C -3.72657 -2.22307 2.63073
H -3.20537 -2.15429 3.60497
H -4.40357 -3.09260 2.67782
H -4.35395 -1.31646 2.53848
C -3.42998 -4.61346 0.34618
H -3.91990 -4.65346 -0.64595
H -4.22318 -4.69390 1.10858
H -2.80155 -5.52212 0.42900
C -1.13254 -3.70782 -1.44949
H -1.78919 -3.55633 -2.32817
H -1.08062 -4.78882 -1.22292
H -0.12834 -3.31834 -1.69494
C -1.57623 -0.19659 1.96975
H -0.83009 0.40134 1.42034
H -1.19817 -0.43995 2.98127
H -2.50453 0.39254 2.05745
P -0.31475 1.57932 -0.97567
C -2.13214 1.93054 -0.94780
C -2.86538 2.62669 0.05791
C -2.90522 1.39756 -2.02333
H -2.32981 3.03997 0.91969
H -2.38245 0.84065 -2.81135
C -4.25579 2.79787 -0.02242
C -4.29191 1.56215 -2.09951
H -4.77440 3.34497 0.77729
H -4.83831 1.13692 -2.95225
C -4.99034 2.27503 -1.10244
H -6.07620 2.41321 -1.16493
H 0.48749 -0.82931 -2.51808
H -0.35321 -0.97262 -2.38128
C 0.36102 2.72774 0.31883
C 1.44716 2.29515 1.13255
C -0.02511 4.09309 0.46023
H 1.76249 1.24758 1.04973
H -0.83610 4.48186 -0.16609
C 2.10738 3.16512 2.01337
C 0.61791 4.95566 1.35706
H 2.94289 2.78737 2.61868
H 0.28519 5.99974 1.43319
C 1.69777 4.50424 2.14364
H 2.20461 5.18233 2.84077

PPh$_2^+$
BP86
SCF = -469.591857565
H(0 K)= -469.414205
H(298 K)= -469.402165
G(298 K)= -469.452343
SCF(C6H6) = -469.627808791
SCF(D3BJ) = -469.640512271
SCF(BS2) = -804.574493254
Low Freq. = 46.0610cm-1, 48.8140cm-1
B97D
SCF(C6H6,BS2)= -804.236477749

23
PPh$_2^+$
P -0.00001 1.47399 0.00006
C 1.45027 0.42634 0.04608
C 2.64953 1.10528 -0.35212
C 1.53549 -0.92507 0.50897
C 3.87022 0.42982 -0.36387
C 2.76694 -1.57650 0.52367
C 3.92837 -0.90851 0.07326
H 2.59980 2.15384 -0.66721
H 0.64718 -1.42665 0.90098
H 4.77895 0.94171 -0.69242
H 2.83965 -2.60107 0.89987
H 4.89035 -1.43061 0.08987
C -1.45025 0.42631 -0.04608
C -1.53547 -0.92512 -0.50891
C -2.64953 1.10528 0.35205
H -0.64715 -1.42674 -0.90084
H -2.59980 2.15385 0.66711

```
C  -2.76693 -1.57653 -0.52363
C  -3.87024  0.42984  0.36376
H  -2.83963 -2.60112 -0.89978
H  -4.77897  0.94177  0.69224
C  -3.92838 -0.90850 -0.07332
H  -4.89037 -1.43060 -0.08995
```

(vii) Reaction with Ph_2P-PPh_2

Ph_2PPh_2

```
BP86
SCF  =      -939.727613395
H(0 K)=     -939.371940
H(298 K)=   -939.347423
G(298 K)=   -939.428245
SCF(C6H6) =       -939.730953677
SCF(D3BJ) =       -939.852335267
SCF(BS2) =        -1609.70529117
Low Freq. = 14.6562cm-1, 23.2086cm-1
B97D
SCF(C6H6,BS2)=  -1608.95910900
```

46

Ph_2PPh_2

```
C  -5.10674  0.70593 -1.18479
C  -4.26058 -0.39564 -0.97523
C  -2.90502 -0.19893 -0.66603
C  -2.37565  1.10782 -0.56438
C  -3.23590  2.20664 -0.78384
C  -4.59247  2.00839 -1.08777
P  -0.59446  1.53131 -0.18829
C  -0.34293  0.91352  1.55465
C   0.88504  1.26107  2.16928
C   1.15571  0.87926  3.49132
C   0.19640  0.16625  4.23142
C  -1.03176 -0.16410  3.63742
C  -1.30021  0.20095  2.30788
H   1.63131  1.83093  1.60462
H   2.11337  1.15072  3.94859
H   0.40277 -0.12144  5.26773
H  -1.78823 -0.71251  4.20948
H  -2.26125 -0.06364  1.85711
H  -2.25604 -1.06527 -0.50596
H  -2.83387  3.22406 -0.71830
H  -4.65572 -1.41429 -1.05418
H  -5.24540  2.87203 -1.25298
H  -6.16338  0.54869 -1.42615
P   0.49970  0.05214 -1.58597
C   0.39581 -1.65864 -0.83597
C   2.25327  0.58663 -1.20377
C   3.29316 -0.33524 -0.95279
C   4.62100  0.10289 -0.81596
C   4.93592  1.46560 -0.92769
C   3.91135  2.39085 -1.19057
C   2.58578  1.95672 -1.33610
H   3.06690 -1.40181 -0.86325
H   5.41161 -0.62951 -0.61901
H   5.97117  1.80476 -0.81698
H   4.14412  3.45690 -1.28728
H   1.79877  2.68865 -1.54897
C  -0.20519 -2.64679 -1.65023
C  -0.35206 -3.96597 -1.19129
C   0.10359 -4.31683  0.08938
C   0.70419 -3.34459  0.90738
C   0.84890 -2.02455  0.45294
H  -0.55541 -2.37411 -2.65250
H  -0.81616 -4.71915 -1.83705
H  -0.00666 -5.34532  0.44941
H   1.05882 -3.61295  1.90834
H   1.31499 -1.27607  1.09961
```

VI

```
BP86
SCF  =      -1877.78080638
H(0 K)=     -1877.068513
H(298 K)=   -1877.017544
G(298 K)=   -1877.158377
SCF(C6H6) =       -1877.78695508
SCF(D3BJ) =       -1878.02815280
SCF(BS2) =        -3885.56864855
Low Freq. = 11.7085cm-1, 15.4492cm-1
B97D
SCF(C6H6,BS2)=  -3884.73795196
```

89

VI

```
C  -1.09999  3.28719  3.86922
C  -1.13437  1.92609  4.21051
C  -1.06245  0.94008  3.21177
C  -0.94864  1.29638  1.84831
C  -0.89939  2.67239  1.51853
C  -0.98452  3.65469  2.51608
P  -0.61107  0.01714  0.51835
C  -1.51994 -1.49967  1.17038
C  -2.74724 -1.48061  1.87155
C  -3.33423 -2.67764  2.31252
C  -2.71774 -3.91148  2.04424
C  -1.50579 -3.94342  1.33522
C  -0.91008 -2.74578  0.90817
Ni  1.47044  0.04018 -0.04348
C   2.04104  1.80199 -0.51940
N   1.94850  2.53804 -1.69286
C   2.46561  3.83655 -1.55889
C   2.90517  3.94380 -0.26011
N   2.64590  2.70506  0.34376
C   1.34438  2.02558 -2.91303
C   2.92714  2.38169  1.73523
C   2.48642  4.82204 -2.68330
C   3.54227  5.08656  0.46397
C   2.55453 -1.48135  0.26391
N   3.34822 -2.16872 -0.64945
C   4.05086 -3.23464 -0.06696
C   3.71274 -3.23387  1.26623
N   2.80737 -2.17666  1.44313
C   3.43517 -1.78103 -2.04756
C   2.21466 -1.81003  2.71983
C   4.96151 -4.13264 -0.84210
C   4.15204 -4.12259  2.38584
H   3.04902 -0.74988 -2.11574
H   2.82892 -2.43782 -2.69770
H   4.48237 -1.80777 -2.39431
H   1.45644 -1.03800  2.51391
```

```
H   2.97062 -1.39915  3.41375
H   1.72857 -2.67945  3.19426
H   4.43744 -4.63935 -1.67370
H   5.37514 -4.91563 -0.18693
H   5.81716 -3.58648 -1.28169
H   4.65271 -3.55762  3.19412
H   4.86682 -4.87617  2.01915
H   3.30575 -4.66593  2.84607
H   2.96377  5.38505  1.35766
H   3.61117  5.96646 -0.19525
H   4.56777  4.84935  0.80433
H   3.09308  4.46900 -3.53833
H   2.91829  5.77780 -2.34654
H   1.47276  5.03458 -3.07073
H   2.05857  2.05735 -3.75525
H   0.44315  2.60216 -3.18533
H   1.04634  0.98329 -2.72824
H   2.89677  1.28236  1.81843
H   2.16822  2.81238  2.41148
H   3.92684  2.74873  2.02411
H  -3.24530 -0.52823  2.07595
H  -4.28321 -2.64508  2.85914
H  -3.18124 -4.84343  2.38647
H  -1.01923 -4.90171  1.12029
H   0.05046 -2.75488  0.37836
H  -1.10216 -0.11639  3.49574
H  -0.78738  2.96789  0.47017
H  -1.22308  1.62491  5.26055
H  -0.95950  4.71372  2.23498
H  -1.16391  4.05492  4.64779
P  -2.05953  0.87234 -1.12196
C  -3.81071  0.52150 -0.58465
C  -1.81640 -0.36688 -2.50264
C  -2.76223 -0.38678 -3.55701
C  -2.56947 -1.19583 -4.68638
C  -1.42015 -1.99700 -4.79665
C  -0.46959 -1.97800 -3.76463
C  -0.66193 -1.17352 -2.62855
H  -3.66114  0.23608 -3.48977
H  -3.31978 -1.19686 -5.48493
H  -1.26936 -2.62944 -5.67816
H   0.42863 -2.60249 -3.83597
H   0.09383 -1.16814 -1.82608
C  -4.47024  1.56312  0.11229
C  -5.78155  1.39710  0.58228
C  -6.46654  0.19197  0.35000
C  -5.82442 -0.84818 -0.34343
C  -4.50639 -0.69150 -0.79799
H  -3.94538  2.50970  0.28486
H  -6.27403  2.21519  1.11953
H  -7.49484  0.06523  0.70540
H  -6.34950 -1.79290 -0.52379
H  -4.01178 -1.51240 -1.32559
```

TS(VI-cis-V)

```
BP86
SCF  =       -1877.77059568
H(0 K)=      -1877.057976
H(298 K)=    -1877.007964
G(298 K)=    -1877.145649
SCF(C6H6) =        -1877.77702785
SCF(D3BJ) =        -1878.01814677
SCF(BS2) =        -3885.55900958
Low Freq. = -47.0347cm-1,
11.5249cm-1
B97D
SCF(C6H6,BS2)=  -3884.72617236
```

89

TS(VI-cis-V)

```
C   3.61982 -3.51195  2.06225
C   3.49894 -2.41685  2.93192
C   2.69545 -1.31771  2.58587
C   1.98685 -1.29436  1.36176
C   2.09675 -2.41757  0.50842
C   2.91227 -3.50581  0.84746
P   0.71510  0.02110  0.94261
C   1.32976  1.48281  1.95615
C   2.68557  1.84617  2.12183
C   3.03659  2.97185  2.88307
C   2.04007  3.75147  3.49576
C   0.69013  3.40003  3.34065
C   0.34072  2.27596  2.57456
Ni -1.22158 -0.47149  0.15844
C  -1.41636 -2.33211 -0.22540
N  -1.50841 -3.00343 -1.43711
C  -1.76649 -4.37472 -1.27986
C  -1.82444 -4.60155  0.07499
N  -1.59952 -3.36025  0.68896
C  -1.41058 -2.33133 -2.72431
C  -1.56693 -3.14755  2.12804
C  -1.91488 -5.31520 -2.43310
C  -2.05023 -5.86271  0.84603
C  -2.71095  0.69762  0.02545
N  -3.22985  1.41317 -1.04802
C  -4.43285  2.06620 -0.73486
C  -4.70031  1.77359  0.58130
N  -3.64657  0.95829  1.02217
C  -2.61172  1.44035 -2.36474
C  -3.54688  0.38599  2.35464
C  -5.18225  2.90252 -1.72229
C  -5.83342  2.18832  1.46461
H  -1.66538  0.88258 -2.29724
H  -2.38957  2.47432 -2.67802
H  -3.26377  0.96455 -3.12002
H  -2.52440 -0.01475  2.45373
H  -4.27107 -0.43677  2.50042
H  -3.72155  1.15449  3.12744
H  -4.58263  3.75872 -2.08367
H  -6.09676  3.31042 -1.26324
H  -5.48916  2.32319 -2.61290
H  -6.38656  1.32023  1.86886
H  -6.55221  2.80376  0.90075
H  -5.49404  2.78861  2.32985
H  -1.17492 -6.13828  1.46373
H  -2.24421 -6.70176  0.15915
H  -2.91763 -5.78455  1.52722
H  -2.73071 -5.01416 -3.11595
H  -2.14533 -6.32970 -2.07098
H  -0.99215 -5.38479 -3.03968
H  -2.40846 -2.16445 -3.17061
H  -0.80400 -2.92866 -3.42564
H  -0.91736 -1.36164 -2.55831
H  -1.10621 -2.16096  2.29731
```

```
H  -0.96031 -3.92469  2.62163
H  -2.58333 -3.15433  2.56287
H   3.46991  1.23720  1.66160
H   4.09236  3.24158  2.99959
H   2.31683  4.62835  4.09110
H  -0.09251  4.00174  3.81635
H  -0.71177  1.99911  2.43799
H   2.61457 -0.47587  3.28001
H   1.51750 -2.43900 -0.42033
H   4.03521 -2.41213  3.88783
H   2.99304 -4.35601  0.16062
H   4.25352 -4.36435  2.32988
P   1.07466  0.55145 -1.32537
C   2.89099  0.21082 -1.66853
C   1.01776  2.42363 -1.38786
C   1.88101  3.15974 -2.23415
C   1.71226  4.54271 -2.40604
C   0.67968  5.22233 -1.73977
C  -0.19100  4.49998 -0.90753
C  -0.03058  3.11613 -0.73889
H   2.68962  2.65008 -2.76669
H   2.39537  5.09009 -3.06546
H   0.55321  6.30244 -1.87102
H  -1.00215  5.01602 -0.38121
H  -0.72685  2.55378 -0.10621
C   3.18095 -0.90741 -2.48154
C   4.50695 -1.26640 -2.77439
C   5.56959 -0.50777 -2.26038
C   5.29717  0.61213 -1.45460
C   3.97271  0.96717 -1.15951
H   2.35329 -1.49556 -2.89540
H   4.70869 -2.13616 -3.40949
H   6.60488 -0.78328 -2.48809
H   6.12197  1.21225 -1.05390
H   3.77071  1.84837 -0.54289
```

³VI

```
BP86
SCF  =      -1877.75027946
H(0 K)=     -1877.037975
H(298 K)=   -1876.986573
G(298 K)=   -1877.130997
SCF(C6H6) =       -1877.75861150
SCF(D3BJ) =       -1877.98983789
SCF(BS2) =        -3885.53199845
Low Freq. = 11.3219cm-1, 12.9660cm-1
B97D
SCF(C6H6,BS2)=  -3884.70536752
```

89

³VI

```
C  -1.10655  4.58681 -2.60444
C   0.22890  4.26757 -2.30351
C   0.55036  3.03720 -1.71091
C  -0.45796  2.08385 -1.41797
C  -1.79658  2.42028 -1.73062
C  -2.11805  3.65750 -2.31179
P  -0.04249  0.49660 -0.53708
C   1.24388 -0.26145 -1.62854
C   2.00161 -1.33855 -1.09503
C   2.95642 -2.01059 -1.87186
C   3.19485 -1.62064 -3.20299
C   2.46587 -0.54369 -3.74059
C   1.50443  0.12512 -2.96851
Ni -1.76095 -0.85494  0.01181
C  -3.17502  0.29244  0.61696
N  -3.21772  1.29573  1.56281
C  -4.50251  1.83207  1.71269
C  -5.31436  1.14943  0.83340
N  -4.48887  0.22431  0.18338
C  -2.05254  1.75539  2.31420
C  -4.94137 -0.71070 -0.83930
C  -4.81297  2.93461  2.67362
C  -6.77594  1.27911  0.54556
C  -1.04676 -2.59220 -0.37432
N  -0.66878 -3.56228  0.53086
C  -0.15171 -4.70216 -0.09648
C  -0.21293 -4.45345 -1.45083
N  -0.76655 -3.17645 -1.59040
C  -0.77601 -3.38678  1.97471
C  -0.98424 -2.50989 -2.86991
C   0.34325 -5.89301  0.66005
C   0.21150 -5.28198 -2.62027
H  -1.32839 -2.44883  2.14627
H   0.22056 -3.30200  2.43936
H  -1.32732 -4.22797  2.42875
H  -1.40234 -1.51567 -2.65005
H  -1.68982 -3.08561 -3.49384
H  -0.03097 -2.38113 -3.40753
H   1.16965 -5.63217  1.34584
H   0.71959 -6.65820 -0.03660
H  -0.45252 -6.36180  1.26805
H  -0.62753 -5.49398 -3.30809
H   0.61343 -6.24893 -2.27986
H   1.00095 -4.77817 -3.20675
H  -6.97020  1.54261 -0.51040
H  -7.21907  2.07073  1.16943
H  -7.32709  0.34409  0.75633
H  -4.57191  2.65547  3.71542
H  -5.88443  3.18548  2.63619
H  -4.24698  3.85525  2.44273
H  -2.15862  1.51707  3.38659
H  -1.92258  2.84325  2.19767
H  -1.15902  1.25631  1.91195
H  -4.06788 -1.31818 -1.12937
H  -5.32508 -0.17593 -1.72526
H  -5.73317 -1.37231 -0.44751
H   1.83964 -1.63962 -0.05402
H   3.52939 -2.83377 -1.42954
H   3.94757 -2.13832 -3.80732
H   2.64545 -0.22221 -4.77335
H   0.94425  0.95691 -3.40728
H   1.59344  2.81294 -1.46689
H  -2.58541  1.69170 -1.51725
H   1.02829  4.98503 -2.51997
H  -3.16288  3.89090 -2.54875
H  -1.35523  5.55005 -3.06327
P   1.76130  1.67511  1.90055
C   3.09750  2.45111  0.87956
C   2.52617  0.13201  2.55505
C   3.89909 -0.22876  2.49337
C   4.37707 -1.38099  3.13251
C   3.50835 -2.21233  3.86174
```

```
C   2.14598 -1.86859  3.94462
C   1.66380 -0.72247  3.29913
H   4.60060  0.41532  1.95494
H   5.44391 -1.62578  3.07095
H   3.88842 -3.10529  4.36928
H   1.45899 -2.49050  4.53165
H   0.60108 -0.46266  3.38167
C   3.26382  3.85448  1.00309
C   4.19228  4.54978  0.21513
C   4.97853  3.86016 -0.72436
C   4.81749  2.47160 -0.87247
C   3.88599  1.77455 -0.08728
H   2.65787  4.39894  1.73700
H   4.30672  5.63281  0.33816
H   5.70587  4.40079 -1.33965
H   5.41138  1.92636 -1.61465
H   3.74937  0.69963 -0.24070
```

^{3}TS(IV-V)

```
BP86
SCF  =       -1877.74543084
H(0 K)=      -1877.033580
H(298 K)=    -1876.982872
G(298 K)=    -1877.125665
SCF(C6H6) =        -1877.75345194
SCF(D3BJ) =        -1877.98553229
SCF(BS2) =         -3885.52750440
Low Freq. = -32.7417cm-1, 5.9158cm-1
B97D
SCF(C6H6,BS2)=  -3884.70132141
```

89

^{3}TS(IV-V)

```
C  -3.79024  0.16458  3.88007
C  -2.54024  0.59246  4.36367
C  -1.38928  0.46329  3.57157
C  -1.45066 -0.10796  2.27398
C  -2.71699 -0.54493  1.80911
C  -3.86977 -0.40554  2.59675
P  -0.01409 -0.21436  1.09465
C   1.43884 -0.46633  2.22155
C   1.35262 -1.18127  3.44546
C   2.49368 -1.43914  4.21759
C   3.76089 -1.00834  3.78722
C   3.86968 -0.32532  2.56445
C   2.72840 -0.06166  1.79329
Ni -0.06346  1.32814 -0.52663
C  -1.95096  1.65404 -0.79299
N  -2.77786  1.25277 -1.82462
C  -4.08878  1.72408 -1.67419
C  -4.10145  2.45088 -0.50476
N  -2.79819  2.39969  0.00132
C  -2.32957  0.44424 -2.95086
C  -2.37990  3.04135  1.24139
C  -5.17762  1.42654 -2.65465
C  -5.21145  3.17187  0.19010
C   1.65845  2.10923 -0.83730
N   2.42171  2.09140 -1.99190
C   3.54249  2.93003 -1.90627
C   3.50656  3.49116 -0.64948
N   2.36281  2.97870 -0.02354
C   2.05441  1.33168 -3.18190
C   1.94860  3.31311  1.33344
C   4.52323  3.09630 -3.02278
C   4.43818  4.44913  0.02153
H   1.31088  0.57401 -2.88853
H   2.93626  0.81690 -3.59666
H   1.62540  1.98980 -3.95879
H   1.10606  2.65101  1.58626
H   1.62801  4.36761  1.40115
H   2.76976  3.13378  2.04655
H   5.02960  2.14722 -3.27856
H   5.30406  3.81946 -2.74001
H   4.04327  3.46997 -3.94568
H   3.92560  5.37828  0.33115
H   5.25326  4.73280 -0.66232
H   4.90112  4.01436  0.92644
H  -5.38658  2.76811  1.20378
H  -6.14977  3.06927 -0.37704
H  -5.00492  4.25269  0.29754
H  -4.94222  1.80506 -3.66645
H  -6.11866  1.90110 -2.33545
H  -5.36788  0.34153 -2.74746
H  -2.38373  1.01587 -3.89436
H  -2.94063 -0.46855 -3.04285
H  -1.28739  0.14658 -2.75851
H  -1.29701  2.87560  1.33821
H  -2.89038  2.59153  2.10879
H  -2.58846  4.12472  1.20558
H   0.37940 -1.54292  3.79239
H   2.39280 -1.99022  5.15985
H   4.65130 -1.21282  4.39126
H   4.85257  0.00290  2.20607
H   2.82532  0.46357  0.83781
H  -0.42689  0.81170  3.96131
H  -2.78777 -1.00708  0.81840
H  -2.46180  1.03671  5.36301
H  -4.83335 -0.75965  2.21237
H  -4.68771  0.26377  4.50028
P   0.35806 -1.91869 -1.75689
C  -1.08520 -3.03283 -1.41164
C   1.80485 -2.89023 -1.16390
C   1.79645 -4.27658 -0.86101
C   2.97889 -4.94514 -0.51671
C   4.20273 -4.25531 -0.46467
C   4.23035 -2.88158 -0.76256
C   3.05097 -2.21001 -1.10595
H   0.85570 -4.83345 -0.90807
H   2.94510 -6.01717 -0.29073
H   5.12395 -4.78167 -0.19345
H   5.17613 -2.32976 -0.71840
H   3.08259 -1.13633 -1.32552
C  -2.01759 -3.26637 -2.45253
C  -3.17167 -4.03662 -2.23634
C  -3.41999 -4.59404 -0.97093
C  -2.50641 -4.37176  0.07461
C  -1.35623 -3.59681 -0.13919
H  -1.81790 -2.85590 -3.44981
H  -3.87138 -4.21285 -3.06143
H  -4.31758 -5.19822 -0.80027
H  -2.69533 -4.79640  1.06695
H  -0.66005 -3.41009  0.68471
```

^{3}V
BP86
SCF = -1877.77239518
H(0 K)= -1877.059321
H(298 K)= -1877.008379
G(298 K)= -1877.149674
SCF(C6H6) = -1877.78040846
SCF(D3BJ) = -1878.01969130
SCF(BS2) = -3885.55521167
Low Freq. = 12.3042cm-1, 15.3514cm-1
B97D
SCF(C6H6,BS2)= -3884.72788476

89
^{3}V

```
C   5.07498  1.57096  2.68461
C   3.96805  1.30121  3.51024
C   2.66895  1.31058  2.98155
C   2.43553  1.59729  1.61186
C   3.56185  1.87885  0.79900
C   4.86365  1.86116  1.32519
P   0.76407  1.50839  0.80598
C  -0.35750  2.32433  2.03060
C   0.08052  3.30192  2.96157
C  -0.83065  3.95573  3.80263
C  -2.20453  3.66553  3.73164
C  -2.65641  2.71254  2.80281
C  -1.74806  2.04901  1.96619
Ni  0.25172 -0.30525 -0.42054
C   2.03031 -0.53184 -1.32350
N   2.40370 -0.08282 -2.57351
C   3.76883 -0.28100 -2.82680
C   4.28078 -0.89040 -1.70312
N   3.20648 -1.03876 -0.81589
C   1.48750  0.56786 -3.50695
C   3.33413 -1.61201  0.52094
C   4.42868  0.12632 -4.10563
C   5.67006 -1.33316 -1.37282
C  -0.15472 -2.06087  0.40354
N  -0.07525 -3.30136 -0.20203
C  -0.50799 -4.33689  0.64042
C  -0.86606 -3.73838  1.82594
N  -0.63632 -2.36558  1.65781
C   0.32690 -3.50749 -1.58906
C  -0.94826 -1.36491  2.67350
C  -0.52438 -5.77277  0.22287
C  -1.40207 -4.32178  3.09396
H   0.69503 -2.54575 -1.97265
H  -0.53041 -3.83613 -2.20225
H   1.12526 -4.26624 -1.65309
H  -0.51459 -0.40990  2.34399
H  -0.51401 -1.65645  3.64404
H  -2.03927 -1.24828  2.78200
H  -1.15423 -5.93953 -0.66997
H  -0.92739 -6.40039  1.03290
H   0.48694 -6.15111 -0.01610
H  -0.73082 -4.14053  3.95351
H  -1.52193 -5.41177  2.99279
H  -2.38950 -3.89999  3.35399
H   6.05192 -0.82779 -0.46733
H   6.35540 -1.09753 -2.20189
H   5.73038 -2.42215 -1.19163
H   4.01251 -0.40828 -4.97946
H   5.50653 -0.09596 -4.06653
H   4.31888  1.20814 -4.30234
H   1.59819  0.13522 -4.51489
H   1.67715  1.65335 -3.55643
H   0.46094  0.40016 -3.14746
H   2.32991 -1.62481  0.96656
H   4.00012 -0.99258  1.14402
H   3.73178 -2.63992  0.46349
H   1.14355  3.55662  3.01990
H  -0.46460  4.70512  4.51406
H  -2.91379  4.17963  4.38909
H  -3.72429  2.47717  2.73091
H  -2.10824  1.30506  1.24585
H   1.81795  1.09585  3.63696
H   3.40584  2.11991 -0.25860
H   4.11903  1.07809  4.57282
H   5.71499  2.09518  0.67531
H   6.08864  1.56641  3.09949
P  -1.74713  0.04616 -1.40921
C  -2.25496  1.68409 -2.11760
C  -3.32505 -0.64333 -0.73155
C  -4.39614  0.16085 -0.26091
C  -5.54508 -0.41743  0.29828
C  -5.65955 -1.81374  0.41448
C  -4.60689 -2.62595 -0.04232
C  -3.46180 -2.05031 -0.61069
H  -4.32383  1.25051 -0.33769
H  -6.35636  0.22999  0.65096
H  -6.55887 -2.26263  0.84942
H  -4.68482 -3.71707  0.03019
H  -2.65837 -2.69686 -0.97974
C  -3.44563  1.83911 -2.87357
C  -3.74773  3.05426 -3.50350
C  -2.86502  4.14512 -3.40712
C  -1.67849  4.00473 -2.66899
C  -1.37562  2.79094 -2.03224
H  -4.13737  0.99522 -2.96900
H  -4.67528  3.14818 -4.08009
H  -3.10206  5.09353 -3.90129
H  -0.98690  4.85009 -2.57538
H  -0.45521  2.69613 -1.44215
```

IMe_4
BP86
SCF = -383.432446265
H(0 K)= -383.255345
H(298 K)= -383.243955
G(298 K)= -383.290250
SCF(C6H6) = -383.436077558
SCF(D3BJ) = -383.462870540
SCF(BS2) = -383.569570682
Low Freq. = 121.9201cm-1, 123.208cm-1
B97D
SCF(C6H6,BS2)= -383.303344820

(viii) IMe_4 Dissociation

21
IMe_4

N -1.06521 -0.71080 -0.00009
N   1.06525 -0.71076 0.00013
C   0.00004 -1.59182 -0.00028
C -0.68851 0.64431 -0.00007
C   0.68851 0.64434 -0.00038
C   1.66774 1.77568 0.00001
H   2.32435 1.75937 0.88991
H   1.13992 2.74309 -0.00058
H   2.32574 1.75911 -0.88884
C -1.66782 1.77561 0.00010
H -2.32490 1.75950 -0.88945
H -1.14006 2.74305 0.00062
H -2.32532 1.75871 0.88932
C -2.44348 -1.18041 0.00009
H -2.98838 -0.83528 -0.89675
H -2.98850 -0.83414 0.89644
H -2.40342 -2.27852 0.00089
C   2.44350 -1.18040 0.00015
H   2.40343 -2.27850 0.00076
H   2.98869 -0.83420 0.89643
H   2.98832 -0.83515 -0.89671

Ni(IMe$_4$)(H)(PPh$_2$)
BP86
SCF =   -1025.01552529
H(0 K)=   -1024.652756
H(298 K)=   -1024.626066
G(298 K)=   -1024.712979
SCF(C6H6) =   -1025.02160654
SCF(D3BJ) =   -1025.11662018
SCF(BS2) =   -2697.68724631
Low Freq. = 11.2491cm-1, 13.1651cm-1
B97D
SCF(C6H6,BS2)= -2697.46834466

46
Ni(IMe$_4$)(H)(PPh$_2$)
Ni -0.59057 -0.08539 -0.31317
P   1.48899 -0.08533 -0.74597
N -3.24747 0.99038 0.35494
N -3.38161 -1.05550 -0.32715
C -2.46125 -0.05209 -0.09481
C -4.60360 0.64874 0.40790
C -4.68973 -0.65725 -0.02584
C -2.69685 2.28502 0.74018
H -1.60119 2.20863 0.65328
H -2.96420 2.52992 1.78214
H -3.06267 3.08590 0.07490
C -3.00602 -2.37523 -0.82117
H -1.91363 -2.36897 -0.96432
H -3.49953 -2.59210 -1.78397
H -3.27316 -3.16064 -0.09352
C -5.66338 1.60028 0.86295
H -5.50694 1.92777 1.90710
H -6.65444 1.12337 0.81146
H -5.69965 2.51018 0.23639
C -5.87079 -1.56041 -0.18403
H -5.99740 -1.89987 -1.22843
H -6.79465 -1.03706 0.10733
H -5.78934 -2.46466 0.44664
C   2.56690 -1.45813 -0.09657
C   2.02182 -2.52072 0.65948
H   0.97352 -2.46773 0.97492
C   2.81100 -3.62508 1.01668
H   2.37134 -4.43587 1.60855
C   4.16089 -3.68642 0.63323
H   4.77746 -4.54579 0.91741
C   4.71477 -2.63483 -0.11663
H   5.76626 -2.67246 -0.42240
C   3.92458 -1.53585 -0.48564
H   4.36510 -0.72811 -1.08082
C   2.42152 1.46522 -0.28933
C   3.38767 1.54769 0.73872
C   2.12708 2.63022 -1.03523
C   4.03574 2.76201 1.01198
C   2.76858 3.84587 -0.75322
C   3.72833 3.91568 0.27043
H   3.63114 0.65643 1.32592
H   1.39049 2.57374 -1.84558
H   4.78090 2.80874 1.81420
H   2.52703 4.73753 -1.34248
H   4.23664 4.86144 0.48659
H   0.47789 -0.09203 0.74399

cis-[Ni(IMe$_4$)(PPh$_2$)$_2$]
BP86
SCF =   -1494.31262686
H(0 K)=   -1493.777883
H(298 K)=   -1493.738974
G(298 K)=   -1493.855058
SCF(C6H6) =   -1494.31915263
SCF(D3BJ) =   -1494.49472842
SCF(BS2) =   -3501.97098611
Low Freq. = 7.3828cm-1, 13.2342cm-1
B97D
SCF(C6H6,BS2)= -3501.38813762

68
cis-[Ni(IMe$_4$)(PPh$_2$)$_2$]
C   4.13068 -0.20303 1.54243
C   2.91814 -0.93476 1.55597
C   2.42891 -1.40420 2.80003
C   3.12643 -1.15116 3.98952
C   4.33180 -0.42889 3.96122
C   4.82881 0.04313 2.73410
P   1.87935 -1.25648 0.06152
C   3.05828 -1.26206 -1.35772
C   2.61580 -0.75401 -2.60281
C   3.43984 -0.82483 -3.73542
C   4.71548 -1.40794 -3.64970
C   5.16235 -1.92221 -2.42018
C   4.34260 -1.85445 -1.28439
Ni -0.17255 -0.79018 -0.06893
C -1.92970 -1.42097 0.23798
N -2.80744 -1.09218 1.24336
C -4.00454 -1.81299 1.15237
C -3.87906 -2.63407 0.05272
N -2.61005 -2.37623 -0.48333
C -2.51657 -0.08853 2.26217
C -2.05241 -3.03032 -1.66231
P -0.54111 1.11248 -1.09314
C -2.27438 1.75291 -0.79367

C	-2.61125	2.80724	0.08888
C	-3.94282	3.22940	0.23531
C	-4.97162	2.60567	-0.49111
C	-4.65409	1.55895	-1.37516
C	-3.32325	1.14531	-1.52738
C	0.45746	2.54576	-0.42224
C	1.38698	2.42903	0.63448
C	2.19213	3.51801	1.01128
C	2.07651	4.74875	0.34769
C	1.14951	4.88398	-0.70093
C	0.35332	3.79516	-1.08380
C	-4.81968	-3.63556	-0.53652
C	-5.13011	-1.63454	2.11955
H	-1.03950	-2.62594	-1.81459
H	-2.66554	-2.82261	-2.55581
H	-1.98871	-4.12137	-1.51292
H	-2.58614	-0.52792	3.27156
H	-3.20973	0.76409	2.17768
H	-1.48957	0.26607	2.08640
H	-1.82024	3.30394	0.65990
H	-4.17736	4.05134	0.92173
H	-6.00889	2.93924	-0.37879
H	-5.44524	1.07506	-1.95929
H	-3.08056	0.34220	-2.23373
H	1.48085	1.47414	1.15962
H	2.90784	3.39844	1.83218
H	2.70207	5.59793	0.64338
H	1.05206	5.83908	-1.22930
H	-0.35544	3.91010	-1.91202
H	1.62363	-0.29107	-2.66917
H	3.08584	-0.41534	-4.68801
H	5.35795	-1.46212	-4.53501
H	6.15340	-2.38355	-2.34570
H	4.69775	-2.26664	-0.33399
H	1.49587	-1.97968	2.82095
H	2.73380	-1.52768	4.94056
H	4.88093	-0.23587	4.88886
H	5.76435	0.61279	2.70435
H	4.52122	0.18030	0.59465
H	-5.96560	-2.30402	1.86280
H	-4.82672	-1.86505	3.15718
H	-5.51653	-0.59941	2.11049
H	-5.75533	-3.66728	0.04287
H	-5.08501	-3.39183	-1.58146
H	-4.39451	-4.65605	-0.53562

2. Ni(iIPr)2

(i) 1st P-H Activation

Ni(iIPr)2, A

BP86
SCF = -1095.27360147
H(0 K)= -1094.805673
H(298 K)= -1094.776142
G(298 K)= -1094.868077
SCF(C6H6) = -1095.27845500
SCF(D3BJ) = -1095.37729712
SCF(BS2) = -2433.13235065
Low Freq. = 11.4144cm-1, 24.1949cm-1
B97D
SCF(C6H6,BS2)= -2432.92816019

55
Ni(iIPr)2

```
Ni  0.00000 -0.00008  0.00003
C  -1.85389 -0.00000 -0.00000
N  -2.72405  0.76778  0.76682
C  -4.05820  0.48473  0.48375
C  -4.05825 -0.48408 -0.48417
N  -2.72413 -0.76750 -0.76700
C  -2.24531  1.75365  1.75279
C  -2.24551 -1.75389 -1.75253
H  -4.88823  0.98169  0.98011
H  -4.88833 -0.98072 -0.98077
C   1.85389  0.00000 -0.00010
N   2.72422 -0.76721  0.76708
C   4.05831 -0.48402  0.48385
C   4.05814  0.48438 -0.48449
N   2.72395  0.76737 -0.76744
C   2.24572 -1.75319  1.75307
C   2.24510  1.75332 -1.75329
H   4.88845 -0.98057  0.98043
H   4.88810  0.98106 -0.98124
H   1.14266 -1.67086  1.67052
C   2.67886 -1.36503  3.17575
C   2.67923 -3.17583  1.36522
H   1.14206  1.67067 -1.67065
C   2.67822  1.36544 -3.17604
C   2.67821  3.17604 -1.36530
C  -2.67745  1.36490  3.17562
C  -2.67956  3.17625  1.36563
H  -1.14227  1.67173  1.66957
H  -1.14245 -1.67161 -1.66979
C  -2.67832 -1.36622 -3.17544
C  -2.67920 -3.17635 -1.36424
H  -2.24499  2.06672  3.90881
H  -3.77584  1.39520  3.29089
H  -2.33102  0.34768  3.42081
H  -2.24689  3.90937  2.06739
H  -2.33484  3.42262  0.34810
H  -3.77801  3.29041  1.39759
H  -2.24671 -3.90983 -2.06574
H  -2.33391 -3.42196 -0.34673
H  -3.77765 -3.29075 -1.39557
H  -2.24586 -2.06836 -3.90832
H  -3.77673 -1.39702 -3.29032
H  -2.33238 -0.34902 -3.42144
H   2.24557  2.06723 -3.90915
H   3.77664  1.39664 -3.29083
H   2.33270  0.34805 -3.42184
H   2.24556  3.90919 -2.06704
H   2.33268  3.42176 -0.34790
H   3.77663  3.29083 -1.39649
H   2.24651 -2.06687  3.90897
H   3.77729 -1.39586  3.29041
H   2.33303 -0.34772  3.42146
H   2.24681 -3.90901  2.06706
H   2.33372 -3.42178  0.34786
H   3.77767 -3.29032  1.39638
```

IiPr
BP86
SCF = -1565.74704448
H(0 K)= -1565.093220
H(298 K)= -1565.050465
G(298 K)= -1565.174253
SCF(C6H6) = -1565.75194446
SCF(D3BJ) = -1565.93689773
SCF(BS2) = -3238.60035520
Low Freq. = 9.2234cm-1, 14.0204cm-1
B97D
SCF(C6H6,BS2)= -3238.03898765

79
IiPr

```
C   1.93145  5.37725 -0.14033
C   0.80417  5.37835 -0.98143
C   0.06436  4.20075 -1.16939
C   0.44622  3.00303 -0.52738
C   1.57822  3.01202  0.31027
C   2.31668  4.19300  0.50720
P  -0.47314  1.36576 -0.76236
C  -2.23353  1.97095 -0.50625
C  -3.26550  1.55371 -1.37637
C  -4.60130  1.91125 -1.13355
C  -4.93306  2.68857 -0.00991
C  -3.91574  3.10418  0.86631
C  -2.58002  2.74862  0.62163
Ni  0.22950 -0.54748 -0.05747
C   2.04002 -0.66602  0.49416
N   3.20966 -0.57289 -0.25993
C   4.35637 -0.69824  0.52566
C   3.93400 -0.86874  1.81433
N   2.53862 -0.85186  1.78314
C   3.20481 -0.36880 -1.71776
C   1.65954 -1.00314  2.95301
H   5.36254 -0.65880  0.11538
H   4.50576 -0.99797  2.73009
C  -1.09181 -1.91082 -0.22249
N  -1.20131 -2.88014 -1.21588
C  -2.31905 -3.69626 -1.03721
C  -2.94548 -3.25794  0.09592
N  -2.20201 -2.17793  0.57302
C  -0.23733 -2.98663 -2.32182
C  -2.50483 -1.42143  1.80055
H  -2.57894 -4.50460 -1.71627
H  -3.84522 -3.62061  0.58677
```

H	0.56412	-2.28219	-2.02266
C	-0.85913	-2.51004	-3.64503
C	0.34483	-4.40625	-2.41805
H	-1.77582	-0.58835	1.77539
C	-2.25167	-2.28600	3.04821
C	-3.92837	-0.84480	1.76747
C	3.75655	-1.60549	-2.44748
C	3.94650	0.92182	-2.10326
H	2.12917	-0.25237	-1.95469
H	0.65020	-1.06486	2.50013
C	1.72916	0.23402	3.86388
C	1.95870	-2.31004	3.70562
H	-3.01795	0.94871	-2.25675
H	-5.38462	1.58900	-1.82910
H	-5.97429	2.97087	0.17845
H	-4.16226	3.71586	1.74171
H	-1.79370	3.09539	1.30201
H	-0.82201	4.21211	-1.81394
H	1.87599	2.07332	0.79348
H	0.50044	6.30072	-1.48946
H	3.19297	4.18642	1.16536
H	2.50622	6.29810	0.00785
H	-0.53619	1.42312	-2.21357
H	3.67194	-1.47648	-3.54000
H	4.82340	-1.76765	-2.21117
H	3.20017	-2.51134	-2.15620
H	3.85820	1.09918	-3.18849
H	3.52202	1.78936	-1.57326
H	5.02299	0.85731	-1.86347
H	1.23065	-2.45265	4.52197
H	1.89543	-3.17518	3.02568
H	2.96561	-2.29902	4.15970
H	1.01571	0.13687	4.70006
H	2.73855	0.35841	4.29549
H	1.47904	1.14604	3.29809
H	-2.39734	-1.68980	3.96519
H	-2.95011	-3.14092	3.08880
H	-1.22376	-2.68395	3.04639
H	-4.09699	-0.21285	2.65525
H	-4.08232	-0.22545	0.87018
H	-4.69020	-1.64514	1.78090
H	-0.10795	-2.52885	-4.45325
H	-1.69949	-3.15900	-3.94995
H	-1.23663	-1.47970	-3.54338
H	1.13374	-4.44001	-3.18832
H	0.78329	-4.71529	-1.45524
H	-0.42436	-5.14637	-2.70211

TS(I-II)iPr

BP86
SCF = -1565.74021817
H(0 K)= -1565.087898
H(298 K)= -1565.045528
G(298 K)= -1565.168844
SCF(C6H6) = -1565.74514448
SCF(D3BJ) = -1565.92895657
SCF(BS2) = -3238.59271845
Low Freq. = -206.2713cm-1, 7.6091cm-1
B97D
SCF(C6H6,BS2)= -3238.02841326

79

TS(I-II)iPr

C	0.84547	5.70243	0.07319
C	-0.37647	5.45358	-0.57519
C	-0.91299	4.15654	-0.60447
C	-0.23558	3.08416	0.01642
C	0.98923	3.34672	0.66846
C	1.52875	4.64333	0.69368
P	-0.83899	1.29898	0.03407
C	-2.66922	1.52526	-0.29827
C	-3.31912	0.76422	-1.29595
C	-4.71499	0.80826	-1.44161
C	-5.49127	1.61436	-0.59136
C	-4.85759	2.37420	0.40741
C	-3.46314	2.32556	0.55622
Ni	0.34002	-0.52818	0.03138
C	2.19747	-0.24325	0.25250
N	3.17596	0.20967	-0.63060
C	4.43486	0.27542	-0.03247
C	4.27851	-0.13778	1.26130
N	2.92894	-0.45043	1.42026
C	2.89851	0.55958	-2.03448
C	2.30725	-0.93353	2.66485
H	5.32558	0.60236	-0.56337
H	5.00779	-0.23285	2.06211
C	-0.72295	-2.09700	-0.13220
N	-0.65626	-3.07238	-1.12310
C	-1.57631	-4.09881	-0.91620
C	-2.25259	-3.79141	0.23186
N	-1.73554	-2.58138	0.69082
C	0.29667	-3.00300	-2.24345
C	-2.16578	-1.90201	1.92712
H	-1.67979	-4.94782	-1.58742
H	-3.04695	-4.32770	0.74494
H	0.92761	-2.13226	-1.97351
C	-0.42845	-2.71823	-3.56861
C	1.17151	-4.26545	-2.30516
H	-1.63199	-0.93398	1.88921
C	-1.70651	-2.68870	3.16670
C	-3.68053	-1.64230	1.92255
C	3.63377	-0.39348	-2.99262
C	3.21892	2.03690	-2.31492
H	1.80787	0.40006	-2.12980
H	1.26862	-1.16027	2.34968
C	2.28298	0.16974	3.73519
C	2.98374	-2.22327	3.15674
H	-2.72018	0.13000	-1.95963
H	-5.19737	0.21696	-2.22837
H	-6.57976	1.65113	-0.70625
H	-5.45265	3.00734	1.07537
H	-2.98244	2.92380	1.33931
H	-1.86674	3.97537	-1.11085
H	1.51970	2.51544	1.14773
H	-0.91397	6.27379	-1.06474
H	2.47964	4.82789	1.20636
H	1.26055	6.71588	0.09636
H	-0.45402	0.89435	-1.33855
H	3.35262	-0.17622	-4.03702
H	4.72953	-0.28075	-2.91107
H	3.38043	-1.44348	-2.77249
H	2.92173	2.29947	-3.34432
H	2.67650	2.69474	-1.61747

H   4.30057 2.24038 -2.21833
H   2.44965 -2.61852 4.03734
H   2.97454 -2.99384 2.36886
H   4.03193 -2.04704 3.45786
H   1.75847 -0.18462 4.63891
H   3.30500 0.46509 4.03344
H   1.75847 1.06288 3.35881
H -1.95724 -2.13311 4.08636
H -2.20133 -3.67488 3.22317
H -0.61667 -2.85230 3.14290
H -3.96035 -1.05407 2.81241
H -3.97950 -1.07528 1.02690
H -4.25741 -2.58423 1.95425
H   0.30100 -2.60408 -4.38864
H -1.11317 -3.54185 -3.83930
H -1.01735 -1.78938 -3.49567
H   1.93911 -4.15448 -3.08969
H   1.68018 -4.43459 -1.34217
H   0.57945 -5.16578 -2.54828

IIiPr
BP86
SCF =   -1565.74380972
H(0 K)=   -1565.090982
H(298 K)=   -1565.048211
G(298 K)=   -1565.171871
SCF(C6H6) =   -1565.74892669
SCF(D3BJ) =   -1565.93402987
SCF(BS2) =   -3238.59506424
Low Freq. = 9.5169cm-1, 14.3403cm-1
B97D
SCF(C6H6,BS2)= -3238.03163161

79
IIiPr
C   5.12467 2.37328 0.19101
C   4.22060 3.05193 -0.64527
C   2.84175 2.81130 -0.54310
C   2.33669 1.88434 0.39791
C   3.25699 1.21012 1.23198
C   4.63718 1.44943 1.13086
P   0.51967 1.44628 0.59650
C -0.28655 3.07268 0.11376
C -1.44650 3.07748 -0.69351
C -2.14565 4.26926 -0.94488
C -1.70154 5.48215 -0.39162
C -0.55302 5.48998 0.41838
C   0.14418 4.29855 0.67190
Ni -0.26032 -0.51245 0.00268
C   1.06471 -1.88533 0.15657
N   2.11972 -2.22662 -0.68121
C   2.87422 -3.28289 -0.17157
C   2.30790 -3.63054 1.02340
N   1.21398 -2.78540 1.20545
C   2.37291 -1.56557 -1.97521
C   0.31534 -2.79385 2.37270
H   3.73734 -3.69614 -0.68739
H   2.59310 -4.39607 1.74059
C -2.14566 -0.63765 -0.27633
N -2.80412 -1.05182 -1.42903
C -4.19121 -1.01160 -1.28778
C -4.44138 -0.56842 -0.01902
N -3.20186 -0.34279 0.57971
C -2.08588 -1.47527 -2.64326
C -3.01498 0.14535 1.95903
H -4.87702 -1.29334 -2.08292
H -5.38502 -0.39818 0.49331
H -1.02628 -1.46660 -2.31941
C -2.27727 -0.45915 -3.78100
C -2.48278 -2.90375 -3.04966
H -1.91850 0.25656 2.05124
C -3.51316 -0.89408 2.97760
C -3.67083 1.52200 2.15414
C   2.06304 -2.51945 -3.14197
C   3.79925 -0.99813 -2.04291
H   1.64830 -0.73030 -1.98458
H -0.51569 -2.13441 2.05249
C   1.00126 -2.17589 3.60183
C -0.23116 -4.20473 2.64305
H -1.79764 2.13000 -1.11945
H -3.03720 4.25121 -1.58251
H -2.24473 6.41243 -0.58948
H -0.19750 6.42935 0.85711
H   1.03846 4.32102 1.30499
H   2.14695 3.35214 -1.19440
H   2.87889 0.48723 1.96416
H   4.59223 3.77258 -1.38287
H   5.33187 0.91941 1.79232
H   6.20008 2.56580 0.11224
H   0.29756 0.77299 -0.82504
H   2.17458 -1.99359 -4.10538
H   2.75244 -3.38253 -3.14825
H   1.03271 -2.90465 -3.07010
H   3.93124 -0.43100 -2.97951
H   3.99442 -0.32060 -1.19694
H   4.55712 -1.80217 -2.03365
H -0.98104 -4.16796 3.45101
H -0.71071 -4.62013 1.74177
H   0.56421 -4.90007 2.96550
H   0.30229 -2.14123 4.45495
H   1.88195 -2.76760 3.90918
H   1.32933 -1.14725 3.38081
H -3.29042 -0.55821 4.00445
H -4.60547 -1.04085 2.90261
H -3.02540 -1.86924 2.81480
H -3.44619 1.90484 3.16381
H -3.28695 2.24523 1.41728
H -4.76986 1.46655 2.05527
H -1.68116 -0.75335 -4.66157
H -3.33470 -0.40015 -4.09539
H -1.95377 0.54522 -3.46311
H -1.87127 -3.23742 -3.90501
H -2.32560 -3.60507 -2.21412
H -3.54186 -2.96282 -3.35793

TS(II-cis-III)iPr
BP86
SCF =   -1565.74225689
H(0 K)=   -1565.089771
H(298 K)=   -1565.047686
G(298 K)=   -1565.167805
SCF(C6H6) =   -1565.74811445
SCF(D3BJ) =   -1565.93368847
SCF(BS2) =   -3238.59224878

Low Freq. = -119.0619cm-1, 8.8617cm-1
B97D
SCF(C6H6,BS2)= -3238.03051936

79
TS(II-cis-III)iPr

```
N  -3.14062  1.07051  0.37346
C  -2.29694  0.30258 -0.41771
N  -3.05928  0.09087 -1.55588
C  -4.30923  0.70699 -1.47367
C  -4.36110  1.32073 -0.25372
Ni -0.48362 -0.17827 -0.03794
C  -0.00005 -1.96808  0.48046
N   0.62567 -2.94160 -0.28338
C   0.88404 -4.09741  0.45269
C   0.42464 -3.86723  1.71938
N  -0.11240 -2.57886  1.71967
C   0.92200 -2.77390 -1.71980
C   2.40592 -3.02658 -2.02445
H   1.36803 -4.97264  0.02705
H   0.43800 -4.50415  2.60010
C  -0.70801 -1.91066  2.89169
C  -1.81751 -2.77453  3.51321
C  -2.54968 -0.63100 -2.73463
C  -3.51126 -1.75348 -3.15608
H  -5.04905  0.65553 -2.26838
H  -5.15620  1.89904  0.21006
C  -2.76043  1.58420  1.70396
C  -2.73115  3.12099  1.71750
P   1.23126  1.05001  0.58874
C   1.42217  2.86125  0.09943
C   0.45758  3.56198 -0.65848
C   0.55749  4.94890 -0.86262
C   1.63316  5.66898 -0.31937
C   2.60487  4.98685  0.43440
C   2.49513  3.60525  0.64735
C   2.90992  0.40156  0.06588
C   3.66410  0.91858 -1.01409
C   4.92499  0.38977 -1.33161
C   5.46534 -0.66656 -0.57786
C   4.72661 -1.19328  0.49661
C   3.46458 -0.66633  0.81122
C  -0.00541 -3.66323 -2.56647
C   0.37341 -1.50674  3.90640
C  -2.23738  0.34531 -3.88122
C  -3.67267  1.00086  2.79580
H  -1.60277 -1.07175 -2.37155
H  -1.72763  1.21023  1.84176
H   0.68990 -1.70735 -1.90625
H  -1.15438 -0.99456  2.46293
H  -0.37950  3.00600 -1.09893
H  -0.20273  5.46530 -1.46054
H   1.71581  6.74905 -0.48166
H   3.45075  5.53584  0.86394
H   3.25548  3.09002  1.24597
H   3.26164  1.75099 -1.60070
H   2.89656 -1.08136  1.65186
H   5.49050  0.80743 -2.17278
H   5.13873 -2.01194  1.09786
H   6.45282 -1.07252 -0.82235
H   0.09304  1.02227 -0.75953
H  -2.38845  3.48047  2.70217
H  -2.03723  3.50595  0.95362
H  -3.73348  3.54775  1.53432
H  -3.32409  1.32099  3.79207
H  -4.71483  1.34839  2.68017
H  -3.67383 -0.10097  2.76226
H  -1.79245 -0.19478 -4.73416
H  -3.15297  0.84831 -4.23972
H  -1.52431  1.11569 -3.54664
H  -3.06663 -2.34297 -3.97515
H  -3.72155 -2.43112 -2.31245
H  -4.47214 -1.35212 -3.52419
H   0.15541 -3.47012 -3.64076
H   0.19640 -4.73394 -2.38484
H  -1.06403 -3.47013 -2.32854
H   2.60972 -2.80325 -3.08508
H   3.05277 -2.38871 -1.40313
H   2.68007 -4.08314 -1.85186
H  -2.57111 -3.05298  2.75840
H  -1.41474 -3.70161  3.95827
H  -0.08451 -0.98861  4.76627
H   0.91264 -2.39036  4.29153
H   1.09905 -0.82197  3.43797
H  -2.32065 -2.21564  4.31987
```

cis-IIIiPr
BP86
SCF =   -1565.74799692
H(0 K)=   -1565.093488
H(298 K)=   -1565.051275
G(298 K)=   -1565.170696
SCF(C6H6) =   -1565.75506751
SCF(D3BJ) =   -1565.94359765
SCF(BS2) =   -3238.59542518
Low Freq. = 15.4313cm-1, 19.9616cm-1
B97D
SCF(C6H6,BS2)= -3238.03900241

79
cis-IIIiPr

```
N  -2.78180  1.88982  0.71001
C  -2.22318  0.97059 -0.16233
N  -3.23735  0.74620 -1.07537
C  -4.36619  1.51941 -0.79481
C  -4.07771  2.23478  0.33390
Ni -0.41868  0.37453 -0.06647
C  -0.52974 -1.54144  0.01362
N   0.04910 -2.46180 -0.84466
C  -0.31296 -3.76703 -0.51853
C  -1.12991 -3.69104  0.57486
N  -1.25346 -2.33706  0.88487
C   0.96102 -2.09749 -1.95608
C   2.18677 -3.02176 -1.99080
H   0.04371 -4.63133 -1.07161
H  -1.61281 -4.47637  1.15049
C  -2.02182 -1.80634  2.02871
C  -3.51563 -2.14112  1.88851
C  -3.07216 -0.10319 -2.27044
C  -4.28372 -1.02389 -2.47943
H  -5.26907  1.48807 -1.39854
H  -4.68633  2.93840  0.89575
```

```
C  -2.03396  2.50599  1.83002
C  -1.67724  3.96291  1.49751
P   1.58851  0.77048  0.75185
C   2.59211  2.12918 -0.05226
C   2.24382  2.74360 -1.27719
C   2.97211  3.83805 -1.77609
C   4.07974  4.33331 -1.07103
C   4.45107  3.72649  0.14217
C   3.71292  2.64559  0.64539
C   2.83905 -0.62325  0.75030
C   4.07272 -0.61063  0.05385
C   4.99178 -1.66509  0.18210
C   4.70282 -2.77161  0.99817
C   3.47668 -2.80850  1.68816
C   2.56650 -1.74916  1.56816
C   0.21813 -2.09166 -3.30248
C  -1.43004 -2.29121  3.36213
C  -2.76655  0.76302 -3.50441
C  -2.80480  2.36690  3.15137
H  -2.18550 -0.71539 -2.03102
H  -1.09803  1.91770  1.88016
H   1.27881 -1.06856 -1.70521
H  -1.88478 -0.71391  1.94565
H   1.38673  2.35312 -1.83551
H   2.67685  4.29753 -2.72679
H   4.65056  5.18293 -1.46166
H   5.31199  4.10545  0.70493
H   4.00014  2.19354  1.60189
H   4.31965  0.24149 -0.58736
H   1.62418 -1.77968  2.12809
H   5.94081 -1.62187 -0.36550
H   3.23575 -3.66075  2.33454
H   5.42240 -3.59113  1.09976
H  -0.14304  1.79494 -0.38295
H  -1.06220  4.39276  2.30545
H  -1.09712  4.00896  0.56232
H  -2.58260  4.58636  1.38748
H  -2.18467  2.74103  3.98243
H  -3.74126  2.95306  3.14722
H  -3.05898  1.31420  3.35843
H  -2.57800  0.12559 -4.38505
H  -3.61515  1.42843 -3.74212
H  -1.87475  1.38424 -3.32307
H  -4.07956 -1.72022 -3.30942
H  -4.49550 -1.61874 -1.57596
H  -5.19367 -0.45719 -2.74445
H   0.91276 -1.80835 -4.11123
H  -0.18820 -3.09140 -3.53885
H  -0.61082 -1.36699 -3.29902
H   2.91626 -2.62705 -2.71639
H   2.67763 -3.07494 -1.00751
H   1.92040 -4.04355 -2.31842
H  -3.91606 -1.74690  0.94064
H  -3.69397 -3.23051  1.91769
H  -1.97724 -1.83704  4.20526
H  -1.50579 -3.38803  3.46563
H  -0.36924 -2.00618  3.44694
H  -4.08408 -1.68841  2.71821
```

TS(II-trans-III)iPr

```
BP86
SCF  =       -1565.72458322
H(0 K)=      -1565.071973
H(298 K)=    -1565.029814
G(298 K)=    -1565.150033
SCF(C6H6) =         -1565.73015536
SCF(D3BJ) =         -1565.91409732
SCF(BS2) =          -3238.57294005
Low Freq. = -144.9638cm-1,
13.7197cm-1
B97D
SCF(C6H6,BS2)=  -3238.00863676
```

79

TS(II-trans-III)iPr

```
C   5.35048 -1.89612  0.67614
C   5.38958 -0.78288 -0.18204
C   4.24956  0.01175 -0.37006
C   3.03424 -0.28181  0.30025
C   3.01295 -1.40989  1.15998
C   4.15266 -2.20659  1.34422
P   1.44895  0.64015  0.03084
C   2.03510  2.36184 -0.34582
C   1.28532  3.13294 -1.26862
C   1.61316  4.47223 -1.52930
C   2.70144  5.07569 -0.87496
C   3.45504  4.32523  0.04444
C   3.12626  2.98702  0.30789
Ni -0.53012 -0.15804 -0.20226
C  -0.51631 -2.09109  0.06173
N   0.06319 -3.08720 -0.70431
C  -0.17245 -4.35437 -0.17441
C  -0.90858 -4.17591  0.96425
N  -1.11532 -2.80274  1.08990
C   0.80569 -2.82678 -1.95782
C  -1.84576 -2.15322  2.19480
H   0.19988 -5.26534 -0.63542
H  -1.29031 -4.90191  1.67746
C  -2.01304  1.05750 -0.10661
N  -3.16290  1.07906 -0.89006
C  -4.02292  2.11273 -0.52530
C  -3.43366  2.76449  0.52062
N  -2.21942  2.12627  0.76254
C  -3.43258  0.13213 -1.99006
C  -1.29987  2.51395  1.85349
H  -4.96856  2.29892 -1.02734
H  -3.77471  3.61837  1.10015
H  -2.68259 -0.66377 -1.83489
C  -3.17945  0.79854 -3.35217
C  -4.84287 -0.46771 -1.87513
H  -0.40539  1.88599  1.68814
C  -1.91312  2.17386  3.22273
C  -0.89980  3.99311  1.74234
C  -0.06874 -3.16527 -3.17628
C   2.15174 -3.56598 -1.97161
H   0.98614 -1.73766 -1.93238
H  -1.95386 -1.10752  1.84624
C  -1.01428 -2.17476  3.48785
C  -3.24063 -2.77170  2.37676
H  -0.72262 -0.25687 -1.63321
H   0.44197  2.65741 -1.78270
H   1.02439  5.04433 -2.25577
H   2.96234  6.11916 -1.08199
H   4.30283  4.78682  0.56389
```

```
H   3.71999  2.41737  1.03087
H   4.29638  0.87026 -1.04808
H   2.08320 -1.65358  1.68660
H   6.31541 -0.53371 -0.71349
H   4.10980 -3.06674  2.02249
H   6.24369 -2.51225  0.82487
H   0.46428 -2.91195 -4.10828
H  -0.31622 -4.24156 -3.20747
H  -1.00933 -2.59150 -3.14849
H   2.72699 -3.26055 -2.86121
H   2.74636 -3.31996 -1.07774
H   2.02280 -4.66229 -2.02499
H  -1.54599 -1.64171  4.29444
H  -0.83117 -3.20895  3.82962
H  -0.04153 -1.68158  3.32968
H  -3.80756 -2.19959  3.13001
H  -3.80606 -2.75556  1.43086
H  -3.18569 -3.81674  2.72989
H  -4.97081 -1.25855 -2.63297
H  -5.00477 -0.91311 -0.87988
H  -5.63249  0.28434 -2.05066
H  -3.33260  0.07271 -4.16900
H  -3.86742  1.64672 -3.51755
H  -2.14406  1.17150 -3.40709
H  -1.19137  2.39864  4.02590
H  -2.82740  2.76328  3.41545
H  -2.17411  1.10445  3.28295
H  -0.14151  4.22800  2.50749
H  -0.46352  4.20908  0.75474
H  -1.75963  4.66700  1.90921
```

trans-IIIiPr
BP86
SCF =   -1565.75457152
H(0 K)=   -1565.100161
H(298 K)=   -1565.058016
G(298 K)=   -1565.177169
SCF(C6H6) =   -1565.76070323
SCF(D3BJ) =   -1565.95383499
SCF(BS2) =   -3238.60037814
Low Freq. = 11.4433cm-1, 17.6532cm-1
B97D
SCF(C6H6,BS2)= -3238.04487349

79
trans-IIIiPr

```
C   3.93948  3.51205 -1.05437
C   3.96042  2.16617 -1.46521
C   2.78247  1.40727 -1.46701
C   1.54163  1.95491 -1.04821
C   1.54189  3.31126 -0.63678
C   2.72020  4.07604 -0.64415
P   0.01807  0.88518 -1.19797
C  -1.31386  2.14743 -0.78433
C  -2.01849  2.76266 -1.84465
C  -3.03727  3.70045 -1.60108
C  -3.36942  4.05278 -0.28351
C  -2.67421  3.46231  0.78559
C  -1.66317  2.51885  0.53510
Ni -0.05602 -0.91848  0.20386
C   1.79625 -1.13796  0.41661
N   2.59740 -2.05511 -0.22989
C   3.90681 -2.01417  0.24923
C   3.93919 -1.04910  1.21754
N   2.65169 -0.52036  1.30166
C   2.08107 -3.00908 -1.23273
C   2.20364  0.50920  2.26408
H   4.69776 -2.64841 -0.14181
H   4.76389 -0.68653  1.82411
C  -1.93797 -1.05652  0.21810
N  -2.79266 -1.40322 -0.80851
C  -4.10289 -1.55213 -0.35209
C  -4.08703 -1.28229  0.98727
N  -2.76773 -0.97651  1.31729
C  -2.35412 -1.68269 -2.19382
C  -2.27188 -0.72853  2.68607
H  -4.92803 -1.80960 -1.01019
H  -4.89605 -1.25920  1.71200
H  -1.31596 -1.30618 -2.22752
C  -3.19298 -0.89163 -3.20790
C  -2.36443 -3.19728 -2.46021
H  -1.24266 -0.35681  2.52495
C  -2.20434 -2.04580  3.47636
C  -3.10806  0.34882  3.39250
C   2.15352 -4.44638 -0.69269
C   2.79371 -2.83740 -2.58256
H   1.01857 -2.71869 -1.33590
H   1.28261  0.91124  1.80173
C   3.23428  1.63693  2.40409
C   1.85524 -0.14531  3.61223
H  -0.12351 -2.23677  0.91680
H  -1.75256  2.50181 -2.87574
H  -3.56769  4.16053 -2.44267
H  -4.16034  4.78561 -0.08975
H  -2.91714  3.74243  1.81764
H  -1.12420  2.06107  1.37210
H   2.81667  0.36749 -1.81198
H   0.60604  3.77753 -0.31334
H   4.89932  1.70758 -1.79727
H   2.68177  5.12430 -0.32446
H   4.85638  4.11110 -1.06247
H   1.69873 -5.14545 -1.41454
H   3.19784 -4.76544 -0.52783
H   1.60779 -4.52517  0.26140
H   2.35467 -3.52084 -3.32863
H   2.68871 -1.80593 -2.95595
H   3.87009 -3.07352 -2.50800
H   3.49574  2.06355  1.42332
H   2.80959  2.44047  3.02805
H   4.15604  1.28996  2.90548
H   1.44232  0.60676  4.30621
H   1.11052 -0.94582  3.47247
H   2.75368 -0.58373  4.08200
H  -1.74035 -3.72544 -1.72075
H  -3.38818 -3.60844 -2.40606
H  -1.96918 -3.40773 -3.46844
H  -3.17678  0.18384 -2.97034
H  -2.77521 -1.02970 -4.21895
H  -4.24328 -1.23339 -3.23365
H  -1.77503 -1.86931  4.47726
H  -3.21051 -2.48102  3.60986
H  -1.57051 -2.77380  2.94477
H  -4.13160 -0.00564  3.60891
```

```
H  -2.64228  0.60935  4.35748
H  -3.17932  1.25918  2.77639
```

^{3}IIIiPr

```
BP86
SCF  =       -1565.71397512
H(0 K)=      -1565.061672
H(298 K)=    -1565.018859
G(298 K)=    -1565.142959
SCF(C6H6) =        -1565.72029686
SCF(D3BJ) =        -1565.90720856
SCF(BS2) =         -3238.55764014
Low Freq. = 8.1703cm-1, 12.8246cm-1
B97D
SCF(C6H6,BS2)=  -3237.99899432
```

79

^{3}IIIiPr

```
N   3.13082 -0.38352 -0.89862
C   2.22295 -0.75517  0.07931
N   3.03244 -1.32077  1.05314
C   4.37370 -1.30299  0.68672
C   4.43679 -0.70772 -0.54290
Ni  0.25850 -0.62709  0.19725
C  -1.43080 -1.59255  0.03306
N  -2.51127 -1.67586  0.88592
C  -3.48446 -2.54269  0.39869
C  -3.02651 -3.02428 -0.79779
N  -1.78198 -2.43615 -1.00724
C  -2.59902 -0.97041  2.18744
C  -3.91831 -0.19744  2.31185
H  -4.41458 -2.73744  0.92573
H  -3.48153 -3.71789 -1.50013
C  -0.90746 -2.69845 -2.16806
C  -0.41623 -4.15524 -2.15943
C   2.51823 -1.92313  2.30290
C   2.52318 -3.45653  2.20289
H   5.16333 -1.70326  1.31690
H   5.29190 -0.48691 -1.17620
C   2.77495  0.25647 -2.18510
C   3.46765  1.62040 -2.32899
P  -0.31660  1.38229 -0.77177
C   0.60816  2.81575 -0.00745
C   1.51714  2.62592  1.05882
C   2.29655  3.69241  1.54053
C   2.17618  4.97215  0.97570
C   1.26787  5.17951 -0.07826
C   0.49945  4.11294 -0.56773
C  -2.07719  1.90774 -0.52219
C  -2.49723  2.93429  0.36205
C  -3.84811  3.30222  0.45537
C  -4.82365  2.65016 -0.31925
C  -4.42839  1.61733 -1.18979
C  -3.07819  1.25631 -1.28985
C  -2.37890 -1.96502  3.33845
C  -1.59876 -2.31125 -3.48417
C   3.28906 -1.40386  3.52514
C   3.07910 -0.69069 -3.35774
H   1.47358 -1.55312  2.34883
H   1.68444  0.42113 -2.12112
H  -1.74584 -0.26875  2.15766
H  -0.04446 -2.02525 -1.99843
H   0.18956 -0.26066  1.71677
H   1.58563  1.62985  1.51052
H   2.99346  3.52390  2.37020
H   2.78197  5.80331  1.35330
H   1.16396  6.17470 -0.52584
H  -0.19490  4.28153 -1.39912
H  -1.75306  3.45228  0.97493
H  -2.78153  0.46235 -1.98540
H  -4.14180  4.10384  1.14326
H  -5.17564  1.09964 -1.80248
H  -5.87669  2.94254 -0.24733
H   2.07460 -3.89703  3.10949
H   1.93680 -3.78946  1.33096
H   3.54945 -3.85449  2.10511
H   2.81358 -1.78046  4.44604
H   4.34021 -1.74402  3.53596
H   3.27783 -0.30252  3.55926
H   3.11479  2.11812 -3.24736
H   3.22872  2.27047 -1.47293
H   4.56526  1.51813 -2.40423
H   2.73000 -0.24090 -4.30215
H   4.16232 -0.88270 -3.45751
H   2.56980 -1.65963 -3.22536
H  -2.38681 -1.43011  4.30310
H  -3.17231 -2.73337  3.37018
H  -1.40510 -2.46999  3.23088
H  -3.90121  0.40220  3.23713
H  -4.06233  0.48653  1.46129
H  -4.79049 -0.87374  2.37643
H   0.29790 -4.32004 -2.98369
H   0.08915 -4.38993 -1.20861
H  -1.25219 -4.86440 -2.29252
H  -0.90415 -2.45349 -4.32881
H  -2.48936 -2.93523 -3.67632
H  -1.90976 -1.25438 -3.46848
```

(ii)2nd P-H Activation

IViPr

```
BP86
SCF  =          -2036.18605906
H(0 K)=         -2035.347051
H(298 K)=       -2035.291509
G(298 K)=       -2035.441390
SCF(C6H6) =     -2036.19247834
SCF(D3BJ) =     -2036.47469560
SCF(BS2) =      -4044.02305280
Low Freq. = 13.7706cm-1, 16.4647cm-
1
B97D
SCF(C6H6,BS2)=  -4043.11631093
```

103

IViPr

```
C   4.84927 -0.16200  0.94486
C   3.63495 -0.02106  0.23276
C   3.66648 -0.15828 -1.17202
C   4.87108 -0.42751 -1.84458
C   6.07001 -0.55427 -1.12475
C   6.05534 -0.41558  0.27418
P   2.05897  0.24266  1.21250
C   2.21943  2.04250  1.68009
```

```
C   3.21223  2.91258  1.17365
C   3.29478  4.24119  1.61664
C   2.38223  4.73501  2.56404
C   1.38397  3.88484  3.06623
C   1.30604  2.55223  2.63385
Ni -0.11725 -0.44233  0.03496
C   0.46085 -1.67194 -1.37662
N   0.25806 -1.65748 -2.75369
C   0.75891 -2.80385 -3.36938
C   1.30121 -3.57533 -2.38158
N   1.11972 -2.88200 -1.18647
C  -0.34277 -0.53881 -3.51023
H   0.69453 -2.97423 -4.44105
H   1.79783 -4.54064 -2.43428
C   1.60157 -3.38204  0.12242
C  -1.26041 -1.55749  1.22728
N  -2.35290 -2.30527  0.80068
C  -2.92776 -3.03787  1.83716
C  -2.20519 -2.75782  2.96123
N  -1.20548 -1.86369  2.58003
H  -3.79318 -3.67992  1.69640
C  -2.87477 -2.32187 -0.58441
C  -0.24859 -1.27258  3.53823
H  -2.32142 -3.11248  3.98207
P  -1.25585  1.62942  0.30282
C  -0.89753  2.98374 -0.94783
C  -1.64391  4.18570 -0.95282
C  -1.28685  5.25220 -1.79055
C  -0.16376  5.15251 -2.63068
C   0.59765  3.97331 -2.62131
C   0.23299  2.90135 -1.79019
C  -3.12642  1.53965  0.19692
C  -3.87515  1.77529 -0.98016
C  -5.27847  1.75497 -0.95889
C  -5.96696  1.48723  0.23749
C  -5.23811  1.24025  1.41315
C  -3.83390  1.27173  1.39099
H  -0.62474  0.19032 -2.73108
C  -1.60414 -0.99002 -4.26394
C   0.69191  0.10709 -4.44643
C   3.10077 -3.71726  0.06338
C   0.76196 -4.57873  0.59925
H   1.44006 -2.53027  0.80640
H   0.44765 -0.69407  2.90748
C   0.55111 -2.36368  4.26717
C  -0.96851 -0.32315  4.50937
C  -2.57513 -3.66505 -1.27091
H  -2.30549 -1.51951 -1.08454
C  -4.37406 -1.98768 -0.61209
H   1.22358  0.45661 -0.13635
H  -1.00798  0.51628 -0.86937
H  -3.35067  2.00064 -1.91478
H  -5.83810  1.94893 -1.88117
H  -7.06205  1.47563  0.25355
H  -5.76320  1.03574  2.35295
H  -3.27137  1.09908  2.31563
H  -2.51631  4.28480 -0.29741
H  -1.88669  6.16952 -1.78322
H   0.11756  5.98875 -3.27968
H   1.48148  3.88510 -3.26369
H   0.83503  1.98452 -1.78420
H   3.93031  2.54551  0.43376
H   4.07541  4.89631  1.21337
H   2.44747  5.77351  2.90576
H   0.66197  4.25789  3.80126
H   0.52485  1.89897  3.03526
H   2.73282 -0.06505 -1.73678
H   4.87254 -0.53176 -2.93560
H   7.00936 -0.76121 -1.64870
H   6.98514 -0.51369  0.84552
H   4.84395 -0.06819  2.03723
H  -2.96630 -3.65613 -2.30253
H  -1.49250 -3.85814 -1.31620
H  -3.06076 -4.50241 -0.73845
H  -4.70721 -1.87546 -1.65735
H  -4.97979 -2.79450 -0.16125
H  -4.58532 -1.04824 -0.08004
H   1.32361 -1.89564  4.89940
H  -0.08807 -2.98205  4.92264
H   1.05553 -3.02840  3.54726
H  -0.23561  0.15779  5.17875
H  -1.50057  0.46792  3.95594
H  -1.70023 -0.85970  5.13954
H   3.46168 -3.96546  1.07525
H   3.29868 -4.59330 -0.58027
H   3.68552 -2.86371 -0.31179
H   1.13162 -4.92890  1.57801
H  -0.29768 -4.30193  0.71089
H   0.83349 -5.42615 -0.10604
H  -2.07374 -0.12346 -4.75835
H  -1.36959 -1.73365 -5.04623
H  -2.34051 -1.43471 -3.57564
H   0.25710  0.99968 -4.92543
H   1.58683  0.42255 -3.88623
H   1.00974 -0.58698 -5.24461
```

IVbiPr

```
BP86
SCF  =             -2036.20857366
H(0 K)=            -2035.366592
H(298 K)=          -2035.311481
G(298 K)=          -2035.460585
SCF(C6H6) =        -2036.21382310
SCF(D3BJ) =        -2036.50439492
SCF(BS2) =         -4044.05271149
Low Freq. = 9.0453cm-1, 10.0023cm-1
B97D
SCF(C6H6,BS2)=  -4043.14895551

103
```

IVbiPr

```
N   2.64301 -1.97132 -0.43021
C   1.44874 -1.39252 -0.86642
N   1.36454 -1.86832 -2.17421
C   2.43832 -2.68952 -2.51443
C   3.24356 -2.75605 -1.41373
Ni  0.24635 -0.27926  0.23111
P   1.35018  1.57933  0.65302
C   3.03876  1.89716 -0.12012
C   4.05974  2.58419  0.57142
C   5.31004  2.80467 -0.02768
C   5.55495  2.35304 -1.33615
```

C	4.54569	1.67157	-2.03598
C	3.30095	1.43704	-1.42712
C	0.29235	-1.50470	-3.11903
C	-0.44512	-2.74816	-3.64048
H	2.54216	-3.15060	-3.49351
H	4.18040	-3.28466	-1.25703
C	3.22816	-1.77646	0.91339
C	3.10931	-3.05356	1.76221
P	-1.63001	0.64989	-0.52633
C	-2.63041	0.25121	-2.08281
C	-2.41052	0.97644	-3.27559
C	-3.06926	0.62639	-4.46587
C	-3.96560	-0.45616	-4.48673
C	-4.19902	-1.18093	-3.30475
C	-3.53507	-0.83290	-2.11814
C	-3.09005	0.90411	0.64211
C	-3.91065	2.04906	0.54754
C	-5.00091	2.22416	1.41399
C	-5.29757	1.25007	2.38269
C	-4.48823	0.10745	2.48733
C	-3.38568	-0.05655	1.62979
C	-0.22593	-1.45551	1.73681
N	-0.14093	-1.29091	3.11868
C	-0.48531	-2.44649	3.81880
C	-0.81762	-3.38559	2.88479
N	-0.66387	-2.78007	1.63747
C	0.20572	-0.01747	3.77628
C	-0.92003	0.44394	4.71688
H	-0.46802	-2.50760	4.90418
H	-1.14701	-4.41454	3.00673
C	-1.02745	-3.43896	0.36637
C	-2.50862	-3.85457	0.37262
C	1.56407	-0.11511	4.48937
C	-0.09805	-4.62471	0.06246
C	0.56512	3.25967	0.32732
C	-0.26288	3.84285	1.31081
C	-0.94487	5.04314	1.05828
C	-0.81875	5.67889	-0.18899
C	-0.00489	5.10312	-1.17779
C	0.68049	3.90329	-0.92277
C	0.83707	-0.63718	-4.26568
C	4.68252	-1.28611	0.82023
H	-0.39946	-0.90162	-2.50952
H	2.59188	-0.98868	1.35594
H	-0.86723	-2.64493	-0.38512
H	0.28293	0.68850	2.93292
H	-2.72845	-0.92552	1.73627
H	-4.70939	-0.65586	3.24211
H	-6.15213	1.38418	3.05486
H	-5.62363	3.12200	1.33021
H	-3.69306	2.80870	-0.21150
H	-1.72276	1.83082	-3.26812
H	-2.89325	1.21094	-5.37634
H	-4.48576	-0.72540	-5.41215
H	-4.90805	-2.01680	-3.30569
H	-3.73929	-1.39618	-1.20022
H	-0.37646	3.35292	2.28518
H	-1.57558	5.48323	1.83874
H	-1.34975	6.61606	-0.38699
H	0.10429	5.59244	-2.15234
H	1.32655	3.47312	-1.69608
H	2.52289	0.87107	-1.95092
H	4.73067	1.31117	-3.05414
H	6.52885	2.52924	-1.80563
H	6.09330	3.33532	0.52529
H	3.87062	2.95296	1.58645
H	-1.50434	2.04562	-0.87156
H	1.80170	1.96057	1.99096
H	-1.31574	-2.43570	-4.23974
H	0.20348	-3.37342	-4.28054
H	-0.80934	-3.37106	-2.80763
H	0.00598	-0.31769	-4.91554
H	1.33292	0.26608	-3.87429
H	1.56431	-1.19131	-4.88590
H	5.05106	-1.02946	1.82774
H	5.35158	-2.06812	0.41775
H	4.76374	-0.39241	0.18334
H	3.53756	-2.88120	2.76449
H	2.05666	-3.35126	1.88496
H	3.66265	-3.89248	1.30278
H	-0.38434	-5.09290	-0.89467
H	0.94904	-4.29448	-0.01784
H	-0.16358	-5.40200	0.84504
H	-2.79596	-4.23588	-0.62163
H	-2.70619	-4.65667	1.10615
H	-3.16021	-2.99971	0.61622
H	-0.68252	1.44279	5.12036
H	-1.87923	0.50601	4.17833
H	-1.04336	-0.23809	5.57721
H	1.83823	0.86164	4.92240
H	1.53638	-0.85190	5.31231
H	2.35650	-0.41529	3.78485

TS(IV-cis-V)iPr

BP86
SCF = -2036.16841072
H(0 K)= -2035.329255
H(298 K)= -2035.274627
G(298 K)= -2035.418463
SCF(C6H6) = -2036.17574826
SCF(D3BJ) = -2036.46867888
SCF(BS2) = -4043.99868733
Low Freq. = -680.9363cm-1, 20.3403cm-1
B97D
SCF(C6H6,BS2)= -4043.09781968

103

TS(IV-cis-V)iPr

C	2.85765	0.08512	3.31684
C	2.65194	0.36278	1.94300
C	3.13449	1.59853	1.44840
C	3.79018	2.51251	2.29104
C	3.97148	2.22198	3.65288
C	3.50317	1.00067	4.16533
P	1.73861	-0.95529	0.96130
C	3.01781	-1.45134	-0.29968
C	2.71206	-2.54291	-1.15715
C	3.65807	-3.06945	-2.04752
C	4.95293	-2.52510	-2.11650
C	5.27862	-1.45201	-1.27161
C	4.33201	-0.92430	-0.37894
Ni	-0.35468	-0.27804	-0.17055
C	-0.76484	-2.18942	-0.58210

```
N  -1.01065 -2.76072 -1.82549
C  -1.26427 -4.12685 -1.71897
C  -1.19577 -4.44086 -0.39348
N  -0.89769 -3.26073  0.28379
C  -0.99674 -2.07815 -3.14508
H  -1.46501 -4.75385 -2.58291
H  -1.32964 -5.39092  0.11602
C  -0.76164 -3.21453  1.76120
C  -1.85445  0.00577  1.11971
N  -3.20353 -0.10045  0.82398
C  -3.99916  0.20997  1.92374
C  -3.15004  0.52509  2.94555
N  -1.85650  0.39851  2.44616
H  -5.08485  0.19060  1.88752
C  -3.76971 -0.46639 -0.49519
C  -0.65267  0.65483  3.27452
H  -3.35578  0.82711  3.96880
P   0.09621  1.99747 -0.06327
C   1.25623  2.50616 -1.45452
C   1.33604  3.88340 -1.78289
C   2.27835  4.35539 -2.70732
C   3.18051  3.46644 -3.31856
C   3.12698  2.10339 -2.99208
C   2.17435  1.62872 -2.07251
C  -1.45826  2.85051 -0.72480
C  -2.31747  3.47455  0.20714
C  -3.45165  4.19296 -0.21120
C  -3.74732  4.30758 -1.57888
C  -2.90647  3.68748 -2.52135
C  -1.78114  2.96430 -2.09786
H  -0.95212 -1.00550 -2.90374
C  -2.29797 -2.35283 -3.91746
C   0.25307 -2.46325 -3.95066
C   0.32047 -4.19645  2.23409
C  -2.12049 -3.46250  2.43642
H  -0.41081 -2.19150  1.96619
H   0.19394  0.44226  2.60506
C  -0.60905 -0.30073  4.47784
C  -0.57390  2.12533  3.71032
C  -4.41949 -1.85911 -0.44115
H  -2.88850 -0.48929 -1.15858
C  -4.74783  0.60899 -0.99163
H   0.30796 -0.15114 -1.46477
H  -0.90126  0.19844 -1.47392
H  -2.08394  3.41232  1.27594
H  -4.09563  4.67503  0.53311
H  -4.62228  4.87752 -1.90984
H  -3.12772  3.77137 -3.59169
H  -1.12593  2.49813 -2.84182
H   0.64755  4.59283 -1.31036
H   2.31039  5.42404 -2.94863
H   3.91930  3.83602 -4.03797
H   3.82871  1.39652 -3.44821
H   2.15633  0.56348 -1.81935
H   1.71677 -2.99765 -1.10621
H   3.38385 -3.91722 -2.68685
H   5.69438 -2.93548 -2.81009
H   6.28430 -1.01628 -1.30237
H   4.62215 -0.09483  0.27221
H   3.01318  1.84312  0.38974
H   4.15544  3.45952  1.87796
H   4.48126  2.93736  4.30764
H   3.65689  0.75144  5.22177
H   2.51736 -0.87902  3.71337
H  -4.82884 -2.12133 -1.43156
H  -3.69096 -2.63343 -0.15413
H  -5.25727 -1.88250  0.27857
H  -4.27947  1.60459 -0.99580
H  -5.06273  0.36861 -2.02098
H  -5.66130  0.64993 -0.37165
H   0.34151 -0.15892  5.01627
H  -1.43207 -0.10408  5.18800
H  -0.66901 -1.35378  4.16004
H  -1.42076  2.40805  4.36154
H   0.36027  2.28281  4.27369
H  -0.54852  2.78684  2.83033
H   0.45763 -4.09065  3.32326
H   0.04663 -5.24782  2.03417
H   1.28134 -3.96630  1.74797
H  -2.00726 -3.40485  3.53204
H  -2.86439 -2.71044  2.12964
H  -2.51685 -4.46557  2.19758
H  -2.31355 -1.73831 -4.83264
H  -2.38427 -3.40824 -4.22984
H  -3.18513 -2.09657 -3.31593
H   1.17281 -2.22496 -3.39358
H   0.25935 -3.54181 -4.18968
H   0.26723 -1.90692 -4.90297
```

Int(IV-cis-V)iPr

```
BP86
SCF  =           -2036.17350653
H(0 K)=          -2035.333036
H(298 K)=        -2035.278144
G(298 K)=        -2035.421559
SCF(C6H6) =      -2036.18134916
SCF(D3BJ) =      -2036.47542706
SCF(BS2) =       -4044.00565089
Low Freq. = 19.0368cm-1, 25.3733cm-1
B97D
SCF(C6H6,BS2)=  -4043.11017471
```

103

Int(IV-cis-V)iPr

```
C   4.11692 -1.79148  0.96261
C   3.13699 -1.16048  0.15749
C   3.29941 -1.23336 -1.24252
C   4.39437 -1.90323 -1.81823
C   5.35107 -2.52734 -1.00212
C   5.20850 -2.46695  0.39439
P   1.72356 -0.29068  1.04592
C   2.61306  1.30526  1.45391
C   3.83343  1.71372  0.86178
C   4.50166  2.86833  1.29418
C   3.96323  3.66339  2.31950
C   2.74908  3.28294  2.91270
C   2.09001  2.11813  2.49199
Ni -0.31921 -0.27416 -0.11228
C  -0.04509 -2.16904 -0.65815
N   0.07835 -2.71455 -1.93527
C   0.07290 -4.10857 -1.90181
C  -0.03143 -4.47339 -0.59299
N  -0.09659 -3.29506  0.14667
```

C 0.26041 -1.97388 -3.20838
H 0.16054 -4.71717 -2.79669
H -0.04754 -5.45752 -0.13323
C -0.15289 -3.30032 1.62654
C -2.00622 -0.53696 0.91909
N -3.23520 -0.97841 0.45410
C -4.18926 -1.03787 1.46657
C -3.56768 -0.63112 2.61173
N -2.24907 -0.33445 2.27246
H -5.21460 -1.35244 1.29190
C -3.52727 -1.35462 -0.94518
C -1.25868 0.11878 3.28250
H -3.94960 -0.52998 3.62401
P -0.48429 2.03790 0.30302
C 0.60508 2.97173 -0.91559
C 0.42423 4.37403 -1.00883
C 1.23164 5.15684 -1.84555
C 2.25437 4.55990 -2.60390
C 2.45881 3.17568 -2.51045
C 1.64171 2.39278 -1.67599
C -2.14135 2.49377 -0.48689
C -2.31941 2.76616 -1.86515
C -3.56064 3.18273 -2.37226
C -4.66461 3.33355 -1.51485
C -4.51253 3.05576 -0.14578
C -3.26792 2.64239 0.35695
H 0.59305 -0.96894 -2.90638
C -1.05997 -1.85524 -3.98618
C 1.36509 -2.61408 -4.06700
C 1.09718 -3.97864 2.21002
C -1.45553 -3.95619 2.11395
H -0.13273 -2.23372 1.89940
H -0.30291 0.15432 2.73163
C -1.13187 -0.89903 4.42904
C -1.60809 1.51781 3.81458
C -3.75252 -2.87059 -1.06988
H -2.60357 -1.08051 -1.48123
C -4.70094 -0.53971 -1.50770
H -0.14725 0.30648 -1.68907
H -0.96182 0.14321 -1.63639
H -1.46402 2.67824 -2.54336
H -3.66438 3.39788 -3.44225
H -5.63013 3.66948 -1.90843
H -5.36167 3.17529 0.53698
H -3.16215 2.45110 1.43031
H -0.36451 4.85509 -0.41904
H 1.06511 6.23862 -1.90209
H 2.88841 5.17122 -3.25537
H 3.26071 2.69823 -3.08548
H 1.81758 1.31519 -1.59129
H 4.26904 1.11741 0.05458
H 5.44724 3.15301 0.81839
H 4.48445 4.56721 2.65299
H 2.31467 3.88956 3.71557
H 1.16039 1.82533 2.98858
H 2.56475 -0.73928 -1.88678
H 4.50883 -1.92417 -2.90858
H 6.20336 -3.04986 -1.44972
H 5.95125 -2.94433 1.04363
H 4.01866 -1.73856 2.05344
H -3.93189 -3.13631 -2.12522
H -2.87555 -3.43562 -0.71637
H -4.63543 -3.19407 -0.49023
H -4.82360 -0.76526 -2.58051
H -5.65221 -0.79761 -1.00876
H -4.52719 0.54121 -1.39514
H -0.31066 -0.59061 5.09648
H -2.05231 -0.95439 5.03711
H -0.90324 -1.90833 4.05482
H -0.87660 1.81318 4.58571
H -1.56644 2.26206 3.00333
H -2.60942 1.53495 4.28118
H 1.09596 -3.87107 3.30769
H 1.12426 -5.05882 1.97999
H 2.01289 -3.50882 1.81891
H -1.48827 -3.95695 3.21623
H -2.34064 -3.41452 1.74480
H -1.52537 -5.00824 1.78477
H -0.89295 -1.30840 -4.92941
H -1.46097 -2.85304 -4.23651
H -1.81973 -1.30771 -3.40730
H 1.61529 -1.93386 -4.89786
H 2.27543 -2.79258 -3.47419
H 1.04239 -3.56945 -4.51640

TS(IV-cis-V)2iPr
BP86
SCF = -2036.17000536
H(0 K)= -2035.331285
H(298 K)= -2035.276029
G(298 K)= -2035.421069
SCF(C6H6) = -2036.17750737
SCF(D3BJ) = -2036.47076389
SCF(BS2) = -4044.00165520
Low Freq. = -379.0419cm-1,
13.888cm-1
B97D
SCF(C6H6,BS2)= -4043.10783528

103
TS(IV-cis-V)2iPr
C -2.61136 -0.62394 3.13652
C -2.66776 -0.68151 1.72240
C -3.38873 -1.75301 1.14267
C -4.01910 -2.72011 1.94297
C -3.93947 -2.65029 3.34329
C -3.23140 -1.59517 3.94016
P -1.76941 0.71280 0.83512
C -3.10277 1.44077 -0.24392
C -2.77276 2.57592 -1.03209
C -3.74161 3.26293 -1.77565
C -5.08302 2.84207 -1.75963
C -5.43269 1.72996 -0.97691
C -4.46376 1.04148 -0.23059
Ni 0.36854 0.29091 -0.00097
C 0.69368 2.25174 -0.33472
N 0.93110 2.97572 -1.50520
C 1.01178 4.34655 -1.25617
C 0.85963 4.51415 0.08777
N 0.67982 3.24556 0.63107
C 1.07089 2.43201 -2.88045
H 1.16942 5.07801 -2.04285
H 0.86672 5.41699 0.69196
C 0.57248 3.02447 2.09195

```
C   2.02203 -0.10521  0.94018
N   3.34155  0.08015  0.56696
C   4.22591 -0.28756  1.57856
C   3.46326 -0.71391  2.62755
N   2.13171 -0.59984  2.23476
H   5.30518 -0.22414  1.46912
C   3.78468  0.62384 -0.73323
C   1.00318 -0.92951  3.14391
H   3.75464 -1.08526  3.60631
P  -0.06979 -2.01543 -0.02204
C  -1.28248 -2.43888 -1.40196
C  -1.45264 -3.81223 -1.71039
C  -2.40025 -4.23497 -2.65280
C  -3.21759 -3.29529 -3.30723
C  -3.07117 -1.93310 -3.00787
C  -2.11000 -1.51050 -2.07004
C   1.46220 -2.75573 -0.86047
C   2.48421 -3.29756 -0.04341
C   3.60103 -3.94587 -0.59423
C   3.72986 -4.07322 -1.98786
C   2.73259 -3.53411 -2.81860
C   1.61910 -2.88309 -2.26264
H   1.34947  1.37826 -2.74645
C   2.20675  3.13392 -3.64491
C  -0.25770  2.49813 -3.64915
C  -0.59702  3.82430  2.68293
C   1.91723  3.34358  2.76790
H   0.34009  1.95113  2.18198
H   0.09953 -0.69178  2.55926
C   1.04807 -0.04949  4.40439
C   0.98110 -2.42202  3.50614
C   4.45719  1.99344 -0.53561
H   2.84551  0.75512 -1.29534
C   4.68772 -0.37158 -1.47650
H   0.03632 -0.03092 -2.15835
H   0.69637 -0.23375 -1.80266
H   2.39324 -3.22899  1.04564
H   4.36540 -4.36684  0.06913
H   4.59278 -4.59088 -2.42062
H   2.81717 -3.62602 -3.90781
H   0.84247 -2.49115 -2.92756
H  -0.83193 -4.55781 -1.20025
H  -2.50707 -5.30362 -2.87166
H  -3.96363 -3.62541 -4.03837
H  -3.70735 -1.18806 -3.49859
H  -2.02955 -0.44696 -1.82235
H  -1.73756  2.93149 -1.04558
H  -3.44697  4.13613 -2.36988
H  -5.84168  3.37523 -2.34224
H  -6.47439  1.38975 -0.94304
H  -4.77197  0.18771  0.37903
H  -3.45843 -1.83404  0.05502
H  -4.56986 -3.53801  1.46494
H  -4.43115 -3.40772  3.96357
H  -3.17728 -1.51608  5.03220
H  -2.08880  0.21774  3.60731
H   4.76681  2.40735 -1.51006
H   3.77043  2.70851 -0.05505
H   5.36450  1.90759  0.08802
H   4.19126 -1.34594 -1.59936
H   4.93232  0.02726 -2.47551
H   5.64162 -0.52919 -0.94249
H   0.14772 -0.23787  5.01160
H   1.92722 -0.27864  5.03265
H   1.07599  1.02132  4.15194
H   1.89447 -2.72704  4.04823
H   0.11461 -2.62016  4.15827
H   0.86212 -3.03912  2.60238
H  -0.69774  3.58867  3.75570
H  -0.44303  4.91467  2.59639
H  -1.53735  3.54725  2.18086
H   1.84751  3.16908  3.85480
H   2.72310  2.70709  2.36734
H   2.20191  4.40067  2.62181
H   2.39902  2.58665 -4.58227
H   1.94770  4.17000 -3.92330
H   3.13977  3.15416 -3.05902
H  -1.05765  1.95990 -3.11793
H  -0.58123  3.54533 -3.78404
H  -0.13584  2.04712 -4.64848
```

cis-ViPr

```
BP86
SCF  =            -2035.02133743
H(0 K)=           -2034.196186
H(298 K)=         -2034.141643
G(298 K)=         -2034.287514
SCF(C6H6) =       -2035.02817000
SCF(D3BJ) =       -2035.31663788
SCF(BS2) =        -4042.85493971
Low Freq. = 12.6311cm-1, 19.4460cm-
1
B97D
SCF(C6H6,BS2)=  -4041.95019178
```

101

cis-ViPr

```
C   3.08406  2.18968 -0.83724
C   2.22125  1.28021 -1.49551
C   2.79719  0.38074 -2.42295
C   4.17642  0.38409 -2.68635
C   5.01708  1.29008 -2.02020
C   4.46402  2.19085 -1.09318
P   0.36157  1.21517 -1.27538
C  -0.01946  3.04624 -1.28406
C   0.71317  3.94120 -2.10794
C   0.33576  5.28469 -2.23983
C  -0.78960  5.78210 -1.55959
C  -1.53458  4.91106 -0.74881
C  -1.15437  3.56592 -0.61415
P   1.02957 -0.76959  1.36136
Ni -0.64196 -0.26546 -0.01155
C  -2.39128  0.55237  0.29788
N  -3.46155  0.68764 -0.56797
C  -4.56480  1.27505  0.05008
C  -4.20314  1.51760  1.34528
N  -2.88850  1.07322  1.48728
C  -3.41572  0.30221 -1.99441
H  -5.49935  1.46925 -0.46981
H  -4.76544  1.96087  2.16302
C  -2.10383  1.22257  2.73602
C   2.31438 -1.99785  0.78877
C   3.58337 -1.63516  0.27524
C   4.50227 -2.61533 -0.13272
```

```
C   4.17741 -3.97965 -0.04541
C   2.91832 -4.35681  0.45314
C   2.00176 -3.37752  0.86474
C   2.09063  0.57200  2.10834
C   1.62622  1.90855  2.15950
C   2.27557  2.88077  2.93556
C   3.41717  2.54604  3.68350
C   3.89568  1.22544  3.64352
C   3.24112  0.25234  2.87362
C  -1.10205 -2.15051 -0.33436
N  -0.83265 -2.90115 -1.46695
C  -1.30067 -4.20796 -1.34488
C  -1.88724 -4.30482 -0.11497
N  -1.76077 -3.05144  0.48392
C  -0.12305 -2.39778 -2.66384
H  -1.17538 -4.95171 -2.12731
H  -2.37299 -5.14625  0.37223
C  -2.30146 -2.71549  1.81656
H  -2.39205 -0.09076 -2.12398
C  -3.59495  1.53248 -2.89800
C  -4.43766 -0.80814 -2.28749
H  -1.13909  0.73390  2.50742
C  -2.77988  0.49446  3.90928
C  -1.86277  2.70603  3.06315
C  -1.02746 -2.47360 -3.90503
C   1.20643 -3.14061 -2.86623
H   0.07772 -1.33628 -2.42666
H  -1.98469 -1.67028  1.96637
C  -1.66780 -3.58756  2.91116
C  -3.83748 -2.79534  1.80703
H   0.75331  2.18599  1.55742
H   1.89440  3.90864  2.94415
H   3.92906  3.30415  4.28591
H   4.78634  0.94702  4.21873
H   3.62375 -0.77350  2.87183
H   3.85251 -0.57774  0.19621
H   1.02631 -3.68409  1.25859
H   5.47682 -2.30679 -0.52744
H   2.65247 -5.41730  0.53108
H   4.89839 -4.74177 -0.36070
H   1.58858  3.57688 -2.65419
H   0.92636  5.94878 -2.88142
H  -1.08004  6.83323 -1.66056
H  -2.41466  5.27960 -0.20898
H  -1.74332  2.89963  0.02080
H   2.14837 -0.31950 -2.95970
H   4.59221 -0.31861 -3.41702
H   6.09328  1.29930 -2.22459
H   5.11029  2.90084 -0.56502
H   2.67188  2.90320 -0.11903
H  -4.24108 -2.48878  2.78677
H  -4.25995 -2.13030  1.03663
H  -4.18813 -3.82402  1.61094
H  -2.06297 -3.29387  3.89821
H  -1.89470 -4.65874  2.76698
H  -0.57451 -3.45292  2.92811
H  -0.52076 -2.00183 -4.76327
H  -1.25408 -3.51818 -4.18413
H  -1.98114 -1.94775 -3.73711
H   1.74337 -2.71253 -3.72968
H   1.84851 -3.05538 -1.97646
H   1.04163 -4.21227 -3.07870
H  -1.18868  2.78824  3.93126
H  -1.39550  3.23321  2.21726
H  -2.80758  3.22031  3.31543
H  -2.12872  0.54399  4.79737
H  -3.74469  0.96129  4.17664
H  -2.96409 -0.56613  3.67798
H  -4.36228 -1.12299 -3.34203
H  -4.25730 -1.68847 -1.64937
H  -5.47297 -0.46168 -2.11998
H  -3.45896  1.24409 -3.95369
H  -4.60552  1.96726 -2.79724
H  -2.85297  2.30804 -2.65075
```

TS(cis-trans-V)iPr

```
BP86
SCF  =           -2035.00090899
H(0 K)=          -2034.175226
H(298 K)=        -2034.121916
G(298 K)=        -2034.263033
SCF(C6H6) =      -2035.00662768
SCF(D3BJ) =      -2035.29419005
SCF(BS2) =       -4042.83348135
Low Freq. = -8.9627cm-1, 14.4935cm-
1
B97D
SCF(C6H6,BS2)=  -4041.92622171
```

101

TS(cis-trans-V)iPr

```
C   3.01879 -2.07122 -1.38813
C   3.01346 -0.66865 -1.61137
C   4.19853 -0.08878 -2.13535
C   5.32636 -0.87323 -2.41954
C   5.31317 -2.25922 -2.18670
C   4.14840 -2.85320 -1.66738
P   1.41399  0.26805 -1.43433
C   2.07388  2.02924 -1.42018
C   1.84669  2.81140 -2.57669
C   2.28821  4.14412 -2.65384
C   2.96580  4.72398 -1.57007
C   3.19848  3.96214 -0.41154
C   2.75596  2.63130 -0.33725
Ni  0.00105 -0.27393  0.23130
C   0.99215  0.46545  1.71461
N   2.15425 -0.00618  2.33209
C   2.48716  0.74034  3.45903
C   1.53679  1.71006  3.58351
N   0.64894  1.54471  2.52675
C   3.00338 -1.12545  1.86954
C  -0.50280  2.44944  2.33245
H   3.36077  0.52301  4.06735
H   1.42090  2.49663  4.32404
P  -2.01022  0.52442 -0.25244
C  -3.62606 -0.33548 -0.56623
C  -4.60316  0.18918 -1.45609
C  -5.77451 -0.52116 -1.75217
C  -6.01028 -1.78265 -1.17763
C  -5.04979 -2.32574 -0.30843
C  -3.87831 -1.61551 -0.00895
C  -0.46103 -2.25045  0.07137
N  -0.78493 -3.09961  1.12417
C  -1.32070 -4.30812  0.67918
```

```
C  -1.32392 -4.25111 -0.68542
N  -0.79814 -3.01200 -1.04029
C  -0.64692 -2.73745  2.55256
C  -0.66009 -2.58033 -2.45533
H  -1.63916 -5.10028  1.35096
H  -1.64541 -4.98545 -1.41918
C  -2.53265  2.30414 -0.43786
C  -1.62302  3.20852 -1.03892
C  -1.91640  4.57811 -1.13286
C  -3.12363  5.08103 -0.61968
C  -4.03547  4.19896 -0.01109
C  -3.74504  2.82986  0.07936
C  -0.14299 -3.93413  3.37622
C  -1.95556 -2.16814  3.12538
H   0.10700 -1.93146  2.55147
H  -0.18243 -1.58651 -2.39668
C  -2.03433 -2.44159 -3.12916
C   0.25103 -3.54387 -3.23538
H  -0.67337  2.82982 -1.43191
H  -1.19639  5.25139 -1.61108
H  -3.35396  6.14946 -0.69205
H  -4.97773  4.58111  0.39836
H  -4.46608  2.15524  0.55319
H  -4.43572  1.16298 -1.92603
H  -6.50645 -0.08760 -2.44331
H  -6.92719 -2.33517 -1.40856
H  -5.21354 -3.30974  0.14579
H  -3.14318 -2.05910  0.66606
C   4.38957 -0.61670  1.44292
C   3.13091 -2.20930  2.95381
H   2.46858 -1.53197  0.99714
H  -0.92264  2.14376  1.36558
C  -1.56286  2.22450  3.42082
C  -0.04658  3.91313  2.23808
H   1.31904  2.36244 -3.42657
H   2.10418  4.72662 -3.56355
H   3.31110  5.76198 -1.62582
H   3.72881  4.40627  0.43873
H   2.93570  2.05510  0.57537
H   4.23311  0.98848 -2.32601
H   6.22599 -0.39488 -2.82385
H   6.19632 -2.86809 -2.40698
H   4.12080 -3.93276 -1.47745
H   2.11805 -2.54144 -0.97569
H  -1.81205 -1.89481  4.18495
H  -2.25815 -1.26526  2.57098
H  -2.77180 -2.91019  3.07364
H   0.11599 -3.59884  4.39432
H  -0.91834 -4.71317  3.48303
H   0.74973 -4.39564  2.92372
H   0.40032 -3.15631 -4.25677
H   1.24107 -3.64254 -2.76513
H  -0.20036 -4.54835 -3.32499
H  -1.89962 -2.05193 -4.15221
H  -2.54631 -3.41745 -3.20788
H  -2.68561 -1.75017 -2.57574
H  -2.44470  2.85218  3.21190
H  -1.88646  1.17123  3.43559
H  -1.18293  2.49112  4.42368
H  -0.90432  4.54053  1.94673
H   0.33836  4.29309  3.20205
H   0.73823  4.03055  1.47389
H   4.96624 -1.44156  0.99429
H   4.31010  0.17957  0.68915
H   4.95648 -0.23240  2.31002
H   3.66690 -3.08009  2.54091
H   3.70929 -1.84863  3.82288
H   2.14821 -2.54666  3.31402
```

trans-ViPr

```
BP86
SCF  =          -2035.03185071
H(0 K)=         -2034.204803
H(298 K)=       -2034.151315
G(298 K)=       -2034.292196
SCF(C6H6) =     -2035.03781505
SCF(D3BJ) =     -2035.33411013
SCF(BS2) =      -4042.86234148
Low Freq. = 11.2452cm-1, 22.5682cm-
1
B97D
SCF(C6H6,BS2)=  -4041.96270980
```

101

trans-ViPr

```
C   5.69918  2.65082 -0.66267
C   4.63626  2.81297  0.23904
C   3.56792  1.89780  0.25088
C   3.52932  0.80469 -0.64178
C   4.61053  0.66159 -1.54674
C   5.68426  1.56498 -1.55524
P   2.17260 -0.49980 -0.79954
C   3.01240 -1.83342  0.22307
C   2.77681 -3.19370 -0.09642
C   3.44614 -4.22981  0.57258
C   4.38070 -3.93727  1.58143
C   4.62718 -2.59443  1.91421
C   3.95139 -1.55959  1.24823
Ni -0.00000  0.00000 -0.21281
C   0.32392  1.86867 -0.06085
N   0.66200  2.79080 -1.03130
C   0.80781  4.06450 -0.48584
C   0.55778  3.95540  0.85398
N   0.25556  2.61825  1.09779
C   0.82723  2.47622 -2.47469
C  -0.08145  2.04308  2.42157
H   1.07586  4.93039 -1.08395
H   0.55659  4.71000  1.63477
P  -2.17252  0.49995 -0.79977
C  -3.52924 -0.80459 -0.64231
C  -3.56793 -1.89777  0.25026
C  -4.63626 -2.81297  0.23820
C  -5.69904 -2.65077 -0.66365
C  -5.68403 -1.56486 -1.55613
C  -4.61033 -0.66145 -1.54742
C  -0.32395 -1.86868 -0.06106
N  -0.25570 -2.61839  1.09750
C  -0.55792 -3.95551  0.85353
C  -0.80783 -4.06446 -0.48633
N  -0.66195 -2.79070 -1.03164
C   0.08120 -2.04336  2.42138
C  -0.82700 -2.47596 -2.47501
H  -0.55685 -4.71019  1.63424
H  -1.07585 -4.93027 -1.08456
```

```
C  -3.01242  1.83341  0.22296
C  -3.95153  1.55945  1.24798
C  -4.62738  2.59420  1.91404
C  -4.38084  3.93709  1.58149
C  -3.44615  4.22976  0.57279
C  -2.77677  3.19374 -0.09629
C  -1.18975 -1.64508  3.19106
H   0.65489 -1.13302  2.16756
C   0.96926 -2.99271  3.23676
H  -0.94214 -1.37763 -2.49980
C  -2.09516 -3.13249 -3.04246
C   0.43005 -2.86797 -3.26927
C   2.09549  3.13276 -3.04191
C  -0.42970  2.86837 -3.26906
H   0.94233  1.37789 -2.49959
H  -0.65509  1.13274  2.16760
C  -0.96963  2.99232  3.23696
C   1.18943  1.64476  3.19135
H  -4.17335  0.51994  1.51101
H  -2.07352  3.44343 -0.89721
H  -5.35468  2.34754  2.69636
H  -3.24740  5.27077  0.29288
H  -4.91449  4.74376  2.09539
H   2.07366 -3.44328 -0.89746
H   4.17315 -0.52012  1.51146
H   3.24744 -5.27078  0.29247
H   5.35438 -2.34788  2.69666
H   4.91432 -4.74401  2.09526
H  -2.75400 -2.03927  0.96374
H  -4.59865  0.17426 -2.25697
H  -4.63654 -3.65357  0.94168
H  -6.50667 -1.42548 -2.26664
H  -6.53101 -3.36329 -0.67297
H   2.75389  2.03927  0.96426
H   4.59894 -0.17406 -2.25636
H   4.63646  3.65352  0.94258
H   6.50700  1.42563 -2.26564
H   6.53116  3.36332 -0.67182
H   1.85176 -3.31442  2.66362
H   1.31622 -2.47039  4.14341
H   0.41208 -3.88607  3.57229
H  -1.81570 -2.52928  3.40635
H  -0.91531 -1.18538  4.15594
H  -1.79168 -0.92009  2.61984
H  -2.26474 -2.75899 -4.06577
H  -1.99037 -4.23016 -3.11101
H  -2.98198 -2.89498 -2.43616
H   0.28716 -2.61969 -4.33501
H   1.31143 -2.31814 -2.89835
H   0.62127 -3.95398 -3.19942
H  -0.28669  2.62020 -4.33481
H  -1.31116  2.31853 -2.89832
H  -0.62089  3.95438 -3.19912
H   2.98222  2.89514 -2.43553
H   2.26517  2.75937 -4.06524
H   1.99075  4.23044 -3.11034
H   1.81533  2.52896  3.40679
H   0.91490  1.18497  4.15616
H   1.79145  0.91985  2.62012
H  -0.41251  3.88565  3.57265
H  -1.85206  3.31406  2.66374
H  -1.31668  2.46989  4.14350
```

³V

```
BP86
SCF  =            -2035.00866308
H(0 K)=           -2034.184100
H(298 K)=         -2034.129177
G(298 K)=         -2034.277998
SCF(C6H6) =       -2035.01528633
SCF(D3BJ) =       -2035.29590018
SCF(BS2) =        -4042.84020991
Low Freq. = 13.8085cm-1, 18.4146cm-1
B97D
SCF(C6H6,BS2)=  -4041.93100797

101
```

³V

```
C    1.63578  0.87087  3.14223
C    2.47078  0.00067  2.40255
C    3.66107 -0.46335  3.01951
C    4.00646 -0.06068  4.31718
C    3.16689  0.80741  5.03872
C    1.98189  1.26985  4.44428
P    1.91989 -0.65883  0.75245
C    3.41883 -0.43047 -0.31203
C    4.48855  0.44342  0.01838
C    5.59043  0.59880 -0.83508
C    5.66225 -0.10300 -2.05072
C    4.60560 -0.96038 -2.40361
C    3.50396 -1.12115 -1.55106
Ni  -0.22559 -0.45380  0.03875
C   -0.00174 -1.37976 -1.70602
N    0.14995 -2.74573 -1.89650
C    0.31295 -3.06333 -3.24524
C    0.25689 -1.88361 -3.93384
N    0.06534 -0.87627 -2.99017
C    0.19895 -3.74662 -0.80300
H    0.45274 -4.08131 -3.59955
H    0.33858 -1.68395 -4.99907
C   -0.00036  0.56537 -3.32547
P   -0.72003  1.70972  0.31953
C    0.30185  3.13646 -0.25828
C   -0.07992  4.49508 -0.10556
C    0.78070  5.53269 -0.48964
C    2.05124  5.24981 -1.02158
C    2.45264  3.91016 -1.15985
C    1.59049  2.86864 -0.78871
C   -2.44207  2.40426  0.38051
C   -3.00574  3.21004 -0.64170
C   -4.31806  3.69560 -0.54164
C   -5.10959  3.38265  0.57809
C   -4.57299  2.57557  1.59603
C   -3.25732  2.09508  1.49749
C   -1.87085 -1.32245  0.79669
N   -2.00855 -1.94085  2.02522
C   -3.28053 -2.48359  2.19368
C   -3.97661 -2.20928  1.04906
N   -3.11201 -1.50278  0.21484
C   -0.95371 -1.95997  3.06476
H   -3.58547 -3.00562  3.09698
H   -5.00030 -2.44734  0.77284
C   -3.49326 -0.97830 -1.11880
```

```
H   0.00654 -3.15144  0.10560
C   1.59600 -4.37817 -0.69678
C  -0.90478 -4.80386 -0.96881
H  -0.10566  1.05984 -2.34310
C  -1.24034  0.87043 -4.18074
C   1.30213  1.02686 -3.99698
C  -0.59196 -3.40003  3.45843
C  -1.36655 -1.10390  4.27176
H  -0.08281 -1.49231  2.57294
H  -2.66829 -0.29068 -1.37317
C  -4.81126 -0.19255 -1.04600
C  -3.56091 -2.10765 -2.15959
H  -1.05570  4.74001  0.32521
H   0.45889  6.57287 -0.36236
H   2.72149  6.06394 -1.31746
H   3.44341  3.66789 -1.56000
H   1.91710  1.82836 -0.89110
H  -2.39994  3.46726 -1.51720
H  -2.83994  1.48180  2.30390
H  -4.72675  4.32135 -1.34345
H  -5.17617  2.33007  2.47755
H  -6.13213  3.76677  0.65709
H   4.45083  1.01084  0.95280
H   6.39863  1.28127 -0.54752
H   6.52554  0.01807 -2.71339
H   4.64079 -1.51608 -3.34788
H   2.69501 -1.79522 -1.84888
H   4.31870 -1.14840  2.47284
H   4.93253 -0.43188  4.77111
H   3.43662  1.11967  6.05346
H   1.32509  1.95624  4.99121
H   0.71494  1.24425  2.67590
H   0.25290 -3.38754  4.16610
H  -1.43500 -3.91659  3.95108
H  -0.29109 -3.98876  2.57671
H  -0.53241 -1.04569  4.98949
H  -1.61266 -0.07813  3.95376
H  -2.24195 -1.53027  4.79354
H  -4.99733  0.29849 -2.01523
H  -5.67147 -0.85659 -0.84571
H  -4.77272  0.58507 -0.26872
H  -3.84043 -1.69402 -3.14337
H  -2.58881 -2.61261 -2.26460
H  -4.32343 -2.85818 -1.88428
H  -0.87415 -5.50643 -0.11928
H  -1.90365 -4.34137 -0.99407
H  -0.77053 -5.39611 -1.89128
H   1.61404 -5.10138  0.13589
H   1.86803 -4.92162 -1.61908
H   2.35904 -3.60976 -0.49690
H   1.29363  2.12369 -4.10372
H   2.17779  0.74555 -3.39101
H   1.41854  0.58911 -5.00521
H  -1.30410  1.95506 -4.36831
H  -1.19513  0.36353 -5.16134
H  -2.16466  0.55524 -3.66993
```

(iii) PPh2H addition to trans-III

TS(trans-III-trans-V)1iPr

```
BP86
SCF  =      -2036.15384021
H(0 K)=     -2035.314013
H(298 K)=   -2035.259909
G(298 K)=   -2035.402624
SCF(C6H6) =       -2036.16040513
SCF(D3BJ) =       -2036.45177819
SCF(BS2) =        -4043.98608766
Low Freq. = -294.2796cm-1,
13.7653cm-1
B97D
SCF(C6H6,BS2)=  -4043.08075277
103
```

TS(trans-III-trans-V)1iPr

```
C   2.61340  4.62523 -2.68098
C   1.34342  4.04609 -2.51920
C   1.14217  3.02445 -1.57849
C   2.20046  2.55073 -0.75577
C   3.47256  3.16274 -0.92484
C   3.67422  4.17372 -1.87488
P   1.79198  1.40498  0.64229
C   3.36076  0.46196  0.97964
C   3.52752  0.00603  2.31393
C   4.67219 -0.69981  2.71424
C   5.70028 -0.96433  1.79173
C   5.55554 -0.52326  0.46504
C   4.40276  0.17094  0.06514
Ni  0.01880 -0.26218  0.40377
C  -1.00260  0.43881  1.93334
N  -1.27877  1.71599  2.38637
C  -2.03735  1.68738  3.55320
C  -2.26139  0.37779  3.85523
N  -1.63483 -0.37229  2.86574
C  -0.87558  3.01766  1.78687
C  -1.58217 -1.85193  2.92204
H  -2.36140  2.59419  4.05491
H  -2.80788 -0.07580  4.67691
P  -1.62815  0.11553 -1.31777
C  -2.49526 -1.46281 -1.87634
C  -3.80813 -1.41552 -2.40720
C  -4.40979 -2.55558 -2.96049
C  -3.71660 -3.77673 -3.01465
C  -2.41031 -3.83935 -2.50467
C  -1.81041 -2.70117 -1.94138
C   1.18016 -1.40181 -0.68713
N   1.71650 -2.62595 -0.30360
C   2.43907 -3.21262 -1.33764
C   2.37499 -2.36044 -2.39741
N   1.61682 -1.26573 -1.99715
C   1.52231 -3.33562  0.98252
C   1.33071 -0.16129 -2.95434
H   2.94447 -4.16710 -1.22669
H   2.81053 -2.43144 -3.38950
C  -3.13440  1.04719 -0.70501
C  -3.97456  0.62844  0.35174
C  -5.13298  1.34778  0.68315
C  -5.48246  2.50081 -0.04204
C  -4.66048  2.93213 -1.09526
C  -3.49580  2.21313 -1.41841
C   0.49680 -4.46920  0.81724
H   1.11109 -2.55957  1.65108
C   2.85974 -3.82487  1.55620
H   0.76622  0.58164 -2.37410
C   0.45996 -0.67473 -4.11422
```

```
C   2.62682  0.47435 -3.47707
C   0.13044  3.73217  2.70272
C  -2.10517  3.89514  1.50319
H  -0.38290  2.75305  0.83968
H  -1.13351 -2.13955  1.95760
C  -2.98900 -2.46104  3.02191
C  -0.66060 -2.29909  4.06860
H  -3.71945 -0.27620  0.91098
H  -2.86218  2.54574 -2.24871
H  -5.77042  1.00391  1.50554
H  -4.92522  3.82676 -1.66955
H  -6.39137  3.05687  0.21169
H   2.74929  0.24005  3.05018
H   4.31571  0.49823 -0.97476
H   4.77198 -1.02621  3.75608
H   6.34643 -0.72279 -0.26761
H   6.60438 -1.49776  2.10433
H  -1.15187 -1.15229  0.18530
H   0.67604 -0.92413  1.55574
H  -4.36754 -0.47505 -2.38562
H  -0.79533 -2.77477 -1.53948
H  -5.43029 -2.48610 -3.35445
H  -1.85204 -4.78224 -2.53685
H  -4.18792 -4.66503 -3.44821
H   0.14783  2.56864 -1.48337
H   4.30873  2.85060 -0.29059
H   0.50335  4.38346 -3.13792
H   4.66975  4.62101 -1.98025
H   2.77470  5.41633 -3.42084
H   3.29973 -4.64028  0.95475
H   3.58687 -3.00072  1.62583
H   2.69145 -4.22617  2.56978
H   0.85888 -5.23634  0.10985
H   0.32288 -4.96499  1.78750
H  -0.46549 -4.08414  0.44132
H  -0.45675 -1.16329 -3.75183
H   0.16617  0.17616 -4.75048
H   1.01491 -1.39102 -4.74678
H   3.22808 -0.23838 -4.06914
H   2.36714  1.32099 -4.13272
H   3.24094  0.87382 -2.65823
H  -2.57348  4.25685  2.43592
H  -1.78321  4.78354  0.93496
H  -2.86280  3.36169  0.91248
H  -0.32776  3.99365  3.67373
H   1.01625  3.09874  2.86309
H   0.46227  4.66720  2.22185
H   0.34444 -1.86208  3.94892
H  -1.05763 -1.98750  5.05065
H  -0.56878 -3.39833  4.07573
H  -3.49564 -2.19405  3.96594
H  -3.62585 -2.14075  2.18185
H  -2.91352 -3.56055  2.98913
```

Int(trans-III-trans-V)iPr

```
BP86
SCF  =       -2036.15507423
H(0 K)=      -2035.313743
H(298 K)=    -2035.259437
G(298 K)=    -2035.402605
SCF(C6H6) =        -2036.16136918
SCF(D3BJ) =        -2036.45596507
SCF(BS2) =         -4043.98597127
Low Freq. = 8.2462cm-1, 18.5951cm-1
B97D
SCF(C6H6,BS2)=  -4043.08142325
103
```

Int(trans-III-trans-V)iPr

```
C   2.51196  4.83837 -2.14357
C   1.24639  4.23714 -2.03880
C   1.05246  3.12902 -1.19996
C   2.11485  2.58571 -0.42951
C   3.38304  3.21765 -0.53949
C   3.57673  4.31874 -1.38605
P   1.72655  1.31802  0.87104
C   3.34448  0.41607  1.12281
C   3.60617 -0.02562  2.44406
C   4.79868 -0.68708  2.77582
C   5.77636 -0.91691  1.79164
C   5.53544 -0.49004  0.47471
C   4.33466  0.15976  0.14491
Ni  0.05282 -0.35611  0.37741
C  -1.14703  0.11100  1.85919
N  -1.48081  1.31321  2.46143
C  -2.36100  1.12150  3.52286
C  -2.60267 -0.21543  3.60846
N  -1.86160 -0.82329  2.59970
C  -0.99173  2.68388  2.14303
C  -1.82778 -2.29861  2.46373
H  -2.74424  1.94888  4.11207
H  -3.23250 -0.77958  4.29012
P  -1.54553  0.23506 -1.34241
C  -2.46317 -1.32477 -1.84369
C  -3.83986 -1.32252 -2.18721
C  -4.46440 -2.46189 -2.71849
C  -3.73700 -3.64226 -2.94172
C  -2.36855 -3.66206 -2.62547
C  -1.74601 -2.52719 -2.08377
C   1.30026 -1.27841 -0.81533
N   1.91034 -2.48985 -0.51551
C   2.69954 -2.93672 -1.57014
C   2.60093 -2.00660 -2.55916
N   1.75553 -1.00257 -2.09520
C   1.71499 -3.32010  0.69724
C   1.40213  0.14599 -2.97565
H   3.26839 -3.86010 -1.52087
H   3.06385 -1.96465 -3.54038
C  -3.00141  1.26480 -0.75710
C  -3.94978  0.86940  0.21608
C  -5.07162  1.66347  0.50181
C  -5.26911  2.88100 -0.17374
C  -4.33261  3.29829 -1.13314
C  -3.21436  2.49586 -1.42028
C   0.76955 -4.49231  0.38730
H   1.23112 -2.63595  1.41487
C   3.05909 -3.77964  1.27976
H   0.78408  0.80719 -2.35314
C   0.56488 -0.34448 -4.16896
C   2.65517  0.90095 -3.44210
C  -0.05098  3.17373  3.25544
C  -2.15780  3.66000  1.92427
H  -0.42419  2.57521  1.20756
H  -1.15524 -2.47369  1.60846
C  -3.21691 -2.85450  2.11608
C  -1.21868 -2.93458  3.72374
```

H -3.81451 -0.08073 0.74153
H -2.50205 2.81037 -2.19190
H -5.79615 1.33024 1.25360
H -4.47711 4.24324 -1.66855
H -6.14710 3.49792 0.04611
H 2.86281 0.17738 3.22443
H 4.16796 0.47673 -0.88853
H 4.97351 -1.00707 3.80947
H 6.28673 -0.66534 -0.30415
H 6.71621 -1.41694 2.04929
H -0.73534 -1.57271 0.20973
H 0.77837 -0.95979 1.52043
H -4.43305 -0.41533 -2.04178
H -0.68020 -2.57183 -1.84035
H -5.53216 -2.42106 -2.96365
H -1.77782 -4.56974 -2.79649
H -4.22652 -4.52930 -3.35735
H 0.06053 2.66232 -1.14756
H 4.22345 2.84755 0.05663
H 0.40352 4.62683 -2.62160
H 4.56938 4.77997 -1.44880
H 2.66679 5.69853 -2.80339
H 3.56935 -4.51068 0.62751
H 3.73230 -2.92512 1.45025
H 2.88014 -4.27956 2.24654
H 1.20910 -5.17126 -0.36495
H 0.58459 -5.08017 1.30245
H -0.19709 -4.13074 -0.00097
H -0.32800 -0.89495 -3.83559
H 0.22736 0.52449 -4.75730
H 1.15668 -0.99611 -4.83697
H 3.30723 0.27515 -4.07718
H 2.34062 1.77014 -4.04196
H 3.23806 1.28698 -2.59316
H -2.70204 3.86850 2.86259
H -1.74870 4.61958 1.56715
H -2.87204 3.28835 1.17748
H -0.58608 3.26473 4.21793
H 0.80373 2.48928 3.36552
H 0.34368 4.16854 2.99008
H -0.22140 -2.51281 3.93109
H -1.85190 -2.77573 4.61435
H -1.11546 -4.02308 3.57942
H -3.95588 -2.65100 2.91120
H -3.58644 -2.42579 1.17075
H -3.15648 -3.94844 1.99062

(iv) Reaction with Ph_2PPPh_2
VIiPr
BP86
SCF = -2035.01763863
H(0 K)= -2034.193236
H(298 K)= -2034.138554
G(298 K)= -2034.285818
SCF(C6H6) = -2035.02296177
SCF(D3BJ) = -2035.30174350
SCF(BS2) = -4042.85266920
Low Freq. = 14.7460cm-1, 19.5304cm-1
B97D
SCF(C6H6,BS2)= -4041.93896449
101

VIiPr
C -4.63734 -0.70853 -0.44792
C -3.84020 0.24025 -1.13110
C -4.44443 1.44790 -1.55169
C -5.80171 1.70260 -1.29969
C -6.58066 0.75241 -0.62028
C -5.99352 -0.45278 -0.19590
P -2.01823 0.06077 -1.50993
C -1.87831 -1.76192 -1.91318
C -2.90470 -2.40412 -2.64664
C -2.75721 -3.73108 -3.08076
C -1.58450 -4.44725 -2.79182
C -0.55502 -3.81628 -2.07377
C -0.69080 -2.48438 -1.64832
P -0.81827 0.32249 0.50156
Ni 1.29727 0.11151 0.06952
C 2.28846 -1.50619 0.35671
N 3.14625 -2.13560 -0.54596
C 3.80915 -3.22073 0.02447
C 3.37455 -3.30901 1.31764
N 2.45303 -2.27965 1.50289
C 3.41265 -1.61549 -1.90003
H 4.51383 -3.84066 -0.52459
H 3.63340 -4.01867 2.09938
C 1.75278 -2.00897 2.77269
C -1.53700 2.03649 0.89016
C -2.85448 2.24780 1.35575
C -3.32173 3.54599 1.61671
C -2.48576 4.65629 1.41111
C -1.17692 4.45964 0.94308
C -0.70805 3.15944 0.68794
C -1.69200 -0.67340 1.85073
C -2.10366 -2.00929 1.63420
C -2.62181 -2.78564 2.68301
C -2.71782 -2.25615 3.98127
C -2.28639 -0.94037 4.21815
C -1.78280 -0.15741 3.16649
C 2.19835 1.68187 -0.53571
N 2.15677 2.34969 -1.75877
C 3.01316 3.45132 -1.78592
C 3.62345 3.50794 -0.56397
N 3.12981 2.43477 0.17823
C 1.25321 1.96672 -2.86090
H 3.12204 4.09358 -2.65649
H 4.35662 4.21071 -0.17560
C 3.49503 2.12150 1.57034
H 2.70107 -0.77372 -1.99237
C 3.11382 -2.66695 -2.97927
C 4.84562 -1.06357 -1.99436
H 0.99091 -1.25759 2.49702
C 2.71376 -1.40343 3.81030
C 1.05361 -3.27031 3.30208
C 2.04661 1.56298 -4.11461
C 0.23445 3.08066 -3.15186
H 0.71037 1.08727 -2.46624
H 2.98708 1.15229 1.74696
C 2.93211 3.17312 2.54091
C 5.01365 1.93482 1.71779
H -2.01495 -2.44759 0.63556
H -2.95149 -3.81141 2.48231
H -3.12264 -2.86228 4.79875
H -2.35189 -0.51315 5.22546

H -1.47022 0.87261 3.36766
H -3.51846 1.39410 1.51961
H 0.31478 2.99099 0.33399
H -4.34673 3.68867 1.97650
H -0.51457 5.31782 0.78219
H -2.85314 5.66792 1.61613
H -3.82929 -1.86477 -2.87606
H -3.56829 -4.20686 -3.64338
H -1.47493 -5.48612 -3.12109
H 0.36096 -4.36632 -1.82947
H 0.11821 -2.00342 -1.07727
H -3.83920 2.19084 -2.08304
H -6.25095 2.64262 -1.63831
H -7.64062 0.94686 -0.42406
H -6.59488 -1.19800 0.33666
H -4.19309 -1.64940 -0.11213
H 0.42819 -3.00904 4.17081
H 0.39719 -3.70702 2.53385
H 1.77984 -4.03718 3.62716
H 2.16317 -1.13659 4.72813
H 3.50791 -2.11991 4.08710
H 3.19400 -0.49331 3.41611
H 3.27684 -2.23459 -3.98103
H 3.77734 -3.54561 -2.88709
H 2.06883 -3.00990 -2.92001
H 5.01851 -0.61801 -2.98882
H 5.00736 -0.28378 -1.23282
H 5.59699 -1.85953 -1.84756
H 5.25724 1.61511 2.74502
H 5.38493 1.16748 1.01911
H 5.56236 2.87401 1.52550
H 3.14990 2.88633 3.58392
H 3.38481 4.16528 2.36355
H 1.84008 3.26394 2.42594
H -0.48894 2.73669 -3.90931
H -0.32428 3.34472 -2.24015
H 0.72811 3.98990 -3.53994
H 1.35884 1.19546 -4.89438
H 2.60263 2.42026 -4.53475
H 2.77149 0.76456 -3.88699

TS(VI-cis-V)1

BP86
SCF = -2035.01080647
H(0 K)= -2034.186803
H(298 K)= -2034.132841
G(298 K)= -2034.278007
SCF(C6H6) = -2035.01631070
SCF(D3BJ) = -2035.29706901
SCF(BS2) = -4042.84756586
Low Freq. = -47.7275cm-1,
14.7119cm-1
B97D
SCF(C6H6,BS2)= -4041.93055519
101

TS(VI-cis-V)1

C 4.14627 -0.41747 -0.68706
C 3.01711 -0.76194 -1.46479
C 3.15666 -1.77565 -2.43728
C 4.38739 -2.42367 -2.63667
C 5.50024 -2.06839 -1.85932
C 5.37595 -1.06329 -0.88439
P 1.31509 0.02600 -1.30081
C 1.70885 1.80430 -1.76017
C 2.88972 2.16699 -2.44968
C 3.08108 3.48445 -2.89875
C 2.10369 4.46599 -2.67016
C 0.92366 4.11438 -1.99262
C 0.72412 2.79855 -1.54924
P 0.76466 -0.01545 0.95853
Ni -1.19696 -0.17627 0.06065
C -2.25692 1.36498 -0.34362
N -2.93224 1.66956 -1.52325
C -3.66719 2.84885 -1.42389
C -3.47445 3.32456 -0.15676
N -2.62249 2.42660 0.48299
C -2.86867 0.82739 -2.73101
H -4.25660 3.25057 -2.24442
H -3.86998 4.21277 0.32974
C -2.12944 2.59303 1.86321
C 1.73373 -1.48306 1.64239
C 2.94949 -1.37602 2.35315
C 3.58147 -2.52007 2.86577
C 3.01732 -3.79155 2.67086
C 1.80815 -3.91022 1.96546
C 1.17003 -2.76533 1.46293
C 1.59303 1.35908 1.95387
C 2.48652 2.33691 1.46568
C 2.97686 3.34846 2.31092
C 2.59300 3.40000 3.65971
C 1.70559 2.43114 4.16098
C 1.20567 1.43006 3.31512
C -1.98169 -1.91945 -0.01102
N -1.94112 -2.96220 -0.93651
C -2.72248 -4.04969 -0.53838
C -3.28034 -3.71554 0.66293
N -2.82887 -2.43271 0.96891
C -1.14538 -2.93409 -2.17724
H -2.82081 -4.95508 -1.13216
H -3.95067 -4.27754 1.30861
C -3.14774 -1.69828 2.20575
H -2.25572 -0.03309 -2.40265
C -2.15341 1.55790 -3.87866
C -4.27163 0.33548 -3.12427
H -1.44362 1.73562 1.99539
C -3.28341 2.48996 2.87396
C -1.33436 3.90000 2.01077
C -2.05043 -3.06454 -3.41449
C -0.04878 -4.01276 -2.15114
H -0.66551 -1.93762 -2.16922
H -2.65392 -0.71843 2.04691
C -2.51911 -2.38582 3.42895
C -4.66410 -1.49354 2.35014
H 2.79782 2.31904 0.41837
H 3.66952 4.09588 1.90810
H 2.98084 4.18639 4.31596
H 1.40233 2.45422 5.21393
H 0.51304 0.68000 3.71683
H 3.39937 -0.39213 2.51803
H 0.21179 -2.84284 0.93658
H 4.52251 -2.41646 3.41794
H 1.35257 -4.89591 1.81683
H 3.51352 -4.68231 3.07121
H 3.66601 1.41906 -2.63531
H 4.00459 3.74174 -3.42988

```
H   2.25805  5.49271 -3.01887
H   0.14962  4.86786 -1.80824
H  -0.19779  2.52231 -1.02508
H   2.28993 -2.05119 -3.04892
H   4.47569 -3.20460 -3.40007
H   6.46078 -2.57297 -2.00924
H   6.24051 -0.78328 -0.27236
H   4.06116  0.36318  0.07514
H  -4.88135 -0.87924  3.24050
H  -5.07634 -0.98308  1.46455
H  -5.19583 -2.45389  2.47533
H  -2.69945 -1.78708  4.33809
H  -2.95304 -3.38805  3.59633
H  -1.43123 -2.49646  3.29303
H  -1.45551 -2.93589 -4.33441
H  -2.52963 -4.05867 -3.46396
H  -2.84633 -2.30280 -3.40424
H   0.55751 -3.95839 -3.07110
H   0.62233 -3.87412 -1.28882
H  -0.48284 -5.02731 -2.09848
H  -0.88553  3.95350  3.01595
H  -0.51633  3.94453  1.27462
H  -1.97904  4.78752  1.87759
H  -2.89007  2.53138  3.90373
H  -4.00044  3.32233  2.75658
H  -3.83424  1.54368  2.74774
H  -4.20632 -0.34409 -3.99094
H  -4.74464 -0.20697 -2.28952
H  -4.93159  1.17284 -3.41305
H  -2.04039  0.88399 -4.74480
H  -2.72695  2.44075 -4.21432
H  -1.15242  1.89380 -3.56439
```

Int(VI-cis-V)iPr

```
BP86
SCF  =       -2035.01738379
H(0 K)=      -2034.192916
H(298 K)=    -2034.138235
G(298 K)=    -2034.284922
SCF(C6H6) =        -2035.02286792
SCF(D3BJ) =        -2035.30761528
SCF(BS2) =         -4042.85632934
Low Freq. = 15.8093cm-1, 16.3163cm-
1
B97D
SCF(C6H6,BS2)=  -4041.94011329
101
```

Int(VI-cis-V)iPr

```
C   3.27081 -1.74979 -0.99099
C   2.09491 -1.40115 -1.69059
C   1.81851 -2.06962 -2.90595
C   2.68836 -3.04975 -3.41113
C   3.84791 -3.39289 -2.69705
C   4.13419 -2.74125 -1.48636
P   0.85673 -0.07271 -1.18742
C   1.70615  1.43824 -1.89437
C   2.90039  1.39257 -2.65340
C   3.41985  2.55578 -3.24352
C   2.76665  3.78961 -3.08824
C   1.57801  3.84682 -2.34010
C   1.05164  2.68566 -1.75638
P   0.85673  0.07270  1.18742
Ni -1.02193  0.00000  0.00000
C  -2.05162  1.59485 -0.38246
N  -2.81215  1.81805 -1.52487
C  -3.44362  3.05990 -1.50840
C  -3.09657  3.65835 -0.33071
N  -2.25785  2.76783  0.33993
C  -2.88353  0.86572 -2.64871
H  -4.07792  3.41241 -2.31774
H  -3.37684  4.62602  0.07761
C  -1.63748  3.06156  1.64588
C   1.70615 -1.43824  1.89437
C   2.90039 -1.39258  2.65340
C   3.41985 -2.55579  3.24352
C   2.76664 -3.78961  3.08824
C   1.57801 -3.84682  2.34010
C   1.05163 -2.68566  1.75638
C   2.09491  1.40115  1.69058
C   3.27082  1.74978  0.99099
C   4.13420  2.74123  1.48636
C   3.84792  3.39288  2.69705
C   2.68837  3.04974  3.41112
C   1.81852  2.06962  2.90595
C  -2.05163 -1.59485  0.38246
N  -2.25785 -2.76783 -0.33993
C  -3.09657 -3.65834  0.33071
C  -3.44363 -3.05989  1.50840
N  -2.81215 -1.81805  1.52487
C  -1.63749 -3.06156 -1.64588
H  -3.37685 -4.62601 -0.07761
H  -4.07793 -3.41240  2.31774
C  -2.88353 -0.86572  2.64871
H  -2.33910 -0.01726 -2.26432
C  -2.14111  1.41641 -3.87690
C  -4.34180  0.48714 -2.95291
H  -1.02864  2.16250  1.85434
C  -2.70852  3.22615  2.73721
C  -0.71328  4.28694  1.55168
C  -2.70853 -3.22615 -2.73721
C  -0.71329 -4.28694 -1.55168
H  -1.02865 -2.16250 -1.85434
H  -2.33910  0.01726  2.26433
C  -2.14111 -1.41641  3.87690
C  -4.34179 -0.48713  2.95291
H   3.51037  1.25064  0.04878
H   5.03746  3.00088  0.92332
H   4.52486  4.16324  3.08195
H   2.45976  3.54504  4.36158
H   0.91489  1.80306  3.46788
H   3.42776 -0.44286  2.78350
H   0.11592 -2.72527  1.18840
H   4.34563 -2.49490  3.82729
H   1.05347 -4.80099  2.21437
H   3.17660 -4.69515  3.54804
H   3.42776  0.44285 -2.78350
H   4.34563  2.49489 -3.82729
H   3.17661  4.69515 -3.54804
H   1.05348  4.80098 -2.21437
H   0.11592  2.72527 -1.18840
H   0.91488 -1.80305 -3.46788
H   2.45975 -3.54504 -4.36159
H   4.52485 -4.16325 -3.08195
H   5.03745 -3.00089 -0.92333
H   3.51036 -1.25065 -0.04878
```

H -4.37519 0.27753 3.74739
H -4.83923 -0.08172 2.05659
H -4.92544 -1.35444 3.31005
H -2.13620 -0.66639 4.68612
H -2.62762 -2.32848 4.26706
H -1.09784 -1.65999 3.61973
H -2.22832 -3.34934 -3.72270
H -3.33570 -4.11801 -2.55938
H -3.37011 -2.34607 -2.77889
H -0.19922 -4.44592 -2.51356
H 0.05835 -4.13998 -0.78003
H -1.28059 -5.20491 -1.31452
H -0.19921 4.44593 2.51356
H 0.05835 4.13998 0.78002
H -1.28058 5.20492 1.31452
H -2.22831 3.34935 3.72270
H -3.33569 4.11802 2.55938
H -3.37010 2.34608 2.77889
H -4.37520 -0.27752 -3.74738
H -4.83923 0.08173 -2.05659
H -4.92543 1.35445 -3.31005
H -2.13620 0.66639 -4.68612
H -2.62761 2.32849 -4.26706
H -1.09784 1.65999 -3.61973

TS(VI-cis-V)2

BP86
SCF = -2035.01458472
H(0 K)= -2034.190157
H(298 K)= -2034.136153
G(298 K)= -2034.281229
SCF(C6H6) = -2035.02036107
SCF(D3BJ) = -2035.30614865
SCF(BS2) = -4042.85023050
Low Freq. = -43.0142cm-1, 10.0581cm-1
B97D
SCF(C6H6,BS2)= -4041.93687905
101

TS(VI-cis-V)2

C -3.51022 -0.33155 1.19205
C -2.28189 -0.56409 1.85201
C -2.27273 -1.47800 2.93010
C -3.44287 -2.14265 3.33476
C -4.65106 -1.90472 2.66230
C -4.68005 -0.99611 1.59102
P -0.64942 0.29374 1.47828
C -1.08607 2.03832 1.98793
C -2.20383 2.35260 2.80274
C -2.39981 3.65473 3.28880
C -1.49198 4.68011 2.97705
C -0.37795 4.38508 2.17136
C -0.17798 3.08548 1.68603
P -0.80644 -0.12039 -1.37337
Ni 0.87503 -0.18817 0.00844
C 2.25047 1.21003 0.05437
N 3.18429 1.42884 1.05794
C 4.01959 2.50840 0.77694
C 3.62915 2.99648 -0.43757
N 2.56444 2.20416 -0.86618
C 3.25872 0.62824 2.29607
H 4.81010 2.83536 1.44755
H 4.01774 3.82643 -1.02205
C 1.83382 2.44174 -2.12909
C -2.05849 -1.48201 -1.55984
C -3.35492 -1.31102 -2.10476
C -4.19087 -2.41391 -2.33577
C -3.76248 -3.71442 -2.02322
C -2.48050 -3.90023 -1.47585
C -1.64186 -2.80127 -1.24821
C -1.81686 1.36302 -1.94816
C -2.40511 2.36690 -1.14824
C -3.05900 3.46711 -1.73155
C -3.15076 3.58471 -3.12671
C -2.57415 2.59365 -3.94000
C -1.90645 1.50580 -3.35664
C 1.67287 -1.98505 -0.19798
N 1.76017 -3.05382 0.68691
C 2.43300 -4.14215 0.13036
C 2.79129 -3.77628 -1.13539
N 2.33210 -2.47309 -1.31739
C 1.17946 -3.07192 2.04468
H 2.60121 -5.07030 0.67037
H 3.32672 -4.32765 -1.90399
C 2.49239 -1.71345 -2.57347
H 2.48161 -0.14344 2.15126
C 2.89378 1.47966 3.52271
C 4.63702 -0.04082 2.42532
H 1.10026 1.61714 -2.17323
C 2.78077 2.34494 -3.33647
C 1.07975 3.78039 -2.08685
C 2.27376 -3.27486 3.10595
C 0.06875 -4.13053 2.14886
H 0.73578 -2.06562 2.15945
H 2.06439 -0.72398 -2.33486
C 1.66705 -2.35042 -3.70268
C 3.97986 -1.56405 -2.93182
H -2.34246 2.30046 -0.06003
H -3.50173 4.23241 -1.08437
H -3.66383 4.44075 -3.57811
H -2.63765 2.67028 -5.03146
H -1.44638 0.74561 -3.99997
H -3.71002 -0.30679 -2.35529
H -0.64394 -2.94920 -0.82229
H -5.18924 -2.25269 -2.75881
H -2.13141 -4.90874 -1.22550
H -4.41925 -4.57255 -2.20136
H -2.92500 1.57146 3.05952
H -3.27339 3.86767 3.91573
H -1.65044 5.69565 3.35519
H 0.33871 5.17376 1.91471
H 0.68708 2.86219 1.05415
H -1.33546 -1.65250 3.47116
H -3.40976 -2.84129 4.17835
H -5.56575 -2.42258 2.97061
H -5.61810 -0.80567 1.05821
H -3.55238 0.37455 0.35808
H 4.08882 -0.93944 -3.83453
H 4.53804 -1.08820 -2.10912
H 4.44931 -2.53975 -3.15028
H 1.74113 -1.73709 -4.61659
H 2.03008 -3.36471 -3.94767
H 0.60586 -2.41632 -3.41413
H 1.84028 -3.17877 4.11559
H 2.73104 -4.27789 3.03463

H   3.07443 -2.52563 2.99892
H -0.37882 -4.10894 3.15628
H -0.73182 -3.93615 1.41824
H   0.46424 -5.14822 1.98052
H   0.46773 3.89245 -2.99669
H   0.40265 3.82236 -1.21979
H   1.77711 4.63606 -2.03473
H   2.20060 2.41897 -4.27129
H   3.52360 3.16260 -3.34149
H   3.32577 1.38707 -3.33972
H   4.66348 -0.68261 3.32220
H   4.85741 -0.66375 1.54317
H   5.44398 0.70567 2.53285
H   2.87673 0.84912 4.42761
H   3.63079 2.28568 3.68932
H   1.89891 1.93572 3.39784

cis-ViPr

BP86
SCF =   -2035.02133743
H(0 K)=   -2034.196186
H(298 K)=   -2034.141643
G(298 K)=   -2034.287514
SCF(C6H6) =   -2035.02817000
SCF(D3BJ) =   -2035.31663788
SCF(BS2) =   -4042.85493971
Low Freq. = 12.6311cm-1, 19.4460cm-1
B97D
SCF(C6H6,BS2)= -4041.95019178
101

cis-ViPr

C   3.08406 2.18968 -0.83724
C   2.22125 1.28021 -1.49551
C   2.79719 0.38074 -2.42295
C   4.17642 0.38409 -2.68635
C   5.01708 1.29008 -2.02020
C   4.46402 2.19085 -1.09318
P   0.36157 1.21517 -1.27538
C -0.01946 3.04624 -1.28406
C   0.71317 3.94120 -2.10794
C   0.33576 5.28469 -2.23983
C -0.78960 5.78210 -1.55959
C -1.53458 4.91106 -0.74881
C -1.15437 3.56592 -0.61415
P   1.02957 -0.76959 1.36136
Ni -0.64196 -0.26546 -0.01155
C -2.39128 0.55237 0.29788
N -3.46155 0.68764 -0.56797
C -4.56480 1.27505 0.05008
C -4.20314 1.51760 1.34528
N -2.88850 1.07322 1.48728
C -3.41572 0.30221 -1.99441
H -5.49935 1.46925 -0.46981
H -4.76544 1.96087 2.16302
C -2.10383 1.22257 2.73602
C   2.31438 -1.99785 0.78877
C   3.58337 -1.63516 0.27524
C   4.50227 -2.61533 -0.13272
C   4.17741 -3.97965 -0.04541
C   2.91832 -4.35681 0.45314
C   2.00176 -3.37752 0.86474
C   2.09063 0.57200 2.10834
C   1.62622 1.90855 2.15950
C   2.27557 2.88077 2.93556
C   3.41717 2.54604 3.68350
C   3.89568 1.22544 3.64352
C   3.24112 0.25234 2.87362
C -1.10205 -2.15051 -0.33436
N -0.83265 -2.90115 -1.46695
C -1.30067 -4.20796 -1.34488
C -1.88724 -4.30482 -0.11497
N -1.76077 -3.05144 0.48392
C -0.12305 -2.39778 -2.66384
H -1.17538 -4.95171 -2.12731
H -2.37299 -5.14625 0.37223
C -2.30146 -2.71549 1.81656
H -2.39205 -0.09076 -2.12398
C -3.59495 1.53248 -2.89800
C -4.43766 -0.80814 -2.28749
H -1.13909 0.73390 2.50742
C -2.77988 0.49446 3.90928
C -1.86277 2.70603 3.06315
C -1.02746 -2.47360 -3.90503
C   1.20643 -3.14061 -2.86623
H   0.07772 -1.33628 -2.42666
H -1.98469 -1.67028 1.96637
C -1.66780 -3.58756 2.91116
C -3.83748 -2.79534 1.80703
H   0.75331 2.18599 1.55742
H   1.89440 3.90864 2.94415
H   3.92906 3.30415 4.28591
H   4.78634 0.94702 4.21873
H   3.62375 -0.77350 2.87183
H   3.85251 -0.57774 0.19621
H   1.02631 -3.68409 1.25859
H   5.47682 -2.30679 -0.52744
H   2.65247 -5.41730 0.53108
H   4.89839 -4.74177 -0.36070
H   1.58858 3.57688 -2.65419
H   0.92636 5.94878 -2.88142
H -1.08004 6.83323 -1.66056
H -2.41466 5.27960 -0.20898
H -1.74332 2.89963 0.02080
H   2.14837 -0.31950 -2.95970
H   4.59221 -0.31861 -3.41702
H   6.09328 1.29930 -2.22459
H   5.11029 2.90084 -0.56502
H   2.67188 2.90320 -0.11903
H -4.24108 -2.48878 2.78677
H -4.25995 -2.13030 1.03663
H -4.18813 -3.82402 1.61094
H -2.06297 -3.29387 3.89821
H -1.89470 -4.65874 2.76698
H -0.57451 -3.45292 2.92811
H -0.52076 -2.00183 -4.76327
H -1.25408 -3.51818 -4.18413
H -1.98114 -1.94775 -3.73711
H   1.74337 -2.71253 -3.72968
H   1.84851 -3.05538 -1.97646
H   1.04163 -4.21227 -3.07870
H -1.18868 2.78824 3.93126
H -1.39550 3.23321 2.21726
H -2.80758 3.22031 3.31543
H -2.12872 0.54399 4.79737

H -3.74469 0.96129 4.17664
H -2.96409 -0.56613 3.67798
H -4.36228 -1.12299 -3.34203
H -4.25730 -1.68847 -1.64937
H -5.47297 -0.46168 -2.11998
H -3.45896 1.24409 -3.95369
H -4.60552 1.96726 -2.79724
H -2.85297 2.30804 -2.65075

TS(cis-trans-V)iPr

BP86
SCF = -2035.00090899
H(0 K)= -2034.175226
H(298 K)= -2034.121916
G(298 K)= -2034.263033
SCF(C6H6) = -2035.00662768
SCF(D3BJ) = -2035.29419005
SCF(BS2) = -4042.83348135
Low Freq. = -8.9627cm-1, 14.4935cm-1
B97D
SCF(C6H6,BS2)= -4041.92622171
101

TS(cis-trans-V)iPr

C 3.01879 -2.07122 -1.38813
C 3.01346 -0.66865 -1.61137
C 4.19853 -0.08878 -2.13535
C 5.32636 -0.87323 -2.41954
C 5.31317 -2.25922 -2.18670
C 4.14840 -2.85320 -1.66738
P 1.41399 0.26805 -1.43433
C 2.07388 2.02924 -1.42018
C 1.84669 2.81140 -2.57669
C 2.28821 4.14412 -2.65384
C 2.96580 4.72398 -1.57007
C 3.19848 3.96214 -0.41154
C 2.75596 2.63130 -0.33725
Ni 0.00105 -0.27393 0.23130
C 0.99215 0.46545 1.71461
N 2.15425 -0.00618 2.33209
C 2.48716 0.74034 3.45903
C 1.53679 1.71006 3.58351
N 0.64894 1.54471 2.52675
C 3.00338 -1.12545 1.86954
C -0.50280 2.44944 2.33245
H 3.36077 0.52301 4.06735
H 1.42090 2.49663 4.32404
P -2.01022 0.52442 -0.25244
C -3.62606 -0.33548 -0.56623
C -4.60316 0.18918 -1.45609
C -5.77451 -0.52116 -1.75217
C -6.01028 -1.78265 -1.17763
C -5.04979 -2.32574 -0.30843
C -3.87831 -1.61551 -0.00895
C -0.46103 -2.25045 0.07137
N -0.78493 -3.09961 1.12417
C -1.32070 -4.30812 0.67918
C -1.32392 -4.25111 -0.68542
N -0.79814 -3.01200 -1.04029
C -0.64692 -2.73745 2.55256
C -0.66009 -2.58033 -2.45533
H -1.63916 -5.10028 1.35096
H -1.64541 -4.98545 -1.41918
C -2.53265 2.30414 -0.43786
C -1.62302 3.20852 -1.03892
C -1.91640 4.57811 -1.13286
C -3.12363 5.08103 -0.61968
C -4.03547 4.19896 -0.01109
C -3.74504 2.82986 0.07936
C -0.14299 -3.93413 3.37622
C -1.95556 -2.16814 3.12538
H 0.10700 -1.93146 2.55147
H -0.18243 -1.58651 -2.39668
C -2.03433 -2.44159 -3.12916
C 0.25103 -3.54387 -3.23538
H -0.67337 2.82982 -1.43191
H -1.19639 5.25139 -1.61108
H -3.35396 6.14946 -0.69205
H -4.97773 4.58111 0.39836
H -4.46608 2.15524 0.55319
H -4.43572 1.16298 -1.92603
H -6.50645 -0.08760 -2.44331
H -6.92719 -2.33517 -1.40856
H -5.21354 -3.30974 0.14579
H -3.14318 -2.05910 0.66606
C 4.38957 -0.61670 1.44292
C 3.13091 -2.20930 2.95381
H 2.46858 -1.53197 0.99714
H -0.92264 2.14376 1.36558
C -1.56286 2.22450 3.42082
C -0.04658 3.91313 2.23808
H 1.31904 2.36244 -3.42657
H 2.10418 4.72662 -3.56355
H 3.31110 5.76198 -1.62582
H 3.72881 4.40627 0.43873
H 2.93570 2.05510 0.57537
H 4.23311 0.98848 -2.32601
H 6.22599 -0.39488 -2.82385
H 6.19632 -2.86809 -2.40698
H 4.12080 -3.93276 -1.47745
H 2.11805 -2.54144 -0.97569
H -1.81205 -1.89481 4.18495
H -2.25815 -1.26526 2.57098
H -2.77180 -2.91019 3.07364
H 0.11599 -3.59884 4.39432
H -0.91834 -4.71317 3.48303
H 0.74973 -4.39564 2.92372
H 0.40032 -3.15631 -4.25677
H 1.24107 -3.64254 -2.76513
H -0.20036 -4.54835 -3.32499
H -1.89962 -2.05193 -4.15221
H -2.54631 -3.41745 -3.20788
H -2.68561 -1.75017 -2.57574
H -2.44470 2.85218 3.21190
H -1.88646 1.17123 3.43559
H -1.18293 2.49112 4.42368
H -0.90432 4.54053 1.94673
H 0.33836 4.29309 3.20205
H 0.73823 4.03055 1.47389
H 4.96624 -1.44156 0.99429
H 4.31010 0.17957 0.68915
H 4.95648 -0.23240 2.31002
H 3.66690 -3.08009 2.54091
H 3.70929 -1.84863 3.82288
H 2.14821 -2.54666 3.31402

(v) cis-V to trans-V isomerisation

cis-ViPr
BP86
SCF = -2035.02133743
H(0 K)= -2034.196186
H(298 K)= -2034.141643
G(298 K)= -2034.287514
SCF(C6H6) = -2035.02817000
SCF(D3BJ) = -2035.31663788
SCF(BS2) = -4042.85493971
Low Freq. = 12.6311cm-1, 19.4460cm-1
B97D
SCF(C6H6,BS2)= -4041.95019178
101
cis-ViPr
C 3.08406 2.18968 -0.83724
C 2.22125 1.28021 -1.49551
C 2.79719 0.38074 -2.42295
C 4.17642 0.38409 -2.68635
C 5.01708 1.29008 -2.02020
C 4.46402 2.19085 -1.09318
P 0.36157 1.21517 -1.27538
C -0.01946 3.04624 -1.28406
C 0.71317 3.94120 -2.10794
C 0.33576 5.28469 -2.23983
C -0.78960 5.78210 -1.55959
C -1.53458 4.91106 -0.74881
C -1.15437 3.56592 -0.61415
P 1.02957 -0.76959 1.36136
Ni -0.64196 -0.26546 -0.01155
C -2.39128 0.55237 0.29788
N -3.46155 0.68764 -0.56797
C -4.56480 1.27505 0.05008
C -4.20314 1.51760 1.34528
N -2.88850 1.07322 1.48728
C -3.41572 0.30221 -1.99441
H -5.49935 1.46925 -0.46981
H -4.76544 1.96087 2.16302
C -2.10383 1.22257 2.73602
C 2.31438 -1.99785 0.78877
C 3.58337 -1.63516 0.27524
C 4.50227 -2.61533 -0.13272
C 4.17741 -3.97965 -0.04541
C 2.91832 -4.35681 0.45314
C 2.00176 -3.37752 0.86474
C 2.09063 0.57200 2.10834
C 1.62622 1.90855 2.15950
C 2.27557 2.88077 2.93556
C 3.41717 2.54604 3.68350
C 3.89568 1.22544 3.64352
C 3.24112 0.25234 2.87362
C -1.10205 -2.15051 -0.33436
N -0.83265 -2.90115 -1.46695
C -1.30067 -4.20796 -1.34488
C -1.88724 -4.30482 -0.11497
N -1.76077 -3.05144 0.48392
C -0.12305 -2.39778 -2.66384
H -1.17538 -4.95171 -2.12731
H -2.37299 -5.14625 0.37223
C -2.30146 -2.71549 1.81656
H -2.39205 -0.09076 -2.12398
C -3.59495 1.53248 -2.89800
C -4.43766 -0.80814 -2.28749
H -1.13909 0.73390 2.50742
C -2.77988 0.49446 3.90928
C -1.86277 2.70603 3.06315
C -1.02746 -2.47360 -3.90503
C 1.20643 -3.14061 -2.86623
H 0.07772 -1.33628 -2.42666
H -1.98469 -1.67028 1.96637
C -1.66780 -3.58756 2.91116
C -3.83748 -2.79534 1.80703
H 0.75331 2.18599 1.55742
H 1.89440 3.90864 2.94415
H 3.92906 3.30415 4.28591
H 4.78634 0.94702 4.21873
H 3.62375 -0.77350 2.87183
H 3.85251 -0.57774 0.19621
H 1.02631 -3.68409 1.25859
H 5.47682 -2.30679 -0.52744
H 2.65247 -5.41730 0.53108
H 4.89839 -4.74177 -0.36070
H 1.58858 3.57688 -2.65419
H 0.92636 5.94878 -2.88142
H -1.08004 6.83323 -1.66056
H -2.41466 5.27960 -0.20898
H -1.74332 2.89963 0.02080
H 2.14837 -0.31950 -2.95970
H 4.59221 -0.31861 -3.41702
H 6.09328 1.29930 -2.22459
H 5.11029 2.90084 -0.56502
H 2.67188 2.90320 -0.11903
H -4.24108 -2.48878 2.78677
H -4.25995 -2.13030 1.03663
H -4.18813 -3.82402 1.61094
H -2.06297 -3.29387 3.89821
H -1.89470 -4.65874 2.76698
H -0.57451 -3.45292 2.92811
H -0.52076 -2.00183 -4.76327
H -1.25408 -3.51818 -4.18413
H -1.98114 -1.94775 -3.73711
H 1.74337 -2.71253 -3.72968
H 1.84851 -3.05538 -1.97646
H 1.04163 -4.21227 -3.07870
H -1.18868 2.78824 3.93126
H -1.39550 3.23321 2.21726
H -2.80758 3.22031 3.31543
H -2.12872 0.54399 4.79737
H -3.74469 0.96129 4.17664
H -2.96409 -0.56613 3.67798
H -4.36228 -1.12299 -3.34203
H -4.25730 -1.68847 -1.64937
H -5.47297 -0.46168 -2.11998
H -3.45896 1.24409 -3.95369
H -4.60552 1.96726 -2.79724
H -2.85297 2.30804 -2.65075
I(cis-trans-V)1iPr
BP86
SCF = -2035.01501618
H(0 K)= -2034.188905
H(298 K)= -2034.134866

G(298 K)=   -2034.277666
SCF(C6H6) =   -2035.02256713
SCF(D3BJ) =   -2035.31222208
SCF(BS2) =   -4042.84518298
Low Freq. = 15.0052cm-1, 19.6477cm-1
B97D
SCF(C6H6,BS2)= -4041.94415939
101

I(cis-trans-V)1iPr

C	0.75560	2.93215	1.61805
C	-0.48182	2.24498	1.51394
C	-1.18894	1.98707	2.71461
C	-0.69119	2.40810	3.95973
C	0.53951	3.08093	4.04322
C	1.26094	3.33768	2.86218
P	-1.08036	1.78793	-0.20694
C	-2.94074	1.92815	0.05510
C	-3.44847	3.03603	0.78868
C	-4.81711	3.33415	0.82454
C	-5.74007	2.54744	0.11284
C	-5.26253	1.46297	-0.63754
C	-3.88985	1.15925	-0.66327
Ni	-0.12823	-0.33473	-0.20997
C	-1.70942	-1.26515	-0.73493
N	-2.69326	-1.92811	-0.02948
C	-3.60997	-2.54942	-0.87648
C	-3.20640	-2.28096	-2.15449
N	-2.05019	-1.50753	-2.06123
C	-2.76848	-1.96233	1.44547
C	-1.33868	-0.94086	-3.23473
H	-4.46112	-3.11607	-0.50886
H	-3.64175	-2.57206	-3.10647
P	1.72127	0.40518	-1.26723
C	3.41855	0.10039	-0.52508
C	3.81203	0.59355	0.73938
C	5.10699	0.36195	1.23600
C	6.03393	-0.37635	0.48088
C	5.66181	-0.86857	-0.78008
C	4.36967	-0.62546	-1.27735
C	0.78003	-1.83110	0.68727
N	1.11744	-3.07328	0.17840
C	1.61766	-3.92252	1.16464
C	1.60713	-3.21247	2.33028
N	1.10364	-1.94832	2.02992
C	0.99365	-3.46678	-1.24089
C	0.98517	-0.87478	3.04607
H	1.94811	-4.93677	0.95755
H	1.92612	-3.49297	3.33029
C	1.96101	2.16834	-1.83981
C	0.98880	2.70647	-2.72235
C	1.12627	3.99037	-3.26648
C	2.24494	4.77938	-2.94868
C	3.22102	4.26410	-2.08121
C	3.08142	2.97870	-1.53256
C	-0.07019	-4.56500	-1.40710
C	2.35918	-3.88492	-1.80963
H	0.66481	-2.54289	-1.74502
H	0.68671	0.02037	2.47453
C	2.33625	-0.62310	3.73688
C	-0.09739	-1.20839	4.08547
H	0.11612	2.10204	-2.98711
H	0.35472	4.37669	-3.94196
H	2.35547	5.78195	-3.37572
H	4.10277	4.86422	-1.82809
H	3.86075	2.59891	-0.86533
H	3.09817	1.18059	1.32793
H	5.39457	0.76415	2.21449
H	7.04179	-0.55810	0.86952
H	6.38127	-1.43341	-1.38375
H	4.09330	-0.98621	-2.27525
C	-4.13080	-1.45623	1.94491
C	-2.43623	-3.36963	1.96919
H	-1.98111	-1.25366	1.75539
H	-0.38274	-0.56784	-2.81987
C	-1.04400	-2.02583	-4.28205
C	-2.12058	0.23724	-3.83806
H	-2.75337	3.68333	1.33272
H	-5.16402	4.19510	1.40776
H	-6.80951	2.78218	0.13762
H	-5.96041	0.83900	-1.20840
H	-3.55901	0.30914	-1.26270
H	-2.15431	1.47240	2.66781
H	-1.27231	2.21228	4.86894
H	0.92400	3.41638	5.01254
H	2.21308	3.87866	2.90815
H	1.31390	3.16256	0.70408
H	2.27475	-4.05278	-2.89639
H	3.11085	-3.09990	-1.63354
H	2.72041	-4.82468	-1.35550
H	-0.17131	-4.83814	-2.47110
H	0.21227	-5.48026	-0.85728
H	-1.05349	-4.22803	-1.04215
H	-0.17635	-0.37923	4.80639
H	-1.08449	-1.34630	3.62030
H	0.15592	-2.12724	4.64411
H	2.26737	0.30050	4.33425
H	2.60179	-1.44832	4.42219
H	3.14497	-0.50659	3.00098
H	-0.40646	-1.60109	-5.07456
H	-0.51433	-2.88479	-3.84029
H	-1.96500	-2.39839	-4.76501
H	-1.55657	0.65942	-4.68670
H	-3.10786	-0.08540	-4.21431
H	-2.27483	1.03603	-3.09570
H	-4.11286	-1.37441	3.04467
H	-4.36420	-0.46795	1.51921
H	-4.94569	-2.15406	1.68178
H	-2.45928	-3.37978	3.07174
H	-3.17577	-4.10890	1.61361
H	-1.43442	-3.69039	1.64153

TS(cis-trans-V)1iPr

BP86
SCF =   -2035.01105316
H(0 K)=   -2034.185621
H(298 K)=   -2034.132214
G(298 K)=   -2034.273251
SCF(C6H6) =   -2035.01900735
SCF(D3BJ) =   -2035.30623113
SCF(BS2) =   -4042.84171962
Low Freq. = -15.1702cm-1, 14.6138cm-1
B97D
SCF(C6H6,BS2)= -4041.94022400

101
TS(cis-trans-V)1iPr

```
C  -0.06118  0.40251  3.45907
C  -1.23457  0.30318  2.66263
C  -2.34446 -0.36871  3.23294
C  -2.27646 -0.93047  4.51898
C  -1.09920 -0.84014  5.27908
C   0.01000 -0.16096  4.74060
P  -1.19959  1.26550  1.05332
C  -3.05114  1.35487  0.66674
C  -3.58412  2.66648  0.69231
C  -4.94329  2.91311  0.43261
C  -5.80975  1.84666  0.14446
C  -5.30304  0.53672  0.11357
C  -3.94139  0.29757  0.36514
Ni  0.06816 -0.19972 -0.26400
C  -1.39892 -1.10837 -1.10387
N  -1.99585 -2.33491 -0.86157
C  -2.95419 -2.64636 -1.82732
C  -2.97411 -1.60173 -2.70713
N  -2.03064 -0.67885 -2.26425
C  -1.64424 -3.22784  0.26185
C  -1.75717  0.60185 -2.96148
H  -3.53735 -3.56303 -1.80525
H  -3.57861 -1.44016 -3.59558
P   1.54902  1.42733 -0.76472
C   3.31936  1.34607 -0.14586
C   3.66847  1.51521  1.21544
C   5.00902  1.47013  1.63396
C   6.03443  1.24138  0.70003
C   5.70896  1.07631 -0.65513
C   4.36732  1.13777 -1.07008

C   1.41353 -1.64479 -0.40233
N   1.95092 -2.22408 -1.53927
C   2.80547 -3.28275 -1.23222
C   2.82375 -3.38476  0.12908
N   1.97818 -2.39200  0.61902
C   1.64660 -1.79935 -2.92207
C   1.77419 -2.14853  2.06511
H   3.32918 -3.85748 -1.99128
H   3.36909 -4.06331  0.77943
C   1.17659  3.22366 -0.43522
C  -0.09698  3.70754 -0.83180
C  -0.42998  5.06569 -0.73579
C   0.50320  5.98980 -0.23662
C   1.77027  5.53271  0.15928
C   2.10239  4.17204  0.06288
C   0.76650 -2.84480 -3.62827
C   2.93682 -1.50838 -3.70409
H   1.08909 -0.85615 -2.79030
H   1.04152 -1.32484  2.10402
C   3.08356 -1.70049  2.73189
C   1.18900 -3.38119  2.77359
H  -0.83423  3.00685 -1.23863
H  -1.42332  5.40276 -1.05358
H   0.24716  7.05216 -0.16194
H   2.51256  6.24055  0.54614
H   3.10051  3.84854  0.37134
H   2.87669  1.70391  1.94874
H   5.25336  1.61335  2.69281
H   7.07925  1.20264  1.02657
H   6.50137  0.91463 -1.39475
H   4.12421  1.05149 -2.13525
C  -2.87347 -3.55894  1.12313
C  -0.94674 -4.49642 -0.25872
H  -0.93575 -2.62866  0.85738
H  -0.96121  1.06840 -2.35294
C  -1.22135  0.35596 -4.38155
C  -3.00156  1.50354 -2.98413
H  -2.91061  3.50030  0.92291
H  -5.32556  3.93985  0.46032
H  -6.87048  2.03360 -0.05586
H  -5.96922 -0.30392 -0.11321
H  -3.56466 -0.72715  0.31420
H  -3.28687 -0.42119  2.67886
H  -3.15767 -1.43423  4.93375
H  -1.05252 -1.27026  6.28528
H   0.92553 -0.04839  5.33332
H   0.79597  0.96157  3.06412
H   2.68613 -1.06022 -4.67986
H   3.57759 -0.80425 -3.15195
H   3.51550 -2.42820 -3.90122
H   0.53477 -2.51663 -4.65559
H   1.28631 -3.81679 -3.69941
H  -0.18383 -2.99499 -3.09200
H   0.93620 -3.11177  3.81182
H   0.27079 -3.74056  2.28521
H   1.91316 -4.21494  2.80451
H   2.88220 -1.41721  3.77846
H   3.82815 -2.51677  2.74313
H   3.52072 -0.83563  2.21146
H  -0.95700  1.32043 -4.84543
H  -0.32091 -0.27620 -4.37341
H  -1.97957 -0.12752 -5.02323
H  -2.74017  2.47300 -3.44049
H  -3.80892  1.05814 -3.59271
H  -3.38719  1.68566 -1.97095
H  -2.56517 -4.17472  1.98419
H  -3.34465 -2.64467  1.51511
H  -3.62787 -4.13339  0.55714
H  -0.65810 -5.14561  0.58472
H  -1.61915 -5.07993 -0.91222
H  -0.03729 -4.24515 -0.82659
```

I(cis-trans-V)2iPr

```
BP86
SCF  =      -2035.01322842
H(0 K)=     -2034.187575
H(298 K)=   -2034.133376
G(298 K)=   -2034.276402
SCF(C6H6) =       -2035.02105082
SCF(D3BJ) =       -2035.31115669
SCF(BS2) =        -4042.84353752
Low Freq. = 17.4229cm-1, 20.4883cm-
1
B97D
SCF(C6H6,BS2)=  -4041.94411504
101
```

I(cis-trans-V)2iPr

```
C   0.51434  0.05718  3.34127
C  -0.78304 -0.00430  2.76275
C  -1.76580 -0.74929  3.46175
C  -1.46311 -1.41138  4.66356
C  -0.17006 -1.35313  5.20741
```

```
C   0.81773 -0.60673  4.53830
P  -1.05879  1.10066  1.27271
C  -2.95022  1.10702  1.18667
C  -3.53804  2.38189  1.36989
C  -4.93198  2.55997  1.33752
C  -5.77737  1.45903  1.12705
C  -5.21594  0.18413  0.94387
C  -3.82144  0.01405  0.96676
Ni  0.03086 -0.19528 -0.33515
C  -1.49669 -1.16381 -0.99850
N  -1.96711 -2.43958 -0.73768
C  -3.06794 -2.76141 -1.53307
C  -3.30842 -1.67411 -2.32349
N  -2.35183 -0.71637 -1.99744
C  -1.34336 -3.38223  0.21365
C  -2.26702  0.60640 -2.66294
H  -3.58368 -3.71569 -1.46822
H  -4.07423 -1.50477 -3.07562
P   1.42108  1.53134 -0.85219
C   3.09017  1.54710 -0.00424
C   3.29823  2.12198  1.27522
C   4.56798  2.12051  1.87386
C   5.66363  1.53441  1.21483
C   5.47419  0.95023 -0.04822
C   4.20430  0.96099 -0.64943
C   1.46289 -1.47343 -0.83185
N   1.88753 -1.82172 -2.10160
C   2.93479 -2.74203 -2.06673
C   3.18601 -2.99392 -0.74814
N   2.28736 -2.22493 -0.01188
C   1.29459 -1.30222 -3.35180
C   2.26665 -2.20106  1.46890
H   3.40792 -3.13195 -2.96410
H   3.92357 -3.64034 -0.28023
C   0.86808  3.29134 -0.56803
C  -0.49967  3.62019 -0.73995
C  -0.94339  4.95085 -0.73040
C  -0.02932  6.00174 -0.55006
C   1.33259  5.69937 -0.38749
C   1.77455  4.36743 -0.39702
C   0.64075 -2.44399 -4.14862
C   2.32945 -0.51913 -4.17307
H   0.52181 -0.59905 -2.99866
H   1.42857 -1.52982  1.72170
C   3.56885 -1.61273  2.03034
C   1.99342 -3.59821  2.05005
H  -1.23065  2.81806 -0.88585
H  -2.01053  5.16520 -0.85969
H  -0.37292  7.04172 -0.54038
H   2.06207  6.50659 -0.25383
H   2.84303  4.16323 -0.28004
H   2.45479  2.59176  1.79263
H   4.70210  2.57787  2.86086
H   6.65485  1.53647  1.68080
H   6.32080  0.49574 -0.57551
H   4.07463  0.51880 -1.64350
C  -2.34870 -3.86036  1.27273
C  -0.70012 -4.55289 -0.55056
H  -0.55966 -2.78108  0.70415
H  -1.38377  1.07983 -2.19656
C  -2.02064  0.44829 -4.17311
C  -3.51914  1.45557 -2.39334
H  -2.88020  3.24211  1.54119
H  -5.35699  3.55961  1.48287
H  -6.86457  1.59215  1.10484
H  -5.86606 -0.68258  0.77637
H  -3.40512 -0.98331  0.80161
H  -2.79150 -0.78135  3.08180
H  -2.25099 -1.96886  5.18395
H   0.06038 -1.86146  6.14983
H   1.82408 -0.52119  4.96488
H   1.28625  0.66063  2.84824
H   1.85881 -0.12743 -5.09053
H   2.70648  0.33664 -3.58996
H   3.17936 -1.15500 -4.47770
H   0.16041 -2.04634 -5.05839
H   1.38679 -3.19185 -4.47041
H  -0.12825 -2.95609 -3.54773
H   1.85040 -3.51277  3.13947
H   1.08722 -4.05251  1.62128
H   2.83975 -4.28583  1.87300
H   3.49013 -1.54004  3.12760
H   4.43340 -2.26032  1.79846
H   3.76411 -0.60969  1.62639
H  -1.85813  1.44074 -4.62459
H  -1.13644 -0.17268 -4.38158
H  -2.88974 -0.00976 -4.67804
H  -3.38930  2.45088 -2.85026
H  -4.41697  0.99680 -2.84459
H  -3.69689  1.58430 -1.31635
H  -1.83995 -4.52221  1.99293
H  -2.76725 -3.01087  1.83409
H  -3.17779 -4.43386  0.82193
H  -0.20977 -5.24725  0.15196
H  -1.45901 -5.12818 -1.10958
H   0.05774 -4.18883 -1.26250
```

TS(cis-trans-V)2iPr

```
BP86
SCF  =       -2035.00091381
H(0 K)=      -2034.175010
H(298 K)=    -2034.121877
G(298 K)=    -2034.261317
SCF(C6H6) =        -2035.00692581
SCF(D3BJ) =        -2035.29591377
SCF(BS2) =         -4042.83318191
Low Freq. = -10.1844cm-1,
17.3602cm-1
B97D
SCF(C6H6,BS2)=  -4041.92888163
101
```

TS(cis-trans-V)2iPr

```
C   2.35300 -2.30490 -1.80018
C   2.64927 -0.91881 -1.85850
C   3.90673 -0.55035 -2.40701
C   4.81184 -1.51784 -2.86898
C   4.49764 -2.88573 -2.79880
C   3.25871 -3.27222 -2.25812
P   1.29806  0.32255 -1.49715
C   2.38638  1.84442 -1.27870
C   2.24656  2.89673 -2.21313
C   3.04121  4.05390 -2.13265
C   3.99963  4.17964 -1.11490
C   4.15783  3.14026 -0.18091
C   3.35917  1.98791 -0.26114
```

```
Ni -0.06284 -0.27227  0.21048
C   1.23532 -0.03424  1.62532
N   2.25987 -0.83752  2.12001
C   2.87474 -0.27045  3.23422
C   2.24366  0.91575  3.47032
N   1.26662  1.05648  2.49167
C   2.70799 -2.12882  1.55526
C   0.37922  2.24020  2.42742
H   3.69937 -0.74911  3.75519
H   2.41135  1.66466  4.23931
P  -1.91123  0.93805 -0.00975
C  -3.60426  0.39980 -0.56323
C  -4.31270  1.06739 -1.59834
C  -5.55843  0.60348 -2.04429
C  -6.13716 -0.54529 -1.47690
C  -5.44678 -1.22646 -0.46082
C  -4.20167 -0.76188 -0.01244
C  -0.98245 -2.08868  0.18124
N  -1.50259 -2.78868  1.26537
C  -2.30719 -3.85547  0.86539
C  -2.29228 -3.86180 -0.50013
N  -1.48799 -2.79901 -0.90090
C  -1.29682 -2.40794  2.68172
C  -1.23123 -2.49191 -2.33106
H  -2.80637 -4.51846  1.56647
H  -2.77568 -4.53225 -1.20563
C  -2.05605  2.77719 -0.26366
C  -0.92137  3.51056 -0.68447
C  -0.94669  4.91222 -0.76362
C  -2.10812  5.62015 -0.41501
C  -3.24437  4.90914  0.01361
C  -3.22011  3.50984  0.08945
C  -1.10500 -3.65220  3.56578
C  -2.44368 -1.52664  3.20399
H  -0.37155 -1.80657  2.66732
H  -0.49370 -1.67095 -2.30991
C  -2.50127 -1.99704 -3.03981
C  -0.63801 -3.71720 -3.04942
H  -0.00692  2.97263 -0.95145
H  -0.05239  5.44683 -1.10315
H  -2.13046  6.71367 -0.47436
H  -4.15516  5.45002  0.29544
H  -4.11288  2.97378  0.42829
H  -3.87593  1.95784 -2.06063
H  -6.07779  1.14081 -2.84609
H  -7.11120 -0.90532 -1.82482
H  -5.88179 -2.12471 -0.00770
H  -3.68244 -1.30947  0.77770
C   4.15797 -2.02970  1.05630
C   2.55062 -3.26150  2.58350
H   2.03064 -2.30404  0.70516
H  -0.12149  2.14627  1.45212
C  -0.68182  2.18264  3.53594
C   1.18682  3.54618  2.45510
H   1.50939  2.79783 -3.01851
H   2.91709  4.85175 -2.87343
H   4.62343  5.07773 -1.05119
H   4.90872  3.22669  0.61297
H   3.49554  1.18568  0.47110
H   4.17467  0.50771 -2.48164
H   5.77286 -1.19760 -3.28792
H   5.20596 -3.63843 -3.16076
H   2.99675 -4.33515 -2.19266
H   1.39519 -2.61786 -1.37120
H  -2.25039 -1.24298  4.25293
H  -2.52980 -0.60690  2.60228
H  -3.40644 -2.06707  3.17219
H  -0.75517 -3.34357  4.56475
H  -2.05305 -4.19953  3.71133
H  -0.36800 -4.35200  3.13965
H  -0.30888 -3.42635 -4.06026
H   0.23143 -4.12957 -2.51465
H  -1.38810 -4.52022 -3.16280
H  -2.25872 -1.74870 -4.08686
H  -3.28448 -2.77552 -3.05079
H  -2.90933 -1.10007 -2.55414
H  -1.37122  3.03695  3.43515
H  -1.27246  1.25656  3.45928
H  -0.22010  2.23042  4.53892
H   0.50938  4.38873  2.24163
H   1.64873  3.73122  3.44203
H   1.97808  3.53948  1.68918
H   4.44456 -2.96931  0.55764
H   4.27373 -1.21712  0.32394
H   4.85797 -1.86099  1.89426
H   2.78647 -4.22825  2.10812
H   3.24047 -3.13554  3.43672
H   1.52466 -3.30989  2.97639
```

I(cis-trans-V)3iPr

```
BP86
SCF  =       -2035.00095655
H(0 K)=      -2034.175214
H(298 K)=    -2034.121059
G(298 K)=    -2034.265568
SCF(C6H6) =        -2035.00668750
SCF(D3BJ) =        -2035.29554754
SCF(BS2) =         -4042.83339075
Low Freq. = 7.3412cm-1, 16.3565cm-1
B97D
SCF(C6H6,BS2)=  -4041.92772269
101
```

I(cis-trans-V)3iPr

```
C   2.73343 -2.18566 -1.54711
C   2.84389 -0.78163 -1.72371
C   4.05624 -0.29045 -2.27637
C   5.09873 -1.16018 -2.63042
C   4.97011 -2.54708 -2.44342
C   3.77722 -3.05377 -1.89720
P   1.33378  0.28584 -1.48104
C   2.16764  1.96932 -1.39125
C   1.89291  2.88786 -2.43107
C   2.47575  4.16727 -2.44679
C   3.35069  4.55373 -1.41959
C   3.63860  3.65210 -0.37942
C   3.05227  2.37614 -0.36474
Ni -0.02363 -0.27069  0.22823
C   1.14280  0.30073  1.66201
N   2.25357 -0.31055  2.24570
C   2.73083  0.40936  3.33811
C   1.92587  1.50228  3.47139
N   0.98257  1.43597  2.45302
C   2.92041 -1.54762  1.78428
C  -0.05750  2.47232  2.27668
H   3.59216  0.09025  3.91843
```

```
H   1.94895  2.31357  4.19355
P  -1.99261  0.67750 -0.11666
C  -3.64079 -0.07394 -0.53438
C  -4.53082  0.51987 -1.47042
C  -5.73212 -0.10594 -1.83124
C  -6.08574 -1.34816 -1.27591
C  -5.21325 -1.95817 -0.35955
C  -4.01189 -1.33270  0.00359
C  -0.64613 -2.20986  0.12665
N  -1.05816 -3.00117  1.19430
C  -1.70174 -4.16378  0.77029
C  -1.68795 -4.13818 -0.59516
N  -1.04407 -2.96298 -0.97170
C  -0.89940 -2.62403  2.61712
C  -0.84945 -2.58207 -2.39440
H  -2.09911 -4.90717  1.45563
H  -2.07133 -4.85581 -1.31553
C  -2.39783  2.48458 -0.32817
C  -1.42541  3.34181 -0.89713
C  -1.63795  4.72716 -0.98274
C  -2.82726  5.29261 -0.49427
C  -3.80284  4.45710  0.08044
C  -3.59209  3.07336  0.16331
C  -0.52297 -3.84706  3.47031
C  -2.15308 -1.92201  3.16517
H  -0.07086 -1.89483  2.60988
H  -0.23603 -1.66478 -2.35529
C  -2.18855 -2.25476 -3.07317
C  -0.09295 -3.68356 -3.15721
H  -0.48945  2.91406 -1.27129
H  -0.86881  5.36254 -1.43592
H  -2.99474  6.37314 -0.55918
H  -4.73243  4.88763  0.47027
H  -4.36154  2.43629  0.61233
H  -4.27183  1.48105 -1.92447
H  -6.39448  0.37902 -2.55752
H  -7.02574 -1.83457 -1.55732
H  -5.46983 -2.92816  0.08167
H  -3.34765 -1.82811  0.71497
C   4.34680 -1.24715  1.29716
C   2.93585 -2.61190  2.89525
H   2.30068 -1.89500  0.94252
H  -0.48363  2.25644  1.28724
C  -1.15910  2.32085  3.33572
C   0.55489  3.88062  2.25665
H   1.21895  2.58639 -3.24179
H   2.25136  4.85769 -3.26758
H   3.80899  5.54852 -1.42926
H   4.32548  3.94356  0.42357
H   3.28391  1.68670  0.45323
H   4.17974  0.78501 -2.43655
H   6.02147 -0.74836 -3.05536
H   5.78618 -3.22311 -2.71959
H   3.66043 -4.13298 -1.74144
H   1.81128 -2.58875 -1.11297
H  -1.99162 -1.63849  4.21949
H  -2.36733 -1.00839  2.58735
H  -3.03391 -2.58694  3.12394
H  -0.23344 -3.51661  4.48172
H  -1.37531 -4.53875  3.59085
H   0.31789 -4.40949  3.03314
H   0.13591 -3.32889 -4.17560
H   0.85861 -3.94371 -2.66939
H  -0.70074 -4.60083 -3.25574
H  -2.00034 -1.92763 -4.10966
H  -2.84672 -3.14088 -3.11399
H  -2.71998 -1.45109 -2.54461
H  -1.96068  3.05318  3.14511
H  -1.59962  1.31206  3.29225
H  -0.76672  2.49554  4.35402
H  -0.22271  4.60275  1.95966
H   0.93488  4.18730  3.24815
H   1.37759  3.94319  1.52668
H   4.79365 -2.15870  0.86902
H   4.34847 -0.47812  0.51129
H   4.98868 -0.91012  2.13089
H   3.32212 -3.56217  2.49032
H   3.59648 -2.31747  3.72992
H   1.93065 -2.79211  3.30288
```

TS(cis-trans-V)3iPr

```
BP86
SCF  =       -2035.00090899
H(0 K)=      -2034.175226
H(298 K)=    -2034.121916
G(298 K)=    -2034.263033
SCF(C6H6) =        -2035.00662768
SCF(D3BJ) =        -2035.29419005
SCF(BS2) =         -4042.83348135
Low Freq. = -8.9627cm-1, 14.4935cm-
1
B97D
SCF(C6H6,BS2)=  -4041.92622171
101
```

TS(cis-trans-V)3iPr

```
C   3.01879 -2.07122 -1.38813
C   3.01346 -0.66865 -1.61137
C   4.19853 -0.08878 -2.13535
C   5.32636 -0.87323 -2.41954
C   5.31317 -2.25922 -2.18670
C   4.14840 -2.85320 -1.66738
P   1.41399  0.26805 -1.43433
C   2.07388  2.02924 -1.42018
C   1.84669  2.81140 -2.57669
C   2.28821  4.14412 -2.65384
C   2.96580  4.72398 -1.57007
C   3.19848  3.96214 -0.41154
C   2.75596  2.63130 -0.33725
Ni  0.00105 -0.27393  0.23130
C   0.99215  0.46545  1.71461
N   2.15425 -0.00618  2.33209
C   2.48716  0.74034  3.45903
C   1.53679  1.71006  3.58351
N   0.64894  1.54471  2.52675
C   3.00338 -1.12545  1.86954
C  -0.50280  2.44944  2.33245
H   3.36077  0.52301  4.06735
H   1.42090  2.49663  4.32404
P  -2.01022  0.52442 -0.25244
C  -3.62606 -0.33548 -0.56623
C  -4.60316  0.18918 -1.45609
C  -5.77451 -0.52116 -1.75217
C  -6.01028 -1.78265 -1.17763
C  -5.04979 -2.32574 -0.30843
C  -3.87831 -1.61551 -0.00895
```

C -0.46103 -2.25045 0.07137
N -0.78493 -3.09961 1.12417
C -1.32070 -4.30812 0.67918
C -1.32392 -4.25111 -0.68542
N -0.79814 -3.01200 -1.04029
C -0.64692 -2.73745 2.55256
C -0.66009 -2.58033 -2.45533
H -1.63916 -5.10028 1.35096
H -1.64541 -4.98545 -1.41918
C -2.53265 2.30414 -0.43786
C -1.62302 3.20852 -1.03892
C -1.91640 4.57811 -1.13286
C -3.12363 5.08103 -0.61968
C -4.03547 4.19896 -0.01109
C -3.74504 2.82986 0.07936
C -0.14299 -3.93413 3.37622
C -1.95556 -2.16814 3.12538
H 0.10700 -1.93146 2.55147
H -0.18243 -1.58651 -2.39668
C -2.03433 -2.44159 -3.12916
C 0.25103 -3.54387 -3.23538
H -0.67337 2.82982 -1.43191
H -1.19639 5.25139 -1.61108
H -3.35396 6.14946 -0.69205
H -4.97773 4.58111 0.39836
H -4.46608 2.15524 0.55319
H -4.43572 1.16298 -1.92603
H -6.50645 -0.08760 -2.44331
H -6.92719 -2.33517 -1.40856
H -5.21354 -3.30974 0.14579
H -3.14318 -2.05910 0.66606
C 4.38957 -0.61670 1.44292
C 3.13091 -2.20930 2.95381
H 2.46858 -1.53197 0.99714
H -0.92264 2.14376 1.36558
C -1.56286 2.22450 3.42082
C -0.04658 3.91313 2.23808
H 1.31904 2.36244 -3.42657
H 2.10418 4.72662 -3.56355
H 3.31110 5.76198 -1.62582
H 3.72881 4.40627 0.43873
H 2.93570 2.05510 0.57537
H 4.23311 0.98848 -2.32601
H 6.22599 -0.39488 -2.82385
H 6.19632 -2.86809 -2.40698
H 4.12080 -3.93276 -1.47745
H 2.11805 -2.54144 -0.97569
H -1.81205 -1.89481 4.18495
H -2.25815 -1.26526 2.57098
H -2.77180 -2.91019 3.07364
H 0.11599 -3.59884 4.39432
H -0.91834 -4.71317 3.48303
H 0.74973 -4.39564 2.92372
H 0.40032 -3.15631 -4.25677
H 1.24107 -3.64254 -2.76513
H -0.20036 -4.54835 -3.32499
H -1.89962 -2.05193 -4.15221
H -2.54631 -3.41745 -3.20788
H -2.68561 -1.75017 -2.57574
H -2.44470 2.85218 3.21190
H -1.88646 1.17123 3.43559
H -1.18293 2.49112 4.42368
H -0.90432 4.54053 1.94673
H 0.33836 4.29309 3.20205
H 0.73823 4.03055 1.47389
H 4.96624 -1.44156 0.99429
H 4.31010 0.17957 0.68915
H 4.95648 -0.23240 2.31002
H 3.66690 -3.08009 2.54091
H 3.70929 -1.84863 3.82288
H 2.14821 -2.54666 3.31402

I(cis-trans-V)4iPr

BP86
SCF = -2035.00547928
H(0 K)= -2034.179870
H(298 K)= -2034.125794
G(298 K)= -2034.269116
SCF(C6H6) = -2035.01133224
SCF(D3BJ) = -2035.29835031
SCF(BS2) = -4042.83680363
Low Freq. = 13.6962cm-1, 18.2124cm-1
B97D
SCF(C6H6,BS2)= -4041.92877028
101

I(cis-trans-V)4iPr

C 3.46065 -2.12226 -0.75723
C 3.45048 -0.70230 -0.82026
C 4.68608 -0.03886 -0.60418
C 5.86667 -0.75498 -0.34867
C 5.85396 -2.15800 -0.29220
C 4.63872 -2.83593 -0.49731
P 1.82852 0.14447 -1.19706
C 2.36797 1.89840 -1.55037
C 3.35036 2.16746 -2.53811
C 3.62643 3.47953 -2.94943
C 2.92028 4.56238 -2.39632
C 1.93407 4.31465 -1.42824
C 1.66236 3.00008 -1.01136
Ni -0.00472 -0.11874 0.16504
C 0.13649 1.05835 1.69316
N 1.27620 1.51856 2.36831
C 0.96479 2.08601 3.59722
C -0.38739 2.01810 3.73581
N -0.88428 1.40984 2.58948
C 2.69359 1.36676 1.96690
C -2.33665 1.14177 2.47812
H 1.72362 2.49127 4.26035
H -1.03204 2.35648 4.54190
P -1.96816 0.26723 -0.87542
C -3.42006 -0.88884 -0.72384
C -4.50059 -0.83594 -1.64391
C -5.53247 -1.78432 -1.60712
C -5.51074 -2.82349 -0.65886
C -4.44624 -2.89851 0.25299
C -3.41909 -1.94060 0.22374
C -0.01168 -2.08428 -0.18563
N 0.19870 -3.04923 0.78698
C 0.19896 -4.33617 0.25439
C -0.01801 -4.20079 -1.08701
N -0.14441 -2.83666 -1.34242
C 0.45216 -2.75694 2.21263
C -0.36162 -2.30423 -2.71432
H 0.34797 -5.22589 0.86026

H -0.09757 -4.95203 -1.86795
C -2.81225 1.92322 -0.92246
C -2.02348 3.01586 -1.36785
C -2.55953 4.30714 -1.46656
C -3.90736 4.54283 -1.14173
C -4.70742 3.47101 -0.71059
C -4.16732 2.18111 -0.59441
C 1.85627 -3.23509 2.61918
C -0.64120 -3.37030 3.10328
H 0.40391 -1.65263 2.26568
H -0.45189 -1.21407 -2.57648
C -1.66850 -2.85595 -3.30681
C 0.84850 -2.60698 -3.61259
H -0.98062 2.83901 -1.65294
H -1.92635 5.12981 -1.81740
H -4.33132 5.54868 -1.23172
H -5.75985 3.64114 -0.45523
H -4.80574 1.36225 -0.24739
H -4.52271 -0.04497 -2.40144
H -6.35440 -1.71792 -2.32900
H -6.31592 -3.56540 -0.63329
H -4.41866 -3.70138 0.99876
H -2.59254 -1.99850 0.93893
C 3.42694 2.71705 1.98815
C 3.39140 0.32072 2.85012
H 2.64954 0.98770 0.93628
H -2.46701 0.73374 1.46331
C -2.76771 0.08038 3.50373
C -3.14020 2.44385 2.61838
H 3.89532 1.33524 -2.99751
H 4.39167 3.65657 -3.71383
H 3.13305 5.58609 -2.72225
H 1.37279 5.14803 -0.98994
H 0.89126 2.81289 -0.25551
H 4.72529 1.05430 -0.64697
H 6.80416 -0.20838 -0.19242
H 6.77562 -2.71567 -0.09570
H 4.60886 -3.93115 -0.46379
H 2.52964 -2.67466 -0.92084
H -0.48231 -3.07287 4.15337
H -1.64517 -3.03588 2.79627
H -0.62173 -4.47386 3.06376
H 2.07997 -2.91679 3.65133
H 1.92747 -4.33716 2.59092
H 2.62488 -2.82363 1.94555
H 0.68514 -2.15891 -4.60699
H 1.76960 -2.17437 -3.19203
H 0.99160 -3.69359 -3.75424
H -1.86123 -2.36989 -4.27775
H -1.60686 -3.94420 -3.48819
H -2.52533 -2.65658 -2.64594
H -3.82820 -0.17841 3.34855
H -2.16914 -0.83773 3.39249
H -2.65509 0.44217 4.54151
H -4.20734 2.23922 2.43735
H -3.05208 2.87488 3.63223
H -2.81120 3.19428 1.88360
H 4.44309 2.58119 1.58297
H 2.91093 3.46326 1.36530
H 3.53319 3.11691 3.01284
H 4.41235 0.14209 2.47560
H 3.46101 0.65523 3.90101
H 2.84798 -0.63587 2.82368

TS(cis-trans-V)4iPr
BP86
SCF = -2035.00347847
H(0 K)= -2034.177887
H(298 K)= -2034.124791
G(298 K)= -2034.264247
SCF(C6H6) = -2035.00944382
SCF(D3BJ) = -2035.29804843
SCF(BS2) = -4042.83514559
Low Freq. = -30.4470cm-1,
17.0512cm-1
B97D
SCF(C6H6,BS2)= -4041.92865022
101
TS(cis-trans-V)4iPr
C -3.59452 2.18853 -0.34332
C -3.52577 0.77580 -0.47095
C -4.67836 0.03866 -0.09438
C -5.83593 0.67760 0.37676
C -5.87999 2.07649 0.49654
C -4.74821 2.82700 0.13414
P -1.94132 0.03887 -1.13056
C -2.50671 -1.57761 -1.86413
C -3.73339 -1.73478 -2.56133
C -4.01138 -2.90455 -3.28376
C -3.07319 -3.94965 -3.34358
C -1.84459 -3.80395 -2.67769
C -1.56935 -2.63553 -1.95222
Ni 0.02280 0.05587 0.14713
C 0.05711 -1.36053 1.47222
N -0.96306 -2.16955 1.97999
C -0.54445 -2.92339 3.06952
C 0.76275 -2.61353 3.28744
N 1.12585 -1.67712 2.32716
C -2.38917 -2.17564 1.58495
C 2.46832 -1.04879 2.39232
H -1.20365 -3.61292 3.58908
H 1.46041 -2.98343 4.03299
P 1.94665 -0.20499 -0.96553
C 3.27612 1.10690 -0.90032
C 4.20689 1.23789 -1.96263
C 5.15250 2.27429 -1.97688
C 5.18348 3.21958 -0.93606
C 4.26244 3.11361 0.11817
C 3.32411 2.06729 0.13715
C -0.07615 2.04118 0.05698
N -0.36547 2.83626 1.15531
C -0.52023 4.17446 0.80412
C -0.31500 4.24814 -0.54426
N -0.04405 2.95444 -0.98555
C -0.53812 2.32518 2.53121
C 0.22730 2.64738 -2.41502
H -0.75795 4.95062 1.52642
H -0.32948 5.10346 -1.21408
C 2.98417 -1.75457 -0.88265
C 2.29740 -2.99588 -0.89759
C 2.98600 -4.21581 -0.90155
C 4.39267 -4.23665 -0.90114
C 5.09210 -3.01903 -0.88321
C 4.40120 -1.79724 -0.86533

C -2.00873 2.40452 2.96988
C 0.39371 3.06577 3.50520
H -0.24362 1.26121 2.45141
H 0.45946 1.56909 -2.42785
C 1.43972 3.44976 -2.91682
C -1.01811 2.90990 -3.27759
H 1.20292 -2.99550 -0.89413
H 2.42233 -5.15562 -0.91146
H 4.93385 -5.18860 -0.91390
H 6.18825 -3.01683 -0.87379
H 4.97279 -0.86503 -0.83512
H 4.17811 0.52269 -2.79240
H 5.86059 2.35138 -2.80965
H 5.91725 4.03252 -0.95020
H 4.27645 3.84568 0.93378
H 2.60463 1.99167 0.95899
C -2.85223 -3.58769 1.19695
C -3.24404 -1.56712 2.70989
H -2.43268 -1.51816 0.70351
H 2.56590 -0.49728 1.44444
C 2.53782 -0.05962 3.56732
C 3.57570 -2.11050 2.48761
H -4.47084 -0.92577 -2.55287
H -4.96774 -2.99615 -3.81160
H -3.29269 -4.86000 -3.91139
H -1.09451 -4.60172 -2.72627
H -0.60529 -2.51642 -1.44619
H -4.67456 -1.05219 -0.18689
H -6.71005 0.07584 0.65203
H -6.78463 2.57478 0.86057
H -4.76635 3.92019 0.21189
H -2.73207 2.79757 -0.63395
H 0.33920 2.60118 4.50387
H 1.44146 3.03684 3.16469
H 0.09994 4.12413 3.62016
H -2.12782 1.94665 3.96669
H -2.34944 3.45282 3.04043
H -2.66100 1.88037 2.25490
H -0.80124 2.63780 -4.32423
H -1.86579 2.29694 -2.93289
H -1.30530 3.97684 -3.26122
H 1.70488 3.11153 -3.93219
H 1.21130 4.52914 -2.97892
H 2.31627 3.31142 -2.26723
H 3.51896 0.44439 3.57427
H 1.75474 0.70885 3.48532
H 2.41478 -0.57580 4.53622
H 4.55535 -1.62367 2.35648
H 3.58671 -2.60605 3.47562
H 3.46892 -2.87616 1.70518
H -3.88765 -3.54409 0.82094
H -2.22314 -4.00584 0.39661
H -2.84197 -4.27834 2.05964
H -4.29261 -1.48636 2.38211
H -3.21756 -2.18672 3.62420
H -2.89042 -0.55507 2.96356

I(cis-trans-V)5iPr

BP86
SCF = -2035.00381238
H(0 K)= -2034.177938
H(298 K)= -2034.123964
G(298 K)= -2034.267046
SCF(C6H6) = -2035.00975463
SCF(D3BJ) = -2035.29882077
SCF(BS2) = -4042.83565729
Low Freq. = 9.6527cm-1, 17.9003cm-1
B97D
SCF(C6H6,BS2)= -4041.92961109
101

I(cis-trans-V)5iPr

C 3.67007 -2.18149 -0.47588
C 3.54005 -0.76816 -0.45358
C 4.62171 -0.03126 0.09260
C 5.77157 -0.67019 0.58288
C 5.87646 -2.07058 0.55139
C 4.81468 -2.82228 0.02032
P 1.95152 -0.05223 -1.12780
C 2.45563 1.56909 -1.88593
C 3.76037 1.91998 -2.32073
C 3.98775 3.09371 -3.05654
C 2.92505 3.95072 -3.38831
C 1.62090 3.60627 -2.99097
C 1.39208 2.43458 -2.25717
Ni -0.01536 -0.09007 0.15865
C -0.10536 1.32882 1.47671
N 0.84694 2.23793 1.93961
C 0.39708 2.96010 3.03846
C -0.86296 2.52275 3.31020
N -1.16708 1.54599 2.37047
C 2.22194 2.43549 1.43377
C -2.45571 0.81713 2.45906
H 1.00179 3.71997 3.52506
H -1.56360 2.82639 4.08242
P -1.93142 0.18622 -0.94990
C -3.27680 -1.11118 -0.90710
C -4.21058 -1.21213 -1.96980
C -5.16730 -2.23799 -2.00174
C -5.20650 -3.20257 -0.97926
C -4.28007 -3.12878 0.07315
C -3.32940 -2.09400 0.10940
C 0.13186 -2.06896 0.05990
N 0.50592 -2.84889 1.14328
C 0.70104 -4.17962 0.78385
C 0.43521 -4.26398 -0.55337
N 0.08859 -2.98347 -0.98055
C 0.71361 -2.32543 2.51038
C -0.26237 -2.68645 -2.39420
H 1.00576 -4.94405 1.49327
H 0.45272 -5.11920 -1.22332
C -2.93289 1.75402 -0.80899
C -2.21560 2.97695 -0.73913
C -2.87689 4.21152 -0.70347
C -4.28198 4.26710 -0.74575
C -5.00976 3.06787 -0.81278
C -4.34802 1.83012 -0.83677
C 2.20666 -2.27158 2.86972
C -0.09512 -3.14360 3.53124
H 0.32427 -1.29106 2.45233
H -0.53681 -1.61787 -2.39269
C -1.46535 -3.53468 -2.84000
C 0.94960 -2.89546 -3.31699
H -1.12161 2.94988 -0.69649
H -2.29211 5.13665 -0.64575
H -4.80043 5.23150 -0.72471

H -6.10541 3.09220 -0.83782
H -4.94145 0.91177 -0.87175
H -4.17529 -0.48230 -2.78649
H -5.87793 -2.29150 -2.83419
H -5.94944 -4.00677 -1.00701
H -4.29802 -3.87814 0.87283
H -2.60225 -2.04820 0.92694
C 2.40343 3.85493 0.87487
C 3.24083 2.10575 2.53868
H 2.31941 1.70438 0.61628
H -2.52996 0.26706 1.50797
C -2.43152 -0.17928 3.62949
C -3.63829 1.79231 2.58207
H 4.60426 1.25839 -2.10420
H 5.00723 3.33554 -3.37917
H 3.10808 4.86534 -3.96218
H 0.77493 4.24876 -3.26084
H 0.36857 2.15612 -1.98056
H 4.56888 1.06080 0.12711
H 6.59045 -0.06799 0.99346
H 6.77517 -2.56833 0.93071
H 4.88163 -3.91556 -0.01959
H 2.86021 -2.78737 -0.89601
H -0.03390 -2.66857 4.52455
H -1.15688 -3.21504 3.24430
H 0.30209 -4.16878 3.63535
H 2.33173 -1.83059 3.87354
H 2.65069 -3.28236 2.88842
H 2.76589 -1.66460 2.14201
H 0.66976 -2.63720 -4.35217
H 1.78332 -2.24233 -3.01371
H 1.28567 -3.94820 -3.31369
H -1.79116 -3.20747 -3.84112
H -1.19863 -4.60454 -2.91396
H -2.31490 -3.42988 -2.14924
H -3.37581 -0.74893 3.65525
H -1.60008 -0.89234 3.52907
H -2.32418 0.34164 4.59776
H -4.58014 1.23521 2.45138
H -3.67645 2.26837 3.57890
H -3.59638 2.57686 1.81263
H 3.40110 3.94762 0.41569
H 1.65784 4.06883 0.09412
H 2.32145 4.62081 1.66742
H 4.26535 2.21357 2.14784
H 3.14426 2.78553 3.40379
H 3.11763 1.06950 2.89204

TS(cis-trans-V)5iPr

BP86
SCF = -2035.00379971
H(0 K)= -2034.177955
H(298 K)= -2034.124889
G(298 K)= -2034.264392
SCF(C6H6) = -2035.00974917
SCF(D3BJ) = -2035.29848800
SCF(BS2) = -4042.83580217
Low Freq. = -11.6341cm-1, 17.5886cm-1
B97D
SCF(C6H6,BS2)= -4041.92942681
101

TS(cis-trans-V)5iPr

C 3.67327 2.21615 0.50902
C 3.53131 0.80760 0.41430
C 4.57924 0.09608 -0.22276
C 5.71046 0.75466 -0.73032
C 5.82792 2.15066 -0.62571
C 4.79866 2.87765 -0.00452
P 1.96332 0.06495 1.10912
C 2.50153 -1.55014 1.85647
C 3.82737 -1.92302 2.19838
C 4.08462 -3.09304 2.93073
C 3.03239 -3.92455 3.34938
C 1.70956 -3.55901 3.04189
C 1.45048 -2.39149 2.31210
Ni -0.01481 0.09343 -0.16225
C -0.11718 -1.32093 -1.48531
N 0.80955 -2.26080 -1.93759
C 0.35483 -2.95775 -3.05079
C -0.88297 -2.47251 -3.34245
N -1.16921 -1.49355 -2.39965
C 2.14553 -2.54818 -1.37471
C -2.43944 -0.73430 -2.49052
H 0.93986 -3.73615 -3.53196
H -1.57861 -2.74278 -4.13144
P -1.92833 -0.19671 0.94719
C -3.28798 1.08528 0.90789
C -4.23787 1.15526 1.95904
C -5.20382 2.17188 1.99961
C -5.23735 3.15844 0.99800
C -4.29536 3.11571 -0.04214
C -3.33506 2.09018 -0.08735
C 0.12915 2.07155 -0.03594
N 0.50823 2.86835 -1.10513
C 0.69942 4.19364 -0.72410
C 0.42647 4.25704 0.61274
N 0.07951 2.96978 1.01850
C 0.71883 2.36862 -2.48080
C -0.27814 2.65138 2.42589
H 1.00764 4.96906 -1.41990
H 0.43961 5.10179 1.29593
C -2.90534 -1.77804 0.80106
C -2.16611 -2.98899 0.75657
C -2.80562 -4.23473 0.71914
C -4.21012 -4.31366 0.73627
C -4.95937 -3.12662 0.78064
C -4.31922 -1.87748 0.80548
C 2.21184 2.32261 -2.84111
C -0.08969 3.20472 -3.48746
H 0.33066 1.33297 -2.44136
H -0.54843 1.58187 2.40782
C -1.48736 3.48798 2.87696
C 0.92743 2.85208 3.35885
H -1.07186 -2.94199 0.73943
H -2.20462 -5.15045 0.68149
H -4.71176 -5.28690 0.71437
H -6.05471 -3.16964 0.78698
H -4.92824 -0.96880 0.82170
H -4.20770 0.40836 2.76031
H -5.92646 2.20099 2.82290
H -5.98781 3.95534 1.03262
H -4.30830 3.88238 -0.82535
H -2.59552 2.06991 -0.89479
C 2.19417 -3.96337 -0.77803

C 3.23112 -2.33080 -2.44316
H 2.26938 -1.80740 -0.56986
H -2.49870 -0.17941 -1.54102
C -2.39556 0.25427 -3.66689
C -3.64446 -1.68273 -2.60749
H 4.66591 -1.28178 1.91125
H 5.11996 -3.35208 3.18166
H 3.23882 -4.83599 3.92033
H 0.87351 -4.18242 3.37905
H 0.41454 -2.09749 2.10459
H 4.51328 -0.99116 -0.31861
H 6.50382 0.17203 -1.21287
H 6.71169 2.66382 -1.01943
H 4.87629 3.96668 0.09208
H 2.88741 2.80136 0.99822
H -0.02653 2.74869 -4.48954
H -1.15188 3.27004 -3.20076
H 0.30712 4.23183 -3.57184
H 2.33582 1.90443 -3.85478
H 2.65591 3.33343 -2.83695
H 2.77129 1.69816 -2.12881
H 0.64191 2.57980 4.38889
H 1.76462 2.20494 3.05212
H 1.26051 3.90574 3.37145
H -1.81608 3.14625 3.87228
H -1.22635 4.55812 2.96584
H -2.33323 3.38788 2.18099
H -3.32333 0.85058 -3.68897
H -1.54316 0.94339 -3.57876
H -2.31186 -0.27534 -4.63278
H -4.57351 -1.10313 -2.48177
H -3.69224 -2.16487 -3.60090
H -3.62132 -2.46220 -1.83245
H 3.15932 -4.11846 -0.26913
H 1.39885 -4.10263 -0.03029
H 2.08881 -4.73855 -1.55870
H 4.22543 -2.52087 -2.00680
H 3.11382 -3.02107 -3.29721
H 3.21403 -1.29685 -2.82308

I(cis-trans-V)6iPr

BP86
SCF = -2035.02742519
H(0 K)= -2034.200566
H(298 K)= -2034.147000
G(298 K)= -2034.287800
SCF(C6H6) = -2035.03366673
SCF(D3BJ) = -2035.32893572
SCF(BS2) = -4042.85676411
Low Freq. = 14.8551cm-1, 20.6298cm-1
B97D
SCF(C6H6,BS2)= -4041.95818575
101

I(cis-trans-V)6iPr

C 3.46196 -0.71364 2.77091
C 3.21343 -0.19165 1.47832
C 3.84445 1.02876 1.13617
C 4.69776 1.68573 2.03653
C 4.93422 1.14723 3.31426
C 4.30563 -0.05428 3.68073
P 2.13722 -1.22622 0.33172
C 3.13101 -1.07950 -1.24341
C 4.54909 -1.14932 -1.21801
C 5.29758 -1.20713 -2.40192
C 4.65833 -1.21443 -3.65480
C 3.25691 -1.15270 -3.70262
C 2.50953 -1.07797 -2.51561
Ni -0.02721 -0.31134 -0.07791
C 0.56803 1.46390 0.19789
N 0.95881 2.43863 -0.69979
C 1.27555 3.62996 -0.04994
C 1.09112 3.41612 1.28567
N 0.66995 2.09510 1.42747
C 1.09204 2.25657 -2.16564
C 0.36138 1.43988 2.72245
H 1.60546 4.51661 -0.58351
H 1.24006 4.07904 2.13192
P -2.12095 0.27754 -0.89768
C -3.51043 -0.31212 0.22497
C -4.23975 0.54271 1.08888
C -5.31861 0.06560 1.85116
C -5.70667 -1.28164 1.77592
C -5.00059 -2.14478 0.92021
C -3.92462 -1.66739 0.15786
C -0.64813 -2.14697 -0.14327
N -0.86110 -2.94730 0.96152
C -1.30432 -4.21506 0.60256
C -1.37414 -4.23475 -0.76131
N -0.97611 -2.97301 -1.20305
C -0.61272 -2.52774 2.35852
C -0.89291 -2.62068 -2.64168
H -1.53317 -4.98580 1.33323
H -1.66618 -5.02860 -1.44286
C -2.62018 2.09615 -1.00151
C -3.45482 2.42400 -2.09794
C -3.90557 3.73681 -2.31081
C -3.52150 4.76351 -1.43236
C -2.69223 4.46066 -0.34109
C -2.25033 3.14293 -0.12810
C 0.43787 -3.44080 3.01097
C -1.92039 -2.45937 3.16169
H -0.19610 -1.50990 2.24976
H -0.56467 -1.56760 -2.64324
C -2.27422 -2.69306 -3.31130
C 0.14457 -3.50972 -3.34714
H -3.74790 1.62868 -2.79336
H -4.55275 3.95839 -3.16697
H -3.86451 5.79057 -1.59827
H -2.38632 5.25271 0.35213
H -1.60830 2.93000 0.72892
H -3.97214 1.60208 1.15251
H -5.86180 0.75759 2.50539
H -6.55198 -1.65148 2.36594
H -5.29570 -3.19711 0.83506
H -3.40761 -2.35837 -0.51471
C 0.21263 3.25323 -2.93830
C 2.56699 2.36289 -2.58767
H 0.73030 1.22945 -2.34440
H 0.67160 0.39070 2.57263
C 1.19682 2.03694 3.86240
C -1.14342 1.48069 3.03054
H 5.06983 -1.16271 -0.25498
H 6.39121 -1.25524 -2.34483
H 5.24523 -1.26374 -4.57801

```
H   2.73976 -1.14767 -4.66937
H   1.41885 -0.99681 -2.56585
H   3.67664  1.46061  0.14496
H   5.18199  2.62304  1.73893
H   5.60572  1.65675  4.01379
H   4.48752 -0.49056  4.66955
H   3.00749 -1.67123  3.05107
H  -1.70958 -2.09231  4.18049
H  -2.65001 -1.78811  2.68460
H  -2.38180 -3.45813  3.25840
H   0.69864 -3.05348  4.01039
H   0.05413 -4.46786  3.14530
H   1.34964 -3.47725  2.39289
H   0.26206 -3.18543 -4.39465
H   1.12750 -3.44836 -2.85514
H  -0.17768 -4.56579 -3.36152
H  -2.18105 -2.41216 -4.37388
H  -2.69538 -3.71312 -3.27148
H  -2.97299 -1.99062 -2.82994
H   1.06025  1.41969  4.76508
H   2.26984  2.05037  3.61657
H   0.86734  3.05995  4.11855
H  -1.34572  0.94107  3.97154
H  -1.49255  2.52080  3.15734
H  -1.72633  1.00318  2.22694
H   0.28141  3.03588 -4.01748
H  -0.84162  3.18743 -2.63391
H   0.55786  4.29169 -2.78977
H   2.65953  2.16942 -3.66886
H   2.96232  3.37565 -2.39203
H   3.19056  1.62443 -2.06212
```

trans-ViPr

```
BP86
SCF  =          -2035.03185071
H(0 K)=         -2034.204803
H(298 K)=       -2034.151315
G(298 K)=       -2034.292196
SCF(C6H6) =     -2035.03781505
SCF(D3BJ) =     -2035.33411013
SCF(BS2) =      -4042.86234148
Low Freq. = 11.2452cm-1, 22.5682cm-
1
B97D
SCF(C6H6,BS2)=  -4041.96270980

101
```

trans-ViPr

```
C   5.69918  2.65082 -0.66267
C   4.63626  2.81297  0.23904
C   3.56792  1.89780  0.25088
C   3.52932  0.80469 -0.64178
C   4.61053  0.66159 -1.54674
C   5.68426  1.56498 -1.55524
P   2.17260 -0.49980 -0.79954
C   3.01240 -1.83342  0.22307
C   2.77681 -3.19370 -0.09642
C   3.44614 -4.22981  0.57258
C   4.38070 -3.93727  1.58143
C   4.62718 -2.59443  1.91421
C   3.95139 -1.55959  1.24823
Ni -0.00000  0.00000 -0.21281
C   0.32392  1.86867 -0.06085
N   0.66200  2.79080 -1.03130
C   0.80781  4.06450 -0.48584
C   0.55778  3.95540  0.85398
N   0.25556  2.61825  1.09779
C   0.82723  2.47622 -2.47469
C  -0.08145  2.04308  2.42157
H   1.07586  4.93039 -1.08395
H   0.55659  4.71000  1.63477
P  -2.17252  0.49995 -0.79977
C  -3.52924 -0.80459 -0.64231
C  -3.56793 -1.89777  0.25026
C  -4.63626 -2.81297  0.23820
C  -5.69904 -2.65077 -0.66365
C  -5.68403 -1.56486 -1.55613
C  -4.61033 -0.66145 -1.54742
C  -0.32395 -1.86868 -0.06106
N  -0.25570 -2.61839  1.09750
C  -0.55792 -3.95551  0.85353
C  -0.80783 -4.06446 -0.48633
N  -0.66195 -2.79070 -1.03164
C   0.08120 -2.04336  2.42138
C  -0.82700 -2.47596 -2.47501
H  -0.55685 -4.71019  1.63424
H  -1.07585 -4.93027 -1.08456
C  -3.01242  1.83341  0.22296
C  -3.95153  1.55945  1.24798
C  -4.62738  2.59420  1.91404
C  -4.38084  3.93709  1.58149
C  -3.44615  4.22976  0.57279
C  -2.77677  3.19374 -0.09629
C  -1.18975 -1.64508  3.19106
H   0.65489 -1.13302  2.16756
C   0.96926 -2.99271  3.23676
H  -0.94214 -1.37763 -2.49980
C  -2.09516 -3.13249 -3.04246
C   0.43005 -2.86797 -3.26927
C   2.09549  3.13276 -3.04191
C  -0.42970  2.86837 -3.26906
H   0.94233  1.37789 -2.49959
H  -0.65509  1.13274  2.16760
C  -0.96963  2.99232  3.23696
C   1.18943  1.64476  3.19135
H  -4.17335  0.51994  1.51101
H  -2.07352  3.44343 -0.89721
H  -5.35468  2.34754  2.69636
H  -3.24740  5.27077  0.29288
H  -4.91449  4.74376  2.09539
H   2.07366 -3.44328 -0.89746
H   4.17315 -0.52012  1.51146
H   3.24744 -5.27078  0.29247
H   5.35438 -2.34788  2.69666
H   4.91432 -4.74401  2.09526
H  -2.75400 -2.03927  0.96374
H  -4.59865  0.17426 -2.25697
H  -4.63654 -3.65357  0.94168
H  -6.50667 -1.42548 -2.26664
H  -6.53101 -3.36329 -0.67297
H   2.75389  2.03927  0.96426
H   4.59894 -0.17406 -2.25636
H   4.63646  3.65352  0.94258
H   6.50700  1.42563 -2.26564
```

H   6.53116 3.36332 -0.67182
H   1.85176 -3.31442 2.66362
H   1.31622 -2.47039 4.14341
H   0.41208 -3.88607 3.57229
H -1.81570 -2.52928 3.40635
H -0.91531 -1.18538 4.15594
H -1.79168 -0.92009 2.61984
H -2.26474 -2.75899 -4.06577
H -1.99037 -4.23016 -3.11101
H -2.98198 -2.89498 -2.43616
H   0.28716 -2.61969 -4.33501
H   1.31143 -2.31814 -2.89835
H   0.62127 -3.95398 -3.19942
H -0.28669 2.62020 -4.33481
H -1.31116 2.31853 -2.89832
H -0.62089 3.95438 -3.19912
H   2.98222 2.89514 -2.43553
H   2.26517 2.75937 -4.06524
H   1.99075 4.23044 -3.11034
H   1.81533 2.52896 3.40679
H   0.91490 1.18497 4.15616
H   1.79145 0.91985 2.62012
H -0.41251 3.88565 3.57265
H -1.85206 3.31406 2.66374
H -1.31668 2.46989 4.14350

(vi) Optimisations in Benzene Solvent.

I
BP86
SCF =   -1408.51006425
H(0 K)=   -1407.967531
H(298 K)=   -1407.928839
G(298 K)=   -1408.042788
SCF(D3BJ) =   -1408.66597053
SCF(BS2) =   -3081.31380723
Low Freq. = 6.0143cm-1, 18.2573cm-1
B97D
SCF(C6H6,BS2)= -3080.83468321

67
I
Ni -0.17911 -0.62159 -0.19725
P   0.17015 1.41681 -0.79409
N -2.67684 -1.81684 -1.28901
N -3.07109 -0.74359 0.54347
N   2.32929 -2.19865 -0.49728
N   1.69928 -2.11496 1.56760
C -2.02610 -1.07690 -0.30949
C -4.05600 -1.93843 -1.05256
C -4.30500 -1.26216 0.11888
C -1.97781 -2.38477 -2.43116
H -0.90111 -2.35042 -2.19689
H -2.16435 -1.80812 -3.35515
H -2.28442 -3.43174 -2.59636
C -2.87430 0.01328 1.77119
H -1.84931 0.41482 1.74652
H -2.99599 -0.62673 2.66452
H -3.58357 0.85495 1.83458
C -4.97729 -2.67981 -1.96818
H -4.96951 -2.26380 -2.99278
H -6.01251 -2.62353 -1.59621
H -4.71435 -3.75100 -2.05035
C -5.57771 -1.05110 0.87531
H -5.52577 -1.46167 1.90077
H -6.41523 -1.54935 0.36210
H -5.83563 0.02041 0.96756
C   1.31927 -1.67634 0.30374
C   3.27935 -2.93070 0.23290
C   2.88258 -2.87126 1.54854
C   3.49992 -3.44654 2.78333
H   3.75721 -2.66527 3.52255
H   4.42856 -3.98305 2.53250
H   2.82988 -4.16536 3.29074
C   4.45502 -3.59979 -0.40492
H   4.14917 -4.34658 -1.16095
H   5.05492 -4.12528 0.35474
H   5.12224 -2.87836 -0.91250
C   2.35830 -2.01434 -1.93988
H   2.09782 -2.94561 -2.47560
H   3.35442 -1.67856 -2.27427
H   1.61329 -1.23820 -2.18044
C   0.93761 -1.79921 2.76565
H -0.02238 -1.37430 2.42993
H   1.45807 -1.05778 3.39839
H   0.74875 -2.70760 3.36346
C -0.73906 2.74527 0.17144
C -2.03672 3.14569 -0.22023
H -2.46658 2.75358 -1.14959
C -2.77884 4.04287 0.56405
H -3.77892 4.35022 0.23727
C -2.23982 4.55418 1.75825
H -2.81759 5.25597 2.36908
C -0.94916 4.16380 2.15600
H -0.51527 4.56413 3.07942
C -0.20576 3.26767 1.37118
H   0.80530 2.98207 1.68382
C   1.86971 2.22299 -0.93725
C   2.13580 3.29419 -1.81757
C   2.92257 1.71440 -0.14941
C   3.42419 3.84485 -1.90557
C   4.20909 2.27600 -0.22203
C   4.46310 3.34037 -1.10291
H   1.32749 3.70149 -2.43662
H   2.72148 0.85878 0.50696
H   3.61817 4.67241 -2.59736
H   5.01633 1.87540 0.40176
H   5.46741 3.77285 -1.16915
H -0.29746 1.86058 -2.09757

TS(II-trans-III)
BP86
SCF =   -1408.48949344
H(0 K)=   -1407.948387
H(298 K)=   -1407.910275
G(298 K)=   -1408.021318
SCF(D3BJ) =   -1408.64580899
SCF(BS2) =   -3081.28789302
Low Freq. = -87.8670cm-1, 14.1459cm-1
B97D
SCF(C6H6,BS2)= -3080.80564968

67
TS(II-trans-III)
Ni -0.22788 -0.54354 -0.24701
P 0.27951 1.51606 0.07782
N -2.71531 -2.08798 -0.83220
N -3.04290 -0.69875 0.79254
N 2.47036 -1.76723 -0.91671
N 1.61716 -2.63257 0.86514
C -2.05553 -1.12143 -0.08756
C -4.05133 -2.25150 -0.43908
C -4.25781 -1.37181 0.59810
C -2.09060 -2.85566 -1.90232
H -1.00489 -2.69804 -1.82741
H -2.43701 -2.51589 -2.89431
H -2.32339 -3.92771 -1.79021
C -2.85774 0.33820 1.80119
H -1.81131 0.67590 1.74620
H -3.06833 -0.05664 2.81003
H -3.52067 1.19752 1.60509
C -4.97856 -3.22742 -1.09069
H -5.07937 -3.04127 -2.17543
H -5.98333 -3.15325 -0.64653
H -4.63993 -4.27312 -0.96924
C -5.47537 -1.10158 1.42335
H -5.30985 -1.32193 2.49408
H -6.31451 -1.72793 1.08281
H -5.79736 -0.04655 1.35445
C 1.35414 -1.68454 -0.10810
C 3.39274 -2.72294 -0.46490
C 2.85180 -3.27183 0.67546
C 3.37307 -4.32886 1.59617
H 3.47747 -3.96074 2.63317
H 4.36654 -4.66812 1.26363
H 2.71444 -5.21641 1.62962
C 4.68142 -3.00418 -1.16953
H 4.51973 -3.34810 -2.20757
H 5.24143 -3.79228 -0.64245
H 5.33061 -2.11103 -1.21963
C 2.67829 -0.92720 -2.09126
H 2.69377 -1.53403 -3.01334
H 3.62807 -0.37416 -2.00705
H 1.84214 -0.21407 -2.13106
C 0.71418 -2.90080 1.97748
H -0.24271 -2.40744 1.74297
H 1.11113 -2.49516 2.92456
H 0.55167 -3.98552 2.09287
H -0.34748 -0.65753 -1.68345
C -0.79987 3.01857 -0.11528
C -1.97070 2.90362 -0.90566
H -2.14500 1.96883 -1.45179
C -2.88913 3.96018 -1.00145
H -3.78223 3.84823 -1.62729
C -2.66027 5.16387 -0.31166
H -3.37339 5.99160 -0.39005
C -1.50373 5.29564 0.47761
H -1.31645 6.22721 1.02427
C -0.58721 4.23773 0.57685
H 0.30414 4.35444 1.20272
C 1.98182 2.24471 0.20238
C 2.39255 3.43396 -0.45330
C 2.96642 1.51425 0.91643
C 3.72374 3.87236 -0.39163
C 4.29816 1.95036 0.97333
C 4.68574 3.13512 0.32192
H 1.65969 4.01663 -1.02121
H 2.66929 0.59278 1.43027
H 4.01337 4.79446 -0.90899
H 5.03567 1.36942 1.53946
H 5.72377 3.48117 0.37145

trans-III
BP86
SCF = -1408.52035530
H(0 K)= -1407.977537
H(298 K)= -1407.939271
G(298 K)= -1408.049881
SCF(D3BJ) = -1408.68719992
SCF(BS2) = -3081.31672031
Low Freq. = 10.6523cm-1, 16.7634cm-1
B97D
SCF(C6H6,BS2)= -3080.84342075

67
trans-III
Ni 0.00232 -1.14258 0.02652
P 0.01318 0.88484 -1.03321
N -2.69124 -2.04938 -0.67385
N -2.72615 -0.81038 1.09466
N 2.78428 -1.47495 -0.93717
N 2.65480 -1.44381 1.21872
C -1.86851 -1.31215 0.14629
C -4.03091 -2.00280 -0.25463
C -4.05172 -1.21443 0.87284
C -2.20507 -2.79032 -1.83156
H -1.10526 -2.78475 -1.78028
H -2.53011 -2.31857 -2.77475
H -2.56757 -3.83138 -1.80408
C -2.30120 0.06727 2.17836
H -1.22803 0.26286 2.03330
H -2.46313 -0.41164 3.15940
H -2.84773 1.02316 2.13732
C -5.13025 -2.71214 -0.97832
H -5.21539 -2.38094 -2.02937
H -6.09680 -2.51507 -0.48941
H -4.98257 -3.80771 -0.99107
C -5.18139 -0.78512 1.75245
H -5.03455 -1.10074 2.80147
H -6.12731 -1.22667 1.40259
H -5.30424 0.31313 1.75207
C 1.87845 -1.32482 0.08811
C 4.08932 -1.68378 -0.46046
C 4.00747 -1.65688 0.91193
C 5.06276 -1.79699 1.96150
H 5.12991 -0.89910 2.60256
H 6.04815 -1.94344 1.49299
H 4.87917 -2.66172 2.62526
C 5.26157 -1.87071 -1.36920
H 5.13473 -2.74215 -2.03704
H 6.17694 -2.03530 -0.78004
H 5.43393 -0.98723 -2.01078
C 2.42139 -1.49041 -2.35075
H 2.49897 -2.51098 -2.76574
H 3.07951 -0.81745 -2.92336

H   1.38485 -1.13232 -2.43613
C   2.11279 -1.37398 2.56889
H   1.01526 -1.38599 2.47713
H   2.43283 -0.45047 3.08186
H   2.43734 -2.24538 3.16178
H   0.00664 -2.56672 0.49916
C -1.42674 1.98175 -0.57061
C -2.71446 1.62551 -1.05079
H -2.82498 0.71953 -1.65856
C -3.84481 2.40709 -0.77352
H -4.82290 2.10023 -1.16280
C -3.72635 3.58807 -0.01663
H -4.60625 4.20615 0.19229
C -2.45730 3.96883 0.45140
H -2.34247 4.89029 1.03498
C -1.32729 3.17903 0.18169
H -0.35088 3.49649 0.56190
C   1.44893 1.96808 -0.47704
C   2.10755 2.76745 -1.43998
C   1.92845 2.01790 0.85325
C   3.20142 3.57972 -1.09376
C   3.01997 2.82954 1.20711
C   3.66251 3.61217 0.23255
H   1.75092 2.74845 -2.47658
H   1.43017 1.41109 1.61711
H   3.69296 4.18898 -1.86097
H   3.36886 2.85362 2.24630
H   4.51512 4.24357 0.50589

H2
BP86
SCF =   -1.17652517490
H(0 K)=   -1.166600
H(298 K)=   -1.163295
G(298 K)=   -1.178106
SCF(D3BJ) =   -1.17663517433
SCF(BS2) =   -1.17765088142
Low Freq. = 4356.7974cm-1, cm-1
B97D
SCF(C6H6,BS2)= -1.18311896779

2
H2
H   0.00000 0.00000 0.37521
H   0.00000 0.00000 -0.37521

PPh2H
BP86
SCF =   -470.455510663
H(0 K)=   -470.270042
H(298 K)=   -470.257650
G(298 K)=   -470.310184
SCF(D3BJ) =   -470.505672172
SCF(BS2) =   -805.447399928
Low Freq. = 16.1061cm-1, 24.0850cm-1
B97D
SCF(C6H6,BS2)= -805.068371137

24
PPh2H
P   0.05670 1.68765 -0.29616
C   1.46992 0.45929 -0.14158
C   1.37306 -0.90155 -0.50042
C   2.69109 0.94784 0.36804
H   0.42569 -1.29562 -0.88332
H   2.77148 2.00005 0.66492
C   2.48046 -1.75238 -0.36136
C   3.80232 0.09724 0.49958
H   2.39382 -2.80746 -0.64302
H   4.74547 0.49029 0.89396
C   3.69788 -1.25441 0.13586
H   4.56018 -1.92081 0.24379
C -1.43992 0.59449 -0.08545
C -2.19257 0.10320 -1.17471
C -1.87262 0.29838 1.22715
H -1.87590 0.32576 -2.19958
H -1.30993 0.68479 2.08488
C -3.34597 -0.66933 -0.95623
C -3.01571 -0.48541 1.44413
H -3.92005 -1.04096 -1.81182
H -3.33391 -0.71119 2.46749
C -3.75720 -0.96905 0.35236
H -4.65414 -1.57381 0.52189
H   0.00239 1.72430 -1.74093

cis-V
BP86
SCF =   -1877.79698105
H(0 K)=   -1877.083146
H(298 K)=   -1877.032649
G(298 K)=   -1877.170346
SCF(D3BJ) =   -1878.05291557
SCF(BS2) =   -3885.58062525
Low Freq. = 12.8659cm-1, 14.3707cm-1
B97D
SCF(C6H6,BS2)= -3884.75020681

89
cis-V
C -1.39374 3.10120 1.51398
C -1.62174 2.94405 0.12790
C -1.93630 4.09809 -0.62643
C -2.03520 5.36051 -0.01951
C -1.81149 5.49701 1.36095
C -1.48919 4.36398 2.12521
P -1.55751 1.29369 -0.77099
C -3.36158 0.81106 -0.59882
C -3.90134 -0.08079 -1.55962
C -5.24953 -0.46727 -1.52737
C -6.10932 0.04144 -0.53752
C -5.59844 0.94152 0.41316
C -4.24586 1.32059 0.38333
Ni -0.08245 -0.28716 -0.08985
C -1.46304 -1.60246 0.10691
N -2.22633 -1.78318 1.23883
C -3.10076 -2.87270 1.11909
C -2.88023 -3.40754 -0.12869
N -1.87801 -2.62057 -0.72026
C -2.16099 -0.90874 2.40530
C -1.38038 -2.82911 -2.07443
C   1.30350 -1.54509 -0.46627
N   2.04449 -1.57339 -1.62710

```
C   2.95499 -2.64072 -1.64592
C   2.78204 -3.31387 -0.45927
N   1.76612 -2.63446  0.23325
C   1.90188 -0.59596 -2.70166
C   1.29611 -3.01831  1.55910
P   1.40582  1.19670  0.80053
C   2.99706  0.32524  1.29691
C   4.15989  0.20537  0.49720
C   5.30808 -0.44725  0.97318
C   5.32775 -1.00538  2.26394
C   4.18675 -0.89269  3.07691
C   3.04348 -0.23018  2.59891
C   2.11646  2.52094 -0.31929
C   1.51559  2.89282 -1.54403
C   2.00713  3.96961 -2.30136
C   3.11929  4.70282 -1.85869
C   3.72865  4.35091 -0.64050
C   3.23075  3.28302  0.11923
C  -4.05489 -3.26441  2.20102
C  -3.51524 -4.56777 -0.82544
C   3.48502 -4.50689  0.10359
C   3.88587 -2.88786 -2.78926
H   0.46242 -2.35323  1.82031
H   2.10007 -2.89798  2.30320
H   0.94591 -4.06431  1.55825
H   1.64992 -1.09527 -3.65281
H   2.83157 -0.01863 -2.83294
H   1.09060  0.09211 -2.41889
H  -1.92721 -1.48693  3.31516
H  -3.11740 -0.37805  2.54160
H  -1.36261 -0.17387  2.21738
H  -0.66331 -2.02744 -2.29321
H  -2.20821 -2.78334 -2.80177
H  -0.87791 -3.80710 -2.16539
H   4.16967  0.64781 -0.50451
H   6.19515 -0.51965  0.33280
H   6.22743 -1.50668  2.63712
H   4.19434 -1.30094  4.09445
H   2.16878 -0.11903  3.25195
H   0.64257  2.33574 -1.90491
H   1.51600  4.23224 -3.24576
H   3.50446  5.54072 -2.44993
H   4.59243  4.91795 -0.27424
H   3.71190  3.03386  1.07146
H  -3.25097 -0.45299 -2.36050
H  -5.63732 -1.15171 -2.29122
H  -7.16573 -0.24747 -0.51780
H  -6.25872  1.35823  1.18314
H  -3.87092  2.03277  1.12613
H  -2.11023  3.99811 -1.70430
H  -2.28308  6.23903 -0.62595
H  -1.88376  6.48116  1.83692
H  -1.30813  4.46109  3.20188
H  -1.12333  2.22804  2.11462
H   4.50201 -3.77875 -2.59284
H   3.34458 -3.05940 -3.73763
H   4.57256 -2.03785 -2.95342
H   4.22661 -4.88602 -0.61634
H   4.02299 -4.25944  1.03665
H   2.78937 -5.33488  0.33186
H  -4.04061 -4.25928 -1.74756
H  -2.77679 -5.33939 -1.11007
H  -4.25758 -5.04571 -0.16798
H  -4.62248 -4.15958  1.90377
H  -3.53667 -3.49713  3.14905
H  -4.78452 -2.46197  2.41153
```

trans-V

```
BP86
SCF  =            -1877.80863912
H(0 K)=           -1877.094551
H(298 K)=         -1877.044055
G(298 K)=         -1877.182931
SCF(D3BJ) =       -1878.06675298
SCF(BS2) =        -3885.59065567
Low Freq. = 11.5821cm-1, 14.6106cm-1
B97D
SCF(C6H6,BS2)=  -3884.76359426
```

89

trans-V

```
Ni  0.00000  0.00002  0.46150
P  -0.69753 -2.16029  0.85244
N  -2.62758  0.68317 -0.69585
N  -2.55312  1.17386  1.40581
N   2.55314 -1.17372  1.40586
N   2.62756 -0.68319 -0.69583
C  -1.79120  0.63682  0.39328
C  -3.87567  1.23681 -0.37982
C  -3.83056  1.54764  0.95852
C  -2.27058  0.17453 -2.01273
H  -1.25713 -0.24735 -1.93039
H  -2.27584  0.98423 -2.76152
H  -2.96699 -0.62279 -2.31871
C  -2.12496  1.26710  2.79851
H  -1.05660  1.01604  2.84091
H  -2.69860  0.56609  3.43010
H  -2.26493  2.29194  3.17788
C  -4.96554  1.39677 -1.38961
H  -4.65069  2.02709 -2.24085
H  -5.84320  1.87581 -0.92904
H  -5.29272  0.42329 -1.79737
C  -4.85547  2.15981  1.85791
H  -5.11715  1.49647  2.70229
H  -5.78062  2.36087  1.29615
H  -4.51193  3.11882  2.28667
C   1.79120 -0.63677  0.39330
C   3.83056 -1.54757  0.95857
C   3.87565 -1.23684 -0.37980
C   4.96550 -1.39690 -1.38960
H   4.65061 -2.02727 -2.24078
H   5.84315 -1.87591 -0.92900
H   5.29268 -0.42345 -1.79744
C   4.85548 -2.15970  1.85798
H   5.11720 -1.49629  2.70230
H   5.78061 -2.36084  1.29621
H   4.51192 -3.11866  2.28683
C   2.12500 -1.26686  2.79857
H   2.69875 -0.56589  3.43012
H   2.26485 -2.29169  3.17799
H   1.05668 -1.01566  2.84099
C   2.27056 -0.17459 -2.01273
H   1.25712  0.24729 -1.93041
```

```
H   2.27583 -0.98432 -2.76149
H   2.96697  0.62272 -2.31874
P   0.69755  2.16034  0.85230
C   0.18441 -3.38092 -0.27950
C   0.61638 -4.60681  0.27690
C   0.44847 -3.15579 -1.65073
C   1.28390 -5.56907 -0.50063
C   1.11089 -4.11528 -2.43556
C   1.53354 -5.32621 -1.86103
H   0.41951 -4.80582  1.33676
H   0.12368 -2.21468 -2.10713
H   1.60639 -6.51114 -0.04283
H   1.29502 -3.91884 -3.49838
H   2.05173 -6.07479 -2.47019
C   2.47372  2.50127  0.36269
C   2.88994  3.18697 -0.80439
C   3.48238  2.10391  1.27717
H   2.14068  3.53164 -1.52452
H   3.18824  1.60853  2.21030
C   4.24873  3.44680 -1.05161
C   4.83959  2.35079  1.02700
H   4.53754  3.98671 -1.96131
H   5.59294  2.03211  1.75676
C   5.23384  3.02510 -0.14293
H   6.29221  3.23189 -0.33482
C  -2.47371 -2.50127  0.36292
C  -3.48234 -2.10382  1.27740
C  -2.88997 -3.18711 -0.80406
H  -3.18816 -1.60835  2.21047
H  -2.14074 -3.53186 -1.52418
C  -4.83956 -2.35073  1.02731
C  -4.24877 -3.44697 -1.05120
H  -5.59288 -2.03198  1.75707
H  -4.53761 -3.98698 -1.96083
C  -5.23385 -3.02518 -0.14253
H  -6.29222 -3.23199 -0.33436
C  -0.18441  3.38091 -0.27970
C  -0.61627  4.60687  0.27659
C  -0.44857  3.15565 -1.65090
H  -0.41932  4.80598  1.33643
H  -0.12387  2.21447 -2.10722
C  -1.28380  5.56909 -0.50099
C  -1.11100  4.11508 -2.43578
H  -1.60621  6.51122 -0.04326
H  -1.29522  3.91855 -3.49857
C  -1.53354  5.32610 -1.86134
H  -2.05174  6.07464 -2.47055
```

TS(IV-cis-V)

```
BP86
SCF  =          -1878.94186855
H(0 K)=         -1878.213963
H(298 K)=       -1878.163301
G(298 K)=       -1878.301055
SCF(D3BJ) =     -1879.20183812
SCF(BS2) =      -3886.72430222
Low Freq. = -675.3042cm-1,
15.1616cm-1
B97D
SCF(C6H6,BS2)=  -3885.89623656

91
```

TS(IV-cis-V)

```
C  -3.44685 -2.56500  0.94306
N  -2.44654 -2.09840  0.07915
C  -1.58322 -1.22317  0.70272
N  -2.07287 -1.16792  1.98890
C  -3.20993 -1.97168  2.16070
C  -1.49542 -0.37252  3.06964
C  -2.37108 -2.48883 -1.32490
Ni -0.08898 -0.25929 -0.17101
P  -1.35790  1.67582  0.09839
P   1.67413  0.28603  1.42802
C   0.90492 -1.89944 -0.60938
N   1.47026 -2.24605 -1.81826
C   2.05927 -3.52046 -1.77731
C   1.85209 -4.00012 -0.50617
N   1.15368 -2.99776  0.18386
C   1.51342 -1.38414 -2.99806
C   0.74280 -3.13166  1.58087
H   1.78318 -1.98743 -3.87567
H   2.25858 -0.58419 -2.86214
H   0.52715 -0.92360 -3.15639
H   0.71379 -2.13009  2.03215
H   1.48355 -3.74133  2.11906
H  -0.24662 -3.61381  1.66074
C  -4.52119 -3.50831  0.50587
C  -3.94137 -2.08037  3.46043
H  -2.10936  0.52099  3.27174
H  -0.48470 -0.06334  2.76656
H  -1.42660 -0.97982  3.98661
H  -1.55907 -1.90732 -1.78223
H  -3.31857 -2.25353 -1.83545
H  -2.16131 -3.56808 -1.41943
C   1.90671  2.09167  1.82895
H   0.61929  0.48399 -1.21057
H  -0.50633 -0.03644 -1.58979
C   3.29234 -0.08560  0.54129
C  -3.05273  1.24041 -0.61077
C  -0.81153  2.95786 -1.15841
C   2.77024 -4.14128 -2.93795
C   2.25421 -5.29702  0.12124
H  -4.30433 -1.09832  3.81270
H  -4.81750 -2.73734  3.34837
H  -3.30984 -2.50342  4.26296
H  -5.15392 -3.06939 -0.28660
H  -4.11003 -4.45593  0.11331
H  -5.17707 -3.75738  1.35417
H   1.38769 -5.85407  0.52047
H   2.74605 -5.94051 -0.62413
H   2.96751 -5.15378  0.95334
H   3.63343 -3.53560 -3.26675
H   3.15383 -5.13345 -2.65458
H   2.10864 -4.28252 -3.81213
C  -3.29664  0.87675 -1.95590
C  -4.59142  0.56734 -2.40096
C  -5.67602  0.60435 -1.50457
C  -5.45372  0.96310 -0.16603
C  -4.15530  1.28387  0.27069
H  -2.45662  0.84135 -2.65867
H  -4.75727  0.29813 -3.45073
H  -6.68682  0.36363 -1.85182
H  -6.29232  1.00640  0.53815
H  -3.99159  1.58444  1.31257
```

```
C  -1.73286  3.73849 -1.89830
C  -1.29763  4.78915 -2.72341
C   0.06839  5.09684 -2.82467
C   0.99438  4.34582 -2.07945
C   0.56068  3.29491 -1.25795
H  -2.80398  3.52437 -1.83221
H  -2.03544  5.36913 -3.29010
H   0.40717  5.91482 -3.46971
H   2.06335  4.58191 -2.13241
H   1.29111  2.73486 -0.66575
C   3.10903  2.81501  1.61344
C   3.25742  4.13730  2.06082
C   2.21051  4.78966  2.73302
C   1.01108  4.09174  2.95714
C   0.86390  2.76766  2.52040
H   3.94498  2.33409  1.09670
H   4.20376  4.66113  1.88000
H   2.32687  5.82273  3.07766
H   0.17984  4.58126  3.47799
H  -0.08034  2.24928  2.71082
C   3.65524  0.47162 -0.70854
C   4.88133  0.16023 -1.31912
C   5.77310 -0.73392 -0.69948
C   5.43028 -1.30193  0.53744
C   4.20786 -0.97246  1.15111
H   2.96375  1.16277 -1.20378
H   5.14207  0.61650 -2.28141
H   6.72873 -0.97955 -1.17557
H   6.12053 -1.99327  1.03450
H   3.95756 -1.40013  2.12909
```

VI

```
BP86
SCF  =            -1877.78711902
H(0 K)=           -1877.074864
H(298 K)=         -1877.023960
G(298 K)=         -1877.164471
SCF(D3BJ) =       -1878.03351452
SCF(BS2) =        -3885.57449300
Low Freq. = 9.0175cm-1, 15.9018cm-1
B97D
SCF(C6H6,BS2)=  -3884.73760837
```

89

VI

```
C   1.09319  3.27110 -3.90544
C   1.12977  1.90596 -4.23167
C   1.05951  0.93094 -3.22164
C   0.94517  1.30194 -1.86179
C   0.89277  2.68172 -1.54793
C   0.97639  3.65355 -2.55623
P   0.61183  0.03444 -0.51791
C   1.54470 -1.47738 -1.15203
C   2.77296 -1.44860 -1.85162
C   3.37774 -2.64145 -2.28054
C   2.77783 -3.88158 -2.00155
C   1.56456 -3.92338 -1.29469
C   0.95150 -2.72960 -0.88029
Ni -1.47317  0.02722  0.03422
C  -2.10037  1.77879  0.49658
N  -2.09193  2.48719  1.69021
C  -2.64905  3.77027  1.56291
C  -3.02404  3.89731  0.24583
N  -2.68752  2.68519 -0.37518
C  -1.55534  1.95732  2.93483
C  -2.89652  2.39548 -1.78695
C  -2.76039  4.72411  2.70932
C  -3.66185  5.03583 -0.48456
C  -2.51008 -1.53344 -0.27096
N  -3.25065 -2.27585  0.64292
C  -3.92785 -3.35248  0.04850
C  -3.62540 -3.30254 -1.29235
N  -2.76552 -2.20596 -1.46166
C  -3.31385 -1.94184  2.05681
C  -2.22533 -1.78005 -2.74387
C  -4.78091 -4.30572  0.82347
C  -4.05873 -4.17734 -2.42540
H  -2.90776 -0.92268  2.16451
H  -2.71434 -2.63800  2.67066
H  -4.35679 -1.96171  2.41635
H  -1.46256 -1.01300 -2.53597
H  -3.00973 -1.34491 -3.38937
H  -1.75691 -2.62635 -3.27379
H  -4.21168 -4.81888  1.62058
H  -5.18945 -5.08143  0.15692
H  -5.63812 -3.80247  1.30821
H  -4.59799 -3.60956 -3.20605
H  -4.73659 -4.96575 -2.06237
H  -3.20411 -4.67656 -2.91865
H  -3.04095  5.38580 -1.32975
H  -3.81038  5.89038  0.19417
H  -4.65152  4.76598 -0.89782
H  -3.38592  4.32181  3.52780
H  -3.21910  5.66877  2.37741
H  -1.77401  4.96914  3.14474
H  -2.34241  1.86973  3.70519
H  -0.74735  2.60039  3.32380
H  -1.14041  0.96067  2.72494
H  -2.72669  1.31470 -1.91928
H  -2.18367  2.94942 -2.42180
H  -3.92751  2.64768 -2.08906
H   3.25793 -0.49145 -2.06515
H   4.32667 -2.60103 -2.82674
H   3.25461 -4.81014 -2.33441
H   1.08980 -4.88603 -1.07324
H  -0.01158 -2.74684 -0.35542
H   1.10064 -0.12839 -3.49449
H   0.77961  2.98938 -0.50321
H   1.21873  1.59311 -5.27823
H   0.94870  4.71547 -2.28680
H   1.15564  4.03021 -4.69247
P   2.05763  0.92797  1.11182
C   3.81547  0.58824  0.58874
C   1.82709 -0.28518  2.51767
C   2.76587 -0.26438  3.57874
C   2.58184 -1.05423  4.72341
C   1.44779 -1.87636  4.84249
C   0.50379 -1.89752  3.80403
C   0.68828 -1.11235  2.65274
H   3.65239  0.37540  3.50563
H   3.32631 -1.02347  5.52673
H   1.30340 -2.49337  5.73584
H  -0.38293 -2.53702  3.88322
H  -0.06214 -1.13700  1.84612
```

C 4.46589 1.62149 -0.12971
C 5.78271 1.46220 -0.58735
C 6.48301 0.27252 -0.32108
C 5.85029 -0.75921 0.39411
C 4.52697 -0.60938 0.83617
H 3.93053 2.55705 -0.32855
H 6.26745 2.27385 -1.14109
H 7.51527 0.15142 -0.66658
H 6.38728 -1.69155 0.60175
H 4.04122 -1.42358 1.38224

TS(cis-trans-V)
BP86
SCF = -1877.77594510
H(0 K)= -1877.062366
H(298 K)= -1877.012840
G(298 K)= -1877.147035
SCF(D3BJ) = -1878.02687535
SCF(BS2) = -3885.56091100
Low Freq. = -24.7870cm-1,
14.2425cm-1
B97D
SCF(C6H6,BS2)= -3884.72697142

89
TS(cis-trans-V)
C 2.18974 3.78322 -0.92431
C 2.08052 2.88752 0.16604
C 2.10004 3.42949 1.47307
C 2.22367 4.81376 1.68371
C 2.32428 5.68798 0.58843
C 2.30787 5.16786 -0.71704
P 1.85807 1.04616 -0.14259
C 3.64742 0.54627 -0.20930
C 3.99954 -0.68097 -0.83088
C 5.33069 -1.11624 -0.88513
C 6.35978 -0.33637 -0.32735
C 6.03305 0.88753 0.28193
C 4.70124 1.32370 0.33956
Ni 0.11166 -0.25765 -0.07250
C 1.02189 -2.07293 -0.20028
N 1.24525 -2.94420 -1.25259
C 2.02183 -4.05462 -0.88239
C 2.29880 -3.89801 0.45578
N 1.68043 -2.70116 0.84242
C 0.82238 -2.69972 -2.62818
C 1.78085 -2.14517 2.18741
C -1.17389 -0.04789 1.30879
N -1.62519 1.08809 1.95545
C -2.54078 0.79383 2.97516
C -2.69050 -0.57277 2.97689
N -1.85732 -1.06203 1.96219
C -1.21952 2.44839 1.62830
C -1.79313 -2.47777 1.61643
P -1.49716 -0.07582 -1.63807
C -3.00854 -1.11422 -1.26452
C -4.04625 -0.73930 -0.37557
C -5.12408 -1.60029 -0.11433
C -5.18968 -2.86413 -0.72972
C -4.17052 -3.25344 -1.61527
C -3.09797 -2.38599 -1.87954
C -2.24519 1.61887 -1.85262
C -1.37124 2.73580 -1.83116
C -1.81991 4.02180 -2.16277
C -3.16053 4.23322 -2.53087
C -4.03873 3.13669 -2.57451
C -3.58907 1.84834 -2.24554
C 2.40098 -5.13837 -1.84080
C 3.07653 -4.75499 1.40286
C -3.53943 -1.46448 3.82495
C -3.16028 1.84913 3.83460
H -1.06568 -2.58434 0.80054
H -2.78055 -2.83331 1.27888
H -1.46768 -3.07218 2.48739
H -0.73695 2.92926 2.49507
H -2.08961 3.04800 1.31522
H -0.50213 2.39144 0.80196
H 0.22117 -3.54411 -3.00585
H 1.69920 -2.56805 -3.28602
H 0.21635 -1.78115 -2.64335
H 1.18821 -1.21918 2.20618
H 2.82938 -1.90275 2.42901
H 1.39442 -2.85853 2.93549
H -4.00582 0.23755 0.11739
H -5.91747 -1.28371 0.57266
H -6.03193 -3.53434 -0.52677
H -4.21593 -4.23021 -2.10997
H -2.31639 -2.69273 -2.58420
H -0.32355 2.58173 -1.54755
H -1.11785 4.86253 -2.12699
H -3.51520 5.23804 -2.78446
H -5.08413 3.28278 -2.87058
H -4.28844 1.00866 -2.30577
H 3.21242 -1.28807 -1.28687
H 5.56817 -2.06594 -1.37848
H 7.40095 -0.67296 -0.37524
H 6.82274 1.51171 0.71635
H 4.47335 2.28117 0.81813
H 2.02270 2.75435 2.33331
H 2.24296 5.20982 2.70554
H 2.41651 6.76720 0.75109
H 2.39254 5.84128 -1.57743
H 2.18449 3.38644 -1.94609
H -3.84801 1.39229 4.56287
H -2.40256 2.41820 4.40351
H -3.73914 2.57991 3.24130
H -4.11253 -0.86731 4.55087
H -4.26232 -2.03877 3.21759
H -2.93760 -2.19479 4.39607
H 3.93403 -4.21227 1.83996
H 2.45535 -5.11731 2.24255
H 3.47455 -5.63864 0.88034
H 3.01792 -5.89688 -1.33447
H 1.51581 -5.65346 -2.25705
H 2.98468 -4.75171 -2.69578

S-4 References

[1] a) R. A. Schunn, S. D. Ittel, M. A. Cushing, R. Baker, R. J. Gilbert, D. P. Madden, *Inorg. Synth.* **1990**, *28*, 94-98; b) J. W. Wielandt, D. Ruckerbauer, T. Zell, U. Radius, *Inorg. Synth.* **2010**, *35*, 120-125.

[2] a) T. Schaub, U. Radius, *Chem. Eur. J.* **2005**, *11*, 5024-5030; b) T. Schaub, M. Backes, U. Radius, *Organometallics* **2006**, *25*, 4196-4206.

[3] a) M. B. Ansell, D. E. Roberts, F. G. N. Cloke, O. Navarro, J. Spencer, *Angew. Chem. Int. Ed.* **2015**, *54*, 5577-5582; b) N. Kuhn, T. Kratz, *Synthesis* **1993**, 561-562.

[4] T. Schaub, U. Radius, A. Brucks, M. P. Choules, M. T. Olsen, T. B. Rauchfuss, *Inorg. Synth.* **2010**, *35*, 78-83.

[5] a) A. Finch, P. J. Gardner, K. K. S. Gupta, *J. Chem. Soc. B: Phys. Org.* **1966**, 1162-1164; b) J. D. Masuda, K. C. Jantunen, O. V. Ozerov, K. J. T. Noonan, D. P. Gates, B. L. Scott, J. L. Kiplinger, *J. Am. Chem. Soc.* **2008**, *130*, 2408-2409.

[6] H. Schneider, D. Schmidt, U. Radius, *Chem. Commun.* **2015**, *51*, 10138-10141.

[7] G. M. Sheldrick, *Acta Cryst.* **2008**, *A64*, 112-122.

[8] O. V. Dolomanov, L. J. Bourhis, R. J. Gildea, J. A. K. Howard, H. Puschmann, *J. Appl. Cryst.* **2009**, *42*, 339-341.

[9] L.J. Bourhis, O. V. Dolomanov, R. J. Gildea, J. A. K. Howard, H. Puschmann, *Acta Cryst.* **2015**, *A71*, 59-75.

[10] M. J. Frisch, G. W. Trucks, H. B. Schlegel, G. E. Scuseria, M. A. Robb, J. R. Cheeseman, G. Scalmani, V. Barone, B. Mennucci, G. A. Petersson, H. Nakatsuji, M. Caricato, X. Li, H. P. Hratchian, A. F. Izmaylov, J. Bloino, G. Zheng, J. L. Sonnenberg, M. Hada, M. Ehara, K. Toyota, R. Fukuda, J. Hasegawa, M. Ishida, T. Nakajima, Y Honda, O Kitao, H. Nakai, T. Vreven, J. A. Montgomery, J. E. Peralta, F. Ogliaro, M. Bearpark, J. J. Heyd, E. Brothers, K. N. Kudin, V. N Staroverov, R. Kobayashi, J. Normand, K. Raghavachari, A. Rendell, J. C. Burant, S. S. Iyengar, J. Tomasi, M. Cossi, N. Rega, J. M. Millam, M.

Klene, J. E. Knox, J. B. Cross, V. Bakken, C. Adamo, J. Jaramillo, R. Gomperts, R. E. Stratmann, O. Yazyev, A. J. Austin, R. Cammi, C. Pomelli, J. W. Ochterski, R. L. Martin, K. Morokuma, V. G. Zakrzewski, G. A. Voth, P. Salvador, J. J. Dannenberg, S. Dapprich, A. D. Daniels, Ö. Farkas; J. B. Foresman, J. V. Ortiz, J. Cioslowski, D. J. Fox, Gaussian 09, Revision D.01, Gaussian Inc.: Wallingford CT, **2013**.

[11] A. D. Becke, *Phys. Rev. A* **1988**, *38*, 3098-3100.

[12] J. P. Perdew, *Phys. Rev. B* **1986**, *33*, 8822-8824.

[13] D. Andrae, U. Häußermann, M. Dolg, H. Stoll, H. Preuß, *Theor. Chim. Acta* **1990**, *77*, 123-141.

[14] W. J. Hehre, R. Ditchfield, J. A Pople, *J. Chem. Physics* **1972**, *56*, 2257-2261.

[15] P. C. Hariharan, J. A. Pople, *Theor. Chim. Acta* **1973**, *28*, 213-222.

[16] A. Höllwarth, M. Böhme, S. Dapprich, A. W. Ehlers, A. Gobbi, V. Jonas, K. F. Köhler, R. Stegmann, A. Veldkamp, G. Frenking, *Chem. Phys. Lett.* **1993**, *208*, 237-240.

[17] L. J. L. Häller, M. J. Page, S. Erhardt, S. A. Macgregor, M. F. Mahon, M. A. Naser, A. Vélez, M. K. Whittlesey, *J. Am. Chem. Soc.* **2010**, *132*, 18408-18416.

[18] S. Grimme, *J. Comp. Chem.* **2006**, *27* 1787-1799.

[19] F. Weigend, R. Ahlrichs, *Phys. Chem. Chem. Phys.* **2005**, *7*, 3297-3305.

[20] F. Weigend, *Phys. Chem. Chem. Phys.* **2006**, *8*, 1057-1065.

[21] J. Tomasi, B. Mennucci, R. Cammi, *Chem. Rev.* **2005**, *105*, 2999-3093.

[22] C. Lee, W. Yang, R. G. Parr, *Phys. Rev. B* **1988**, *37,* 785-789.

[23] A. D. Becke, *J. Chem. Phys*. **1993**, *98*, 5648-5652.

[24] J. P. Perdew, K. Burke, M. Ernzerhof, *Phys. Rev. Lett.* **1996**, *77* , 3865-3868.

[25] C. Adamo, V. Barone, *J. Chem. Phys.* **1999**, *110,* 6158-69.

[26] S. Grimme, S. Ehrlich, L. Goerigk, *J. Comp. Chem.* **2011**, *32,* 1456-1465

[27] J.-D. Chai, M. Head-Gordon, *Phys. Chem. Chem. Phys*. **2008**, *10*, 6615-6620.

[28] Y. Zhao, D. G. Truhlar, *Theor. Chem. Acc*. **2008**, *120*, 215-241.

[29] J. M. Tao, J. P. Perdew, V. N. Staroverov, G. E. Scuseria, *Phys. Rev. Lett.* **2003**, *91*, 146401.

[30] Chemcraft - graphical software for visualization of quantum chemistry computations. https://www.chemcraftprog.com
